# Human Estrogen Receptor Alpha Antagonists, Part 3: 3-D Pharmacophore and 3-D QSAR Guided Brefeldin A Hit-to-Lead Optimization toward New Breast Cancer Suppressants

**DOI:** 10.3390/molecules27092823

**Published:** 2022-04-28

**Authors:** Nezrina Kurtanović, Nevena Tomašević, Sanja Matić, Elenora Proia, Manuela Sabatino, Lorenzo Antonini, Milan Mladenović, Rino Ragno

**Affiliations:** 1 Kragujevac Center for Computational Biochemistry, Department of Chemistry, Faculty of Science, University of Kragujevac, Radoja Domanovića 12, P.O. Box 60, 34000 Kragujevac, Serbia; nezrina.mihovic@pmf.kg.ac.rs (N.K.); nevena.stankovic@pmf.kg.ac.rs (N.T.); 2 Institute for Informational Technologies Kragujevac, University of Kragujevac, Jovana Cvijića bb, 34000 Kragujevac, Serbia; sanjamatic@kg.ac.rs; 3 Rome Center for Molecular Design, Department of Drug Chemistry and Technology, Faculty of Pharmacy and Medicine, Sapienza University of Rome, P.le A. Moro 5, 00185 Rome, Italy; eleonora.proia@uniroma1.it (E.P.); manuela.sabatino@uniroma1.it (M.S.); lorenzo.antonini@uniroma1.it (L.A.)

**Keywords:** breast cancer, estrogen receptor α, structure-based 3-D pharmacophores, structure-based 3-D QSAR, brefeldin a derivatives synthesis, anticancer activity in vitro and in vivo

## Abstract

The estrogen receptor α (ERα) is an important biological target mediating 17β-estradiol driven breast cancer (BC) development. Aiming to develop innovative drugs against BC, either wild-type or mutated ligand-ERα complexes were used as source data to build structure-based 3-D pharmacophore and 3-D QSAR models, afterward used as tools for the virtual screening of National Cancer Institute datasets and hit-to-lead optimization. The procedure identified Brefeldin A (**BFA**) as hit, then structurally optimized toward twelve new derivatives whose anticancer activity was confirmed both in vitro and in vivo. Compounds as SERMs showed picomolar to low nanomolar potencies against ERα and were then investigated as antiproliferative agents against BC cell lines, as stimulators of p53 expression, as well as BC cell cycle arrest agents. Most active leads were finally profiled upon administration to female Wistar rats with pre-induced BC, after which **3DPQ-12**, **3DPQ-3**, **3DPQ-9**, **3DPQ-4**, **3DPQ-2**, and **3DPQ-1** represent potential candidates for BC therapy.

## 1. Introduction

**Figure 1:**
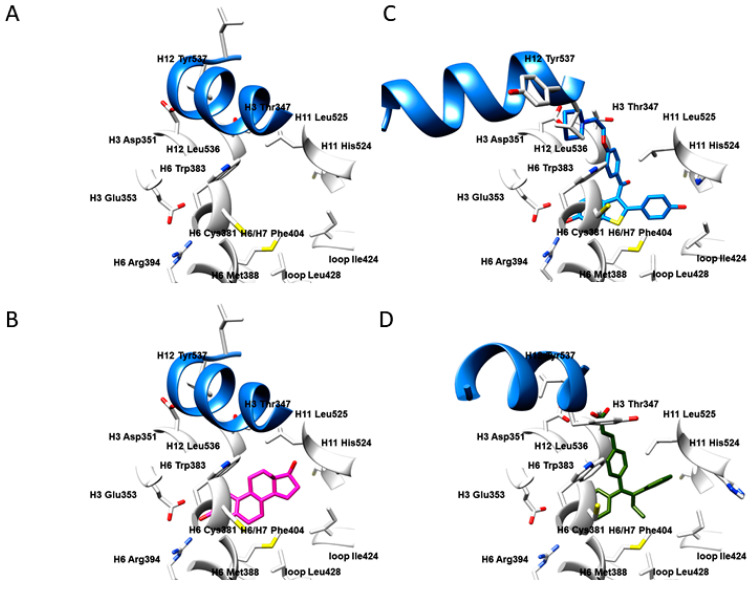
The active site of ERα in the apo form (PDB ID: **4Q13** [21]) (**A**); in complex with 17β-estradiol (PDB ID: **1ERE** [13], i.e., agonist/partial agonist) (**B**); in complex with Raloxifene (PDB ID: **1ERR** [13], i.e., SERM antagonist) (**C**); in complex with GW568 (PDB ID: **1R5K** [21], i.e., SERD antagonist) (**D**). The residues depicted as white sticks and ribbons belong to the helices H3 (residues 332–354), H6 (residues 383–394), H7 (residues 429–438), H11 (residues 517–528), H12 (residues 531–547), loop (residues 418–428), and S1 and S2 antiparallel β-sheets (residues 402–410). H12 helix is depicted as a blue ribbon, as a crucial delimiter for partial agonists, SERMs, and SERDs.

**Figure 2:**
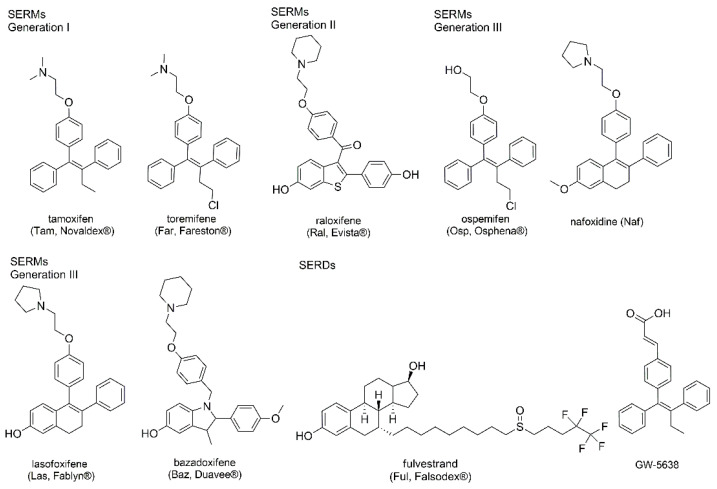
SERMs and SERDs as FDA-approved drugs and compounds in clinical trials for BC treatment.

Estrogen receptor α (ERα) mediates as nuclear receptor (NR) the hormonal breast cancer (BC) development [1,2,3], being stimulated by 17β-estradiol (**E_2_**); the initialization of tumor progression is regulated by either genomic direct or indirect pathway [4,5,6,7,8,9,10,11], as well as by the recruitment of transcriptional basal machinery (TBM) complex (see Supplementary Material: Introduction for further information and references). As there are no known cellular mechanisms to fully suppress BC development in vivo [1], clinical cases are treated with selective estrogen receptor modulators (SERMs, mixed agonists/antagonists of ERα), and selective ERα down-regulators (SERDs, full antagonists of ERα). Both SERMs and SERDs bind the ERα ligand-binding domain (LBD, Figure 1), inducing LBD’s helix 12 (H12) induced fitting, leading to different pharmacological profiles: while SERMs, as non-steroid compounds, prevent the ERα signaling at genomic direct or genomic indirect level, SERDs, as steroid-based drugs, force the rapid downregulation and proteasomal degradation of ERα [12,13,14,15,16,17]. Herein, a simplified representation of LBD, either free or saturated with agonists, SERM, or SERD, respectively, is depicted (Figure 1). So-far FDA-approved SERMs (Figure 2) are tamoxifen (Tam, Nolvadex^®^) and toremifene (Far, Fareston^®^), i.e., the representatives of SERM I generation; raloxifene (Ral, Evista^®^ (Figure 1C), namely a member of the second-generation SERM family); and nafoxidine (Naf), lasofoxifene (Las, Fablyn^®^), ospemifene (Osp, Osphena^®^), and bazadoxifene (Baz, Duavee^®^) (i.e., third-generation SERMs) [16], whereas fulvestrant (Ful, Faslodex^®^) is the only FDA-approved SERD (Glaxo SmithKline’s GW-5538 [1], Figure 1D, has reached clinical trials). Yet, despite indubitable efficacy, long-term treatment with Nolvadex^®^ [17] causes endometrial cancer, Evista^®^ [18] has modest efficacy in advanced BCs, while other SERMs exert transitory clinical effectiveness accompanied by almost-inevitable BC resistance and relapse [19,20]. The defectiveness described encourages the investigation and development of further SERM classes.

**Figure 3:**
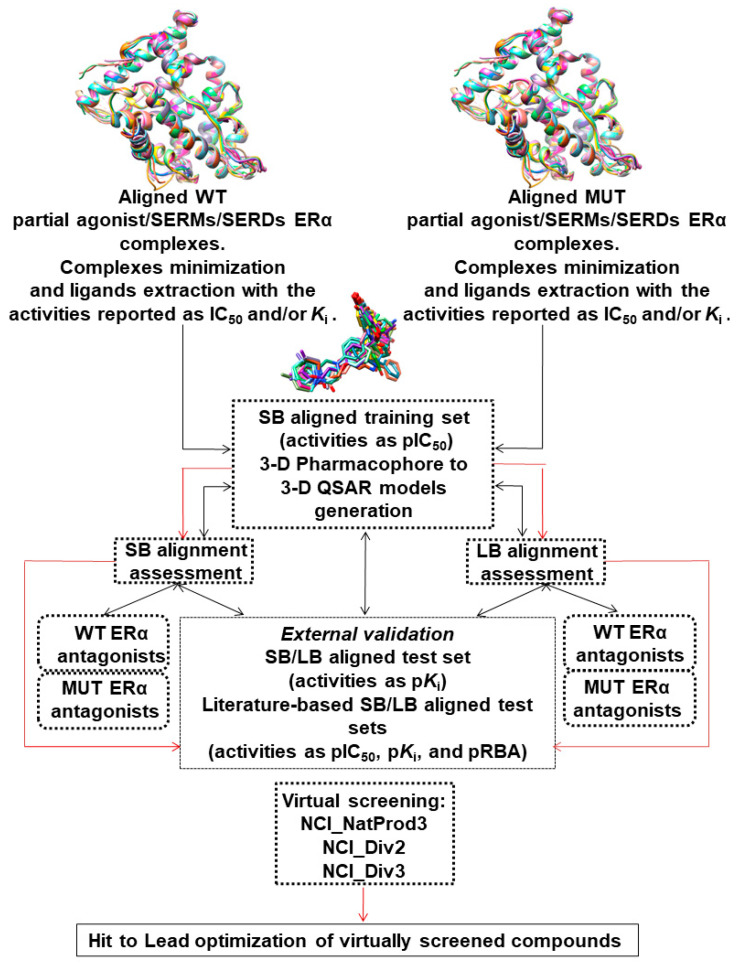
The overall procedure workflow used for the definition of the 3-D pharmacophore/3-D QSAR models and their analysis is depicted as a “black” pathway. The application of generated 3-D pharmacophore/3-D QSAR models in structure-based and ligand-based virtual screening is depicted as a “red” pathway.

Computer-aided drug design (CADD) approaches were extensively used to achieve an understanding of the potency of ERα partial agonists, SERMs, and SERDs through the development of 3-D pharmacophore hypotheses [22,23,24,25,26,27,28,29,30,31,32,33,34,35,36,37,38,39,40,41,42,43,44,45,46,47,48,49,50,51,52,53,54,55,56,57,58] (see Supplementary Materials: ERα 3-D pharmacophore models generation overview). Recently, a list of ERα ligands [13,59,60,61,62,63,64,65,66,67,68,69,70,71,72,73,74,75,76,77,78,79] was investigated to build predictive field-based SB 3-D QSAR models [80] that drove the disclosure of innovative coumarin and coumarin-like SERMs [81]. Herein (Figure 3), partial agonists, SERMs, and SERDs, co-crystalized with either wild-type (WT) or mutated (MUT) ERαs, as found deposited and available from the Protein Data Bank (39 complexes) [13,59,60,61,62,63,64,65,66,67,68,69,70,71,72,73,74,75,76,77,78,79], were retrieved to build structure-based (SB) 3-D pharmacophore models and atom-based 3-D QSAR models [61,62] in order to develop innovative SERMs that would exert no or diminished known side effects [17,18,19,20].

Nonetheless, to the best of the authors’ knowledge, no comprehensive study has yet been conducted to explore all such structural data for generating the SB 3-D pharmacophore models that are generated herein and compared with previous ligand-based (LB) and SB findings [22,23,24,25,26,27,28,29,30,31,32,33,34,35,36,37,38,39,40,41,42,43,44,45,46,47,48,49,50,51,52,53,54,55,56,57,58] (see Supplementary Materials: ERα 3-D pharmacophore models generation overview). The optimal 3-D pharmacophore hypothesis and the associated 3-D QSAR model were applied in a virtual screening (VS) campaign, using the National Institute of Health database, from which Brefeldin A (BFA) was indicated as a suitable hit for hit-to-lead optimization, driving to a series of twelve new BFA derivatives with a potential of being new ERα SERM antagonists (**3DPQ-1** to **3DPQ-12**, Figure 3). The **3DPQ**-derivatives were promptly synthesized and subjected to in vitro and in vivo biological screening. Among them, **3DPQ-12**, **3DPQ-9**, **3DPQ-3**, **3DPQ-4**, **3DPQ-2**, and **3DPQ-1** showed a biological profile as a promising new SERM class of compounds for potential anticancer therapy.

## 2. Results and Discussion

### 2.1. Datasets Compilation

All the available ERαs, co-crystallized with partial agonists, SERMs, and SERDs (PDB accessed in October 2015, see Supplementary Materials: Crystal structures compilation and preparation and Table S1, [13,59,60,61,62,63,64,65,66,67,68,69,70,71,72,73,74,75,76,77,78,79,82,83,84,85,86,87]) were retrieved. Unfortunately, the biological experimental data available for the bound ERα ligands (Supplementary Materials Table S1) revealed a heterogeneous distribution of the associated potencies, expressed as either pIC_50_s (−log[IC_50_]) or p*K*_i_s (−log[K_i_]), and only a few of them with both values. Being higher the number of inhibitors associated with pIC_50_s values, they were used to compile the training set (TR, Table 1 and Table 2) [13,59,60,61,62,63,64,65,66,67,68,69,70,71,72,73,74]. To evaluate the under-building 3-D pharmacophore/3-D QSAR models’ predictive ability, the 13 compounds, characterized by p*K*_i_s values and those with dual potencies (both p*K*_i_s and pIC_50_s), were filed in the crystal test set (TS_CRY_, Table 3) [69,75,76,77,78,79]. To indicate TR and TS_CRY_ ligands, PDB codes as listed in Table 1, Table 2 and Table 3 were used.

Furthermore, 97 known ERα binders, taken from the literature, were used to compile modeled test sets TS_MOD1_, TS_MOD2_, and TS_MOD3_, grouped in agreement with the associated pIC_50_, p*K*_i_, and pRBA values, respectively (Supplementary Materials Tables S10–S15).

### 2.2. 3-D Pharmacophore and 3-D QSAR Modeling and Models’ Interpretation

**Figure 4:**
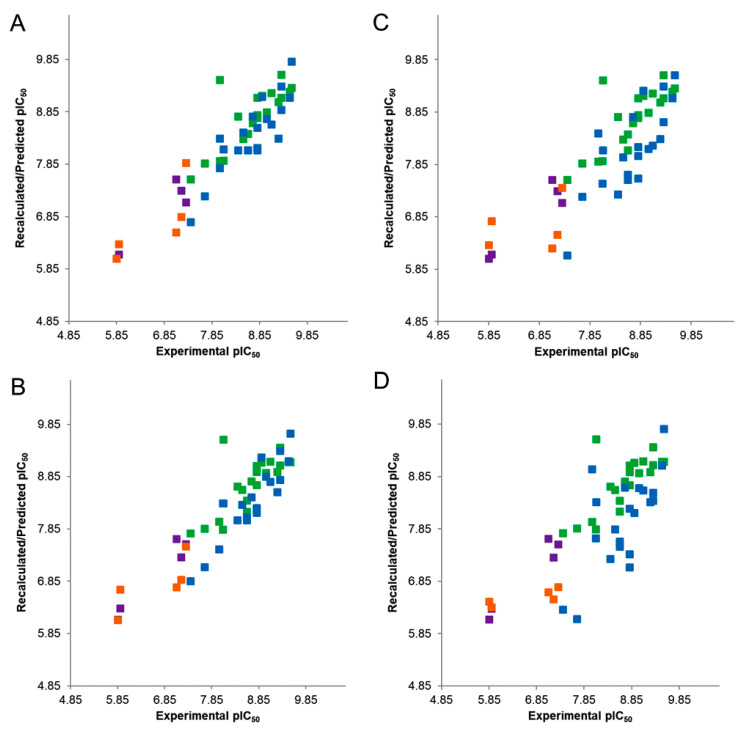
Experimental vs. recalculated (“actives”: green squares; “inactives”: purple squares) and predicted (“actives”: blue squares; “inactives”: orange squares) pIC_50_s for **ADDHHHP.13** hypothesis and LOO cross-validation (**A**); **ADDRRRP.11** hypothesis and LOO cross-validation (**B**); **ADDHHHP.13** hypothesis and LSO cross-validation (**C**); **ADDRRRP.11** hypothesis and LSO cross-validation (**D**).

**Figure 5:**
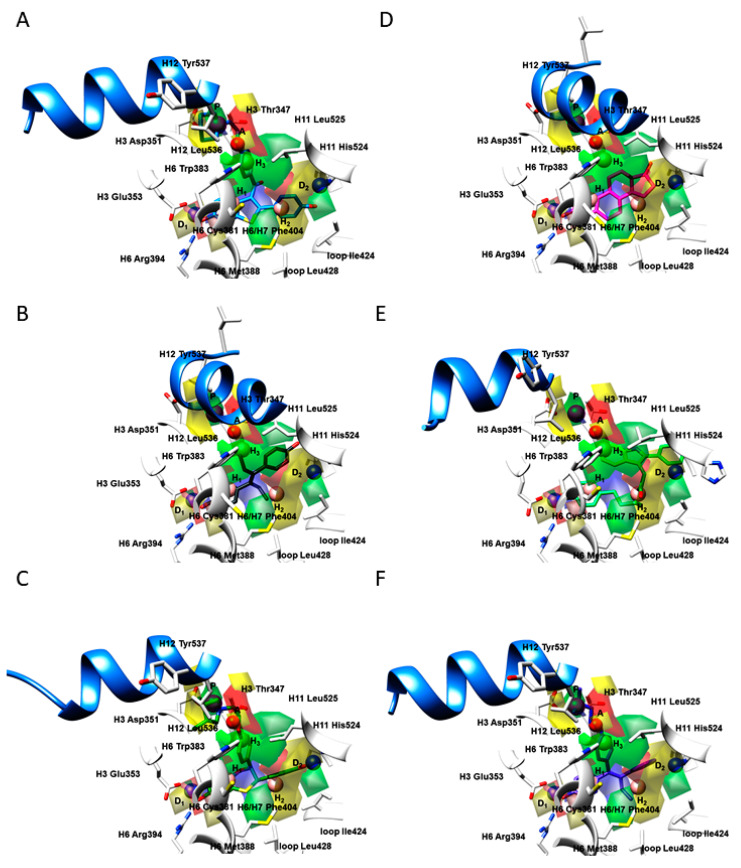
The **3-D PhypI** features (**D**: hydrogen-bond donators, **A**: hydrogen-bond acceptors, **H**: hydrophobic features, **P**: positive ionizable features) and **3-D QSAR** *PLS-coefficients* contour maps (**GREEN*_PLS-coefficients_***: positive steric interactions, **YELLOW*_PLS-coefficients_***; negative steric interactions, **BLUE*_PLS-coefficients_***: areas where positively charged functional groups and H-bond donators are favored whereas the negatively charged functional groups and H-bond acceptors are disfavored, **RED*_PLS-coefficients_***: areas negatively charged functional groups and H-bond acceptors are favored, whereas the positively charged functional groups and H-bond donators are disfavored) for **1ERR** (**A**); **3ERD** (**B**); **1XP1** (**C**); **1ERE** (**D**); **2IOK** (**E**); **2BJ4** (**F**). Amino acid residues are depicted in white. For the clarity of presentation, only the H12 helix is presented in a cornflower blue ribbon, as a crucial delimiter for partial agonists, SERMs, and SERDs.

SB 3-D pharmacophore hypotheses (**3-D Phyp**) and atom-based **3-D QSAR** models were built with the TR using Schrödinger’s PHASE program [88,89] and interpreted as a unique **3-D Phyp**/**3-D QSAR model** ensemble. To derive the best PHASE hypotheses (associated with the highest *q*^2^ values [90,91]), TR molecules were classified into “actives” and “inactives,” using a pIC_50_ threshold value of 7.30, as suggested by the default settings (Table 1 and Table 2). While searching for the optimal **3-D Phyp**/**3-D QSAR model** ensemble, all the available pharmacophoric feature combinations were explored, from which both common pharmacophore hypothesis (CPH) and atom-based 3-D QSAR models were built (top hypotheses are displayed in Supplementary Material Table S2). Based on the highest associated *q*^2^ values, the two best hypotheses were selected, **ADDHHHP.13** and **ADDRRRP.11** (Table 4, Figure 4), herein named **3-D PhypI** and **3-D PhypII**, respectively. Both hypotheses consisted of one hydrogen-bond acceptor (**A**), two hydrogen-bond donators (**D_1_** and **D_2_**), either three hydrophobic (**H_1_**, **H_2_**, and **H_3_**) or aromatic rings (**R_1_**, **R_2_**, and **R_3_**), and one with positively ionizable (**P**) features, which were coupled with the under-developing **3-D QSAR model** *PLS-coefficients* contour maps revealing the areas associated to positive and negative steric (**GREEN***_PLS-coefficients_* and **YELLOW***_PLS-coefficients_*) and HB bonding (**BLUE***_PLS-coefficients_* and **RED***_PLS-coefficients_*) interactions, respectively. Considering that in the PHASE definition, the **H** features are statistically more important, **3-D PhypI** was consequently taken as the base model for the upcoming discussion (Table 4). Only the most important implications of two top hypotheses (Figure 5 and Supplementary Materials Figures S1–S9) on the potency against ERα were presented, whereas the detailed analyses and comparison with previous hypotheses [22,23,24,25,26,27,28,29,30,31,32,33,34,35,36,37,38,39,40,41,42,43,44,45,46,47,48,49,50,51,52,53,54,55,56,57,58] are reported as Supplementary Materials (see the sections The Origin/Significance of the **D_1_** Feature and the Interrelated PLS-coefficients, The Origin/Significance of the **D_2_** Feature and the Interrelated PLS-coefficients, The Origin/Significance of the **H_1_**/**R_1_** Feature and the Interrelated PLS-coefficients, The Origin/Significance of the **H_2_**/**R_2_** Feature and the Interrelated PLS-coefficients, The Origin/Significance of the **H_3_**/**R_3_** Feature and the Interrelated PLS-coefficients, The Origin/Significance of the **A** Feature and the Interrelated PLS-coefficients, and The Origin/Significance of the **P** Feature and the Interrelated PLS-coefficients). For the graphical analysis [80,92,93], either **3-D PhypI** (Figure 5 and Supplementary Materials Figures S1–S4) or **3-D PhypII** (Supplementary Materials Figures S5–S9) features were superimposed with the derived steric and electrostatic *PLS-coefficients* and jointly interpreted. The models’ robustness was monitored through leave-one-out (LOO) and leave-some-out (LSO) cross-validations (CV) (Figure 4 and Supplementary Material Tables S3–S6) [80,92], whereas any lack of chance correlation was confirmed by employing Y-scrambling (Y-S) [80,92].

The **D_1_**/**RED*_PLS-coefficients_*** (Figure 5 and Supplementary Materials Figures S1–S9) emphasized that the ERα binder should possess the mixed hydrogen bond donating (HBD)/hydrogen bond accepting (HBA) functional group (like the frequently present aromatic hydroxyl group, i.e., **1st PhOH**, as in **1ERR**, Table 1, Figure 5A, [13]), to form hydrogen bonds (HBs) with H3 Glu353 and H6 Arg394, at the same time not too voluminous, according to the **YELLOW*_PLS-coefficients_*** maps.

The **D_2_** feature/**GREEN*_PLS-coefficients_***/**RED*_PLS-coefficients_*** (Figure 5 and Supplementary Materials Figures S1–S4) indicated that another, *p*-positioned HBD/HBA functional group (i.e., **2nd PhOH**, as found in **1ERR**, Table 1, Figure 5A, [13]) is required to form HB with H11 His524 [17,18,19,20].

**Figure 6:**
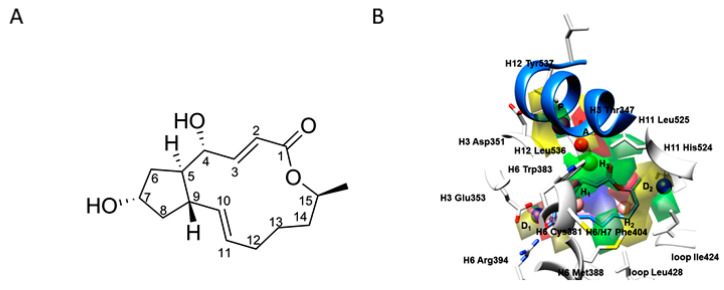
The **NCI89671** (viz., BFA) structure and nomenclature (**A**); the SB/LB virtually screened conformations of **NCI89671**, SB conformation blue, LB conformation pink (**B**).

The **H_1_** (**R_1_**) feature/**GREEN*_PLS-coefficients_***/**YELLOW*_PLS-coefficients_*** (Figure 5 and Supplementary Materials Figures S1–S9) suggested that the **1st PhOH** and **2nd PhOH** should be interconnected with five-membered (**1ERR**, Table 1, Figure 6A and Supplementary Materials Figure S5A [13]) or six-membered heterocyclic aliphatic bridge (**1XP1**, Table 1, Figure 6C and Supplementary Materials Figure S5C [64]), to interact with H6 Met388 H6-to-H7 loop residues Phe404, Ile424, and Leu428, maintaining the voluminosity toward distinct residues as low as possible [66]; according to the **BLUE***_PLS-coefficients_*, the bridge may be improved by means of an HBD, to face H3 Glu353 or H3 Thr347 (see **1XP1**, Table 1, Figure 5C [64]).

The **H_2_** (**R_2_**) feature/**GREEN*_PLS-coefficients_*/YELLOW*_PLS-coefficients_*** (Figure 5 and Supplementary Materials Figures S1–S9) indicated that the chemical linker between the **1st PhOH** and the **2nd PhOH** should not be further degraded (for instance toward the ethyl group of **3ERD** [69], Table 1, Figure 5B, Supplementary Materials Figure S5A), to avoid ERα partial agonism and pure ERβ antagonism and that the bulkiness of **2nd Ph-OH** toward H6 Met388 and H6-to-H7 loop residues Phe404, Ile424, and Leu428 is sufficient as is.

The **H_3_** (**R_3_**) feature/**GREEN***_PLS-coefficients_*/**RED***_PLS-coefficients_* (Figure 5 and Supplementary Materials Figures S1–S9) indicated that SERMs and SERDs, differently from partial agonists and ERβ selective binders (**3ERD** [69], Table 1, Figure 5B, Supplementary Materials Figure S5A), should possess a central phenyl ring, hereinafter labeled as **Ph** (see **1ERR**, Table 1, Figure 5A) [13] and **1XP1**, Table 1, Figure 5C [64]) to sterically interact with the H3 Thr347 side chain methyl group and alleviate the H3 Thr347-H11 Leu525-H12 Leu536 hydrophobic network formation (stabilized by the auxiliary H3 Ala350-**Ph**-H11 Leu525 network) [13]. The bulkiness of **Ph** could be increased toward H6 Trp383 (note the **GREEN*_PLS-coefficients_***), whereas the *o*-hydrophobic/HBA substituents of **Ph** could activate Thr347′s side-chain hydroxyl group (see **GREEN*_PLS-coefficients_*/RED*_PLS-coefficients_***).

The **A** feature and **RED*_PLS-coefficients_*/YELLOW*_PLS-coefficients_*** (Figure 5 and Supplementary Materials Figures S1–S9) emphasized the electrostatic interactions of an ethanolamine’s oxygen atom (hereinafter labeled as **Oxy**), an extension of **Ph** (Table 1 and Table 2) with the H3 Thr347′s side-chain -OH group.

The **P** feature**/BLUE*_PLS-coefficients_***/**GREEN*_PLS-coefficients_*/YELLOW***_PLS-coefficients_* (Figure 5 and Supplementary Materials Figures S1–S9, see Supplementary Materials) discriminated SERMs from SERDs. Hence, SERMs (Table 1 and Table 2) should form an HB with H3 Asp351 by means of an HBD, such as the positively charged nitrogen within heterocyclic and aliphatic scaffolds of low(er) voluminosity (see **1ERR**, **1SJ0**, **1YIN**, **2R6W**, and **1UOM**, Figure 5A, Supplementary Materials Figures S1B,F, and S2B, respectively) [2,13,61,73], **1XP1** (Figure 5C) [64], **1XP6** (Supplementary Materials Figure S1A) [64], **2R6Y**, **1XP9**, **1YIM**, and **1XPC** (Supplementary Materials Figures S1C–E, and S2A) [26,28,36], **2IOK** and **2IOG** (Figure 5E Figure 6C) [68], and **1XQC** (Supplementary Materials Figure S3B) [65]), to stabilize the H12 in the open conformation [6,10,13,64], at the same time keeping the steric pressure toward H12 at minimum or reducing it. On the other hand, SERDs (Table 1 and Table 2) should form an HB with H3 Asp351 via the HBA/HDB portion (like carboxylic acid within the phenyl acrylic acid (as in **1R5K**, Supplementary Materials Figure S2D [59] and **5AK2**, Figure 6B [74]), to provoke the proteasomal degradation of ERα [17,18,19,20].

### 2.3. Predictive Ability Assessment of the 3-D PhypI/3-D QSAR Model Ensemble

To validate the **3-D PhypI/3-D QSAR model**’s predictive ability, the TS_CRY_ (Table 3 and Table 5) (Refs. [69,75,76,77,78,79]) and TS_MOD1_-TS_MOD3_ (Supplementary Materials Tables S10–S15) [94,95,96,97,98,99,100,101,102] were used. For the sake of the reader, only the predictions of TS_CRY_ are herein discussed. Using a consensus score strategy [80,91,92], the bioactive conformations of modeled compounds [103] within the TS_MOD1_-TS_MOD3_ (see the section Predictive ability assessment of the 3-D PhypI/3-D QSAR model ensemble), were obtained using SB [104,105,106,107] or LB alignment [80,91,92], as described in the Supplementary Material (see Supplementary Materials Alignment assessment rules, Structure-based alignment assessments, and Ligand-based alignment assessments sections, as well as Tables S7–S9 and Figures S10–S19).

TS_CRY_’s experimentally available binding conformation’s p*K*_i_ values (herein improperly assumed as pIC_50_s) were thereafter predicted with an average absolute error of predictions (AAEPs) of 0.66 and 2.35 for the model optimized with LOO and LSO CVs, respectively (Table 5) and associated predictive *q*^2^ (*q*^2^_pred_) values were 0.51 and 0.39, respectively. Interestingly and as expected, the SB re-aligned molecules were predicted with lower errors (*q*^2^_pred_/AAEP values of 0.46/1.27 and 0.46/1.27 for LOO and LSO derived models) than those LB re-aligned (*q*^2^_pred_/AAEP values of 0.29/1.37 and 0.31/1.40 for LOO and LSO derived models). These values indicated the good predictive ability [108,109,110] of the **3-D PhypI/3-D QSAR model** ensemble and support the goodness of the realignment methodology.

### 2.4. Virtual Screening, Anticancer Potency, and Binding Mode Analysis of Brefeldin A as a Hit for Hit-to-Lead Optimization towards Innovative SERMs

The **3-D PhypI/3-D QSAR model** coupled with SB/LB alignment rules was used to perform a virtual screening (SB/LB VS) [87,90] on 4411 compounds taken from the National Cancer Institute (NCI). The top-ranked 18 virtual hits (See Supplementary Materials: Virtual screening, Table S16, and Supplementary Materials Figures S20–S22), with either SB or LB predicted pIC_50_ values, were experimentally validated as either ERα binders or antiproliferative agents against MCF-7, MDA-MB-231, and MRC-5 cell lines (Supplementary Materials Table S17). Compound coded as **NCI89671**, a naturally occurring compound Brefeldin A (**BFA**, Figure 6A) [111], as the most potency predicted, did exert promising activity against ERα (IC_50_ of 8.34 μM) and the MCF-7 cell line (IC_50_ of 9.01 μM), and selectivity against the MDA-MB-231 cell line (selectivity index (SI) of 11.10), although less potent than the references **E_2_** [13], 4-hydroxytamoxifen (**4-OHT**) [32], and raloxifene (**Ral**) [13] (Supplementary Materials Table S17). Previously assessed anti-BC properties of **BFA** and its derivatives were associated with the apoptosis and the compounds’ ability to disrupt the *cis*-Golgi apparatus [112,113]. Interestingly, C4- and C7-esters of **BFA** exerted nM antiproliferative activity against MCF-7 cell lines [114], C4-succinyl, glutaryl **BFA** analogs, and C7-long lipids derivatives showed μM to nM potencies against MCF-7 cell lines [115], whereas the sulfide- and sulfoxide-conjugated **BFA** analogs were active against MDA-MC-435 cell lines as μM and sub-micromolar ranges [116].

**BFA** binding mode analysis showed an interaction profile as a putative partial agonist, likely inducing the H12 in a closed conformation (Figure 6B) [13]. Thus, the **BFA**’s cyclopentane ring and the C7-OH group formed H-bonds with H3 Glu353 and H6 Arg394 (*d*_HB_ = 2.855 and 2.990 Å, respectively). Moreover, the C4-OH portion established the electrostatic interactions with H3 Glu353. On the other hand, the close contact of the C15-CH_3_ with H11 His524 was accounted as unfavorable by the **3-D PhypI/3-D QSAR model** ensemble, suggesting the insertion of either HBA or HBD functionality. Consequently, the C1-to-C4 carbon atoms were interfaced to H12, whereas the C9-to-C15 skeleton was engaged in van der Waals interactions with H6 Met388 and H6-to-H7 loop residues Ile423 and Leu428. Finally, the C1 carbonyl group was observed away from any interesting interactions, not satisfying any **3-D PhypI/3-D QSAR model** features, indicating it as a possible substitution point into an HBA group. Hence, the **3-D PhypI/3-D QSAR model** ensemble indicated that the modification of the C15-CH_3_ into C15-OH could endow **BFA**’s horizontal flip toward Glu353/Arg394, at the same time positioning the cyclopentane ring’s C7-OH group toward the His524 (an alignment comparable to the **E_2′_**s D ring and C17-OH group experimental conformation [13]). In such a scenario the C1 carbonyl group would face Glu353 and the C-4 OH group would become a further anchor point for the implementation of a **Ph**-containing scaffold.

### 2.5. Rules for the Rational Design of Novel Brefeldin A Derivatives as SERMs

The **BFA** structural optimization toward novel ERα SERMs (Table 6) was thereafter performed by applying the guidelines from the **3-D PhypI/3-D QSAR model** ensemble, applicable only for the rational design of SERMs. The *partial agonist-to-SERM* conversion was undertaken by applying the following strategies:

1. The **BFA**’s C15-CH_3_ group was converted to C15-OH as a mixed HBA/HBD functional group to increase the compounds’ capacity for establishing hydrogen bonds with either H3 Glu353 and H6 Arg394 (or H11 His524) and hopefully the solubility (data not shown).
2. The **BFA**’s C4-OH was substituted with 3-acetyl-4-hydroxybenzoic acid to provide interactions with H6 Trp383 and H3 Thr347, as well as to stabilize the H3 Thr347-Leu525-H12 Leu536 hydrophobic network, and consequent H12 dislocation. Choosing 3-acetyl-4-hydroxybenzoic acid as a **BFA**’s C4-OH substituent was an experimentally-guided decision since the tentative attempts to synthetically incorporate (see further text) the 1-(1,4-dihydroxynaphthalen-2-yl)ethenone as a fragment, perhaps more suitable to target H6 Trp383 by means of steric interactions, failed.
3. The 3-acetyl-4-hydroxybenzoic acid’s *p*-OH was further substituted with either ethanolamine-based moieties, bearing primary and secondary amines, or various *N*-, *O*-, and *N*, *O*-heterocycles or 2-hydroxyethanesulfonic acid functions, capable of inducing the AF-2 function dislocation. The primary amine, secondary amine, and 2-hydroxyethanesulfonic acid were chosen as the AF-2 function invaders to reduce the steric pressure on H12, at the same time with the eligibility to establish HBs with H3 Asp351. On the other hand, as the **3-D PhypI/3-D QSAR** model ensemble was not explicit on whether to keep the steric pressure on H12 or to reduce it completely, the various *N*-, *O*-, and *N*, *O*-heterocycles were chosen as bioisosteres of heterocycles found within the ERα binders (Table 1 and Table 2) in a way that their HBD functional groups could primarily engage H3 Asp351, thus influencing, alongside the steric pressure, the H12′s induced fitting, whereas the existing HBA functional groups could produce additional favorable interactions with the surrounding residues.
4. The 12 designed compounds, belonging to the **3-D PhypI/3-D QSAR**-based series, viz., **3DPQ**, were then subjected to the SB/LB alignment (Supplementary Materials Figures S23 and S24) and the pIC_50_ prediction procedures against ERα (Table 6). This way, the designed compounds composed the ultimate prediction set [109,110] for the **3-D PhypI/3-D QSAR model** ensemble, in which the SB and LB models’ associated *q*^2^_pred_ and AAEP values were 0.858/0.045 and 0.732/0.1, respectively. Indeed, even eight compounds, namely **3DPQ-12**, **3DPQ-3**, **3DPQ-9**, **3DPQ-4**, **3DPQ-2**, **3DPQ-1**, **3DPQ-7**, and **3DPQ-11** were predicted as more potent than **1ERR** [13] (the most potent TR compound; see further text).

### 2.6. Synthesis of Brefeldin A Derivatives 3DPQ-1 to 3DPQ-12

Designed compounds **3DPQ-1** to **3DPQ-12** were synthesized in high yields and purities (Scheme 1). The synthetic protocols and associated ^1^H NMR, ^13^C NMR, ^15^N NMR, and ^17^O NMR spectral data, as well as the HPLC spectra confirming compounds’ purity of 95% and higher, are reported in Supplementary Materials (Synthetic protocols for the preparation of compounds **3DPQ-1** to **3DPQ-12**, Synthesized Compounds spectral data interpretation, Supplementary Materials Figures S26–S190).

Thus, the building of a **BFA**-like core started with the previously reported two-step conversion of 1,5-pentanediol towards the aldehyde **R1** (87% yield), containing the aldehyde functional group at position C1-OH and *tert*-butyldimethylsilyl chloride (TBS-Cl)-protected C5-OH portion [117]. Following this, **R1** was converted into **R2** (88% yield), an intermediate containing the single-methylated hydroxyl group within the geminal diol sub-structure as a forebear of what would be the **BFA**’s C15 methyl group: the conversion occurred upon the asymmetric addition of dimethylzinc using the (-)-1,8-diazabicyclo [5.4.0]undec-7-ene ((-)-DBNE) as chiral ligand at a reaction temperature of 0 °C; the **R2** was purified by silica gel flash chromatography (Et_2_O:EtOAc = 10:1 *v*/*v* as eluent) [118]. Afterward, **R2** was TBS-deprotected with 1*N* HCl to give **R3** (95% of yield), further converted to the 1-phenyl-1*H*-tetrazole-5-thiol derivative **R4** (70% of yield) using a Mitsunobu reaction that assumed: (*i*) the protection of the free hydroxyl group of the geminal diol sub-structure by TBS-Cl; (*ii*) the addition of 1-phenyl-1*H*-tetrazole-5-thiol in dry THF to the deprotected C5-OH of **R3**, as well as the inclusion of TBS-Cl in imidazole and 4-(dimethylamino)pyridine (DMAP) onto the free hydroxyl group of the geminal diol (the product was purified using silica gel flash chromatography (Et_2_O:EtOA = 40:1 *v*/*v* as eluent)) [114]. Following this, the Mo(VI)-catalyzed oxidation of **R4** produced tetrazolyl sulfone **R5** (75% of yield), refined by silica gel flash chromatography (Et_2_O:EtOAc = 2:1 *v*/*v* as eluent) [119] and further subjected to Julia−Kocienski olefination with **R6** (prepared as described elsewhere in 67% yield [119,120] and containing the MEM-protected hydroxyl groups), using potassium hexamethyldisilazane (KHMDS) in toluene as a base, to give *E*-olefin **R7** in 73% yield [121,122]. Subsequently, the selective deprotection of TBS-OH within the geminal diol with 1*N* HCl gave **R8** (89% yield), purified by silica gel flash chromatography (Et_2_O:EtOAc = 3:1 *v*/*v* as eluent) [114].

**R8** was then subjected to the Yamaguchi lactonization, furnishing **BFA-D1** (88% yield) [123]. In particular: (*i*) the **R8′**s methyl ester (the functionality originating from **R6**) was hydrolyzed with 1*N* LiOH; (*ii*) the Yamaguchi reagent (2,4,6-trichlorobenzoyl chloride) was added to the carboxylic acid, in the environment of NEt_3_, resulting in the formation of an intermediate anhydride (not isolated); (*iii*) the reaction of the anhydride with the hydroxyl group of the geminal diol in the presence of DMAP generated the lactone **BFA-D1**, where the **BFA**’s C15 methyl group was successfully transformed into the methoxy one. Subsequently, the MEM-protecting groups were removed from **BFA-D1** with HBr [124], and the obtained **BFA-D2** was purified by silica gel flash chromatography (Et_2_O:EtOAc = 1:1 *v*/*v* as eluent) and recrystallized from MeOH in 75% yield [114]. Subsequently, the **BFA-D2′**s C7-OH group was protected with *tert*-butyldimethylsilyl trifluoromethanesulfonate (TBSOTf) in 2,6-lutidine to form **BFA-D3** in moderate yield (25%, purified by silica gel flash chromatography (*n*-hexane:EtOAc = 8:2 *v*/*v* as eluent) [114]. The **BFA-D3′**s C4-OH was afterward subjected to esterification with 3-acetyl-4-hydroxybenzoic acid, in the presence of 1-ethyl-3-(3-dimethylaminopropyl) carbodiimide hydrochloride (EDAC∙HCl) and DMAP, and in the prolonged reaction time (24 h), to give **BFA-D4,** purified by column chromatography on silica gel (*n*-hexane:EtOAc = 1:1 *v*/*v* as eluent) [125].

The **BFA-D4′**s *p*-OH moiety then was equimolar alkylated with either commercially available pro-reagents **Pro-R1, Pro-R2**, **Pro-R4**, **Pro-R5**, **Pro-R10**, and **Pro-R11** (i.e., 2-chloro-*N*-methylethanamine, 2-chloro-*N*,*N*-dimethylethanamine, 2-chloroethanesulfonic acid, 1-(2-chloroethyl)pyrrolidine-2,5-dione, 1-(2-chloroethyl)piperidine, and 1-(2-chloroethyl)pyrrolidine, respectively), or with prepared **Pro-R3**, **Pro-R6**, **Pro-R7**, **Pro-R8**, **Pro-R9,** and **Pro-R12** (i.e., 1-(2-chloroethyl)-1,2,5,6-tetrahydropyridine-3-carboxylic acid, 6-(2-chloroethyl)-6,7-dihydro-5*H*-pyrrolo [3,4-b]pyridine, 2-(2-chloroethyl)-1,2-dihydroisoquinoline, 1-(2-chloroethyl)decahydro-1,8-naphthyridine, 1-(2-chloroethyl)-6-methylene-1,2,5,6-tetrahydropyridine-3-carboxylic acid, and 1-(2-chloroethyl)-3-hydroxypiperidin-2-one, respectively), in the environment of potassium carbonate under reflux, to give **Pro-3DPQ-1** to **Pro-3DPQ-12** [126]. Subsequent deprotection of compounds’ C7-OH, with *tert*-butylammonium fluoride in THF [114], and C15-OH groups, using the complete demethylation of C15-OH using the boron tribromide (2 equiv. per methoxy function) in dry dichloromethane at 0 °C [127], finally gave the designed compounds **3DPQ-1** to **3DPQ-12**.

### 2.7. Synthesized Compounds Antagonistic Potency and Relative Binding Affinities against ERα and ERβ

The **3DPQ-1** to **3DPQ-12** were then investigated for their potency to antagonize either ERα (Table 7 and Supplementary Materials Figures S191 and S192) or ERβ (Table 7 and Supplementary Materials Figures S193 and S194) [128,129]. The experimentally determined IC_50_ values for **3DPQ-1** to **3DPQ-12** against ERα (Table 7) were highly correlated to those predicted by the **3-D PhypI/3-D QSAR model** ensemble (Table 6). Compounds **3DPQ-12**, **3DPQ-3**, **3DPQ-9**, **3DPQ-4**, **3DPQ-2**, **3DPQ-1**, **3DPQ-7**, and **3DPQ-11** were more potent ERα antagonists than both **Ral** and **4-OHT**, exerting potency in the pM range. All the compounds were potent ERα binders and poor ERβ binders (see logRBA values Table 7).

**Figure 7:**
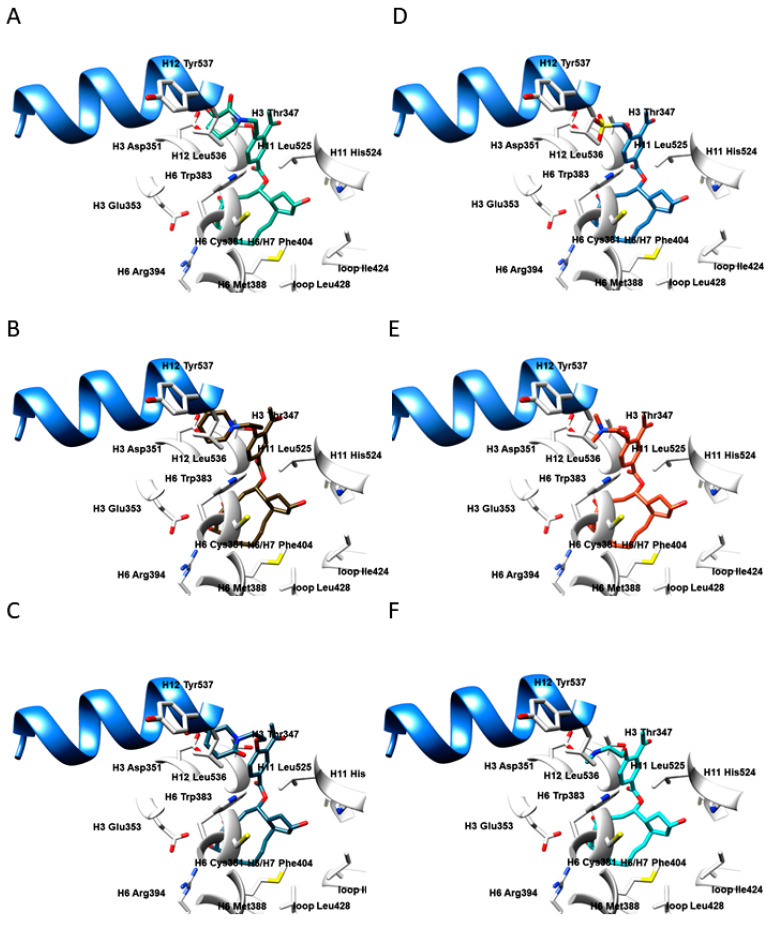
The bioactive conformations of **3DPQ-12** (**A**); **3DPQ-3** (**B**); **3DPQ-9** (**C**); **3DPQ-4** (**D**); **3DPQ-2** (**E**); **3DPQ-1** (**F**) within the ERα active site. Amino acid residues are depicted in white, H12 helix is presented in cornflower blue ribbon.

Compared to **BFA**, in all the synthesized compounds, the C15-CH_3_ to C15-OH conversion seemed to participate in an ERα’s LDB main core horizontal flipping (Figure 7 and Supplementary Materials Figure S195). Thus, the C15-OH faced the H3 Glu353 and H6 Arg394 to establish two further HBs (see Supplementary Materials Table S18 for details). Consequently, the C1 carbonyl portion produced weak electrostatic interactions with H6 Trp383′s indole ring nitrogen. The C8-C15 carbon skeleton was observed to be sterically attracted by H6 Met388 and H6-to-H7 loop residues Ile423 and Leu428. The inverse alignment of the main core influenced the spatial positioning of the cyclopentane ring’s C7-OH, as well, which produced HBs with H11 His524 (see Supplementary Materials Table S18 for details). The remaining C1-C4 carbon backbone participated in steric hindrance with H6 Trp383. Furthermore, the esterification of the C4-OH portion with 3-acetyl-4-hydroxybenzoic acid influenced the H3 Thr347-H11 Leu525-H12 Leu536 hydrophobic network [13,69] formation: the ester oxygen electrostatically targeted the H11 His524 side chain, while the *p*-carbonyl group made H-bonds with H3 Thr347′s side-chain hydroxyl (see Supplementary Materials Table S18 for details); the incorporated *o*-Ac-Ph moiety formed eclipsed (i.e., edge to edge) van der Walls interactions with the H3 Thr347′s side chain methyl group using its own methyl group, as well as the additional HBs with H3 Thr347′s side chain hydroxyl group (see Supplementary Materials Table S18 for details) by the acetyl group carbonyl portion. The unsubstituted 3-acetyl-4-hydroxybenzoic ac carbons faced the H12 Leu536 in a T-shaped fashion. Furthermore, the *p*-O-CH_2_-CH_2_- bridge bore the **3DPQ-1′**s to **3DPQ-12′**s functionalities that forced the H12 drifting, at the same time establishing the electrostatic attraction with H3 Thr347′s hydroxyl group via the oxygen atom and the steric interactions between the methylene carbons and the Leu536 isobutyl group.

The activity and SERM pharmacology [13] of **3DPQ-12** (Table 7, Supplementary Materials Figure S191A, Figure 7A, potency 1.85-fold higher than **Ral**) could be also ascribed to the 3-hydroxypiperidin-2-one portion: positioned beneath the Asp351-Leu536 plane, its hydroxyl group established an HB with Asp351 (the *d*_HB_ = 3.112 Å), stabilizing ERα with H12 in the open conformation; the carbonyl group electrostatically interfered with the Thr347′s side chain hydroxyl group, whereas the carbon skeleton was in the proximity of Leu536 isobutyl group. A slightly less potent SERM, for just 0.04 nM, was the **3DPQ-3** (Table 7, Supplementary Materials Figure S191B, Figure 7B, potency 1.68-fold higher than **Ral**), whose 1,2,5,6-tetrahydropyridine-3-carboxylic acid scaffold formed an HB with Asp351 (the *d*_HB_ = 3.222 Å) via the carboxyl group, whereas the carbon skeleton behaved similarly as in **3DPQ-12**. Furthermore, the potency of **3DPQ-9** (Table 7, Supplementary Materials Figure S191C, Figure 7C, 1.64-fold stronger binder than **Ral**), decreased by 0.01 nM related to **3DPQ-3** with the introduction of the carbonyl portion at position C6 of 1,2,5,6-tetrahydropyridine-3-carboxylic acid, which electrostatically attracted the Trp383′s indole ring nitrogen, having a consequence in C3-COOH group dispositioning and a weaker HB with H3 Asp351 (the *d*_HB_ = 3.314 Å).

The substitution of the bulky heterocycle, bearing an HBD, with a sulphonyl group, like as in the SERM **3DPQ-4** (Table 7, Supplementary Materials Figure S191D, Figure 7D, 1.57-fold stronger binder than **Ral**), lowered the potency by only a low nM fraction relative to **3DPQ-12**, despite the sulphonyl group forming a weak HB with Asp351 (the *d*_HB_ = 3.347 Å). However, the sulphonyl group replacement with either *N*,*N*-dimethyl, or *N*-methyl ones, within **3DPQ-2** (Supplementary Materials Figure S191E, Table 7, Figure 7E) and **3DPQ-1** (Supplementary Materials Figure S191F, Table 7, Figure 7F) as SERMs (HB lengths with Asp351 of 3.122 and 3.083 Å, respectively), led to a potency decrease (compounds were still 1.37-fold to 1.30-fold more potent than **Ral**, respectively).

SERMs like **3DPQ-7** (Table 7, Supplementary Materials Figures S192A and S195A, 1.16-fold more potent than **Ral**) and **3DPQ-11** (Table 7, Supplementary Materials Figures S192B and S195B, 1.06-fold more potent than **Ral**) formed via 1,2-dihydroisoquinoline and 1-(2-chloroethyl)pyrrolidine scaffolds hydrophobic interactions with the Leu536 isobutyl group and weaker HBs with Asp351 (*d*_HB_s = 3.922 and 3.136 Å, respectively, thus lowering the potency) via the nitrogen atom. Furthermore, the piperidine (**3DPQ-10**, Table 7, Supplementary Materials Figures S192C and S195C, 1.45-fold more potent than **4-OHT**), pyrrolidine-2,5-dione (**3DPQ-5**, Table 7, Supplementary Materials Figures S192D and 195D, 1.38-fold more potent than **4-OHT**), decahydro-1,8-naphthyridine (**3DPQ-8**, Table 7, Supplementary Materials Figures S192E and 195E, 1.38-fold more potent than **4-OHT**), and 6,7-dihydro-5H-pyrrolo [3,4-b]pyridine (**3DPQ-6**, Table 7, Supplementary Materials Figures S192F and 195F, 1.33-fold more potent than **4-OHT**) reduced the potency due to their inability to form HBs with Asp351.

### 2.8. Synthesized Compounds Antiproliferative Activity against ERα(+)- and ERα(-)-Dependent Breast Cancer Cell Lines as Well as against ERα(+)-Dependent Endometrial Cancer Cell Lines

Synthesized compounds were evaluated as antiproliferative agents against MCF-7 (Table 8, Supplementary Materials Figures S196 and S197), and MDA-MB-231 (Table 8, Supplementary Materials Figures S198 and S199) cells lines [130], respectively, as well as for the ability to induce ERα downregulation in MCF-7 cells (Table 8) [15,21,131,132] and to antagonize the progesterone receptor (PR) (Table 8) [126].

Compounds-proposed bioactive conformations anticipated a SERM-like profile, which was experimentally confirmed as they induced no ERα degradation, at the same time exerting no antagonism against PR (Table 8) [125]. Therefore, the further focus was on the antiproliferative activity, where even eight derivatives showed antiproliferation against MCF-7 better or comparable to **Ral** (Table 8). **3DPQ-12** (Table 8, Supplementary Materials Figure S196A) was the most potent MCF-7 cell growth inhibitor with an IC_50_ value equal to 560 pM and a selectivity index (SI) relative to MDA-MB-231 cell lines of 147.93. Similar antiproliferation profiles were also exerted by **3DPQ-3** (Table 8, Supplementary Materials Figure S196B, potency 1.11-fold lower than **3DPQ-12** but 1.43-fold higher than **Ral,** SI equal to 131.66) and **3DPQ-9** (Table 8, Supplementary Materials Figure S196C, potency 1.09-fold lower than **3DPQ-12** but 1.46-fold more potent than **Ral,** SI equal to 142.02).

Comparably with the latter two, **3DPQ-4** (Table 8, Supplementary Materials Figure S196D) had an antiproliferative potency 1.14-fold lower than **3DPQ-12** and 1.39-fold higher than **Ral**, with an SI of 105.17. The **3DPQ-2** (Table 8, Supplementary Materials Figure S196E, 1.21-fold more potent than **Ral**), **3DPQ-1** (Table 8, Supplementary Materials Figure S196F, 1.17-fold more potent than **Ral**), **3DPQ-7** (Table 8, Supplementary Materials Figure S197A, 1.14-fold more potent than **Ral**), and **3DPQ-11** (Table 8, Supplementary Materials Figure S197B, 1.10-fold more potent than **Ral**) showed antiproliferative potency ranging from 730 and 810 pM, but with lower SIs.

As SERMs profile is often associated with the stimulation of endometrial cell proliferation and an increase in the incidence of endometrial cancer (EC) [130], the herein compounds were therefore evaluated against Ishikawa endometrial adenocarcinoma cells (Table 8, Supplementary Material Figures S200 and S201). At this stage of evaluation, the herein SERMs significantly inhibited Ishikawa cell lines growth. However, future experimental elaboration, currently beyond the authors’ experimental facilities, is required to confirm compounds’ promising profiles in terms of no EC induction [130].

### 2.9. The Impact of Targeted ERα Antagonists on the MCF-7 Cells Signaling

The exerted antiproliferation against MCF-7 cell lines was further inspected for the inner mechanisms of action. **BFA** is known for inducing the endoplasmic reticulum stress within the MCF-7 cell lines, as well as for increasing the expression of p53, a major BC suppressor [132]. Nonetheless, ERα binds to p53, resulting in the inhibition of transcriptional regulation by p53, p53-mediated cell cycle arrest, and apoptosis [133], raising the question of whether the ERα antagonists herein described could have also inhibited MCF-7 cells’ growth by decreasing the ERα recruitment and by stimulating the p53′s transactivation function. To investigate this hypothesis, the conventional and sequential site-specific ChIP assays were employed to reveal the mechanisms by which the **3DPQ-1** to **3DPQ-12-**antagonized ERα influenced the p53-mediated transcriptional activation of the p21 gene (a prototypic p53-target gene) [133]. Experimentally, all the compounds except **3DPQ-5**, **3DPQ-6**, and **3DPQ-8** have been re-administered in 0.1 and 1 nM to MCF-7 cells (i.e., two concentrations encircling the IC_50_ values against MCF-7 cells, Table 8); for the marked compounds, the concentrations were 1 and 10 nM.

**Figure 8:**
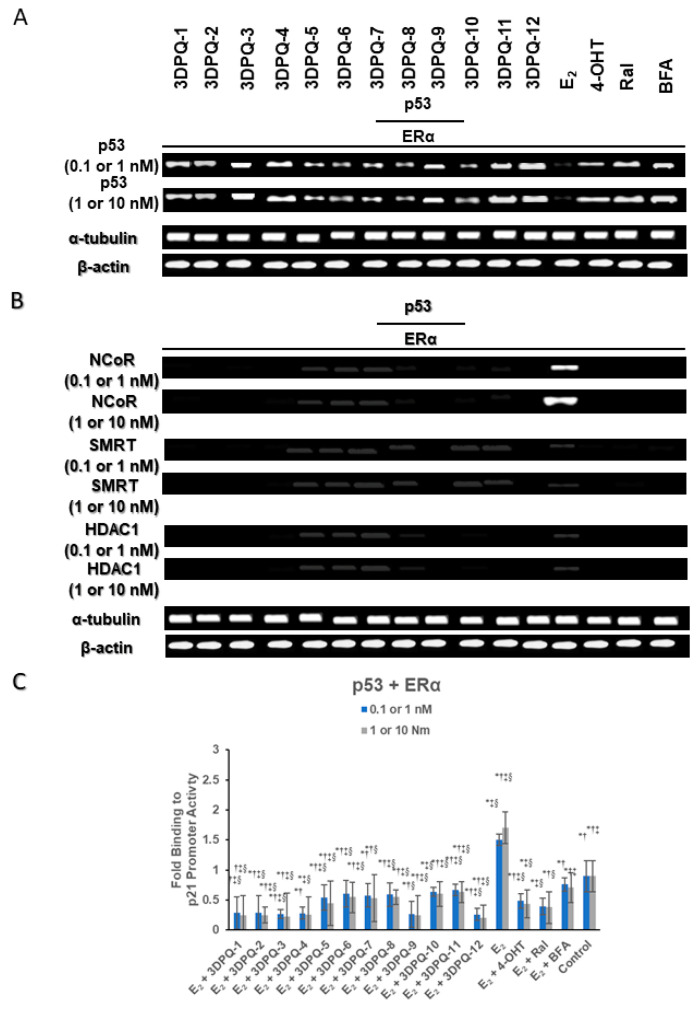
ERα recruits transcriptional corepressors to repress p53-mediated transcriptional activation. (**A**) ChIP and sequential ChIP assays were performed on MCF-7 cells saturated with **3DPQ-1** to **3DPQ-12** in concentrations of 0.1 and 1 nM (for **3DPQ-5**, **3DPQ-6**, and **3DPQ-8** the concentrations were 1 and 10 nM) with primers specific to the p53-binding site of the p21 promoter. The primary ChIP was performed with anti-p53 antibody, and the immunoprecipitate was subjected to a second ChIP with anti-ERα antibody; (**B**) The immunoprecipitate from the ERα ChIP was then subjected to the third ChIP with antibodies against NCoR, SMRT, and HDAC1 antibodies; (**C**) qChIP was performed to analyze the ERα–p53 interaction on the p21 promoter in MCF-7 cells saturated with **3DPQ-1** to **3DPQ-12**. Cells were grown in media with dextran-coated charcoal-treated FBS for 4 d and treated with E_2_ (1 and 10 nM) with or without **3DPQ-1** to **3DPQ-12** for 3 h. * *p* < 0.05 when compared with control group; ^†^ *p* < 0.05 when compared with **E_2_**; ^‡^ *p* < 0.05 when compared with **4-OTH**; ^§^ *p* < 0.05 when compared with **Ral**.

Upon the addition of primers specific to the p53-binding site of the p21 promoter, the chromatin was immunoprecipitated with the anti-p53 antibody and re-immunoprecipitated with the anti-ERα antibody, enabling the conclusion that the p53 expression occurred after the ERα has been antagonized by compounds (Figure 8A). The final round of re-immunoprecipitation was performed with NCoR and SMRT corepressors, guided by the premise that **3DPQ-1** to **3DPQ-12** as antiestrogens could promote their binding to ERα, followed by the recruitment of HDACs and leading to transcriptional repression [134,135]. Nonetheless, as NCoR, SMRT, and HDAC1 had been not recruited to the p21 promoter when ERα was knocked down (Figure 8B), ERα-**3DPQ-1** to ERα-**3DPQ-12** complexes, conversely to ERα, stimulated the p53-mediated transcriptional activation without recruiting the distinct corepressors.

Furthermore, the quantitative ChIP (qChIP) analysis measured the strength of **3DPQ-1** to **3DPQ-12** to affect the ERα’s ability to bind to p53. Contrary to **E_2_**, **3DPQ-1** to **3DPQ-12** disrupted the receptor’s interaction with the p21 promoter (Figure 8A) and stimulated the p53 transcriptional activity. The highest rate of p53 promoter activity was induced upon the **3DPQ-12**, **3DPQ-3**, and **3DPQ-9** administration, 0.65-fold and 0.55-fold, 0.68-fold and 0.61-fold, as well as 0.68-fold and 0.66-fold higher than the one provoked by **Ral** in lower and higher concentrations, respectively (Figure 8B). The **3DPQ-4** was similarly potent to **3DPQ-9**, exerting 0.70-fold and 0.68-fold higher potency than **Ral**, respectively, whereas **3DPQ-2** and **3DPQ-1** exerted the matching potency, 0.733-fold and 0.66-fold higher than **Ral** (Figure 8A). Conclusively, as ERα and SERMs, **3DPQ-1** to **3DPQ-12** have indeed decreased ERα recruitment and stimulated the p53 (p21) pathway, as another way of preventing the growth of MCF-7 cells.

### 2.10. Effects of Synthesized Compounds on Cytotoxicity and Cell Cycle Distribution of MCF-7 Cell Lines

The above data encouraged further analysis of the cell cycle of MCF-7 cells treated by **3DPQ-1** to **3DPQ-12** (Table 9, Supplementary Material Figures S202–S213) [130], administered at the same concentrations used for the cell signaling assay. Thus, compounds induced the MCF-7 cells’ arrest in the G_0_/G_1_ phase, i.e., the phase in between the non-division, post mitosis (viz., G_0_), and DNA replication (viz., G_1_). The G_0_/G_1_ phase arrest was accompanied by a decrease in the S phase, suggesting that compounds stopped the MCF-7 proliferation before the DNA replication induced by the transcriptional machinery. The results agreed with previous findings that SERMs block MCF-7 cell cycle progression in G_0_/G_1_ [136]. It is worth emphasizing that for all the compounds, applied in both concentrations, the contribution of the G_0_/G_1_ phase to the MCF-7 cells’ arrest was higher than 70%.

The distribution of **3DPQ-12** (Table 9, Supplementary Material Figures S202A,E), and **3DPQ-4** (Table 9, Supplementary Material Figures S205A,E) within the cell cycle mostly affected the cells’ proliferation, reaching 77 to 80% of the contribution of the G_0_/G_1_ phase upon administering either 0.1 or 1 nM of the compound, respectively. On the other hand, **3DPQ-3** (Table 9, Supplementary Material Figures S203A,E), **3DPQ-9** (Table 9, Supplementary Material Figures S204A,E), **3DPQ-2** (Table 9, Supplementary Material Figures S206A,E), and **3DPQ-1** (Table 9, Supplementary Material Figures S207A,E) blocked the MCF-7 cycle in the initial phase between 71 and 76%. The cell cycle arrest in the G_0_/G_1_ phase may be a key mechanism by which targeted antiproliferative agents inhibit MCF-7 cell proliferation.

### 2.11. Prediction of ADMETox Properties for the Compounds

Before the in vivo examination, ADMETox properties [137] were predicted in silico to assess the safety of the compounds as drug-like compounds (Table 10).

Hence, considering the Lipinski rule of five (RO5) (molecular weight < 500 Da, n-octanol–water partition coefficient < 5, hydrogen bond donor ≤ 5, hydrogen bond acceptor ≤ 10, polar surface area between 40–130) [138], of all the examined compounds only **3DPQ-2** and **3DPQ-7** could be considered drug-like, as they violated one or fewer of the RO5 criteria.

However, as more compounds that do not obey all the RO5 rules still reach the market as commercial drugs [139], tentative attempts have been made to revise RO5 [140,141,142,143]. Therefore, the optimal physicochemical and pharmacokinetic properties are considered preferable to RO5 [137]. In that sense, the binding to human serum albumin (QPlogKhsa), the IC_50_ values for the blockage of HERG K^+^ channels (QPlogHERG), the Caco-2 cell (i.e., the gut–blood barrier) permeability (QPPCaco), as well as the MDCK cell (i.e., the blood–brain barrier mimic) permeability (QPPMDCK), and the brain/blood partition coefficient (QPlogBB) were predicted by means of the Schrödinger’s QikProp module [144]. Indeed, the **3DPQ-12**, **3DPQ-3**, **3DPQ-9**, **3DPQ-4**, **3DPQ-2**, and **3DPQ-1**, as the most promising compounds elaborated so-far, showed optimal QPlogKhsa, QPlogHERG, and QPPCaco, accompanied by satisfying values for QPPMDC and QPlogBB. The toxicological assessments of organ and genomics performed by virtue of the admetSAR 2.0 webserver (http://lmmd.ecust.edu.cn/admetsar2, accessed on 1 March 2022) [145], viz., carcinogenicity, eye corrosion, eye irritation, Ames mutagenesis, micronuclear, hepatotoxicity androgen receptor binding, and PPAR-γ gamma, proved the safety of the leads.

### 2.12. In Vivo Anticancer Screening

Due to the observed data, **3DPQ-12**, **3DPQ-3**, **3DPQ-9**, **3DPQ-4**, **3DPQ-2**, and **3DPQ-1** were subjected to the in vivo screening to determine their impact on the mammary tumorigenesis (Table 11) [146].

Experimentally, the adult female Wistar rats were pretreated intraperitoneally (*i.p.*) with methyl nitrosourea (**MNU**) with a dose of 50 mg/kg of each rat’s body weight (bwt) to induce the BC, after which the compounds herein described were administered per os in two doses, 5 and 50 mg/kg of bwt [81]. The compounds were evaluated employing latency period (i.e., the time passed between the rats being exposed to **MNU** and the BC detection), tumor burden (i.e., the number of cancer cells), and tumor volume.

Hence, **3DPQ-12**, **3DPQ-3**, and **3DPQ-9** induced the longest latency period, 12 to 15 weeks depending on the concentration applied, followed by its low burden and volume, overpowering the efficiency of **Ral** (Table 11). The **3DPQ-4** induced a latency period between 9 and 12 weeks. The remaining leads, **3DPQ-2** and **3DPQ-1**, were slightly less efficient tumor suppressants, with tumor latency between 7 to 12 weeks and more emphasized tumor burdens and volumes, but were still more potent than **Ral**. Of course, the safety of the compound during administration was confirmed with liver enzyme catalytic activities and redox status [147,148,149,150,151,152,153,154,155] (Supplementary Materials Tables S19 and S20), where no significant harm was detected.

Being orally administered to rats, **3DPQ-12**, **3DPQ-3**, **3DPQ-9**, **3DPQ-4**, **3DPQ-2**, and **3DPQ-1** exerted good pharmacokinetic profiles (Table 11) [74,156], with high affinity for plasma protein binding [157], relatively low in vivo clearances [158], and no damage to hepatocytes, which correlated with results concerning the low liver enzyme catalytic activities redox status (Supplementary Materials Tables S19 and S20). Overall good oral exposure was observed in all the leads alongside favorable bioavailability.

**Figure 9:**
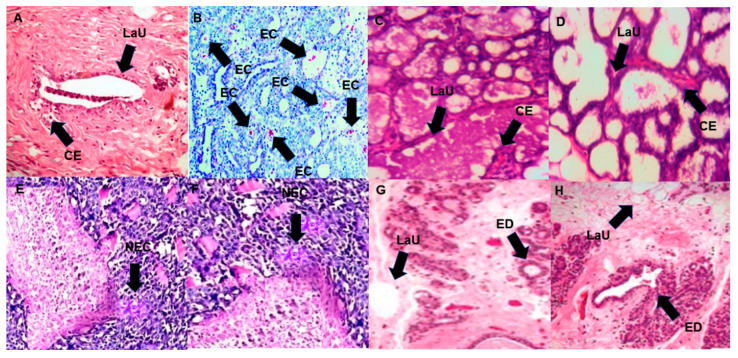
Photomicrograph of breast section of a normal control rat showing lobuloalveolar unit (LaU) and cuboidal epithelial cells (CE) (**A**); photomicrograph of breast section treated with **MNU** showing mammary gland carcinoma alongside with massive proliferation of neoplastic epithelial cells (EC) (**B**); photomicrograph of breast section treated with **3DPQ-12** in a concentration of 5 mg/kg of bwt showing lobuloalveolar unit (LaU) and cuboidal epithelial cells (CE) (**C**); photomicrograph of breast section treated with **3DPQ-12** in concentration of 50 mg/kg of bwt showing lobuloalveolar unit (LaU) and cuboidal epithelial cells (CE) (**D**); photomicrograph of breast section treated with **4-OHT** in a concentration of 5 mg/kg of bwt showing necrosis (NEC) (**E**); photomicrograph of breast section treated with **4-OHT** in concentration of 50 mg/kg of bwt showing necrosis (NEC) (**F**); photomicrograph of breast section treated with **Ral** in a concentration of 5 mg/kg of bwt showing differentiated extralobular ducts (ED) (**G**); photomicrograph of breast section treated with **Ral** in a concentration of 50 mg/kg of bwt showing differentiated extralobular ducts (ED) (**H**), shown in ×200 magnification and stained with hematoxylin and eosin.

The impact of selected leads on BC tissue was registered after their administration to experimental animals with **MNU**-induced BC (Figure 9 and Supplementary Material Figures S211–S218) [159]. Thus, compared to the normal pathological finding of animals treated with saline, reflected in photomicrographs revealing lobuloalveolar unit (LaU) and cuboidal epithelial cells (CE) (Figure 9A), **MNU** provoked ductal mammary gland carcinoma and massive proliferation of neoplastic epithelial cells (EC) (Figure 9B), changes found within the terminal ductal-lobular unit, that formed discrete clusters with duct-like morphology. In contrast to this, the administered leads were harmless in both concentrations, neutralizing the **MNU**-induced changes, judging by the lobuloalveolar units and cuboidal epithelial cells found (Figure 9C,D, and Supplementary Material Figures S214–S218). These compounds were safer than **4-OHT**, which caused severe necrosis (NEC) (Figure 9E,F), and **Ral**, which caused extralobular ducts (ED) (Figure 9G,H).

Finally, the compounds were assayed for the maximum tolerated dose (MTD) or maximum feasible dose (MFD, in the absence of MTD) and weight loss (WL) studies (Table 11). Compounds and controls were daily re-administered per os in five doses, 5, 50, 100, 500, and 1000 mg/kg bwt [160] for 5 days. On the 5th day, the body weights were measured, and the postmortem evaluations were performed by means of a gross examination of all the animals at the terminal necropsy, as well as the histopathological examination of lungs, spleen, liver, kidneys, heart, and colon (Supplementary Materials Figures S219–S224, respectively). Hence, except for **MNU**, with an MTT of 100 mg/kg bwt, no mortality was observed in the treatment groups for 5 days even at the highest dose (Table 11). The orally administered compounds **3DPQ-12**, **3DPQ-3**, **3DPQ-9**, **3DPQ-4**, **3DPQ-2**, and **3DPQ-1** did not produce significant changes in body weight. Moreover, no obvious pathologic changes were observed based on histology or necropsy compared to placebo-treated controls. Therefore, given that the Food and Drug Administration (FDA) recommends 1000 mg/kg bwt as the high limit dose for acute, subchronic, and chronic toxicity studies in rodents and non-rodents [160], MTDs were not explicitly determined, and the 1000 mg/kg bwt could be considered as MFD (https://www.fda.gov/drugs/guidance-compliance-regulatory-information/guidances-drugs, accessed on 1 March 2022) for **3DPQ-12**, **3DPQ-3**, **3DPQ-9**, **3DPQ-4**, **3DPQ-2**, and **3DPQ-1** [160]. All the compounds were proven safe for further pre-clinical and clinical trials at a concentration of 50 mg/kg bwt.

## 3. Materials and Methods

### 3.1. ERα LBD-Partial Agonists/Antagonists Complexes Structures Preparation

The 39 complexes of ERα partial agonists and antagonists, co-crystallized with either wild-type (WT) or mutated (MUT) receptors, retrieved from PDB (TR, Table 1: 18 WT ERα binders with the activities reported as pIC_50_s; Table 2: 8 MUT ERα binders with the activities reported as pIC_50_s) and test set (TS_CRY_, Table 3: 13 WT and MUT ERα binders with the activities reported as p*K*_i_s) were prepared [93,161] using the validated procedures described elsewhere [80,92] (see the Supplementary Materials: Crystal structures compilation and preparation and Supporting Information Table S1 for detailed information).

### 3.2. 3-D Pharmacophore Hypotheses and 3-D QSAR Models Generation

A set of 3-D pharmacophore hypotheses and atom-based 3-D QSAR models were generated using the PHASE software [88] as implemented in Schrödinger’s suite [89], using the default setup (see the Supplementary Materials: Pharmacophore modeling and 3-D QSAR modeling for detailed information). For the statistically best hypotheses/models (endowed with the highest *q*^2^ values), robustness was confirmed by means of leave-one-out (LOO) and leave-some-out (LSO) cross-validations (CV) [80,92] while lack of chance correlation was checked by a Y-scrambling procedure [80,92]. Models were graphically interpreted by means of UCSF Chimera [93].

### 3.3. SB Alignment Assessment

All the scoring functions of the Glide software [104,105,106], as implemented in Schrödinger’s Suite [89], were evaluated to select the best one to perform an SB alignment assessment on TR compounds. The SB procedure was assessed through four methods, similar to those previously described in [80,92]: experimental conformation re-docking (ECRD), randomized conformation re-docking (RCRD), experimental conformation cross-docking (ECCD), and randomized conformation cross-docking (RCCD). The experimental protocols and Glide’s settings [105,106] are reported in the Supplementary Materials: Alignment assessment rules, Ligand’s experimental conformations randomizations, and Glide settings.

### 3.4. LB Alignment Assessment

To rule out the LB molecular alignment of TR compounds, all the available scoring functions of the flexible ligand alignment tool (FLA) [89], as implemented in Schrödinger’s Suite [89], were evaluated. The LB alignment procedure assessment was conducted at different levels of difficulty, similar to those previously described in [80,92]: experimental conformation re-alignment (ECRA), randomized conformation re-alignment (RCRA), experimental conformation cross-alignment (ECCA), and randomized conformation cross-alignment (RCCA). The experimental protocols and FLA setup [89] are reported in the sections Supplementary Materials: Alignment assessment rules and Flexible Ligand Alignment tool settings.

### 3.5. The SB/LB Alignment Accuracy

The alignment fitness was then quantified by evaluating both the RMSD and the subsequent docking accuracy (DA) and alignment accuracy (AA), as previously reported [80,92]. Both DA and AA were used to evaluate how the algorithms used could predict the ligand poses as closely as possible to the experimentally observed ones, by separating the correctly (RMSD ≤ 2 Å) and partially (2 Å ≤ RMSD ≤ 3 Å) docked/aligned poses for those mis-docked/mis-aligned (RMSD ≥ 3 Å). The rules for DA and AA calculation are reported in Supplementary Materials *Alignment assessment rules* section.

### 3.6. Generation of Modeled and Designed Compounds

Either TS_MOD1′_s, TS_MOD2′_s, and TS_MOD3′_s (Supplementary Materials Tables S10–S15) or the designed compounds (Table 8) were drawn through the Chemaxon’s msketch module [103] by means of the optimization of the molecular mechanics using the MMFF94 force field and the default settings, upon which the hydrogen atoms were assigned at pH 7.4. Upon structures’ generation, compounds were uploaded into previously described best-performing SB and LB protocols to obtain the bioactive conformations (see Supplementary Materials: Alignment assessment rules, Structure-based alignment assessments, and Ligand-based alignment assessments).

### 3.7. Test Sets and Designed Compounds Alignment

The TS_MOD1_, TS_MOD2_, and TS_MOD3_ (Supplementary Materials Tables S10–S15), as well as all the designed compounds (Table 6), were aligned applying either the best performing SB or LB protocols (see Supplementary Materials: Test sets alignment, Alignment assessment rules, Structure-based alignment assessments, and Ligand-based alignment assessments).

### 3.8. Virtual Screening

The virtual screening of NCI compound libraries (486 compounds from Natural Products Set 3 and 1574 and 2351 compounds from the Diversity Sets 2 and 3), taken from the NCI (NCI, https://www.cancer.gov/, accessed on 1 October 2015) was conducted following the guidelines as described elsewhere [90,91]. The compounds were retrieved in structure data file (sdf) format, split into individual files, imported in Chemaxon’s msketch module [103], and energy minimized by means of molecular mechanics’ optimization using the MMFF94 force field and the default settings, upon which the hydrogen atoms were assigned at pH 7.4. Upon the generation of the structures, compounds were uploaded into previously determined best-performing SB and LB protocols to perform cross-docking and cross-alignment and obtain the bioactive conformations against ERα (see Supplementary Materials: Virtual screening, Alignment assessment rules, Structure-based alignment assessments, and Ligand-based alignment assessments).

### 3.9. 3-D Pharmacophore Hypotheses and 3-D QSAR Models External Validation and Prediction Ability

The TS_CRY_ (Table 5), TS_MOD1_, TS_MOD2_, TS_MOD3_ (Supporting Information Tables S10–S15), virtually screened compounds (Supporting Information Tables S16–S17), and the designed compounds (Table 6) were imported into the best **3-D pharmacophore hypothesis/3-D QSAR model** ensemble (see 3-D pharmacophore and 3-D QSAR modeling and models’ interpretation) and predicted by means of the activity [80,92].

### 3.10. Synthesis of Compounds 3DPQ-1 to 3DPQ-12

All the experimental work regarding the conventional synthesis of designed compounds **3DPQ-1** to **3DPQ-12**, as well as regarding spectral data interpretation and purity, is described in detail as Supplementary Materials under the Experimental and Results and discussion sections, respectively.

### 3.11. ADMETox Predictions for Compounds 3DPQ-1 to 3DPQ-12

The ADMETox properties were predicted by means of Schrödinger’s QikProp module [144] and admetSAR 2.0 webserver (http://lmmd.ecust.edu.cn/admetsar2, accessed on 1 March 2022) [145], using the default setup.

### 3.12. Biochemical Evaluation

All the biochemical experimental work was performed following the guidelines already reported in the literature. These are detailed in Supporting Materials, under the Experimental sections: Synthesized Compounds Antagonistic Potency and Relative Binding Affinities to ERα and ERβ [128,129], Synthesized Compounds Antiproliferative Activity against ERα(+)- and ERα(-)-Dependent Breast Cancer Cell Lines [130], ERα Down-Regulation [138,139], ERα Functional Antagonism Cell Assay [15,74,131], The Impact of targeted ERα Antagonists on the MCF-7 Cells Signaling [132,133,134,135], Effects of Synthesized Compounds on Cytotoxicity and Cell Cycle Distribution in ERα(+)Dependent Breast Cancer Cell Lines [130], Determination of Lipophilicity [74,156], In vivo Anticancer Screening [146], Measurement of Serum Biochemical Markers [159], Determination of Antioxidant Markers in Liver Homogenate [159], Plasma Protein Binding Determination [139], Determination of the Intrinsic Clearance of Hepatocytes [158], Pharmacokinetics Studies In Rats [158], and Histopathological Studies [159].

## 4. Conclusions

The reported investigation summarizes the usage of rational drug design protocol by means of the SB and LB techniques to disclose new potent and selective antagonists against ERα as in vitro and in vivo anticancer agents, which emerged upon the lead optimization of the virtually screened compound Brefeldin A. The SB 3-D pharmacophore/QSAR models, coupled with molecular docking and ligand-based alignment, were revealed to be effective tools in the design of new Brefeldin A derivatives and were used for the very first time to describe their potency against ERα in physiological conditions, using the ERα antagonists and partial agonists co-crystallized within both wild-type or mutated receptors. Notably, the models emerged from a wide-ranging molecular diversity within the training set, consisting of a variety of antagonists and partial agonists associated with SERDs, SERMs, and naturally occurring sub-groups of compounds. The best **ADDHHHP.13** hypothesis (**3-DPhypI**), alongside the derived 3-D QSAR model, differentiated full antagonists from partial agonists and provided some guidelines for the selectivity toward ERα, describing all the important 3-D pharmacophoric properties desired for a powerful SERM to occupy the natural hormonal environment and to invoke in perspective the complete shut-down of estrogen-initiated basal transcriptional machinery. Moreover, the **ADDHHHP.13** hypothesis was used to virtually screen NCI datasets disclosing BFA as an interesting hit, which was structurally optimized by engineering twelve innovative SERMs, **3DPQ-1** to **3DPQ-12**, that were synthesized, and broadly biochemically evaluated as ERα antagonists, as prospective BC suppressants. From determining the antagonistic potential against ERα, to elaborating the antiproliferative activity in ERα(+) BC cell lines, including the impact on the inner mechanisms of cancer development and toxicity predicted *in silico*, all of the designed and synthesized hits exerted notable potency, where slight differences in the activity can be understood from the structure-based point of view. The in vivo administration to adult Wistar rats discriminated the lead compounds by means of their impact on mammary tumorigenesis. Hence, **3DPQ-12**, **3DPQ-3**, **3DPQ-9**, **3DPQ-4**, **3DPQ-2**, and **3DPQ-1** were indeed found to be as potent as Ral, the most potent compound listed in the TR, at any stage of evaluation. By exerting more-than-promising anticancer activity, a favorable preclinical profile, and notable safety, **3DPQ-12**, **3DPQ-3**, **3DPQ-9**, **3DPQ-4**, **3DPQ-2**, and **3DPQ-1** can be considered candidates for pre-clinical and clinical trials as the future of SERM-related BC clinical therapy. In a future study, a model for the ERβ antagonists will be also developed to design selective antagonists.

## Figures and Tables

**Scheme 1:**
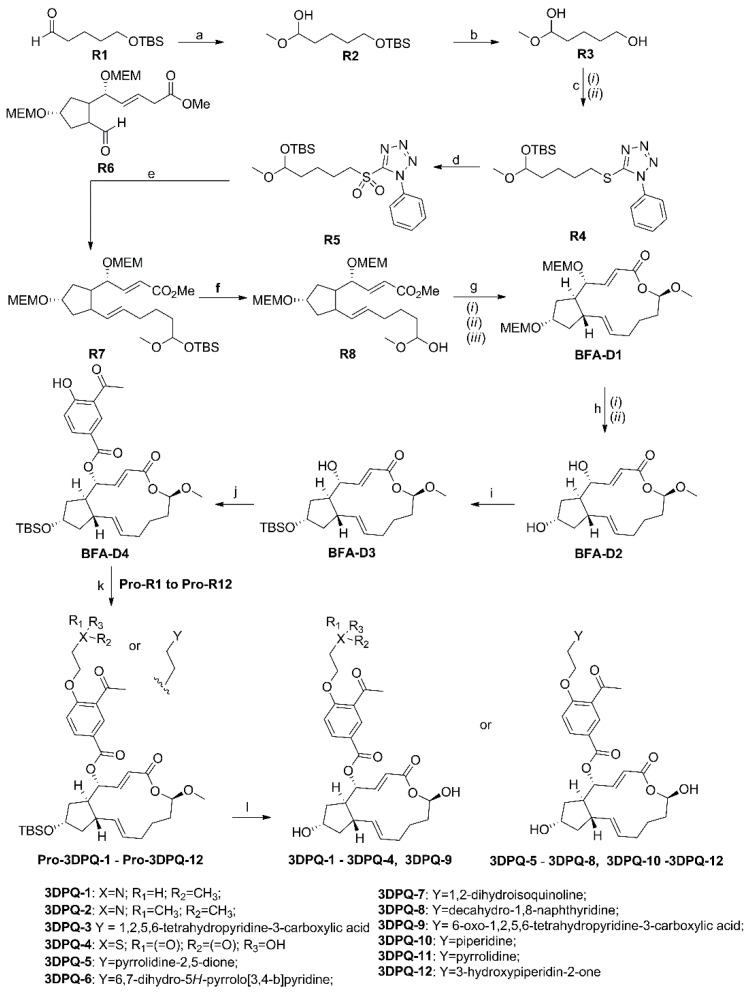
Synthesis of Brefeldin A derivatives **3DPQ-1** to **3DPQ-12**. Reagents and conditions: (a) Me_2_Zn, (−)-DBNE, toluene, 0 °C, 24 h, 87% ee; (b) HCl, THF, rt, 25 min; (c) (*i*) TBS-Cl, imidazole, DMAP, CH_2_Cl_2_, 0 °C, 3 h, (*ii*) PPh3, DEAD, 1-phenyl-1*H*-tetrazole-5-thiol, THF, 0 °C, 16h; (d) (NH_4_)_6_Mo_7_O_24_, H_2_O_2_, EtOH, rt, 16 h; (e) compound R6, KHDMS, 1,2-dimetoxyethane; -78 °C, 18h; (f) HCl, THF, rt, 1.5 h; (g) (*i*) LiOH, THF/H_2_O, rt, 2h, (*ii*) 2,4,6-trichlobenzoylchloride, NEt_3_, THF, rt, 1.5 h, (*iii*) DMAP, toluene, reflux, 5h; (h) (*i*) cc HBR, THF, rt, 1.5 h (*ii*) recrystallization; (i) TBSOTf, 2,6-lutidine, CH_2_Cl_2_, rt; (j) 3-acetyl-4-hydroxybenzoic acid, ECD, DMAP, CH_2_Cl_2_, reflux; (k) K_2_CO_3_, EtOH, reflux; (l) (*i*) TBAF, THF, rt, (*ii*) BBr_3_, CH_2_Cl_2_, 0 °C, 3h, reflux.

**Table 1:** PDB codes, ligand structures, and pharmacological profile of wild-type (WT) estrogen receptor α complexed with antagonists and partial agonists, for the 3-D Pharmacophore hypotheses generation compounds were classified into “actives” (PDB codes marked with a star) and “inactives” (PDB codes marked with a double star) using a threshold pIC_50_ value of 7.30.

PDB	Ligand Structure	pIC_50_	Ref.	PDB	Ligand Structure	pIC_50_	Ref.
1ERE * PA ^a^ H12: CC ^b^	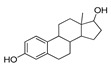	9.24	[13]	1XP9 * SERM H12: OC	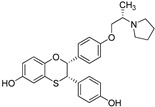	8.80	[64]
1ERR * SERM ^c^ H12: OC ^d^	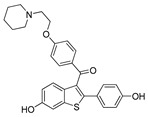	9.52	[13]	1XPC * SERM H12: OC	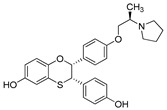	8.70	[64]
1GWQ ** PA H12: CC	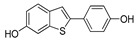	5.85	[60]	1XQC ** SERM H12: OC	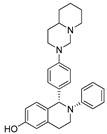	7.20	[65]
1R5K * SERD ^e^ H12: OC	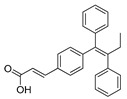	7.40	[59]	1YIM * SERM H12: OC	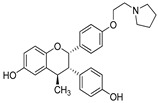	8.80	[66]
1SJ0 * SERM H12: OC	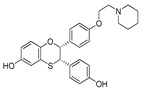	9.09	[61]	1YIN * SERM H12: OC	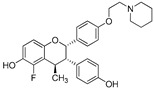	8.80	[66]
1X7E ** PA H12: CC	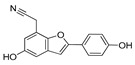	5.90	[62]	2BJ4 * SERM H12: OC	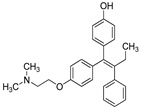	8.60	[67]
1X7R * PA H12: CC	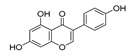	8.01	[63]	2IOG * SERM H12: OC	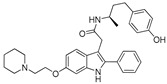	8.09	[68]
1XP1 * SERM H12: OC	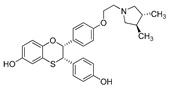	9.30	[64]	2IOK * SERM H12: OC	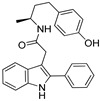	9.00	[68]
1XP6 * SERM H12: OC	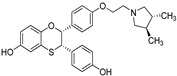	9.30	[64]	3ERD * PA H12: CC	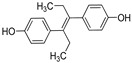	9.48	[69]

^a^ Partial agonist; ^b^ H12: closed conformation; ^c^ SERM—mixed agonist/antagonist; ^d^ H12: open conformation; ^e^ SERD—full antagonist.

**Table 2:** PDB codes, ligand structures, and pharmacological profile of mutated (MUT) estrogen receptor α complexed with antagonists and partial agonists; for the 3-D pharmacophore hypothesis generation, compounds were classified into “actives” (PDB codes marked with a star *) and “inactives” (PDB codes marked with a double star **) using a threshold pIC_50_ value of 7.30.

PDB	Ligand Structure	pIC_50_	Ref.	PDB	Ligand Structure	pIC_50_	Ref.
1L2I * PA ^a^ H12: CC ^b^	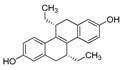	8.50	[2]	2R6W * SERM H12: OC	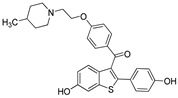	8.60	[73]
1UOM * SERM ^c^ H12: OC ^d^	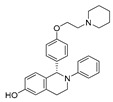	7.70	[70]	2R6Y * SERM H12: OC	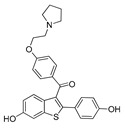	8.90	[73]
2B1Z ** PA H12: CC	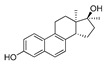	7.10	[71]	2QA8 * PA H12: CC	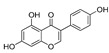	8.01	[72]
2QA6 ** PA H12: CC	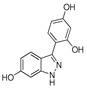	7.30	[72]	5AK2 * SERD ^e^ H12: OC	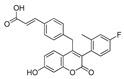	8.40	[74]

^a^ Partial agonist; ^b^ H12: closed conformation; ^c^ SERM—mixed agonist/antagonist; ^d^ H12: open conformation; ^e^ SERD—full antagonist.

**Table 3:** PDB codes, ligand structures, and pharmacological profile of WT and MUT estrogen receptor α complexed (the qualification indicated below the code) with antagonists and partial agonists used as test set (TS_CRY_).

PDB	Ligand Structure	p*K*_i_	Ref.	PDB	Ligand Structure	p*K*_i_	Ref.
**3ERT** **(WT)** PA ^a^ H12: CC ^b^	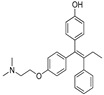	9.60	[69]	**4MG9** **(MUT)** PA H12: CC	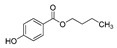	6.00	[77]
**3UU7** **(MUT)** PA H12: CC	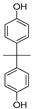	8.79	[75]	**4MGA** **(MUT)** PA H12: CC	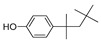	6.00	[77]
**3UUA** **(MUT)** PA H12: CC	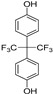	8.79	[75]	**4MGC** **(MUT)** PA H12: CC	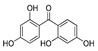	7.00	[77]
**3UUC** **(WT)** PA H12: CC	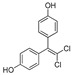	5.70	[75]	**4MGD** **(MUT)** PA H12: CC	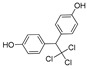	6.00	[77]
**4DMA** **(WT)** PA H12: CC	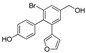	5.60	[76]	**4TUZ** **(MUT)** PA H12: CC	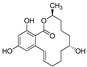	10.00	[78]
**4MG6** **(MUT)** PA H12: CC	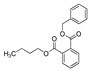	6.00	[77]	**4ZN9** **(MUT)** PA H12: CC	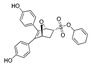	9.60	[79]
**4MG8** **(MUT)** PA H12: CC	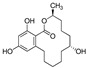	10.00	[77]				

^a^ Partial agonist; ^b^ H12: closed conformation.

**Table 4:** The alignment of best hypotheses pharmacophoric features (**A**: hydrogen-bond acceptor, D: hydrogen bond donor, **R**: ring feature, **H**: hydrophobic feature, **P**: positive ionizable feature) against **1ERR** (blue) and **1ERE** (pink). Scores of the different parameters (the upper part) and PLS statistical parameters (the lower part) of the top two hypotheses.

ADDRRRP.11						ADDHHHP.13				
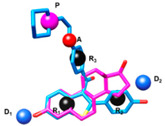						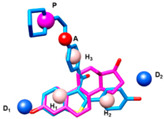				
**HID ^a^**	**S ^b^**	**S-I ^c^**	**P-H ^d^**	**S ^e^**	**V ^f^**	**VOL ^g^**	**SE ^h^**	**M ^i^**	**A ^j^**	**I ^k^**
**ADDRRRP.11**	3.741	0.967	6.429	0.81	0.991	0.426	2.678	17	9.52	1.751
**ADDHHHP.13**	3.743	0.963	6.432	0.83	0.993	0.431	2.674	17	9.30	1.755
	**PLSF ^l^**	***r* ^2 m^**	**SD ^n^**	***F* ^o^**	***P* ^p^**	**Stability ^q^**	***q* ^2^_LOO_^r^**	***q* ^2^_LSO_^s^**	***q* ^2^_YS LOO_^t^**	***q* ^2^_YS LSO_^u^**
**ADDRRRP.11**	5	0.949	0.264	61.3	4.38e^−15^	0.971	0.825	0.627	−0.234	−0.247
**ADDHHHP.13**	5	0.951	0.257	61.4	4.41e^−15^	0.977	0.826	0.659	−0.241	−0.258

^a^ Hypothesis identification; ^b^ Survival score; ^c^ Survival-inactives score; ^d^ Post-hoc—the result of rescoring; ^e^ Site score—an RMDS value for the site points superimposition in an alignment to the pharmacophore of the structures that contribute to this hypothesis; ^f^ Vector alignment score; ^g^ Volume of the contributing structures’ overlap when aligned on the pharmacophore; ^h^ Selectivity—the fraction of molecules matching the hypothesis regardless of their potency; ^i^ Matches—number of actives that match the hypothesis; ^j^ Activity—Activity of the reference ligand (pIC_50_); ^k^ Inactive—Survival score of inactives; ^l^ PLS factor, i.e., *N*/5, where *N* is the number of ligands present in the training set; ^m^ Conventional square-correlation coefficient. ^n^ Standard deviation of regression; ^o^ Ratio of the model variance to the observed activity variance; ^p^ Significance level of variance ratio; ^q^ Stability of the model predictions to changes in the training set composition; ^r^ Cross-validation correlation coefficient using the leave-one-out (LOO) method. ^s^ Cross-validation correlation coefficient using the leave-some-out (LSO) method with 5 random groups; ^t^ Average cross-validation correlation coefficient using the leave-one-out (LOO) method obtained after Y-scrambling process. ^u^ Average cross-validation correlation coefficient using the leave-some-out (LSO) method with 5 random groups obtained after the Y-scrambling process.

**Table 5:** Summary of the **3-D PhypI/3-D QSAR model** ensemble experimental/structure-based/ligand-based predictive ability for TS_CRY_.

Entry	p*K*_i_	EC Pred. p*K*_i_^a^		AAEP ^d^	SB Pred. p*K*_i_^a^		AAEP ^d^	LB Pred. p*K*_i_^a^		AAEP ^d^
		LOO ^b^	LSO ^c^		LOO ^b^	LSO ^c^		LOO ^b^	LSO ^c^	
**3ERT**	9.60	8.76	8.64	0.90	8.36	8.34	1.25	7.99	8.12	1.55
**3UU7**	8.79	8.14	6.91	1.27	8.09	7.22	1.14	7.85	7.14	1.30
**3UUA**	8.79	8.15	7.54	0.94	7.05	7.12	1.71	8.07	7.37	1.07
**3UUC**	5.70	4.36	4.39	1.33	4.45	4.06	1.45	5.67	6.77	0.55
**4DMA**	5.60	6.54	7.69	1.52	7.91	7.59	2.15	8.86	7.7	2.68
**4MG6**	6.00	4.76	4.77	1.24	4.17	3.03	2.40	4.16	4.82	1.51
**4MG8**	10.00	8.86	8.87	1.14	9.16	7.76	1.54	8.99	8.85	1.08
**4MG9**	6.00	7.12	6.52	0.82	6.19	4.10	1.05	4.51	5.96	0.77
**4MGA**	6.00	8.13	6.99	1.56	7.13	6.89	1.01	7.41	4.98	1.22
**4MGC**	7.00	8.66	6.7	0.98	6.36	6.54	0.55	7.58	5.85	0.87
**4MGD**	6.00	7.66	9.04	2.35	8.46	7.13	1.80	9.19	9.48	3.34
**4TUZ**	10.00	8.64	8.88	1.24	9.17	7.52	1.66	9.06	8.7	1.12
**4ZN9**	9.60	8.96	8.92	0.66	8.74	7.06	1.70	8.78	8.49	0.97

^a^ Predictions were obtained with a **3-D PhypI/3-D QSAR model** ensemble optimized with LOO and LSO cross-validations. ^b^ Leave-one-out cross-validation. ^c^ Leave-some-out cross-validation with 5-random-groups-out. ^d^ AAEP, the average absolute error of prediction of LOO and LSO cross-validations.

**Table 6:** Structures of designed hits and their predicted activities against ERα.

#	Ligand Structure	3DPhypI/3-D QSAR pred. pIC_50_		#	Ligand Structure	3DPhypI/3-D QSAR pred. pIC_50_^b^	
		SB ^a^	LB ^b^			SB ^a^	LB ^b^
**3DPQ-1**	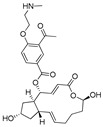	9.20	9.17	**3DPQ-7**	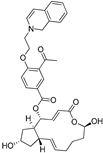	9.26	9.11
**3DPQ-2**	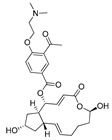	9.21	9.12	**3DPQ-8**	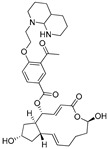	9.04	8.95
**3DPQ-3**	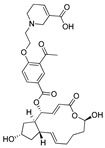	9.37	9.29	**3DPQ-9**	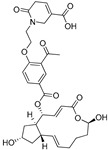	9.31	9.26
**3DPQ-4**	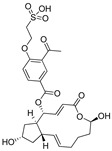	9.26	9.22	**3DPQ-10**	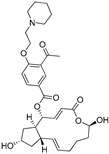	9.18	9.05
**3DPQ-5**	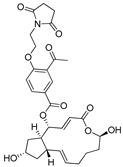	9.05	8.92	**3DPQ-11**	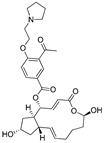	9.12	9.28
**3DPQ-6**	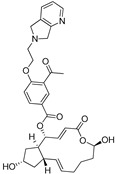	9.01	8.91	**3DPQ-12**	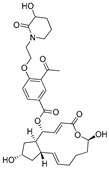	9.42	9.35

^a^ The designed compounds SB predicted activities by the **3DPhypI/3-D QSAR model**; ^b^ The designed compounds LB predicted activities by the **3DPhypI/3-D QSAR model**.

**Table 7:** Antagonistic potencies (IC_50_s) and the logarithm of the relative binding affinities (RBA) against ERα and ERβ of the newly synthesized compounds. Isoform affinity preferences and respective antagonist constants are also reported.

Comp.	ERα ^a^	ERβ ^b^	logRBA ^c^	logRBA ^d^	*K_a_* Erα ^e^	*K_a_* Erβ ^f^
	(IC_50_ nM)	(IC_50_ nM)	ERα	ERβ	(nM)	(nM)
**3DPQ-1**	0.57 ± 0.54 ^g,†,‡,§^	74.33 ± 0.46 ^†,‡,§^	2.19 ^‡,§^	0.08 ^†,‡,§^	0.13 ^†,‡^	41.76 ^†,‡,§^
**3DPQ-2**	0.54 ± 0.31 ^†,‡,§^	77.24 ± 0.42 ^†,‡,§^	2.22 ^†,‡,§^	0.06 ^†,‡,§^	0.12 ^†,‡^	43.39 ^†,‡,§^
**3DPQ-3**	0.44 ± 0.31 ^†,‡,§^	74.86 ± 0.14 ^†,‡,§^	2.31 ^†,‡,§^	0.08 ^†,‡,§^	0.10 ^†,‡^	42.06 ^†,‡,§^
**3DPQ-4**	0.47 ± 0.12 ^†,‡,§^	82.45 ± 0.54 ^†,‡,§^	2.28 ^†,‡,§^	0.03 ^†,‡,§^	0.11 ^†,‡^	46.32 ^†,‡,§^
**3DPQ-5**	0.81 ± 0.43 ^†,‡,§^	74.41 ± 0.46 ^†,‡,§^	2.04 ^‡^	0.08 ^†,‡,§^	0.18 ^†,‡^	41.80 ^†,‡,§^
**3DPQ-6**	0.84 ± 0.11 ^†,‡,§^	86.56 ± 0.33 ^†,‡,§^	2.03 ^‡^	0.01 ^†,‡,§^	0.19 ^‡^	48.63 ^†,‡,§^
**3DPQ-7**	0.64 ± 0.13 ^†,‡,§^	72.34 ± 0.17 ^†,‡,§^	2.14 ^†,‡^	0.09 ^†,‡,§^	0.14 ^†,‡^	40.64 ^†,‡,§^
**3DPQ-8**	0.81 ± 0.14 ^†,‡,§^	72.35 ± 0.78 ^†,‡,§^	2.04 ^‡^	0.09 ^†,‡,§^	0.18 ^†,‡^	40.65 ^†,‡,§^
**3DPQ-9**	0.45 ± 0.14 ^†,‡,§^	83.56 ± 0.46 ^†,‡,§^	2.30 ^†,‡,§^	0.03 ^†,‡,§^	0.10 ^†,‡^	46.94 ^†,‡,§^
**3DPQ-10**	0.77 ± 0.14 ^†,‡,§^	79.54 ± 0.76 ^†,‡,§^	2.06 ^‡^	0.05 ^†,‡,§^	0.17 ^†,‡^	44.69 ^†,‡,§^
**3DPQ-11**	0.70 ± 0.33 ^†,‡,§^	76.52 ± 0.48 ^†,‡,§^	2.10 ^‡^	0.07 ^†,‡,§^	0.16 ^†,‡^	42.99 ^†,‡,§^
**3DPQ-12**	0.40 ± 0.43 ^†,‡,§^	89.45 ± 0.31 ^†,‡,§^	2.35 ^†,‡,§^	0.00 ^†,‡,§^	0.09 ^†,‡,§^	50.25 ^†,‡,§^
**E_2_^h^**	0.88 ± 0.24 ^‡,§^	0.88 ± 0.32 ^‡,§^	2.00	2.00 ^‡,§^	0.20 ^‡,§^	0.49 ^‡,§^
**4-OHT. ^i^**	1.13 ± 0.24 ^†,§^	3.62 ± 0.43 ^†,§^	1.90 ^§^	1.39 ^†^	0.25 ^†,§^	2.03 ^†,§^
**Ral. ^j^**	0.73 ± 0.35 ^†,‡^	3.39 ± 0.16 ^†,‡^	2.09 ^‡^	1.42 ^†^	0.16 ^†,‡^	1.90 ^†,‡^
**Control ^k^**	NA ^l^	NA	NA	NA	NA	NA

^a^ Concentration that antagonizes the 50% of ERα signaling activity; ^b^ Concentration that antagonizes (inhibits) the 50% of ERβ signaling activity; ^c^ Logarithmic value of the percentage of relative binding affinity toward the ERα; ^d^ Logarithmic value of the percentage of relative binding affinity toward the ERβ (for both ^c^ values and ^d^ values relative binding affinity (RBA) values where calculated related to estradiol with an affinity of 100%, logRBA values higher than 0 refer to strong binders, logRBA values between −2 and 0 refer to moderate binders, logRBA values below −2 refer to weak binders); ^e^ Calculated antagonistic (i.e., inhibitory) constants against ERα; ^f^ Calculated antagonistic (i.e., inhibitory) constants against ERβ; ^g^ Results are presented as mean value ± standard deviation; ^h^ **17β-estradiol**; ^i^ **4-hydroxytamoxifen**; ^j^ **Raloxifene**; ^k^ No ligand (0.9% NaCl). ^l^ Not available. * *p* < 0.05 when compared with control group; ^†^ *p* < 0.05 when compared with **E_2_**; ^‡^ *p* < 0.05 when compared with **4-OTH**; ^§^ *p* < 0.05 when compared with **Ral**.

**Table 8:** Synthesized compound antiproliferative activity and selectivity index against hormone-dependent MCF-7, hormone-independent MDA-MB-231 breast cancer cell lines, normal MRC-5 human lung tissue fibroblasts cell lines, and Ishikawa endometrial adenocarcinoma cell lines, as well as the downregulation of ERα in MCF-7 and PR antagonism in MCF-7 cell lines.

Comp.	MCF-7 ^a^	MDA-MB-231 ^b^	SI ^c^	MRC-5 ^d^	MCF-7 DR ^e^	PR MCF-7 ^f^	Ishikawa ^g^
	(IC_50_ nM)	(IC_50_ nM)		(IC_50_ nM)	(IC_50_ nM)	(IC_50_ nM)	(IC_50_ nM)
**3DPQ-1**	0.76 ± 0.24 ^h,‡,§^	72.44 ± 0.32 ^‡,§^	95.31 ^‡,§^	>100	>100	>100	0.94 ± 0.36 ^g,‡,§^
**3DPQ-2**	0.73 ± 0.42 ^‡,§^	72.42 ± 0.47 ^‡,§^	99.20 ^‡,§^	>100	>100	>100	0.99 ± 0.35 ^‡^
**3DPQ-3**	0.61 ± 0.56 ^‡,§^	86.63 ± 0.68 ^‡,§^	142.02 ^‡,§^	>100	>100	>100	0.84 ± 0.74 ^‡,§^
**3DPQ-4**	0.64 ± 0.15 ^‡,§^	67.31 ± 0.34 ^‡,§^	105.17 ^‡,§^	>100	>100	>100	0.92 ± 0.43 ^‡,§^
**3DPQ-5**	1.02 ± 0.64 ^‡,§^	52.64 ± 0.69 ^‡,§^	51.61 ^‡,§^	>100	>100	>100	1.42 ± 0.32 ^‡,§^
**3DPQ-6**	1.14 ± 0.49 ^‡,§^	52.31 ± 0.46 ^‡,§^	45.89 ^‡,§^	>100	>100	>100	1.46 ± 0.43 ^‡,§^
**3DPQ-7**	0.78 ± 0.52 ^‡,§^	51.96 ± 0.68 ^‡,§^	66.61 ^‡,§^	>100	>100	>100	1.74 ± 0.43 ^‡,§^
**3DPQ-8**	1.06 ± 0.45 ^‡,§^	42.56 ± 0.35 ^‡,§^	40.15 ^‡,§^	>100	>100	>100	1.98 ± 0.32 ^‡,§^
**3DPQ-9**	0.62 ± 0.15 ^‡,§^	81.63 ± 0.42 ^‡,§^	131.66 ^‡,§^	>100	>100	>100	0.89 ± 0.24 ^‡,§^
**3DPQ-10**	0.97 ± 0.34 ^‡,§^	41.97 ± 0.32 ^‡,§^	42.27 ^‡,§^	>100	>100	>100	1.55 ± 0.42 ^‡,§^
**3DPQ-11**	0.81 ± 0.22 ^‡,§^	67.12 ± 0.54 ^‡,§^	82.86 ^‡,§^	>100	>100	>100	1.37 ± 0.47 ^‡,§^
**3DPQ-12**	0.56 ± 0.11 ^‡,§^	82.84 ± 0.61 ^‡,§^	147.93 ^‡,§^	>100	>100	>100	0.77 ± 0.43 ^‡,§^
**E_2_^i^**	N ^m^	NA	NA	NA	NA	NA	NA
**4-OHT. ^j^**	1.19 ± 0.57 ^§^	37.10 ± 0.45 ^§^	31.18 ^§^	>10	>100	>100	1.29 ± 0.43 ^§^
**Ral. ^k^**	0.90 ± 0.19 ^‡^	93.41 ± 0.48 ^‡^	103.97 ^‡^	>10	>100	>100	0.97 ± 0.35 ^‡^
**Control ^l^**	NA	NA	NA	NA	NA	NA	NA

^a^ Concentration that prevents the growth of 50% of MCF-7 cell lines; ^b^ Concentration that prevents the growth of 50% of MDA-MB-231 cell lines; ^c^ Selectivity index toward the cell line: [IC_50_(MDA-MB-231)]/[IC_50_(MCF-7)] for the antiproliferative effect of both designed compounds and reference compounds; ^d^ Concentration that prevents the growth of 50% of MRC-5 cell lines (human lung fibroblast cell lines, as a neutral control); ^e^ ERα downregulation measured in MCF-7 cell lines; ^f^ Progesteron receptor was measured as a biomarker for ERα antagonism in MCF-7 cell lines; ^g^ Concentration that prevents the growth of 50% of Ishikawa cell lines; ^h^ Results are presented as mean value ± standard deviation; ^i^ **17β-estradiol**; ^j^ **4-hydroxytamoxifen**; ^k^ **Raloxifene**; ^l^ 0.9%NaCl; ^m^ Not available. * *p* < 0.05 when compared with control group. ^‡^ *p* < 0.05 when compared with **4-OTH**; ^§^ *p* < 0.05 when compared with **Ral**.

**Table 9:** Effects of synthesized compounds on the MCF-7 cell cycle.

Comp.	Cell Cycle (%)					
Stage	G_0_/G_1_^a,b^		S ^c^		G_2_/M ^d,e^	
Conc. (nM)	0.1 (1) ^f^	1 (10)	0.1 (1) ^f^	1 (10)	0.1 (1) ^f^	1 (10)
**3DPQ-1**	72.62 ± 2.47 *^,†,‡,§^	75.08 ± 2.13 *^,†,‡,§^	9.98 ± 1.65 *^,†,‡,§^	10.69 ± 1.42 *^,†,‡,§^	17.40 ± 3.63 *^,†,‡,§^	14.24 ± 2.54 *^,†,‡,§^
**3DPQ-2**	73.64 ± 5.32 *^,†,‡,§^	76.10 ± 1.43 *^,†,‡,§^	11.88 ± 0.87 *^,†,‡,§^	12.59 ± 1.57 *^,†,‡,§^	14.48 ± 2.54 *^,†,‡,§^	11.32 ± 3.25 *^,†,‡,§^
**3DPQ-3**	72.99 ± 1.32 *^,†,‡,§^	75.45 ± 1.53 *^,†,‡,§^	8.98 ± 1.64 *^,†,‡,§^	9.69 ± 0.94 *^,†,‡,§^	18.03 ± 1.65 *^,†,‡,§^	14.87 ± 2.43 *^,†,‡,§^
**3DPQ-4**	77.78 ± 3.54 *^,†,‡,§^	80.24 ± 2.53 *^,†,‡,§^	7.20 ± 2.88 *^,†,‡,§^	7.91 ± 0.1.54 *^,†,‡,§^	15.02 ± 4.23 *^,†,‡,§^	11.86 ± 3.43 *^,†,‡,§^
**3DPQ-5**	71.78 ± 0.67 *^,†,‡,§^	74.24 ± 2.15 *^,†,‡,§^	9.21 ± 1.95 *^,†,‡,§^	9.92 ± 0.76 *^,†,‡,§^	19.01 ± 3.55 *^,†,‡,§^	15.85 ± 4.43 *^,†,‡,§^
**3DPQ-6**	70.52 ± 1.53 *^,†,‡,§^	71.98 ± 2.44 *^,†,‡,§^	13.27 ± 2.64 *^,†,‡,§^	13.98 ± 1.33 *^,†,‡,§^	16.21 ± 3.25 *^,†,‡,§^	14.05 ± 2.43 *^,†,‡,§^
**3DPQ-7**	73.25 ± 2.54 *^,†,‡,§^	75.71 ± 1.43 *^,†,‡,§^	14.06 ± 1.58 *^,†,‡,§^	14.77 ± 1.46 *^,†,‡,§^	12.69 ± 2.64 *^,†,‡,§^	9.53 ± 3.54 *^,†,‡,§^
**3DPQ-8**	72.39 ± 1.43 *^,†,‡,§^	74.85 ± 2.54 *^,†,‡,§^	12.50 ± 1.22 *^,†,‡,§^	13.21 ± 2.15 *^,†,‡,§^	15.11 ± 2.56 *^,†,‡,§^	11.95 ± 2.45 *^,†,‡,§^
**3DPQ-9**	71.47 ± 0.99 *^,†,‡,§^	75.93 ± 152 *^,†,‡,§^	12.97 ± 1.65 *^,†,‡,§^	13.68 ± 1.74 *^,†,‡,§^	15.56 ± 2.65 *^,†,‡,§^	10.40 ± 3.54 *^,†,‡,§^
**3DPQ-10**	71.96 ± 1.43 *^,†,‡,§^	74.42 ± 2.12 *^,†,‡,§^	11.96 ± 2.41 *^,†,‡,§^	12.67 ± 2.46 *^,†,‡,§^	16.08 ± 1.56 *^,†,‡,§^	12.92 ± 4.32 *^,†,‡,§^
**3DPQ-11**	72.53 ± 0.47 *^,†,‡,§^	74.99 ± 2.54 *^,†,‡,§^	13.31 ± 1.66 *^,†,‡,§^	14.02 ± 1.43 *^,†,‡,§^	14.16 ± 2.13 *^,†,‡,§^	11.00 ± 3.43 *^,†,‡,§^
**3DPQ-12**	77.83 ± 0.92 *^,†,‡,§^	80.29 ± 1.24 *^,†,‡,§^	16.96 ± 1.23 *^,†,‡,§^	17.67 ± 1.32 *^,†,‡,§^	5.21 ± 2.54 *^,†,‡,§^	2.05 ± 1.43 *^,†,‡,§^
**E_2_^g^**	17.34 ± 0.35 *^,‡,§^	25.34 ± 0.36 *^,‡,§^	28.15 ± 0.52 *^,‡,§^	29.52 ± 0.46 *^,‡,§^	54.51 ± 0.57 *^,‡,§^	45.14 ± 0.33 *^,‡,§^
**4-OTH. ^h^**	57.22 ± 0.37 *^,†,§^	63.26 ± 0.41 *^,†,§^	18.76 ± 0.41 *^,†,§^	21.14 ± 0.25 *^,†,§^	24.02 ± 0.53 *^,,†§^	15.60 ± 0.15 *^,†,§^
**Ral. ^i^**	59.14 ± 0.54 *^,†,‡^	66.52 ± 0.56 *^,†,‡^	15.83 ± 0.53 *^,†,‡^	16.37 ± 0.46 *^,†,‡^	25.03 ± 0.35 *^,†,‡^	17.11 ± 0.46 *^,†,‡^
**Control ^j^**	32.21 ± 0.45		34.97 ± 0.53		32.82 ± 0.35	

^a^ Cell resting states: G_0_—a cell has left the cycle and has stopped dividing; ^b^ Cell interphase (i.e., synthesis) state: G_1_—cells size increase (preparation for DNA synthesis); ^c^ Cell interphase (i.e., synthesis) state: S DNA replication; ^d^ Cell interphase (i.e., synthesis) state: G_2_—the gap between DNA synthesis and mitosis, in which the cell continues to grow; ^e^ Cell division states: M cell growth stops, division occurs; ^f^ The compounds concentration in nM administered to MCF-7 cells (all the compounds except **3DPQ-5**, **3DPQ-6**, and **3DPQ-8** have been re-administered in concentrations of 0.1 and 1 nM; for the marked compounds, the concentrations were 1 and 10 nM; Values: mean ± standard deviation. ^g^ **17β-estradiol**; ^h^ **4-hydroxytamoxifen**; ^i^ **raloxifene**; ^j^ 0.9% NaCl. * *p* < 0.05 when compared with control group; ^†^ *p* < 0.05 when compared with **E_2_**; ^‡^ *p* < 0.05 when compared with **4-OTH**; ^§^ *p* < 0.05 when compared with **Ral**.

**Table 10:** In silico physicochemical and pharmacokinetic properties of synthesized compounds.

**Comp.**	**mol_MWT ^a^**	**donorHB ^b^**	**acceptHB ^c^**	**QPlogPo/w ^d^**	**PSA ^e^**	**R05 ^f^**	**QPlogKshsa ^g^**	**QPlogHERG ^h^**	**QPPCaco ^i^**
**3DPQ-1**	501.243	3	9	2.11	133.084	2	−0.571	−5.759	26.396
**3DPQ-2**	515.254	2	9	2.49	124.532	1	−0.529	−5.242	27.138
**3DPQ-3**	597.263	3	11	2.29	131.324	3	−0.539	−5.354	31.352
**3DPQ-4**	552.175	3	11	1.43	136.387	3	−0.645	−5.367	25.872
**3DPQ-5**	569.234	2	11	1.45	160.686	3	−0.934	−4.029	26.464
**3DPQ-6**	590.261	2	10	3.01	154.432	2	0.005	−4.903	22.432
**3DPQ-7**	601.272	2	9	4.33	122.038	1	0.198	−5.836	34.075
**3DPQ-8**	610.336	3	10	3.51	133.649	2	0.191	−4.976	165.259
**3DPQ-9**	611.243	3	12	1.39	140.653	3	−0.562	−5.321	27.621
**3DPQ-10**	555.286	2	9	3.32	143.543	2	0.135	−4.324	132.594
**3DPQ-11**	541.276	2	9	2.81	143.653	2	0..162	−4.321	135.594
**3DPQ-12**	585.243	3	11	1.58	140.795	3	−0.900	−5.239	26.295
**E_2_^s^**	278.434	2	3	2.487	47.727	0	0.214	−1.994	1322.153
**4-OTH. ^t^**	407.679	1	5	4.201	36.102	0	0.669	−3.909	669.539
**Ral ^u^**	495.759	3	9	2.381	73.257	0	0.173	−3.648	130.539
	**QPPMDCK ^j^**	**QPlogBB ^k^**	**A ^l^**	**B ^m^**	**C ^n^**	**D ^o^**	**E ^p^**	**F ^q^**	**G ^r^**
**3DPQ-1**	26.435	−1.964	−	−	−	−	−	−	−
**3DPQ-2**	31.095	−1.892	−	−	−	−	−	−	−
**3DPQ-3**	34.542	−2.963	−	−	−	−	−	−	−
**3DPQ-4**	31.921	−2.735	−	−	−	−	−	−	−
**3DPQ-5**	32.351	−2.029	−	−	−	−	−	−	+
**3DPQ-6**	23.658	−2.432	−	−	−	−	+	−	+
**3DPQ-7**	14.190	−3.977	−	−	+	+	+	−	+
**3DPQ-8**	70.677	−3.237	−	−	−	−	+	−	+
**3DPQ-9**	36.284	−2.876	−	−	−	−	−	−	+
**3DPQ-10**	16.325	−3.321	−	−	+	−	−	−	−
**3DPQ-11**	18.362	−3.431	−	−	+	−	−	−	−
**3DPQ-12**	32.285	−2.682	−	−	−	−	−	−	+
**E_2_^s^**	669.023	−0.209	−	−	−	−	−	−	−
**4-OTH. ^t^**	354.743	−0.136	−	−	−	−	−	−	−
**Ral ^u^**	88.081	−0.582	−	−	−	−	−	−	−

^a^ Molecular weight (range:130.0–725.0); ^b^ Number of hydrogen bond donors (range: 0.0–6.0); ^c^ Number of hydrogen bond acceptors (range: 2.0–20.0); ^d^ Predicted *n*-octanol/water partition coefficient (Range: −2.0–6.5); ^e^ Van der Waals surface area (Range: 7.0 to 200.0); ^f^ Lipinski’s rule of five violations number (range: maximum is 4); ^g^ Prediction of human serum albumin binding (Range: −1.5 to +1.5); ^h^ Predicted IC_50_ for HERG K^+^ channels blockage (optimal: –5); ^i^ Predicted Caco-2 cell permeability in nm/sec (a gut–blood barrier model; <25 poor, >500 great; ^j^ Predicted MDCK cell permeability in nm/sec (a blood–brain barrier model; <25 poor, >500 great; ^k^ Predicted brain/blood partition coefficient (range: −3.0 to 1.2); ^l^ Carcinogenicity; ^m^ Eye corrosion; ^n^ Eye irritation; ^o^ Ames mutagenesis; ^p^ Hepatotoxicity; ^q^ PPAR gamma; ^r^ Androgen receptor binding; Active = (+), Inactive = (−); ^s^ **17β-estradiol**; ^t^ **4-hydroxytamoxifen**; ^u^ **raloxifene**.

**Table 11:** Effects of synthesized compounds on mammary tumorigenesis.

Comp.	Dose	log D_7.4_^a^	Tumor Latency	Tumor Burden	Tumor Volume	Rat PPB ^b^	Rat CL ^c^	BIO ^d^	MFD ^e^ (5 days)	WL after MFD ^f^ (day 1, mg) ^g^
	(mg/kg)		(week)	(week)	(mm^3^)	(%free)	in vivo		(mg/kg)	(day 5, mg) ^h^
**3DPQ-1**	5	1.94 ^‡,‖^	9 *	3.38 ± 0.31 ^i,^*^,†,‖^	1.09 ± 0.23 *^,†,‡,‖^	1.33 ^‡,‖^	60 ^‡,‖^	91	1000	310.34 ± 0.34 ^i^
	50		12 *^,†^	2.04 ± 0.35 *^,†,§,┴^	0.68 ± 0.35 *^,†,§,┴^	1.22 ^§,┴^	69 ^§,┴^	94		300.23 ± 0.62
**3DPQ-2**	5	1.99 ^‡,‖^	9 *	3.34 ± 0.57 *^,†,‖^	0.96 ± 0.41 *^,†,‡,‖^	1.15 ^‡,‖^	59 ^‡,‖^	92	1000	305.03 ± 0.66
	50		12 *^,†^	1.98 ± 0.45 *^,†,§,┴^	0.69 ± 0.23 *^,†,§,┴^	1.24 ^§,┴^	64 ^§,┴^	94		300.43 ± 0.65
**3DPQ-3**	5	2.07 ^‡,‖^	12 *^,†,‡^	2.18 ± 0.69 *^,†,‡,‖^	0.78 ± 0.43 *^,†,‡,‖^	1.34 ^‡,‖^	66 ^‡,‖^	90	1000	320,45 ± 0.62
	50		15 *^,†,§^	1.16 ± 0.64 *^,†,§,┴^	0.66 ± 0.21 *^,†,§,┴^	1.47 ^§,┴^	71 ^§,┴^	93		300.31 ± 0.52
**3DPQ-4**	5	1.88 ^‡,‖^	10 *^,†^	2.39 ± 0.56 *^,†,‡,‖^	0.98 ± 0.31 *^,†,‡,‖^	1.23 ^‡,‖^	64 ^‡,‖^	90	1000	320.73 ± 0.36
	50		14 *^,†^	1.33 ± 0.15 *^,†,§,┴^	0.41 ± 0.23 *^,†,§,┴^	1.51 ^§,┴^	76 ^§,┴^	93		305.56 ± 0.68
**3DPQ-9**	5	2.02 ^‡,‖^	12 *^,†,‡^	2.28 ± 0.47 *^,†,‡,‖^	0.77 ± 0.32 *^,†,‡,‖^	1.28 ^‡,‖^	62 ^‡,‖^	94 ^‡^	1000	315.54 ± 0.65
	50		15 *^,†,§^	1.14 ± 0.65 *^,†,§,┴^	0.40 ± 0.43 *^,†,§,┴^	1.31 ^§,┴^	78 ^§,┴^	97		310.33 ± 0.95
**3DPQ-12**	5	2.06 ^‡,‖^	12 *^,†,‡^	2.24 ± 0.54 *^,†,‡,‖^	0.67 ± 0.22 *^,†,‡,‖^	1.24 ^‡,‖^	63 ^‡,‖^	93 ^‡^	1000	305.06 ± 0.94
	50		15 *^,†,§^	0.94 ± 0.35 *^,†,§,┴^	0.34 ± 0.11 *^,†,§,┴^	1.31 ^§,┴^	71 ^§,┴^	96		299.56 ± 0.45
**4-OTH. ^j^**	5	3.64 ^‖^	7 *	3.36 ± 0.38 *^,†,‖^	1.88 ± 0.35 *^,†,‖^	1.85	35	88 ^‖^	1000	305.84 ± 0.59
	50		10 *^,†^	3.22 ± 0.21 *^,†,┴^	1.35 ± 0.63 *^,†,┴^	2.52 ^┴^	42	94		297.65 ± 0.39
**Ral. ^k^**	5	2.39 ^‡^	8 *	3.11 ± 0.47 *^,†,‡^	1.67 ± 0.31 *^,†,‡^	1.85	36	93 ^‡^	1000	310.54 ± 0.45
	50		13 *^,†^	2.91 ± 0.22 *^,†,§^	1.41 ± 0.54 *^,†§^	1.90 ^§^	42	96		300.54 ± 0.48
**MNU ^l^**	50	NA ^o^	5 *^,†,‡,§,‖,┴^	4.55 ± 0.15 *^,‡,§,‖,┴^	4.48 ± 0.54	NA	NA	NA	100	305.44 ± 0.62
**C ^m^**		NA	0 ^†,‡,§,‖,┴^	0 ^†,‡,§,‖,┴^	0 ^†,‡,§,‖,┴^	NA	NA	NA	NA	210.54 ± 0.29
**Placebo ^n^**		NA	NA	NA	NA	NA	NA	NA	NA	300.54 ± 0.63
		NA	NA	NA	NA	NA	NA	NA	NA	325.43 ± 0.29

^a^ The average lipophilicity form the concentration range 5, 10, 20, 30, 40, and 50 mg/kg of bwt measured using shake-flask methodology; ^b^ Plasma protein binding (PPB) at 37 °C; ^c^ Intrinsic clearance in vivo; ^d^ Bioavailability of compound; ^e^ Maximum-tolerated-dose obtained after the 5-days per os administration in the concentration of 5, 50, 100, 500, and 1000 mg/kg bwt; ^f^ The effect of the orally administered compound at maximum-tolerated-dose; ^g^ The effect of the orally administered compound at maximum-feasible-dose on the body weight at day 1, showing the average body weight (mg) in placebo/control and the compound-treated rats; ^h^ The effect of the orally administered compound at maximum-feasible-dose on the bodyweight 5 days after starting treatment at the time of sacrifice, showing the average body weight (mg) in placebo/control and the compound-treated rats; ^i^ Results are presented as mean value ± standard deviation; ^j^ 4-hydroxytamoxifen; ^k^ Raloxifene; ^l^ Methyl nitrosourea; ^m^ 0.9% NaCl; ^n^ Vehicle; carboxymethylcellulose; ^o^ Not available. * *p* < 0.05 when compared with control group; ^†^ *p* < 0.05 when compared with MNU in concentration of 50 mg/kg; ^‡^ *p* < 0.05 when compared with **4-OTH** in concentration of 5 mg/kg; ^§^ *p* < 0.05 when compared with **4-OTH** in concentration of 50 mg/kg; ^‖^ *p* < 0.05 when compared with **Ral** in concentration of 5 mg/kg; ^┴^ *p* < 0.05 when compared with **Ral** in concentration of 50 mg/kg.

## Data Availability

All the experimental complexes used to build the 3-D pharmacophore and 3-D QSAR models, as well as the structure-based and ligand-based alignment assessments, can be retrieved free of charge from Protein Data Bank (https://www.rcsb.org/, accessed on 1 October 2015). All the compound structures used as test sets can be found in the Protein Data Bank or retrieved from the cited literature (see Supplementary Materials for specifics). All the computational results from 3-D pharmacophore and 3-D QSAR models studies and structure-based/ligand-based alignment assessments, as well as the UCSF Chimera sessions, are available from Milan Miladenović (files in machine-readable formats, e-mail: milan.mladenovic@pmf.kg.ac.rs). All the computational results regarding the design of new compounds can be obtained from Rino Ragno (e-mail: rino.ragno@uniroma1.it) and Milan Mladenović. Datasets for virtual screening can be obtained from National Cancer Institute (https://www.cancer.gov/, accessed on 1 October 2015). Open Access Software. The UCSF Chimera software, used for graphical analysis of 3-D QSAR models and structure-based and ligand-based aligned structures can be obtained free of charge at https://www.cgl.ucsf.edu/chimera/ (accessed on 1 October 2015). Marvin Beans for academics can be obtained free of charge at http://www.chemaxon.com (accessed on 1 October 2015). Commercial Software. Schrödinger Suite can be obtained from Canvas, Schrödinger, LLC, New York, NY. ChemDraw can be obtained from PerkinElmer Informatics (http://www.cambridgesoft.com/, accessed on 1 October 2015) and was herein used from drawing structures under the academic license bought by the University of Kragujevac, Faculty of Science, Milan Mladenović’s home institution. The Office365 package can be obtained from Microsoft Office (https://www.office.com/, accessed on 1 January 2022) and was herein used for writing and preparing figures under the academic license bought by the University of Kragujevac, Faculty of Science, Milan Mladenović’s home institution.

## Supplementary Materials

The following are available online at https://www.mdpi.com/article/10.3390/molecules27092823/s1. This material contains the Introduction (i.e., The Genomic classical pathway, Genomic indirect pathway, Tethered pathway alternative routes, Non-genomic pathways, Abbreviations, ERα 3-D pharmacophore models generation overview), Results and discussion (i.e., Tables and Figures describing data sets compilation, 3-D pharmacophore models, 3-D QSAR models, SB and LB alignment assessments, activity prediction of test sets, virtual screening, designed compounds SB and LB alignments, synthesized compounds spectral data interpretation, Figures of 1H NMR, ^13^C NMR, ^15^N NMR, ^17^O NMR, and HPLC spectral data of synthesized precursors and bioactive compounds, related tables with biochemical data), and Experimental section (i.e., the training set selection, preparation of antagonists-ERα complexes, interpretation of 3-D QSARs, SB and LB alignment assessment rules definition, virtual screening, equipment, commercial compounds supply, synthetic protocols, the in vitro and in vivo experimental protocols). Figure S1–S9: Data associated with the 3-D pharmacophore and 3-D QSAR model interpretation, Figures S10–S19: Data associated with the structure-based and ligand-based alignment assessments, Figures S20–S22: Data associated with the virtual screening, Figures S23–S25: Data associated with designed compounds binding conformations, Figures S26–S177: Data associated with synthesized compound ^1^H NMR, ^13^C NMR, ^15^N NMR, ^17^O NMR spectra, Figures S178–S190: Data associated with synthesized compound HPLC spectra, Figures S191–S224: Data associated with synthesized compound biological activity in vitro and in vivo, Tables S1–S6: Data associated with the 3-D pharmacophore and 3-D QSAR models development, Tables S7–S9: Data associated with the structure-based and ligand-based alignment assessments, Tables S10–S15: Data associated with the external validation of 3-D pharmacophore and 3-D QSAR models predictive abilities, Tables S16–S17: Data associated with the virtual screening, Table S18: Data associated with designed compounds binding conformations, Tables S19–S20: Data associated with the synthesized compounds’ toxicity.

## Sample Availability

Samples of the compounds **3DPQ-1** to **3DPQ-12** are available from the authors.
