# Supplementary material for: Human Estrogen Receptor Alpha Antagonists, Part 3: 3-D Pharmacophore and 3-D QSAR Guided Brefeldin A Hit-to-Lead Optimization toward New Breast Cancer Suppressants
Source: Molecules. 2022 Apr 28;27(9):2823. doi: 10.3390/molecules27092823 (PMC9101642; doi:10.3390/molecules27092823)
Supplement: Supplementary file 1 [file molecules-27-02823-s001.zip › molecules-1660584-supplementary.pdf]

Supplementary Materials for

Human Estrogen Receptor Alpha Antagonists, Part 3: 3-D Pharmacophore and 3-D QSAR Guided Brefeldin A Hit-To-Lead Optimization toward New Breast Cancer Suppressants

Nezrina Kurtanović [1], Nevena Tomašević [1], Sanja Matić [2], Elenora Proia[3], Manuela Sabatino[3], Lorenzo Antonini[3], Milan Mladenović[1]* and Rino Ragno[3]*

[1] Kragujevac Center for Computational Biochemistry, Department of Chemistry, Faculty of Science,
University of Kragujevac, Radoja Domanovića 12, P.O. Box 60, 34000 Kragujevac, Serbia;
nezrina.mihovic@pmf.kg.ac.rs (N.K.); nevena.stankovic@pmf.kg.ac.rs (N.T.)
[2] Institute for Informational Technologies Kragujevac, University of Kragujevac, Jovana Cvijića bb,
34000 Kragujevac, Serbia; sanjamatic@kg.ac.rs
[3] Rome Center for Molecular Design, Department of Drug Chemistry and Technology, Faculty of Pharmacy and Medicine, Sapienza University of Rome, P.le A. Moro 5, 00185 Rome, Italy;
eleonora.proia@uniroma1.it (E.P.); manuela.sabatino@uniroma1.it (M.S.);
lorenzo.antonini@uniroma1.it (L.A.)
* Correspondence: milan.mladenovic@pmf.kg.ac.rs (M.M.); rino.ragno@uniroma1.it (R.R.);
Tel.: +381-34336223 (M.M.); Tel.: +39-49913937 (R.R.)

Content

Introduction

Genomic Classical Pathway [6-8]
Genomic Indirect Pathway y[6-8]
Tethered Pathway Alternative Routes [6-8]
Non-Genomic Pathways [6-8]
Abbreviations
ERα 3-D Pharmacophore Models Generation Overview.

Results and discussion

Table S1. PDB codes and ligand structures of Estrogen Receptor *α* agonists, partial agonists, and antagonists currently available at Protein Data Bank (date of access 2015).
Table S2. Scores of the different parameters (the upper part) and PLS statistical parameters (the lower part) of the top ten hypotheses.
Table S3. Experimental *vs*. recalculated and predicted training set $pIC_{50}s$ values using the **ADDHHHP.13** hypothesis and LOO cross-validation.
Table S4. Experimental *vs*. recalculated and predicted training set $pIC_{50}s$ values using the **ADDRRRP.11** hypothesis and LOO cross-validation.
Table S5. Experimental *vs*. recalculated and predicted training set $pIC_{50}s$ values using the **ADDHHHP.13** hypothesis and LSO cross-validation.
Table S6. Experimental *vs*. recalculated and predicted training set $pIC_{50}s$ values using the **ADDRRRP.11** hypothesis and LSO cross-validation.

The Origin/Significance of the ***D₁*** *Feature and the Interrelated PLS-Coefficients.*
The Origin/Significance of the ***D₂*** *Feature and the Interrelated PLS-Coefficients*
The Origin/Significance of the ***H₁/R₁*** *Feature and the Interrelated PLS-Coefficients*
The Origin/Significance of the ***H₂/R₂*** *Feature and the Interrelated PLS-Coefficients*
The Origin/Significance of the ***H₃/R₃*** *Feature and the Interrelated PLS-Coefficients*
The Origin/Significance of the ***A*** *Feature and the Interrelated PLS-Coefficients*
The Origin/Significance of The ***P*** *Feature And The Interrelated PLS-Coefficients*
Alignment Assessments Rules.
Structure-Based Alignment Assessments.
Ligand-Based Alignment Assessments.

Table S7. Structure-based alignment assessment for wild-type and mutated ER*α* antagonists.
Table S8. Ligand-based alignment assessment for wild-type antagonists.
Table S9. Ligand-based alignment assessment for mutated (MT) ER*α* antagonists.

Predictive Ability Assessment of the 3-D PhypI/3-D QSAR Model Ensemble.

Table S10. Chemical structures, activity, and structure-based predictions of a test set complied of compounds which activity is in literature expressed as pIC_{50} using the **3DPhypI/3-D QSAR model** enable.

Table S11. Chemical structures, activity, and ligand-based predictions of a test set complied of compounds which activity is in literature expressed as pIC_{50} using the **3DPhypI/3-D QSAR model** enable.

Table S12. Chemical structures, activity, and structure-based predictions of a test set complied of compounds which activity is in literature expressed as pK_i using the **3DPhypI/3-D QSAR model** enable.

Table S13. Chemical structures, activity, and ligand-based predictions of a test set complied of compounds which activity is in literature expressed as pK_i using the **3DPhypI/3-D QSAR model** enable.

Table S14. Chemical structures, activity, and structure-based predictions of a test set complied of compounds which activity is in literature expressed as pRBA using the **3DPhypI/3-D QSAR model** enable.

Table S15. Chemical structures, activity and ligand-based predictions of test set complied of compounds which activity is in literature expressed as pRBA using the **3DPhypI/3-D QSAR model** enable.

Table S16. NCI Codes, ligand structures, binding affinities of 18 virtually screened partial agonists against either wild type (WT) or mutated (MUT) Estrogen Receptor α, and average scores of LB alignment against the TR compounds

Table S17. Virtually screened hits SB and LB predicted and experimental antagonistic activities against ERα, antiproliferative activity, and selectivity index against hormone-dependent MCF-7 and hormone-independent MDA-MB-231 breast cancer cell lines, antiproliferative activity against the normal MRC-5 human lung tissue fibroblasts cell lines.

Table S18. The molecular docking studies analysis in terms of hydrogen bonding with the crucial LBD residues.

Table S19. Effects of synthesized compounds-based treatment on mammary tumorigenesis seen through the serum biochemical markers.

Table S20. Effects of synthesized compounds-based treatment of mammary tumorigenesis seen through the liver oxidative stress markers.

Figure S1. The **3-D PhypI** features (**D**: hydrogen-bond donators, **A**: hydrogen-bond acceptors, **H**: hydrophobic features, **P**: positive ionizable features) and **3-D QSAR** *PLS-coefficients* contour maps (**GREEN***PLS-coefficients*: positive steric interactions, **YELLOW***PLS-coefficients*; negative steric interactions, **BLUE***PLS-coefficients*: areas where positively charged functional groups and H-bond donators are favored whereas the negatively charged functional groups and H-bond acceptors are disfavored, **RED***PLS-coefficients*: areas negatively charged functional groups and H-bond acceptors are favored, whereas the positively charged functional groups and H-bond donators are disfavored) for **1L2I** (**A**), **5AK2** (**B**), **2IOG** (**C**), **1X7R** (**D**), **1UOM** (**E**), and **1GWQ** (**F**). Amino acid residues are depicted in white. For the clarity of presentation, only the H12 helix is presented in a cornflower blue ribbon, as a crucial delimiter for partial agonists, SERMs, and SERDs.

Figure S2. The **3-D PhypI** features (**D**: hydrogen-bond donators, **A**: hydrogen-bond acceptors, **H**: hydrophobic features, **P**: positive ionizable features) and **3-D QSAR** *PLS-coefficients* contour maps (**GREEN***PLS-coefficients*: positive steric interactions, **YELLOW***PLS-coefficients*; negative steric interactions, **BLUE***PLS-coefficients*: areas where positively charged functional groups and H-bond donators are favored whereas the negatively charged functional groups and H-bond acceptors are disfavored, **RED***PLS-coefficients*: areas negatively charged functional groups and H-bond acceptors are favored, whereas the positively charged functional groups and H-bond donators are disfavored) for **1XP6** (A), **1SJ0** (B), **2R6Y** (C), **1XP9** (D), **1YIM** (E), and **1YIN** (F). Amino acid residues are depicted in white. For the clarity of presentation, only H12 helix is presented in cornflower blue ribbon, as a crucial delimiter for partial agonists, SERMs, and SERDs.

Figure S3. The **3-D PhypI** features (**D**: hydrogen-bond donators, **A**: hydrogen-bond acceptors, **H**: hydrophobic features, **P**: positive ionizable features) and **3-D QSAR** *PLS-coefficients* contour maps (**GREEN***PLS-coefficients*: positive steric interactions, **YELLOW***PLS-coefficients*; negative steric interactions, **BLUE***PLS-*

coefficients: areas where positively charged functional groups and H-bond donators are favored whereas the negatively charged functional groups and H-bond acceptors are disfavored, **RED***PLS-coefficients*: areas negatively charged functional groups and H-bond acceptors are favored, whereas the positively charged functional groups and H-bond donators are disfavored) for **1XPC** (A), **2R6W** (B), **2QA8** (C), and **1R5K** (D). Amino acid residues are depicted in white. For the clarity of presentation, only H12 helix is presented in cornflower blue ribbon, as a crucial delimiter for partial agonists, SERMs, and SERDs.

Figure S4. The **3-D PhypI** features (**D**: hydrogen-bond donators, **A**: hydrogen-bond acceptors, **H**: hydrophobic features, **P**: positive ionizable features) and **3-D QSAR** *PLS-coefficients* contour maps (**GREEN***PLS-coefficients*: positive steric interactions, **YELLOW***PLS-coefficients*; negative steric interactions, **BLUE***PLS-coefficients*: areas where positively charged functional groups and H-bond donators are favored whereas the negatively charged functional groups and H-bond acceptors are disfavored, **RED***PLS-coefficients*: areas negatively charged functional groups and H-bond acceptors are favored, whereas the positively charged functional groups and H-bond donators are disfavored) for **2QA6** (A), **1XQC** (B), **2B1Z** (C), and **1X7E** (D). Amino acid residues are depicted in white. For the clarity of presentation, only H12 helix is presented in cornflower blue ribbon, as a crucial delimiter for partial agonists, SERMs, and SERDs.

Figure S5. The **3-D PhypII** features (**D**: hydrogen-bond donators, **A**: hydrogen-bond acceptors, **R**: ring features, **P**: positive ionizable features) and **3-D QSAR** *PLS-coefficients* contour maps (**GREEN***PLS-coefficients*: positive steric interactions, **YELLOW***PLS-coefficients*: negative steric interactions, **BLUE***PLS-coefficients*: areas where positively charged functional groups and H-bond donators are favored whereas the negatively charged functional groups and H-bond acceptors are disfavored, **RED***PLS-coefficients*: areas negatively charged functional groups and H-bond acceptors are favored, whereas the positively charged functional groups and H-bond donators are disfavored) for **1ERR** (A); **3ERD** (B); **1XP1** (C); **1ERE** (D); **2IOK** (E); **2BJ4** (F). Amino acid residues are depicted in white. For the clarity of presentation, only H12 helix is presented in cornflower blue ribbon, as a crucial delimiter for partial agonists, SERMs, and SERDs.

Figure S6. The **3-D PhypII** features (**D**: hydrogen-bond donators, **A**: hydrogen-bond acceptors, **R**: ring features, **P**: positive ionizable features) and **3-D QSAR** *PLS-coefficients* contour maps (**GREEN***PLS-coefficients*: positive steric interactions, **YELLOW***PLS-coefficients*: negative steric interactions, **BLUE***PLS-coefficients*: areas where positively charged functional groups and H-bond donators are favored whereas the negatively charged functional groups and H-bond acceptors are disfavored, **RED***PLS-coefficients*: areas negatively charged functional groups and H-bond acceptors are favored, whereas the positively charged functional groups and H-bond donators are disfavored)) for **1L2I** (A), **5AK2** (B), **2IOG** (C), **1X7R** (D), **1UOM** (E), and **1GWQ** (F). Amino acid residues are depicted in white. For the clarity of presentation, only H12 helix is presented in cornflower blue ribbon, as a crucial delimiter for partial agonists, SERMs, and SERDs.

Figure S7. The **3-D PhypII** features (**D**: hydrogen-bond donators, **A**: hydrogen-bond acceptors, **R**: ring features, **P**: positive ionizable features) and **3-D QSAR** *PLS-coefficients* contour maps (**GREEN***PLS-coefficients*: positive steric interactions, **YELLOW***PLS-coefficients*: negative steric interactions, **BLUE***PLS-coefficients*: areas where positively charged functional groups and H-bond donators are favored whereas the negatively charged functional groups and H-bond acceptors are disfavored, **RED***PLS-coefficients*: areas negatively charged functional groups and H-bond acceptors are favored, whereas the positively charged functional groups and H-bond donators are disfavored) for **1XP6** (A), **1SJ0** (B), **2R6Y** (C), **1XP9** (D), **1YIM** (E), and **1YIN** (F). Amino acid residues are depicted in white. For the clarity of presentation, only H12 helix is presented in cornflower blue ribbon, as a crucial delimiter for partial agonists, SERMs, and SERDs.

Figure S8. The **3-D PhypII** features (**D**: hydrogen-bond donators, **A**: hydrogen-bond acceptors, **R**: ring features, **P**: positive ionizable features) and **3-D QSAR** *PLS-coefficients* contour maps (**GREEN***PLS-coefficients*: positive steric interactions, **YELLOW***PLS-coefficients*: negative steric interactions, **BLUE***PLS-coefficients*: areas where positively charged functional groups and H-bond donators are favored whereas the negatively charged functional groups and H-bond acceptors are disfavored, **RED***PLS-coefficients*: areas negatively charged functional groups and H-bond acceptors are favored, whereas the positively charged functional groups

and H-bond donators are disfavored) for **1XPC** (A), **2R6W** (B), **2QA8** (C), and **1R5K** (D). Amino acid residues are depicted in white. For the clarity of presentation, only H12 helix is presented in cornflower blue ribbon, as a crucial delimiter for partial agonists, SERMs, and SERDs.

Figure S9. The **3-D PhypII** features (**D**: hydrogen-bond donators, **A**: hydrogen-bond acceptors, **R**: ring features, **P**: positive ionizable features) and **3-D QSAR** *PLS-coefficients* contour maps (**GREEN***PLS-coefficients*: positive steric interactions, **YELLOW***PLS-coefficients*: negative steric interactions, **BLUE***PLS-coefficients*: areas where positively charged functional groups and H-bond donators are favored whereas the negatively charged functional groups and H-bond acceptors are disfavored, **RED***PLS-coefficients*: areas negatively charged functional groups and H-bond acceptors are favored, whereas the positively charged functional groups and H-bond donators are disfavored) for **2QA6** (A), **1XQC** (B), **2B1Z** (C), and **1X7E** (D). Amino acid residues are depicted in white. For the clarity of presentation, only H12 helix is presented in cornflower blue ribbon, as a crucial delimiter for partial agonists, SERMs, and SERDs.

Figure S10. Structure-based alignment assessment of ERα partial agonists, SERMs, and SERDs using Glide's XP module: **1ERR** (A), EC blue, ECRD yellow, RCRD magenta, ECCD blue, RCCD black; **3ERD** (B), EC black, ECRD yellow, RCRD magenta, ECCD blue, RCCD black; **1XP1** (C), EC light green, ECRD yellow, RCRD magenta, ECCD blue, RCCD black; **1ERE** (D), EC violet, ECRD yellow, RCRD magenta, ECCD blue, RCCD black; **2IOK** (E), EC light green, ECRD yellow, RCRD magenta, ECCD blue, RCCD black; and **2BJ4** (F) EC purple, ECRD yellow, RCRD magenta, ECCD blue, RCCD black. Amino acid residues are depicted in white. For the clarity of presentation, only H12 helix is presented in cornflower blue ribbon, as a crucial delimiter for partial agonists, SERMs, and SERDs.

Figure S11. Structure-based alignment assessment of ERα partial agonists, SERMs, and SERDs using Glide's XP module: **1L2I** (A), EC dark blue, ECRD yellow, RCRD magenta, ECCD blue, RCCD black; **5AK2** (B), EC dark blue, ECRD yellow, RCRD magenta, ECCD blue, RCCD black; **2IOG** (C), EC dark green, ECRD yellow, RCRD magenta, ECCD blue, RCCD black; **1X7R** (D), EC purple, ECRD yellow, RCRD magenta, ECCD blue, RCCD black; **1UOM** (E), EC purple, ECRD yellow, RCRD magenta, ECCD blue, RCCD black; and **1GWQ** (F) EC gray, ECRD yellow, RCRD magenta, ECCD blue, RCCD black. Amino acid residues are depicted in white. For the clarity of presentation, only H12 helix is presented in cornflower blue ribbon, as a crucial delimiter for partial agonists, SERMs, and SERDs.

Figure S12. Structure-based alignment assessment of ERα partial agonists, SERMs, and SERDs using Glide's XP module: **1XP6** (A), EC brown, ECRD yellow, RCRD magenta, ECCD blue, RCCD black; **1SJ0** (B), EC dark blue, ECRD yellow, RCRD magenta, ECCD blue, RCCD black; **2R6Y** (C), EC light blue, ECRD yellow, RCRD magenta, ECCD blue, RCCD black; **1XP9** (D), EC blue, ECRD yellow, RCRD magenta, ECCD blue, RCCD black; **1YIM** (E), EC purple, ECRD yellow, RCRD magenta, ECCD blue, RCCD black; and **1YIN** (F) EC purple, ECRD yellow, RCRD magenta, ECCD blue, RCCD black. Amino acid residues are depicted in white. For the clarity of presentation, only H12 helix is presented in cornflower blue ribbon, as a crucial delimiter for partial agonists, SERMs, and SERDs.

Figure S13. Structure-based alignment assessment of ERα partial agonists, SERMs, and SERDs using Glide's XP module: **1XPC** (A), EC gray, ECRD yellow, RCRD magenta, ECCD blue, RCCD black; **2R6W** (B), EC dark red, ECRD yellow, RCRD magenta, ECCD blue, RCCD black; **2QA8** (C), EC orange, ECRD yellow, RCRD magenta, ECCD blue, RCCD black; and **1R5K** (D) EC dark green, ECRD yellow, RCRD magenta, ECCD blue, RCCD black. Amino acid residues are depicted in white. For the clarity of presentation, only H12 helix is presented in cornflower blue ribbon, as a crucial delimiter for partial agonists, SERMs, and SERDs.

Figure S14. Structure-based alignment assessment of ERα partial agonists, SERMs, and SERDs using Glide's XP module: **2QA6** (A), EC light blue, ECRD yellow, RCRD magenta, ECCD blue, RCCD black; **1XQC** (B), EC light green, ECRD yellow, RCRD magenta, ECCD blue, RCCD black; **2B1Z** (C), EC light pink, ECRD yellow, RCRD magenta, ECCD blue, RCCD black; and **1X7E** (D) EC yellow, ECRD yellow, RCRD magenta, ECCD blue, RCCD black. Amino acid residues are depicted in white. For the clarity of

presentation, only H12 helix is presented in cornflower blue ribbon, as a crucial delimiter for partial agonists, SERMs, and SERDs.

Figure S15. Ligand-based alignment assessment of ERα partial agonists, SERMs, and SERDs virtually superimposed to ERα active site using Flexible Ligand Alignment tool's FSBA module: **1ERR** (A), EC blue, ECRA yellow, RCRA magenta, ECCA blue, RCCA black; **3ERD** (B), EC black, ECRA yellow, RCRA magenta, ECCA blue, RCCA black; **1XP1** (C), EC light green, ECRA yellow, RCRA magenta, ECCA blue, RCCA black; **1ERE** (D), EC violet, ECRA yellow, RCRA magenta, ECCA blue, RCCA black; **2IOK** (E), EC light green, ECRA yellow, RCRA magenta, ECCA blue, RCCA black; and **2BJ4** (F) EC purple, ECRA yellow, RCRA magenta, ECCA blue, RCCA black. Amino acid residues are depicted in white. For the clarity of presentation, only H12 helix is presented in cornflower blue ribbon, as a crucial delimiter for partial agonists, SERMs, and SERDs.

Figure S16. Ligand-based alignment assessment of ERα partial agonists, SERMs, and SERDs virtually superimposed to ERα active site using Flexible Ligand Alignment tool's FSBA module: **1L2I** (A), EC dark blue, ECRA yellow, RCRA magenta, ECCA blue, RCCA black; **5AK2** (B), EC dark blue, ECRA yellow, RCRA magenta, ECCA blue, RCCA black; **2IOG** (C), EC dark green, ECRA yellow, RCRA magenta, ECCA blue, RCCA black; **1X7R** (D), EC purple, ECRA yellow, RCRA magenta, ECCA blue, RCCA black; **1UOM** (E), EC purple, ECRA yellow, RCRA magenta, ECCA blue, RCCA black; and **1GWQ** (F) EC gray, ECRA yellow, RCRA magenta, ECCA blue, RCCA black. Amino acid residues are depicted in white. For the clarity of presentation, only H12 helix is presented in cornflower blue ribbon, as a crucial delimiter for partial agonists, SERMs, and SERDs.

Figure S17. Ligand-based alignment assessment of ERα partial agonists, SERMs, and SERDs virtually superimposed to ERα active site using Flexible Ligand Alignment tool's FSBA module: **1XP6** (A), EC brown, ECRA yellow, RCRA magenta, ECCA blue, RCCA black; **1SJ0** (B), EC dark blue, ECRA yellow, RCRA magenta, ECCA blue, RCCA black; **2R6Y** (C), EC light blue, ECRA yellow, RCRA magenta, ECCA blue, RCCA black; **1XP9** (D), EC blue, ECRA yellow, RCRA magenta, ECCA blue, RCCA black; **1YIM** (E), EC purple, ECRA yellow, RCRA magenta, ECCA blue, RCCA black; and **1YIN** (F) EC purple, ECRA yellow, RCRA magenta, ECCA blue, RCCA black. Amino acid residues are depicted in white. For the clarity of presentation, only H12 helix is presented in cornflower blue ribbon, as a crucial delimiter for partial agonists, SERMs, and SERDs.

Figure S18. Ligand-based alignment assessment of ERα partial agonists, SERMs, and SERDs virtually superimposed to ERα active site using Flexible Ligand Alignment tool's FSBA module: **1XPC** (A), EC gray, ECRA yellow, RCRA magenta, ECCA blue, RCCA black; **2R6W** (B), EC dark red, ECRA yellow, RCRA magenta, ECCA blue, RCCA black; **2QA8** (C), EC orange, ECRA yellow, RCRA magenta, ECCA blue, RCCA black; and **1R5K** (D) EC dark green ECRA yellow, RCRA magenta, ECCA blue, RCCA black. Amino acid residues are depicted in white. For the clarity of presentation, only H12 helix is presented in cornflower blue ribbon, as a crucial delimiter for partial agonists, SERMs, and SERDs.

Figure S19. Ligand-based alignment assessment of ERα partial agonists, SERMs, and SERDs virtually superimposed to ERα active site using Flexible Ligand Alignment tool's FSBA module: **2QA6** (A), EC light blue, ECRA yellow, RCRA magenta, ECCA blue, RCCA black; **1XQC** (B), EC light green, ECRA yellow, RCRA magenta, ECCA blue, RCCA black; **2B1Z** (C), EC light pink, ECRA yellow, RCRA magenta, ECCA blue, RCCA black; and **1X7E** (D) EC yellow ECRA yellow, RCRA magenta, ECCA blue, RCCA black. Amino acid residues are depicted in white. For the clarity of presentation, only H12 helix is presented in cornflower blue ribbon, as a crucial delimiter for partial agonists, SERMs, and SERDs.

Figure 20. The SB/LB virtually screened conformations of **NCI89671**, SB conformation blue, LB conformation pink (A); **NCI101789**, SB conformation light green, LB conformation purple (B); **NCI645330**, SB conformation red, LB conformation green (C); **NCI59814**, SB conformation light blue, LB conformation pink (D); **NCI76747**, SB conformation pink, LB conformation yellow (E); **NCI9782**, SB conformation purple, LB conformation orange (F), incorporated into the **3-D PhypI/3-D QSAR model** prediction

engine. Amino acid residues are depicted in white. For the clarity of presentation, only H12 helix is presented in a cornflower blue ribbon, as a crucial delimiter for partial agonists, SERMs, and SERDs.

Figure S21. The SB/LB virtually screened conformations of **NCI71795** SB conformation green, LB conformation orange (A); **NCI85239**, SB conformation green, LB conformation yellow (B); **NCI69359**, SB conformation coral, LB conformation green (C); **NCI16437**, SB conformation green, LB conformation blue (D); **NCI17128**, SB conformation purple, LB conformation red (E); and **NCI10211**, SB conformation green, LB conformation blue (F), incorporated into the **3-D PhypI/3-D QSAR model** prediction engine. Amino acid residues are depicted in white. For the clarity of presentation, only H12 helix is presented in a cornflower blue ribbon, as a crucial delimiter for partial agonists, SERMs, and SERDs.

Figure S22. The SB/LB virtually screened conformations of **NCI30813**, SB conformation blue, LB conformation red (A); **NCI10416**, SB conformation blue, LB conformation red (B); **NCI6145**, SB conformation blue, LB conformation red (C); **NCI19824**, SB conformation red, LB conformation blue (D); **NCI36586**, SB conformation blue, LB conformation red (E); and **NCI93427**, SB conformation blue, LB conformation red (F), incorporated into the **3-D PhypI/3-D QSAR model** prediction engine. Amino acid residues are depicted in white. For the clarity of presentation, only H12 helix is presented in a cornflower blue ribbon, as a crucial delimiter for partial agonists, SERMs, and SERDs.

Figure S23. The superimposition between the designed compounds **3DPQ-12** (A), SBC light green-blue, LBC pink; **3DPQ-3** (B), SBC brown, LBC orchid; **3DPQ-9** (C), SBC dark green-blue, LBC pink; **3DPQ-4** (D), SBC blue, LBC brown; **3DPQ-2** (E), SBC red, LBC green; **3DPQ-1** (F). SBC blue, LBC yellow-green; and **1ERE**, EC blue, within the ERα active site. Amino acid residues are depicted in white. For the clarity of presentation, only H12 helix is presented in cornflower blue ribbon, as a crucial delimiter for partial agonists, SERMs, and SERDs.

Figure S24. The superimposition between the designed compounds **3DPQ-7** (A), SBC blue, LBC green; **3DPQ-11** (B), SBC purple, LBC pink; **3DPQ-10** (C), SBC green, LBC red; **3DPQ-5** (D), SBC blue, LBC orange; **3DPQ-8** (E), SBC pink, LBC green; **3DPQ-6** (F). SBC green, LBC yellow; and **1ERE**, EC blue, within the ERα active site. Amino acid residues are depicted in white. For the clarity of presentation, only H12 helix is presented in cornflower blue ribbon, as a crucial delimiter for partial agonists, SERMs, and SERDs.

Figure S25. The bioactive conformations of **3DPQ-7** (A); **3DPQ-11** (B); **3DPQ-10** (C); **3DPQ-5** (D); **3DPQ-8** (E); **3DPQ-6** (F) within the ERα active site. Amino acid residues are depicted in white, H12 helix is presented in cornflower blue ribbon.

Synthesized Compounds Spectral Data Interpretation.

Figure S26. The ^{1}H NMR spectrum of **R1**.
Figure S27. The ^{13}C NMR spectrum of **R1**.
Figure S28. The ^{17}O NMR spectrum of **R1**.
Figure S29. The ^{1}H NMR spectrum of **R2**.
Figure S30. The ^{13}C NMR spectrum of **R2**.
Figure S31. The ^{17}O NMR spectrum of **R2**.
Figure S32. The ^{1}H NMR spectrum of **R3**.
Figure S33. The ^{13}C NMR spectrum of **R3**.
Figure S34. The ^{17}O NMR spectrum of **R3**.
Figure S35. The ^{1}H NMR spectrum of **R4**.
Figure S36. The ^{13}C NMR spectrum of **R4**.
Figure S37. The ^{15}N NMR spectrum of **R4**.
Figure S38. The ^{17}O NMR spectrum of **R4**.
Figure S39. The ^{1}H NMR spectrum of **R5**.

Figure S40. The ^{13}C NMR spectrum of **R5**.
Figure S41. The ^{15}N NMR spectrum of **R5**.
Figure S42. The ^{17}O NMR spectrum of **R5**.
Figure S43. The ^{1}H NMR spectrum of **R6**.
Figure S44. The ^{13}C NMR spectrum of **R6**.
Figure S45. The ^{17}O NMR spectrum of **R6**.
Figure S46. The ^{1}H NMR spectrum of **R7**.
Figure S47. The ^{13}C NMR spectrum of **R7**.
Figure S48. The ^{17}O NMR spectrum of **R7**.
Figure S49. The ^{1}H NMR spectrum of **R8**.
Figure S50. The ^{13}C NMR spectrum of **R8**.
Figure S51. The ^{17}O NMR spectrum of **R8**.
Figure S52. The ^{1}H NMR spectrum of **BFA-D1**.
Figure S53. The ^{13}C NMR spectrum of **BFA-D1**.
Figure S54. The ^{17}O NMR spectrum of **BFA-D1**.
Figure S55. The ^{1}H NMR spectrum of **BFA-D2**.
Figure S56. The ^{13}C NMR spectrum of **BFA-D2**.
Figure S57. The ^{17}O NMR spectrum of **BFA-D2**.
Figure S58. The ^{1}H NMR spectrum of **BFA-D3**.
Figure S59. The ^{13}C NMR spectrum of **BFA-D3**.
Figure S60. The ^{17}O NMR spectrum of **BFA-D3**.
Figure S61. The ^{1}H NMR spectrum of **BFA-D4**.
Figure S62. The ^{13}C NMR spectrum of **BFA-D4**.
Figure S63. The ^{17}O NMR spectrum of **DFA-D4**.
Figure S64. The ^{1}H NMR spectrum of **Pro-R3**.
Figure S65. The ^{13}C NMR spectrum of **Pro-R3**.
Figure S66. The ^{15}N NMR spectrum of **Pro-R3**.
Figure S67. The ^{17}O NMR spectrum of **Pro-R3**.
Figure S68. The ^{1}H NMR spectrum of **Pro-R6**.
Figure S69. The ^{13}C NMR spectrum of **Pro-R6**.
Figure S70. The ^{15}N NMR spectrum of **Pro-R6**.
Figure S71. The ^{1}H NMR spectrum of **Pro-R7**.
Figure S72. The ^{13}C NMR spectrum of **Pro-R7**.
Figure S73. The ^{15}N NMR spectrum of **Pro-R7**.
Figure S74. The ^{1}H NMR spectrum of **Pro-R8**.
Figure S75. The ^{13}C NMR spectrum of **Pro-R8**.
Figure S76. The ^{15}N NMR spectrum of **Pro-R8**.
Figure S77. The ^{1}H NMR spectrum of **Pro-R9**.
Figure S78. The ^{13}C NMR spectrum of **Pro-R9**.
Figure S79. The ^{15}N NMR spectrum of **Pro-R9**.
Figure S80. The ^{17}O NMR spectrum of **Pro-R9**.
Figure S81. The ^{1}H NMR spectrum of **Pro-R12**.
Figure S82. The ^{13}C NMR spectrum of **Pro-R12**.
Figure S83. The ^{15}N NMR spectrum of **Pro-R12**.
Figure S84. The ^{17}O NMR spectrum of **Pro-R12**.
Figure S85. The ^{1}H NMR spectrum of **Pro-3DPQ-1**.
Figure S86. The ^{13}C NMR spectrum of **Pro-3DPQ-1**.
Figure S87. The ^{13}N NMR spectrum of **Pro-3DPQ-1**.

Figure S88. The ^{13}O NMR spectrum of **Pro-3DPQ-1**.
Figure S89. The ^{1}H NMR spectrum of **Pro-3DPQ-2**.
Figure S90. The ^{13}C NMR spectrum of **Pro-3DPQ-2.**
Figure S91. The ^{15}N NMR spectrum of **Pro-3DPQ-2**.
Figure S92. The ^{17}O NMR spectrum of **Pro-3DPQ-2**.
Figure S93. The ^{1}H NMR spectrum of **Pro-3DPQ-3**.
Figure S94. The ^{13}C NMR spectrum of **Pro-3DPQ-3**.
Figure S95. The ^{15}N NMR spectrum of **Pro-3DPQ-3**.
Figure S96. The ^{17}O NMR spectrum of **Pro-3DPQ-3**.
Figure S97. The ^{1}H NMR spectrum of **Pro-3DPQ-4**.
Figure S98. The ^{13}C NMR spectrum of **Pro-3DPQ-4**.
Figure S99. The ^{17}O NMR spectrum of **Pro-3DPQ-4**.
Figure S100. The ^{1}H NMR spectrum of **Pro-3DPQ-5**.
Figure S101. The ^{13}C NMR spectrum of **Pro-3DPQ-5**.
Figure S102. The ^{15}N NMR spectrum of **Pro-3DPQ-5**.
Figure S103. The ^{17}O NMR spectrum of **Pro-3DPQ-5**.
Figure S104. The ^{1}H NMR spectrum of **Pro-3DPQ-6**.
Figure S105. The ^{13}C NMR spectrum of **Pro-3DPQ-6**.
Figure S106. The ^{15}N NMR spectrum of **Pro-3DPQ-6**.
Figure S107. The ^{17}O NMR spectrum of **Pro-3DPQ-6**.
Figure S108. The ^{1}H NMR spectrum of **Pro-3DPQ-7**.
Figure S109. The ^{13}C NMR spectrum of **Pro-3DPQ-7**.
Figure S110. The ^{15}N NMR spectrum of **Pro-3DPQ-7**.
Figure S111. The ^{17}O NMR spectrum of **Pro-3DPQ-7**.
Figure S112. The ^{1}H NMR spectrum of **Pro-3DPQ-8**.
Figure S113. The ^{13}C NMR spectrum of **Pro-3DPQ-8**.
Figure S114. The ^{15}N NMR spectrum of **Pro-3DPQ-8**.
Figure S115. The ^{17}O NMR spectrum of **Pro-3DPQ-8**.
Figure S116. The ^{1}H NMR spectrum of **Pro-3DPQ-9**.
Figure S117. The ^{13}C NMR spectrum of **Pro-3DPQ-9**.
Figure S118. The ^{15}N NMR spectrum of **Pro-3DPQ-9**.
Figure S119. The ^{17}O NMR spectrum of **Pro-3DPQ-9**.
Figure S120. The ^{1}H NMR spectrum of **Pro-3DPQ-10**.
Figure S121. The ^{13}C NMR spectrum of **Pro-3DPQ-10**.
Figure S122. The ^{15}N NMR spectrum of **Pro-3DPQ-10**.
Figure S123. The ^{17}O NMR spectrum of **Pro-3DPQ-10**.
Figure S124. The ^{1}H NMR spectrum of **Pro-3DPQ-11**.
Figure S125. The ^{13}C NMR spectrum of **Pro-3DPQ-11**.
Figure S126. The ^{15}N NMR spectrum of **Pro-3DPQ-11**.
Figure S127. The ^{17}O NMR spectrum of **Pro-3DPQ-11**.
Figure S128. The ^{1}H NMR spectrum of **Pro-3DPQ-12**.
Figure S129. The ^{13}C NMR spectrum of **Pro-3DPQ-12**.
Figure S130. The ^{15}N NMR spectrum of **Pro-3DPQ-12**.
Figure S131. The ^{17}O NMR spectrum of **Pro-3DPQ-12**.
Figure S132. The ^{1}H NMR spectrum of **3DPQ-1**.
Figure S133. The ^{13}C NMR spectrum of **3DPQ-1**.
Figure S134. The ^{15}N NMR spectrum of **3DPQ-1**.
Figure S135. The ^{17}O NMR spectrum of **3DPQ-1**.

Figure S136. The ^{1}H NMR spectrum of **3DPQ-2**.
Figure S137. The ^{13}C NMR spectrum of **3DPQ-2**.
Figure S138. The ^{15}N NMR spectrum of **3DPQ-2**.
Figure S139. The ^{17}O NMR spectrum of **3DPQ-2**.
Figure S140. The ^{1}H NMR spectrum of **3DPQ-3**.
Figure S141. The ^{13}C NMR spectrum of **3DPQ-3**.
Figure S142. The ^{15}N NMR spectrum of **3DPQ-3**.
Figure S143. The ^{17}O NMR spectrum of **3DPQ-3**.
Figure S144. The ^{1}H NMR spectrum of **3DPQ-4**.
Figure S145. The ^{13}C NMR spectrum of **3DPQ-4**.
Figure S146. The ^{17}O NMR spectrum of **3DPQ-4**.
Figure S147. The ^{1}H NMR spectrum of **3DPQ-5**.
Figure S148. The ^{13}C NMR spectrum of **3DPQ-5**.
Figure S149. The ^{15}N NMR spectrum of **3DPQ-5**.
Figure S150. The ^{17}O NMR spectrum of **3DPQ-5**.
Figure S151. The ^{1}H NMR spectrum of **3DPQ-6**.
Figure S152. The ^{13}C NMR spectrum of **3DPQ-6**.
Figure S153. The ^{15}N NMR spectrum of **3DPQ-6**.
Figure S154. The ^{17}O NMR spectrum of **3DPQ-6**.
Figure S155. The ^{1}H NMR spectrum of **3DPQ-7**.
Figure S156. The ^{13}C NMR spectrum of **3DPQ-7**.
Figure S157. The ^{15}N NMR spectrum of **3DPQ-7**.
Figure S158. The ^{17}O NMR spectrum of **3DPQ-7**.
Figure S159. The ^{1}H NMR spectrum of **3DPQ-8**.
Figure S160. The ^{13}C NMR spectrum of **3DPQ-8**.
Figure S161. The ^{15}N NMR spectrum of **3DPQ-8**.
Figure S162. The ^{17}O NMR spectrum of **3DPQ-8**.
Figure S163. The ^{1}H NMR spectrum of **3DPQ-9**.
Figure S164. The ^{13}C NMR spectrum of **3DPQ-9**.
Figure S165. The ^{15}N NMR spectrum of **3DPQ-9**.
Figure S166. The ^{17}O NMR spectrum of **3DPQ-9**.
Figure S167. The ^{1}H NMR spectrum of **3DPQ-10**
Figure S168. The ^{13}C NMR spectrum of **3DPQ-10**.
Figure S169. The ^{15}N NMR spectrum of **3DPQ-10**.
Figure S170. The ^{17}O NMR spectrum of **3DPQ-10**.
Figure S171. The ^{1}H NMR spectrum of **3DPQ-11**.
Figure S172. The ^{13}C NMR spectrum of **3DPQ-11**.
Figure S173. The ^{15}N NMR spectrum of **3DPQ-11**.
Figure S174. The ^{17}O NMR spectrum of **3DPQ-11**.
Figure S175. The ^{1}H NMR spectrum of **3DPQ-12**.
Figure S176. The ^{13}C NMR spectrum of **3DPQ-12**.
Figure S177. The ^{15}N NMR spectrum of **3DPQ-12**.
Figure S178. The ^{17}O NMR spectrum of **3DPQ-12**.
Figure S179. The HPLC spectrum of **3DPQ-1**
Figure S180. The HPLC spectrum of **3DPQ-2**
Figure S181. The HPLC spectrum of **3DPQ-3**
Figure S182. The HPLC spectrum of **3DPQ-4**
Figure S183. The HPLC spectrum of **3DPQ-5.**

Figure S184. The HPLC spectrum of **3DPQ-6.**
Figure S185. The HPLC spectrum of **3DPQ-7.**
Figure S186. The HPLC spectrum of **3DPQ-8.**
Figure S187. The HPLC spectrum of **3DPQ-9.**
Figure S188. The HPLC spectrum of **3DPQ-10.**
Figure S189. The HPLC spectrum of **3DPQ-11**
Figure S190. The HPLC spectrum of **3DPQ-12.**
Figure S191. The estrogen receptor α antagonism in the presence of **3DPQ-12** (**A**); **3DPQ-3** (**B**); **3DPQ-9** (**C**); **3DPQ-4** (**D**); **3DPQ-2** (**E**); **3DPQ-1** (**F**).
Figure S192. The estrogen receptor α antagonism in the presence of **3DPQ-7** (A); **3DPQ-11** (B); **3DPQ-10** (C); **3DPQ-5** (D); **3DPQ-8** (E); **3DPQ-6** (F).
Figure S193. The estrogen receptor β antagonism in the presence of **3DPQ-12** (A); **3DPQ-3** (B); **3DPQ-9** (C); **3DPQ-4** (D); **3DPQ-2** (E); **3DPQ-1** (F).
Figure S194. The estrogen receptor β antagonism in the presence of **3DPQ-7** (A); **3DPQ-11** (B); **3DPQ-10** (C); **3DPQ-5** (D); **3DPQ-8** (E); **3DPQ-6** (F).
Figure S195. The bioactive conformations of **3DPQ-7** (A); **3DPQ-11** (B); **3DPQ-10** (C); **3DPQ-5** (D); **3DPQ-8** (E); **3DPQ-6** (F) within the ERα active site. Amino acid residues are depicted in white, H12 helix is presented in cornflower blue ribbon.
Figure S196. The antiproliferative activity of **3DPQ-12** (**A**); **3DPQ-3** (**B**); **3DPQ-9** (**C**); **3DPQ-4** (**D**); **3DPQ-2** (**E**); **3DPQ-1** (**F**) against MCF-7 cell line.
Figure S197. The antiproliferative activity of **3DPQ-7** (A); **3DPQ-11** (B); **3DPQ-10** (C); **3DPQ-5** (D); **3DPQ-8** (E); **3DPQ-6** (F) against MCF-7 cell lines.
Figure S198. The antiproliferative activity of **3DPQ-12** (A); **3DPQ-3** (B); **3DPQ-9** (C); **3DPQ-4** (D); **3DPQ-2** (E); **3DPQ-1** (F) against MDA-MB-231 cell lines.
Figure S199. The antiproliferative activity of **3DPQ-7** (A); **3DPQ-11** (B); **3DPQ-10** (C); **3DPQ-5** (D); **3DPQ-8** (E); **3DPQ-6** (F) against MDA-MB-231 cell lines.
Figure S200. The antiproliferative activity of **3DPQ-12** (A); **3DPQ-3** (B); **3DPQ-9** (C); **3DPQ-4** (D); **3DPQ-2** (E); **3DPQ-1** (F) against Ishikawa cell lines.
Figure S201. The antiproliferative activity of **3DPQ-7** (A); **3DPQ-11** (B); **3DPQ-10** (C); **3DPQ-5** (D); **3DPQ-8** (E); **3DPQ-6** (F) against Ishikawa cell lines.
Figure S202. Cell cycle analysis of MCF-7 cell lines treated with **3DPQ-12** in concentration of 0.1 nM (A); $\mathbf{E_2}$ in concentration of 0.1 nM (B); **4-OHT** in concentration of 0.1 nM (C); **Ral** in concentration of 0.1 nM (D); **3DPQ-12** in concentration of 1 nM (E); $\mathbf{E_2}$ in concentration of 1 nM (F); **4-OHT** in concentration of 1 nM (G); **Ral** in concentration of 1 nM.
Figure S203. Cell cycle analysis of MCF-T cell lines treated with **3DPQ-3** in concentration of 0.1 nM (A); $\mathbf{E_2}$ in concentration of 0.1 nM (B); **4-OHT** in concentration of 0.1 nM (C); **Ral** in concentration of 0.1 nM (D); **3DPQ-3** in concentration of 1 nM (E); $\mathbf{E_2}$ in concentration of 1 nM (F); **4-OHT** in concentration of 1 nM (G); **Ral** in concentration of 1 nM.
Figure S204. Cell cycle analysis of MCF-T cell lines treated with **3DPQ-9** in concentration of 0.1 nM (A); $\mathbf{E_2}$ in concentration of 0.1 nM (B); **4-OHT** in concentration of 0.1 nM (C); **Ral** in concentration of 0.1 nM (D); **3DPQ-9** in concentration of 1 nM (E); $\mathbf{E_2}$ in concentration of 1 nM (F); **4-OHT** in concentration of 1 nM (G); **Ral** in concentration of 1 nM.
Figure S205. Cell cycle analysis of MCF-T cell lines treated with **3DPQ-4** in concentration of 0.1 nM (A); $\mathbf{E_2}$ in concentration of 0.1 nM (B); **4-OHT** in concentration of 0.1 nM (C); **Ral** in concentration of 0.1 nM (D); **3DPQ-4** in concentration of 1 nM (E); $\mathbf{E_2}$ in concentration of 1 nM (F); **4-OHT** in concentration of 1 nM (G); **Ral** in concentration of 1 nM.
Figure S206. Cell cycle analysis of MCF-T cell lines treated with **3DPQ-2** in concentration of 0.1 nM (A); $\mathbf{E_2}$ in concentration of 0.1 nM (B); **4-OHT** in concentration of 0.1 nM (C); **Ral** in concentration of 0.1 nM

(D); **3DPQ-2** in concentration of 1 nM (E); **E2** in concentration of 1 nM (F); **4-OHT** in concentration of 1 nM (G); **Ral** in concentration of 1 nM.

Figure S207. Cell cycle analysis of MCF-T cell lines treated with **3DPQ-1** in concentration of 0.1 nM (A); **E2** in concentration of 0.1 nM (B); **4-OHT** in concentration of 0.1 nM (C); **Ral** in concentration of 0.1 nM (D); **3DPQ-1** in concentration of 1 nM (E); **E2** in concentration of 1 nM (F); **4-OHT** in concentration of 1 nM (G); **Ral** in concentration of 1 nM.

Figure S208. Cell cycle analysis of MCF-T cell lines treated with **3DPQ-7** in concentration of 0.1 nM (A); **E2** in concentration of 0.1 nM (B); **4-OHT** in concentration of 0.1 nM (C); **Ral** in concentration of 0.1 nM (D); **3DPQ-7** in concentration of 1 nM (E); **E2** in concentration of 1 nM (F); **4-OHT** in concentration of 1 nM (G); **Ral** in concentration of 1 nM.

Figure S209. Cell cycle analysis of MCF-T cell lines treated with **3DPQ-11** in concentration of 0.1 nM (A); **E2** in concentration of 0.1 nM (B); **4-OHT** in concentration of 0.1 nM (C); **Ral** in concentration of 0.1 nM (D); **3DPQ-11** in concentration of 1 nM (E); **E2** in concentration of 1 nM (F); **4-OHT** in concentration of 1 nM (G); **Ral** in concentration of 1 nM.

Figure S210. Cell cycle analysis of MCF-T cell lines treated with **3DPQ-10** in concentration of 0.1 nM (A); **E2** in concentration of 0.1 nM (B); **4-OHT** in concentration of 0.1 nM (C); **Ral** in concentration of 0.1 nM (D); **3DPQ-10** in concentration of 1 nM (E); **E2** in concentration of 1 nM (F); **4-OHT** in concentration of 1 nM (G); **Ral** in concentration of 1 nM.

Figure S211. Cell cycle analysis of MCF-T cell lines treated with **3DPQ-5** in concentration of 1 nM (A); **E2** in concentration of 0.1 nM (B); **4-OHT** in concentration of 0.1 nM (C); **Ral** in concentration of 0.1 nM (D); **3DPQ-5** in concentration of 10 nM (E); **E2** in concentration of 1 nM (F); **4-OHT** in concentration of 1 nM (G); **Ral** in concentration of 1 nM.

Figure S212. Cell cycle analysis of MCF-T cell lines treated with **3DPQ-8** in concentration of 1 nM (A); **E2** in concentration of 0.1 nM (B); **4-OHT** in concentration of 0.1 nM (C); **Ral** in concentration of 0.1 nM (D); **3DPQ-8** in concentration of 10 nM (E); **E2** in concentration of 1 nM (F); **4-OHT** in concentration of 1 nM (G); **Ral** in concentration of 1 nM.

Figure S213. Cell cycle analysis of MCF-T cell lines treated with **3DPQ-6** in concentration of 1 nM (A); **E2** in concentration of 0.1 nM (B); **4-OHT** in concentration of 0.1 nM (C); **Ral** in concentration of 0.1 nM (D); **3DPQ-6** in concentration of 10 nM (E); **E2** in concentration of 1 nM (F); **4-OHT** in concentration of 1 nM (G); **Ral** in concentration of 1 nM.

Figure S214. Photomicrograph of breast section of normal control rat showing lobuloalveolar unit (LaU) and cuboidal epithelial cells (CE) (A); photomicrograph of breast section treated with **MNU** showing mammary gland carcinoma alongside with massive proliferation of neoplastic epithelial cells (EC) (B); photomicrograph of breast section treated with **3DPQ-3** in concentration of 5 mg/kg of bwt showing lobuloalveolar unit (LaU) and cuboidal epithelial cells (CE) (C); photomicrograph of breast section treated with **3DPQ-3** in concentration of 50 mg/kg of bwt showing lobuloalveolar unit (LaU) and cuboidal epithelial cells (CE) (D); photomicrograph of breast section treated with **4-OHT** in concentration of 5 mg/kg of bwt showing necrosis (NEC) (E); photomicrograph of breast section treated with **4-OHT** in concentration of 50 mg/kg of bwt showing necrosis (NEC) (F); photomicrograph of breast section treated with **Ral** in concentration of 5 mg/kg of bwt showing differentiated extralobular ducts (ED) (G); photomicrograph of breast section treated with **Ral** in concentration of 50 mg/kg of bwt showing differentiated extralobular ducts (ED) (G), shown in x200 magnification and stained with hematoxylin and eosin.

Figure S215. Photomicrograph of breast section of normal control rat showing lobuloalveolar unit (LaU) and cuboidal epithelial cells (CE) (A); photomicrograph of breast section treated with **MNU** showing mammary gland carcinoma alongside with massive proliferation of neoplastic epithelial cells (EC) (B); photomicrograph of breast section treated with **3DPQ-9** in concentration of 5 mg/kg of bwt showing lobuloalveolar unit (LaU) and cuboidal epithelial cells (CE) (C); photomicrograph of breast section treated

with **3DPQ-9** in concentration of 50 mg/kg of bwt showing lobuloalveolar unit (LaU) and cuboidal epithelial cells (CE) (D); photomicrograph of breast section treated with **4-OHT** in concentration of 5 mg/kg of bwt showing necrosis (NEC) (E); photomicrograph of breast section treated with **4-OHT** in concentration of 50 mg/kg of bwt showing necrosis (NEC) (F); photomicrograph of breast section treated with **Ral** in concentration of 5 mg/kg of bwt showing differentiated extralobular ducts (ED) (G); photomicrograph of breast section treated with **Ral** in concentration of 50 mg/kg of bwt showing differentiated extralobular ducts (ED) (G), shown in x200 magnification and stained with hematoxylin and eosin.

Figure S216. Photomicrograph of breast section of normal control rat showing lobuloalveolar unit (LaU) and cuboidal epithelial cells (CE) (A); photomicrograph of breast section treated with **MNU** showing mammary gland carcinoma alongside with massive proliferation of neoplastic epithelial cells (EC) (B); photomicrograph of breast section treated with **3DPQ-4** in concentration of 5 mg/kg of bwt showing lobuloalveolar unit (LaU) and cuboidal epithelial cells (CE) (C); photomicrograph of breast section treated with **3DPQ-4** in concentration of 50 mg/kg of bwt showing lobuloalveolar unit (LaU) and cuboidal epithelial cells (CE) (D); photomicrograph of breast section treated with **4-OHT** in concentration of 5 mg/kg of bwt showing necrosis (NEC) (E); photomicrograph of breast section treated with **4-OHT** in concentration of 50 mg/kg of bwt showing necrosis (NEC) (F); photomicrograph of breast section treated with **Ral** in concentration of 5 mg/kg of bwt showing differentiated extralobular ducts (ED) (G); photomicrograph of breast section treated with **Ral** in concentration of 50 mg/kg of bwt showing differentiated extralobular ducts (ED) (G), shown in x200 magnification and stained with hematoxylin and eosin.

Figure S217. Photomicrograph of breast section of normal control rat showing lobuloalveolar unit (LaU) and cuboidal epithelial cells (CE) (A); photomicrograph of breast section treated with **MNU** showing mammary gland carcinoma alongside with massive proliferation of neoplastic epithelial cells (EC) (B); photomicrograph of breast section treated with **3DPQ-2** in concentration of 5 mg/kg of bwt showing lobuloalveolar unit (LaU) and cuboidal epithelial cells (CE) (C); photomicrograph of breast section treated with **3DPQ-1** in concentration of 50 mg/kg of bwt showing lobuloalveolar unit (LaU) and cuboidal epithelial cells (CE) (D); photomicrograph of breast section treated with **4-OHT** in concentration of 5 mg/kg of bwt showing necrosis (NEC) (E); photomicrograph of breast section treated with **4-OHT** in concentration of 50 mg/kg of bwt showing necrosis (NEC) (F); photomicrograph of breast section treated with **Ral** in concentration of 5 mg/kg of bwt showing differentiated extralobular ducts (ED) (G); photomicrograph of breast section treated with **Ral** in concentration of 50 mg/kg of bwt showing differentiated extralobular ducts (ED) (G), shown in x200 magnification and stained with hematoxylin and eosin.

Figure S218. Photomicrograph of breast section of normal control rat showing lobuloalveolar unit (LaU) and cuboidal epithelial cells (CE) (A); photomicrograph of breast section treated with **MNU** showing mammary gland carcinoma alongside with massive proliferation of neoplastic epithelial cells (EC) (B); photomicrograph of breast section treated with **3DPQ-1** in concentration of 5 mg/kg of bwt showing lobuloalveolar unit (LaU) and cuboidal epithelial cells (CE) (C); photomicrograph of breast section treated with **3DPQ-1** in concentration of 50 mg/kg of bwt showing lobuloalveolar unit (LaU) and cuboidal epithelial cells (CE) (D); photomicrograph of breast section treated with **4-OHT** in concentration of 5 mg/kg of bwt showing necrosis (NEC) (E); photomicrograph of breast section treated with **4-OHT** in concentration of 50 mg/kg of bwt showing necrosis (NEC) (F); photomicrograph of breast section treated with **Ral** in concentration of 5 mg/kg of bwt showing differentiated extralobular ducts (ED) (G); photomicrograph of breast section treated with **Ral** in concentration of 50 mg/kg of bwt showing differentiated extralobular ducts (ED) (G), shown in x200 magnification and stained with hematoxylin and eosin.

Figure S219. Photomicrograph of a lung section of a rat being treated with placebo (2% carboxylmethyl cellulose) showing no morphological changes (A); photomicrograph of a lung section of a rat being treated with saline showing no morphological changes (B); photomicrograph of a lung section of a rat being treated with **MNU** at the maximum tolerated dose of 100 mg/kg bwt showing alveolar bronchiolar single adenoma (C); photomicrograph of a lung section of a rat being treated with **4-OHT** at the maximum feasible dose of 1000 mg/kg showing no morphological changes (D); photomicrograph of a lung section of a rat being treated with **Ral** at the maximum feasible dose of 1000 mg/kg showing no morphological changes (E); photomicrograph of a lung section of a rat being treated with **3DPQ-1** at the maximum feasible dose of 1000 mg/kg showing no morphological changes (F); photomicrograph of a lung section of a rat being treated with **3DPQ-2** at the maximum feasible dose of 1000 mg/kg showing no morphological changes (G); photomicrograph of a lung section of a rat being treated with **3DPQ-3** at the maximum feasible dose of 1000 mg/kg showing no morphological changes (H); photomicrograph of a lung section of a rat being treated with **3DPQ-4** at the maximum feasible dose of 1000 mg/kg showing no morphological changes (I); photomicrograph of a lung section of a rat being treated with **3DPQ-9** at the maximum feasible dose of 1000 mg/kg showing no morphological changes (J); photomicrograph of a lung section of a rat being treated with **3DPQ-12** at the maximum feasible dose of 1000 mg/kg showing no morphological changes (K); shown in x200 magnification and stained with hematoxylin and eosin.

Figure S220. Photomicrograph of a spleen section of a rat being treated with placebo (2% carboxylmethyl cellulose) showing no morphological changes (A); photomicrograph of a spleen section of a rat being treated with saline showing no morphological changes (B); photomicrograph of a spleen lung section of a rat being treated with **MNU** at the maximum tolerated dose of 100 mg/kg bwt showing faded red pulp with numerous numbers of medium to large sized undifferentiated, pleomorphic nuclei (C); photomicrograph of a spleen section of a rat being treated with **4-OHT** at the maximum feasible dose of 1000 mg/kg showing no morphological changes (D); photomicrograph of a spleen section of a rat being treated with **Ral** at the maximum feasible dose of 1000 mg/kg showing no morphological changes (E); photomicrograph of a spleen section of a rat being treated with **3DPQ-1** at the maximum feasible dose of 1000 mg/kg showing no morphological changes (F); photomicrograph of a spleen section of a rat being treated with **3DPQ-2** at the maximum feasible dose of 1000 mg/kg showing no morphological changes (G); photomicrograph of a spleen section of a rat being treated with **3DPQ-3** at the maximum feasible dose of 1000 mg/kg showing no morphological changes (H); photomicrograph of a spleen section of a rat being treated with **3DPQ-4** at the maximum feasible dose of 1000 mg/kg showing no morphological changes (I); photomicrograph of a spleen section of a rat being treated with **3DPQ-9** at the maximum feasible dose of 1000 mg/kg showing no morphological changes (J); photomicrograph of a spleen section of a rat being treated with **3DPQ-12** at the maximum feasible dose of 1000 mg/kg showing no morphological changes (K); shown in x200 magnification and stained with hematoxylin and eosin.

Figure S221. Photomicrograph of a liver section of a rat being treated with placebo (2% carboxylmethyl cellulose) showing no morphological changes (A); photomicrograph of a liver section of a rat being treated with saline showing no morphological changes (B); photomicrograph of a liver section of a rat being treated with **MNU** at the maximum tolerated dose of 100 mg/kg bwt showing metastasis of numerous numbers of medium to large size neoplastic lymphocytes to the liver (C); photomicrograph of a liver section of a rat being treated with **4-OHT** at the maximum feasible dose of 1000 mg/kg showing no morphological changes (D); photomicrograph of a liver section of a rat being treated with **Ral** at the maximum feasible dose of 1000 mg/kg showing no morphological changes (E); photomicrograph of a liver section of a rat being treated with **3DPQ-1** at the maximum feasible dose of 1000 mg/kg showing no morphological changes (F); photomicrograph of a liver section of a rat being treated with **3DPQ-2** at the maximum feasible dose of 1000 mg/kg showing no morphological changes (G); photomicrograph of a liver section of a rat being treated with **3DPQ-3** at the maximum feasible dose

of 1000 mg/kg showing no morphological changes (H); photomicrograph of a liver section of a rat being treated with **3DPQ-4** at the maximum feasible dose of 1000 mg/kg showing no morphological changes (I); photomicrograph of a liver section of a rat being treated with **3DPQ-9** at the maximum feasible dose of 1000 mg/kg showing no morphological changes (J); photomicrograph of a liver section of a rat being treated with **3DPQ-12** at the maximum feasible dose of 1000 mg/kg showing no morphological changes (K); shown in x200 magnification and stained with hematoxylin and eosin.

Figure S222. Photomicrograph of a kidney section of a rat being treated with placebo (2% carboxylmethyl cellulose) showing no morphological changes (A); photomicrograph of a kidney section of a rat being treated with saline showing no morphological changes (B); photomicrograph of a kidney section of a rat being treated with **MNU** at the maximum tolerated dose of 100 mg/kg bwt showing clumps of medium to large size neoplastic lymphocytes infiltrated into the interstitial space of the renal tubules and around the renal artery (C); photomicrograph of a kidney section of a rat being treated with **4-OHT** at the maximum feasible dose of 1000 mg/kg showing no morphological changes (D); photomicrograph of a kidney section of a rat being treated with **Ral** at the maximum feasible dose of 1000 mg/kg showing no morphological changes (E); photomicrograph of a kidney section of a rat being treated with **3DPQ-1** at the maximum feasible dose of 1000 mg/kg showing no morphological changes (F); photomicrograph of a kidney section of a rat being treated with **3DPQ-2** at the maximum feasible dose of 1000 mg/kg showing no morphological changes (G); photomicrograph of a kidney section of a rat being treated with **3DPQ-3** at the maximum feasible dose of 1000 mg/kg showing no morphological changes (H); photomicrograph of a kidney section of a rat being treated with **3DPQ-4** at the maximum feasible dose of 1000 mg/kg showing no morphological changes (I); photomicrograph of a kidney section of a rat being treated with **3DPQ-9** at the maximum feasible dose of 1000 mg/kg showing no morphological changes (J); photomicrograph of a kidney section of a rat being treated with **3DPQ-12** at the maximum feasible dose of 1000 mg/kg showing no morphological changes (K); shown in x200 magnification and stained with hematoxylin and eosin.

Figure S223. Photomicrograph of a heart section of a rat being treated with placebo (2% carboxylmethyl cellulose) showing no morphological changes (A); photomicrograph of a heart section of a rat being treated with saline showing no morphological changes (B); photomicrograph of a heart section of a rat being treated with **MNU** at the maximum tolerated dose of 100 mg/kg bwt showing interstitial hydrops (C); photomicrograph of a heart section of a rat being treated with **4-OHT** at the maximum feasible dose of 1000 mg/kg showing no morphological changes (D); photomicrograph of a heart section of a rat being treated with **Ral** at the maximum feasible dose of 1000 mg/kg showing no morphological changes (E); photomicrograph of a heart section of a rat being treated with **3DPQ-1** at the maximum feasible dose of 1000 mg/kg showing no morphological changes (F); photomicrograph of a heart section of a rat being treated with **3DPQ-2** at the maximum feasible dose of 1000 mg/kg showing no morphological changes (G); photomicrograph of a heart section of a rat being treated with **3DPQ-3** at the maximum feasible dose of 1000 mg/kg showing no morphological changes (H); photomicrograph of a heart section of a rat being treated with **3DPQ-4** at the maximum feasible dose of 1000 mg/kg showing no morphological changes (I); photomicrograph of a heart section of a rat being treated with **3DPQ-9** at the maximum feasible dose of 1000 mg/kg showing no morphological changes (J); photomicrograph of a heart section of a rat being treated with **3DPQ-12** at the maximum feasible dose of 1000 mg/kg showing no morphological changes (K); shown in x200 magnification and stained with hematoxylin and eosin.

Figure S224. Photomicrograph of a colon section of a rat being treated with placebo (2% carboxylmethyl cellulose) showing no morphological changes (A); photomicrograph of a colon section of a rat being treated with saline showing no morphological changes (B); photomicrograph of a colon section of a rat being treated with **MNU** at the maximum tolerated dose of 100 mg/kg bwt showing the induced colon cancer (C); photomicrograph of a colon section of a rat being treated with **4-OHT** at the maximum feasible dose of 1000 mg/kg showing no morphological changes (D); photomicrograph of a colon section

of a rat being treated with **Ral** at the maximum feasible dose of 1000 mg/kg showing no morphological changes (E); photomicrograph of a colon section of a rat being treated with **3DPQ-1** at the maximum feasible dose of 1000 mg/kg showing no morphological changes (F); photomicrograph of a colon section of a rat being treated with **3DPQ-2** at the maximum feasible dose of 1000 mg/kg showing no morphological changes (G); photomicrograph of a colon section of a rat being treated with **3DPQ-3** at the maximum feasible dose of 1000 mg/kg showing no morphological changes (H); photomicrograph of a heart section of a rat being treated with **3DPQ-4** at the maximum feasible dose of 1000 mg/kg showing no morphological changes (I); photomicrograph of a colon section of a rat being treated with **3DPQ-9** at the maximum feasible dose of 1000 mg/kg showing no morphological changes (J); photomicrograph of a colon section of a rat being treated with **3DPQ-12** at the maximum feasible dose of 1000 mg/kg showing no morphological changes (K); shown in x200 magnification and stained with hematoxylin and eosin.

Experimental

Crystal Structures Compilation and Preparation
Pharmacophore Modeling.
3-D QSAR Modeling.
Alignment Assessment Rules.
Ligand's Experimental Conformations Randomizations
Glide Settings.
Flexible Ligand Alignment Tool Settings.
Generation Of Modeled And Designed Compounds.
Test Sets Alignment.
Virtual Screening
Admetox Predictions
Chemistry (the equipment)
Chemistry (commercial compounds supply).
Synthetic procedures
*Synthesis of 5-((tert-butyldimethylsilyl)oxy)pentanal (**R1**)*
*Synthesis of 5-methoxy-2,2,3,3,11,11,12,12-octamethyl-4,10-dioxa-3,11-disilatridecane (**R2**)*
*Synthesis of 1-methoxypentane-1,5-diol (**R3**)*
*Synthesis of 5-((5-((tert-butyldimethylsilyl)oxy)-5-methoxypentyl)thio)-1-phenyl-1H-tetrazole (**R4**)*
*Synthesis of 5-((5-((tert-butyldimethylsilyl)oxy)-5-methoxypentyl)sulfonyl)-1-phenyl-1H-tetrazole (**R5**)*
*Synthesis of (R,E)-methyl 5-((1R,2R,4R)-2-formyl-4-((2-methoxyethoxy)methoxy)cyclopentyl)-5-((2-methoxyethoxy)methoxy)pent-3-enoate (**R6**)*
*(4R,E)-methyl 4-((1R,2S,4S)-2-((E)-6-((tert-butyldimethylsilyl)oxy)-6-methoxyhex-1-en-1-yl)-4-((2-methoxyethoxy)methoxy)cyclopentyl)-4-((2-methoxyethoxy)methoxy)but-2-enoate (**R7**)*
*Synthesis of (4R,E)-methyl 4-((1R,2S,4S)-2-((E)-6-hydroxy-6-methoxyhex-1-en-1-yl)-4-((2-methoxyethoxy)methoxy)cyclopentyl)-4-((2-methoxyethoxy)methoxy)but-2-enoate (**R8**)*
*Synthesis of (1R,2E,6R,10E,11aS,13S,14aR)-6-methoxy-1,13-bis((2-methoxyethoxy)methoxy)-6,7,8,9,12,13,14,14a-octahydro-1H-cyclopenta[f][1]oxacyclotridecin-4(11aH)-one (**BFA-D1**)*
*Synthesis of (1R,2E,6R,10E,11aS,13S,14aR)-1,13-dihydroxy-6-methoxy-6,7,8,9,12,13,14,14a-octahydro-1H-cyclopenta[f][1]oxacyclotridecin-4(11aH)-one (**BFA-D2**)*
*Synthesis of (1R,2E,6R,10E,11aS,13S,14aR)-13-((tert-butyldimethylsilyl)oxy)-1-hydroxy-6-methoxy-6,7,8,9,12,13,14,14a-octahydro-1H-cyclopenta[f][1]oxacyclotridecin-4(11aH)-one (**BFA-D3**)*
*Synthesis of (1R,2E,6R,10E,11aS,13S,14aR)-13-((tert-butyldimethylsilyl)oxy)-6-methoxy-4-oxo-4,6,7,8,9,11a,12,13,14,14a-decahydro-1H-cyclopenta[f][1]oxacyclotridecin-1-yl 3-acetyl-4-hydroxybenzoate (**BFA-D4**)*
*General procedure for the synthesis of **Pro-R3**, **Pro-R6**, **Pro-R7**, **Pro-R8**, **Pro-R9**, and **Pro-R12***

*1-(2-chloroethyl)-1,2,5,6-tetrahydropyridine-3-carboxylic acid (**Pro-R3***

*6-(2-chloroethyl)-6,7-dihydro-5H-pyrrolo[3,4-b]pyridine (**Pro-R6**).*

*2-(2-chloroethyl)-1,2-dihydroisoquinoline (**Pro-R7**).*

*1-(2-chloroethyl)decahydro-1,8-naphthyridine (**Pro-R8**).*

*1-(2-chloroethyl)-6-oxo-1,2,5,6-tetrahydropyridine-3-carboxylic acid (**Pro-R9**).*

*1-(2-chloroethyl)-3-hydroxypiperidin-2-one (**Pro-R12**).*

*General procedure for the synthesis of **Pro-3DPQ-1** to **Pro-3DPQ-12***

*(1R,2E,6R,10E,11aS,13S,14aR)-13-((tert-butyldimethylsilyl)oxy)-6-methoxy-4-oxo-4,6,7,8,9,11a,12,13,14,14a-decahydro-1H-cyclopenta[f][1]oxacyclotridecin-1-yl 3-acetyl-4-(2-(methylamino)ethoxy)benzoate (**Pro-3DPQ-1**).*

(1R,2E,6R,10E,11aS,13S,14aR)-13-((tert-butyldimethylsilyl)oxy)-6-methoxy-4-oxo-4,6,7,8,9,11a,12,13,14,14a-decahydro-1H-cyclopenta[f][1]oxacyclotridecin-1-yl 3-acetyl-4-(2-(dimethylamino)ethoxy)benzoate (**Pro-3DPQ-2**).

1-(2-(2-acetyl-4-((((1R,2E,6R,10E,11aS,13S,14aR)-13-((tert-butyldimethylsilyl)oxy)-6-methoxy-4-oxo-4,6,7,8,9,11a,12,13,14,14a-decahydro-1H-cyclopenta[f][1]oxacyclotridecin-1-yl)oxy)carbonyl)phenoxy)ethyl)-1,2,5,6-tetrahydropyridine-3-carboxylic acid (**Pro-3DPQ-3**).

*2-(2-acetyl-4-((((1R,2E,6R,10E,11aS,13S,14aR)-13-((tert-butyldimethylsilyl)oxy)-6-methoxy-4-oxo-4,6,7,8,9,11a,12,13,14,14a-decahydro-1H-cyclopenta[f][1]oxacyclotridecin-1-yl)oxy)carbonyl)phenoxy)ethanesulfonic acid (**Pro-3DPQ-4**).*

*(1R,2E,6R,10E,11aS,13S,14aR)-13-((tert-butyldimethylsilyl)oxy)-6-methoxy-4-oxo-4,6,7,8,9,11a,12,13,14,14a-decahydro-1H-cyclopenta[f][1]oxacyclotridecin-1-yl 3-acetyl-4-(2-(2,5-dioxopyrrolidin-1-yl)ethoxy)benzoate (**Pro-3DPQ-5**).*

*(1R,2E,6R,10E,11aS,13S,14aR)-13-((tert-butyldimethylsilyl)oxy)-6-methoxy-4-oxo-4,6,7,8,9,11a,12,13,14,14a-decahydro-1H-cyclopenta[f][1]oxacyclotridecin-1-yl 4-(2-(5H-pyrrolo[3,4-b]pyridin-6(7H)-yl)ethoxy)-3-acetylbenzoate (**Pro-3DPQ-6**).*

*(2E,10E,11aS,14aR)-13-((tert-butyldimethylsilyl)oxy)-6-methoxy-4-oxo-4,6,7,8,9,11a,12,13,14,14a-decahydro-1H-cyclopenta[f][1]oxacyclotridecin-1-yl 4-(2-(isoquinolin-2(1H)-yl)ethoxy)-3-methylbenzoate (**Pro-3DPQ-7**).*

*(1R,2E,6R,10E,11aS,13S,14aR)-13-((tert-butyldimethylsilyl)oxy)-6-methoxy-4-oxo-4,6,7,8,9,11a,12,13,14,14a-decahydro-1H-cyclopenta[f][1]oxacyclotridecin-1-yl 3-acetyl-4-(2-(octahydro-1,8-naphthyridin-1(2H)-yl)ethoxy)benzoate (**Pro-3DPQ-8**).*

*1-(2-(2-acetyl-4-((((1R,2E,6R,10E,11aS,13S,14aR)-13-((tert-butyldimethylsilyl)oxy)-6-methoxy-4-oxo-4,6,7,8,9,11a,12,13,14,14a-decahydro-1H-cyclopenta[f][1]oxacyclotridecin-1-yl)oxy)carbonyl)phenoxy)ethyl)-6-oxo-1,2,5,6-tetrahydropyridine-3-carboxylic acid (**Pro-3DPQ-9**).*

*(1R,2E,6R,10E,11aS,13S,14aR)-13-((tert-butyldimethylsilyl)oxy)-6-methoxy-4-oxo-4,6,7,8,9,11a,12,13,14,14a-decahydro-1H-cyclopenta[f][1]oxacyclotridecin-1-yl 3-acetyl-4-(2-(piperidin-1-yl)ethoxy)benzoate (**Pro-3DPQ-10**).*

*(1R,2E,6R,10E,11aS,13S,14aR)-13-((tert-butyldimethylsilyl)oxy)-6-methoxy-4-oxo-4,6,7,8,9,11a,12,13,14,14a-decahydro-1H-cyclopenta[f][1]oxacyclotridecin-1-yl 3-acetyl-4-(2-(pyrrolidin-1-yl)ethoxy)benzoate (**Pro-3DPQ-11**).*

(1R,2E,6R,10E,11aS,13S,14aR)-13-((tert-butyldimethylsilyl)oxy)-6-methoxy-4-oxo-4,6,7,8,9,11a,12,13,14,14a-decahydro-1H-cyclopenta[f][1]oxacyclotridecin-1-yl 3-acetyl-4-(2-(3-hydroxy-2-oxopiperidin-1-yl)ethoxy)benzoate (**Pro-3DPQ-12**).

*General procedure for the synthesis of **3DPQ-1** to **3DPQ-12***

(1R,2E,6R,10E,11aS,13S,14aR)-6,13-dihydroxy-4-oxo-4,6,7,8,9,11a,12,13,14,14a-decahydro-1H-cyclopenta[f][1]oxacyclotridecin-1-yl 3-acetyl-4-(2-(methylamino)ethoxy)benzoate (**3DPQ-1**).

(1R,2E,6R,10E,11aS,13S,14aR)-6,13-dihydroxy-4-oxo-4,6,7,8,9,11a,12,13,14,14a-decahydro-1H-cyclopenta[f][1]oxacyclotridecin-1-yl 3-acetyl-4-(2-(dimethylamino)ethoxy)benzoate (**3DPQ-2**).

1-(2-(2-acetyl-4-((((2E,6R,10E,11aS,13S,14aR)-6,13-dihydroxy-4-oxo-4,6,7,8,9,11a,12,13,14,14a-decahydro-1H-cyclopenta[f][1]oxacyclotridecin-1-yl)oxy)carbonyl)phenoxy)ethyl)-1,2,5,6-tetrahydropyridine-3-carboxylic acid (**3DPQ-3**).

2-(2-acetyl-4-((((1R,2E,6R,10E,11aS,13S,14aR)-6,13-dihydroxy-4-oxo-4,6,7,8,9,11a,12,13,14,14a-decahydro-1H-cyclopenta[f][1]oxacyclotridecin-1-yl)oxy)carbonyl)phenoxy)ethanesulfonic acid (**3DPQ-4**).

-decahydro-1H-cyclopenta[f][1]oxacyclotridecin-1-yl 3-acetyl-4-(2-(2,5-dioxopyrrolidin-1-yl)ethoxy)benzoate (**3DPQ-5**

(1R,2E,6R,10E,11aS,13S,14aR)-6,13-dihydroxy-4-oxo-4,6,7,8,9,11a,12,13,14,14a-decahydro-1H-cyclopenta[f][1]oxacyclotridecin-1-yl 4-(2-(5H-pyrrolo[3,4-b]pyridin-6(7H)-yl)ethoxy)-3-acetylbenzoate (**3DPQ-6**).

(1R,2E,6R,10E,11aS,13S,14aR)-6,13-dihydroxy-4-oxo-4,6,7,8,9,11a,12,13,14,14a-decahydro-1H-cyclopenta[f][1]oxacyclotridecin-1-yl 3-acetyl-4-(2-(isoquinolin-2(1H)-yl)ethoxy)benzoate (**3DPQ-7**).

(1R,2E,6R,10E,11aS,13S,14aR)-6,13-dihydroxy-4-oxo-4,6,7,8,9,11a,12,13,14,14a-decahydro-1H-cyclopenta[f][1]oxacyclotridecin-1-yl 3-acetyl-4-(2-(octahydro-1,8-naphthyridin-1(2H)-yl)ethoxy)benzoate (**3DPQ-8**).

1-(2-(2-acetyl-4-((((1R,2E,6R,10E,11aS,13S,14aR)-6,13-dihydroxy-4-oxo-4,6,7,8,9,11a,12,13,14,14a-decahydro-1H-cyclopenta[f][1]oxacyclotridecin-1-yl)oxy)carbonyl)phenoxy)ethyl)-6-oxo-1,2,5,6-tetrahydropyridine-3-carboxylic acid (**3DPQ-9**).

(1R,2E,6R,10E,11aS,13S,14aR)-6,13-dihydroxy-4-oxo-4,6,7,8,9,11a,12,13,14,14a-decahydro-1H-cyclopenta[f][1]oxacyclotridecin-1-yl 3-acetyl-4-(2-(piperidin-1-yl)ethoxy)benzoate (**3DPQ-10**).

(1R,2E,6R,10E,11aS,13S,14aR)-6,13-dihydroxy-4-oxo-4,6,7,8,9,11a,12,13,14,14a-decahydro-1H-cyclopenta[f][1]oxacyclotridecin-1-yl 3-acetyl-4-(2-(pyrrolidin-1-yl)ethoxy)benzoate (**3DPQ-11**).

(1R,2E,6R,10E,11aS,13S,14aR)-6,13-dihydroxy-4-oxo-4,6,7,8,9,11a,12,13,14,14a-decahydro-1H-cyclopenta[f][1]oxacyclotridecin-1-yl 3-acetyl-4-(2-(3-hydroxy-2-oxopiperidin-1-yl)ethoxy)benzoate (**3DPQ-12**).

Synthesized Compounds Antagonistic Potency and Relative Binding Affinities to ERα And ERβ [128,129]
Synthesized Compounds Antiproliferative Activity against ERα(+)- and ERα(-)-Dependent Breast Cancer Cell Lines [130]
ERα Down-Regulation [15,74,131]
ERα Functional Antagonism Cell Assay [15,74,131]
The Impact of Targeted ERα Antagonists on the MCF-7 Cells Signaling [132-135]
Antibodies, Drugs, and Western Analysis.
qPCR.
ChIP, qChIP, and sequential ChIP.
Effects of Synthesized Compounds on Cytotoxicity and Cell Cycle Distribution in ERα(+)Dependent Breast Cancer Cell Lines [130]
Determination of Lipophilicity [75,156]
In Vivo Anticancer Screening [146]
Measurement of Serum Biochemical Markers [159]
Determination of Antioxidant Markers in Liver Homogenate [159]
Plasma Protein Binding Determination [157]
Determination of the Intrinsic Clearance of Hepatocytes [158]
Preparation of Rat Hepatocytes.
Determination of Metabolic CL_{int} Using Rat Hepatocytes.
Pharmacokinetics Studies In Rats [158]
Histopathological Studies [159]
Maximum-Tolerated-Dose (MTD) Study [160]

Introduction

Genomic Classical Pathway [6-8]

The classical (*i.e.* the genomic direct) pathway occurs upon the E_2 binding to LBD and E_2-ERα post-translatorial phosphorylation of D region Ser294, catalyzed by p38 MAPK. The phosphorylation triggers the involvement of Ser294 in the pathway, a regulator of cancer mitogenesis, carcinogenesis, and metastasis. The D region further supports the $^{P(Ser294)}$-E_2-ERα dimerization (activation) which occurs as a consequence of 'D box' interfaces interaction. The $^{P(Ser294)}$-E_2-ERα-ERα-E_2-$^{P(Ser294)}$ complex is then *via* the DBD bound directly to *h*ERE. The $^{P(Ser294)}$-E_2-ERα-ERα-E_2-$^{P(Ser294)}$-*h*ERE merging is facilitated by HMG proteins (HMG1 and HMG2) involved in chromatin remodeling and DNA denudation. In particular aggregation, the 'P box' sub-region of the CI finger interacts with the AGGTCA sequence, while the CII finger recognizes the remaining *h*ERE half-site. In such a manner activated, *h*ERE serves as a promoter for the transcription. Following, the activated promoter sequence recruits RNA polymerase II (RNAP II or Pol II) to start gene expression and consequent breast cancer development.

Genomic Classical Pathway [6-8]

The genomic indirect (*i.e.* the tethered) pathway regulates the *h*ERE-driven transcription of breast cancer developing genes a process that is yet not fully understood. The tethered pathway is initiated upon the post-translational phosphorylation of AF-1 domain's Ser104, Ser106, Ser118, and Tyr537 catalyzed either by p38 MAPK or transcription factor TFIIH-associated kinase CDk7. Peculiar modification (*i.e.* the E_2-ERα complex activation) is mandatory for the subsequent awakening of DBD domain, ERα dimerization and binding to *h*ERE [6-8]. The $^{P(Ser118)}$-E_2-ERα dimer is a major mediator for interactions between the transcription factors and the receptor, as it interconnects with activator/co-activator or repressor/co-repressor assemblies [6-8]. Hence, when $^{P(Ser118)}$-E_2-ERα dimer is tethered to the *h*ERE, it leads to gene transcription of target genes, regulated by the synergistic activity of AF1 and AF2 [6-8].

The actual *h*ERE-driven transcription of breast cancer starts with the involvement of either AP-1 or GC-rich/CCAAT/TATA responsive elements as promoters (see *Tethered pathway alternative routes* section) [9]. Still, the transcription is rather regulated by the AP-1 responsive elements as promoters [10]. In a few details, the $^{P(Ser118)}$-E_2-ERα dimer-initiated transcriptional cascade is positively regulated upon the interaction of an AP-1 protein (*i.e.* the c-Fos-c-Jun protooncogenes heterodimer) with the AP-1 responsive element with the consensus sequence 5′-TGAG/CTCA-3′[5]. Being involved, AP-1 activates the transcription. Following, the AF-1 contributes to the initiated transcriptional cascade by accepting the direct co-activators, such as CBP or p300 (affiliated with the HAT superfamily), or indirect co-activators, like CARM1 or PRMT1 (originating from HMT superfamily). Consequently, the complementary binding of AP-1 activator and AF-1 co-activators occurs to complete the DBD activation (the $^{P(Ser118)}$-E_2-ERα dimer is fully tethered to the *h*ERE). The LBD is thereupon united with either SRC-1, SRC-2, and/or SRC-3, belonging to the p160/SRC family, acting as the secondary ERα HAT superfamily co-activators. Moreover, the LBD co-activators cooperation is facilitated with the ability of H12 helix to attract the co-activator to the LBD surface, all as a consequence of agonist-forced LBD domain induced-fit spatial arrangement (the H12 glued to the remaining of the LBD, see the *3-D QSAR models interpretation* section of further references). The co-activators must possess the LXXLL motif within the NR region, a hydrophobic docking module that interferes with the NR box-like LLEML sequence of H12 (the cross-section compiled of Leu536, Leu539, Glu542, Met530, and Leu540). Being glued, the co-activators unwind the AP-1 responsive element to facilitate the recruitment of transcription factors (TF), within the transcriptional basal machinery (TBM) complex [11]. Within the TBM, TFIID is bound to *h*ERE (*i.e.* 5′-GGTCA(n)$_{3\text{-}5}$TGACC-3′), after which TFIIA and TFIIB facilitate the formation of a complex between the

RNA polymerase II (RNAP II or Pol II) and TFIIF. Finally, the TBM complex is completed upon the involvement of TFIIE and TFIIH; gene expression and consequent breast cancer development may start.

Tethered Pathway Alternative Routes [6-8]

The alternative route of the tethered pathway, including both the allowance and the suppression of the gene transcription is the one in which the activators/repressors from SP1-like/KLF family initiate the mobilization of GC-rich responsive elements as promoters. Hence, the SP1 activator (dominantly expressed among the SP1, SP4, SP6, KLF1, KLF2, KLF5, and KLF7 activators, respectively) may form the protein-protein complex with the DBD (a direct formation of ternary ERα/SP-DNA complex not yet confirmed). Particular complex may be further enlarged with either CRSP3, CBP/p300, or TAFII30 as direct co-activators. Further on, the SCR-3 co-activator outlines the GC-rich ERE promoter sequence (*i.e.* the consensus SP1 binding site, the 5'-(G/T)GGGCGG(G/A)(G/A)(C/T)-3' sequence), distant from the *h*ERE, which becomes a target for the SP1 activator Cys2/His2 zinc fingers. What is characteristic for the distinct route is that the GC-rich ERE promoter cooperatively interacts with the half site *h*ERE sequence (*h*ERE$_{1/2,}$ the 5'-AGGTCA segment) to activate the expression of genes facilitated by RNAP II. The $^{P(Ser118)-}$E$_2$-ERα-SP-1 complex enhances binding of another activator, NF-YA which, co-activated with cAMP/PKA, this time interacts with the NF-YA promoter (*i.e.* the CCAAT box, a pattern of nucleotides with GGCCAATCT consensus sequence).

Nevertheless, the tethered pathway may be negatively regulated to suppress the $^{P(Ser118)-}$E$_2$-ERα transcriptional activity. Thus, some members of Sp1-the like/KLF family (KLF3, KLF8, and KLF12) bound to the BD domain act as repressors. Their interference with DBD is the pre-condition for the binding of class I co-repressors (NCoR or SMRT) possessing the HDAC function, which are aggregated to Lthe LEML sequence of the LBD *via* the CoRNR (co-repressor–nuclear-receptor) box (*i.e.* the LXX I/H IXXX I/L motif). Following, the class II co-repressors, such as SHP, RIP140 or DAX-1, are merged to the LBD throughout the LXXLL and associated with HDACs. As a consequence, the co-repressors binding influences co-activators blocking; the chromatin relaxation is prevented by HDACs and the RNA polymerase II ability to express genes is alleviated.

Non-Genomic Pathways [6-8]

In a third mechanism, also called non-genomic with rapid effects, it appears that the LBD is enough to mediate actions of estrogens: actions that are too rapid to be accounted for by the activation of RNA and protein synthesis. The non-genomic actions of **E$_2$** include the mobilization of intracellular calcium, and the stimulation of adenylate cyclase activity and cAMP production. Hence, E$_2$ activates a receptor, located in plthe asma membrane. The ERα at the plasma membrane associates with the scaffold protein caveolin-1 and at the same time activates endothelial nitric oxide synthase (eNOS) through protein kinase-mediated phosphorylation. After this rather unclear event, signaling cascades are initiated *via* the second messengers (SM), *i.e.* the proximal signaling molecules of which the Src kinase is a crucial one; the interaction of E$_2$-ERα with Src kinase leads to the increased Src kinase activity and consequent activation of the MAPK signaling pathway (*i.e.* the signal transduction) which regulates the breast cancer development.

Abbreviations

p38 MAPK, p38 mitogen-activated protein kinase; SHC-1, SHC-transforming protein 1; HMG1, High mobility group 1 protein; HMG2, High mobility group 1 protein; CDk7, cyclin-dependent kinase 7; TF, transcriptional factor; TFIIA, transcriptional factor II A; TFIIB, transcriptional factor II B; TFIID, transcriptional factor II D; TFIIF, transcriptional factor II F; TFIIH, transcriptional factor II H; TFII30, transcriptional factor II 30; AP-1, activator protein 1; CBP, CREB-binding protein or CREBBP; p300 (EP300 or E1A binding protein p300); HAT, histone acetyltransferase; CARM1, co-activator-associated arginine

methyltransferase 1; PRMT1, protein arginine methyltransferase 1; HMT, histone methyltransferase; SRC-1, steroid receptor co-activator-1; SRC-2, steroid receptor co-activator-2; SRC-3, steroid receptor co-activator-3; p160/SRC, steroid receptor coactivator family; CtBP2, C-terminal-binding protein 2; mSin3A, multiple SIN3 Transcription Regulator Family Member A complex; CtBPI, carboxyl terminal binding protein I; MTAIs, metastasis-associated proteins; MRF-1, mitochondrial Peptide chain release factor 1; NcoR, Nuclear receptor co-repressor 1; SMRT, silencing mediator of retinoic acid and thyroid hormone receptor; SPs, specificity proteins; KLF, Krüppel-like family of transcription factors; SP1, specificity protein 1; SP4, specificity protein 4; SP6, specificity protein 6; KLF1, Krüppel-like factor 1, KLF2, Krüppel-like factor 2; KLF3, Krüppel-like factor 3; KLF5, Krüppel-like factor 5; KLF7, Krüppel-like factor 7; KLF8, Krüppel-like factor 8; KLF12, Krüppel-like factor 12; CRSP3, Mediator of RNA polymerase II transcription subunit 23 (MED23); Nuclear transcription factor Y subunit alpha; NcoR, Nuclear receptor co-repressor 1; SMRT, silencing mediator of retinoic acid and thyroid hormone receptor; HDAC, histone deacetyltransferase; Src, proto-oncogene tyrosine-protein kinase.

ERα 3-D Pharmacophore Models Generation Overview.

Thus, the seminal paper of Anstead *et al*. reviewed the pharmacophore of $\mathbf{E_2}$ on either ligand-based (LB) or structure-based level (SB) [22]. Within, the $\mathbf{E_2}$'s A-ring hydroxyl group was denoted as a hydrogen-bond donator (HBD), whereas the D-ring 17β-OH group was specified as a hydrogen-bond acceptor (HBA). Further LB 3-D Pharmacophore models (3DP models) were built form naturally occurring compounds. Thus, with the aid of steroidal sapogenins, alkaloids, flavonoids, and galactomannans from Fenugreek (*Trigonella foenum-graceum*) the model was generated with LigandScout [23], resulting in the pharmacophore complied of three features, namely Hydrophobic Interactions (HI), Hydrogen Bond Acceptor (HBA), and Hydrogen Bond Donor (HBD), well superimposed to $\mathbf{E_2}$ [24].

One of the pioneering work in generating the 3DP models against ERα was the analysis of 31 literature available compounds with the associated RBAs, which led to the generation of rapid pharmacophore fingerprinting (PharmPrint method) [25]: the three-point hypothesis aligned to $\mathbf{E_2}$ and diethylstilbestrol, confirmed both the A-ring hydroxyl group and the D-ring 17β-OH group as HBD and HBA, respectively, while the A-ring itself was recognized as an aromatic feature. On the other hand, the bridged bicyclic-1,1-diarylethylene derivatives were subjected to Accelrys Discovery Studio's [26] Catalyst [27] to derive a hypothesis externally evaluated against **Ral**: compound's first phenolic portion and the central phenyl ring were characterized as ring aromatic (r) features, the second phenolic portion was characterized as hydrophobic (p) feature, while the ethanolamine's oxygen was assigned as HB acceptor (a) [28]. The following model was built using (**Ral-**based) arylbenzothiophene derivatives only, also developed by means of Accelrys Discovery Studio's [26] Catalyst [27], this time verified the first phenolic portion as HB donor (d), the second benzene ring as hydrophobic (p), the carbonyl linker between the main core and the central benzene ring as an HB acceptor-lipid (al), and the nitrogen-bearing heterocycle as the second hydrophobic (p) [29]. An Accelrys Discovery Studio's [26] Catalyst [27]-based model was also derived from a group of 35 estrogen ligands was compiled of two HBA features, one hydrophobic (p), and one ring aromatic (r) feature [30].

The literature available SERMs were used to generate the LB Catalyst-based hypothesis, discussed against **Ral** as a training set representative: hydrogen-bond acceptor (HBA) and hydrophobic aromatic (HY1) features were matched with compound's first phenolic portion, hydrogen-bond donor (HBD), and hydrophobic (HY2) were matched with compound's second phenolic portion, whereas the second HY1 was reserved for the remaining phenyl ring [31]. Interestingly, no pharmacophoric feature explained the contribution of the piperidine ring. The obtained 3DP model was externally validated on tetrahydrofuran metabolites with an acetogenic scaffold isolated from *Laurencia glandulifera*. Following, the 74 ERa selective ligands were also collected from the literature and were used to make the Accelrys Discovery Studio's [26] Catalyst [26]-based LB hypothesys [32] externally evaluated against genistein co-

crystalized in **1X7R** complex: the interactions of genistein's phenolic portion facing the Glu353 and Arg394 were recognized by means of the hydrogen-bond acceptor (HA) feature, the interactions with His524 were characterized by hydrogen-bond donor (HB) feature, while two of three generated hydrophobic (HY) features covered the main core.

A significant contribution in understanding the LB 3-D Pharmacophore of literature-available SERMs, when **Ral** potency was explained using the PHASE [88,89]-generated hypothesis [33]: the hydrogen-bond acceptor features (D) overlapped with the first phenolic portion and carbonyl group, hydrogen-bond acceptor feature (D) overlapped with the second phenolic portion, and two aromatic groups (R) features within the main core. The AARRD was used to search the Asinex 3D gold collection chemical database (Asinex Ltd., 5 Gabrichevskogo, St. Building 8, Moscow 125367, Russia).

The further LB 3DP models were made from the series of literature-available compounds, highlighting the 4-hydroxytamoxifen (**4-OHT**) as the most potent training set compound [34]. Hence, the best hypothesis, made by Accelrys Discovery Studio's [26] Catalyst [27] was compiled of and one hydrogen-bond donor (HBD), placed onto the compound's phenolic hydroxyl group, one aromatic ring (RA), aligned onto compound's phenolic benzene ring, one hydrogen-bond acceptor (HBA) feature, positioned onto the ethanolamine bridge oxygen atom, and one hydrophobic moiety (Hy) superimposed to the positively charged nitrogen. The hypothesis was externally evaluated by means of the fevicordin-A from *Phaleria macrocarpa* (Scheff) Boerl. seeds.

Moreover, literature-available ERα binders were analyzed by the Accelrys Discovery Studio's [26] Catalyst [27] to yield a hypothesis compiled of two hydrogen bond acceptors (HBA) and two hydrophobic (HP) features HBA [35], externally validated by performing the VS of Available Chemical Directory database.

The SERMs obtained from ChEMBL database with the pIC_{50} values ranging from 5.0 to 9.7 were analyzed by means of PHASE [88,89] to yield an LB hypothesis elaborated on **4-OHT**: the hydrogen bond acceptor (A) was aligned on the compound's phenolic portion, the positively charged group (P) was placed onto nitrogen atom, while three aromatic rings (R) features were as expected superimposed onto benzene rings [36]. The APRRR hypothesis was used to VS the ZINK database.

Further LB 3DP models were built from a series of 7-thiabicyclo[2.2.1]hept-2-ene-7-oxides, using Accelrys Discovery Studio's [26] Catalyst [27], in the form of HBD-Hyd-Hyd-Hyd-HBD [37]. Another LB contribution was given by generating the 3DP hypothesys [38] from polybrominated diphenyl ethers, by means of the Accelrys Discovery Studio's [26] Catalyst [27], compiled of two hydrophobic groups (H), two hydrophobic aromatic rings (HA), and one hydrogen-bond acceptor (HBA) and describing compound's estrogenic potency.

The biggest progress so far in terms of deriving the 3DP model [39] from co-crystallized ERα binders came with the utilization of ligands found in **1ERR** [13], **1SJ0** [61], **1XP1** [64], **2IOG** [68], **3ERT** [69], **5FQP** [40], and **6B0F** [41], where the LB hypothesis was generated with the aid of Accelrys Discovery Studio's [26] Catalyst [27]. Still, a very poor hypothesis was obtained with only three features hydrogen-bond acceptor (HBA), hydrophobic center 1 (H1), and hydrophobic center 2 (H2), matched for instance with the phenolic portions and benzene rings of Tamoxifen (**Tam**). The hypothesis was used for VS against the ZINK Traditional Chinese Medicine database. Another LB 3DP model was derived from α-mangostin derivatives using LigandScout [23] comprising one HBD, one HBA, and one HI feature [42].

Recently, an LB 3DP model [43], emerging from 34 ERα binders obtained from PubChem database, was derived using LigandScout [23] and was matched in an SB fashion against the ligand co-crystallized in **1SJ0** complex [24]: two hydrogen bond donor (HBD) features were co-aligned with the **1SJ0** first phenolic portion describing its interaction with H3 Glu353 and H3 Lee387, one HBA and HBD feature were placed onto compound's second phenol portion describing the interactions with H11 His524 and H11 Gly521, respectively, while the remaining three hydrophobic (aromatic, Ar) features were

superimposed to the aromatic rings. The hypothesis was used to VS the asymmetrical hexahydro-2*H*-indazole analogs of curcumin (AIACs),

On the other hand, one of the first tentative to create the structure-based (SB) pharmacophore for ERα was the one [44] using agonists co-crystallized in **1QLU** [45], **3ERD** [69], and **2P15** [46] complexes, built with the aid of Pharmacophore Query Editor in MOE [47]. Hence, the hypothesis was slightly revised until that point common representation of LB pharmacophore for **E$_2$** **[**22,24] as it emphasized: (i) the presence of an aromatic ring Ar (aromatic ring preferred to non-aromatic ring); (ii) the presence of groups able to perform HB interactions (OH groups) at each end of the molecule (A-ring hydroxyl group, elsewhere LB-denoted as HBD, more crucial for binding than the D-ring 17β-OH, elsewhere LB-portrayed as HBA); (iii) a precise distance (11 Å) between the hydroxyl oxygen atoms at the 3rd and 17th positions; (iv) a hydrophobic rigid backbone in terms that both A-ring hydroxyl group and D-ring 17β-OH were specified as mixed HBD/HBA features.

The structure-based (SB) 3DP model [48] has been also obtained by means of LigandScout [23] from ligands co-crystallized with mutated ERα, namely **1UOM** [34], **2JFA** [49], and **4XI3** [50], merged with the wild-type **1R5K** [59] complex. The most important application of the model was the characterization of the phenolic portion of **1UOM**, **Ral** (**2JFA**), and **4XI3** facing the Glu353 and H6 Arg394 as both HBD (for H3 Glu353), and HBA (for H6 Arg394), respectively, and the labeling of the phenolic portion facing the H11 His524 as likely HBA, although not confirmed by the corresponding feature. As expected, the aromatic rings were adequately recognized, while the heterocyclic positively charged nitrogen was stressed up as an HBD. The hypothesis was used to VS the ZINK database.

The **4-OHT**, co-crystallized in **3ERT** [32] complex, by means of LigandScout [23] derived a hypothesis comprised of positive ionizable (PI), hydrophobic (HI), hydrogen bond donor (HBD), and acceptor (HBA) features [38]. The model specified the phenolic portion interacting with H3 Glu353 and H6 Arg394 as both HBD (for H3 Glu353), and HBA (for H6 Arg394). The aromatic moieties of **3ERT** were explained as HI or aromatic ring features, the ethanolamine bridge oxygen atom was denoted as HBA for Thr347, while the positively charged nitrogen was specified as HBD for H12 Asp351. The model was used for the virtual screening (VS) of α-mangostin derivatives that mostly occupied the H12 area. Two more matching SB 3DP models from **3ERT** were used for the VS of 2′,4′-dihydroxy-6-methoxy-3,5-dimethylchalcones [93] and chalcones [103] from *Eugenia aquea* Burm F. leaves. The **3ERT** complex was a template for the Glide [104-106]-based SB VS of UNDP, NPACT, FDA, and SANDB databases from which a PHASE [88,89]-based SB four-point 3DP model emerged, with one hydrophobic (H) feature, one hydrogen-bond acceptor feature (A), and two aromatic rings (RR), yet poorly correlated with the active site [105]. The significant progress came with creating the Schrödinger's E-Pharmacophore-based hypothesis [105] build with the aid of ligands co-crystalized with the mutated Y537S ERα complexes, namely **3UUD** [75], **4ZNV** [55], **4ZNS** [55], **5TLT** [56], **5TM8** [56], **5TN4** [56], **5TN5** [56], **5KCD** [57], **5KCT** [57], **5KD9** [57], and **5DXE** [58]. The generated ADRHRD hypothesis, paradoxically poorly discussed against the ligand-active site residues interactions, was used to design new 3-acetyl-2*H*-benzo[g]chromen-2-ones.

Results and discussion

Table S1. PDB codes and ligand structures of Estrogen Receptor α agonists, partial agonists, and antagonists currently available at Protein Data Bank (date of access 2015).

PDB	Ligand structure	pIC_{50}	pK_i	Mutation	Ref.
**1A52**	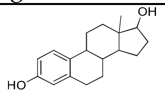	9.24	9.79	None	82
**1ERE**	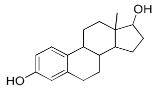	9.24	9.79	None	13
**1ERR**	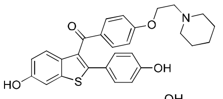	9.52	10.5	None	13
**1G50**	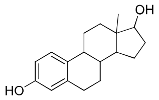	8.72	6.27	None	89
**1GWQ**	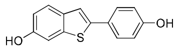	5.85	NA[a]	None	59
**1L2I**	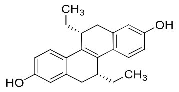	8.5	8.5	None	69
**1QKU**	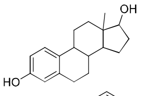	8.72	6.27	None	45
**1R5K**	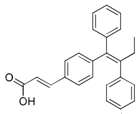	7.4	NA	None	21
**1SJ0**	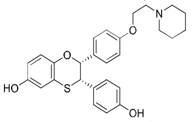	9.09	NA	None	60
**1UOM**	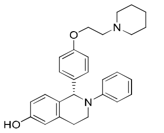	7.7	NA	None	70
**1X7E**	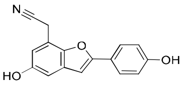	5.9	NA	None	61
**1X7R**	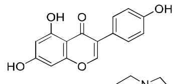	8.01	NA	None	62
**1XP1**	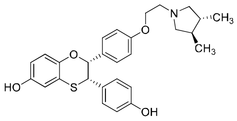	9.3	NA	None	63
**1XP6**	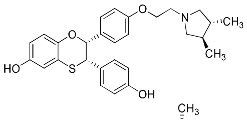	9.3	NA	None	63
**1XP9**	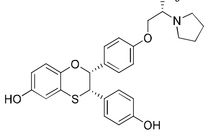	8.8	NA	None	63

**1XPC**	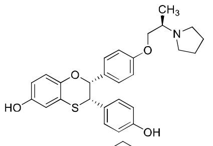	8.7	NA	None	63
**1XQC**	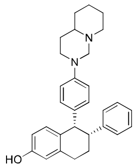	7.2	NA	None	64
**1YIM**	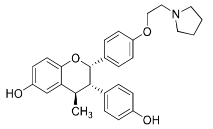	8.8	NA	None	65
**1YIN**	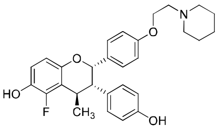	8.8	NA	None	65
**2B1Z**	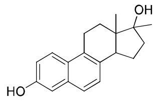	7.1	NA	C381S<br>C417S	70
**2BJ4**	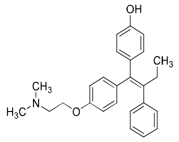	8.6	10.4	None	66
**2IOG**	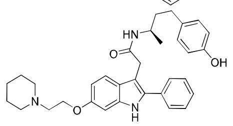	8.09	NA	None	67
**2IOK**	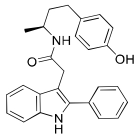	9.00	NA	None	67
**2QA6**	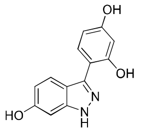	7.3	NA	Y537S	71
**2QA8**	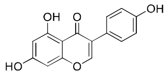	8.01	6.4	Y537S	75
**2QGT**	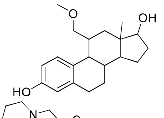				72
**2R6W**	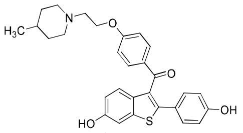	8.6	9.4	C381S<br>C417S	72
**2R6Y**	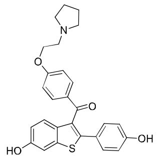	8.9	9.5	C381S<br>C417S	72

**3DT3**	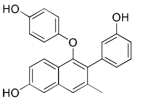	NA	NA	None	84
**3ERD**	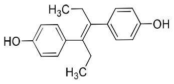	9.48	NA	None	68
**3ERT**	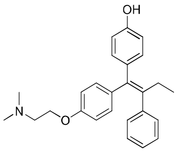	NA	9.60	None	68
**3UU7**	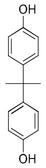	NA	8.79	Y537S	74
**3UUA**	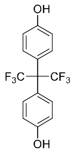	NA	8.79	Y537S	74
**3UUC**	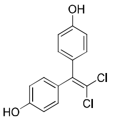	NA	5.7	None	74
**4DMA**	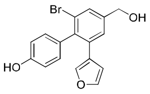	NA	5.6	None	75
**4IUI**	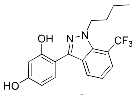	NA	NA	None	85
**4IV2**	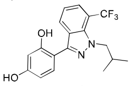	NA	NA	None	85
**4IV4**	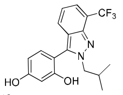	NA	NA	None	85
**4IVY**	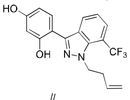	NA	NA	None	85
**4IW6**	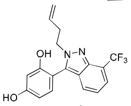	NA	NA	None	85
**4IW8**	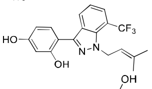	NA	NA	None	85
**4IWC**	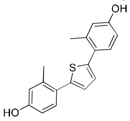	NA	NA	None	85
**4IWF**	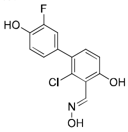	NA	NA	None	85

**4MG5**	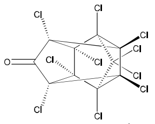	NA	NA	Y537S	77
**4MG6**	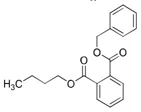	NA	6.00	Y537S	77
**4MG7**	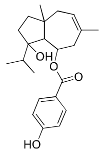	NA	NA	Y537S	77
**4MG8**	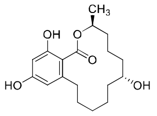	NA	10	Y537S	77
**4MG9**	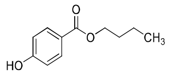	NA	6	Y537S	77
**4MGA**	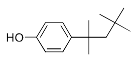	NA	6	Y537S	77
**4MGC**	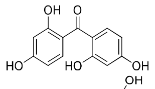	NA	7	Y537S	77
**4MGD**	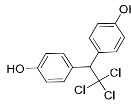	NA	6	Y537S	77
**4PPP**	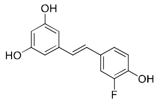	NA	NA	None	86
**4PPS**	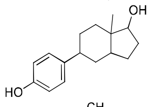	NA	NA	None	86
**4TUZ**	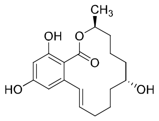	NA	10	Y537S	78
**4TV1**	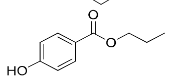	NA	NA	Y537S	78
**4ZN9**	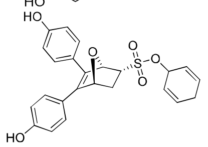	NA	9.6	Y537S	79
**5AAU**	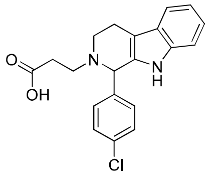	4.86	NA	None	137

**5AAV**	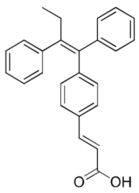	9.17	NA	None	137
**5AK2**	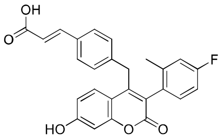	8.4	10	C381S<br>C417S	73

[a]Not available.

Table S2. Scores of the different parameters (the upper part) and PLS statistical parameters (the lower part) of the top ten hypotheses.

HID[a]	S[b]	S-I[c]	P-H[d]	S[e]	V[f]	VOL[g]	SE[h]	M[i]	A[j]	I[k]
**DDRRRP.34**	3.432	1.432	5.432	0.76	0.821	0.314	2.157	14	9.52	1.594
**AARRRP.37**	3.449	1.453	5.623	0.74	0.863	0.338	2.147	14	9.52	1.658
**DDHHHP.26**	3.338	1.324	5.321	0.77	0.872	0.367	2.153	14	9.52	1.451
**AAHHHP.46**	3.442	1.442	5.634	0.72	0.902	0.367	2.156	14	9.52	1.558
**ADDRRRP.11**	3.741	0.967	6.429	0.81	0.991	0.426	2.678	17	9.52	1.751
**ADDHHHP.13**	3.743	0.963	6.432	0.83	0.993	0.431	2.674	17	9.52	1.755
**AAAHHHP.29**	3.532	1.021	5.331	0.77	0.912	0.416	2.365	15	9.30	1.458
**AAARRRP.19**	3.561	1.113	5.231	0.74	0.922	0.426	2.358	15	9.30	1.552
**ADDHHHHP.37**	3.563	1.096	5.336	0.78	0.923	0.429	2.357	16	9.52	1.574
**ADDRRRHP.41**	3.543	1.532	5.475	0.78	0.925	0.425	2.361	16	9.52	1.594
	PLSF[l]	r^2[m]	SD[n]	F[o]	P[p]	Stability[q]	q^2_{LOO}[r]	q^2_{LSO}[s]	$q^2_{YS\ LOO}$[t]	$q^2_{YS\ LSO}$[u]
**DDRRRP.34**	4	0.912	0.214	58.9	3.94e^{-15}	0.845	0.695	0.548	-0.212	-0.127
**AARRRP.37**	4	0.918	0.216	58.3	3.98e^{-15}	0.861	0.691	0.535	-0.264	-0.126
**DDHHHP.26**	4	0.914	0.219	59.2	3.96e^{-15}	0.914	0.684	0.552	-0.187	-0.187
**AAHHHP.46**	4	0.916	0.216	58.6	4.01e^{-15}	0.904	0.687	0.539	-0.164	-0.146
**ADDRRRP.11**	5	0.949	0.264	61.3	4.38e^{-15}	0.971	0.825	0.627	-0.234	-0.247
**ADDHHHP.13**	5	0.951	0.257	61.4	4.41e^{-15}	0.977	0.826	0.659	-0.241	-0.258
**AAAHHHP.29**	4	0.924	0.228	56.4	4.26e^{-15}	0.934	0.712	0.623	-0.174	-0.136
**AAARRRP.19**	4	0.931	0.234	54.3	4.29e^{-15}	0.954	0.705	0.593	-0.165	-0.187
**ADDHHHHP.37**	4	0.329	0.217	52.7	4.31e^{-15}	0.941	0.726	0.617	-0.175	-0.134
**ADDRRRHP.41**	4	0.935	0.246	53.3	4.33e^{-15}	0.956	0.722	0.618	-0.264	-0.129

[a] Hypothesys identification; [b]Survival - the weighted combination of the vector, site, volume, and survival scores, and a term for the number of matches; [c]Survival-inactives - survival score for actives with a multiple of the survival score for inactives subtracted; [d]Post-hoc - the result of rescoring; [e]Site score – a measure of site points superimposition in an alignment to the pharmacophore of the structures that contribute to this hypothesis, based on the RMS deviation of the site points of a ligand from those of the reference ligand; [f]Vector alignment score – a measure of vectors for acceptors, donors, and aromatic rings alignedment in the structures that contribute to this hypothesis when the structures themselves are aligned to the pharmacophore; [g]Volume – a measure how much the volumes of the contributing structures overlap when aligned on the pharmacophore; [h]Selectivity - the fraction of molecules likely to match the hypothesis regardless of their potency against the receptor; [i]Matches - Number of actives that match the hypothesis; [j]Activity - Activity of the reference ligand; [k]Inactive - Survival score of inactives; [l]PLS factor, *i.e.* N/5, where N is the number of ligands present in the training set; [m]Conventional square-correlation coefficient. [n]Standard deviation of regression; [o]Ratio of the model variance to the observed activity variance; [p]Significance level of variance ratio; [q]Stability of the model predictions to changes in the training set composition; [r]Cross-validation correlation coefficient using the leave-one-out method. [d]Cross-validation correlation coefficient using the leave-some-out (LSO) method with 5 random groups; [t]Average cross-validation correlation coefficient using the leave-one-out (LOO) method obtained after Y-scrambling process. [u]Average cross-validation correlation coefficient using the leave-some-out (LSO) method with 5 random groups obtained after Y-scrambling process.

Table S3. Experimental *vs*. recalculated and predicted training set $pIC_{50}s$ values using the **ADDHHHP.13** hypothesis and LOO cross-validation.

Comp.	Experimental	Recalculated	Error	Predicted	Error
**1ERE**	9.24	9.04	0.20	8.34	0.90
**1ERR**	9.52	9.31	0.21	9.81	-0.29
**1GWQ**	5.85	6.05	-0.20	6.05	-0.20
**1L2I**	8.50	8.33	0.17	8.46	0.04
**1RK5**	7.40	7.56	-0.16	6.75	0.65
**1SJ0**	9.09	9.21	-0.12	8.61	0.48
**1UOM**	7.70	7.87	-0.17	7.24	0.46
**1X7E**	5.90	6.13	-0.23	6.33	-0.43
**1X7R**	8.01	7.91	0.10	7.78	0.23
**1XP1**	9.30	9.56	-0.26	9.34	-0.04
**1XP6**	9.30	9.12	0.18	8.89	0.41
**1XP9**	8.80	8.74	0.06	8.16	0.64
**1XPC**	8.70	8.64	0.06	8.76	-0.06
**1XQC**	7.20	7.35	-0.15	6.85	0.35
**1YIM**	8.80	9.12	-0.32	8.12	0.68
**1YIN**	8.80	8.79	0.01	8.55	0.25
**2B1Z**	7.10	7.56	-0.46	6.55	0.55
**2BJ4**	8.60	8.43	0.17	8.12	0.48
**2IOG**	8.09	7.92	0.17	8.14	-0.05
**2IOK**	9.00	8.84	0.16	8.72	0.28
**2QA6**	7.30	7.12	0.18	7.88	-0.58
**2QA8**	9.70	9.46	0.24	8.34	1.36
**2R6W**	8.60	8.12	0.48	8.12	0.48
**2R6Y**	8.90	9.16	-0.26	9.14	-0.24
**3ERD**	9.48	9.24	0.24	9.12	0.36
**5AK2**	8.40	8.76	-0.36	8.12	0.28

Table S4. Experimental *vs*. recalculated and predicted training set $pIC_{50}s$ values using the **ADDRRRP.11** hypothesis and LOO cross-validation.

Comp.	Experimental	Recalculated	Error	Predicted	Error
**1ERE**	9.24	8.94	0.30	8.56	0.68
**1ERR**	9.52	9.13	0.39	9.68	-0.16
**1GWQ**	5.85	6.12	-0.27	6.11	-0.26
**1L2I**	8.50	8.60	-0.10	8.31	0.19
**1RK5**	7.40	7.77	-0.37	6.85	0.55
**1SJ0**	9.09	9.14	-0.05	8.75	0.34
**1UOM**	7.70	7.86	-0.16	7.12	0.58
**1X7E**	5.90	6.33	-0.43	6.69	-0.79
**1X7R**	8.01	7.99	0.02	7.46	0.55
**1XP1**	9.30	9.41	-0.11	9.34	-0.04
**1XP6**	9.30	9.07	0.23	8.79	0.51
**1XP9**	8.80	8.69	0.11	8.16	0.64
**1XPC**	8.70	8.76	-0.06	8.46	0.24
**1XQC**	7.20	7.31	-0.11	6.88	0.32
**1YIM**	8.80	9.06	-0.26	8.22	0.58
**1YIN**	8.80	8.94	-0.14	8.25	0.55
**2B1Z**	7.10	7.66	-0.56	6.74	0.36
**2BJ4**	8.60	8.39	0.21	8.07	0.53
**2IOG**	8.09	7.84	0.25	8.34	-0.25
**2IOK**	9.00	8.92	0.08	8.85	0.15
**2QA6**	7.30	7.56	-0.26	7.52	-0.22
**2QA8**	9.70	9.56	0.14	8.34	1.36
**2R6W**	8.60	8.18	0.42	8.02	0.58
**2R6Y**	8.90	9.12	-0.22	9.22	-0.32
**3ERD**	9.48	9.13	0.35	9.15	0.33
**5AK2**	8.40	8.66	-0.26	8.02	0.38

Table S5. Experimental *vs.* recalculated and predicted training set $pIC_{50}s$ values using the **ADDHHHP.13** hypothesis and LSO cross-validation.

Comp.	Experimental	Recalculated	Error	Predicted	Error
**1ERE**	9.24	8.34	0.30	8.56	0.68
**1ERR**	9.52	9.81	0.39	9.68	-0.16
**1GWQ**	5.85	6.05	-0.27	6.11	-0.26
**1L2I**	8.50	8.46	-0.10	8.31	0.19
**1RK5**	7.40	6.75	-0.37	6.85	0.55
**1SJ0**	9.09	8.61	-0.05	8.75	0.34
**1UOM**	7.70	7.24	-0.16	7.12	0.58
**1X7E**	5.90	6.33	-0.43	6.69	-0.79
**1X7R**	8.01	7.78	0.02	7.46	0.55
**1XP1**	9.30	9.34	-0.11	9.34	-0.04
**1XP6**	9.30	8.89	0.23	8.79	0.51
**1XP9**	8.80	8.16	0.11	8.16	0.64
**1XPC**	8.70	8.76	-0.06	8.46	0.24
**1XQC**	7.20	6.85	-0.11	6.88	0.32
**1YIM**	8.80	8.12	-0.26	8.22	0.58
**1YIN**	8.80	8.55	-0.14	8.25	0.55
**2B1Z**	7.10	6.55	-0.56	6.74	0.36
**2BJ4**	8.60	8.12	0.21	8.07	0.53
**2IOG**	8.09	8.14	0.25	8.34	-0.25
**2IOK**	9.00	8.72	0.08	8.85	0.15
**2QA6**	7.30	7.88	-0.26	7.52	-0.22
**2QA8**	9.70	8.34	0.14	8.34	1.36
**2R6W**	8.60	8.12	0.42	8.02	0.58
**2R6Y**	8.90	9.14	-0.22	9.22	-0.32
**3ERD**	9.48	9.12	0.35	9.15	0.33
**5AK2**	8.40	8.12	-0.26	8.02	0.38

Table S6. Experimental *vs.* recalculated and predicted training set $pIC_{50}s$ values using the **ADDRRRP.11** hypothesis and LSO cross-validation.

Comp.	Experimental	Recalculated	Error	Predicted	Error
**1ERE**	9.24	9.35	-0.11	8.89	0.35
**1ERR**	9.52	9.62	-0.10	9.01	0.51
**1GWQ**	5.85	6.47	-0.62	6.96	-1.11
**1L2I**	8.50	8.70	-0.20	8.91	-0.41
**1RK5**	7.40	7.64	-0.24	8.13	-0.73
**1SJ0**	9.09	9.17	-0.08	8.93	0.16
**1UOM**	7.70	7.59	0.11	7.70	0.00
**1X7E**	5.90	5.86	0.04	6.45	-0.55
**1X7R**	8.01	8.19	-0.18	8.34	-0.33
**1XP1**	9.30	9.30	0.00	8.88	0.42
**1XP6**	9.30	9.33	-0.03	9.14	0.16
**1XP9**	8.80	8.50	0.30	8.38	0.42
**1XPC**	8.70	8.81	-0.11	8.84	-0.14
**1XQC**	7.20	7.13	0.07	7.41	-0.21
**1YIM**	8.80	9.01	-0.21	9.21	-0.41
**1YIN**	8.80	8.77	0.03	9.46	-0.66
**2B1Z**	7.10	6.89	0.21	6.35	0.75
**2BJ4**	8.60	8.41	0.19	7.68	0.92
**2IOG**	8.09	8.05	0.04	8.62	-0.53
**2IOK**	9.00	8.94	0.06	9.42	-0.42
**2QA6**	7.30	7.15	0.15	7.27	0.03
**2QA8**	9.70	8.75	0.95	8.21	1.49
**2R6W**	8.60	8.58	0.02	8.82	-0.22
**2R6Y**	8.90	9.07	-0.17	9.05	-0.15
**3ERD**	9.48	9.75	-0.27	9.06	0.42
**5AK2**	8.40	8.24	0.16	8.29	0.11

*The Origin/Significance of the **D$_1$** Feature and the Interrelated PLS-Coefficients.*

The **D$_1$/RED*$_{PLS-coefficients}$*** alignment described the mixed hydrogen bond donating (HBD)/hydrogen bond accepting (HBA) affinity of the frequently present aromatic hydroxyl group (*i.e.* **1st PhOH**, present in all compounds except **1R5K** [59], *i.e.* GW-5638, Table 1, Supplementary Materials Figures S2D and S7D) to form hydrogen bonds (HBs) with H3 Glu353 and H6 Arg394 as in the complexes of raloxifene [13] (**Ral**, **1ERR**, the most potent SERM, Table 1, Figure 5A, in disagreement/agreement with LB [28,31,33,40]/SB [90] hypotheses, respectively), **3ERD** [69] (the second most potent ER*α* binder, Table 1, Figure 5B, similarly to the LB hypothesis described elsewhere [80]), and **1ERE** [13] (Table 1 and Figure 5D, matching the previous LB hypotheses [22,25,44]). Interestingly, the **D$_1$/RED*$_{PLS-coefficients}$*** described even the third HB, through a water molecule [13]. However, the **YELLOW*$_{PLS-coefficients}$*** map positioned in-between the **1st PhOH** and H3 Glu353 instructed the **1st PhOH** as a sterical benchmark and that the more voluminous HBD/HBA would not be suitable. On the other hand, the **D$_1$/RED*$_{PLS-coefficients}$*** were insensitive to the lack of H-bonding for ligands found in **2IOK** [68] (Table 1 and Figure 5E) and **2IOG** [68] (Table 1 and Figure 6C), whose **1st PhOH's** failed to form HBs due to ~20° in-plane rotation related to H3 Glu353, as well as for the lack of propensity for ligand in **1R5K** (Table 1, Supplementary Materials Figure S2D) [59], revealing the **3-D PhypI/3-D QSAR model** ensemble first limitations.

*The Origin/Significance of the **D$_2$** Feature and the Interrelated PLS-Coefficients.*

Furthermore, the **D$_2$** feature/**GREEN*$_{PLS-coefficients}$*/RED*$_{PLS-coefficients}$*** described the TR compounds' interactions with H11 His524 *via* ligand's second phenolic portion, namely **2nd PhOH**, as a second mixed HBD/HBA feature (see **1ERR**, **1XP1**, **1XP6**, **1SJ0**, **2R6Y**, **1XP9**, **1XPC**, **2R6W**, **1X7R**, **2QA8**, **2QA6**, **1X7E**, and **1GWQ**, Tables 1 and 2, Figures 5A and 5C, Supplementary Materials Figures S1A, S1B, S1C, S1D, S2A, S2B, Figure 6D, Supplementary Materials Figures S2C, S2C, S2D, and Figure 6F, respectively). The H11 His524 seals the ligand-occupied LBD by creating the electrostatic interactions with the H7-to-H8 loop, which in turn endows in destabilization between H11 and H12 [17-20]. The interaction of either partial agonist or SERM/SERD with H11 His524 consequently places the H12 into closed or open conformation, respectively [17-20].

Functional groups improperly interpreted as **2nd Ph-OH**, like **1ERE**'s D-ring (*S*)-17β-OH (Table 2, Figure 5D) [13], or **2B1Z**'s D-ring (*R*)-17β-OH (Table 2, Supplementary Materials Figure S3C) [71] were not superimposed to the **D$_2$** feature (similarly to other LB hypotheses [22,25,44]) but were within the **GREEN*$_{PLS-coefficients}$*** and **RED*$_{PLS-coefficients}$*** range of scope, just partly describing the interactions with H11 His524. Within **2B1Z**, the sterical rearrangement provoked the H11 His524 flipping out of the LBD, caused by the Y537S mutation [103], and was even recognized as unfavorable according to the **YELLOW*$_{PLS-coefficients}$*/RED*$_{PLS-coefficients}$***.

Furthermore, the **D$_2$/RED*$_{PLS-coefficients}$***, accompanied with the significant portion of **GREEN*$_{PLS-coefficients}$***, were aligned in-between the H11 His524 and **5AK2** (Table 2, the H6 C381S and H6-to-H7 loop C417S mutations, causing no change in estrogen-induced transactivation [103], had no particular influence on the **5AK2**'s binding as SERD [74]), genistein (**1X7R**, Table 1, and **2QA8**, Table 2; respectively), **2QA6** (Table 2), **1XQC**, and **1UOM** (Tables 1 and 2, respectively). The **5AK2**'s *m*-F atom as an HBA feature found within the 1-fluoro-3-methylbenzene portion attached to the position C3 of umbelliferone as the main core, according to the experimental data paradoxically prevented the HB with H11 His524 and reduced the **5AK2** potency (Table 2) [74]; the **D$_2$/RED*$_{PLS-coefficients}$*/GREEN*$_{PLS-coefficients}$*** suggested that the bioisosteric replacement of *p*-HBA feature with *p*-HBD/HBA one would be beneficial for the potency. Indeed, the total lack of distinct *p*-HBA feature, as in **1XQC** [65] and **1UOM** [70] (Tables 1 and 2, Supplementary Materials Figure S5B, Figure 6E), or down-position to the *m*-HBA one as in the genistein's A-ring (**1X7R** [63], Table 1, Figure 6B, **2QA8** [72], Table 2, Supplementary Materials Figure S4), resulted in no HB-based activation of H11 His524 and potency lower than 8 pIC_{50} units, or selectivity towards ERβ, respectively.

*The Origin/Significance of the **H_1**/**R_1** Feature and the Interrelated PLS-Coefficients.*

Further analysis focused on the chemical linkers between the **1st PhOH** and the **2nd PhOH**. Hence, the **H_1** (**R_1**) feature co-aligned with **YELLOW***PLS-coefficients* indicated that the steric pressure of **1st PhOH**, an integral part of **1ERR**'s, **2R6Y**'s, and **2R6W**'s 2-(4-hydroxyphenyl)benzo[*b*]thiophen-6-ol core (Supplementary Materials Figures S4A, S6C, and S7B, toward H6 should be reduced (similar to other LB [25,33] or SB [90] hypotheses). On the other hand, despite not being co-aligned with the **H_1/R_1** features, the **1ERR**'s 2,3-dihydrothiophene's sulfur atom (Table 1, Figure 5A, Supplementary Materials Figure S4A) established sterically favorable interactions with H6 Met388 [13], witnessed by **GREEN***PLS-coefficients*. Such disparity between the **3-D PhypI/3-D PhypII** and the *PLS-coefficients* could be assigned to the atom-based nature of the enclosed **3-D QSAR models**. The H6 C381S and H6-to-H7 loop C417S mutations, found in **2R6Y** and **2R6W**, seemed not to have a great influence on the potency [73].

The **BLUE***PLS-*coefficients described the 2,3-dihydrothiophene to 1,4-oxathiane replacement, found in **1XP1**'s, **1XP6**'s, **1SJ0**'s, **1XP9**'s and **1XPC**'s 4-[(3*S*)-1,4-oxathian-3-yl]phenol moiety [61,64] (Table 1, Figure 5C, Supplementary Materials Figures S1A, S1B, S1C, S1D, S2A, S4A, S6A, S6B, S6D, and S7A respectively), as unfavorable and that the 1,4-oxathiane's oxygen atom as an HBA should be replaced with HBD to prevent the potency decrease: **1XP1** and **1XP6** were among the most potent ER*α* antagonists (3rd and 4th most potent), while **1SJ0**, **1XP9** and **1XPC** showed potencies higher than 8.7 pIC_{50} units. However, the **GREEN***PLS-coefficients* and **YELLOW***PLS-coefficients* overlap suggested an aliphatic or aromatic bridge, although not too voluminous, would be suitable to interact with H6 Met388: the additionally substitutution of 1,4-oxathiane's -S- with -CH-CH_3, as in **1YIM** and **1YIN,** led to reduced potency (Table 1, Supplementary Materials Figures S1E and S1F) [66].

*The Origin/Significance of the **H_2**/**R_2** Feature and the Interrelated PLS-Coefficients.*

The **H_2** feature (Figures 5 and 6, Supplementary Materials Figures S1-S3, in accordance with the previous LB hypotheses [28,29,31,39]) (or the **R_2** feature, Supplementary Materials Figures S4-S8, in agreement with the previous LB [33,34,36] and SB [38,48,49] hypotheses) overlayed with **YELLOW***PLS-coefficients* emerged from the steric interactions of **2nd Ph-OH** with H6 Leu391, as well as with H6-to-H7 loop residues Phe404, Ile424, and Leu428, and that the bulkiness of **2nd Ph-OH** should be reduced. Opposite to **H_1/R_1**, the **H_2/R_2** features partly described the contribution of the chemical linker between the **1st PhOH** and the **2nd PhOH**, and contributed to differentiating the partial agonists from SERMs/SERDs. Thus, **3ERD** [69] (Table 1, Figure 5B, Supplementary Materials Figure S4A), and **1L2I** [2] (tetrahydrochrysene derivative Table 2, Figure 6A, Supplementary Materials Figure S5A) as ER*α* partial agonists and pure ERβ antagonists sterically interfered with H3 Ala350, H6 Leu384, H6-to-H7 loop Phe404, and Leu428 side chains through one of the ethyl groups, which were, despite being superimposed to the **H_2** feature (not foreseen in previous SB hypothesis [80]), covered with sterical *PLS-coefficients* of opposite signs, instructing that the H6-invading ethyl groups are not appropriate for SERMs/SERDs design.

The remaining **3-D PhypI/3-D PhypII** features, accompanied with the corresponding **3-D QSAR** maps, were spaned throughout the cavity enclosed by helices H3, H11, and H12 and contributed to differentiating partial agonists from antagonists (SERMs/SERDs), as well as SERMs from SERDs themselves (Tables 1 and 2). The deep insight into TR compounds' experimental conformations revealed that the latter LBD area remained unoccupied during the agonist and partial agonists binding but was saturated with specific functional groups of SERMs/SERDs [2,13,69]. Within the cavity, the key residue is H3 Thr347: its ligand-directed interactions with H11 Leu525 and H12 Leu536 determine whether LBD (*i.e.* H12) remains in a closed conformation, for which the AF-2 surface is maintained stable and there is a possibility for co-activators binding, or H12 is displaced and co-activators binding is disabled [13].

*The Origin/Significance of the **H_3**/**R_3** Feature and the Interrelated PLS-Coefficients.*

The **H_3** (**R_3**) feature co-aligned with **GREEN***PLS-coefficients*/**RED***PLS-coefficients* described the SERMs/SERDs interactions with H3 Thr347, emphasing the importance of the central phenyl ring, hereinafter labeled as

Ph, found in side chains scaffolds like **1ERR**'s [13], **2R6W**'s and **2R6Y**'s [73] C(=O)-Ph-O-(CH_2)$_2$-heterocycle (Tables 1 and 2, Figure 5A, Supplementary Materials Figures S2B, S1C, S4A, S7B, and S6C respectively), **1XP1**'s, **1XP6**'s, **1SJ0**'s, **1XP9**'s, **1YIM**'s, **1YIN**'s, and **1XPC**'s [60,63] Ph-O-(CH_2)$_2$-heterocycle (Tables 1 and 2, Figure 5C, Supplementary Materials Figures, S1A, S1B, S1D, S1E, S1F, S2A, S4C, S6A, S6B, S6D, S6E, S6F, and S7A, respectively), or Ph-O-(CH_2)$_2$-heterocycle variations like Ph-O-(CH_2)$_2$-$N^+R_1R_2$[29] or Ph-O-*trans*-CH=CH-COOH [38], found in **2BJ4** (Table 1, Figure 5F, Supplementary Materials S4F) or **5AK2**'s (Table 2, Figure 6B Supplementary Materials S5B) complexes, respectively (a pharmacophoric feature frequently found in the previous LB [28,31,33,37,43] and SB [74] hypotheses). The absence of discussed scaffolds, as in **1GWQ** [60] (Table 1, Figure 6F), representing a molecular simplification of **1ERR** [13], **2R6Y** [73], and **2R6W** [73], led to significantly diminished potency. The **Ph**'s binding to the main core *via* carbonyl group is depicted with **GREEN**$_{PLS\text{-}coefficients}$/**RED**$_{PLS\text{-}coefficients}$/**BLUE**$_{PLS\text{-}coefficients}$ only (and not a pharmacophoric feature, although the carbonyl linker of **Ral** was elsewhere LB-denoted as an HBA-lipid feature [69]), indicating that the potency could be improved with the replacement of carbonyl group with HBD bioisosteres while interacting with H11 His524. The **GREEN**$_{PLS\text{-}coefficients}$ alignment over **Ph** implied that bulkiness may be increased towards H6 Trp383, as well as towards H3 Thr347 and H12 Leu536: **Ph** sterically interfered with H3 Thr347's side chain methyl group, forcing residue shifting toward the H11 Leu25 and H12 Leu536 and resulting in the formation of H3 Thr347-H11 Leu525-H12 Leu536 hydrophobic network (stabilized by the auxiliary H3 Ala350-**Ph**-H11 Leu525 network) [13]. Still, since Thr347's side chain hydroxyl group was moved out of LBD toward H3 Met343 [13], according to the **GREEN**$_{PLS\text{-}coefficients}$/**RED**$_{PLS\text{-}coefficients}$ it should be stabilized by incorporating the *o*-hydrophobic/HBA functionality.

On the other hand, partial agonists (**3ERD** [32], **1ERE** [13], **2B1Z** [71], and **1X7R/2QA8** [63,72], Tables 1 and 2) did not enable the formation of a distinct network. Indeed, the **3ERD**'s (Figure 5B, Supplementary Materials Figure S4B) and **2B1Z**'s second ethyl group (Supplementary Materials Figures S3C, S8c, respectively), similarly to the **1ERE**'s 18α-CH_3 group (Figure 5D, Supplementary Materials Figure S4D) [13], failed to fully hydrophobically stabilize to the H3 Thr347 side chain methyl group, resulting in no AF-2 surface disruption [4,6,10,13,69]. The replacement of the **3ERD**'s ethyl group with a bulkier aromatic one, like in **2BJ4** (Table 1, Figure 5F, Supplementary Materials Figure S4F), led to the AF-2 surface disorder (*viz.* H12 opening) [4,6,10,13,69]. On the other hand, the lack of ligand-H3 Thr347 interactions, like in **1X7R** [63] (Figure 6D and Supplementary Materials Figure S5D) and **2QA8** [72] (Supplementary Materials Figure S2C and S7C) complexes, even led to selectivity towards ERβ.

*The Origin/Significance of the **A** Feature and the Interrelated PLS-Coefficients.*

Within the C(=O)-Ph-O-(CH_2)$_2$-heterocycle, Ph-O-(CH_2)$_2$-heterocycle, or similar arrangements the ethanolamine portion served as a bridge between the **Ph** and the terminal functional groups. The **A** feature (similarly to previous LB [28,34] and SB [90] hypotheses) and **RED**$_{PLS\text{-}coefficients}$ emphasized the node-like oxygen atom as the central atom within the linker (hereinafter labeled as **Oxy**), describing its electrostatic interactions with H3 Thr347's side-chain -OH group. At the same time, distinct interactions were repulsive according to the co-aligned **YELLOW**$_{PLS\text{-}coefficients}$, thus excluding the possibility for hydrogen bonding. However, the additional **YELLOW**$_{PLS\text{-}coefficients}$ indicated that the **Oxy**-H3 Thr347 interactions should not be sterically interrupted, as with (*S*)-methyl (**1XP9**, Supplementary Materials Figures S1D and S6D) or the (*R*)-methyl (**1XPC**, Supplementary Materials Figures S2A and S7A) substituents of the ethanolamine bridge, to maintain the high level of potency [64].

*The Origin/Significance of the **P** Feature and the Interrelated PLS-Coefficients.*

The **P** feature/**BLUE**$_{PLS\text{-}coefficients}$ (similarly as in the previous LB [72] and SB [74] hypotheses) emerged from the HB formed between the H3 Asp351 and the positively charged nitrogen found in terminal functional groups substituting the ethanolamine bridge: the **1ERR**'s, **1SJ0**'s, **1YIN**'s, **2R6W**'s, and **1UOM**'s piperazine. (Figure 5A, Supplementary Materials Figures S1B, S1F, and S2B, Figure 6E,

respectively) [13,60,70,73], the **1XP1**'s (3*R*,4*R*)-3,4-dimethylpyrrolidine (Figure 5C) [64], the **1XP6**'s (3*S*,4*S*)-3,4-dimethylpyrrolidine (Supplementary Materials Figure S1A) [64], the **2R6Y**'s, **1XP9**'s, **1YIM**'s, and **1XPC**'s pyrrolidine (Supplementary Materials Figure S1C, S1D, S1E, and S2A) [63,65,72], **2IOK**'s and **2IOG**'s 2-phenyl-1*H*-indole (Figures 5E and 6C) [31], and the **1XQC**'s octahydro-1*H*-pyrido[1,2-a]pyrazine (Supplementary Materials Figure S3B) [65]. The HB formed with H3 Asp351 stabilized the H12 in the open conformation and prevented the SRC-3 protein co-activator binding within either genomic direct or indirect pathways. Consequently, no unwinding of the AP-1 responsive element within the DNA occurred, alongside no recruitment of the RNAP II transcriptional machinery, and no BC development [6,10,13].

Based on the **GREEN***PLS-coefficients*, the HB with H3 Asp351 was recognized as sterically favorable. However, **YELLOW***PLS-coefficients*, overlaying either piperazine (**1ERR**), (3*R*,4*R*)-3,4-dimethylpyrrolidine (**1XP1**), (3*S*,4*S*)-3,4-dimethylpyrrolidine (**1XP6**), or the remaining similar *N*-heterocycles, indicated that the reduction of the van der Waals interactions with the H12 Leu536-Leu539-Glu542-Met530-Leu540 cross-section (*i.e.* NR box-like LLEML sequence) [13,27] might induce an easier H12 drifting and that the *N*-heterocycle could not necessarily be the most suitable carrier of the HB donor group (HBD) to interact with H3 Asp351. The heterocycles curtail to dimethylaminoethanol, as in **2BJ4** [67] (Table 1, Figure 5F), or its replacement by **HBA/HDB**-containing functional group, such as the carboxyl one within the phenyl acrylic acid (as in **1R5K** [59] and **5AK2**, *i.e.* SERD [74]), likewise resulted in H-bonding with H3 Asp351, recommending the mixed **HBA/HBD** functionalities, **HBX**, in the future rational design of effective ERα antagonists. Still, if the **HBX**s are going to be used, their implications on D351Y (associated with estrogenic response to antiestrogens, decreased basal transactivation, no change in estrogen-induced transactivation, MTA-1 decreased estrogen-induced transactivation) [75-79,103] or D351E (associated with no change in basal or estrogen-induced transactivation) [75-79,103] would remain uncertain, as distinct mutated receptors have not yet being complexed with any full antagonist. What is definite is that the interference of the phenyl acrylic acid with H3 Asp351 causes the destabilization of ERα and consequent proteasomal degradation upon H12 drifting, by means of disarranging the H-bonding network between H12 Leu536 and H12 Tyr537 in the H11-loop-H12 loop and increasing the hydrophobicity surface of the ERα LDB (the H12 Leu536, H12 Leu539, H12 Leu540, and H12 Met543 are no longer buried in the hydrophobic core) [17-20].

Alignment Assessments Rules.

To validate the **3-D PhypI/3-D QSAR model**'s predictive ability, the SB and LB alignment assessments (AA) procedures were set up, by means of Schrodinger's Glide [104-106] and Flexible Ligand Alignment (FLA) [89] programs, by applying the consensus score strategy [80,91]. The SB/LB AA rules were afterward used to align the compounds with unknown binding modes and incorporate SB/LB bioactive conformations into the prediction engine. The SB and LB AA protocols [80,91] were investigated at four levels: experimental (EC) or randomized (RC) ligand conformation re-docking (RD)/re-alignment (RA) (ECRD, RCRD, ECRA, and RCRA, respectively) and cross-docking (CD)/cross-alignment (CA) (ECCD, RCCD, ECCA, and RCCA, respectively (see Supplementary Materials *Alignment assessment rules* section). Regarding the SB AAs [80,91], Glide's XP (extra precision) mode was the best performing one (see Supplementary Materials *Structure-based alignment assessments* section, as well as Supplementary Materials Table S7 and Figures S9-S13), whereas the highest accuracy within the LB AAs was achieved with FLA's Flexible shape-based alignment (FSBA) mode (see Supplementary Materials *Ligand-based alignment assessments* section, as well as Supplementary Materials Tables S8 and S9, and Figures S14-S18).

Structure-Based Alignment Assessments.

For SB alignment assessment, ERα antagonists were evaluated by means of a previously described re/cross-docking protocol [91] (see Supplementary Materials Alignment assessment section). Two different scoring functions as implemented in Glide [103,104], namely SP (standard precision) or XP (extra precision), were used for the reproduction of ERα antagonists' experimental poses. By means of

cross-docking procedures, the flexibility of receptors was included in a discrete fashion way [91]. The calculated docking accuracy percentages (DAs%) [91] revealed that XP scoring was superior in reproducing either WT or MUT ECs in all stages, with an accuracy above the 50% (the SP scoring function failed in the RCCD stage, expressing the DA below the 40%, for WT crystals and 50% for MUT crystals, Supplementary Materials Table S7 and Figures S9-S13).

Ligand-based alignment assessments.

Similar to the SB alignment assessment protocols, the LB AA alignment assessment was carried out on either WT or MT ECs of ERα partial agonists and antagonists [91]. The LB experiments were performed by means of Flexible Ligand Alignment tool (FLA) tool [61], evaluating both Flexible shape-based alignment (FSBA) and Common scaffold alignment (CSA) modes, alongside with its available modules: Largest common Bemis-Murcko scaffold (LCBMC), Maximum common substructure (MCS), and SMARTS in all four difficulty levels. Considering the LB alignment assessment of both WT and MT (Supplementary Materials Tables S8 and S9, and Figures S14-S18) ERα antagonists, FSBA mode was the most accurate, exerting more than 50% of AA in all difficulty levels. Therefore, Glide's XP scoring function and FLA's FSBA mode were selected for the subsequent SB and LB alignment of test sets, VS, and design of novel antagonists.

Table S7. Structure-based alignment assessment for wild-type and mutated ERα antagonists.

**Cmpd.**				**Glide**				
**EC**[a]	**SP**[b]	**XP**[c]	**SP**[b]	**XP**[c]	**SP**[b]	**XP**[c]	**SP**[b]	**XP**[c]
**WT**	*ERCD*		*RCRD*		*ECCD*		*RCCD*	
**1ERE**	1.932[d]	1.944	1.973	1.972	2.203	2.125	2.524	2.225
**1ERR**	1.413	1.325	1.454	1.403	1.584	1.506	1.905	1.606
**1GWQ**	3.899	3.811	4.940	4.889	9.070	8.992	9.391	9.092
**1R5K**	2.477	1.889	2.518	1.967	2.648	2.570	2.969	2.670
**1SJ0**	1.895	1.807	1.936	1.885	1.966	1.988	2.387	1.988
**1X7E**	4.280	4.192	4.321	4.270	4.451	4.373	4.772	4.473
**1X7R**	8.851	8.763	8.892	8.841	9.022	8.944	9.343	9.044
**1XP1**	2.379	1.991	2.420	2.369	1.550	1.472	1.871	1.572
**1XP6**	2.148	1.960	2.189	2.138	1.919	1.841	2.640	1.941
**1XP9**	2.039	1.951	2.080	2.029	1.990	1.932	2.531	1.992
**1XPC**	1.867	1.779	1.908	1.857	2.038	1.960	2.359	2.060
**1XQC**	1.838	1.750	1.879	1.828	2.009	1.931	2.330	2.031
**1YIM**	2.670	2.582	2.711	2.660	2.841	2.763	3.162	2.863
**1YIN**	2.274	2.186	2.315	2.264	2.445	2.367	2.766	2.467
**2BJ4**	2.888	2.800	2.929	2.878	3.059	2.981	3.38	3.081
**2IOG**	1.438	1.350	1.479	1.428	1.609	1.531	1.930	1.631
**2IOK**	1.742	1.654	1.783	1.732	1.913	1.835	2.234	2.935
**3ERD**	2.052	1.964	2.093	2.042	2.223	2.145	2.544	2.245
**DA%**[e]	58.33	72.22	61.11	63.88	55.55	66.66	38.88	55.55
**MUT**	*ERCD*		*RCRD*		*ECCD*		*RCCD*	
**1L2I**	1.358[d]	1.248	1.451	1.384	2.651	1.987	2.984	1.989
**1UOM**	2.432	2.341	2.658	2.684	1.985	1.894	2.251	1.954
**2B1Z**	1.943	1.894	2.651	2.651	2.215	2.147	2.517	2.541
**2QA6**	2.453	1.897	2.589	2.517	2.694	2.651	2.614	2.964
**2R6W**	2.983	2.226	2.654	2.691	3.215	2.894	3.874	2.846
**2R6Y**	2.599	2.594	2.698	2.478	2.658	2.584	2.659	2.512
**4TUZ**	1.771	1.559	1.894	1.751	2.121	2.224	2.658	2.229
**5AK2**	2.345[f]	2.368	2.654	2.546	2.651	2.195	2.651	2.185
**DA%**[e]	68.75	75	62.5	62.5	50	62.5	43.75	62.5

[a]Experimental conformation. [b]Best docked conformation obtained by means of Glide's SP module. [c]Best docked conformation obtained by means of Glide's XP module. [d]Root-Mean-Square-Deviation measured between the heavy atoms of the ligand's experimental and the ligand's aligned conformation. [g]Docking accuracy.

Table S8. Ligand-based alig66nment assessment for wild-type antagonists.

Cmpd.	Flexible Ligand Alignment							
	FSBA[b]	CSA			FSBA	CSA		
		LCBMC[c]	MCS[d]	SMARTS[e]		LCBMC[c]	MCS[d]	SMARTS[e]
EC[a]	*ECRA*				*RCRA*			
1ERE	0.884[f]	1.120	1.248	0.997	1.052	1.55	1.531	1.969
1ERR	0.984	1.652	1.348	1.097	1.152	1.65	1.631	2.069
1GWQ	0.584	0.965	0.948	0.697	0.752	1.25	1.231	1.669
1QKU	0.625	0.594	0.989	0.738	0.793	1.291	1.272	1.71
1R5K	0.984	1.326	1.348	1.097	1.152	1.65	1.631	2.069
1SJ0	1.625	1.562	1.989	1.738	1.793	2.291	2.272	2.71
1X7E	0.685	1.851	1.049	0.798	0.853	1.351	1.332	1.77
1X7R	1.333	1.451	1.697	1.446	1.501	1.999	1.98	2.418
1XP1	0.635	0.894	0.999	0.748	0.803	1.301	1.282	1.72
1XP6	1.025	1.236	1.389	1.138	1.193	1.691	1.672	2.11
1XP9	1.268	1.351	1.632	1.381	1.436	1.934	1.915	2.353
1XPC	1.136	1.025	1.500	1.249	1.304	1.802	1.783	2.221
1XQC	0.782	0.895	1.146	0.895	0.95	1.448	1.429	1.867
1YIM	1.324	1.326	1.688	1.437	1.492	1.99	1.971	2.409
1YIN	1.265	1.158	1.629	1.378	1.433	1.931	1.912	2.35
2BJ4	1.635	1.459	1.999	1.748	1.803	2.301	2.282	2.72
2IOG	1.235	1.741	1.599	1.348	1.403	1.901	1.882	2.32
2IOK	1.845	1.269	2.209	1.958	2.013	2.511	2.492	2.93
3ERD	0.694	0.784	1.058	0.807	0.862	1.36	1.341	1.779
AA%[g]	100	100	97.22	100	97.22	94.44	94.44	61.11
	ECCA				*RCCA*			
1ERE	1.859	2.212	2.826	2.481	2.608	2.577	2.726	2.725
1ERR	1.959	2.312	2.926	2.581	2.708	2.677	2.826	2.825
1GWQ	1.559	1.912	2.526	2.181	2.308	2.277	2.426	2.425
1QKU	1.600	1.953	2.567	2.222	2.349	2.318	2.467	2.466
1R5K	1.959	2.312	2.926	2.581	2.708	2.677	2.826	2.825
1SJ0	2.600	2.953	3.567	3.222	2.349	3.318	3.467	3.466
1X7E	1.660	2.013	2.627	2.282	2.409	2.378	2.527	2.526
1X7R	2.308	2.661	3.275	2.930	2.057	3.026	3.175	3.174
1XP1	1.611	1.963	2.577	2.232	2.359	2.328	2.477	2.476
1XP6	2.000	2.353	2.967	2.622	2.749	2.718	2.867	2.866
1XP9	2.243	2.596	3.210	2.865	2.992	2.961	3.110	3.109
1XPC	2.111	2.464	3.078	2.733	2.860	2.829	2.978	2.977
1XQC	1.757	2.11	2.724	2.379	2.506	2.475	2.624	2.623
1YIM	2.299	2.652	3.266	2.921	2.048	3.017	3.166	3.165
1YIN	2.240	2.593	3.207	2.862	2.989	2.958	3.107	3.106
2BJ4	2.610	2.963	3.577	3.232	2.359	3.328	3.477	3.476
2IOG	2.210	2.563	3.177	2.832	2.959	2.928	3.077	3.076
2IOK	2.820	3.173	3.787	3.442	2.569	3.538	3.687	3.686
3ERD	1.669	2.022	2.636	2.291	2.418	2.387	2.536	2.535
AA%	55.55	77.77	27.77	44.44	50.00	38.88	30.55	30.55

[a]Experimental conformation. [b]Best aligned conformation obtained by means of Flexible Ligand Alignment's FSBA mode. [c]Best aligned conformation obtained by means of Flexible Ligand Alignment's CSA mode and LCBMC module. [d]Best aligned conformation obtained by means of Flexible Ligand Alignment's CSA mode and MCS module. [e]Best aligned conformation obtained by means of Flexible Ligand Alignment's CSA mode and SMARTS module. [f]Root-Mean-Square-Deviation measured between the heavy atoms of the ligand's experimental and the ligand's aligned conformation. [g]Alignment accuracy.

Table S9. Ligand-Based Alignment Assessment for Mutated (MT) ERα Antagonists.

**Cmpd.**	**Flexible Ligand Alignment**							
	**FSBA[b]**	**CSA**			**FSBA[a]**	**CSA**		
		**LCBMC[c]**	**MCS[d]**	**SMARTS[e]**		**LCBMC[c]**	**MCS[d]**	**SMARTS[e]**
**EC[b]**		*ECRA*				*RCRA*		
**1L2I**	1.141	1.481	1.594	1.845	1.915	1.784	2.116	1.978
**1UOM**	1.241	1.594	1.953	1.561	1.594	2.849	1.162	1.774
**2B1Z**	0.841	1.658	1.597	0.849	1.249	2.624	1.596	1.362
**2QA6**	0.882	1.854	1.595	0.695	1.625	1.658	1.694	1.547
**2R6W**	1.241	1.516	2.621	1.398	1.748	1.241	2.623	1.462
**2R6Y**	1.882	1.557	1.298	2.016	1.449	2.336	2.165	1.548
**4TUZ**	0.942	1.658	0.584	0.594	1.326	1.856	1.965	1.698
**5AK2**	1.590	1.684	1.953	1.894	1.874	1.984	2.856	2.654
**AA%[h]**	100	100	97.22	97.22	100	81.25	75.00	61.11
		ECCA				*RCCA*		
**1L2I**	2.654	2.784	2.548	2.710	2.739	2.739	3.125	2.825
**1UOM**	2.842	2.652	2.659	2.810	2.839	2.839	3.225	2.925
**2B1Z**	1.695	2.326	2.514	2.410	2.439	2.439	2.825	2.525
**2QA6**	2.953	2.996	5.652	2.451	2.480	2.480	2.866	2.566
**2R6W**	1.985	2.152	2.598	2.810	2.839	2.839	3.225	2.925
**2R6Y**	1.965	3.326	3.625	3.451	2.480	3.480	3.866	3.566
**4TUZ**	2.564	2.856	2.594	2.511	2.540	2.540	2.926	2.626
**5AK2**	1.659	2.659	2.695	3.159	2.188	3.188	3.574	3.274
**AA%[h]**	75.00	43.75	37.50	37.50	50.00	37.50	18.75	37.50

[a]Experimental conformation. [b]Best aligned conformation obtained by means of Flexible Ligand Alignment's FSBA mode. [c]Best aligned conformation obtained by means of Flexible Ligand Alignment's CSA mode and LCBMC module. [d]Best aligned conformation obtained by means of Flexible Ligand Alignment's CSA mode and MCS module. [e]Best aligned conformation obtained by means of Flexible Ligand Alignment's CSA mode and SMARTS module. [f]Root-Mean-Square-Deviation measured between the heavy atoms of the ligand's experimental and the ligand's aligned conformation. [g]Alignment accuracy.

Table S10. Chemical structures, activity, and structure-based predictions of a test set complied of compounds whose activity is in literature expressed as pIC_{50} using the **3DPhypI/3-D QSAR model** enable.

Entry	Ligand structure	pIC_{50}	Pred. pIC_{50}		Pred. pIC_{50}		Ref.
			LOO[a]	AEP[c]	LSO[b]	AEP[c]	
**Baicalein**	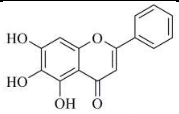	4.71	6.23	1.52	7.52	2.81	94
**Eupatorin**	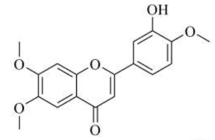	5.09	5.13	0.04	6.42	1.33	94
**Fisetin**	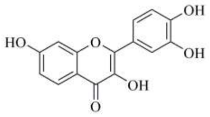	5.38	4.33	1.05	5.62	0.24	95
**Galangin**	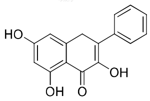	4.81	2.79	2.02	4.08	0.73	95
**Hesperetin**	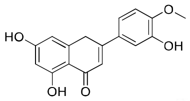	4.4	2.34	2.06	3.63	0.77	95
**Naringenin**	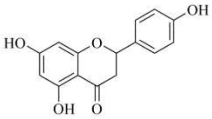	4.18	2.73	1.45	4.02	0.16	95
**Quercetin**	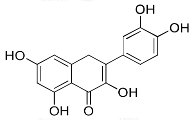	4.76	6.33	1.57	7.62	2.86	95
**I11-3a**	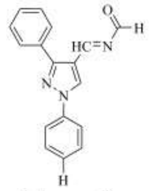	3.7	6.83	3.13	8.12	4.42	96
**I11-3b**	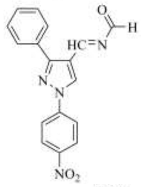	3.47	6.63	3.16	7.92	4.45	96
**I11-4a10**	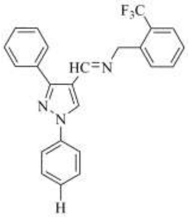	3.61	6.25	2.64	7.54	3.93	95

**I11-4a1a**	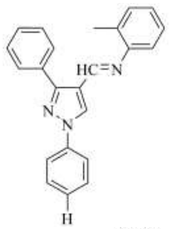	4.33	5.80	1.47	7.09	2.76	95
**I11-4a2**	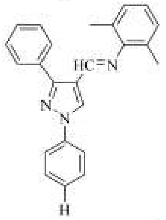	4.13	3.91	0.22	5.20	1.07	96
**I11-4a3b**	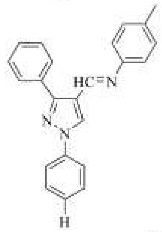	4.25	3.47	0.78	4.76	0.51	96
**I11-4a5**	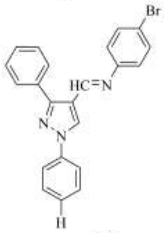	5.52	3.35	2.17	4.64	0.88	96
**I114a6**	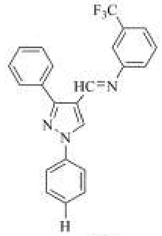	4.89	4.02	0.87	5.31	0.42	96
**I11-4a7**	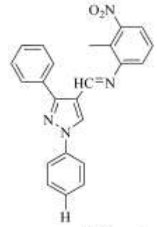	4.89	4.13	0.76	5.42	0.53	96
**I11-4a8**	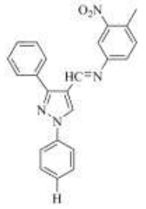	5.4	5.34	0.06	6.63	1.23	96

**I11-4a9**	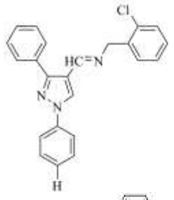	4.89	6.24	1.35	7.53	2.64	96
**I11-4b1**	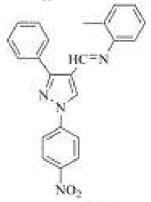	3.58	6.02	2.44	7.31	3.73	96
**I11-4b6**	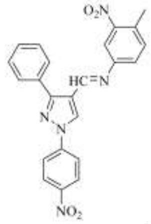	3.63	3.57	0.06	4.86	1.23	96
**I11-4b7**	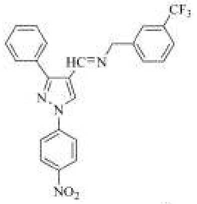	3.65	2.68	0.97	3.97	0.32	96
**I11-4b2**	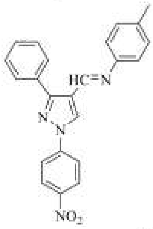	3.58	3.36	0.22	4.65	1.07	96
**I11-4b3**	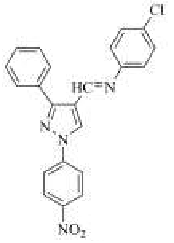	3.61	3.68	0.07	4.97	1.36	96

[a]LOO, leave-one-out cross-validation;
[b]LSO, 5-random-groups-out (K-5-Fold) cross-validation;
[c]AEP, absolute error of prediction.

Table S11. Chemical structures, activity, and ligand-based predictions of a test set complied of compounds which activity is in literature expressed as pIC_{50} using the **3DPhypI/3-D QSAR model** enable.

Entry	Ligand structure	pIC_{50}	Pred. pIC_{50}		Pred. pIC_{50}		Ref.
			LOO[a]	AEP[c]	LSO[b]	AEP[c]	
**Baicalein**	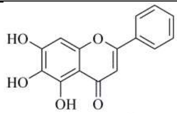	4.71	7.21	2.50	8.11	3.40	94
**Eupatorin**	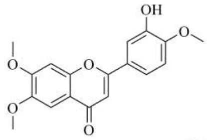	5.09	7.14	2.05	7.64	2.55	94
**Fisetin**	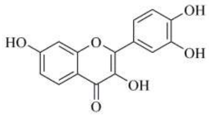	5.38	6.69	1.31	7.74	2.36	95
**Galangin**	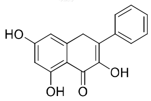	4.81	7.45	2.64	7.18	2.37	95
**Hesperetin**	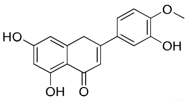	4.40	6.32	1.92	7.96	3.56	95
**Naringenin**	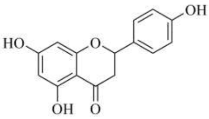	4.18	6.76	2.58	7.11	2.93	95
**Quercetin**	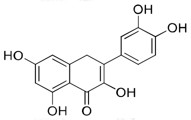	4.76	6.48	1.72	6.78	2.02	95
**I11-3a**	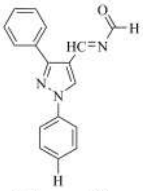	3.70	4.45	0.75	5.14	1.44	96
**I11-3b**	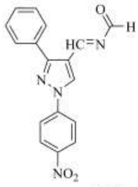	3.47	4.47	1.00	5.21	1.74	96
**I11-4a10**	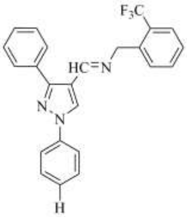	3.61	4.23	0.62	4.91	1.30	95

**I11-4a1a**	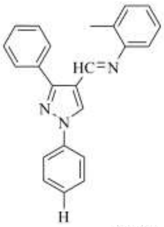	4.33	4.68	0.35	5.53	1.20	95
**I11-4a2**	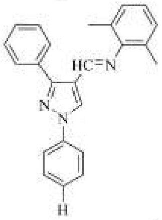	4.13	5.48	1.35	5.34	1.21	96
**I11-4a3b**	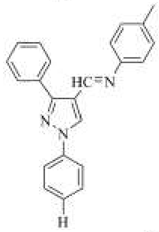	4.25	5.31	1.06	5.73	1.48	96
**I11-4a5**	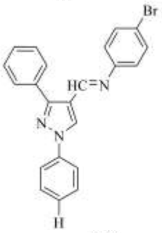	5.52	6.32	0.80	7.11	1.59	96
**I11-4a6**	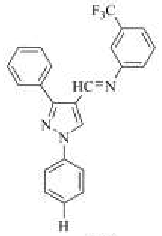	4.89	3.64	1.25	6.36	1.47	96
**I11-4a7**	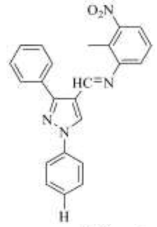	4.89	5.34	0.45	6.41	1.52	96
**I11-4a8**	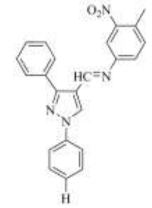	5.40	6.15	0.75	6.13	0.73	96

**I11-4a9**	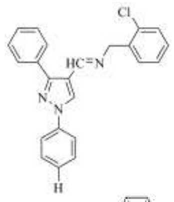	4.89	6.32	1.43	6.37	1.48	96
**I11-4b1**	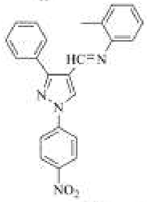	3.58	5.56	1.98	5.34	1.76	96
**I11-4b6**	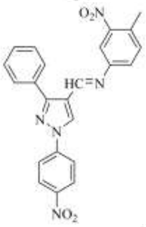	3.63	5.23	1.60	5.78	2.15	96
**I11-4b7**	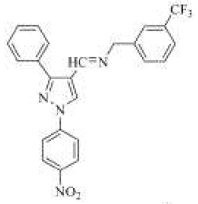	3.65	4.54	0.89	5.23	1.58	96
**I11-4b2**	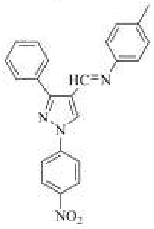	3.58	4.24	0.66	5.11	1.53	96
**I11-4b3**	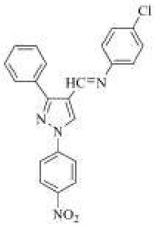	3.61	3.41	0.20	4.53	0.92	96

[a]LOO, leave-one-out cross-validation;
[b]LSO, 5-random-groups-out (K-5-Fold) cross-validation;
[c]AEP, absolute error of prediction.

Table S12. Chemical structures, activity, and structure-based predictions of a test set complied of compounds which activity is in literature expressed as pK_i using the **3DPhypI/3-D QSAR model** enable.

Entry	Ligand structure	pK_i	Pred. pK_i		Pred. pK_i		Ref.
			LOO[a]	AEP[c]	LSO[b]	AEP[c]	
**I3-I1**	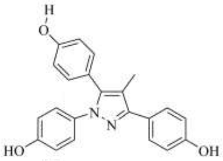	7.25	8.12	0.87	7.78	0.53	97
**I3-I2**	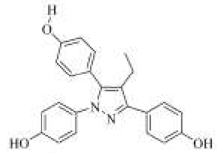	9.08	6.43	2.65	7.48	1.60	97
**I3-I3**	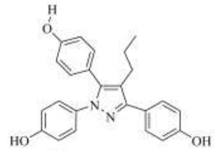	9.23	3.14	6.09	4.38	4.85	97
**I3-I4**	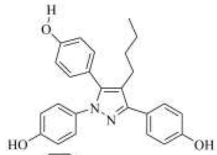	8.68	6.96	1.72	8.16	0.52	97
**I3-I-A1**	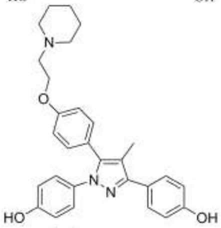	7.57	6.42	1.15	7.06	0.51	97
**I3-I-A2**	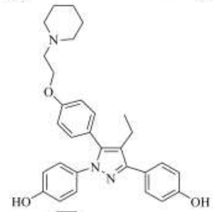	8.60	6.52	2.08	6.59	2.01	97
**I3-I-A3**	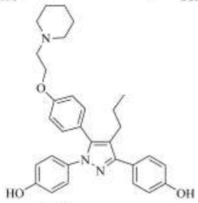	8.36	5.33	3.03	5.27	3.09	97
**I3-I-B**	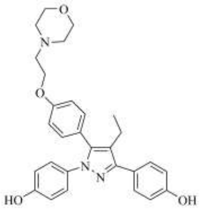	8.49	4.29	4.20	5.43	3.06	98

**I3-I-C1**	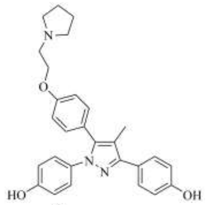	7.60	7.73	0.13	6.91	0.69	99
**I3-I-C2**	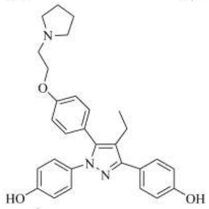	8.72	5.94	2.78	6.28	2.44	99
**I3-I-D**	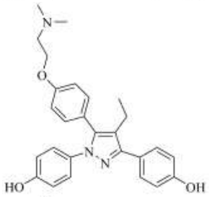	8.48	6.81	1.67	6.91	1.57	99
**I3-I-E1**	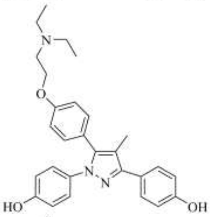	8.42	6.08	2.34	6.25	2.17	99
**I3-I-E2**	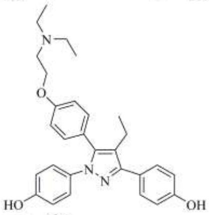	8.74	5.43	3.31	4.99	3.75	99
**I3-I-F**	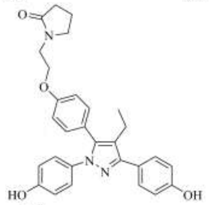	8.64	5.64	3.00	5.52	3.12	99
**I3-I-G**	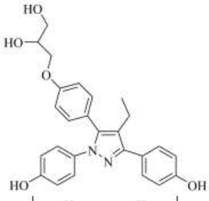	8.37	8.53	0.16	7.04	1.33	99
**I4-1a**	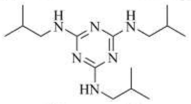	3.19	5.64	2.45	5.21	2.02	100
**I4-1b**	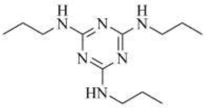	3.10	7.52	4.42	8.03	4.93	100

**I4-1c**	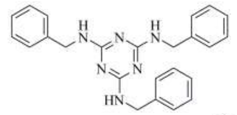	3.39	7.55	4.16	8.16	4.77	100
**I4-1e**	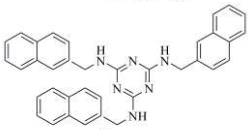	4.49	5.25	0.76	6.18	1.69	100
**I6-10**	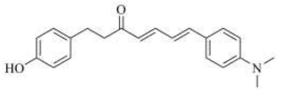	3.63	6.00	2.37	6.65	3.02	100
**I6-12**	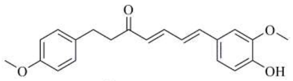	5.86	2.52	3.34	3.26	2.60	100
**I6-14**	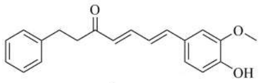	6.01	4.86	1.15	6.02	0.01	100
**I6-15**	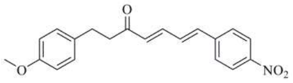	5.63	3.58	2.05	3.48	2.15	100
**I6-16**	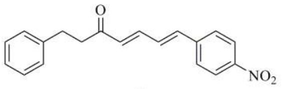	6.29	3.47	2.82	3.27	3.02	100
**I6-17**	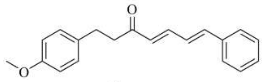	5.83	3.88	1.95	5.04	0.79	100
**I6-18**	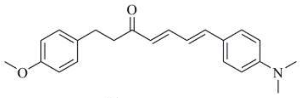	5.66	5.46	0.20	5.15	0.51	100
**I6-19**	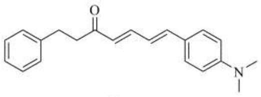	6.04	4.19	1.85	4.26	1.78	100
**I6-1**	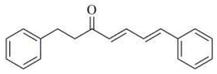	6.19	4.65	1.54	6.15	0.04	100

[a]LOO, leave-one-out cross-validation;
[b]LSO, 5-random-groups-out (K-5-Fold) cross-validation;
[c]AEP, absolute error of prediction.

Table S13. Chemical structures, activity, and ligand-based predictions of a test set complied of compounds which activity is in literature expressed as p*K*i using the **3DPhypI/3-D QSAR model** enable.

Entry	Ligand structure	p*K*i	Pred. p*K*i		Pred. p*K*i		Ref.
			LOO[a]	AEP[c]	LSO[b]	AEP[c]	
**I3-I1**	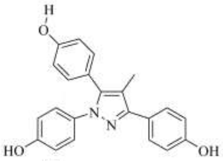	7.25	6.73	0.52	7.12	0.13	97
**I3-I2**	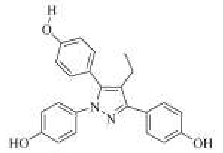	9.08	8.07	1.01	7.58	1.50	97
**I3-I3**	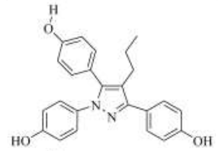	9.23	7.83	1.40	4.69	4.54	97
**I3-I4**	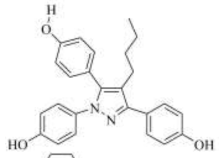	8.68	7.62	1.06	8.15	0.53	97
**I3-I-A1**	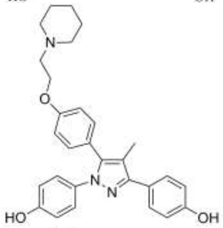	7.57	6.75	0.82	7.70	0.13	97
**I3-I-A2**	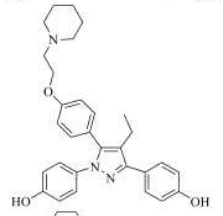	8.60	6.70	1.90	7.02	1.58	97
**I3-I-A3**	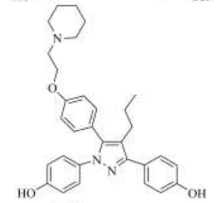	8.36	6.78	1.58	7.27	1.09	97
**I3-I-B**	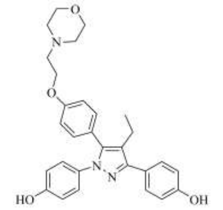	8.49	5.94	2.55	6.27	2.22	98

**I3-I-C1**	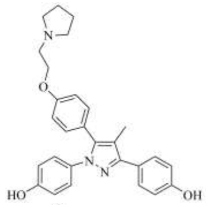	7.60	6.88	0.72	7.15	0.45	99
**I3-I-C2**	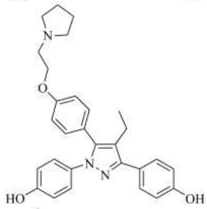	8.72	7.29	1.43	7.72	1.00	99
**I3-I-D**	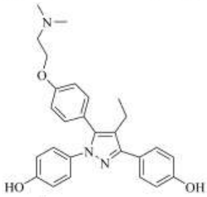	8.48	7.36	1.12	8.35	0.13	99
**I3-I-E1**	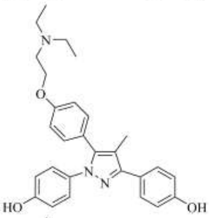	8.42	6.60	1.82	6.82	1.60	99
**I3-I-E2**	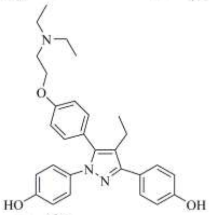	8.74	7.00	1.74	7.43	1.31	99
**I3-I-F**	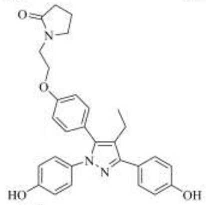	8.64	7.36	1.28	7.92	0.72	99
**I3-I-G**	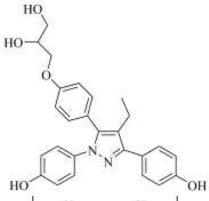	8.37	7.17	1.20	7.48	0.89	99
**I4-1a**	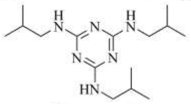	3.19	5.69	2.50	5.95	2.76	100
**I4-1b**	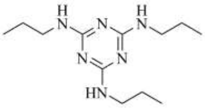	3.10	5.13	2.03	5.47	2.37	100

**I4-1c**	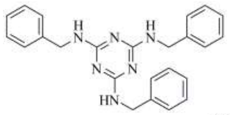	3.39	5.28	1.89	5.60	2.21	100
**I4-1e**	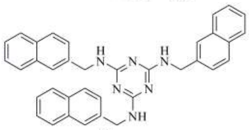	4.49	6.20	1.71	6.60	2.11	100
**I6-10**	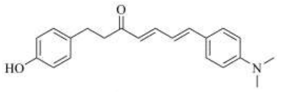	3.63	6.75	3.12	7.02	3.39	100
**I6-12**	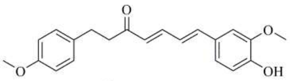	5.86	3.27	2.59	3.58	2.28	100
**I6-14**	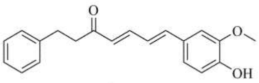	6.01	4.92	1.09	5.46	0.55	100
**I6-15**	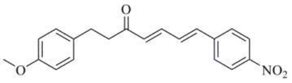	5.63	5.03	0.60	6.26	0.63	100
**I6-16**	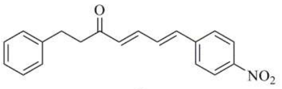	6.29	3.02	3.27	3.26	3.03	100
**I6-17**	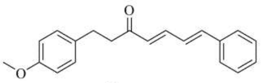	5.83	5.51	0.32	5.82	0.01	100
**I6-18**	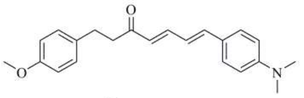	5.66	5.41	0.25	5.81	0.15	100
**I6-19**	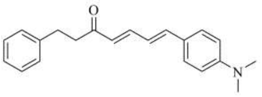	6.04	4.84	1.20	5.05	0.99	100
**I6-1**	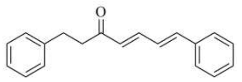	6.19	5.31	0.88	5.82	0.37	100

[a]LOO, leave-one-out cross-validation;
[b]LSO, 5-random-groups-out (K-5-Fold) cross-validation;
[c]AEP, absolute error of prediction.

Table S14. Chemical structures, activity, and structure-based predictions of a test set complied of compounds which activity is in literature expressed as pRBA using the **3DPhypI/3-D QSAR model** enable.

Entry	Ligand structure	pRBA	Pred. pRBA		Pred. pRBA		Ref.
			LOO[a]	AEP[c]	LSO[b]	AEP[c]	
**3′,4′,7-Trihydroxy isoflavone**	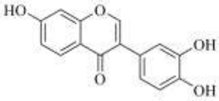	5.35	7.24	1.89	8.52	3.17	100
**3,6,4′-Trihydroxy flavone**	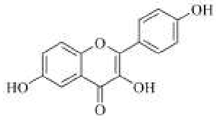	3.35	7.17	3.82	7.52	4.17	100
**3′-Hydroxyflavanone**	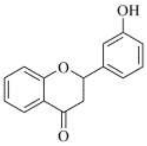	5.77	6.75	0.98	6.41	0.64	100
**4′-Hydroxyflavanone**	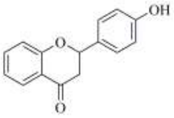	5.64	6.89	1.25	5.62	0.02	100
**6,4′-Dihydroxyflavone**	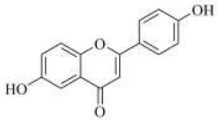	3.82	6.06	2.24	6.41	2.59	100
**6-Hydroxyflavanone**	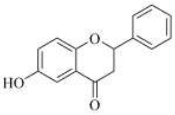	6.05	6.97	0.92	6.52	0.47	100
**6-Hydroxyflavone**	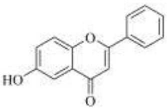	6.40	7.37	0.97	6.42	0.02	100
**7-Hydroxyflavanone**	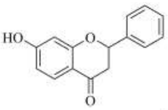	6.72	7.37	0.65	6.30	0.42	100
**Apigenin**	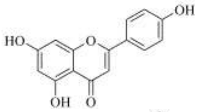	4.55	6.51	1.96	5.52	0.97	100
**Baicalein**	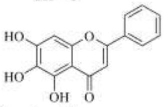	6.05	6.53	0.48	4.75	1.30	100
**Biochanin A**	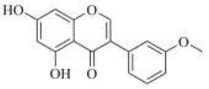	5.37	8.12	2.75	7.62	2.25	100

**Daidzein**	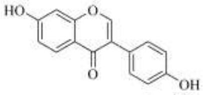	4.64	7.73	3.09	6.64	2.00	100
**Fisetin**	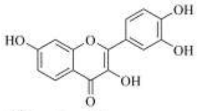	5.35	6.60	1.25	5.63	0.28	100
**Formononetin**	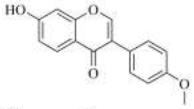	5.89	7.74	1.85	7.41	1.52	100
**Genistein**	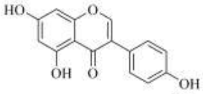	3.35	6.69	3.34	6.52	3.17	100
**Kaempferol**	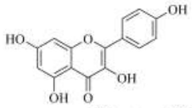	4.60	7.20	2.60	6.62	2.02	100
**Morin**	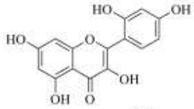	6.35	7.52	1.17	6.52	0.17	100
**Myricetin**	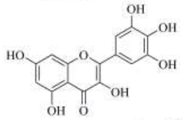	5.75	6.18	0.43	4.96	0.79	100
**Naringenin**	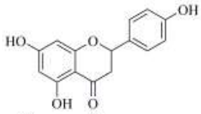	5.13	6.41	1.28	5.08	0.05	100
**Prunetin**	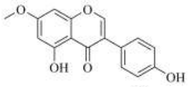	5.75	4.18	1.57	2.75	3.00	100
**Flavone**	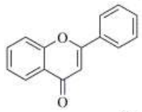	5.35	5.52	0.17	4.40	0.95	100
**I101**	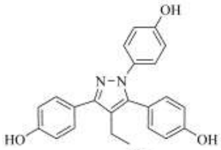	1.45	8.52	7.07	7.30	5.85	101
**I102**	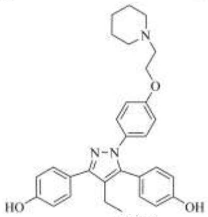	3.19	8.33	5.14	7.30	4.11	101
**I103**	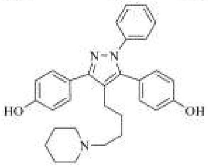	3.30	7.48	4.18	6.30	3.00	101

**I104**	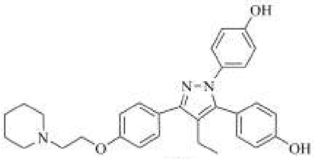	3.88	8.13	4.25	6.98	3.10	101
**I105**	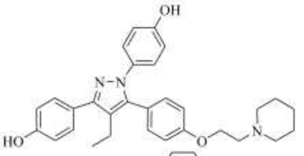	1.94	6.90	4.96	6.63	4.69	101
**I912**	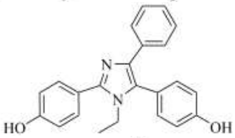	3.60	6.85	3.25	6.43	2.83	102
**I922a**	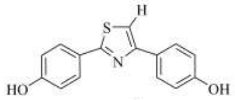	5.35	8.52	3.17	8.52	3.17	102
**I922b**	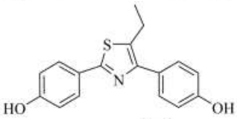	3.35	7.52	4.17	7.52	4.17	102
**I929**	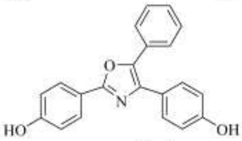	5.77	6.41	0.64	6.41	0.64	102
**I931**	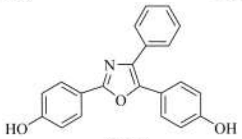	5.64	5.62	0.02	5.62	0.02	102
**I935a**	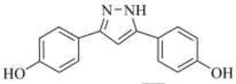	3.82	7.33	3.51	6.41	2.59	102
**I935b**	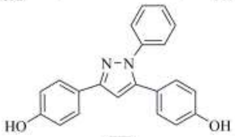	6.05	7.62	1.57	6.52	0.47	102
**I935c**	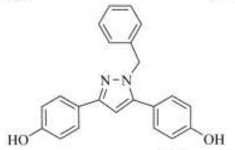	6.40	7.37	0.97	6.42	0.02	102
**I935d**	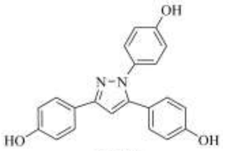	6.72	7.37	0.65	6.30	0.42	102
**I938a**	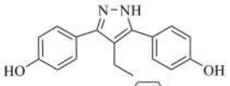	4.55	6.51	1.96	5.52	0.97	102
**I938b**	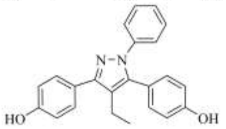	6.05	6.53	0.48	4.75	1.30	102

**I938c**	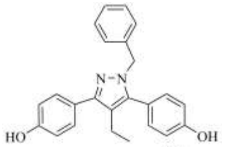	5.37	8.12	2.75	7.62	2.25	102
**I938d**	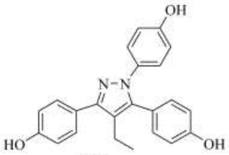	4.64	7.73	3.09	6.64	2.00	102
**I93**	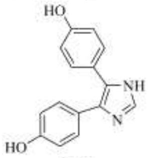	5.35	6.60	1.25	5.63	0.28	102
**I941**	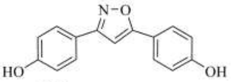	5.89	7.74	1.85	7.41	1.52	102
**I96a**	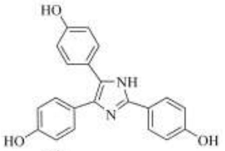	3.35	6.69	3.34	6.52	3.17	102
**I96b**	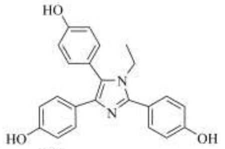	4.60	7.20	2.60	6.62	2.02	102
**I96c**	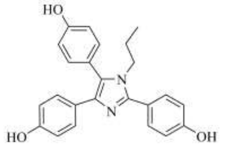	6.35	7.52	1.17	6.52	0.17	102
**I96d**	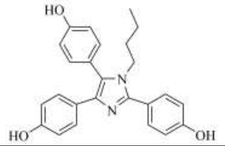	5.75	6.18	0.43	4.96	0.79	102

[a]LOO, leave-one-out cross-validation;
[b]LSO, 5-random-groups-out (K-5-Fold) cross-validation;
[c]AEP, absolute error of prediction.

Table S15. Chemical structures, activity, and ligand-based predictions of a test set complied of compounds which activity is in literature expressed as pRBA using the **3DPhypI/3-D QSAR model** enable.

Entry	Ligand structure	pRBA	Pred. pRBA		Pred. pRBA		Ref.
			LOO[a]	AEP[c]	LSO[b]	AEP[c]	
**3′,4′,7-Trihydroxy isoflavone**	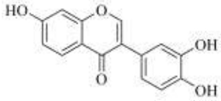	5.35	4.77	0.59	3.76	1.59	100
**3,6,4′-Trihydroxy flavone**	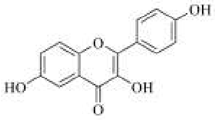	3.35	6.91	3.56	5.86	2.51	100
**3′-Hydroxyflavanone**	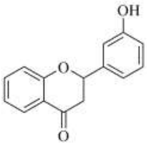	5.77	4.77	1.01	3.88	1.89	100
**4′-Hydroxyflavanone**	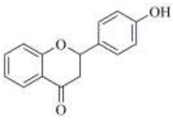	5.64	6.91	1.27	6.52	0.88	100
**6,4′-Dihydroxyflavone**	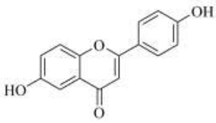	3.82	6.91	3.09	6.07	2.25	100
**6-Hydroxyflavanone**	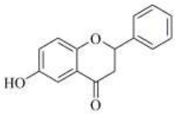	6.05	6.91	0.86	6.09	0.04	100
**6-Hydroxyflavone**	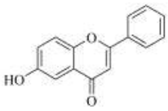	6.40	4.77	1.64	4.19	2.21	100
**7-Hydroxyflavanone**	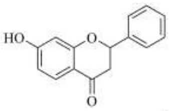	6.72	4.77	1.96	3.76	2.96	100
**Apigenin**	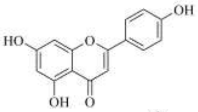	4.55	6.91	2.36	6.11	1.56	100
**Baicalein**	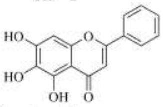	6.05	6.91	0.86	6.15	0.09	100
**Biochanin A**	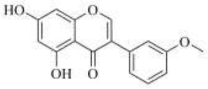	5.37	6.91	1.54	6.13	0.76	100

**Daidzein**	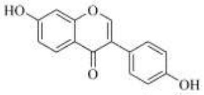	4.64	8.01	3.37	6.91	2.27	100
**Fisetin**	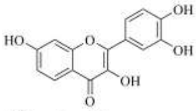	5.35	8.17	2.82	7.18	1.83	100
**Formononetin**	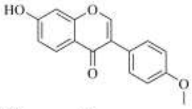	5.89	6.91	1.02	6.03	0.14	100
**Genistein**	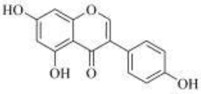	3.35	7.84	4.49	7.04	3.69	100
**Kaempferol**	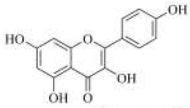	4.60	6.91	2.31	6.54	1.94	100
**Morin**	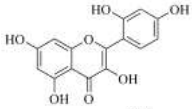	6.35	6.91	0.56	6.41	0.06	100
**Myricetin**	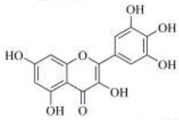	5.75	6.91	1.16	6.63	0.88	100
**Naringenin**	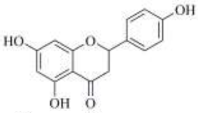	5.13	4.77	0.37	3.83	1.30	100
**Prunetin**	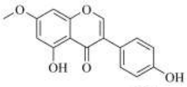	5.75	6.91	1.16	5.98	0.23	100
**Flavone**	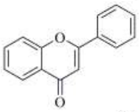	5.35	6.44	1.09	5.08	0.27	100
**I101**	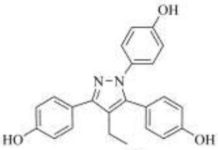	1.45	6.91	5.46	6.40	4.95	101
**I102**	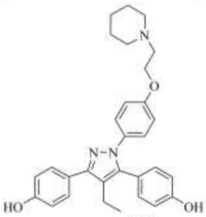	3.19	4.77	1.58	3.75	0.56	101
**I103**	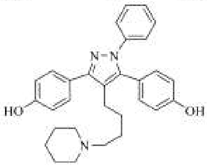	3.30	6.91	3.61	6.41	3.11	101

**I104**	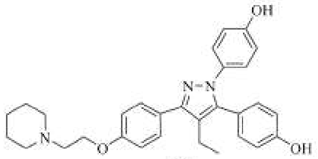	3.88	6.91	3.03	6.63	2.75	101
**I105**	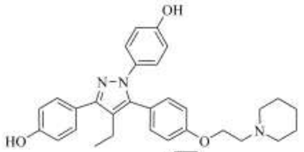	1.94	6.91	4.97	6.52	4.58	101
**I912**	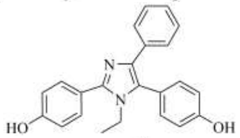	3.60	6.91	3.31	6.41	2.81	102
**I922a**	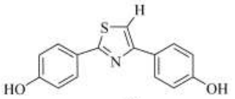	5.35	4.77	0.59	3.76	1.59	102
**I922b**	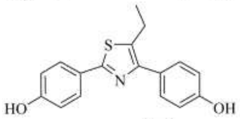	3.35	6.91	3.56	5.86	2.51	102
**I929**	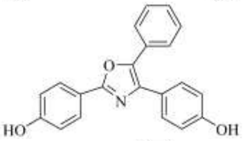	5.77	4.77	1.01	3.88	1.89	102
**I931**	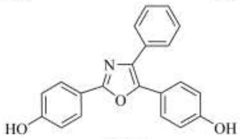	5.64	6.91	1.27	6.52	0.88	102
**I935a**	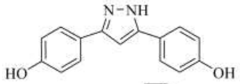	3.82	6.91	3.09	6.07	2.25	102
**I935b**	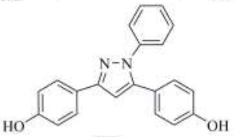	6.05	6.91	0.86	6.09	0.04	102
**I935c**	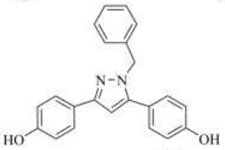	6.40	4.77	1.64	4.19	2.21	102
**I935d**	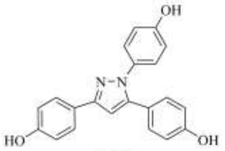	6.72	4.77	1.96	3.76	2.96	102
**I938a**	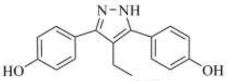	4.55	6.91	2.36	6.11	1.56	102
**I938b**	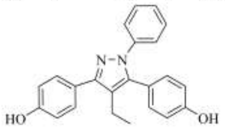	6.05	6.91	0.86	6.15	0.09	102

**I938c**	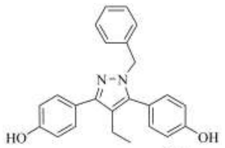	5.37	6.91	1.54	6.13	0.76	102
**I938d**	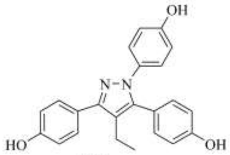	4.64	8.01	3.37	6.91	2.27	102
**I93**	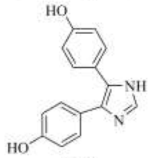	5.35	8.17	2.82	7.18	1.83	102
**I941**	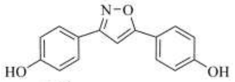	5.89	6.91	1.02	6.03	0.14	102
**I96a**	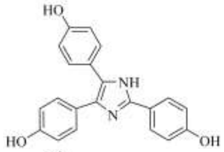	3.35	7.84	4.49	7.04	3.69	102
**I96b**	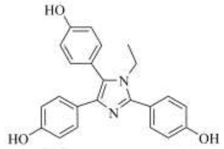	4.60	6.91	2.31	6.54	1.94	102
**I96c**	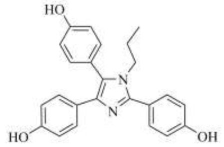	6.35	6.91	0.56	6.41	0.06	102
**I96d**	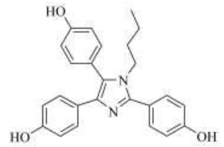	5.75	6.91	1.16	6.63	0.88	102

[a]LOO, leave-one-out cross-validation;
[b]LSO, 5-random-groups-out (K-5-Fold) cross-validation;
[c]AEP, absolute error of prediction.

Predictive ability assessment of the 3-D PhypI/3-D QSAR model ensemble.

Furthermore, **3-D PhypI/3-D QSAR model** ensemble was proven as worthy in terms of predictive ability after analyzing the literature compiled TSs predicted coefficients. Again, the SB-predicted compounds were better predicted than the LB-based ones. Thus, potencies of pIC_{50}-based TS_{MOD1}, compiled of naturally occurring compounds and their analogs, ranging from 3.47 to 5.52; were predicted with an AAEP of 1.41. Still, considering only the most potent compounds (almost 17% of the compounds, experimental pIC_{50}s lower than 10 mM and close to the TR range 5.85-9.52), the error of prediction was significantly lower (AAEP equal to 1.04), indicating the **3-D PhypI/3-D QSAR model** ensemble's applicability in perspective in virtual screening (VS) campaign. Furthermore, the **3-D PhypI/3-D QSAR model** ensemble external evaluation of TS_{MOD2} (pK_i-based) and TS_{MOD3} (pRBA-based) revealed AAEP values of 1.78 and 1.81, respectively. Again, similarly as for the TS_{CRY}, pK_i and pRBA were improperly assumed as pIC_{50}s: a small-scale VS of TS_{MOD2} resulted in 50% of compounds with the predicted potencies higher than 7 (100 nM), among which 78% of hits had pK_i values ranging from 7.25 to 9.08; among the TS_{MOD3} compounds (pRBA in the range 1.45 to 6.72), 40% had a predicted pRBA value higher than 7. Conclusively, the **3-D PhypI/3-D QSAR model** ensemble represents, either on the SB or LB level, a valuable tool for drug design or VS when considering the design or selection of new more potent ERα antagonists/partial agonists.

Table S16. NCI Codes, ligand structures, binding affinities of 18 virtually screened partial agonists against either wild type (WT) or mutated (MUT) Estrogen Receptor α, and average scores of LB alignment against the TR compounds

NCI ID	Ligand structure	WT[a]/MUT[b] ERα BA (kcal/mol)	LB ASCORE[c] *vs.* TR	NCI ID	Ligand structure	WT[a]/MUT[b] ERα BA (kcal/mol)	LB ASCORE[c] *vs.* TR
**6145**	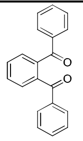	-9.4[a]<br>-9.1[b]	0.451	**59814**	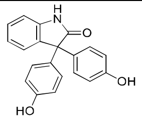	-9.7<br>-9.0	0.398
**9782**	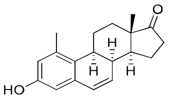	-10.8<br>-9.7	0.534	**69359**	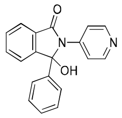	-10.1<br>-9.0	0.371
**10211**	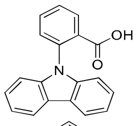	-10.2<br>-9.4	0.497	**71795**	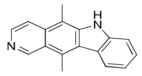	-9.8<br>-9.3	0.465
**10416**	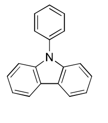	-9.7<br>-9.5	0.364	**76747**	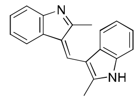	-9.7<br>-9.5	0.459
**16437**	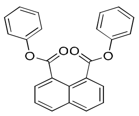	-9.2<br>-10.0	0.365	**85239**	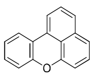	-9.4<br>-8.7	0.448
**17128**	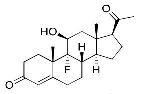	-9.3<br>-9.6	0.597	**89671**	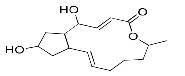	-9.8<br>-9.0	0.574
**19824**	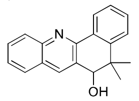	-11.0<br>-10.5	0.464	**93427**	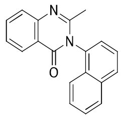	-9.4<br>-8.6	0.451
**30813**	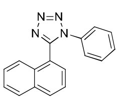	-10.0<br>-9.5	0.439	**101789**	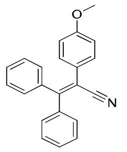	-9.6<br>-9.1	0.481
**36586**	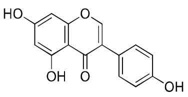	-9.1<br>-9.0	0.459	**645330**	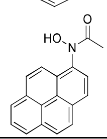	-9.4<br>-9.0	0.418

[a]The average binding affinity value from cross-docking experiment against wild type Estrogen Receptor α using the Glide's XP docking protocol; [b]The average binding affinity value from cross-docking experiment against mutated Estrogen Receptor α using the Glide's XP docking protocol; [c]The average score of cross-alignment against TR compounds using the FLA's FSBA LB alignment protocol.

Table S17. Virtually screened hits SB and LB predicted and experimental antagonistic activities against ERα, antiproliferative activity, and selectivity index against hormone-dependent MCF-7 and hormone-independent MDA-MB-231 breast cancer cell lines, antiproliferative activity against the normal MRC-5 human lung tissue fibroblasts cell lines.

NCI ID	SB. Pred. pIC_{50}[a]	LB. Pred. pIC_{50}[b]	ERα[c] (IC_{50} μM)	MCF-7[d] (IC_{50} μM)	MDA-MB-231[e] (IC_{50} μM)	SI[f]	MRC-5[g] (IC_{50} μM)
**6145**	4.02†‡§	3.78†‡§	75.54±6.46[h]†‡§	93.42±11.36†‡§	> 100‡§	NA[i]	> 100
**9782**	4.11†‡§	3.59†‡§	53.21±6.46†‡§	57.35±11.36†‡§	> 100‡§	NA	> 100
**10211**	3.98†‡§	4.14†‡§	66.93±6.46†‡§	73.94±11.36†‡§	> 100‡§	NA	> 100
**10416**	4.41†‡§	4.18†‡§	74.83±6.46†‡§	89.03±11.36†‡§	> 100‡§	NA	> 100
**16437**	4.02†‡§	3.76†‡§	61.35±6.46†‡§	67.13±11.36†‡§	86.43±11.36‡§	1.29‡§	34.84±11.36‡§
**17128**	4.44†‡§	4.12†‡§	64.31±6.46†‡§	67.53±11.36†‡§	92.94±11.36‡§	1.38‡§	23.65±11.36‡§
**19824**	3.77†‡§	3.65†‡§	97.31±6.46†‡§	> 100†‡§	> 100‡§	NA	> 100
**30813**	4.61†‡§	4.87†‡§	73.94±6.46†‡§	83.72±11.36†‡§	> 100‡§	NA	> 100
**36586**	3.56†‡§	3.96†‡§	> 100†‡§	> 100†‡§	> 100‡§	NA	> 100
**59814**	4.26†‡§	4.58†‡§	43.92±6.46†‡§	53.95±11.36†‡§	83.41±11.36‡§	1.54‡§	> 100
**69359**	4.03†‡§	3.61†‡§	58.02±6.46†‡§	61.63±11.36†‡§	34.21±11.36‡§	0.56‡§	51.52±11.36‡§
**71795**	3.86†‡§	3.48†‡§	54.35±6.46†‡§	77.77±11.36†‡§	> 100‡§	1.29‡§	> 100
**76747**	3.99†‡§	4.19†‡§	47.72±6.46†‡§	52.63±11.36†‡§	45.36±11.36‡§	0.86‡§	> 100
**85239**	4.07†‡§	4.59†‡§	55.85±6.46†‡§	69.63±11.36†‡§	8.45±11.36‡§	0.12‡§	25.92±11.36‡§
**89671**	5.09†‡§	5.06†‡§	8.34±1.34†‡§	9.01±2.78†‡§	> 100‡§	11.10‡§	> 100
**93427**	3.22†‡§	3.49†‡§	> 100†‡§	> 100†‡§	162.24±11.36‡§	1.62‡§	> 100
**101789**	4.81†‡§	4.97†‡§	17.94±1.34†‡§	25.93±11.36†‡§	> 100‡§	NA	14.23±11.36‡§
**645330**	4.81†‡§	4.78†‡§	35.31±6.46†‡§	44.72±11.36†‡§	35.78±11.36‡§	0.80‡§	24.78±11.36‡§
			**(IC_{50} nM)**	**(IC_{50} nM)**	**(IC_{50} nM)**		**(IC_{50} nM)**
**E_2**[j]	8.98	8.86	0.91±0.23	NA	NA	NA	NA
**4-OHT.**[k]	8.91	8.84	1.16±0.24	1.24±0.32	37.04±0.32	29.87	>100
**Ral.**[l]	9.04	9.00	0.77±0.15	0.90±0.22	93.12±0.24	103.47	>100
**Control**[m]	NA	NA	NA[o]	NA	NA	NA	NA

[a]The designed compounds SB predicted activities by the **3-D PhypI/3-D QSAR model** ensemble; [b]The designed compounds LB predicted activities by the **-D PhypI/3-D QSAR model** ensemble. [c]Concentration that antagonizes the 50% of ERα signaling activity; [d]Concentration that prevents the growth of 50% of MCF-7 cell lines; [d]Concentration that prevents the growth of 50% of MDA-MB-231 cell lines; [f]Selectivity index toward the cell line: [IC_{50}(MDA-MB-231)]/[IC_{50}(MCF-7)] for the antiproliferative effect of both designed compounds and reference compounds; [g]Concentration that prevents the growth of 50% of MRC-5 cell lines (human lung fibroblast cell lines, as a neutral control); [h]Results are presented as mean value ± standard deviation; [i]Not available; [j]**17β-estradiol**; [k]**4-hydroxytamoxifen**; [l]**Raloxifene**; [m]No ligand (0.9% NaCl). *$p < 0.05$ when compared with control group; †$p < 0.05$ when compared with **E_2**; ‡$p < 0.05$ when compared with **4-OTH**; §$p < 0.05$ when compared with **Ral**.

Table S18. The molecular docking studies analysis in terms of hydrogen bonding with the crucial LBD residues.

**Cmpd**	Residue with strength (length) of the established hydrogen bond (established via specific functional compound's group)					
	H3 Glu353 (d_{HB} in Å)	H6 Arg394 (d_{HB} in Å)	H3 Thr347 (d_{HB} in Å) (*via p*-CO)	H3 Thr347 (d_{HB} in Å) (*via o*-Ac-Ph)	H11 His524 (d_{HB} in Å)	H3 Asp351 (d_{HB} in Å)
**3DPQ-1**	2.951	2.830	3.244	3.431	3.937	3.083
**3DPQ-2**	3.051	3.244	3.914	3.546	3.640	3.122
**3DPQ-3**	2.891	2.839	3.316	3.254	3.958	3.222
**3DPQ-4**	2.949	2.829	3.234	3.441	3.998	3.347
**3DPQ-5**	2.993	2.824	3.262	3.553	3.972	/
**3DPQ-6**	2.841	2.950	3.567	3.543	3.905	/
**3DPQ-7**	2.891	2.817	3.175	3.352	3.394	3.922
**3DPQ-8**	2.862	2.651	3.962	3.144	3.925	/
**3DPQ-9**	2.850	2.826	3.764	3.453	3.993	3.314
**3DPQ-10**	2.848	2.835	3.919	3.565	3.975	/
**3DPQ-11**	2.863	2.931	3.892	3.445	3.981	3.136
**3DPQ-12**	3.032	3.533	3.972	3.753	3.431	3.112

Table S19. Effects of synthesized compounds-based treatment on mammary tumorigenesis seen through the serum biochemical markers.

Comp.	Dose (mg/kg)	AST[a] (U/L)	ALT[b] (A/L)	ALP[c] (A/L)	γ-GT[d] (U/L)	TBC[e] (μmol/L)
**3DPQ-1**	5	$44.78 \pm 0.61^{f,*\dagger\ddagger\|}$	$51.63 \pm 0.32^{*\dagger\ddagger\|}$	$104.53 \pm 0.75^{*\dagger\ddagger\|}$	$13.54 \pm 1.74^{*\dagger\ddagger\|}$	$15.43 \pm 0.32^{*\dagger\ddagger\|}$
	50	$49.54 \pm 0.65^{*\dagger\S\perp}$	$53.43 \pm 1.34^{*\dagger\S\perp}$	$107.52 \pm 0.6^{*\dagger\S\perp}$	$16.43 \pm 0.76^{*\dagger\S\perp}$	$19.41 \pm 0.42^{*\dagger\S\perp}$
**3DPQ-2**	5	$56.43 \pm 0.21^{*\dagger\ddagger\|}$	$46.89 \pm 0.74^{*\dagger\ddagger\|}$	$98.53 \pm 0.14^{*\dagger\ddagger\|}$	$16.52 \pm 0.42^{*\dagger\|}$	$16.43 \pm 0.36^{*\dagger\ddagger\|}$
	50	$59.54 \pm 0.32^{*\dagger\S\perp}$	$49.47 \pm 0.64^{*\dagger\S\perp}$	$119.47 \pm 0.13^{*\dagger\S\perp}$	$17.35 \pm 0.56^{*\dagger\S\perp}$	$17.72 \pm 0.56^{*\dagger\S\perp}$
**3DPQ-3**	5	$58.43 \pm 0.31^{*\dagger\ddagger\|}$	$49.84 \pm 0.59^{*\dagger\ddagger\|}$	$87.47 \pm 0.21^{*\dagger\ddagger\|}$	$10.36 \pm 0.45^{*\dagger\ddagger\|}$	$17.54 \pm 0.36^{*\dagger\ddagger\|}$
	50	$61.63 \pm 0.47^{*\dagger\S\perp}$	$55.84 \pm 0.79^{*\dagger\S\perp}$	$86.65 \pm 0.44^{*\dagger\S\perp}$	$11.64 \pm 0.84^{*\dagger\S\perp}$	$20.32 \pm 0.46^{*\dagger\S\perp}$
**3DPQ-4**	5	$57.53 \pm 0.43^{*\dagger\ddagger\|}$	$55.87 \pm 0.62^{*\dagger\ddagger\|}$	$99.41 \pm 0.15^{*\dagger\ddagger}$	$11.54 \pm 0.53^{*\dagger\ddagger}$	$19.31 \pm 0.24^{*\dagger\ddagger\|}$
	50	$59.46 \pm 0.63^{*\dagger\S\perp}$	$59.74 \pm 0.1^{*\dagger\S\perp}$	$104.52 \pm 0.31^{*\dagger\S\perp}$	$13.46 \pm 0.55^{*\dagger\S\perp}$	$22.74 \pm 0.66^{*\dagger\S\perp}$
**3DPQ-9**	5	$64.52 \pm 0.43^{*\dagger\ddagger\|}$	$46.58 \pm 0.94^{*\dagger\ddagger\|}$	$125.54 \pm 0.21^{*\dagger\ddagger\|}$	$10.32 \pm 0.21^{*\dagger\ddagger\|}$	$22.33 \pm 0.73^{*\dagger\ddagger\|}$
	50	$69.76 \pm 0.74^{*\dagger\S\perp}$	$51.95 \pm 0.86^{*\dagger\S\perp}$	$139.42 \pm 0.31^{*\dagger\S\perp}$	$12.36 \pm 0.53^{*\dagger\S}$	$24.46 \pm 0.98^{*\dagger\S\perp}$
**3DPQ-12**	5	$51.54 \pm 0.74^{*\dagger\ddagger\|}$	$50.99 \pm 0.84^{*\dagger\ddagger\|}$	$132.67 \pm 0.43^{*\dagger\ddagger\|}$	$13.53 \pm 0.35^{*\dagger\ddagger\|}$	$19.28 \pm 0.72^{*\dagger\ddagger\|}$
	50	$52.69 \pm 0.95^{*\dagger\S\perp}$	$59.65 \pm 0.84^{*\dagger\perp}$	$121.33 \pm 0.23^{*\dagger\S\perp}$	$15.54 \pm 0.83^{*\dagger\S\perp}$	$23.43 \pm 0.76^{*\dagger\S\perp}$
**4-OTH.**[g]	5	$53.76 \pm 0.32^{*\dagger\|}$	$52.44 \pm 0.31^{*\dagger\|}$	$105.32 \pm 0.31^{*\dagger\|}$	$16.54 \pm 0.31^{*\dagger\|}$	$23.56 \pm 0.75^{*\dagger\|}$
	50	$58.41 \pm 0.25^{*\dagger}$	$59.63 \pm 0.32^{*\dagger\perp}$	$131.43 \pm 0.43^{*\dagger\perp}$	$18.47 \pm 0.45^{*\dagger\perp}$	$25.57 \pm 0.76^{*\dagger\perp}$
**Ral.**[h]	5	$53.57 \pm 0.43^{*\dagger\ddagger}$	$50.14 \pm 0.35^{*\dagger\ddagger}$	$99.54 \pm 0.32^{*\dagger\ddagger}$	$11.43 \pm 0.53^{\dagger\ddagger}$	$18.53 \pm 0.95^{*\dagger\ddagger}$
	50	$58.41 \pm 0.32^{*\dagger}$	$52.46 \pm 0.42^{*\dagger\S}$	$106.43 \pm 0.54^{*\dagger\S}$	$12.35 \pm 0.31^{*\dagger\S}$	$22.46 \pm 0.98^{*\dagger\S}$
**MNU**[i]	50	$135.43 \pm 0.54^{*\ddagger\S\|\perp}$	$157.31 \pm 0.31^{*\ddagger\S\|\perp}$	$232.54 \pm 0.32^{*\ddagger\S\|\perp}$	$19.43 \pm 0.42^{*\ddagger\S\|\perp}$	$32.34 \pm 0.52^{*\ddagger\S\|\perp}$
**Control**[j]		$46.43 \pm 0.11^{\dagger\ddagger\S\|\perp}$	$40.31 \pm 0.47^{\dagger\ddagger\S\|\perp}$	$77.43 \pm 0.22^{\dagger\ddagger\S\|\perp}$	$11.46 \pm 0.45^{\dagger\ddagger\S\perp}$	$14.25 \pm 0.74^{\dagger\ddagger\S\|\perp}$

[a]The catalytic activity of aspartate transaminase; [b]The catalytic activity of alanine transaminase; [c]The catalytic activity of alkaline phosphatase; [d]The catalytic activity of γ-glutamyltransferase; [d]The total bilirubin content; [f]Results are presented as mean value ± standard deviation; [g]**4-hydroxytamoxifen**; [h]**Raloxifene**; [i]methyl nitrosourea; [j]0.9% NaCl. $^{*}p < 0.05$ when compared with negative control group; $^{\dagger}p < 0.05$ when compared with **MNU** in concentration of 50 mg/kg; $^{\ddagger}p < 0.05$ when compared with **4-OTH** in concentration of 5 mg/kg; $^{\S}p < 0.05$ when compared with **4-OTH** in concentration of 50 mg/kg; $^{\|}p < 0.05$ when compared with **Ral** in concentration of 5 mg/kg; $^{\perp}p < 0.05$ when compared with **Ral** in concentration of 50 mg/kg.

Table S20. Effects of synthesized compounds-based treatment of mammary tumorigenesis seen through the liver oxidative stress markers.

Comp.	Dose (mg/kg)	TP[a] (g/L)	GSH[b] (mg/g)	CAT[c] (μmol/mg)	SOD[d] (U/mg)	TBARS[e] (nmol/mg)
**3DPQ-1**	5	33.54±0.32$^{f,*\dagger\ddagger\parallel}$	9.54±0.53$^{*\dagger\ddagger\parallel}$	16.21±0.77$^{*\dagger\ddagger\parallel}$	7.65±0.63$^{*\dagger\ddagger\parallel}$	0.96±0.04$^{*\dagger\ddagger\parallel}$
	50	37.21±0.43$^{*\dagger\S\perp}$	9.35±0.45$^{*\dagger\S\perp}$	17.35±0.56$^{*\dagger\S\perp}$	8.83±0.51$^{*\dagger\S\perp}$	0.65±0.32$^{*\dagger\S\perp}$
**3DPQ-2**	5	36.24±0.12$^{*\dagger\ddagger\parallel}$	9.96±0.27$^{*\dagger\ddagger\parallel}$	15.54±0.51$^{*\dagger\ddagger\parallel}$	9.63±0.56$^{*\dagger\ddagger\parallel}$	0.56±0.17$^{*\dagger\ddagger\parallel}$
	50	41.54±0.32$^{*\dagger\S\perp}$	10.35±0.55$^{*\dagger\S\perp}$	17.31±0.63$^{*\dagger\S\perp}$	12.3±0.36$^{*\dagger\S\perp}$	0.75±0.21$^{*\dagger\S\perp}$
**3DPQ-3**	5	38.32±0.19$^{*\dagger\ddagger\parallel}$	9.15±0.77$^{*\dagger\ddagger\parallel}$	17.46±0.82$^{*\dagger\ddagger\parallel}$	7.32±0.18$^{*\dagger\ddagger\parallel}$	0.24±0.13$^{*\dagger\ddagger\parallel}$
	50	46.47±0.46$^{*\dagger\S\perp}$	9.74±0.45$^{*\dagger\S\perp}$	18.32±0.63$^{*\dagger\S\perp}$	11.46±0.36$^{*\dagger\S\perp}$	0.56±0.32$^{*\dagger\S\perp}$
**3DPQ-4**	5	43.53±0.51$^{*\dagger\parallel}$	9.87±052$^{*\dagger\ddagger\parallel}$	16.34±0.73$^{*\dagger\ddagger\parallel}$	8.73±0.38$^{*\dagger\ddagger\parallel}$	0.65±0.21$^{*\dagger\ddagger\parallel}$
	50	48.54±0.31$^{*\dagger\S\perp}$	10.36±0.46$^{*\dagger\S\perp}$	16.43±0.66$^{*\dagger\S\perp}$	10.57±0.73$^{*\dagger\S\perp}$	0.65±0.34$^{*\dagger\S\perp}$
**3DPQ-9**	5	39.31±0.22$^{*\dagger\ddagger\parallel}$	8.66±0.45$^{*\dagger\ddagger\parallel}$	10.83±0.46*†‡	7.74±0.37$^{*\dagger\ddagger\parallel}$	0.96±0.21$^{*\dagger\ddagger\parallel}$
	50	39.43±0.14$^{*\dagger\S\perp}$	8.89±0.63$^{*\dagger\S\perp}$	13.32±0.54$^{*\dagger\S\perp}$	14.43±0.35$^{*\dagger\S\perp}$	0.96±0.24$^{*\dagger\S\perp}$
**3DPQ-12**	5	336.12±0.78$^{*\dagger\ddagger\parallel}$	8.96±0.22$^{*\dagger\ddagger\parallel}$	14.55±0.51$^{*\dagger\ddagger\parallel}$	5.43±0.25$^{*\dagger\ddagger\parallel}$	0.92±0.18$^{*\dagger\ddagger\parallel}$
	50	389.35±0.32$^{*\dagger\S\perp}$	9.02±0.57$^{*\dagger\S\perp}$	19.46±0.75$^{*\dagger\S\perp}$	13.32±0.43$^{*\dagger\S\perp}$	1.05±0.34$^{*\dagger\S\perp}$
**4-OTH.[k]**	5	43.43±0.67$^{*\dagger\parallel}$	4.67±0.25$^{*\dagger\parallel}$	13.56±0.88$^{*\dagger\parallel}$	4.87±0.72$^{*\dagger\parallel}$	12.84±0.21$^{*\dagger\parallel}$
	50	47.46±0.25$^{*\dagger\perp}$	3.33±0.56$^{*\dagger\perp}$	20.46±0.76$^{*\dagger\perp}$	7.46±0.34$^{*\dagger\perp}$	3.63±0.32$^{*\dagger\perp}$
**Ral.[l]**	5	42.67±0.32*†‡	6.65±0.21*†‡	10.73±0.32*†‡	6.89±0.56*†‡	2.31±0.56*†‡
	50	44.43±0.22*†§	5.54±0.36*†§	14.43±0.56*†§	8.46±0.23*†§	2.63±0.21*†§
**MNU**	50	51.32±0.43$^{*\ddagger\S\parallel\perp}$	2.23±0.32$^{*\ddagger\S\parallel\perp}$	3.08±0.68$^{*\ddagger\S\parallel\perp}$	3.32±0.14$^{*\ddagger\S\parallel\perp}$	3.31±0.72$^{*\ddagger\S\parallel\perp}$
**Control**		31.36±0.84$^{\dagger\ddagger\S\parallel\perp}$	7.43±0.31$^{\dagger\ddagger\S\parallel\perp}$	14.21±0.65$^{\dagger\ddagger\S\parallel\perp}$	9.34±0.12$^{\dagger\ddagger\S\parallel\perp}$	1.61±0.05$^{\dagger\ddagger\S\parallel\perp}$

[a]The total protein content; [b]The concentration of reduced glutathione; [c]The catalytic activity of catalase; [d]The catalytic activity of superoxide dismutase; [e]The thiobarbituric acid reactive substances content; [f]Results are presented as mean value ± standard deviation; [g]**4-hydroxytamoxifen**; [h]**Raloxifene**; [i]methyl nitrosourea. $^{*}p < 0.05$ when compared with negative control group; $^{\dagger}p < 0.05$ when compared with **MNU** in concentration of 50 mg/kg; $^{\ddagger}p < 0.05$ when compared with **4-OTH** in concentration of 5 mg/kg; $^{\S}p < 0.05$ when compared with **4-OTH** in concentration of 50 mg/kg; $^{\parallel}p < 0.05$ when compared with **Ral** in concentration of 5 mg/kg; $^{\perp}p < 0.05$ when compared with **Ral** in concentration of 50 mg/kg.

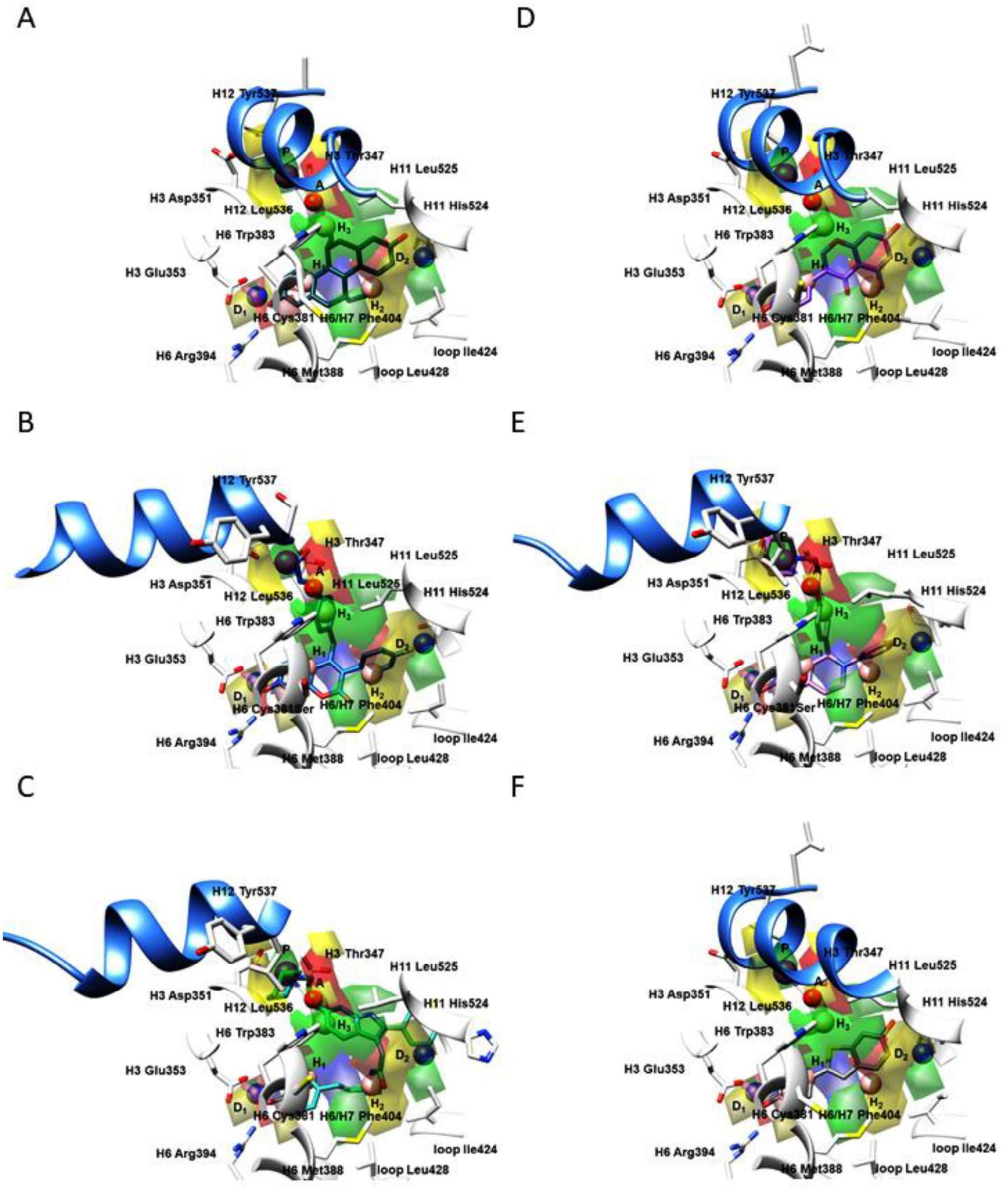

Figure S1. The **3-D PhypI** features (**D**: hydrogen-bond donators, **A**: hydrogen-bond acceptors, **H**: hydrophobic features, **P**: positive ionizable features) and **3-D QSAR** *PLS-coefficients* contour maps (**GREEN***PLS-coefficients*: positive steric interactions, **YELLOW***PLS-coefficients*; negative steric interactions, **BLUE***PLS-coefficients*: areas where positively charged functional groups and H-bond donators are favored whereas the negatively charged functional groups and H-bond acceptors are disfavored, **RED***PLS-coefficients*: areas negatively charged functional groups and H-bond acceptors are favored, whereas the positively charged functional groups and H-bond donators are disfavored) for **1L2I** (**A**), **5AK2** (**B**), **2IOG** (**C**), **1X7R** (**D**), **1UOM** (**E**), and **1GWQ** (**F**). Amino acid residues are depicted in white. For the clarity of presentation, only the H12 helix is presented in a cornflower blue ribbon, as a crucial delimiter for partial agonists, SERMs, and SERDs.

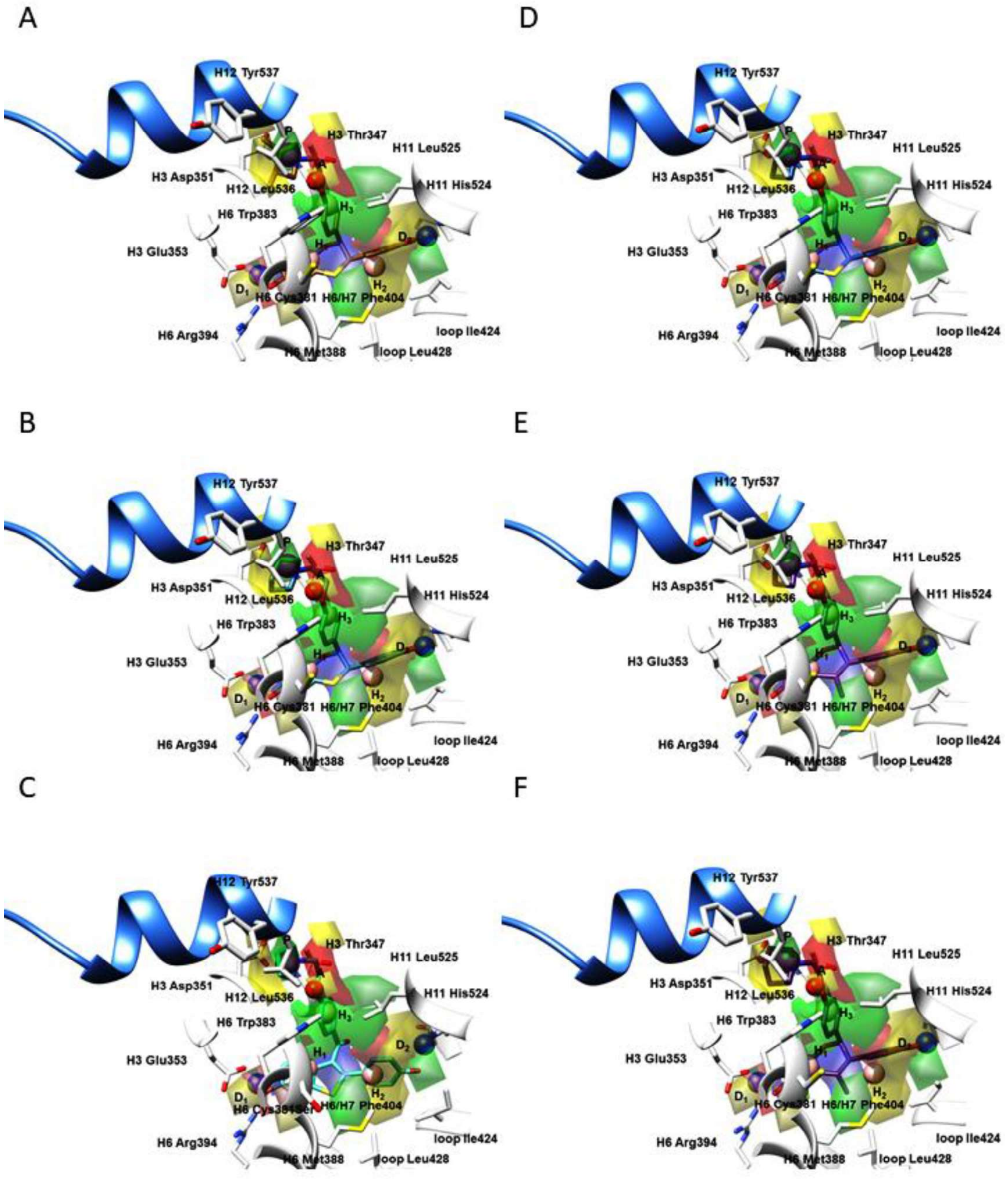

Figure S2. The **3-D PhypI** features (**D**: hydrogen-bond donators, **A**: hydrogen-bond acceptors, **H**: hydrophobic features, **P**: positive ionizable features) and **3-D QSAR** *PLS-coefficients* contour maps (**GREEN***PLS-coefficients*: positive steric interactions, **YELLOW***PLS-coefficients*; negative steric interactions, **BLUE***PLS-coefficients*: areas where positively charged functional groups and H-bond donators are favored whereas the negatively charged functional groups and H-bond acceptors are disfavoured, **RED***PLS-coefficients*: areas negatively charged functional groups and H-bond acceptors are favored, whereas the positively charged functional groups and H-bond donators are disfavored) for **1XP6** (A), **1SJ0** (B), **2R6Y** (C), **1XP9** (D), **1YIM** (E), and **1YIN** (F). Amino acid residues are depicted in white. For the clarity of presentation, only H12 helix is presented in cornflower blue ribbon, as a crucial delimiter for partial agonists, SERMs, and SERDs.

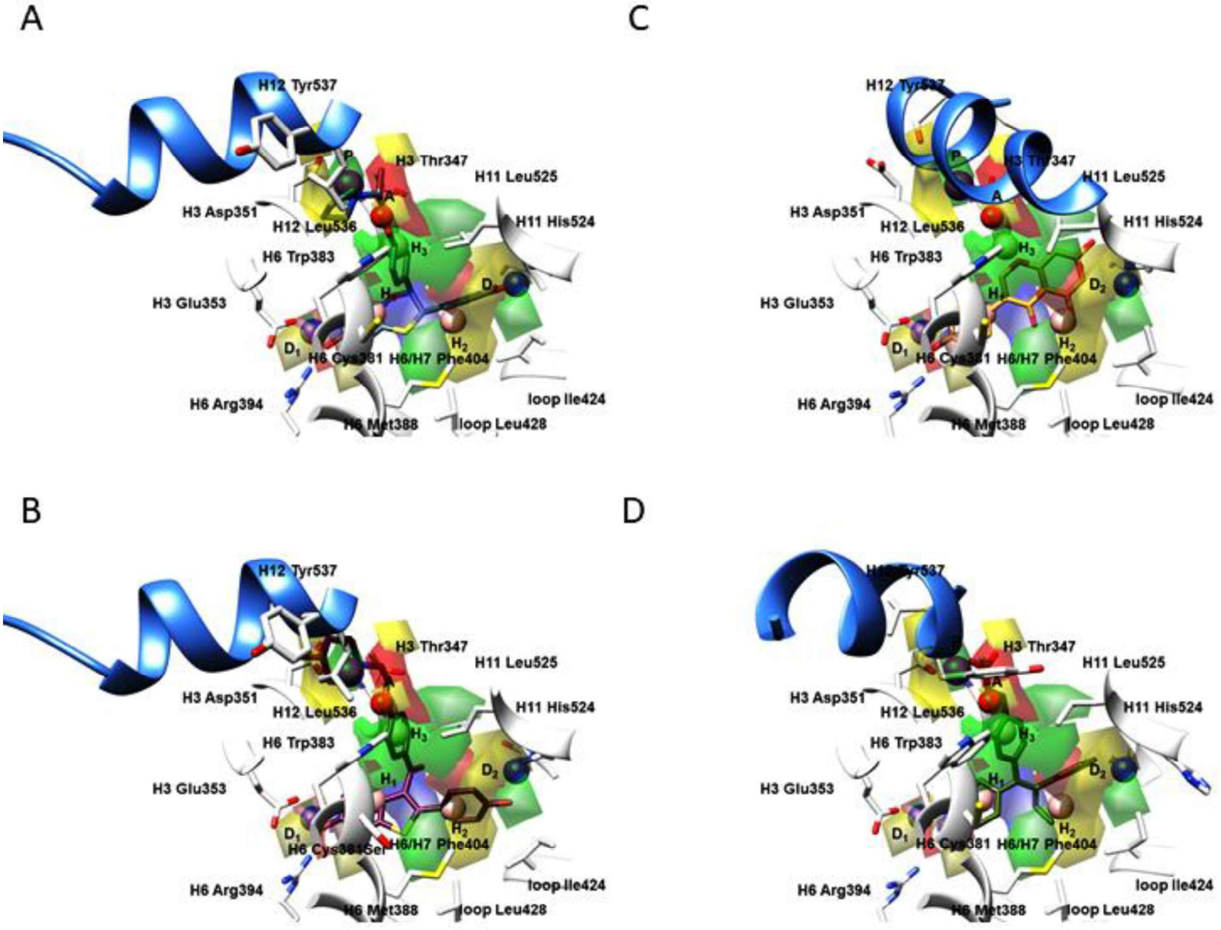

Figure S3. The **3-D PhypI** features (**D**: hydrogen-bond donators, **A**: hydrogen-bond acceptors, **H**: hydrophobic features, **P**: positive ionizable features) and **3-D QSAR** *PLS-coefficients* contour maps (**GREEN**$_{PLS\text{-}coefficients}$: positive steric interactions, **YELLOW**$_{PLS\text{-}coefficients}$; negative steric interactions, **BLUE**$_{PLS\text{-}coefficients}$: areas where positively charged functional groups and H-bond donators are favored whereas the negatively charged functional groups and H-bond acceptors are disfavoured, **RED**$_{PLS\text{-}coefficients}$: areas negatively charged functional groups and H-bond acceptors are favored, whereas the positively charged functional groups and H-bond donators are disfavored) for **1XPC** (A), **2R6W** (B), **2QA8** (C), and **1R5K** (D). Amino acid residues are depicted in white. For the clarity of presentation, only H12 helix is presented in cornflower blue ribbon, as a crucial delimiter for partial agonists, SERMs, and SERDs.

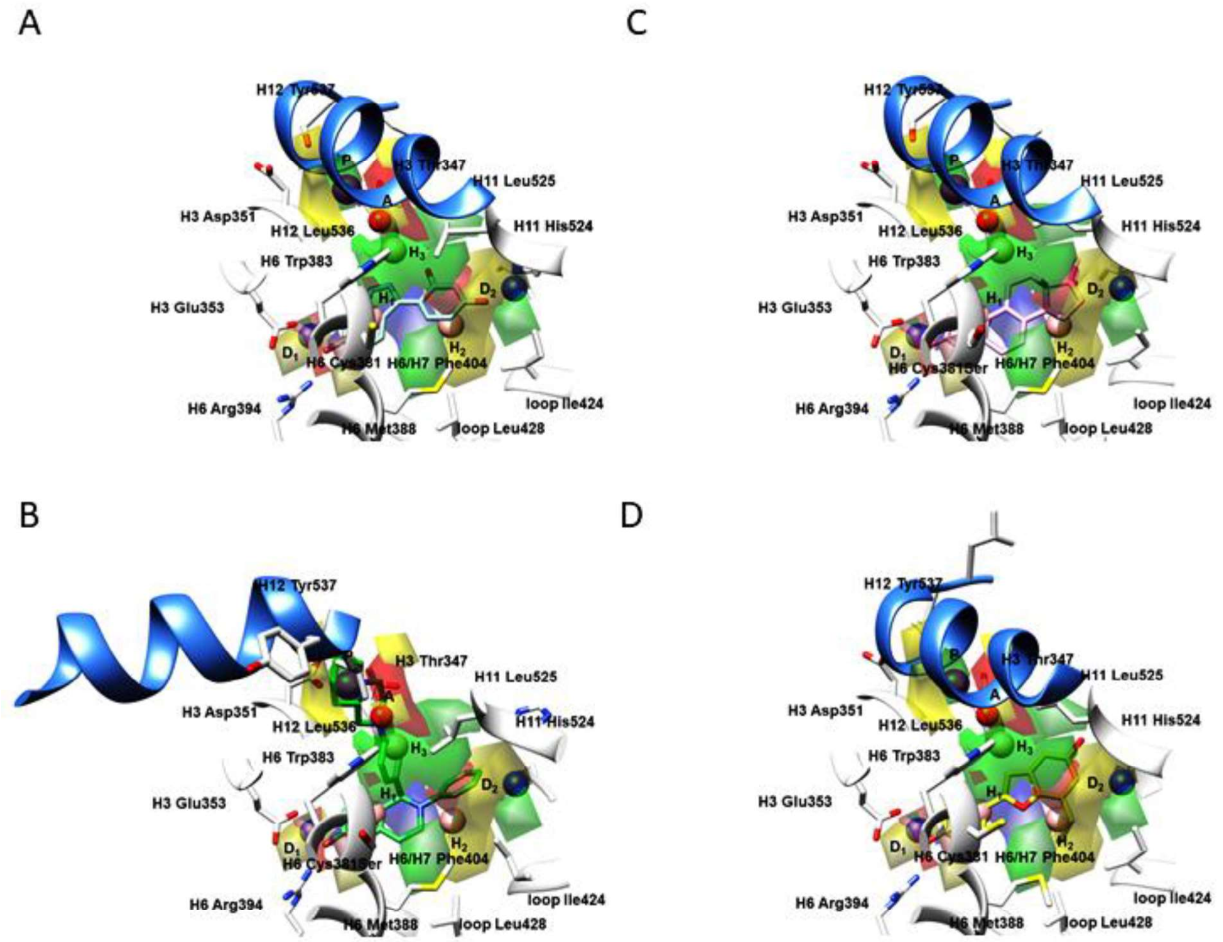

Figure S4. The **3-D PhypI** features (**D**: hydrogen-bond donators, **A**: hydrogen-bond acceptors, **H**: hydrophobic features, **P**: positive ionizable features) and **3-D QSAR** *PLS-coefficients* contour maps (**GREEN**$_{PLS\text{-}coefficients}$: positive steric interactions, **YELLOW**$_{PLS\text{-}coefficients}$; negative steric interactions, **BLUE**$_{PLS\text{-}coefficients}$: areas where positively charged functional groups and H-bond donators are favored whereas the negatively charged functional groups and H-bond acceptors are disfavoured, **RED**$_{PLS\text{-}coefficients}$: areas negatively charged functional groups and H-bond acceptors are favored, whereas the positively charged functional groups and H-bond donators are disfavored) for **2QA6** (A), **1XQC** (B), **2B1Z** (C), and **1X7E** (D). Amino acid residues are depicted in white. For the clarity of presentation, only H12 helix is presented in cornflower blue ribbon, as a crucial delimiter for partial agonists, SERMs, and SERDs.

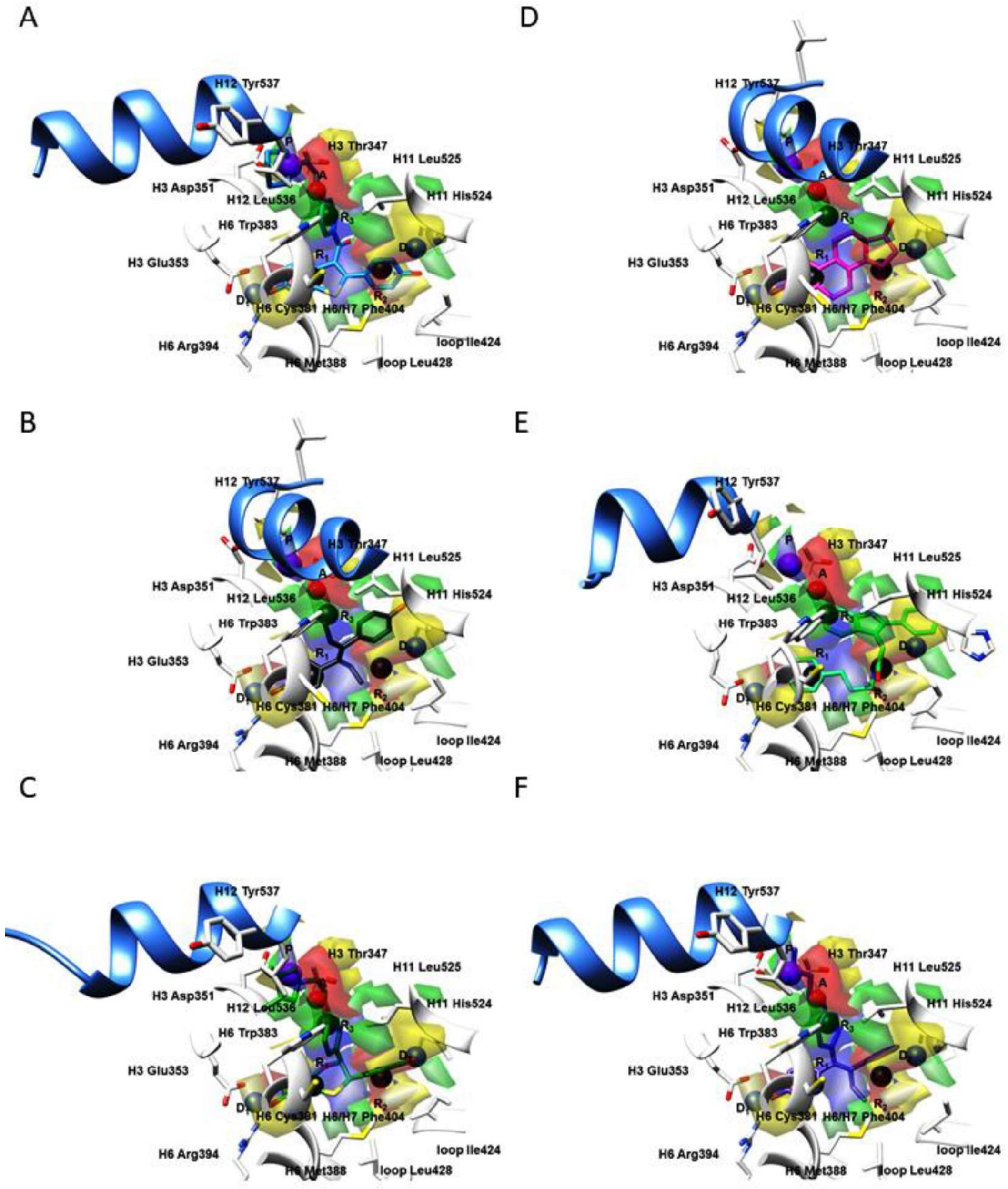

Figure S5. The **3-D PhypII** features (**D**: hydrogen-bond donators, **A**: hydrogen-bond acceptors, **R**: ring features, **P**: positive ionizable features) and **3-D QSAR** *PLS-coefficients* contour maps (**GREEN***PLS-coefficients*: positive steric interactions, **YELLOW***PLS-coefficients*: negative steric interactions, **BLUE***PLS-coefficients*: areas where positively charged functional groups and H-bond donators are favored whereas the negatively charged functional groups and H-bond acceptors are disfavoured, **RED***PLS-coefficients*: areas negatively charged functional groups and H-bond acceptors are favored, whereas the positively charged functional groups and H-bond donators are disfavored) for **1ERR** (A); **3ERD** (B); **1XP1** (C); **1ERE** (D); **2IOK** (E); **2BJ4** (F). Amino acid residues are depicted in white. For the clarity of presentation, only H12 helix is presented in cornflower blue ribbon, as a crucial delimiter for partial agonists, SERMs, and SERDs.

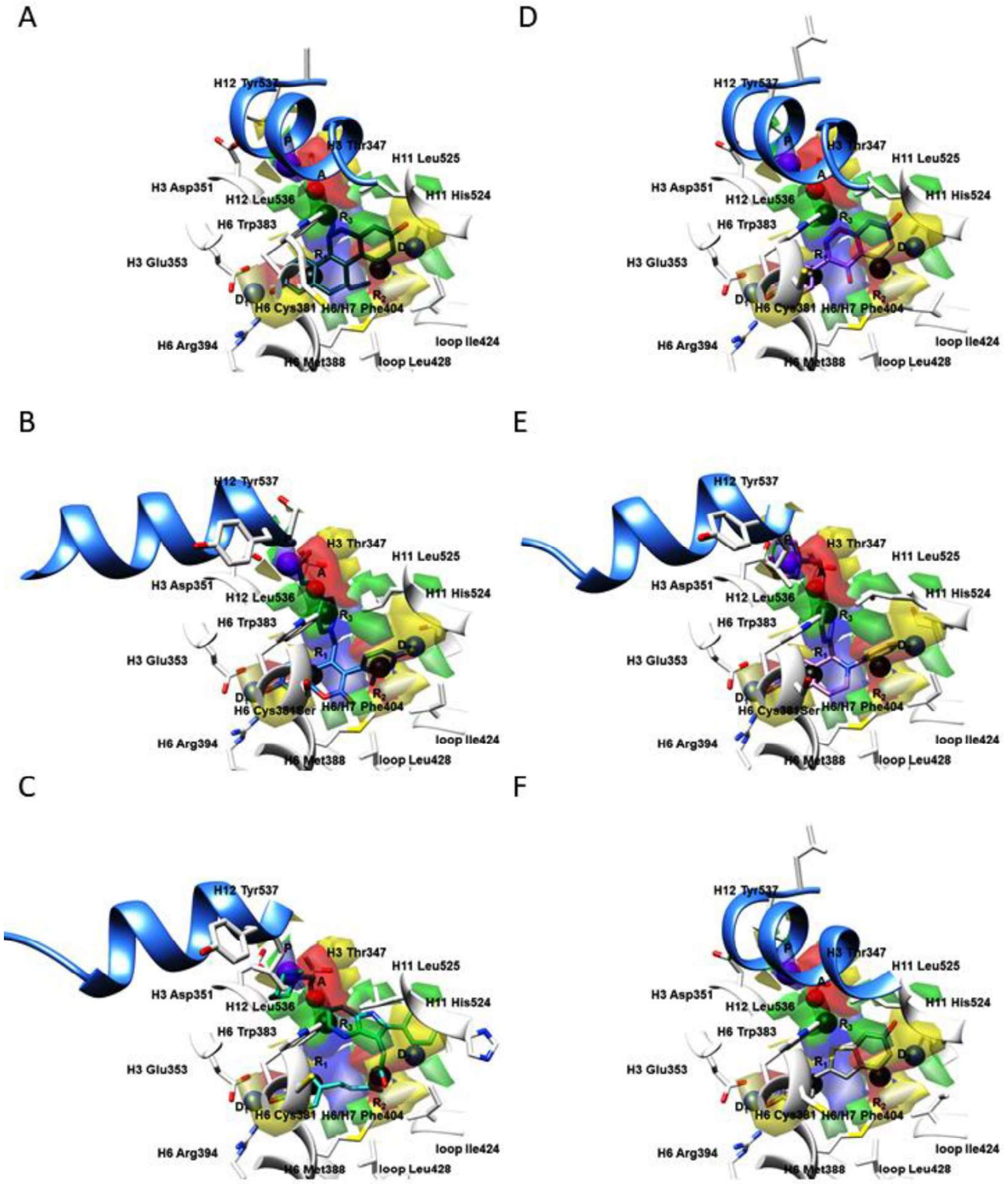

Figure S6. The **3-D PhypII** features (**D**: hydrogen-bond donators, **A**: hydrogen-bond acceptors, **R**: ring features, **P**: positive ionizable features) and **3-D QSAR** *PLS-coefficients* contour maps (**GREEN***PLS-coefficients*: positive steric interactions, **YELLOW***PLS-coefficients*: negative steric interactions, **BLUE***PLS-coefficients*: areas where positively charged functional groups and H-bond donators are favored whereas the negatively charged functional groups and H-bond acceptors are disfavoured, **RED***PLS-coefficients*: areas negatively charged functional groups and H-bond acceptors are favored, whereas the positively charged functional groups and H-bond donators are disfavored)) for **1L2I** (A), **5AK2** (B), **2IOG** (C), **1X7R** (D), **1UOM** (E), and **1GWQ** (F). Amino acid residues are depicted in white. For the clarity of presentation, only H12 helix is presented in cornflower blue ribbon, as a crucial delimiter for partial agonists, SERMs, and SERDs.

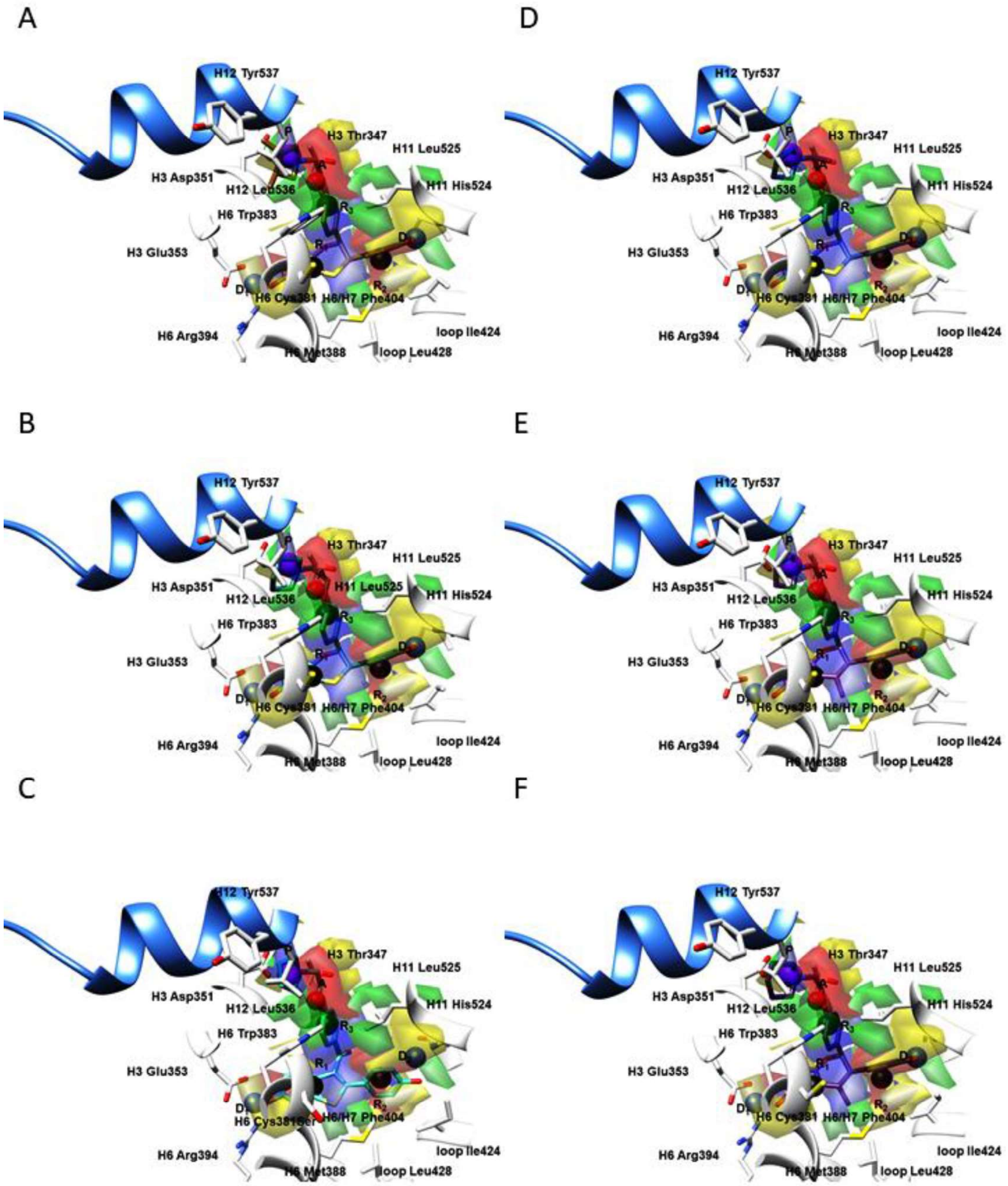

Figure S7. The **3-D PhypII** features (**D**: hydrogen-bond donators, **A**: hydrogen-bond acceptors, **R**: ring features, **P**: positive ionizable features) and **3-D QSAR** *PLS-coefficients* contour maps (**GREEN***PLS-coefficients*: positive steric interactions, **YELLOW***PLS-coefficients*: negative steric interactions, **BLUE***PLS-coefficients*: areas where positively charged functional groups and H-bond donators are favored whereas the negatively charged functional groups and H-bond acceptors are disfavoured, **RED***PLS-coefficients*: areas negatively charged functional groups and H-bond acceptors are favored, whereas the positively charged functional groups and H-bond donators are disfavored) for **1XP6** (A), **1SJ0** (B), **2R6Y** (C), **1XP9** (D), **1YIM** (E), and **1YIN** (F). Amino acid residues are depicted in white. For the clarity of presentation, only H12 helix is presented in cornflower blue ribbon, as a crucial delimiter for partial agonists, SERMs, and SERDs.

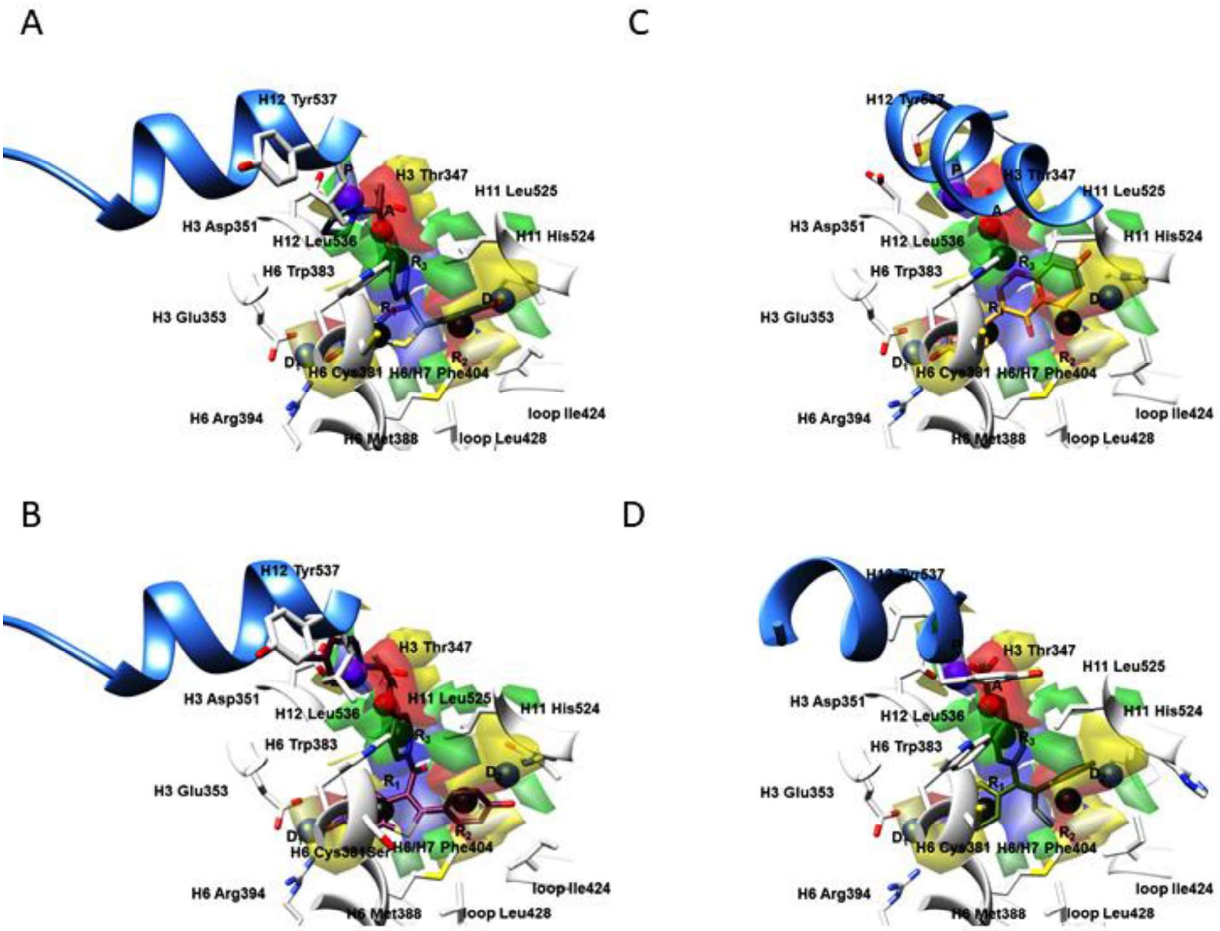

Figure S8. The **3-D PhypII** features (**D**: hydrogen-bond donators, **A**: hydrogen-bond acceptors, **R**: ring features, **P**: positive ionizable features) and **3-D QSAR** *PLS-coefficients* contour maps (**GREEN**$_{\mathit{PLS\text{-}coefficients}}$: positive steric interactions, **YELLOW**$_{\mathit{PLS\text{-}coefficients}}$: negative steric interactions, **BLUE**$_{\mathit{PLS\text{-}coefficients}}$: areas where positively charged functional groups and H-bond donators are favored whereas the negatively charged functional groups and H-bond acceptors are disfavoured, **RED**$_{\mathit{PLS\text{-}coefficients}}$: areas negatively charged functional groups and H-bond acceptors are favored, whereas the positively charged functional groups and H-bond donators are disfavored) for **1XPC** (A), **2R6W** (B), **2QA8** (C), and **1R5K** (D). Amino acid residues are depicted in white. For the clarity of presentation, only H12 helix is presented in cornflower blue ribbon, as a crucial delimiter for partial agonists, SERMs, and SERDs.

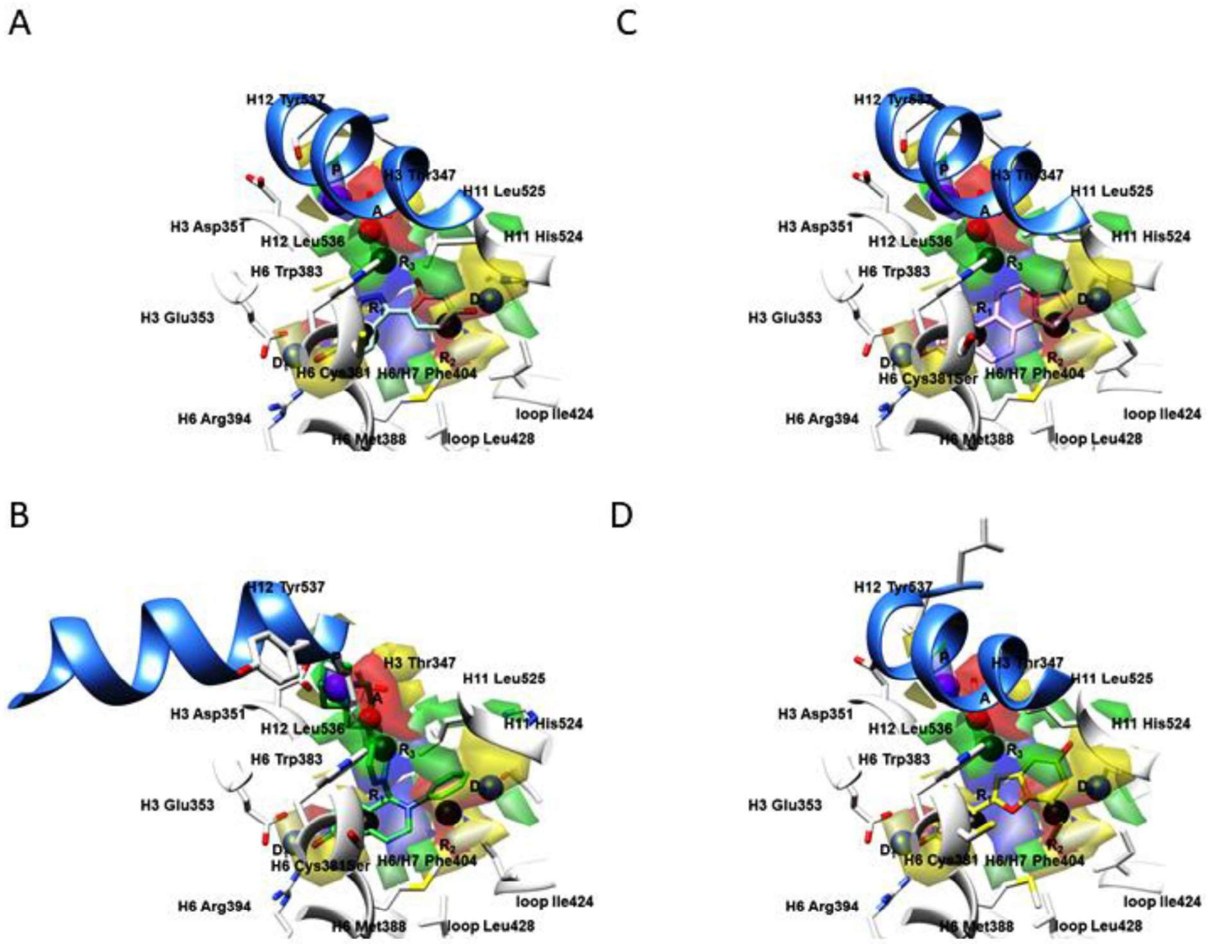

Figure S9. The **3-D PhypII** features (**D**: hydrogen-bond donators, **A**: hydrogen-bond acceptors, **R**: ring features, **P**: positive ionizable features) and **3-D QSAR** *PLS-coefficients* contour maps (**GREEN***PLS-coefficients*: positive steric interactions, **YELLOW***PLS-coefficients*: negative steric interactions, **BLUE***PLS-coefficients*: areas where positively charged functional groups and H-bond donators are favored whereas the negatively charged functional groups and H-bond acceptors are disfavoured, **RED***PLS-coefficients*: areas negatively charged functional groups and H-bond acceptors are favored, whereas the positively charged functional groups and H-bond donators are disfavored) for **2QA6** (A), **1XQC** (B), **2B1Z** (C), and **1X7E** (D). Amino acid residues are depicted in white. For the clarity of presentation, only H12 helix is presented in cornflower blue ribbon, as a crucial delimiter for partial agonists, SERMs, and SERDs.

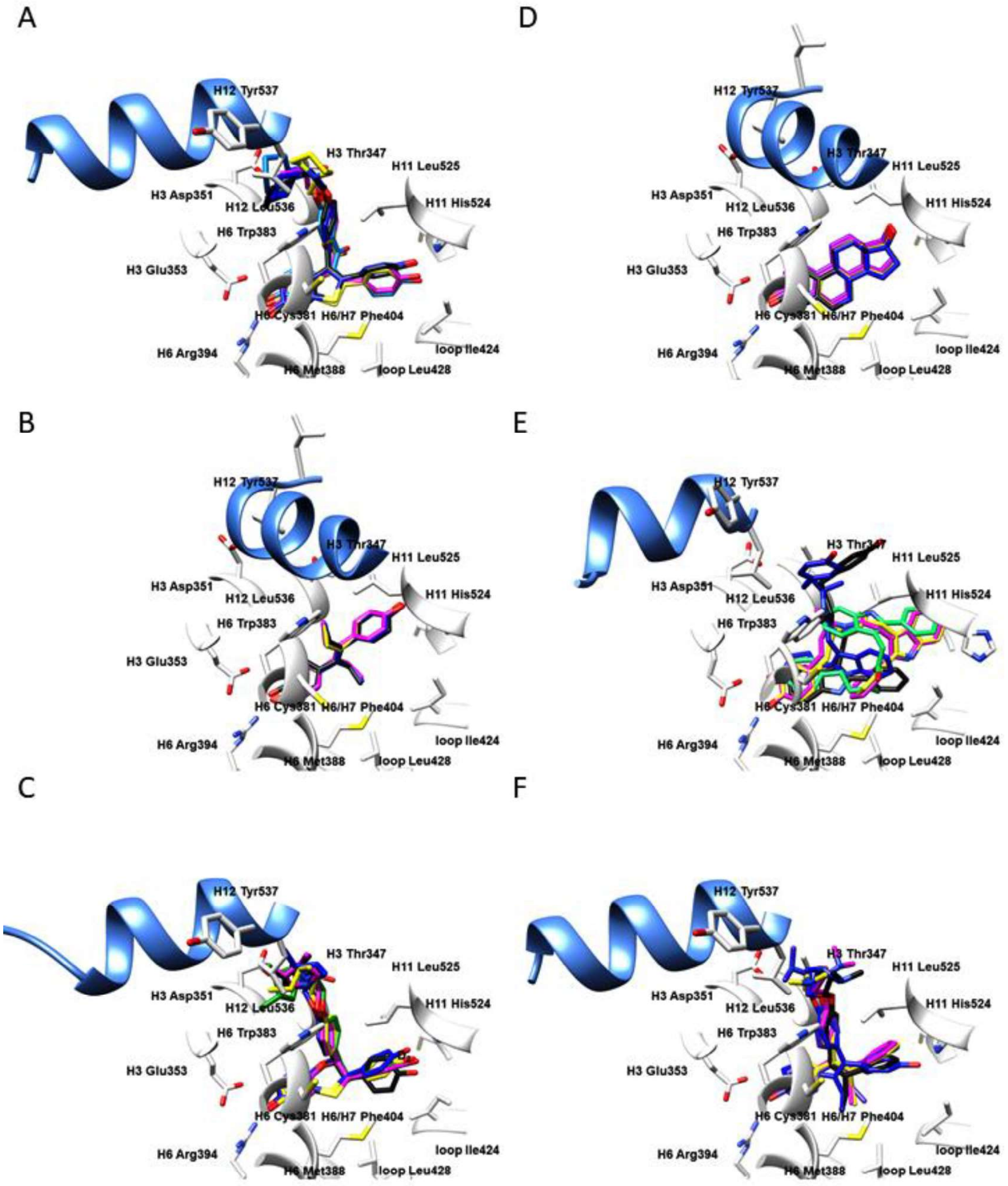

Figure S10. Structure-based alignment assessment of ERα partial agonists, SERMs, and SERDs using Glide's XP module: **1ERR** (A), EC blue, ECRD yellow, RCRD magenta, ECCD blue, RCCD black; **3ERD** (B), EC black, ECRD yellow, RCRD magenta, ECCD blue, RCCD black; **1XP1** (C), EC light green, ECRD yellow, RCRD magenta, ECCD blue, RCCD black; **1ERE** (D), EC violet, ECRD yellow, RCRD magenta, ECCD blue, RCCD black; **2IOK** (E), EC light green, ECRD yellow, RCRD magenta, ECCD blue, RCCD black; and **2BJ4** (F) EC purple, ECRD yellow, RCRD magenta, ECCD blue, RCCD black. Amino acid residues are depicted in white. For the clarity of presentation, only H12 helix is presented in cornflower blue ribbon, as a crucial delimiter for partial agonists, SERMs, and SERDs.

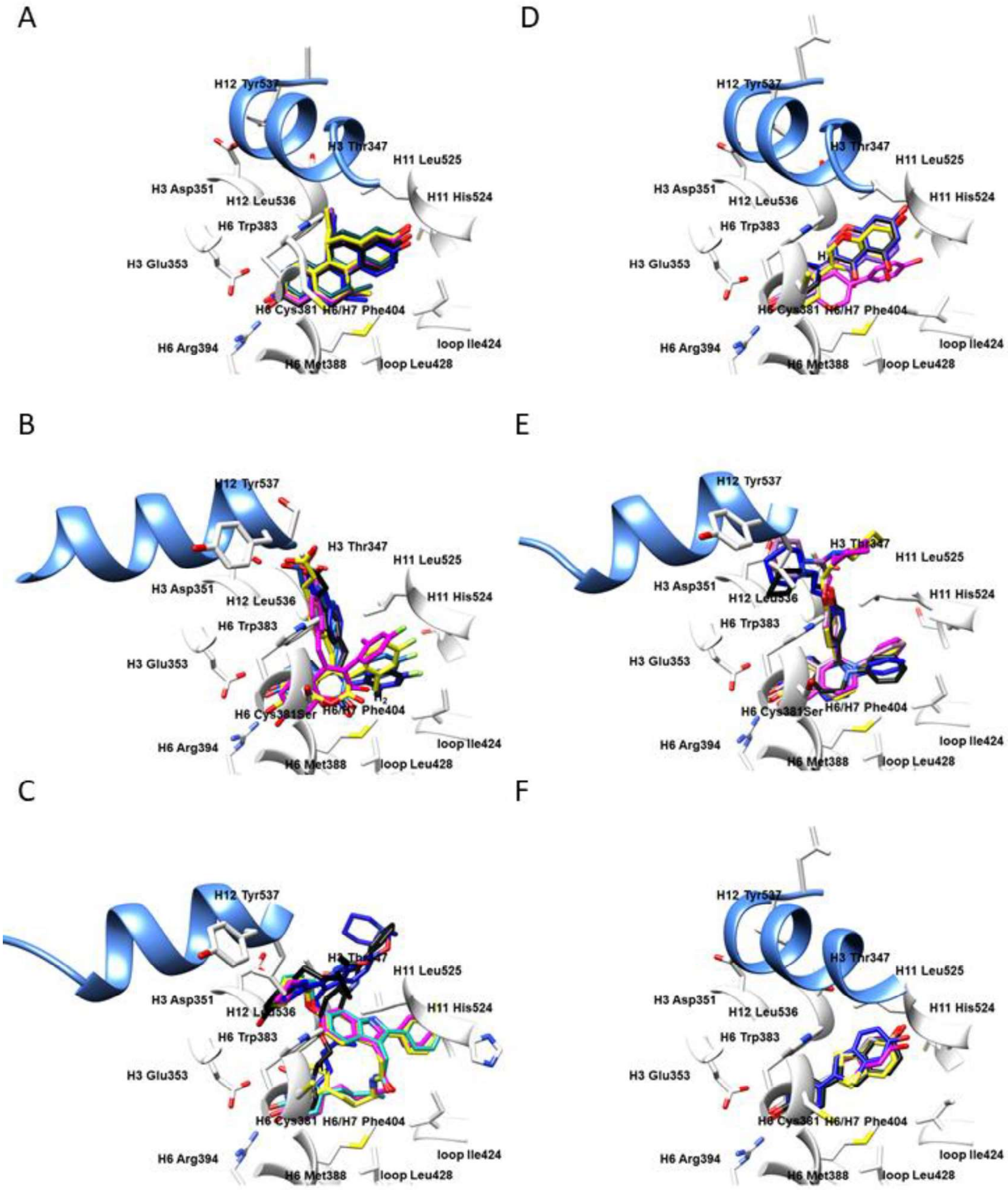

Figure S11. Structure-based alignment assessment of ERα partial agonists, SERMs, and SERDs using Glide's XP module: **1L2I** (A), EC dark blue, ECRD yellow, RCRD magenta, ECCD blue, RCCD black; **5AK2** (B), EC dark blue, ECRD yellow, RCRD magenta, ECCD blue, RCCD black; **2IOG** (C), EC dark green, ECRD yellow, RCRD magenta, ECCD blue, RCCD black; **1X7R** (D), EC purple, ECRD yellow, RCRD magenta, ECCD blue, RCCD black; **1UOM** (E), EC purple, ECRD yellow, RCRD magenta, ECCD blue, RCCD black; and **1GWQ** (F) EC gray, ECRD yellow, RCRD magenta, ECCD blue, RCCD black. Amino acid residues are depicted in white. For the clarity of presentation, only H12 helix is presented in cornflower blue ribbon, as a crucial delimiter for partial agonists, SERMs, and SERDs.

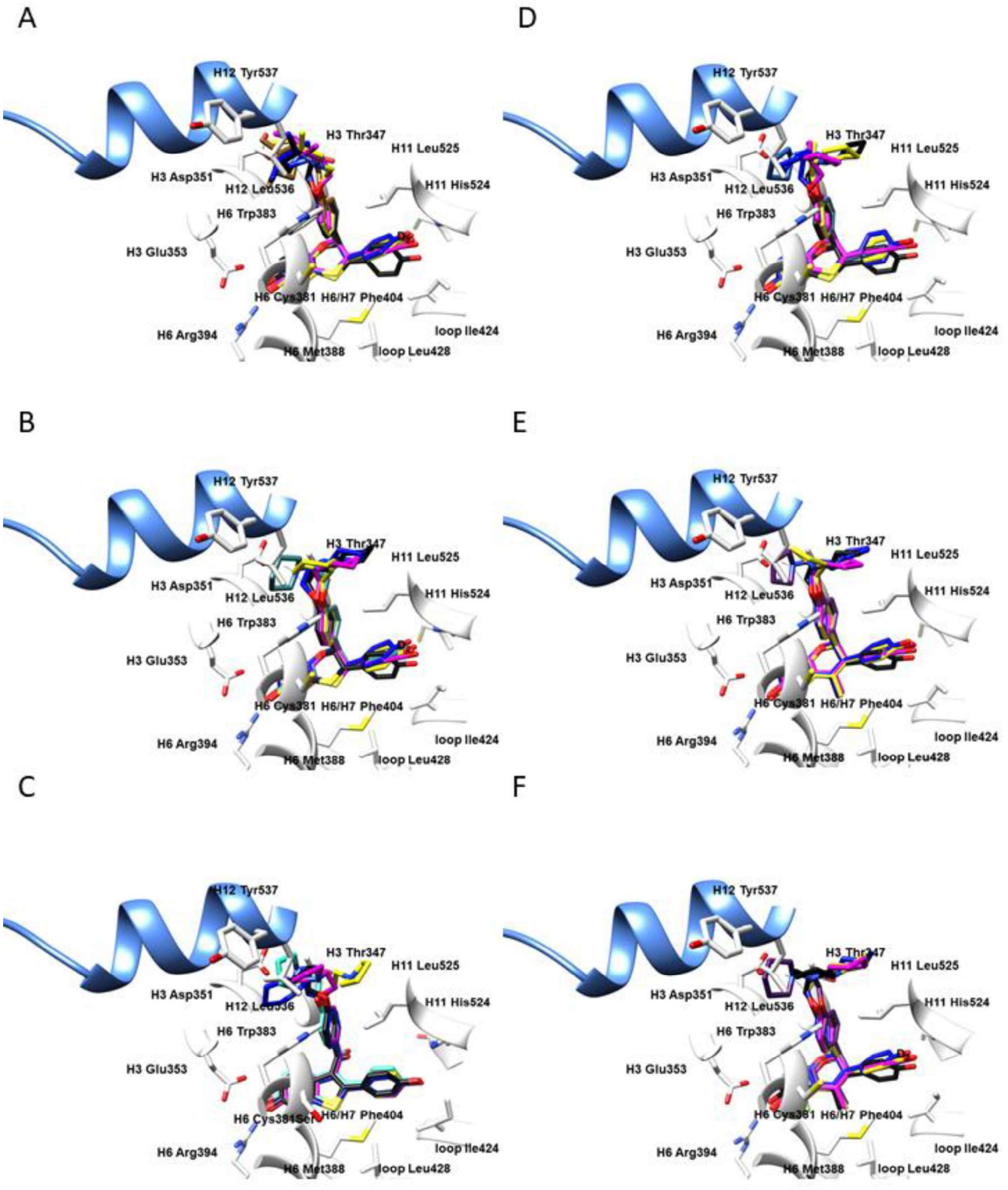

Figure S12. Structure-based alignment assessment of ERα partial agonists, SERMs, and SERDs using Glide's XP module: **1XP6** (A), EC brown, ECRD yellow, RCRD magenta, ECCD blue, RCCD black; **1SJ0** (B), EC dark blue, ECRD yellow, RCRD magenta, ECCD blue, RCCD black; **2R6Y** (C), EC light blue, ECRD yellow, RCRD magenta, ECCD blue, RCCD black; **1XP9** (D), EC blue, ECRD yellow, RCRD magenta, ECCD blue, RCCD black; **1YIM** (E), EC purple, ECRD yellow, RCRD magenta, ECCD blue, RCCD black; and **1YIN** (F) EC purple, ECRD yellow, RCRD magenta, ECCD blue, RCCD black. Amino acid residues are depicted in white. For the clarity of presentation, only H12 helix is presented in cornflower blue ribbon, as a crucial delimiter for partial agonists, SERMs, and SERDs.

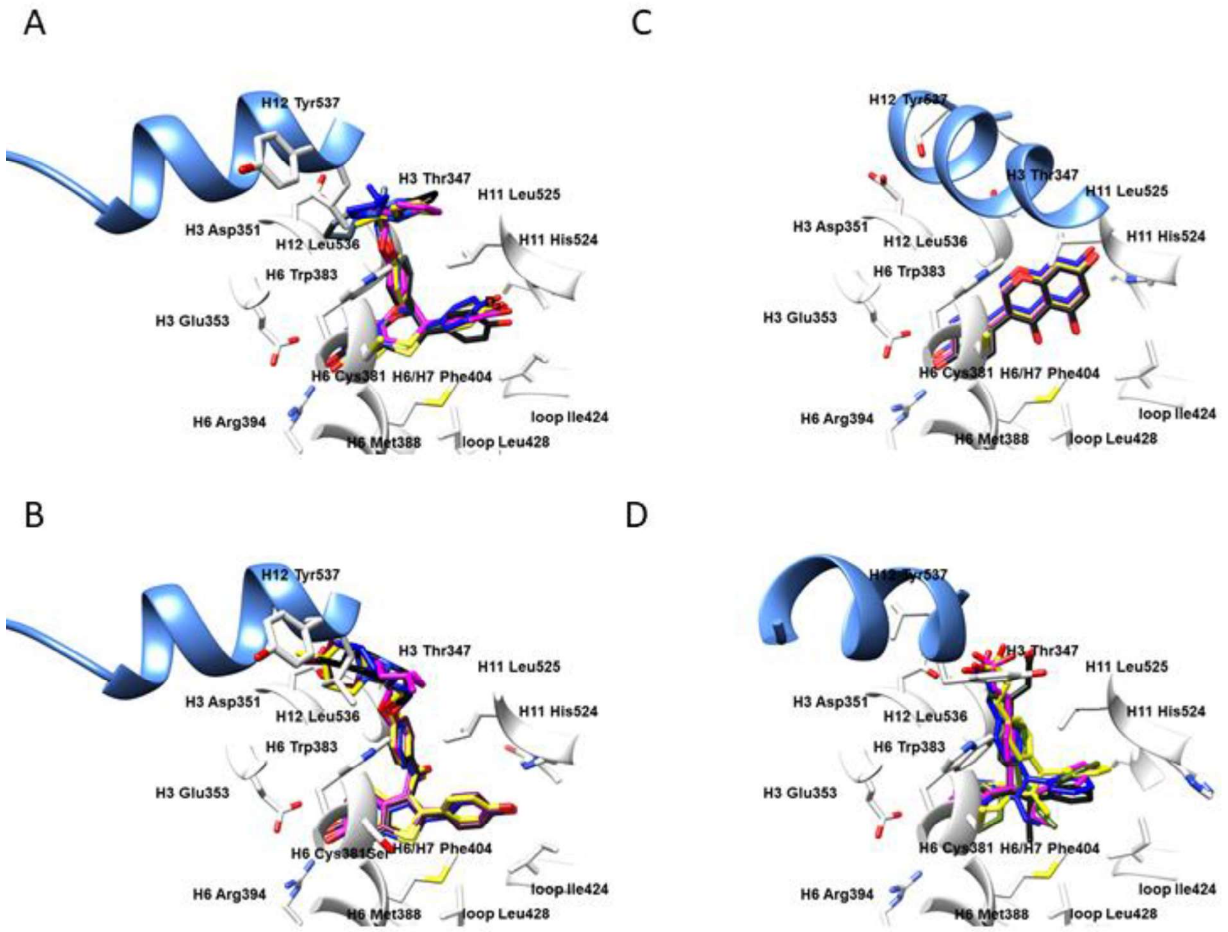

Figure S13. Structure-based alignment assessment of ERα partial agonists, SERMs, and SERDs using Glide's XP module: **1XPC** (A), EC gray, ECRD yellow, RCRD magenta, ECCD blue, RCCD black; **2R6W** (B), EC dark red, ECRD yellow, RCRD magenta, ECCD blue, RCCD black; **2QA8** (C), EC orange, ECRD yellow, RCRD magenta, ECCD blue, RCCD black; and **1R5K** (D) EC dark green, ECRD yellow, RCRD magenta, ECCD blue, RCCD black. Amino acid residues are depicted in white. For the clarity of presentation, only H12 helix is presented in cornflower blue ribbon, as a crucial delimiter for partial agonists, SERMs, and SERDs.

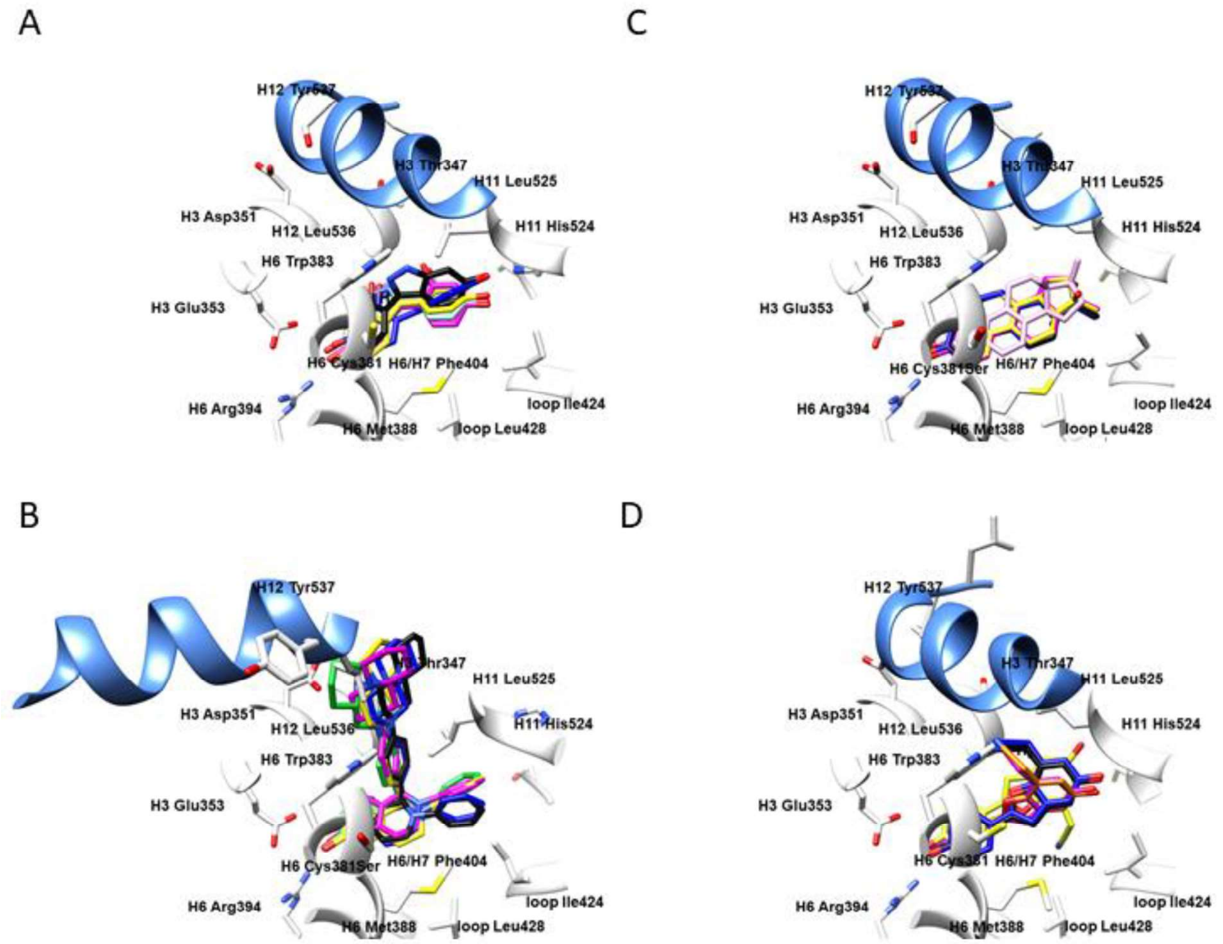

Figure S14. Structure-based alignment assessment of ERα partial agonists, SERMs, and SERDs using Glide's XP module: **2QA6** (A), EC light blue, ECRD yellow, RCRD magenta, ECCD blue, RCCD black; **1XQC** (B), EC light green, ECRD yellow, RCRD magenta, ECCD blue, RCCD black; **2B1Z** (C), EC light pink, ECRD yellow, RCRD magenta, ECCD blue, RCCD black; and **1X7E** (D) EC yellow, ECRD yellow, RCRD magenta, ECCD blue, RCCD black. Amino acid residues are depicted in white. For the clarity of presentation, only H12 helix is presented in cornflower blue ribbon, as a crucial delimiter for partial agonists, SERMs, and SERDs.

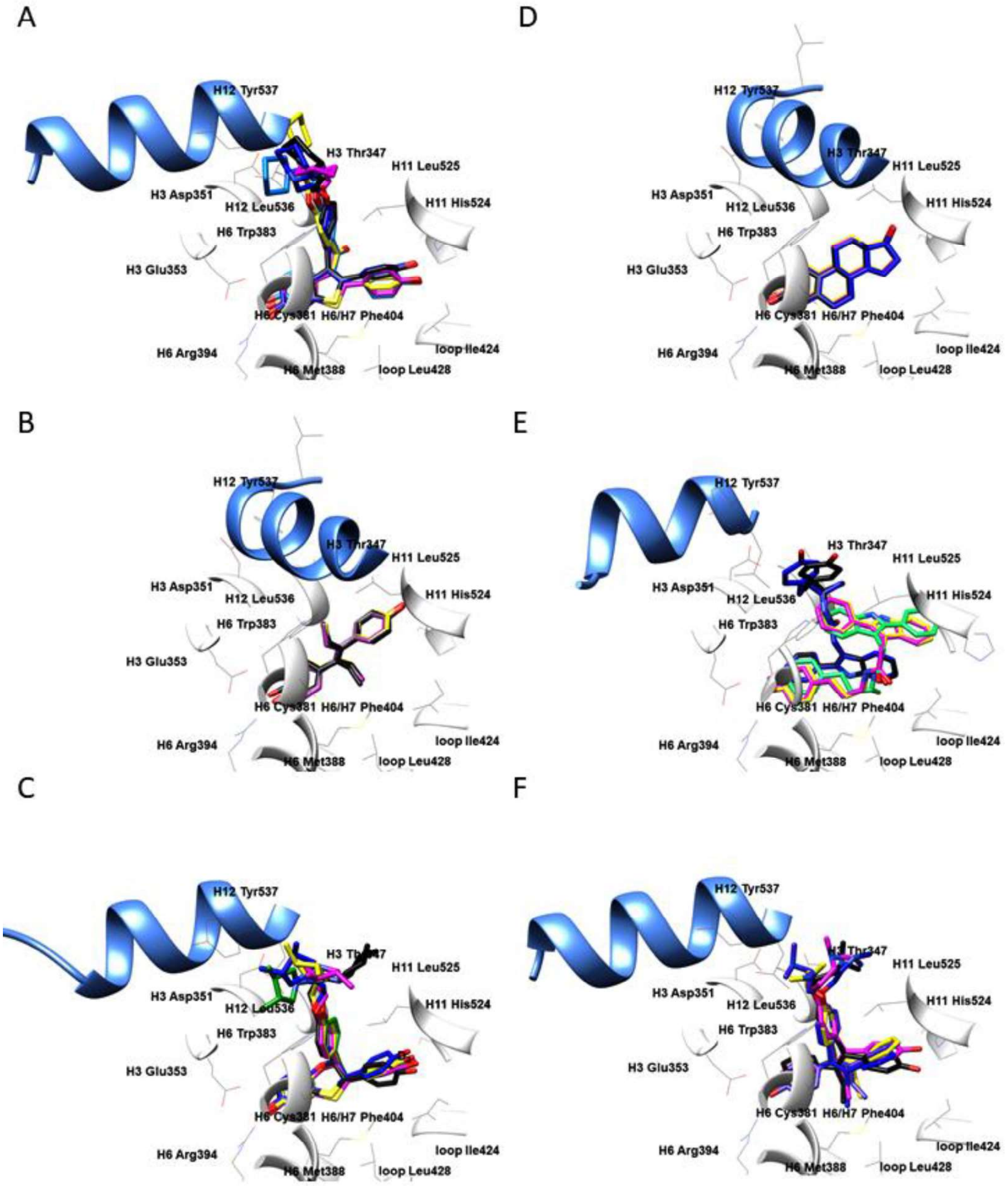

Figure S15. Ligand-based alignment assessment of ERα partial agonists, SERMs, and SERDs virtually superimposed to ERα active site using Flexible Ligand Alignment tool's FSBA module: **1ERR** (A), EC blue, ECRA yellow, RCRA magenta, ECCA blue, RCCA black; **3ERD** (B), EC black, ECRA yellow, RCRA magenta, ECCA blue, RCCA black; **1XP1** (C), EC light green, ECRA yellow, RCRA magenta, ECCA blue, RCCA black; **1ERE** (D), EC violet, ECRA yellow, RCRA magenta, ECCA blue, RCCA black; **2IOK** (E), EC light green, ECRA yellow, RCRA magenta, ECCA blue, RCCA black; and **2BJ4** (F) EC purple, ECRA yellow, RCRA magenta, ECCA blue, RCCA black. Amino acid residues are depicted in white. For the clarity of presentation, only H12 helix is presented in cornflower blue ribbon, as a crucial delimiter for partial agonists, SERMs, and SERDs.

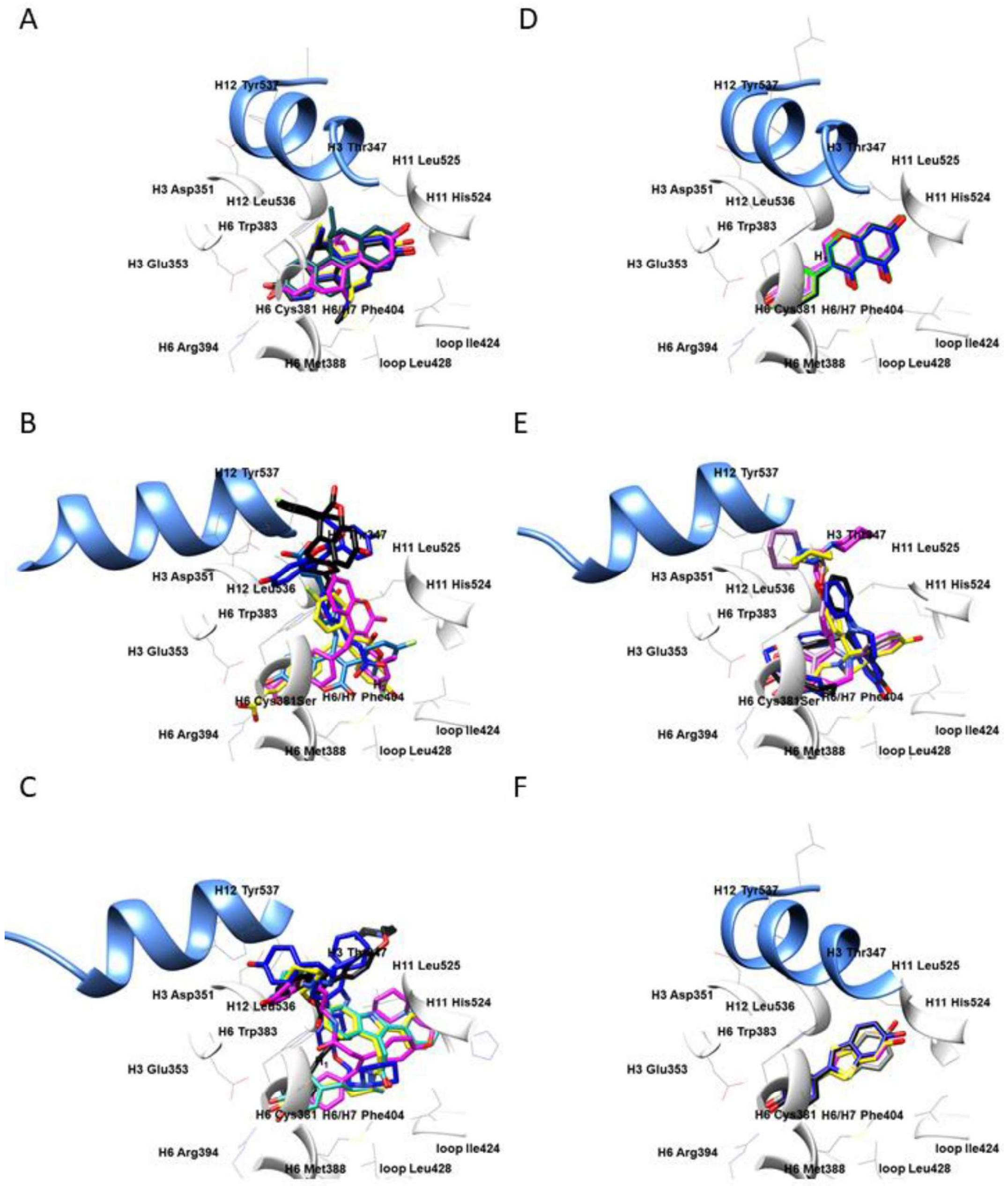

Figure 16. Ligand-based alignment assessment of ERα partial agonists, SERMs, and SERDs virtually superimposed to ERα active site using Flexible Ligand Alignment tool's FSBA module: **1L2I** (A), EC dark blue, ECRA yellow, RCRA magenta, ECCA blue, RCCA black; **5AK2** (B), EC dark blue, ECRA yellow, RCRA magenta, ECCA blue, RCCA black; **2IOG** (C), EC dark green, ECRA yellow, RCRA magenta, ECCA blue, RCCA black; **1X7R** (D), EC purple, ECRA yellow, RCRA magenta, ECCA blue, RCCA black; **1UOM** (E), EC purple, ECRA yellow, RCRA magenta, ECCA blue, RCCA black; and **1GWQ** (F) EC gray, ECRA yellow, RCRA magenta, ECCA blue, RCCA black. Amino acid residues are depicted in white. For the clarity of presentation, only H12 helix is presented in cornflower blue ribbon, as a crucial delimiter for partial agonists, SERMs, and SERDs.

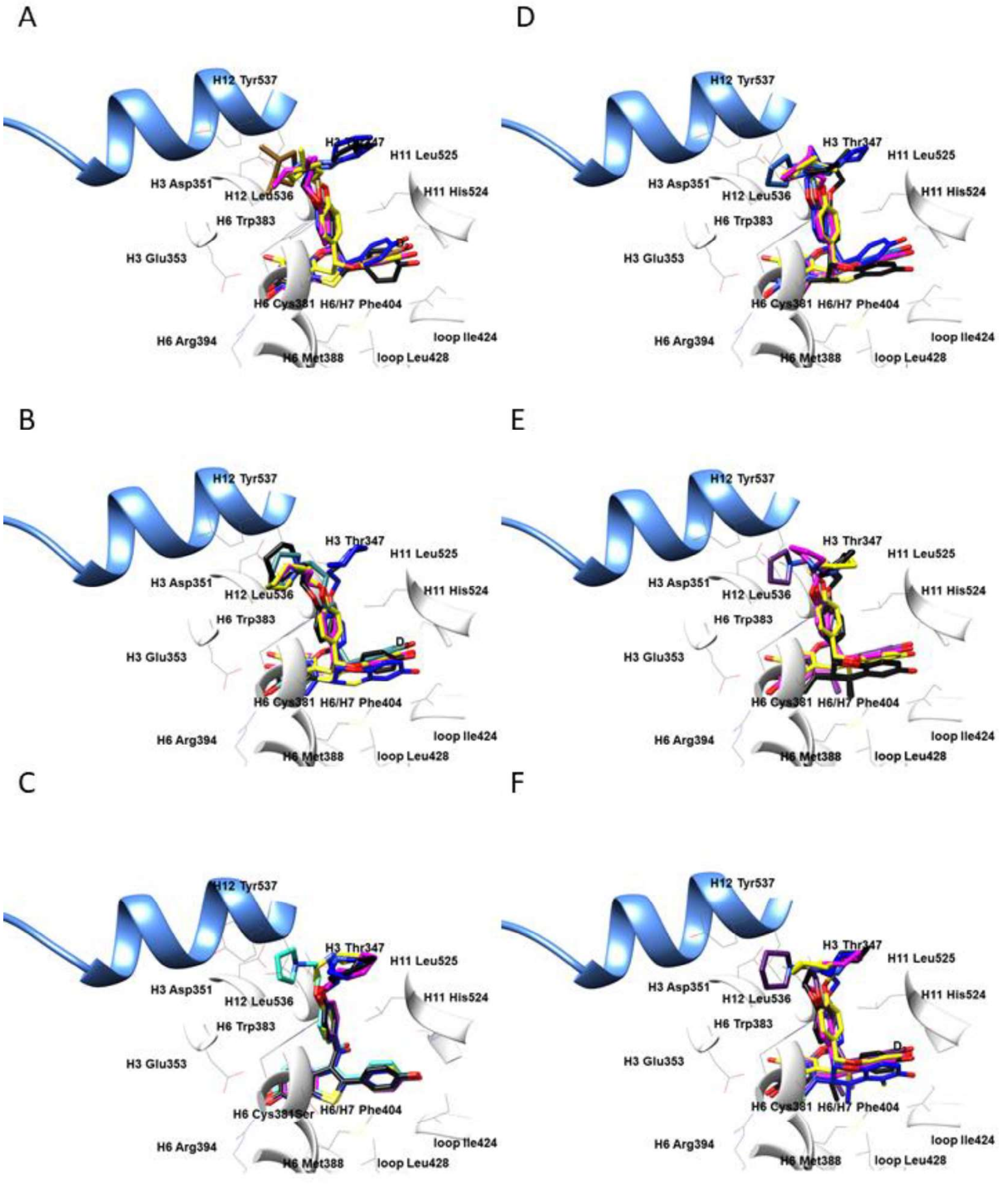

Figure S17. Ligand-based alignment assessment of ERα partial agonists, SERMs, and SERDs virtually superimposed to ERα active site using Flexible Ligand Alignment tool's FSBA module: **1XP6** (A), EC brown, ECRA yellow, RCRA magenta, ECCA blue, RCCA black; **1SJ0** (B), EC dark blue, ECRA yellow, RCRA magenta, ECCA blue, RCCA black; **2R6Y** (C), EC light blue, ECRA yellow, RCRA magenta, ECCA blue, RCCA black; **1XP9** (D), EC blue, ECRA yellow, RCRA magenta, ECCA blue, RCCA black; **1YIM** (E), EC purple, ECRA yellow, RCRA magenta, ECCA blue, RCCA black; and **1YIN** (F) EC purple, ECRA yellow, RCRA magenta, ECCA blue, RCCA black. Amino acid residues are depicted in white. For the clarity of presentation, only H12 helix is presented in cornflower blue ribbon, as a crucial delimiter for partial agonists, SERMs, and SERDs.

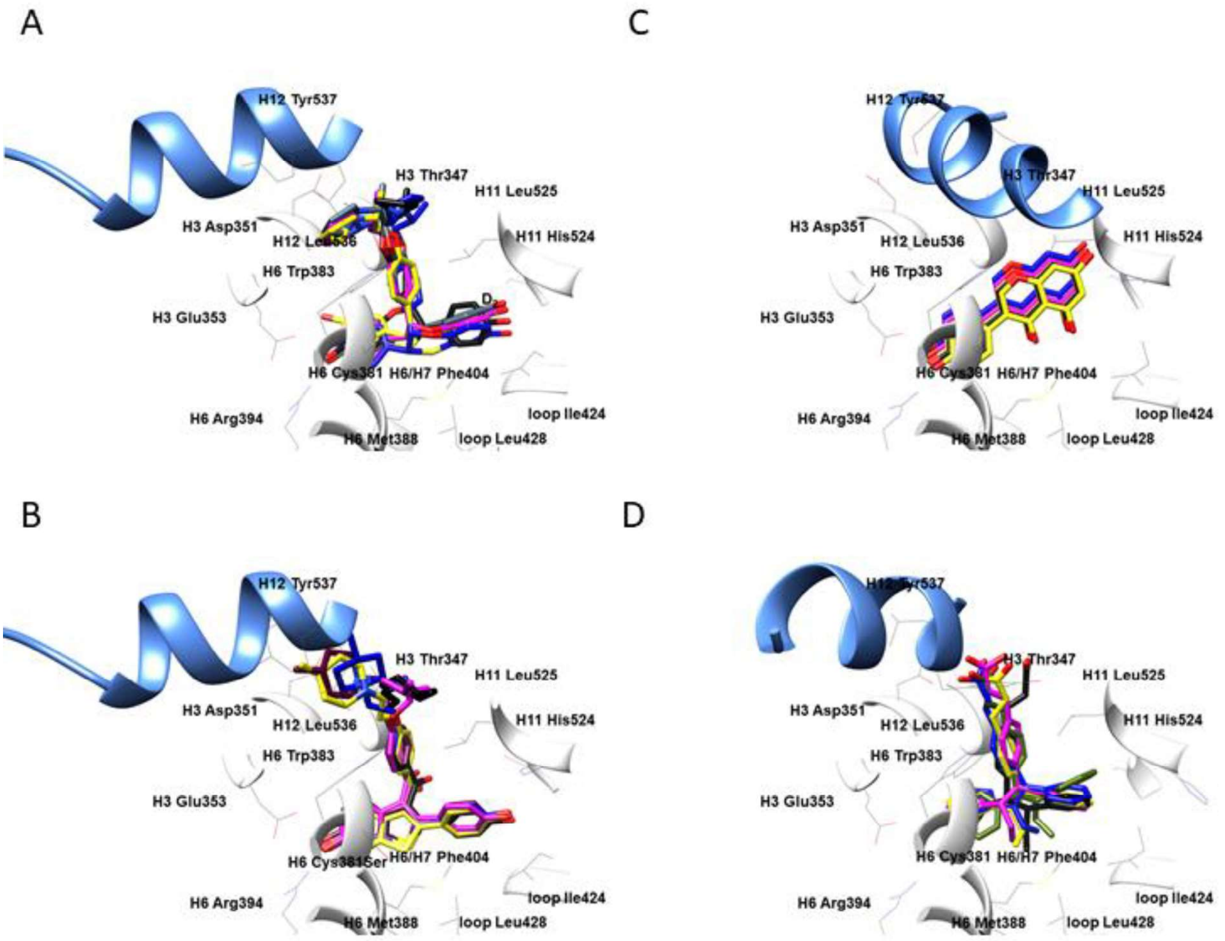

Figure S18. Ligand-based alignment assessment of ERα partial agonists, SERMs, and SERDs virtually superimposed to ERα active site using Flexible Ligand Alignment tool's FSBA module: **1XPC** (A), EC gray, ECRA yellow, RCRA magenta, ECCA blue, RCCA black; **2R6W** (B), EC dark red, ECRA yellow, RCRA magenta, ECCA blue, RCCA black; **2QA8** (C), EC orange, ECRA yellow, RCRA magenta, ECCA blue, RCCA black; and **1R5K** (D) EC dark green ECRA yellow, RCRA magenta, ECCA blue, RCCA black. Amino acid residues are depicted in white. For the clarity of presentation, only H12 helix is presented in cornflower blue ribbon, as a crucial delimiter for partial agonists, SERMs, and SERDs.

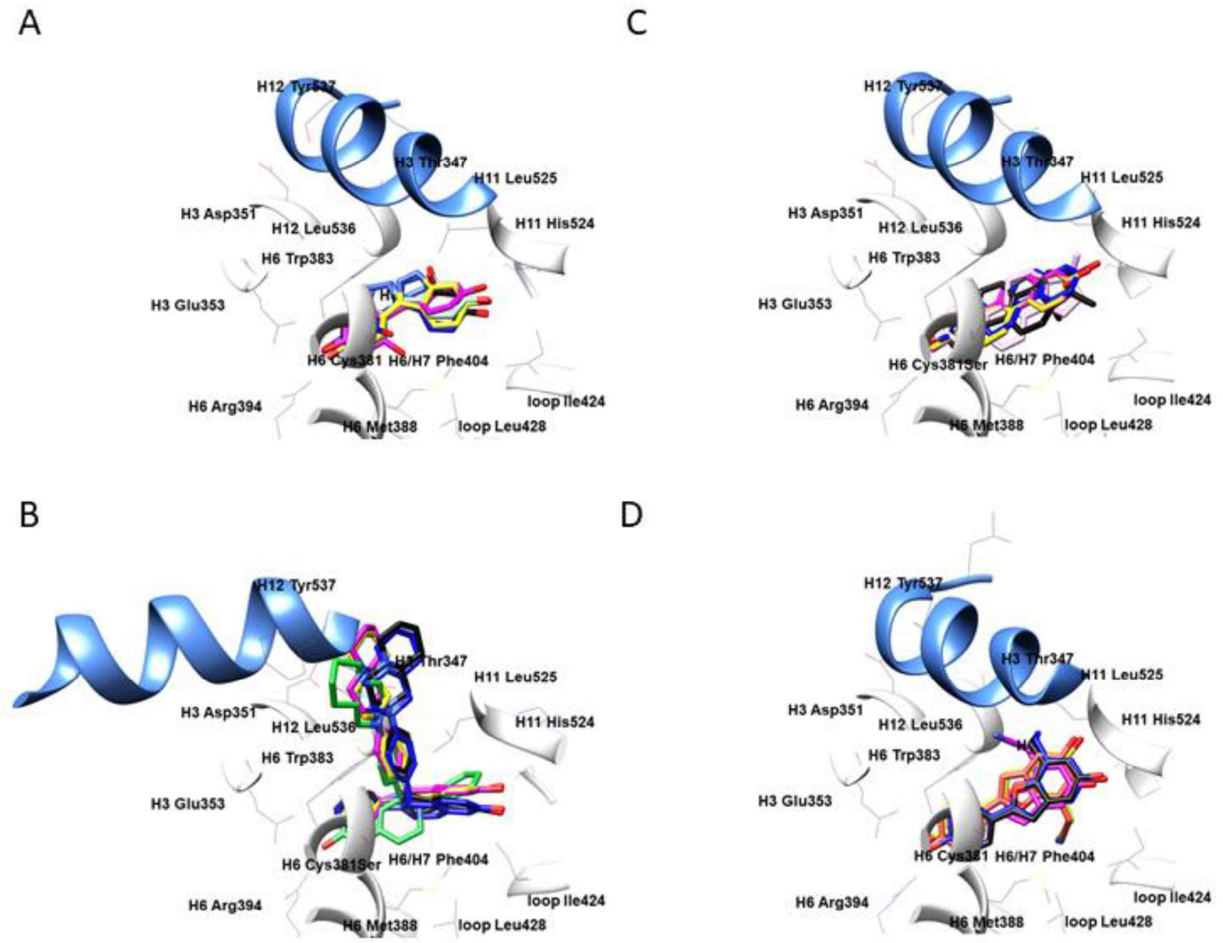

Figure S19. Ligand-based alignment assessment of ERα partial agonists, SERMs, and SERDs virtually superimposed to ERα active site using Flexible Ligand Alignment tool's FSBA module: **2QA6** (A), EC light blue, ECRA yellow, RCRA magenta, ECCA blue, RCCA black; **1XQC** (B), EC light green, ECRA yellow, RCRA magenta, ECCA blue, RCCA black; **2B1Z** (C), EC light pink, ECRA yellow, RCRA magenta, ECCA blue, RCCA black; and **1X7E** (D) EC yellow ECRA yellow, RCRA magenta, ECCA blue, RCCA black. Amino acid residues are depicted in white. For the clarity of presentation, only H12 helix is presented in cornflower blue ribbon, as a crucial delimiter for partial agonists, SERMs, and SERDs.

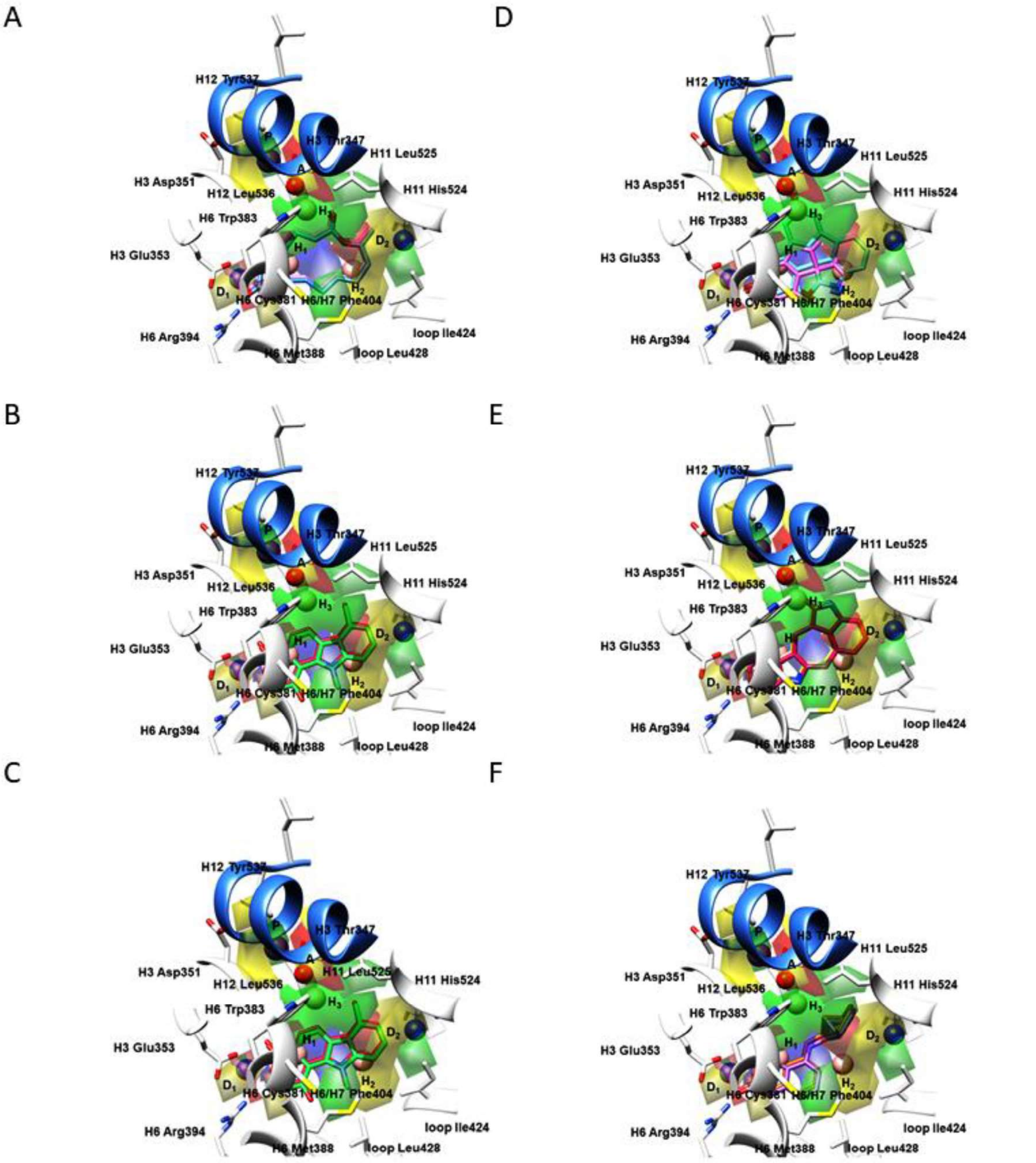

Figure 20. The SB/LB virtually screened conformations of **NCI89671**, SB conformation blue, LB conformation pink (A); **NCI101789**, SB conformation light green, LB conformation purple (B); **NCI645330**, SB conformation red, LB conformation green (C); **NCI59814**, SB conformation light blue, LB conformation pink (D); **NCI76747**, SB conformation pink, LB conformation yellow (E); **NCI9782**, SB conformation purple, LB conformation orange (F), incorporated into the **3-D PhypI/3-D QSAR model** prediction engine**.** Amino acid residues are depicted in white. For the clarity of presentation, only H12 helix is presented in a cornflower blue ribbon, as a crucial delimiter for partial agonists, SERMs, and SERDs.

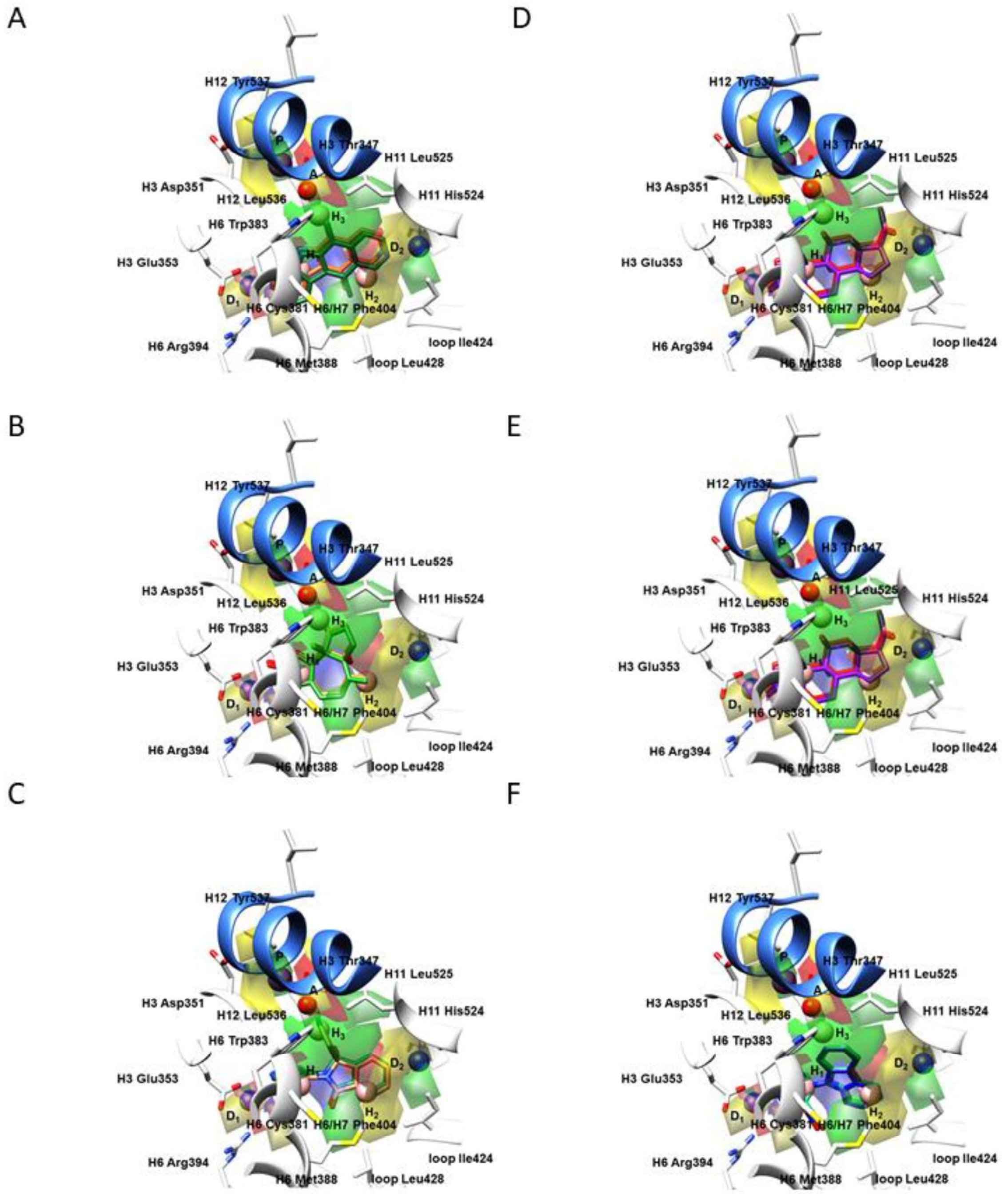

Figure S21. The SB/LB virtually screened conformations of **NCI71795** SB conformation green, LB conformation orange (A); **NCI85239**, SB conformation green, LB conformation yellow (B); **NCI69359**, SB conformation coral, LB conformation green (C); **NCI16437**, SB conformation green, LB conformation blue (D); **NCI17128**, SB conformation purple, LB conformation red (E); and **NCI10211**, SB conformation green, LB conformation blue (F), incorporated into the **3-D PhypI/3-D QSAR model** prediction engine. Amino acid residues are depicted in white. For the clarity of presentation, only H12 helix is presented in a cornflower blue ribbon, as a crucial delimiter for partial agonists, SERMs, and SERDs.

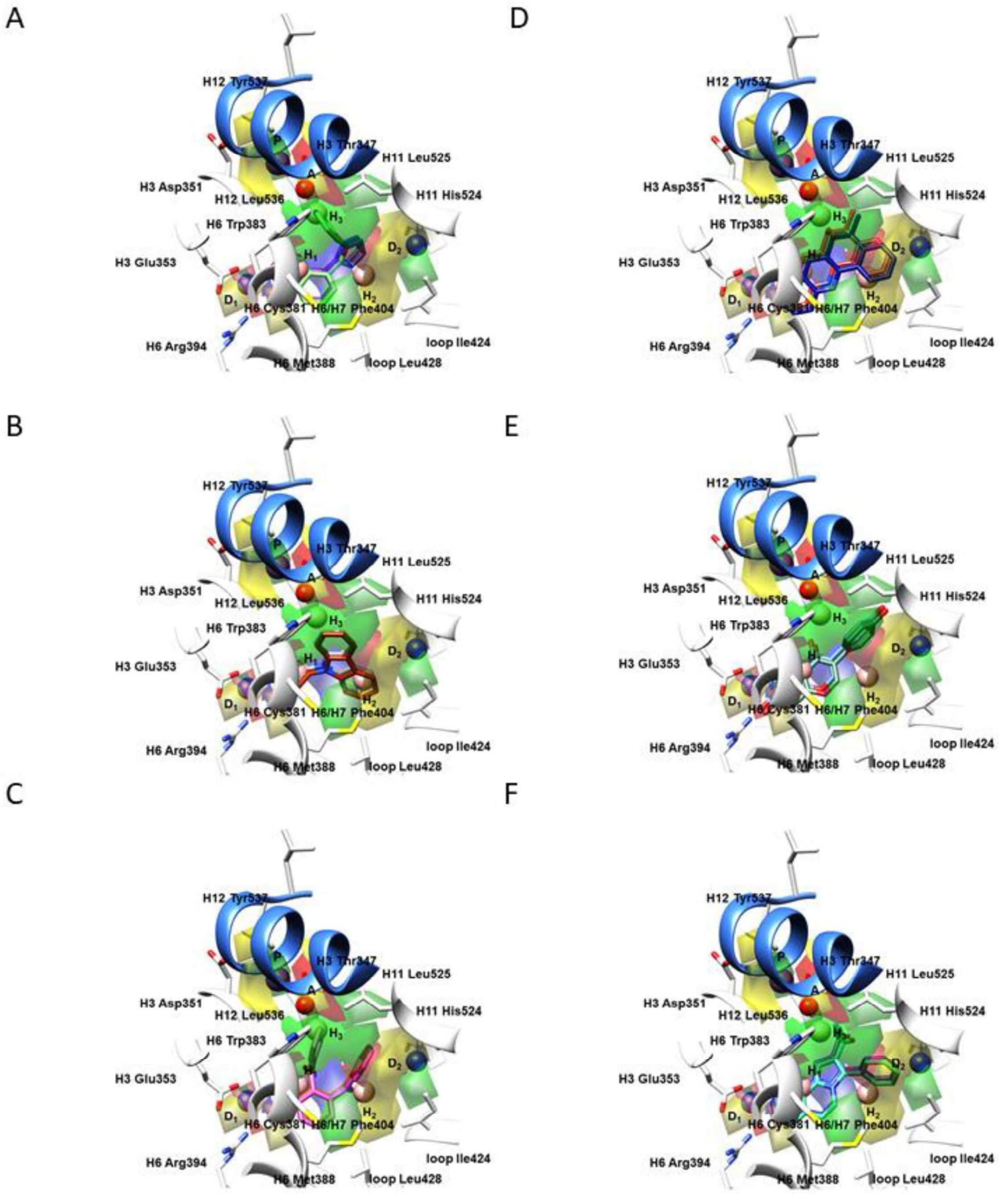

Figure S22. The SB/LB virtually screened conformations of **NCI30813**, SB conformation blue, LB conformation red (A); **NCI10416**, SB conformation blue, LB conformation red (B); **NCI6145**, SB conformation blue, LB conformation red (C); **NCI19824**, SB conformation red, LB conformation blue (D); **NCI36586**, SB conformation blue, LB conformation red (E); and **NCI93427**, SB conformation blue, LB conformation red (F), incorporated into the **3-D PhypI/3-D QSAR model** prediction engine. Amino acid residues are depicted in white. For the clarity of presentation, only H12 helix is presented in a cornflower blue ribbon, as a crucial delimiter for partial agonists, SERMs, and SERDs.

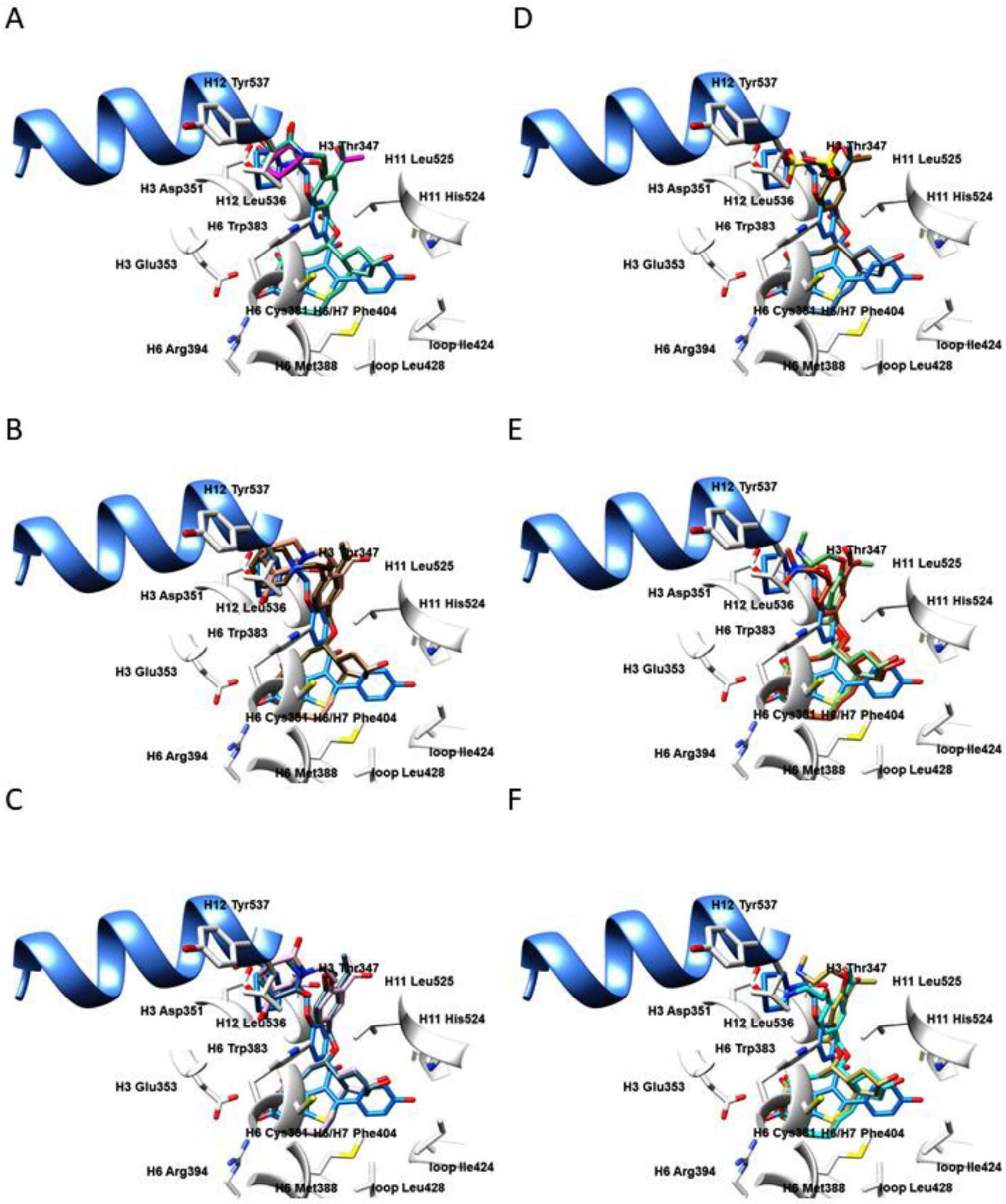

Figure S23. The superimposition between the designed compounds **3DPQ-12** (A), SBC light green-blue, LBC pink; **3DPQ-3** (B), SBC brown, LBC orchid; **3DPQ-9** (C), SBC dark green-blue, LBC pink; **3DPQ-4** (D), SBC blue, LBC brown; **3DPQ-2** (E), SBC red, LBC green; **3DPQ-1** (F). SBC blue, LBC yellow-green; and **1ERE**, EC blue, within the ERα active site. Amino acid residues are depicted in white. For the clarity of presentation, only H12 helix is presented in cornflower blue ribbon, as a crucial delimiter for partial agonists, SERMs, and SERDs.

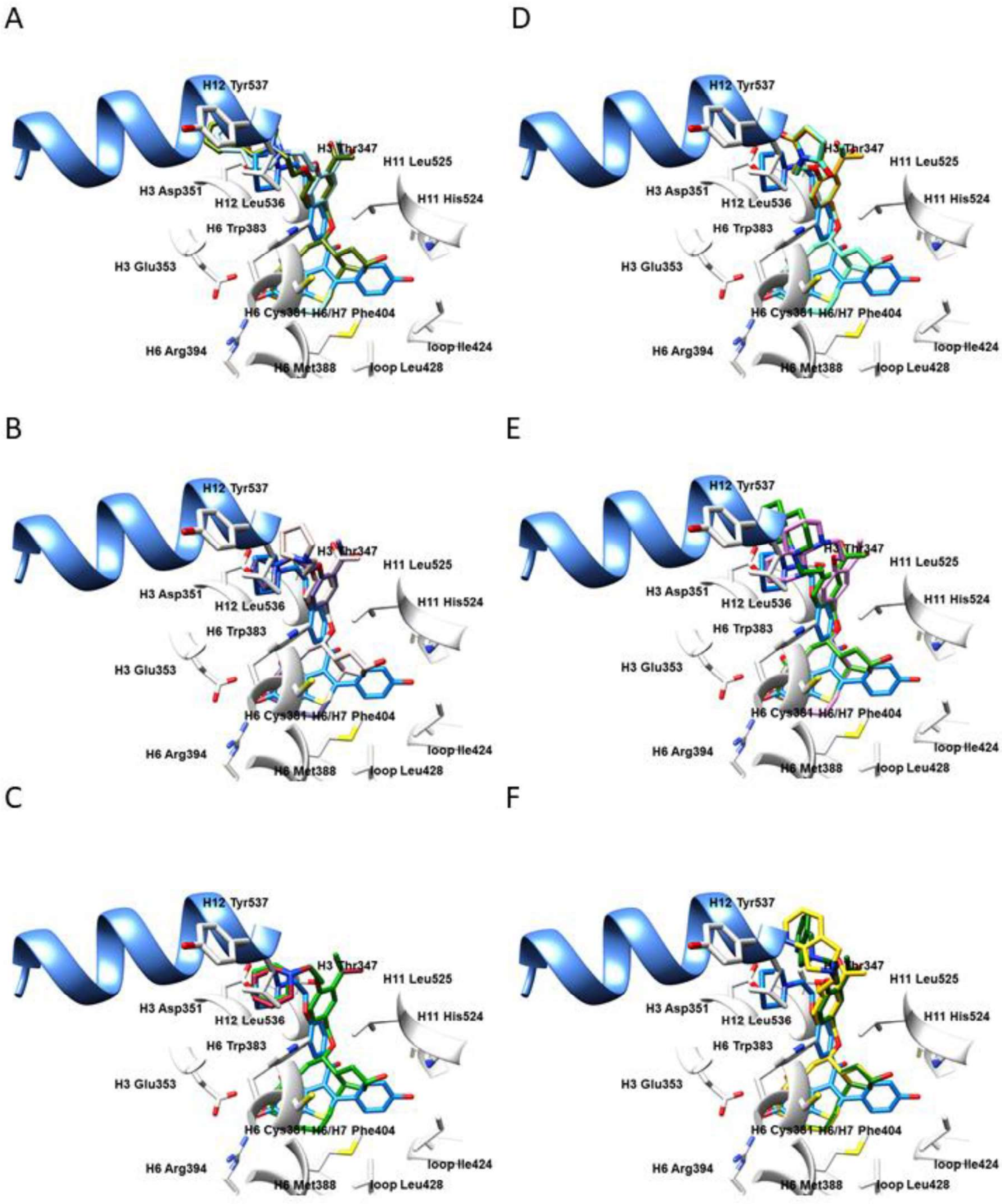

Figure S24. The superimposition between the designed compounds **3DPQ-7** (A), SBC blue, LBC green; **3DPQ-11** (B), SBC purple, LBC pink; **3DPQ-10** (C), SBC green, LBC red; **3DPQ-5** (D), SBC blue, LBC orange; **3DPQ-8** (E), SBC pink, LBC green; **3DPQ-6** (F). SBC green, LBC yellow; and **1ERE**, EC blue, within the ERα active site. Amino acid residues are depicted in white. For the clarity of presentation, only H12 helix is presented in cornflower blue ribbon, as a crucial delimiter for partial agonists, SERMs, and SERDs.

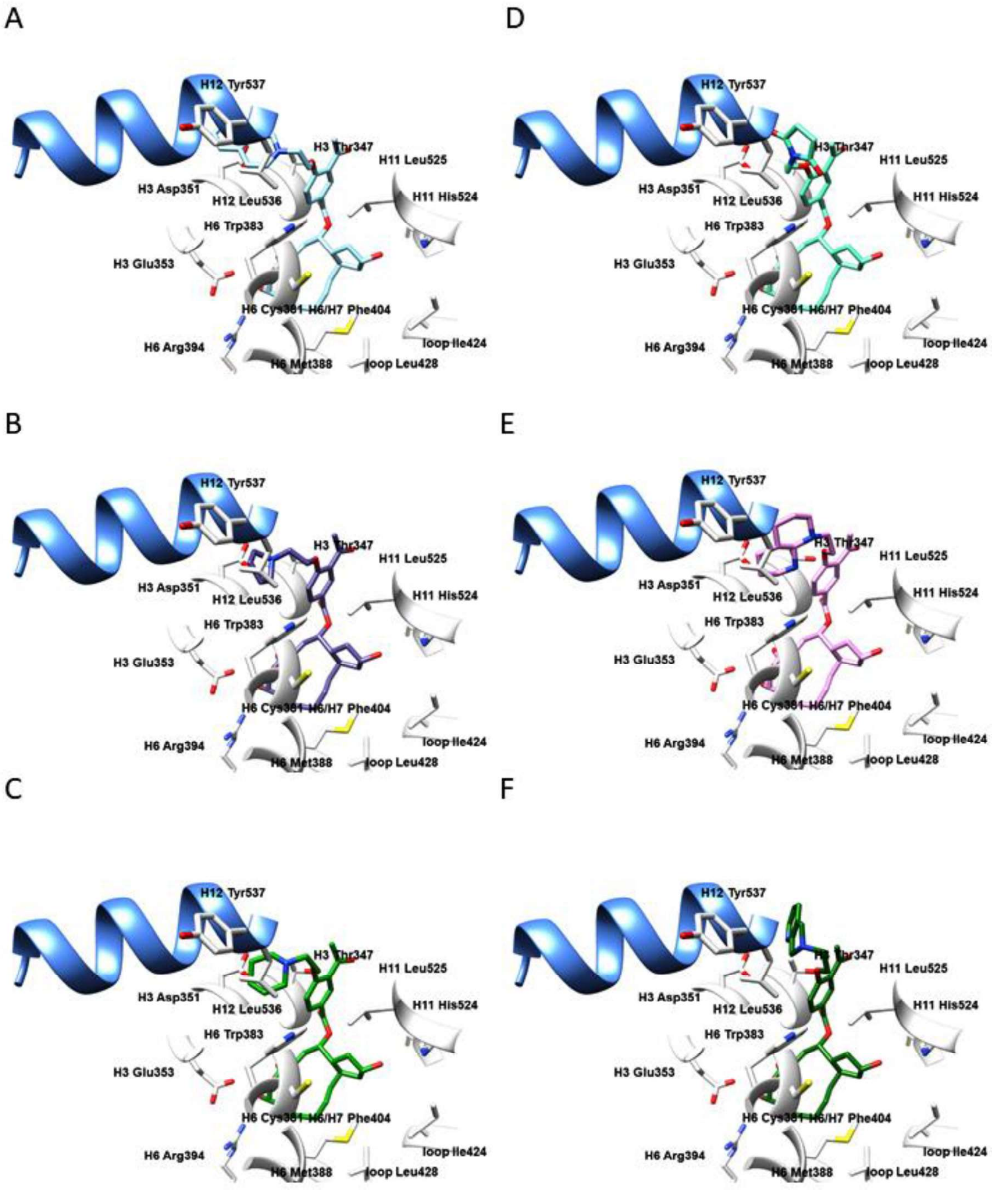

Figure S25. The bioactive conformations of **3DPQ-7** (A); **3DPQ-11** (B); **3DPQ-10** (C); **3DPQ-5** (D); **3DPQ-8** (E); **3DPQ-6** (F) within the ERα active site. Amino acid residues are depicted in white, H12 helix is presented in cornflower blue ribbon.

Synthesized compounds spectral data interpretation.

Using the modified synthetic approach (Schemes 1), designed compounds **3DPQ-1** to **3DPQ-12** were produced in high yields. The spectral data (^{1}H NMR, ^{13}C NMR, ^{15}N NMR, ^{17}O NMR, and GC/MS), were recorded in DMSO-d6 as a solvent, and the elemental analysis of synthesized compounds were in full agreement with the suggested molecular structures. Hence, the synthesis of **R1** (Supplementary Materials Figures S26-S28) had been characterized by the introduction of the TBS protective group onto the terminal hydroxyl group of 5-hydroxypentanal. Thus, the hydroxyl group protection was confirmed by the appearance of TBS methyl groups as high-intensity ^{1}H NMR multiplets at 0.14 and 1.05 ppm (Supplementary Materials Figure S26), as well as by ^{13}C NMR multiplet at -3.21 ppm, assigned to methyl groups directly attached to a Si atom, and multiplet at 25.53 ppm for *tert*-butyl group (Supplementary Materials Figure S27). The free aldehyde group was confirmed by a low-intensity ^{1}H NMR singlet at 9.65 ppm (Supplementary Materials Figure S26) and ^{13}C NMR singlet at 198.49 ppm (Supplementary Materials Figure S27). The methylene group substituted by the -O-TBS scaffold was recognized as ^{1}H NMR multiplet at 3.74 ppm (Supplementary Materials Figure S26) and ^{13}C NMR singlet at 63.02 ppm (Supplementary Materials Figure S27), whereas the oxygen atom was evident at ^{17}O NMR's singlet at 6.00 ppm (Supplementary Materials Figure S28). The methyl group vicinal to aldehyde function was observed as ^{1}H NMR multiplet at 2.46 ppm (Supplementary Materials Figure S26) and ^{13}C NMR singlet at 36.09 ppm (Supplementary Materials Figure S27), whereas the linking ethylene portion was evident at 1H NMR 1.54 and 1.59 ppm multiplets (Supplementary Materials Figure S26) and ^{13}C NMR singlet at 22.78 ppm of 25.53 ppm multiplet and 30.03 ppm singlet (Supplementary Materials Figure S27), respectively.

The reduction of an aldehyde group carbonyl portion towards the hydroxyl one within the **R2** was confirmed by the ^{1}H NMR singlet at 4.24 ppm (Supplementary Materials Figure S29) and ^{17}O NMR singlet at 44.50 ppm (Supplementary Materials Figure S31). Furthermore, the substitution of former aldehyde hydrogen with the methoxy group in **R2**, further brefeldin A C15 methoxy portion, was validated by the appearance of the methyl group multiplet in the ^{1}H NMR spectrum at 3.33 ppm (Supplementary Materials Figure S29) and singlet within the ^{13}C NMR spectrum at 53.07 ppm (Supplementary Materials Figure S30), as well as by the existence of the methoxy group oxygen atom as ^{17}O NMR singlet at 23.00 ppm (Supplementary Materials Figure S31).

The further transformation was a strip of TBS protective group of **R2**'s terminal hydroxyl group, which was within the **R3** confirmed with the appearance of the new hydroxyl portion at ^{1}H NMR's 3.80 ppm (Supplementary Materials Figure S32) as a moderate-intensity singlet, sharp singlet at ^{17}O NMR's at -6.00 ppm (Supplementary Materials Figure S34), and the absence of TBS methyl groups within the ^{13}C NMR spectrum (Supplementary Material Figures S33).

The incorporation of 1-phenyl-1*H*-tetrazole-5-thiol and protection of the secondary hydroxyl group gave the sulfide **R4**. Thus, distinct reactions were at once confirmed by the absence of the terminal hydroxyl group within the ^{1}H NMR spectrum and the existence of the phenyl group multiplets at ^{1}H NMR's 7.12, 7.31, and 7.81 ppm (Supplementary Materials Figure S35) and ^{13}C NMR's 124.93, 129.59, and 134.73 ppm (Supplementary Materials Figure S36). The incorporation of 1*H*-tetrazole was obvious after the ^{15}N NMR singlets at -148.10 (the *N*-Phe signal), -100.50, -30.50, and 5.10 ppm (the sp^2 hybridized nitrogen signals, respectively) have been recorded (Supplementary Materials Figure S37). The carbon single bonded to sulfur and double bonded to nitrogen was seen as ^{13}C NMR singlet at 157.35 ppm (Supplementary Materials Figure S36), thus indirectly confirming the incorporation of the sulfur atom. Moreover, there was an absence of hydroxyl group signals originating from the reduced carbonyl portion; the methyl groups belonging to the TBS were evident and the appearance of the ^{13}C NMR multiplets at -3.09 and 25.66 ppm (Supplementary Materials Figure S35) and ^{17}O NMR singlet at 16.80 ppm (Supplementary Materials Figure S38) indicated that TBS indeed protected different hydroxyl group compared to **R2**.

The oxidation of **R4** furnished the **R5** (Supplementary Materials Figures S39-S42), leading to the sulfuric acid fragment recognized by the existence of the ^{17}O NMR multiplet at 147.00 ppm (Supplementary Materials Figure S42).

The reagent **R6** was prepared according to the procedure described elsewhere and its structure (Supplementary Materials Figures S43-S46) was in agreement with previous findings. As the **R6** was prepared from Brefeldin A (BFA), the common nomenclature for BFA will be used for the description of the **R6** structure. Thus, the carbonyl group at position C1 was confirmed by a ^{13}C NMR singlet at 173.10 ppm (Supplementary Materials Figure S44), whereas the substituting methoxy portion was spotted at 3.74 ppm within the ^{1}H NMR's 3.75 ppm multiplet (Supplementary Materials Figure S43), as ^{13}C NMR singlet at 51.82 ppm (Supplementary Materials Figure S44), and ^{17}O NMR singlet at 142.70 ppm (Supplementary Materials Figure S45). The methylene portion between the C1 carbonyl portion and the C3=C4 double bond was evident as ^{1}H NMR's 2.92 ppm multiplet (Supplementary Materials Figure S43) and ^{13}C NMR singlet at 34.95 ppm (Supplementary Materials Figure S44). Furthermore, the C3 carbon within the *trans*-$\Delta^{3,4}$ double bond was spotted at ^{1}H NMR's singlet at 6.03 ppm (Supplementary Materials Figure S43) and a ^{13}C NMR singlet at 134.67 ppm (Supplementary Materials Figure S44), whereas the corresponding C4 carbon emitted at 5.56 ppm within the ^{1}H NMR's 5.49 ppm triplet (Supplementary Materials Figure S43) and as ^{13}C NMR singlet at 134.50 ppm (Supplementary Materials Figure S44). Furthermore, the C4 methylene carbon substituted by MEMO- portion was evident as a sharp signal at 3.78 ppm within the ^{1}H NMR's multiplet at 3.75 ppm (Supplementary Materials Figure S43), and as a ^{13}C NMR singlet at 78.25 ppm (Supplementary Materials Figure S434). Moreover, the MEM protection was used for the C7-OH group as well; the C4 methylene carbon substituted by MEMO- portion was evident as a sharp signal at 3.31 ppm within the ^{1}H NMR's multiplet at 3.78 ppm (Supplementary Materials Figure S43) and as a ^{13}C NMR singlet at 78.25 ppm (Supplementary Materials Figure S44). The MEM signals associated with the substitution of either C4-OH or C7-OH groups were seen as ^{1}H NMR's 3.31, 3.50, 3.53, 4.30, and 5.45 ppm multiplets (Supplementary Materials Figure S43), ^{13}C NMR's 57.81, 67.74, 73.66 multiplets, as well as with 93.28 and 94.14 ppm singlets (Supplementary Materials Figure S44), and by the ^{17}O NMR's -23.80, 18.00, and 38.50 ppm multiplets (Supplementary Materials Figure S45), where the 4.30/5.45 ppm and 93.28/94.14 signals corresponded to the methylene groups directly attached to either C4 or C7 hydroxyl groups, respectively (either C4-O or C7-O atoms emitted as 18.00 ppm within the ^{17}O NMR multiplet). Further characterization is related to the former cyclopentane ring of BFA. Thus, the C5 carbon was noticed as a ^{13}C NMR's 43.69 singlet (Supplementary Materials Figure S44) while its *trans*-hydrogen substituent was spotted as a low-intensity ^{1}H NMR singlet at 1.96 ppm (Supplementary Materials Figure S43). The C6 methylene portion appeared as a ^{1}H NMR singlet at 1.46 ppm (Supplementary Materials Figure S43) and a ^{13}C NMR's 37.56 singlet (Supplementary Materials Figure S44), whereas the corresponding C8 position was emitting as ^{1}H NMR doublet at 2.14 ppm (Supplementary Materials Figure S43) and a ^{13}C NMR's 36.28 singlet (Supplementary Materials Figure S44); each of the signals was of low or medium intensity. In in-between the latter carbons, the C7 methylene backbone bearing the C7-OMEM arose as a high-intensity ^{1}H NMR peak at 3.31 ppm, as a part of 3.36 – 3.28 ppm multiplet (Supplementary Materials Figure S43), and a ^{13}C NMR's 80.61 ppm singlet (Supplementary Materials Figure S44). Finally, the C9 carbon as ^{13}C NMR singlet at 52.86 ppm (Supplementary Materials Figure S44) completed the cyclopentane ring, where its *cis*-hydrogen appeared at 2.06 ppm as a part of low-intensity ^{1}H NMR doublet at 2.08 ppm (Supplementary Materials Figure S43). Finally, the C10 aldehyde portion was confirmed by a ^{1}H NMR singlet at 9.72 ppm (Supplementary Materials Figures S42), by a ^{13}C NMR's singlet at 205.39 ppm (Supplementary Materials Figures S44).

The **R6**'s coupling with **R5** gave the *E*-olefin **R7** (Supplementary Materials Figures S46-S48) where the olefination reaction was confirmed by the loss of signals for C10 aldehyde portion and the appearance of *trans*-$\Delta^{10,11}$ double bond. Thus, the sp^2 hybridized C10 carbon was within the **R7** found at the lower fields, as a ^{1}H NMR 5.24 ppm singlet (Supplementary Materials Figure S46) and ^{13}C NMR's

singlet at 132.97 ppm (Supplementary Materials Figure S47). Moreover, the sp^2 hybridized C11 carbon was concluded as a low-intensity ^{1}H NMR singlet at 5.49 ppm (Supplementary Materials Figure S46) and a medium intensity ^{13}C NMR's singlet at 130.82 ppm (Supplementary Materials Figure S47). The C12, C13, C14, and C15 carbons were found as a ^{1}H NMR's 1.60 ppm multiplet (Supplementary Materials Figure S46) and a ^{13}C NMR's singlet at 32.41 ppm (Supplementary Materials Figure S47), a signal at 1.35 ppm of ^{1}H NMR's 1.44 ppm doublet (Supplementary Materials Figure S46), and a ^{13}C NMR's singlet at 22.92 ppm (Supplementary Materials Figure S47), a ^{1}H NMR's 1.62 ppm singlet (Supplementary Materials Figure S46) and a ^{13}C NMR's singlet at 32.63 ppm (Supplementary Materials Figure S47), a ^{1}H NMR's 4.82 ppm singlet (Supplementary Materials Figure S46) and a ^{13}C NMR's singlet at 100.70 ppm (Supplementary Materials Figures S47), respectively. The C15 methoxy group was recognized within the ^{1}H NMR's 3.33 ppm multiplet (Supplementary Materials Figure S46) as a 3.32 ppm signal, a ^{13}C NMR's singlet at 55.30 ppm (Supplementary Materials Figure S47), and a ^{17}O NMR's 23.00 ppm singlet (Supplementary Materials Figure S48). Finally, the C15-O-TBS scaffold was evident in the expected fields (Supplementary Materials Figures S47-S48).

Furthermore, the **R8**'s (Supplementary Materials Figures S49-S51) synthesis assumed the C15 hydroxyl group deprotection, which was confirmed by ^{1}H NMR's 4.25 ppm singlet (Supplementary Materials Figure S49) and ^{17}O NMR's 44.50 ppm singlet (Supplementary Materials Figure S51).

Within the **BFA-D1** (Supplementary Materials Figures S52-S54), the corresponding ^{1}H NMR peak for the C15-OH group was lost due to the ring closure (Supplementary Materials Figure S52). The ring closure was verified by the slight shift of the C15 carbon towards the upper field within the ^{1}H NMR spectrum as a low-intensity singlet at 6.37 ppm (Supplementary Materials Figure S52) and a moderate intensity singlet ^{13}C NMR singlet at 101.68 ppm (Supplementary Materials Figure S53). On the other hand, the synthesis of **BFA-D2** (Supplementary Materials Figures S55-S57) was characterized by the deprotection of the C7-OH portion, quantified by the appearance of medium intensity ^{1}H NMR singlet peak at 4.19 ppm (Supplementary Materials Figure S55) and high intensity ^{17}O NMR singlet at 33.40 ppm (Supplementary Materials Figure S57), which could be differentiated from the C4-OH portion (^{1}H NMR singlet at 4.05 ppm, ^{17}O NMR singlet at 32.30 ppm Supplementary Materials Figures S55 and S57, respectively). Of course, the MEM signals were absent in the corresponding spectra (Supplementary Materials Figures S55-S57).

Nevertheless, for further guided synthesis of **BFA-D4**, within the **BFA-D3** (Supplementary Materials Figures S58-S60) the C7-OH portion was protected with TBS, resulting in the absence of the corresponding ^{1}H NMR singlet at 4.19 ppm (Supplementary Materials Figure S58) and the appearance of known signals for the protective group (Supplementary Materials Figures S59-S60).

The aforementioned guided synthesis of **BFA-D4** (Supplementary Materials Figures S61-S63) assumed the esterification of the **BFA-D3**'s C4-OH portion with the 3-acetyl-4-hydroxybenzoic acid. Thus, the esterification was initially confirmed by the appearance of the ester carbonyl group within the ^{13}C NMR spectrum as a moderate intensity singlet at 166.92 ppm (Supplementary Materials Figure S62) and within the ^{17}O NMR spectrum as a medium intensity singlet at 338.90 ppm (Supplementary Materials Figure S63), as well as by the existence of ester C4 oxygen within the ^{17}O NMR spectrum as a medium intensity singlet at 130.60 ppm (Supplementary Materials Figure S63) and the absence of the corresponding C4-OH singlet within the ^{1}H NMR spectrum (Supplementary Materials Figure S61). The ^{1}H NMR spectrum, however, confirmed the incorporation of benzene moiety by means of the 7.00, 8.00, and 8.64 ppm singlets (Supplementary Materials Figure S61), as well as by the appearance of ^{13}C NMR singlets at 166.92 ppm (originating from the carbon bearing the ester functionality), 122.70 ppm (from the carbon bearing the C3′-acetyl group), as well as at 165.45 ppm (the carbon bearing the *p*-OH portion), respectively (Supplementary Materials Figure S62). The C3′-acetyl substituent was recognized as a 2.53 ppm multiplet within the ^{1}H NMR spectrum (Supplementary Materials Figure S61) and ^{13}C NMR's 28.28 ppm singlet, belonging to the methyl group (Supplementary Materials Figure S62), as well as by 202.48

ppm singlet within the ^{13}C NMR spectrum (Supplementary Materials Figure S62) and 488.00 ppm singlet within the ^{17}O NMR spectrum, originating from the carbonyl group (Supplementary Materials Figure S63). The *p*-OH portion was evident as a low-intensity ^{1}H NMR singlet at 8.62 ppm (Supplementary Materials Figure S61) and a high intensity ^{17}O NMR singlet at 85.80 ppm (Supplementary Materials Figure S63).

The synthesis of **BFA-D4** provided the basis for the preparation of **3DPQ-1** to **3DPQ-12**, *via* the corresponding precursors **Pro-3DPQ-1** to **Pro-3DPQ-12**. Nevertheless, before the **Pro-3DPQ-3**, **3DPQ-6**, **3DPQ-7**, **Pro-3DPQ-8**, **3DPQ-9**, and **Pro-3DPQ-12** preparation, the corresponding precursors **Pro-R3**, **Pro-R6**, **Pro-R7**, **Pro-R8**, **Pro-R9**, and **Pro-R12** needed to be synthesized.

Thus, within **Pro-R3** (Supplementary Materials Figures S64-S67) the remains of the 2-chloroethanol were elucidated by means of a ^{1}H NMR multiplet at 3.70 ppm (Supplementary Materials Figure S64) and ^{13}C NMR singlet at 40.27 ppm (Supplementary Materials Figure S65), assigned to the methylene group substituted by chlorine, as well as by ^{1}H NMR singlet at 2.63 ppm (Supplementary Materials Figure S64) and ^{13}C NMR singlet at 56.04 ppm (Supplementary Materials Figure S65), originating from the methylene group substituted by 1,2,5,6-tetrahydropyridine-3-carboxylic acid. Within the heterocycle, the carboxyl group was recognized by the presence of the sharp ^{1}H NMR's singlet at 13.18 ppm (Supplementary Materials Figure S64), whereas the carbonyl portion was spotted at ^{13}C NMR's 166.47 ppm as a singlet (Supplementary Materials Figure S65) and ^{17}O NMR's 337.20 ppm as a singlet (Supplementary Materials Figure S67). The C3=C4 double bond bearing the -COOH portion was evident by means of the C3 signal at ^{13}C NMR's 126.73 ppm in the form of a singlet (Supplementary Materials Figure S65), as well as by the C4's low-intensity ^{1}H NMR's singlet at 7.31 ppm (Supplementary Materials Figure S64) and medium-intensity ^{13}C NMR's singlet at 142.15 ppm (Supplementary Materials Figure S65). Of course, the substitution of 2-chloroethanol with 1,2,5,6-tetrahydropyridine-3-carboxylic acid (*i.e* nitrogen within the 2-chloroethanamine fragment) was spotted as ^{15}N NMR singlet at -330.10 ppm (Supplementary Materials Figure S66).

Thus, within **Pro-R6** (Supplementary Materials Figures S68-S70) the remains of the 2-chloroethanol were elucidated by means of a ^{1}H NMR multiplet at 3.69 ppm (Supplementary Materials Figure S68) and ^{13}C NMR singlet at 40.27 ppm (Supplementary Materials Figure S69), assigned to the methylene group substituted by chlorine, as well as by ^{1}H NMR singlet at 2.61 ppm (Supplementary Materials Figure S68) and ^{13}C NMR singlet at 55.47 ppm (Supplementary Materials Figure S69), originating from the methylene group substituted by 6,7-dihydro-5*H*-pyrrolo[3,4-b]pyridine. Within the heterocycle, the five-membered ring (*i.e.* pyrrolidine's) methylene groups were recognized as ^{1}H NMR's 3.26 ppm (Supplementary Materials Figure S68) and ^{13}C NMR's 63.36 ppm (Supplementary Materials Figure S69) singlets, as well as by ^{1}H NMR's 3.58 ppm (Supplementary Materials Figure S68) and ^{13}C NMR's 61.31 ppm (Supplementary Materials Figure S69) singlets, whereas the six-membered ring (*i.e.* pyridine's) methylene groups were recognized as ^{1}H NMR's 7.28 ppm (Supplementary Materials Figure S68) and ^{13}C NMR's 121.84 ppm (Supplementary Materials Figure S69) singlets, ^{1}H NMR's 7.76 ppm (Supplementary Materials Figure S67) and ^{13}C NMR's 130.94 ppm (Supplementary Materials Figure S69) singlets, and ^{1}H NMR's ppm 8.30 ppm (Supplementary Materials Figure S68) and ^{13}C NMR's 146.33 (the iminium function within the pyridine) ppm (Supplementary Materials Figure S69) singlets. The 6,7-dihydro-5*H*-pyrrolo[3,4-b]pyridine's double bond was evident at ^{13}C NMR's 126.54 and 162.61 (the =C-N-fragment carbon within the pyridine) ppm (Supplementary Materials Figure S69) singlets. Of course, 6,7-dihydro-5*H*-pyrrolo[3,4-b]pyridine substituting the 2-chloroethanol (*i.e* nitrogen within the 2-chloroethanamine fragment) was spotted as ^{15}N NMR singlet at -315.50 ppm, whereas the pyridine nitrogen within the construct emitted as ^{15}N NMR singlet at -47.00 ppm (Supplementary Materials Figure S70).

As for **Pro-R7** (Supplementary Materials Figures S71-S73), the substitution of 2-chloroethanol with 1,2-dihydroisoquinoline resulted in the change of fields on which the methylene group attached to

the nitrogen atom of 1,2-dihydroisoquinoline emitted: the singlets at ^{1}H NMR's 2.79 ppm (Supplementary Materials Figure S71) and ^{13}C NMR's at 52.74 ppm (Supplementary Materials Figure S72) characterized such coupling. As expected, the 1,2-dihydroisoquinoline was characterized by the appropriate ^{1}H NMR signals within the 4.44 to 7.41 ppm range (Supplementary Materials Figure S71), as well as by ^{13}C NMR signals between the 51.86 ppm and 140.38 ppm (Supplementary Materials Figure S72) (the limit values are for methylene groups covalently bonded to the nitrogen atom). The nitrogen atom itself signalized as ^{15}N NMR singlet at -287.10 ppm (Supplementary Materials Figure S73).

The derivatization of 2-chloroethanol with decahydro-1,8-naphthyridine yielded **Pro-R8** (Supplementary Materials Figures S74-S76), in which the methylene group attached to one of the nitrogens within the decahydro-1,8-naphthyridine emitted as ^{1}H NMR's singlet at 3.51 ppm (Supplementary Materials Figure S74) and ^{13}C NMR's singlet at 54.16 ppm (Supplementary Materials Figure S75). The decahydro-1,8-naphthyridine skeleton emitted in the ^{1}H NMR range of 1.26 to 2.88 ppm (Supplementary Materials Figure S74) and in the ^{13}C NMR range of 23.23 to 65.38 ppm (Supplementary Materials Figure S75). Regarding nitrogen substitution, it was confirmed with the presence of ^{15}N NMR singlet at -326.40 ppm (Supplementary Materials Figure S76); the unsubstituted -NH- portion was evident as a ^{1}H NMR singlet at 2.77 ppm (Supplementary Materials Figure S74) and a ^{15}N NMR singlet at -334.90 ppm (Supplementary Materials Figure S76).

The derivatization of 2-chloroethanol with 6-oxo-1,2,5,6-tetrahydropyridine-3-carboxylic acid yielded **Pro-R9** (Supplementary Materials Figures S77-S79), where the newly introduced C6 carbonyl group related to **Pro-R3** (Supplementary Materials Figures S64-S67) was qualified by means of ^{13}C NMR's singlet at 167.89 ppm (Supplementary Materials Figure S78) and ^{17}O NMR's singlet at 346.00 ppm (Supplementary Materials Figure S80).

Finally, the derivatization of 2-chloroethanol with 3-hydroxypiperidin-2-one resulted in **Pro-R12** (Supplementary Materials Figures S81-S84), in which the methylene group attached to the nitrogen atom within the heterocycle emitted as 3.44 peak within the ^{1}H NMR's multiplet at 3.41 ppm (Supplementary Materials Figure S81) and ^{13}C NMR's singlet at 48.88 ppm (Supplementary Materials Figure S82). The 3-hydroxypiperidin-2-one skeleton emitted in the ^{1}H NMR range of 1.67 to 4.44 ppm (Supplementary Materials Figure S81) and in the ^{13}C NMR range of 22.42 to 175.82 ppm (Supplementary Materials Figure S82), the hydroxyl group was found at ^{1}H NMR's 5.44 ppm singlet (Supplementary Materials Figure S81), ^{13}C NMR's 67.27 ppm singlet (Supplementary Materials Figure S82) and ^{17}O NMR singlet at 32.30 ppm (Supplementary Materials Figure S84), while the carbonyl group was recognized as ^{13}C NMR's 175.82 ppm singlet (Supplementary Materials Figure S82) and ^{17}O NMR singlet at 346.00 ppm (Supplementary Materials Figure S84). Regarding nitrogen substitution, it was confirmed with the presence of a ^{15}N NMR singlet at -257.80 ppm (Supplementary Materials Figure S83).

Being produced, **BFA-D4**'s *p*-OH moiety underwent the equimolar alkylation with either 2-chloro-*N*-methylethanamine (**Pro-R1**), 2-chloro-*N*,*N*-dimethylethanamine (**Pro-R2)**, 1-(2-chloroethyl)-1,2,5,6-tetrahydropyridine-3-carboxylic acid (**Pro-R3**), 2-chloroethanesulfonic acid (**Pro-R4)**, 1-(2-chloroethyl)pyrrolidine-2,5-dione (**Pro-R5)**, 6-(2-chloroethyl)-6,7-dihydro-5*H*-pyrrolo[3,4-b]pyridine (**Pro-R6**), 2-(2-chloroethyl)-1,2-dihydroisoquinoline (**Pro-R7**), 1-(2-chloroethyl)decahydro-1,8-naphthyridine (**Pro-R8**), 1-(2-chloroethyl)-6-oxo-1,2,5,6-tetrahydropyridine-3-carboxylic acid (**Pro-R9)**, 1-(2-chloroethyl)piperidine **(Pro-R10**), 1-(2-chloroethyl)pyrrolidine (**Pro-R11**), or 1-(2-chloroethyl)-3-hydroxypiperidin-2-one (**Pro-R12**), to give either **Pro-3DPQ-1** (Supplementary Materials Figures S85-S88), **Pro-3DPQ-2** (Supplementary Materials Figures S89-S92), **Pro-3DPQ-3** (Supplementary Materials Figures S93-S96), **Pro-3DPQ-4** (Supplementary Materials Figures S97-S99), **Pro-3DPQ-5** (Supplementary Materials Figures S100-S103), **Pro-3DPQ-6** (Supplementary Materials Figures S104-S107), **Pro-3DPQ-7** (Supplementary Materials Figures S108-S111), **Pro-3DPQ-8** (Supplementary Materials Figures S112-S115), **Pro-3DPQ-9** (Supplementary Materials Figures S116-S119), **Pro-3DPQ-10** (Supplementary Materials

Figures S120-S123), **Pro-3DPQ-11** (Supplementary Materials Figures S124-S127), or **Pro-3DPQ-12** (Supplementary Materials Figures S128-S131), respectively.

The *p*-OH portion substitution was evident with the loss of the former ^{1}H NMR singlet at 8.62 ppm (Supplementary Materials Figure S61) and a ^{17}O NMR singlet at 85.80 ppm (Supplementary Materials Figure S63), as well as with the appearance of the etheric oxygen as ^{17}O NMR singlet at 78.00 ppm within either **Pro-3DPQ-1** (Supplementary Materials Figure S88), **Pro-3DPQ-2** (Supplementary Materials Figure S92), **Pro-3DPQ-3** (Supplementary Materials Figure S96), **Pro-3DPQ-4** (Supplementary Materials Figure S99), **Pro-3DPQ-5** (Supplementary Materials Figure S103), **Pro-3DPQ-6** (Supplementary Materials Figure S107), **Pro-3DPQ-7** (Supplementary Materials Figure S111), **Pro-3DPQ-8** (Supplementary Materials Figure S115), **Pro-3DPQ-9** (Supplementary Materials Figure S119), **Pro-3DPQ-10** (Supplementary Materials Figure S123), **Pro-3DPQ-11** (Supplementary Materials Figure S127), or **Pro-3DPQ-12** (Supplementary Materials Figure S131), respectively. The remaining newly introduced functional groups for either **Pro-3DPQ-1**, **Pro-3DPQ-2**, **Pro-3DPQ-3**, **Pro-3DPQ-4**, **Pro-3DPQ-5**, **Pro-3DPQ-6**, **Pro-3DPQ-7**, **Pro-3DPQ-8**, **Pro-3DPQ-10**, **Pro-3DPQ-11**, or **Pro-3DPQ-12** were at their expected chemical shifts.

Finally, the deprotection of either C7-OTMS or C15-O-CH_3 within either **Pro-3DPQ-1**, **Pro-3DPQ-2**, **Pro-3DPQ-3**, **Pro-3DPQ-4**, **Pro-3DPQ-5**, **Pro-3DPQ-6**, **Pro-3DPQ-7**, **Pro-3DPQ-8**, **Pro-3DPQ-9**, **Pro-3DPQ-10**, **Pro-3DPQ-11**, or **Pro-3DPQ-12** gave **3DPQ-1** (Supplementary Materials Figures S132-S135), **3DPQ-2** (Supplementary Materials Figures S136-S139), **3DPQ-3** (Supplementary Materials Figures S140-S143), **3DPQ-4** (Supplementary Materials Figures S144-S146), **3DPQ-5** (Supplementary Materials Figures S147-S150), **3DPQ-6** (Supplementary Materials Figures S151-S154), **3DPQ-7** (Supplementary Materials Figures S155-S158), **3DPQ-8** (Supplementary Materials Figures S155-S162), **3DPQ-9** (Supplementary Materials Figures S163-S166), **3DPQ-10** (Supplementary Materials Figures S167-S170), **3DPQ-11** (Supplementary Materials Figures S171-S174), or **3DPQ-12** (Supplementary Materials Figures S175-S178), respectively.

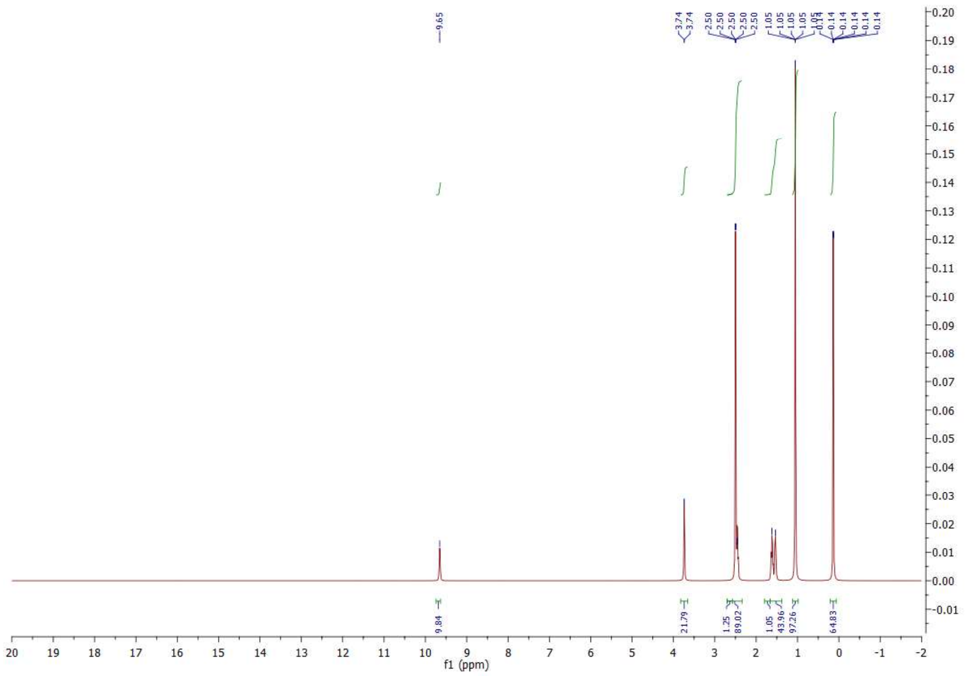

Figure S26. The ^{1}H NMR spectrum of **R1**.

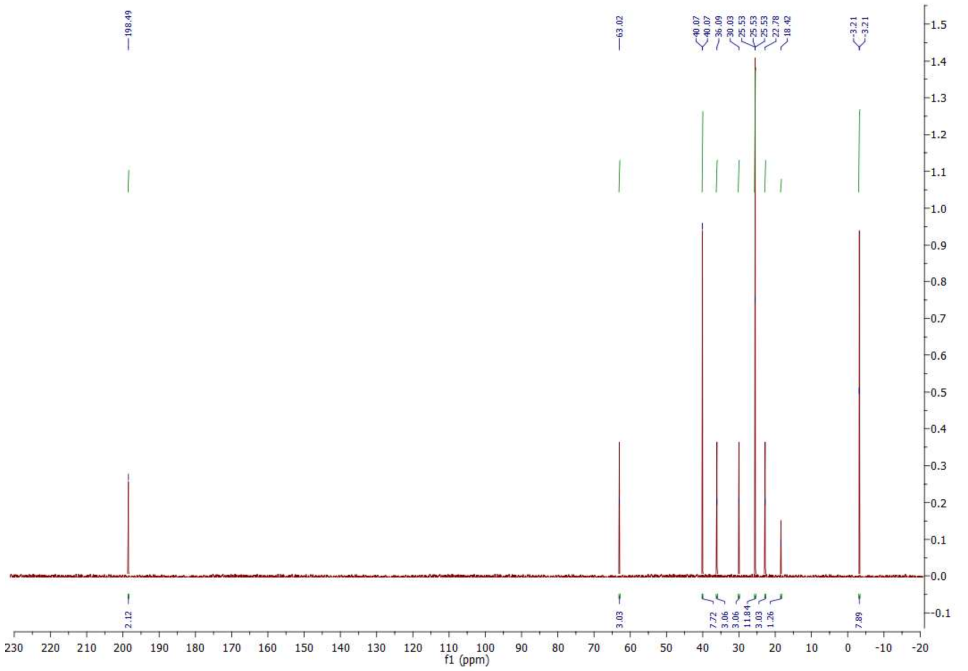

Figure S27. The ^{13}C NMR spectrum of **R1**.

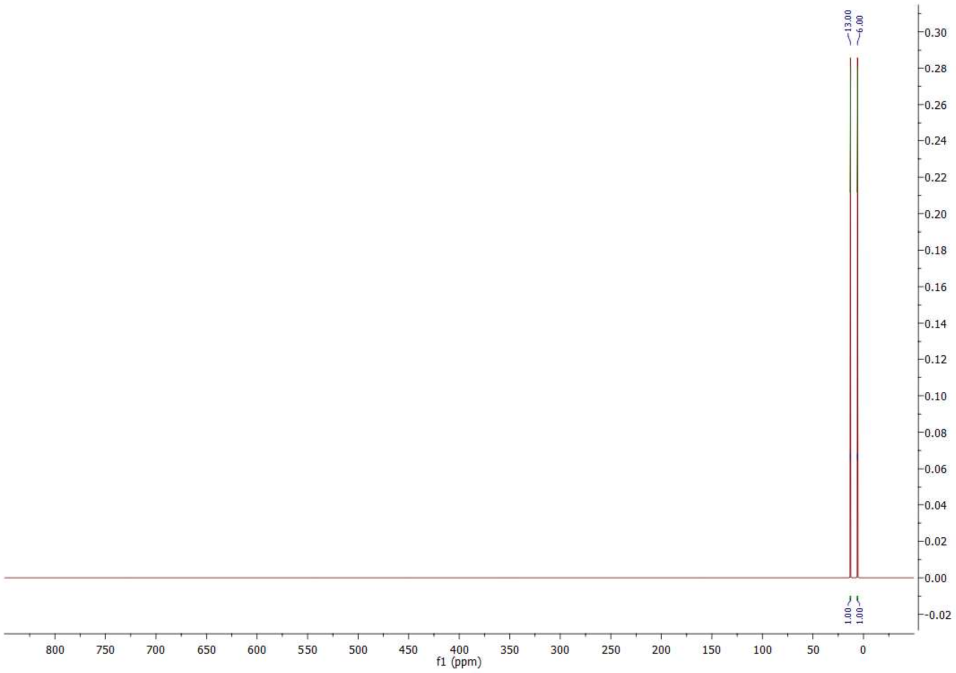

Figure S28. The ^{17}O NMR spectrum of **R1**.

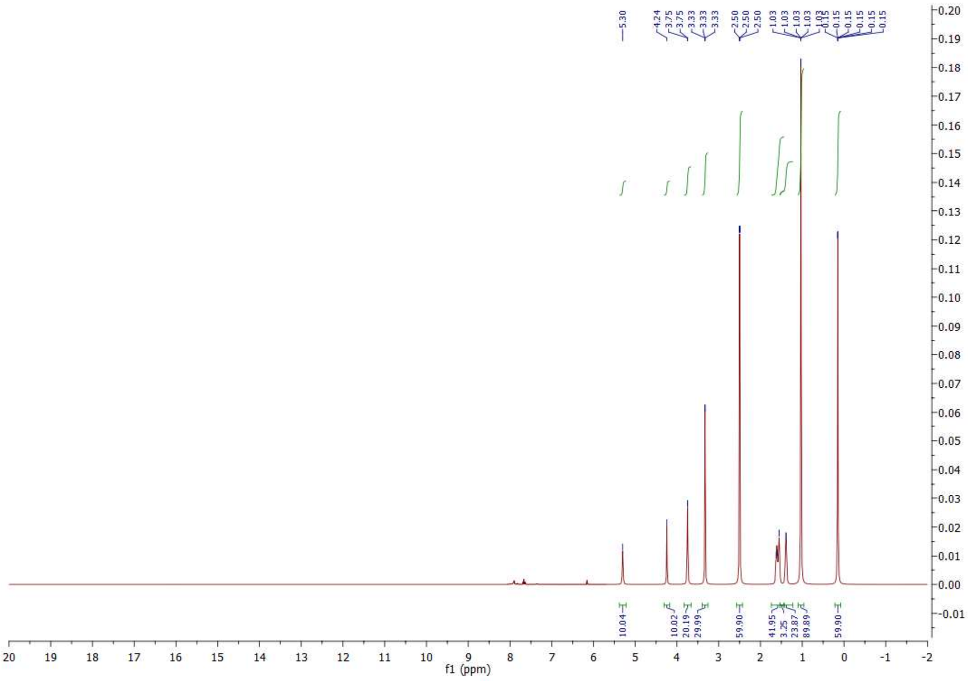

Figure S29. The ^{1}H NMR spectrum of **R2**.

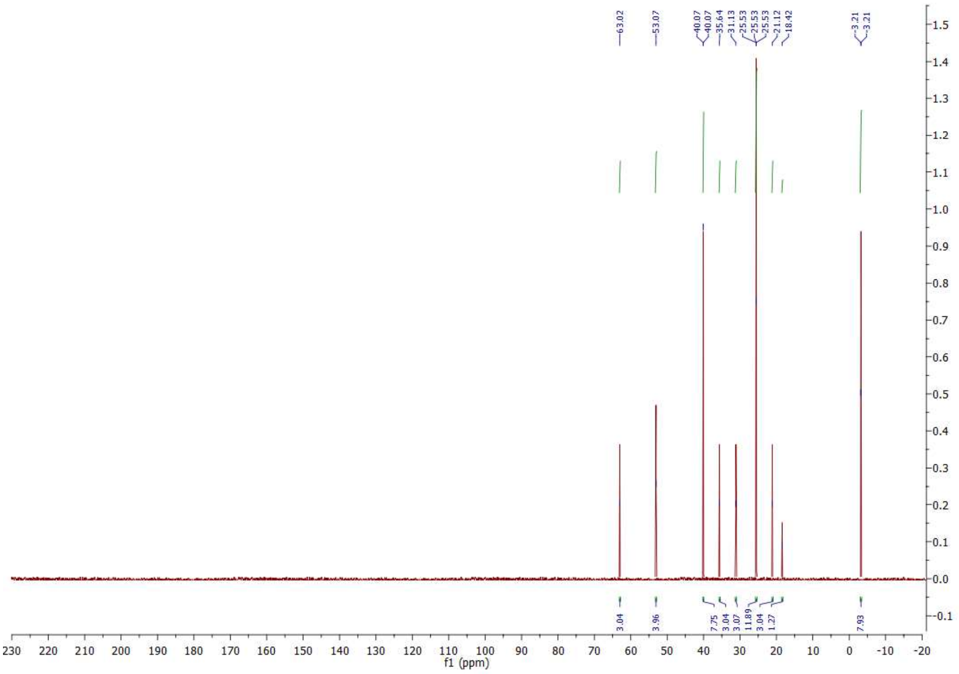

Figure S30. The ^{13}C NMR spectrum of **R2**.

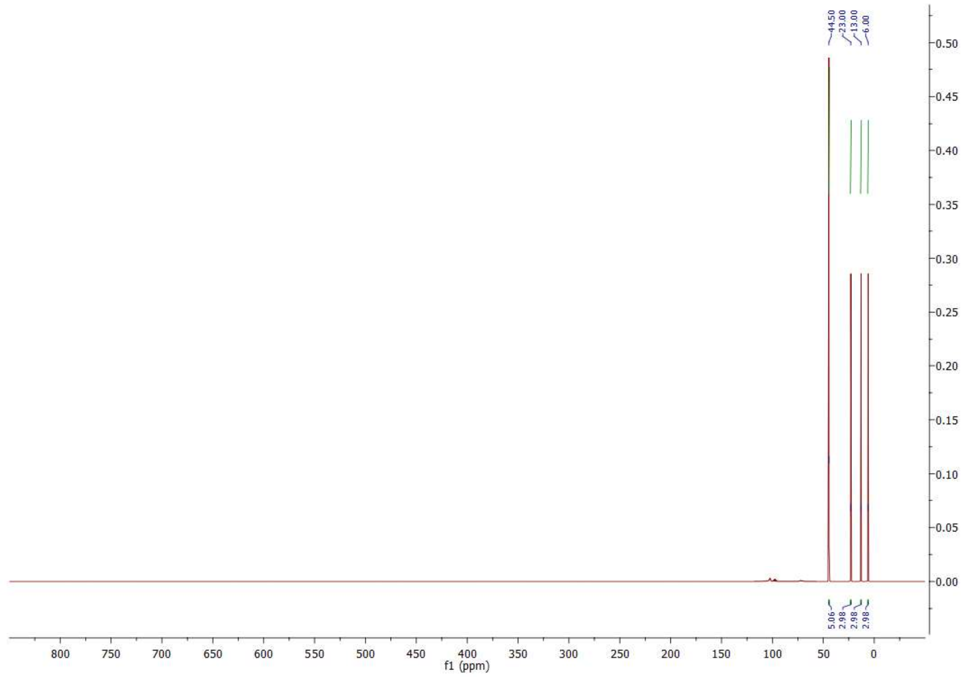

Figure S31. The ^{17}O NMR spectrum of **R2**.

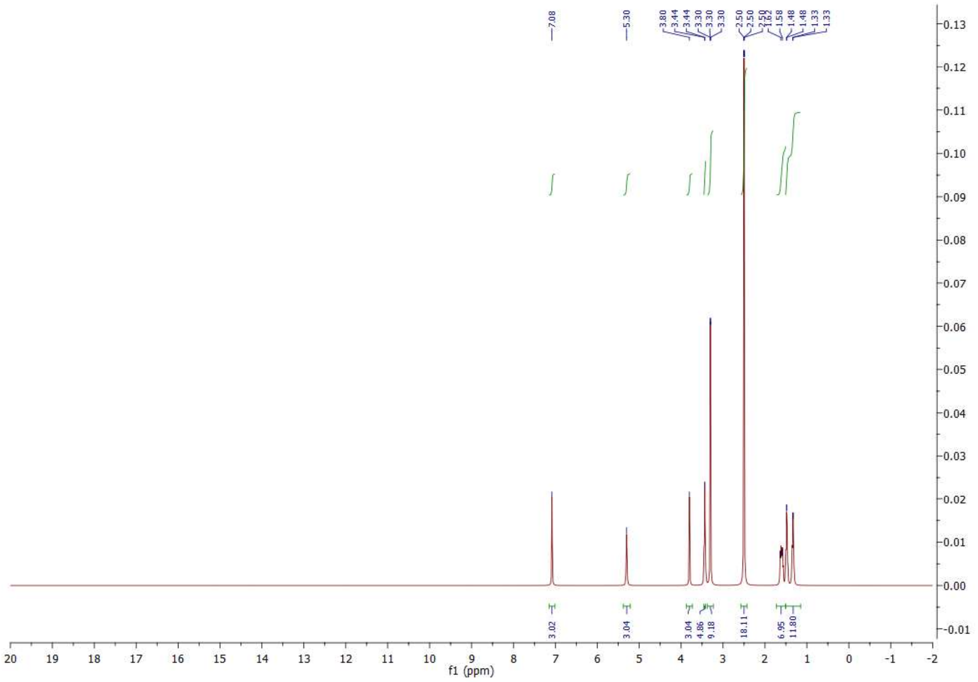

Figure S32. The ^{1}H NMR spectrum of **R3**.

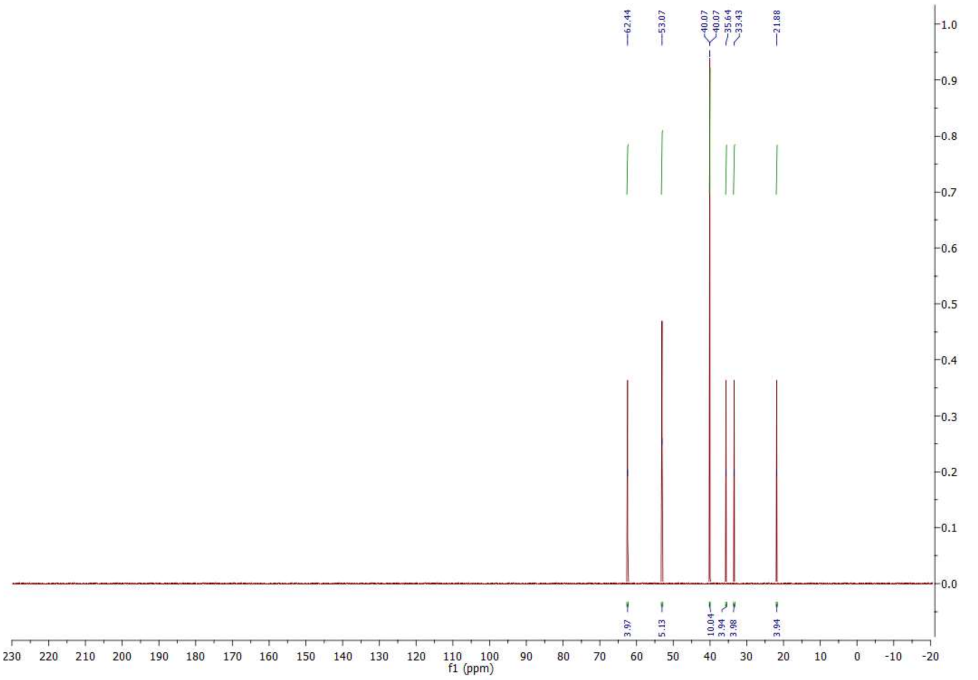

Figure S33. The ^{13}C NMR spectrum of **R3**.

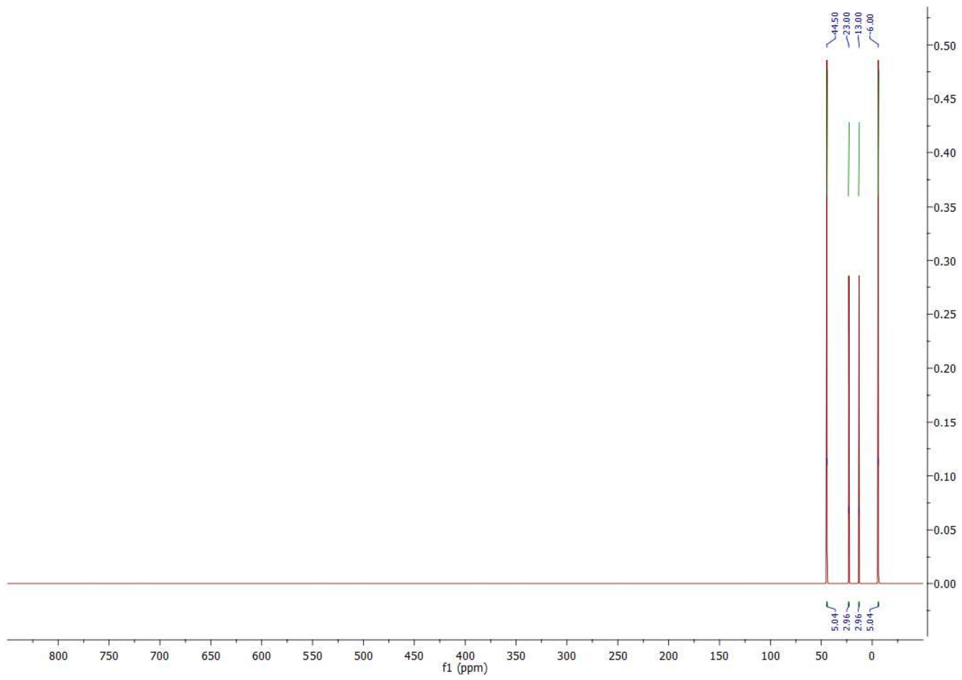

Figure S34. The ^{17}O NMR spectrum of **R3**.

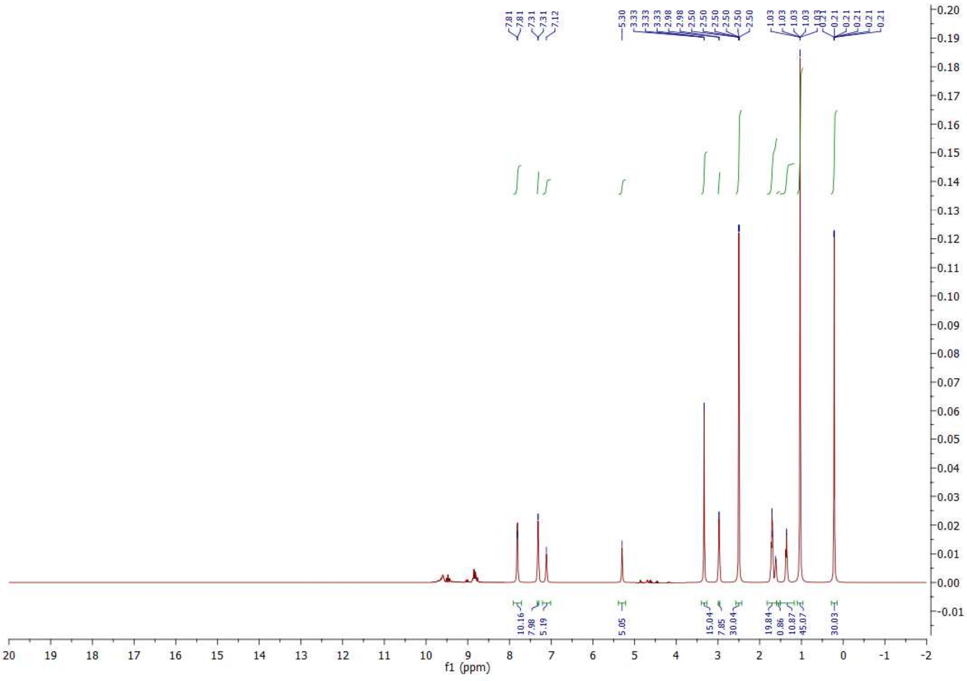

Figure S35. The ^{1}H NMR spectrum of **R4**.

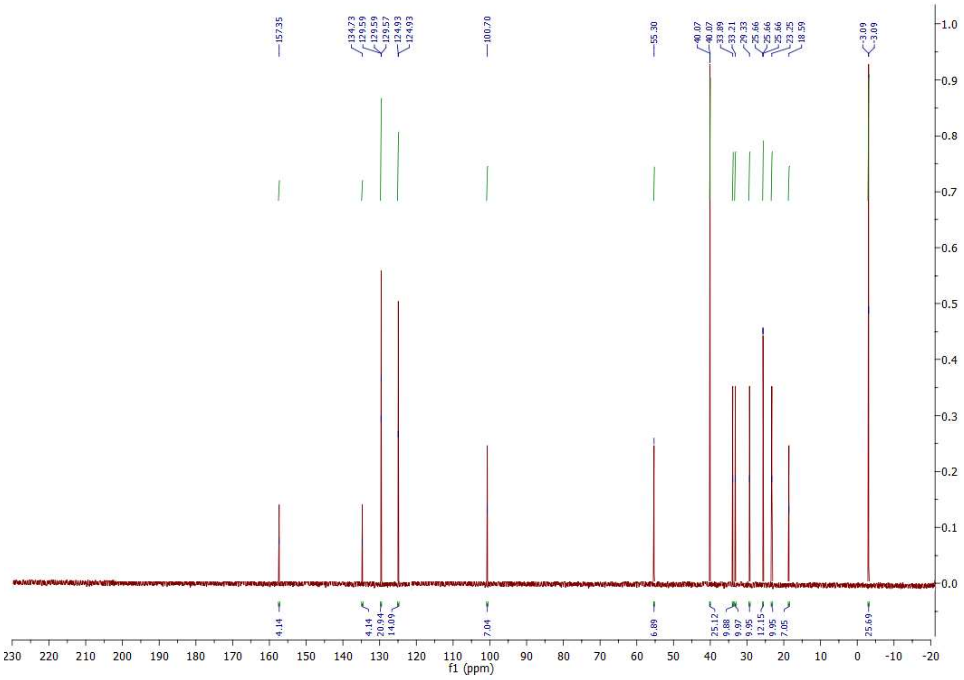

Figure S36. The ^{13}C NMR spectrum of **R4**.

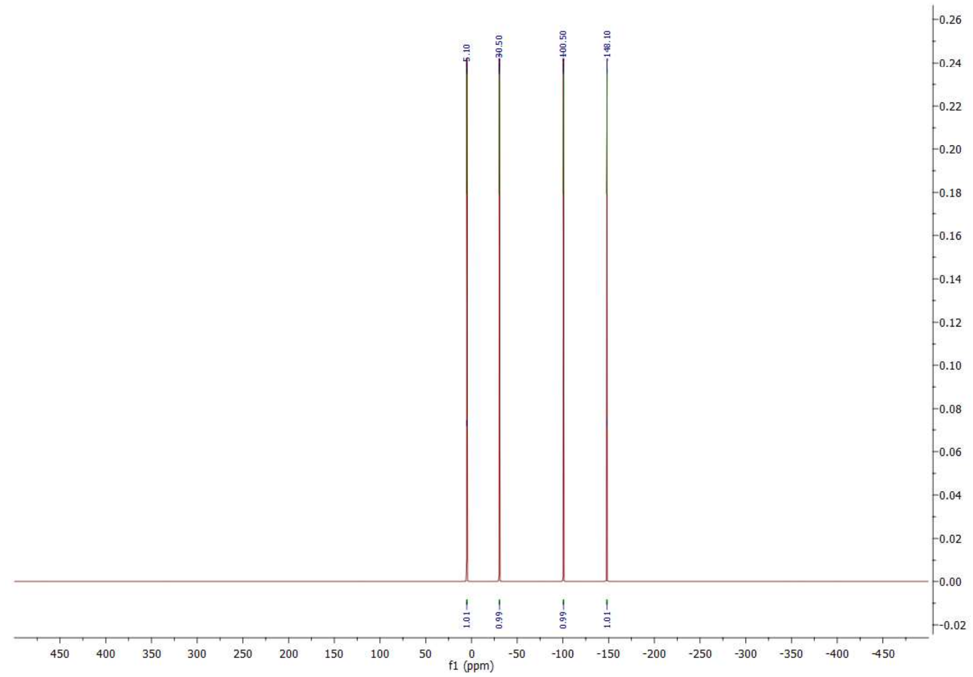

Figure S37. The ^{15}N NMR spectrum of **R4**.

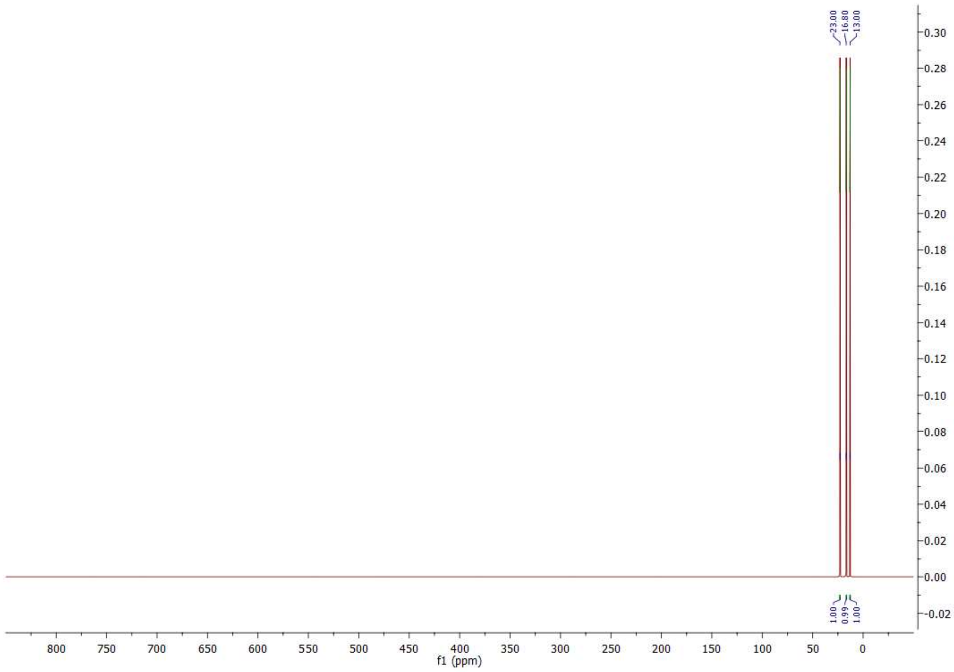

Figure S38. The ^{17}O NMR spectrum of **R4**.

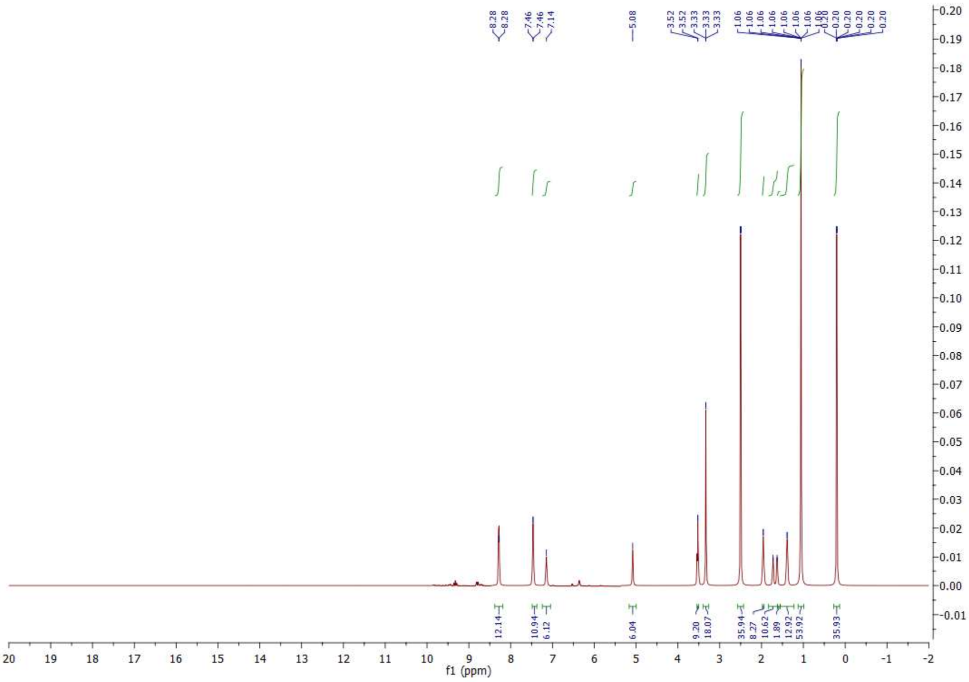

Figure S39. The ^{1}H NMR spectrum of **R5**.

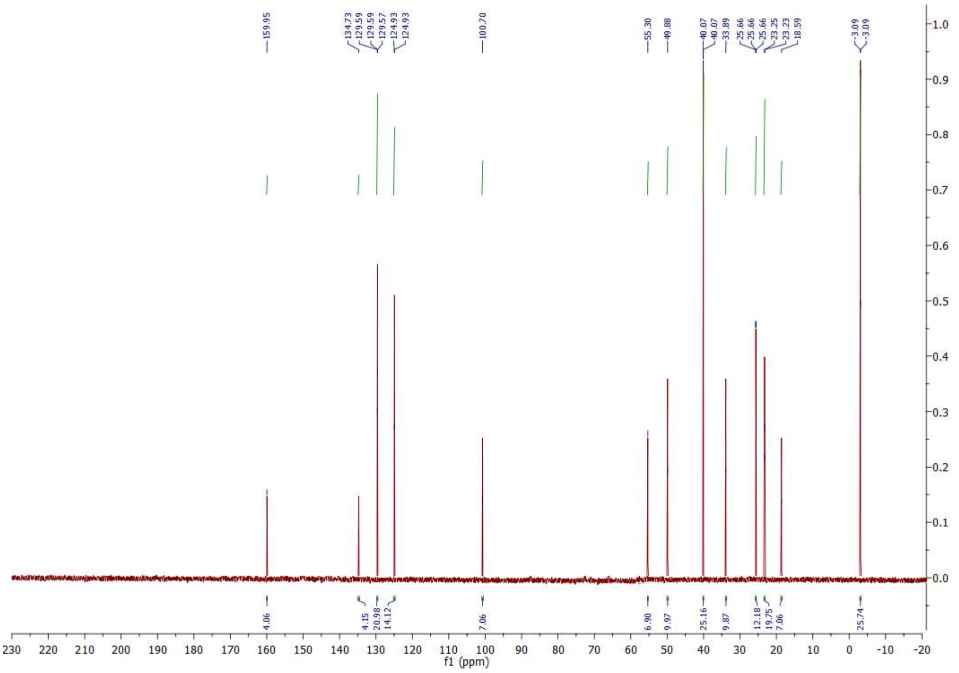

Figure S40. The ^{13}C NMR spectrum of **R5**.

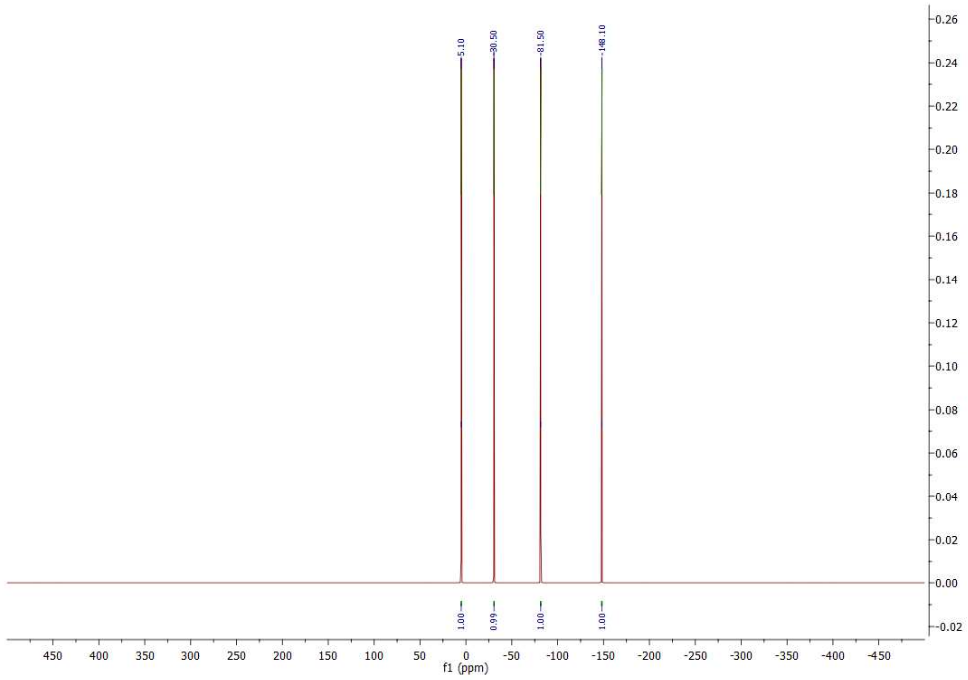

Figure S41. The ^{15}N NMR spectrum of **R5**.

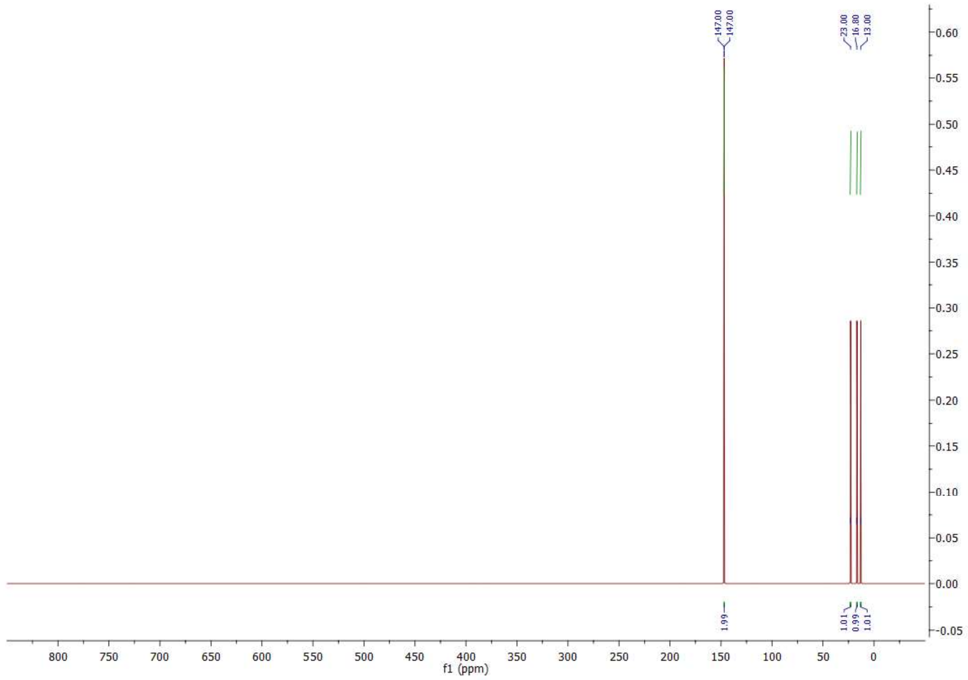

Figure S42. The ^{17}O NMR spectrum of **R5**.

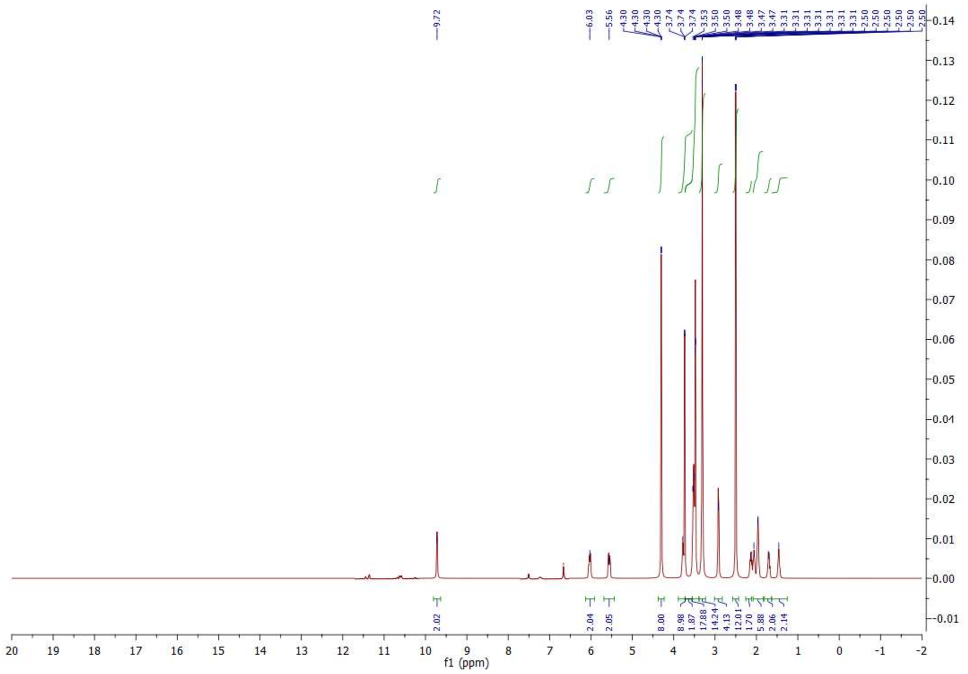

Figure S43. The ^{1}H NMR spectrum of **R6**.

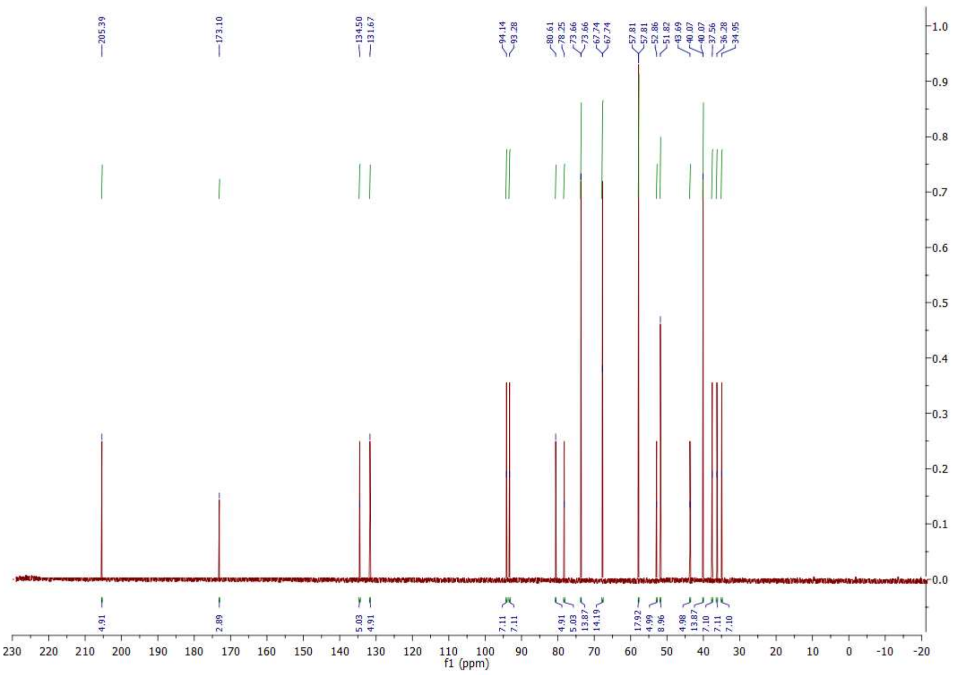

Figure S44. The ^{13}C NMR spectrum of **R6**.

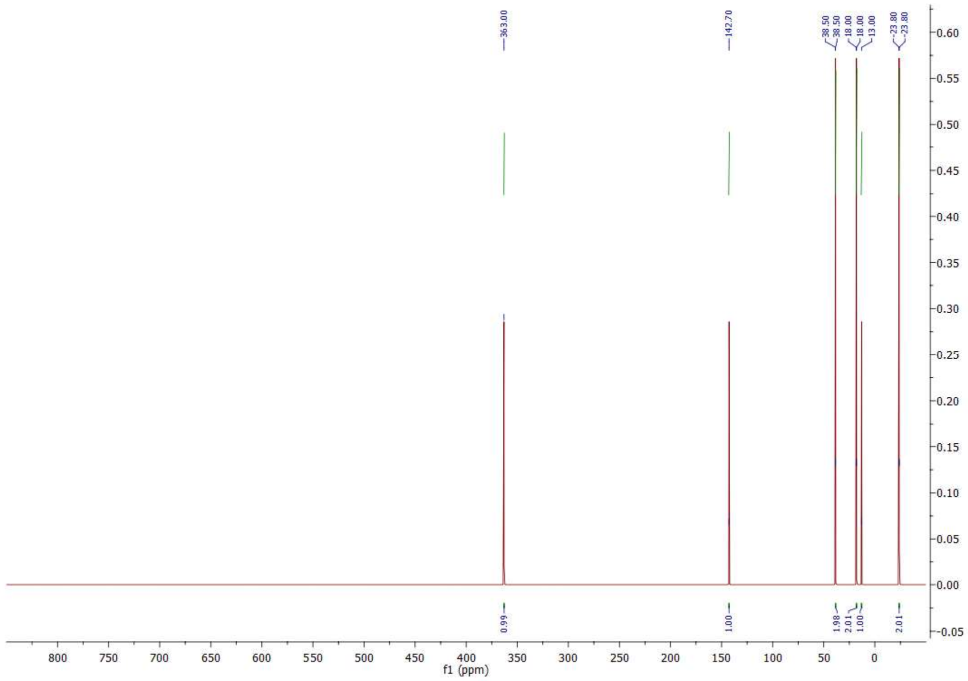

Figure S45. The ^{17}O NMR spectrum of **R6**.

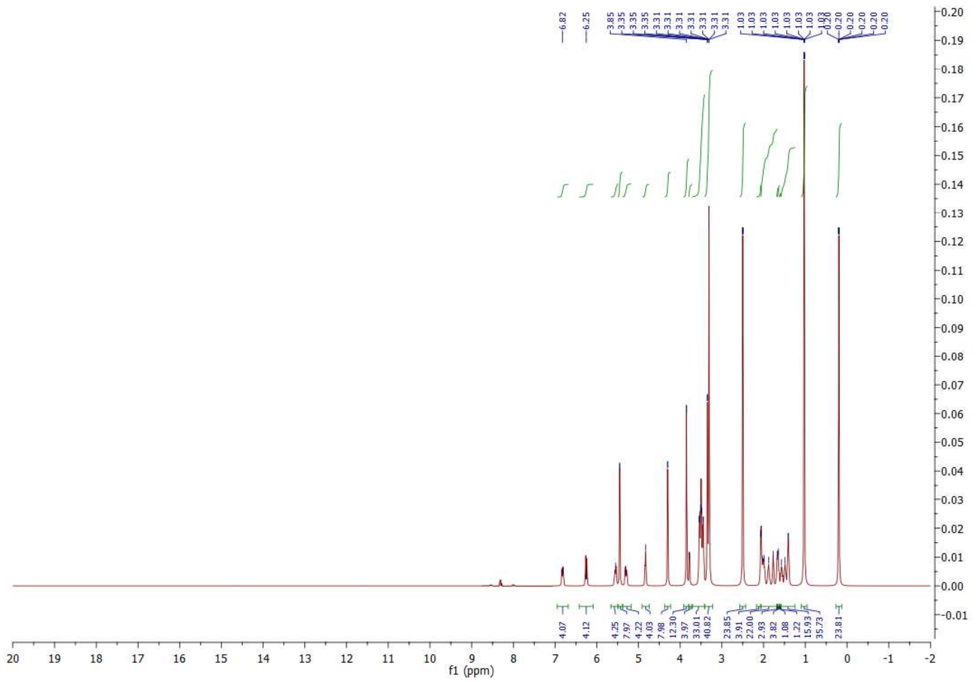

Figure S46. The ^{1}H NMR spectrum of **R7**.

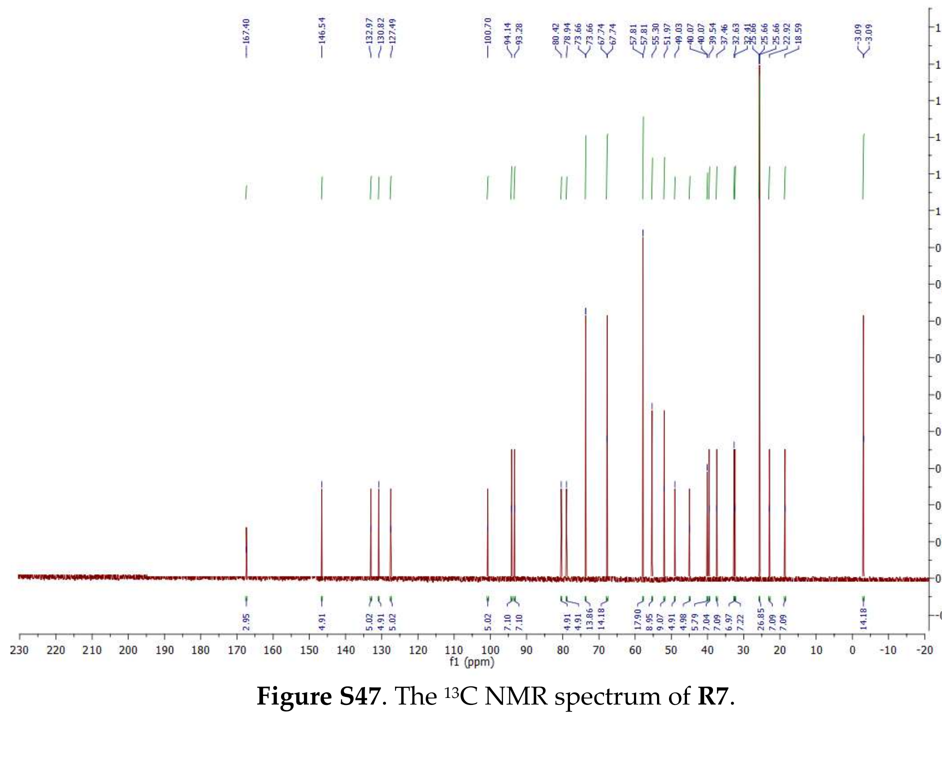

Figure S47. The ^{13}C NMR spectrum of **R7**.

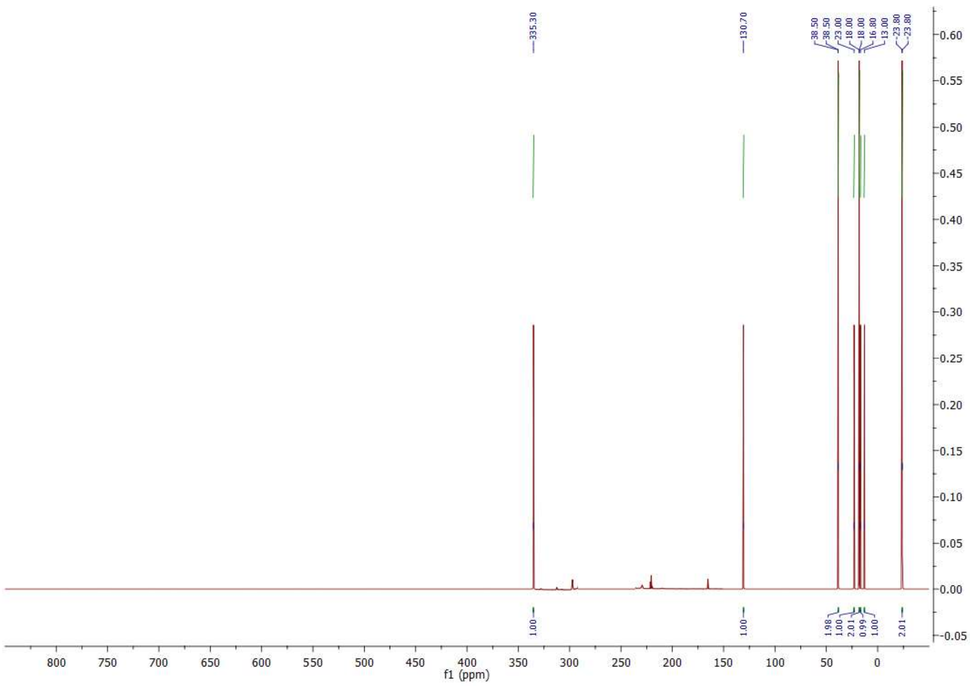

Figure S48. The ^{17}O NMR spectrum of **R7**.

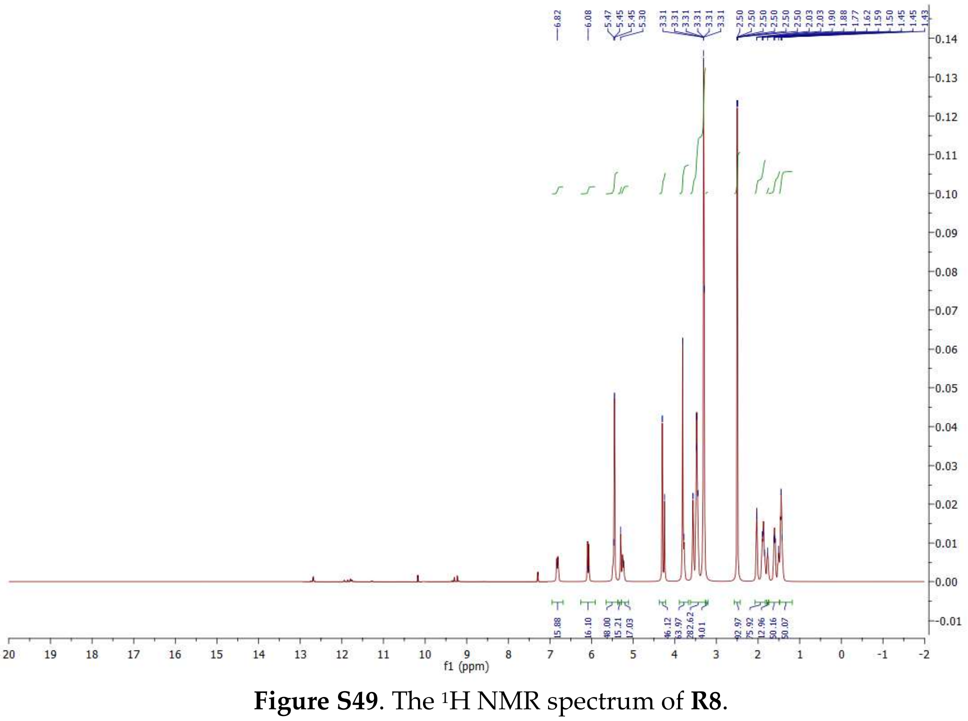

Figure S49. The ^{1}H NMR spectrum of **R8**.

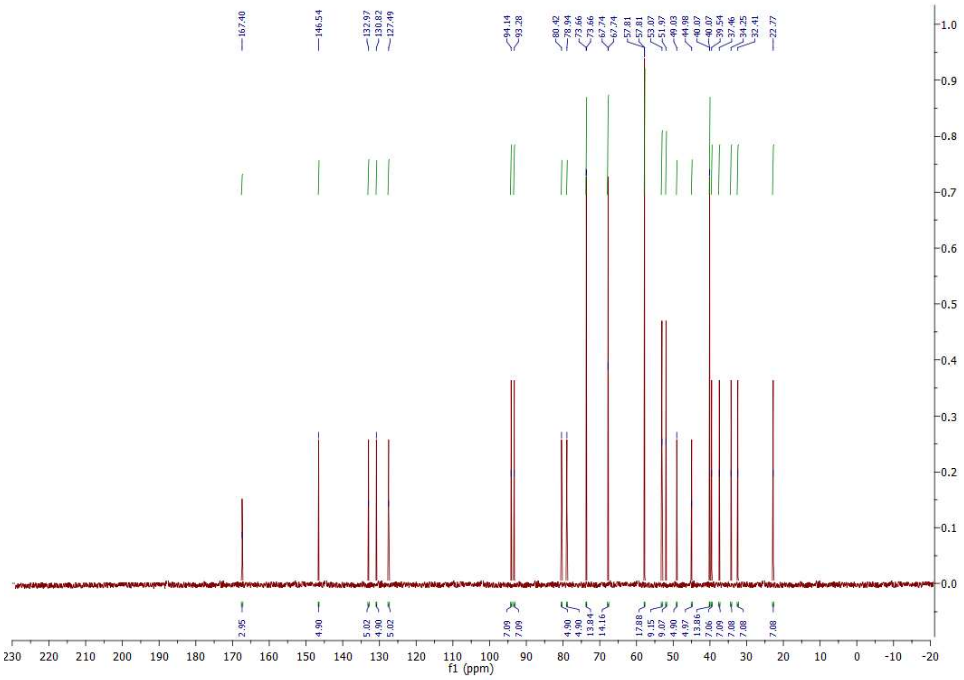

Figure S50. The ^{13}C NMR spectrum of **R8**.

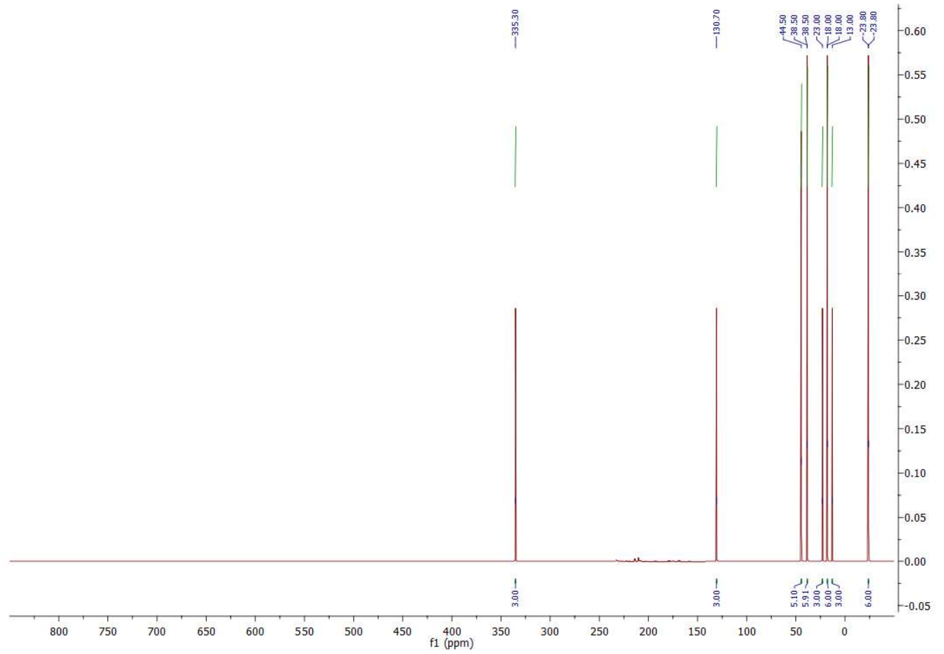

Figure S51. The ^{17}O NMR spectrum of **R8**.

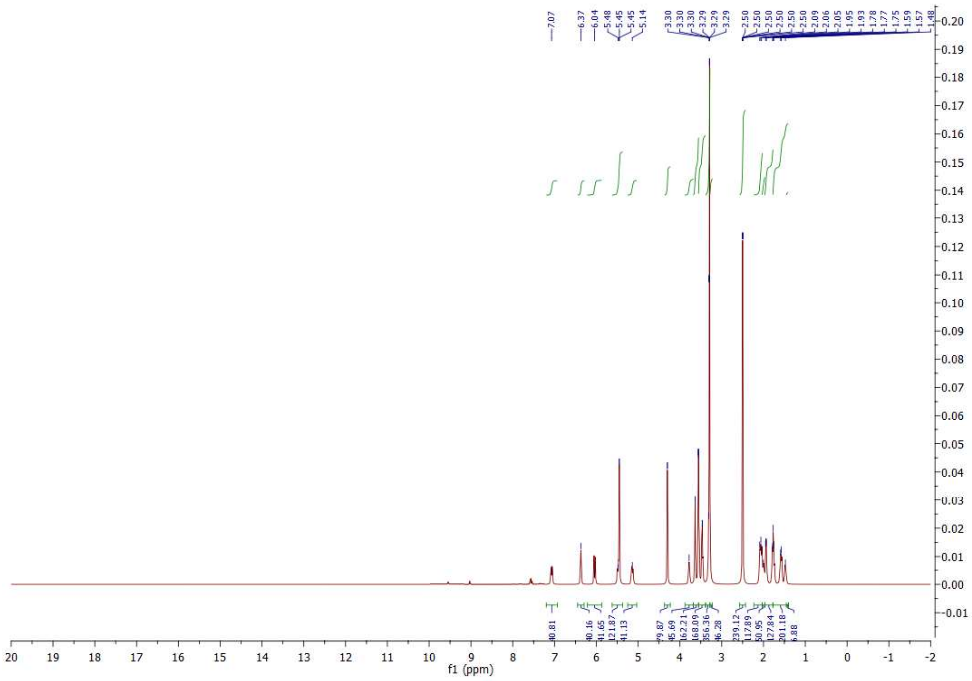

Figure S52. The ^{1}H NMR spectrum of **BFA-D1**.

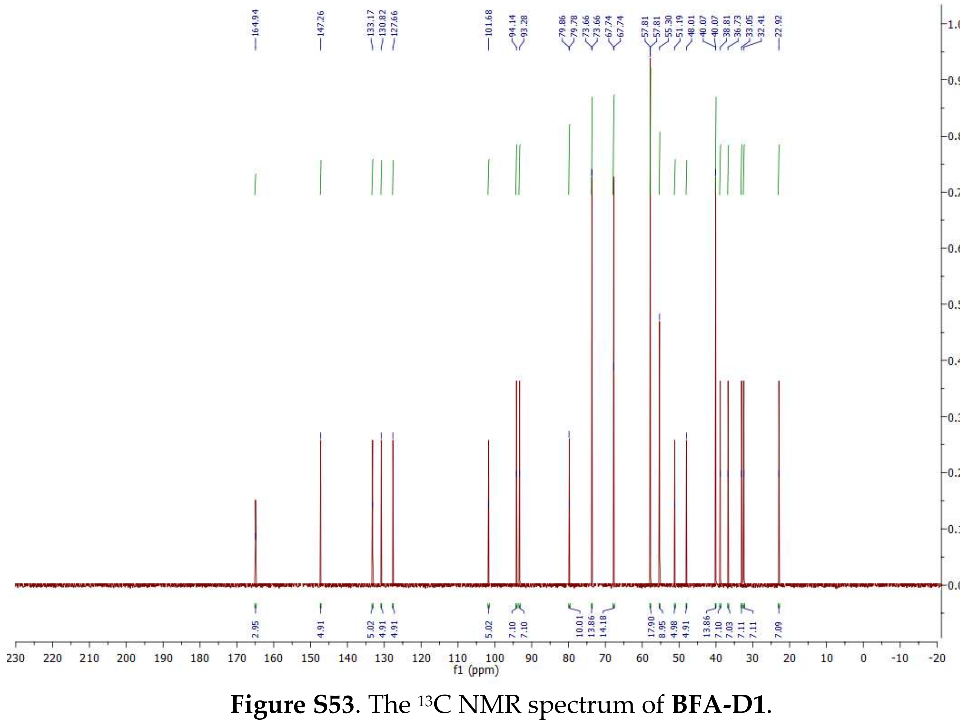

Figure S53. The ^{13}C NMR spectrum of **BFA-D1**.

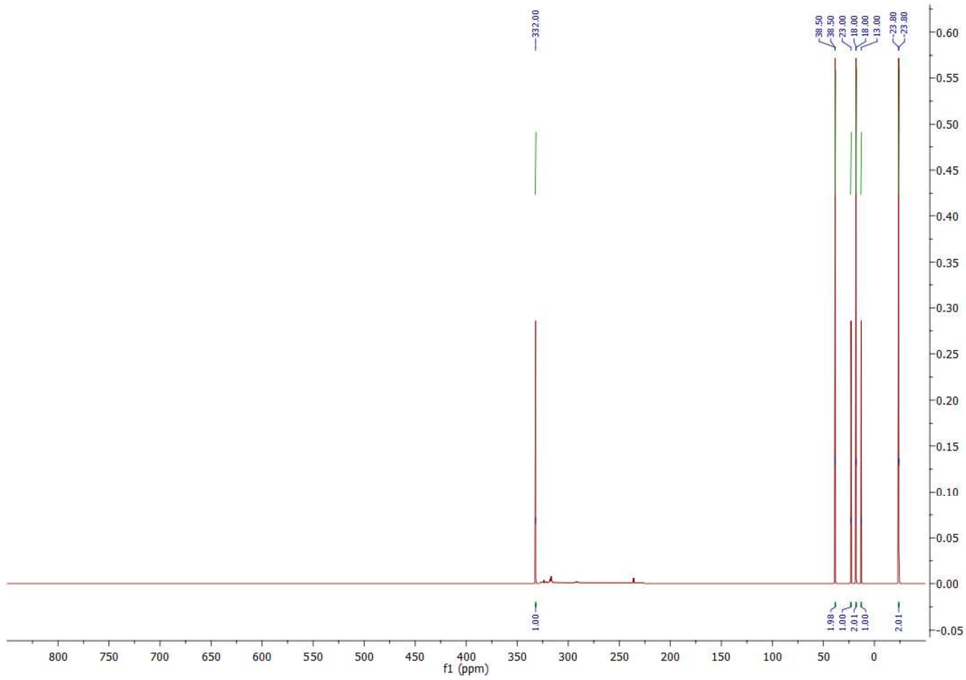

Figure S54. The ^{17}O NMR spectrum of **BFA-D1**.

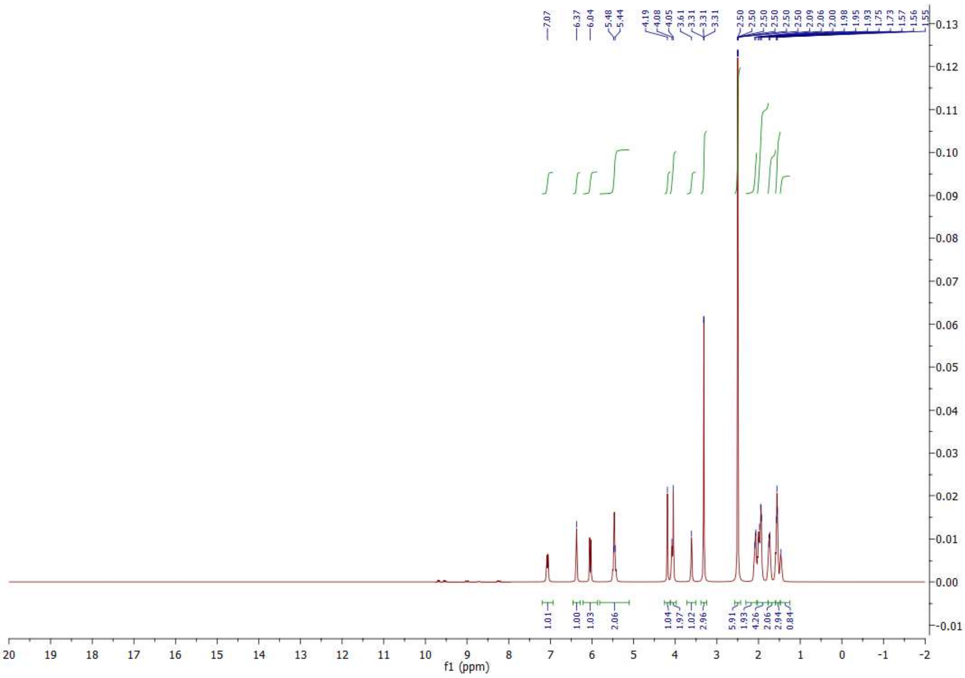

Figure S55. The ^{1}H NMR spectrum of **BFA-D2**.

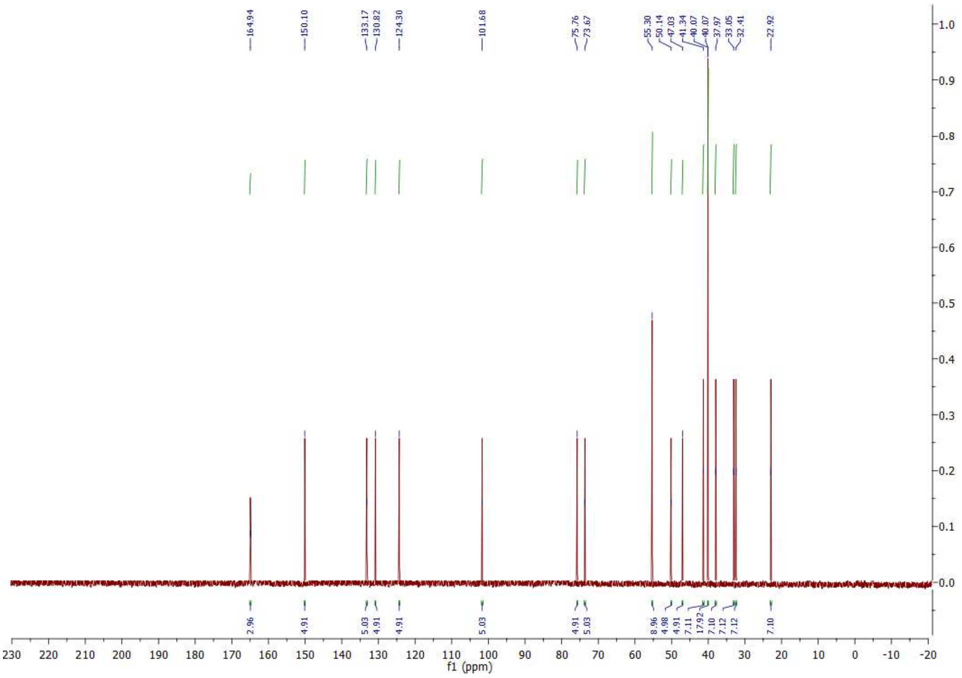

Figure S56. The ^{13}C NMR spectrum of **BFA-D2**.

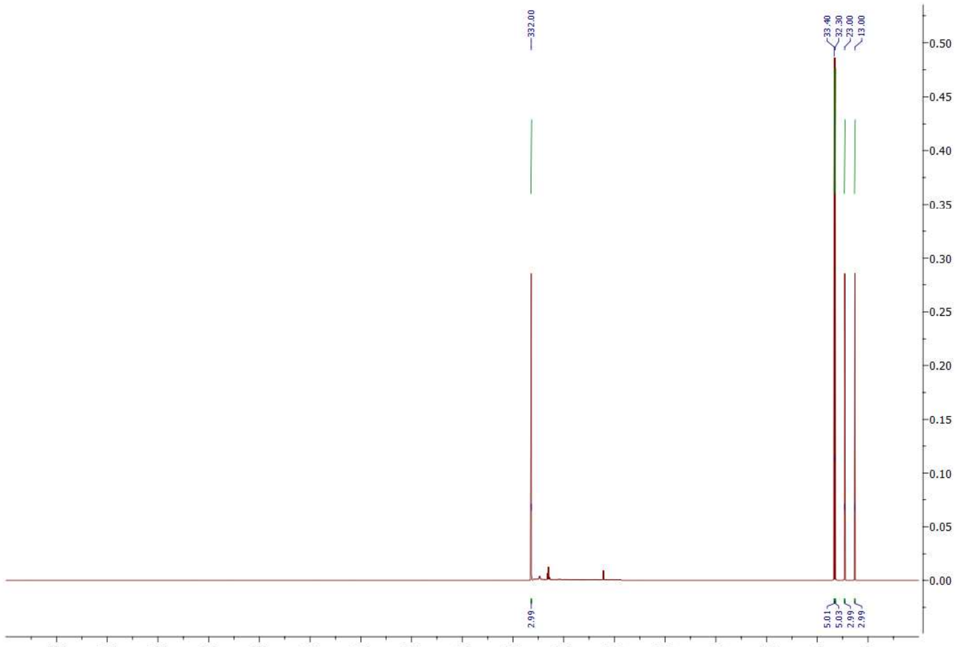

Figure S57. The ^{17}O NMR spectrum of **BFA-D2**.

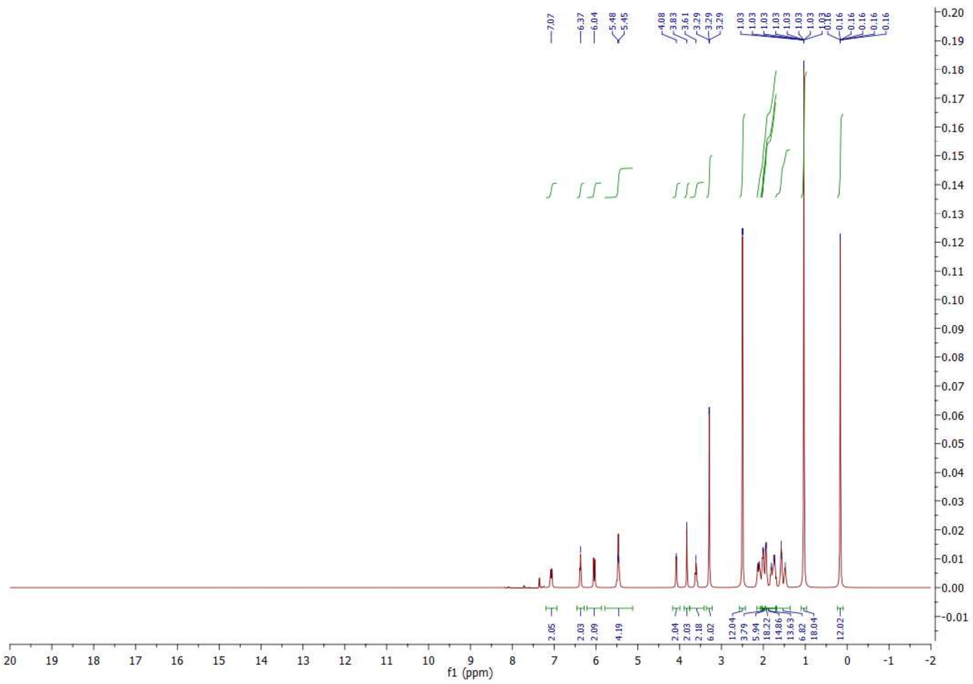

Figure S58. The ^{1}H NMR spectrum of **BFA-D3**.

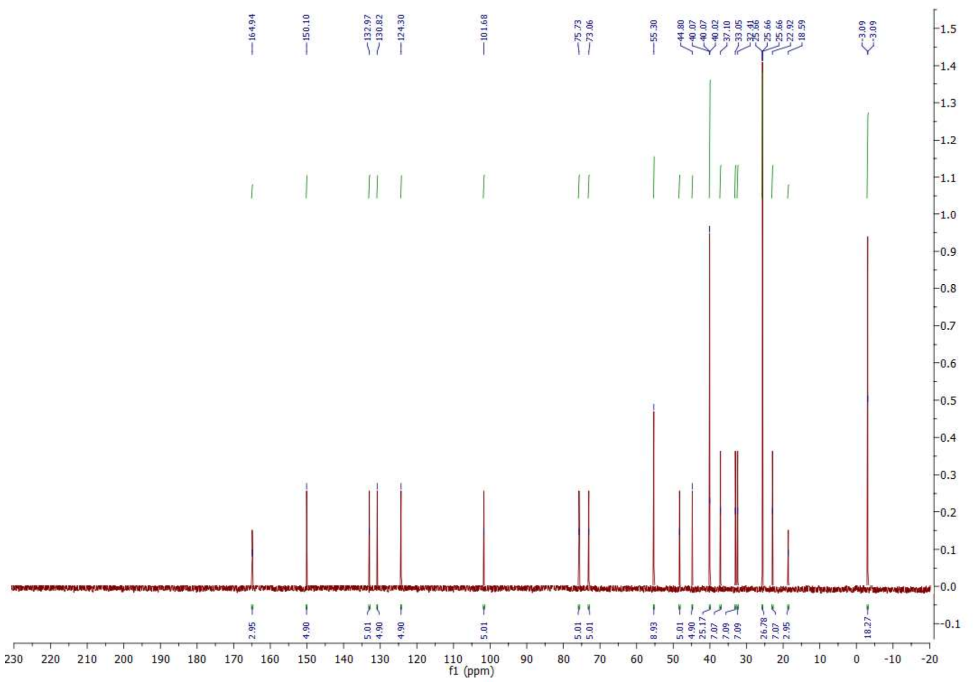

Figure S59. The ^{13}C NMR spectrum of **BFA-D3**.

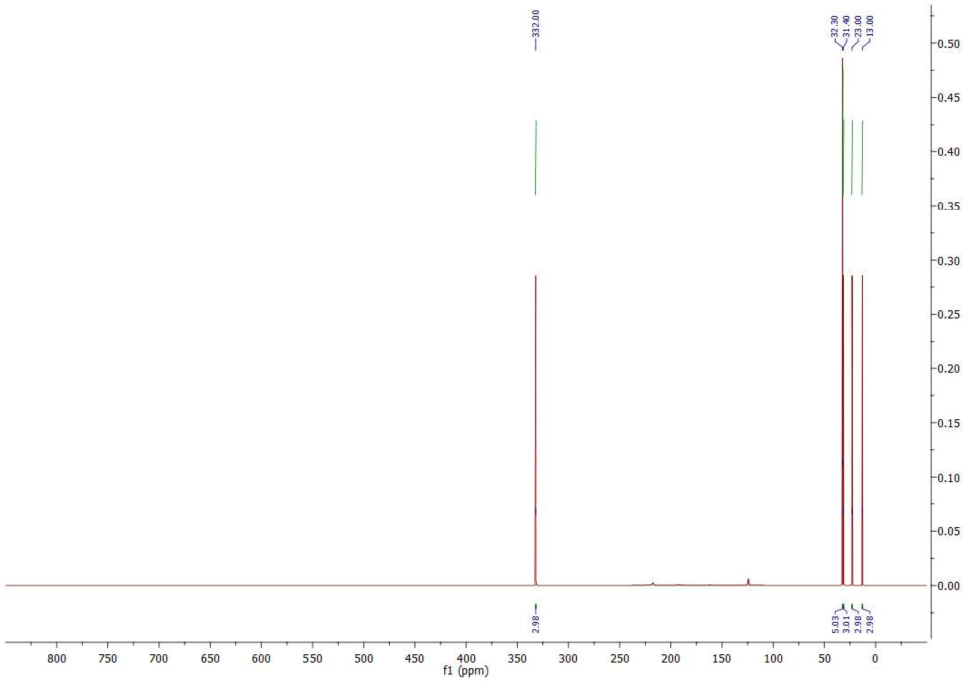

Figure S60. The ^{17}O NMR spectrum of **BFA-D3**.

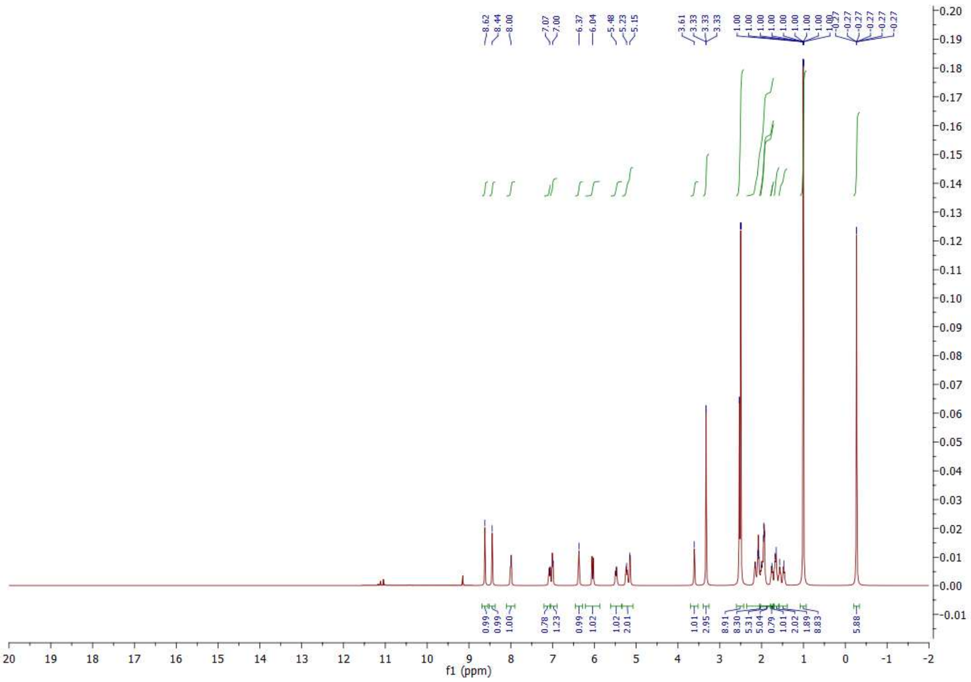

Figure S61. The ^{1}H NMR spectrum of **BFA-D4**.

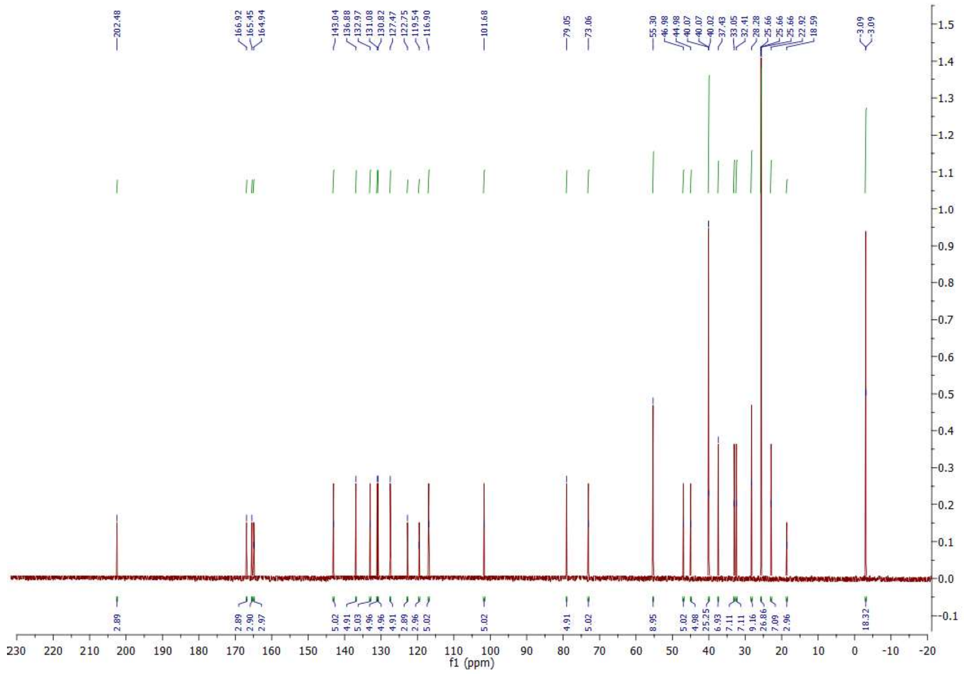

Figure S62. The ^{13}C NMR spectrum of **BFA-D4**.

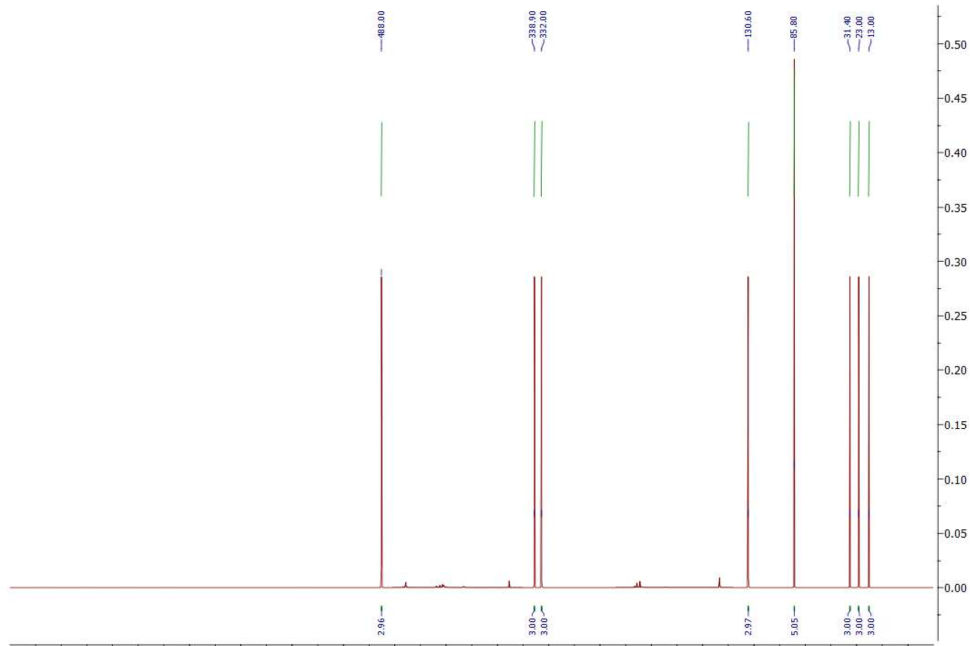

Figure S63. The ^{17}O NMR spectrum of **DFA-D4**.

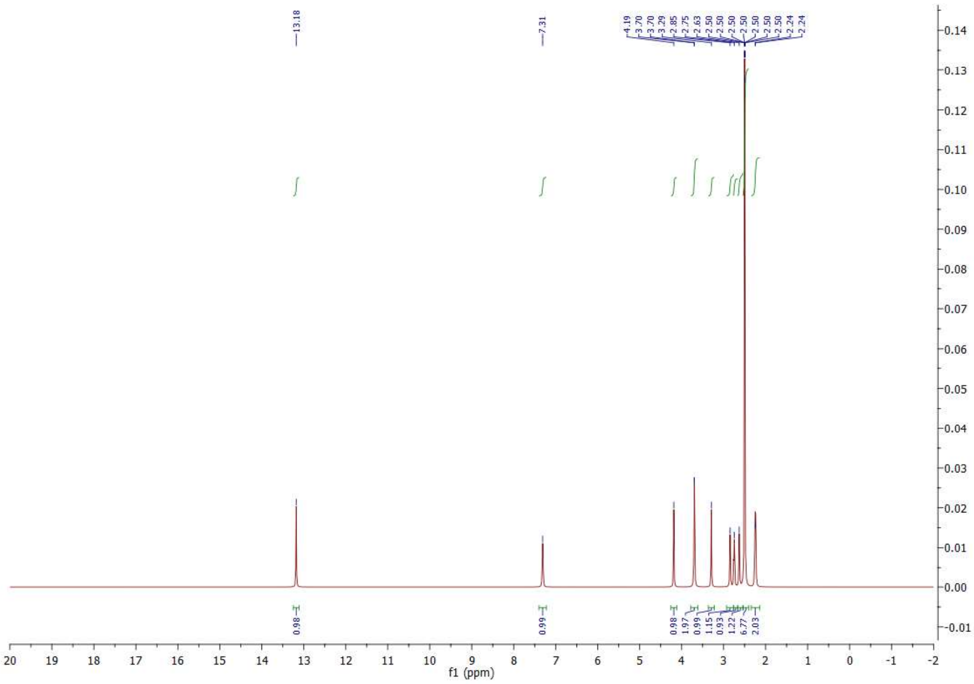

Figure S64. The ^{1}H NMR spectrum of **Pro-R3**.

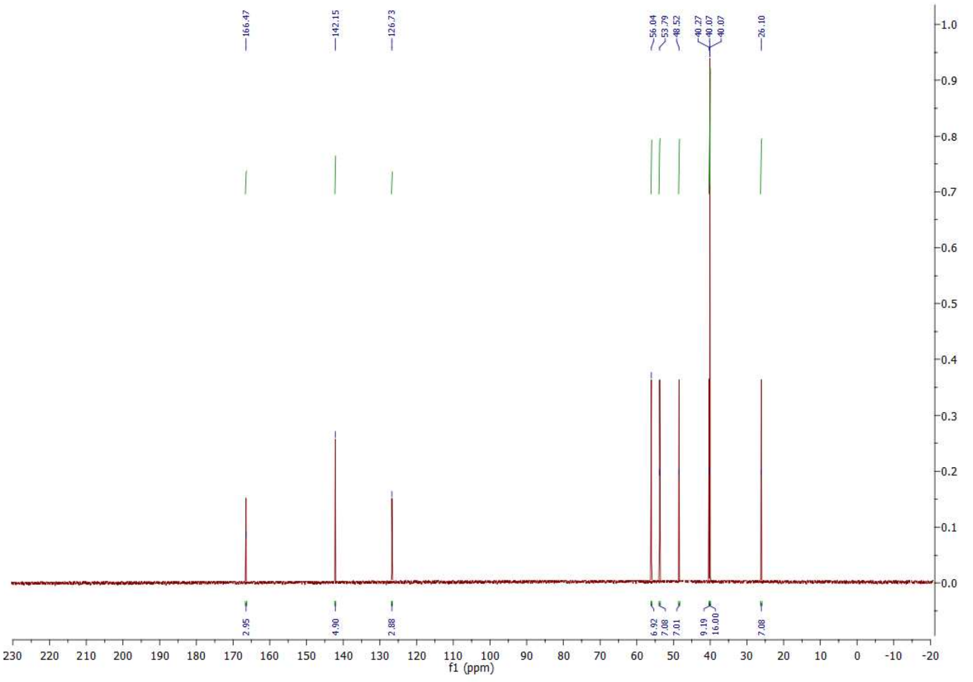

Figure S65. The ^{13}C NMR spectrum of **Pro-R3**.

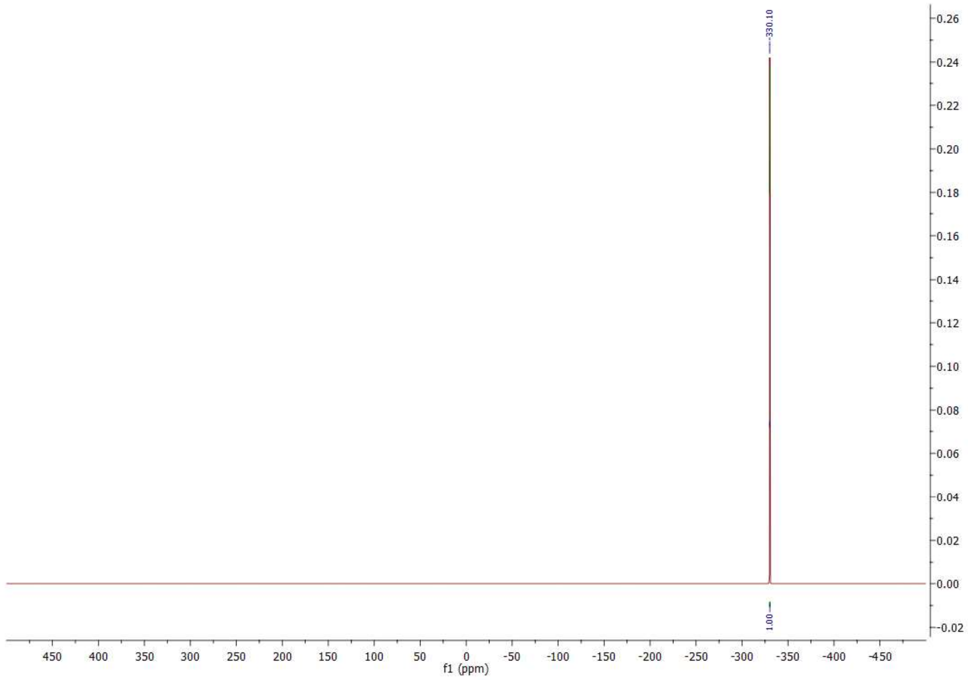

Figure S66. The ^{15}N NMR spectrum of **Pro-R3**.

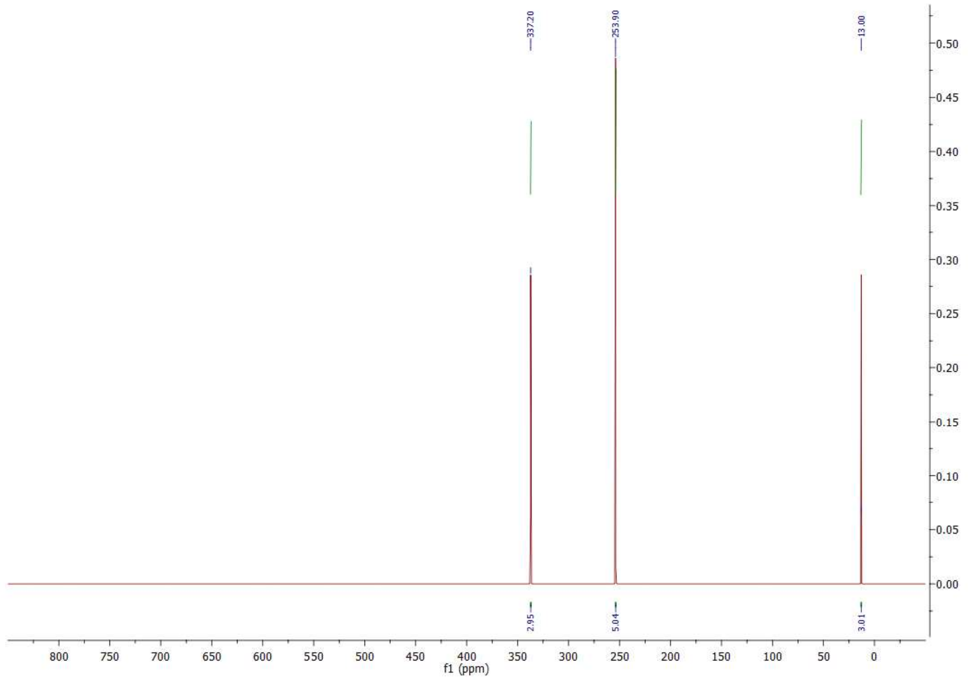

Figure S67. The ^{17}O NMR spectrum of **Pro-R3**.

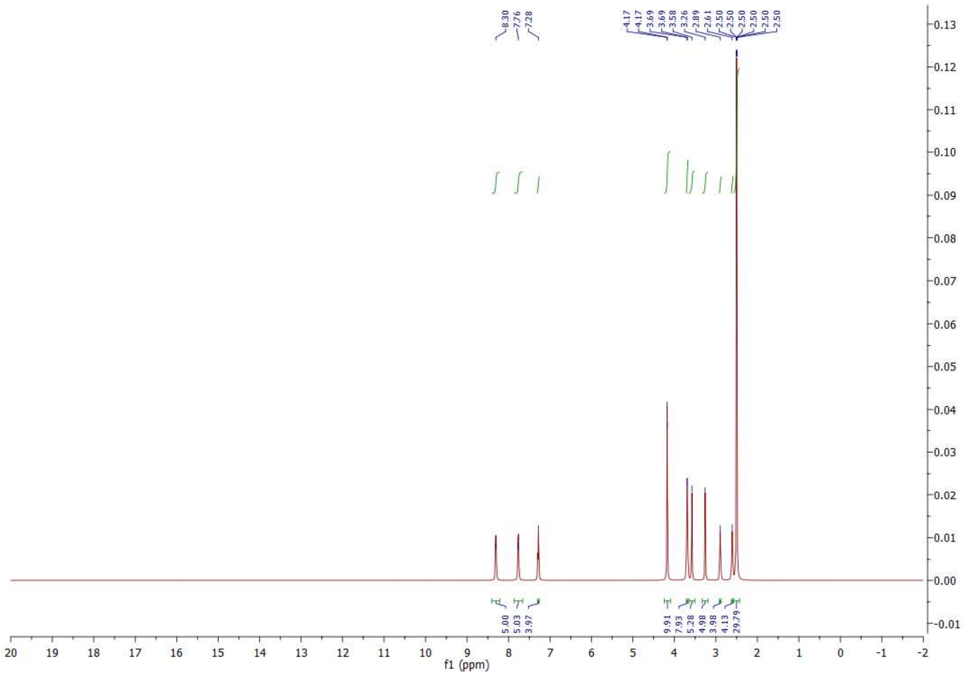

Figure S68. The ^1H NMR spectrum of **Pro-R6**.

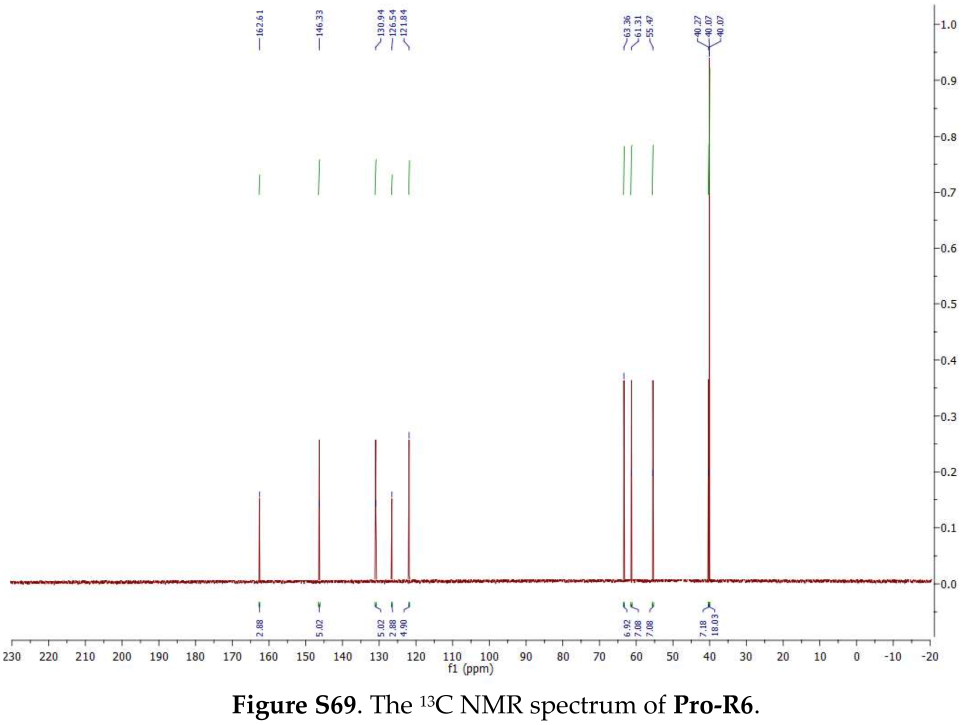

Figure S69. The ^{13}C NMR spectrum of **Pro-R6**.

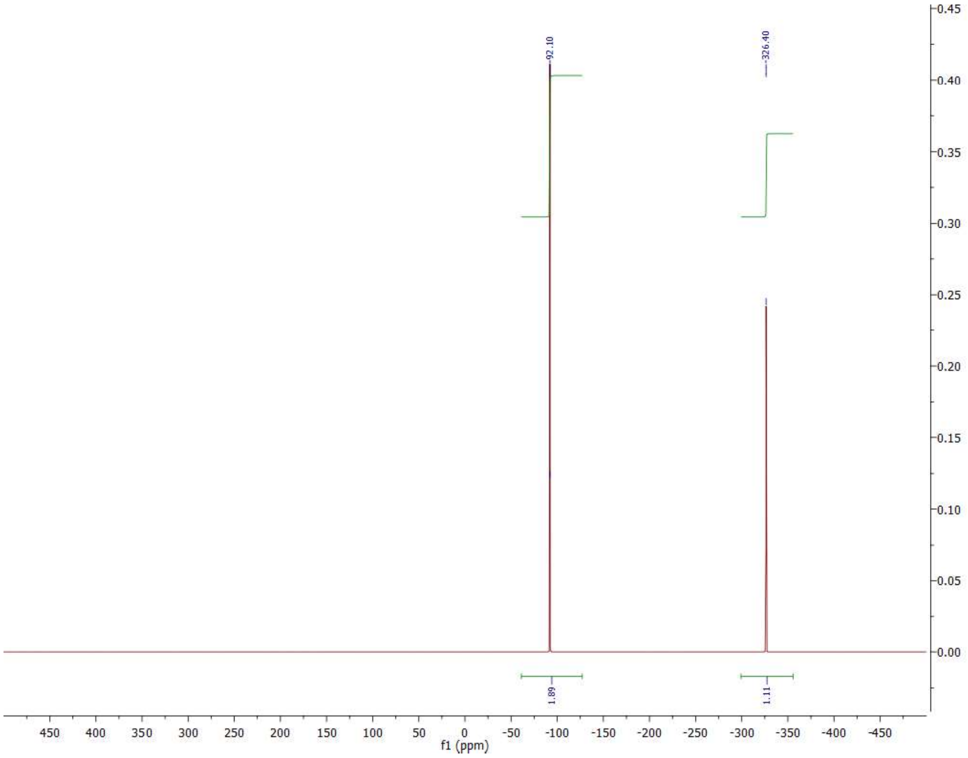

Figure S70. The ^{15}N NMR spectrum of **Pro-R6**.

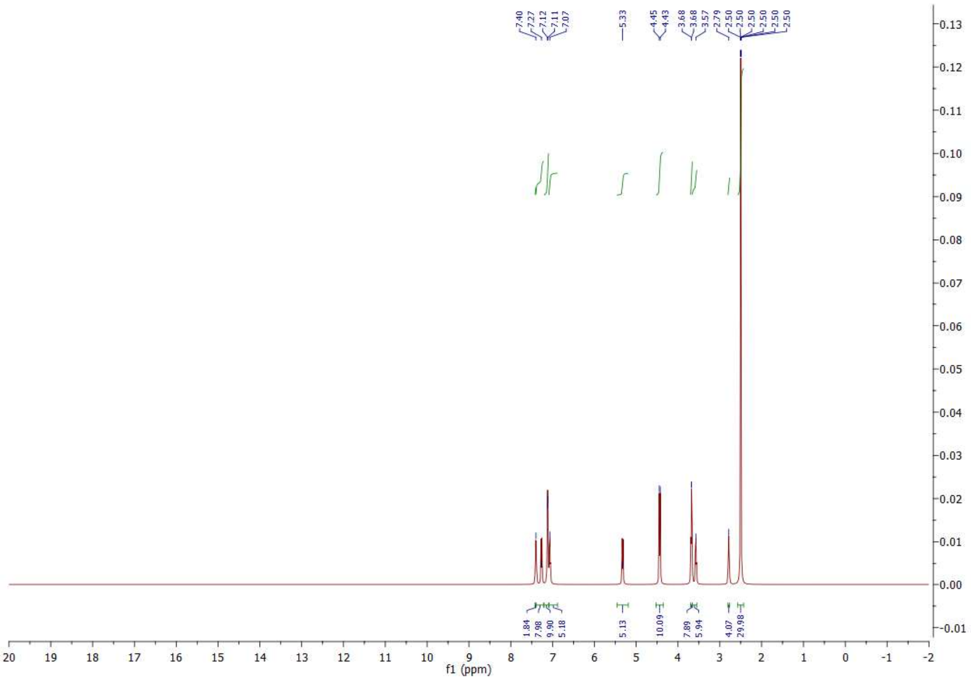

Figure S71. The ^{1}H NMR spectrum of **Pro-R7**.

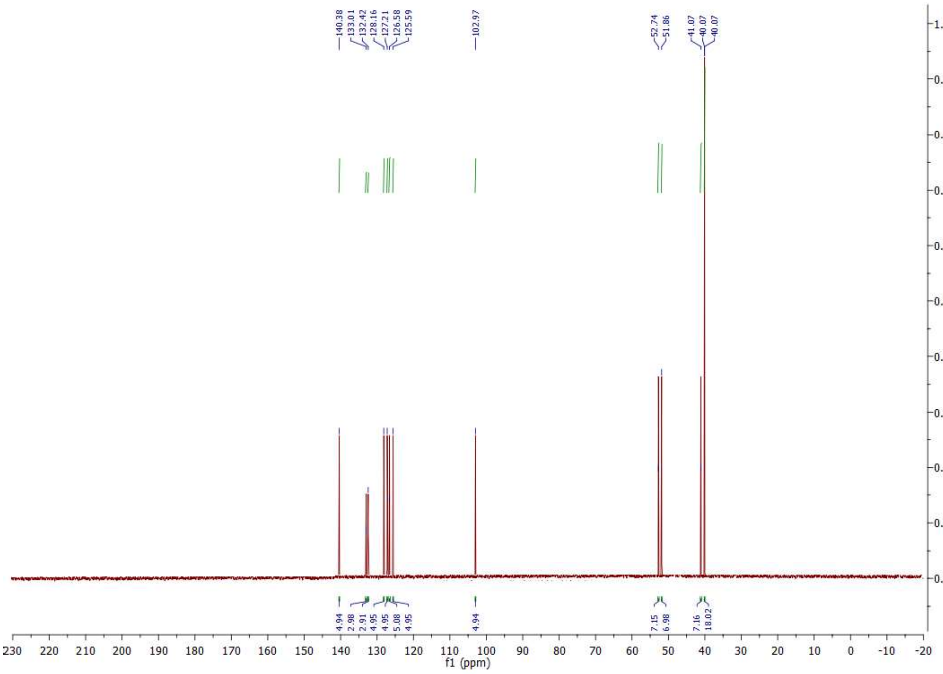

Figure S72. The ^{13}C NMR spectrum of **Pro-R7**.

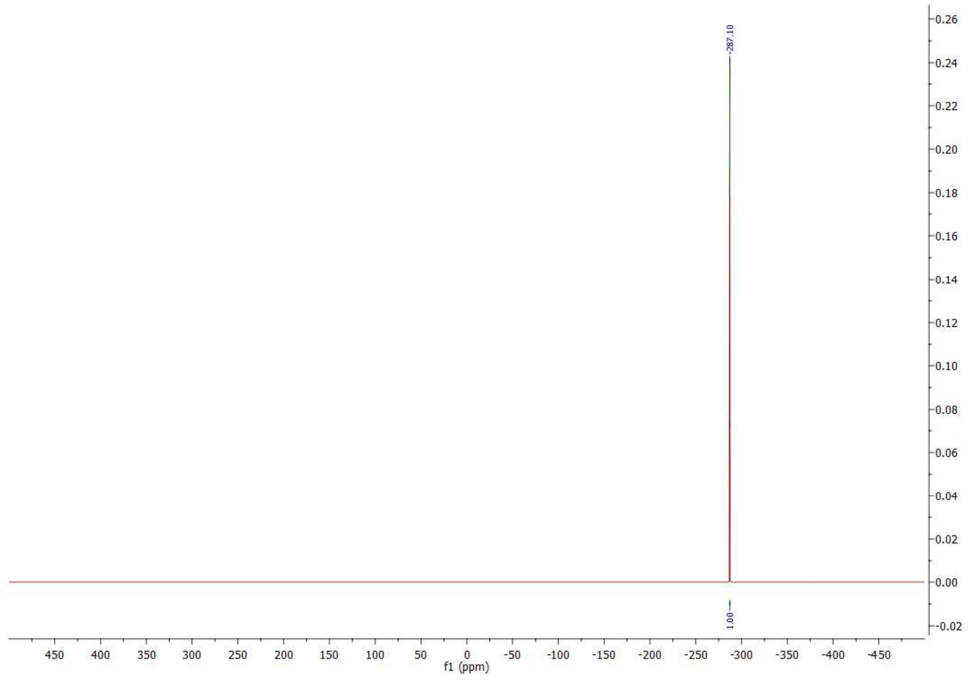

Figure S73. The ^{15}N NMR spectrum of **Pro-R7**.

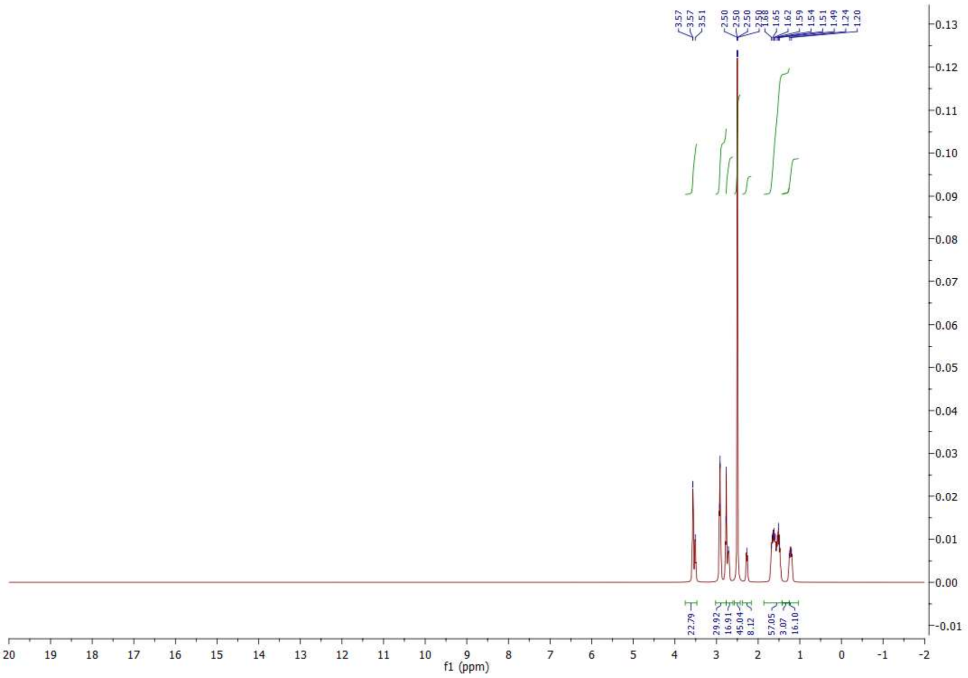

Figure S74. The ¹H NMR spectrum of **Pro-R8**.

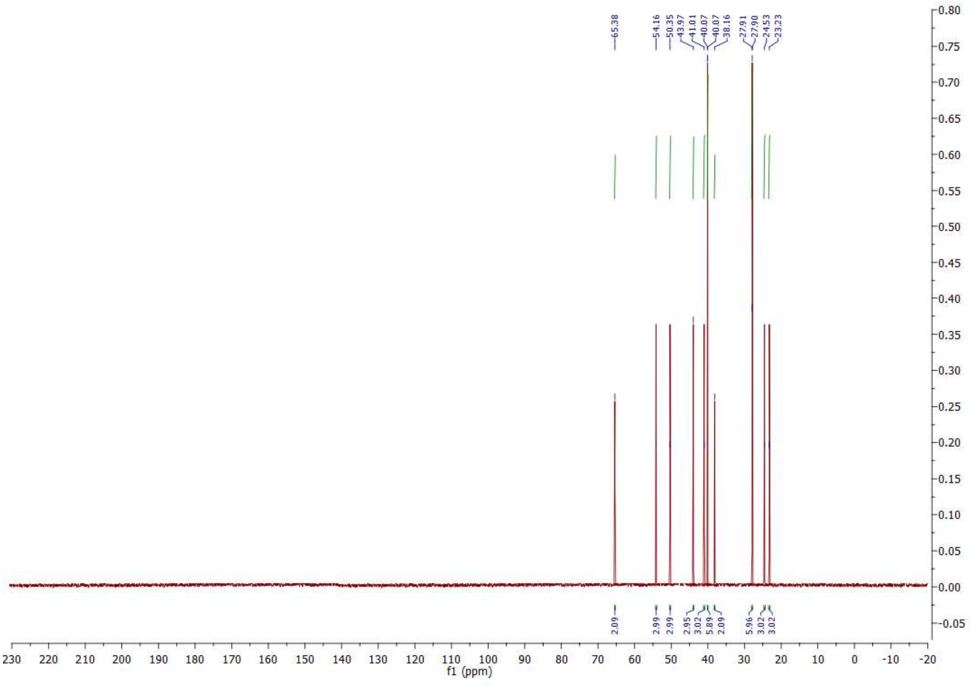

Figure S75. The ¹³C NMR spectrum of **Pro-R8**.

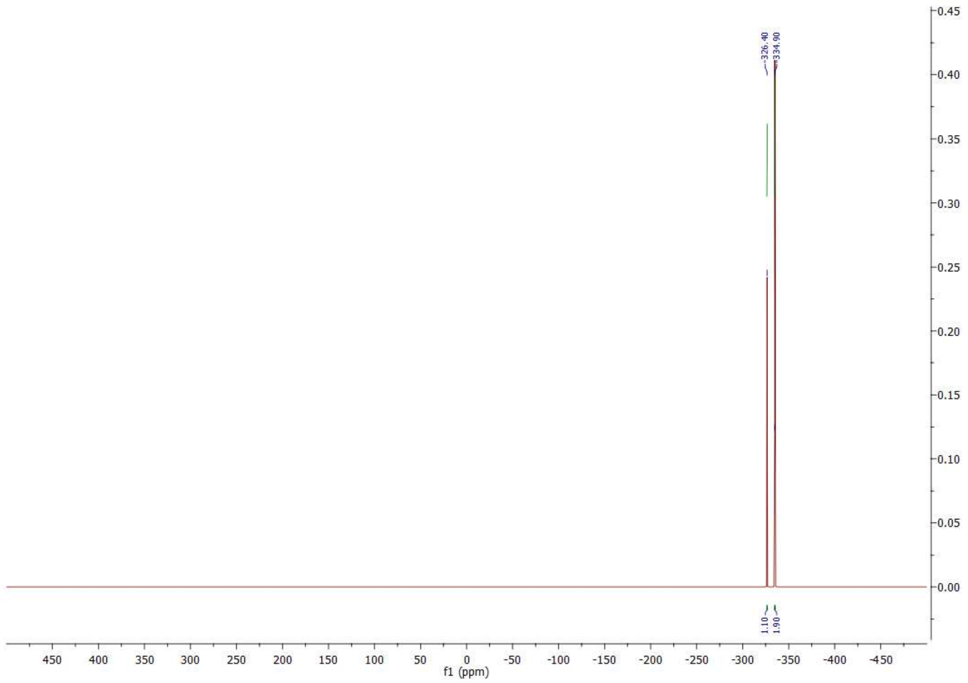

Figure S76. The ^{15}N NMR spectrum of **Pro-R8**.

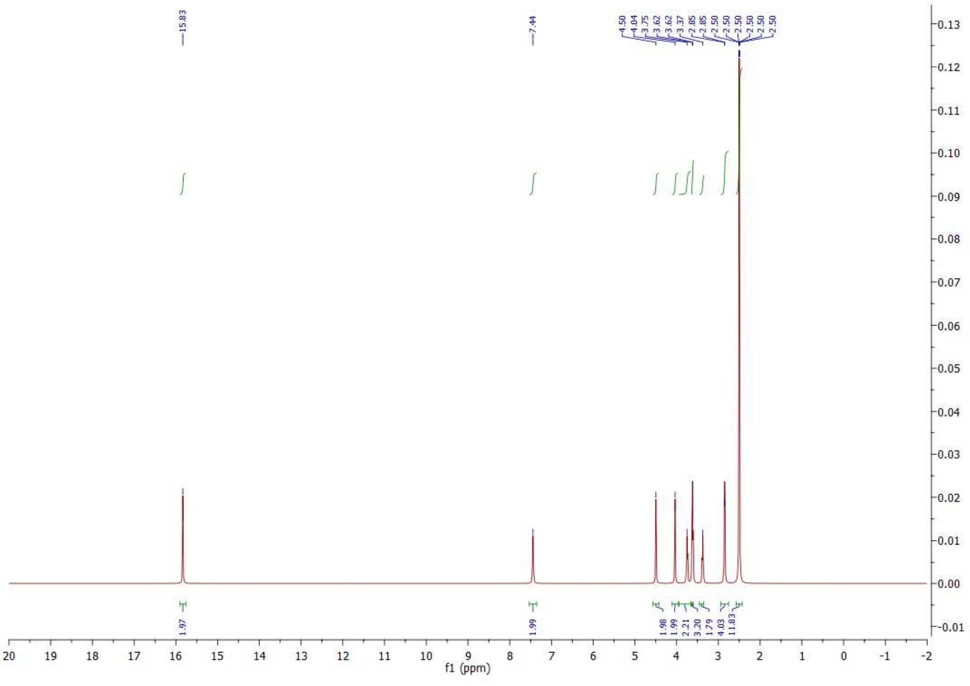

Figure S77. The ^{1}H NMR spectrum of **Pro-R9**.

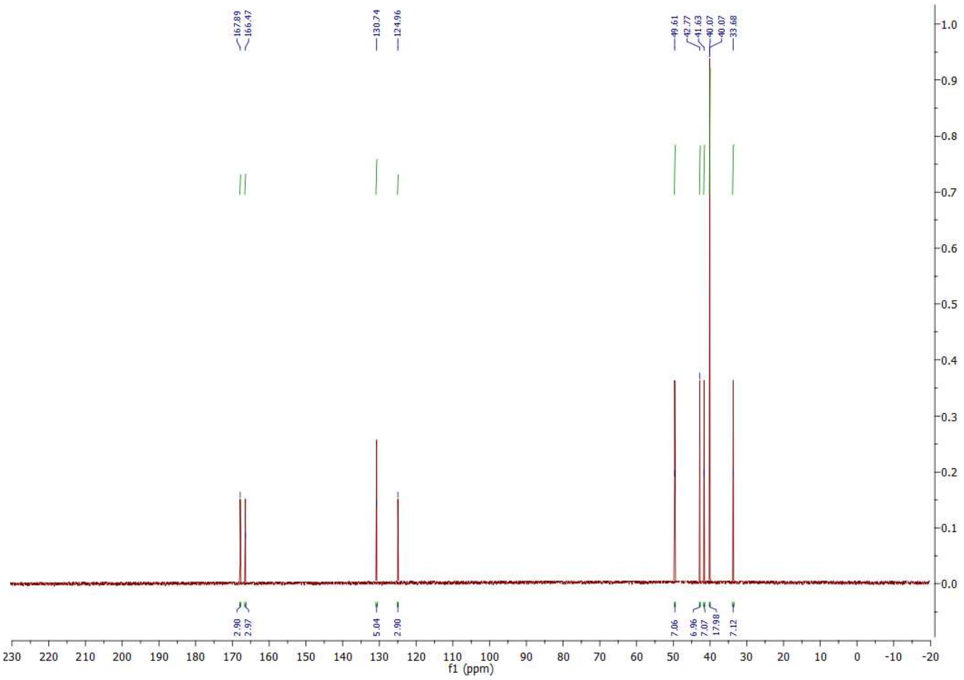

Figure S78. The ^{13}C NMR spectrum of **Pro-R9**.

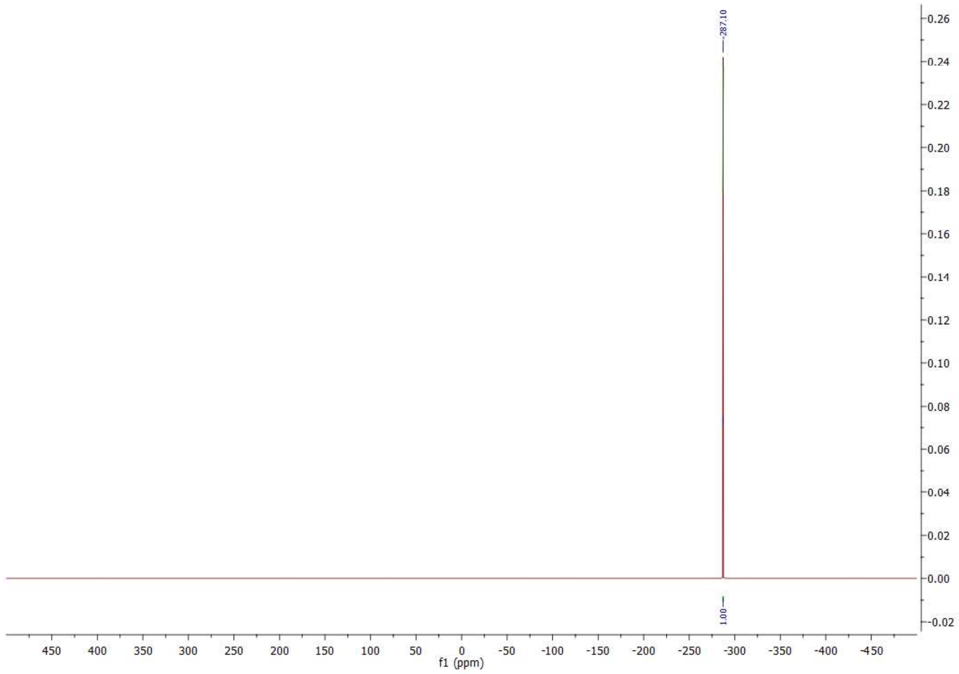

Figure S79. The ^{15}N NMR spectrum of **Pro-R9**.

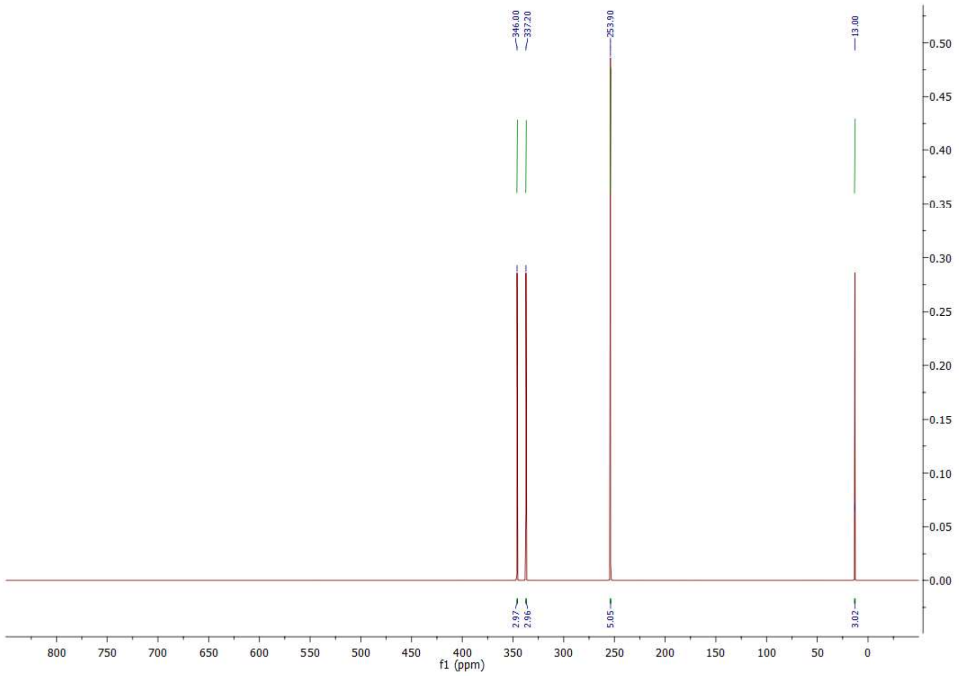

Figure S80. The ^{17}O NMR spectrum of **Pro-R9**.

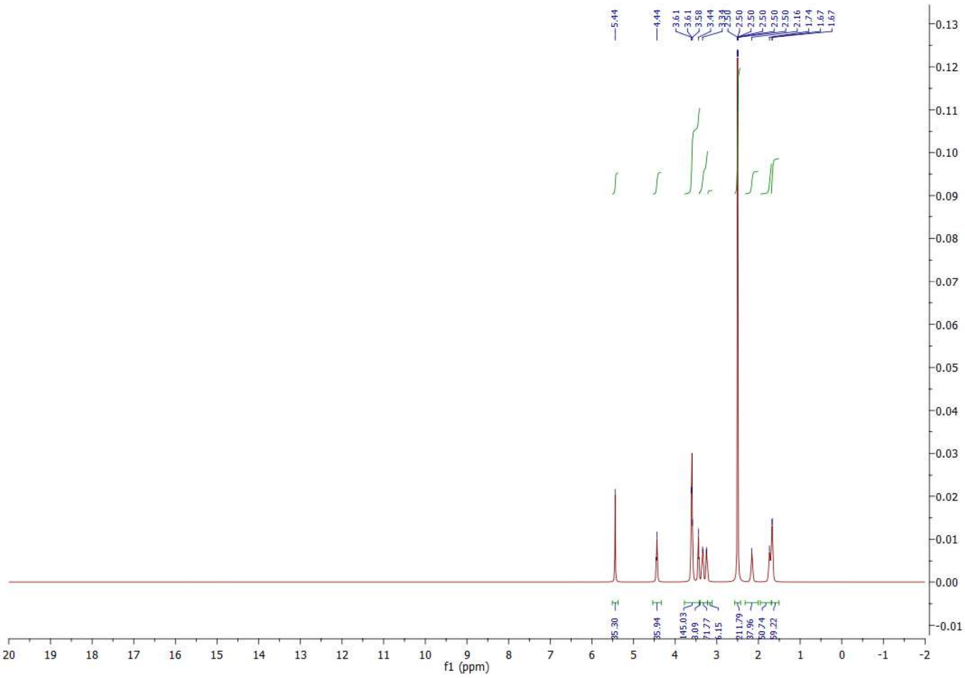

Figure S81. The ^{1}H NMR spectrum of **Pro-R12**.

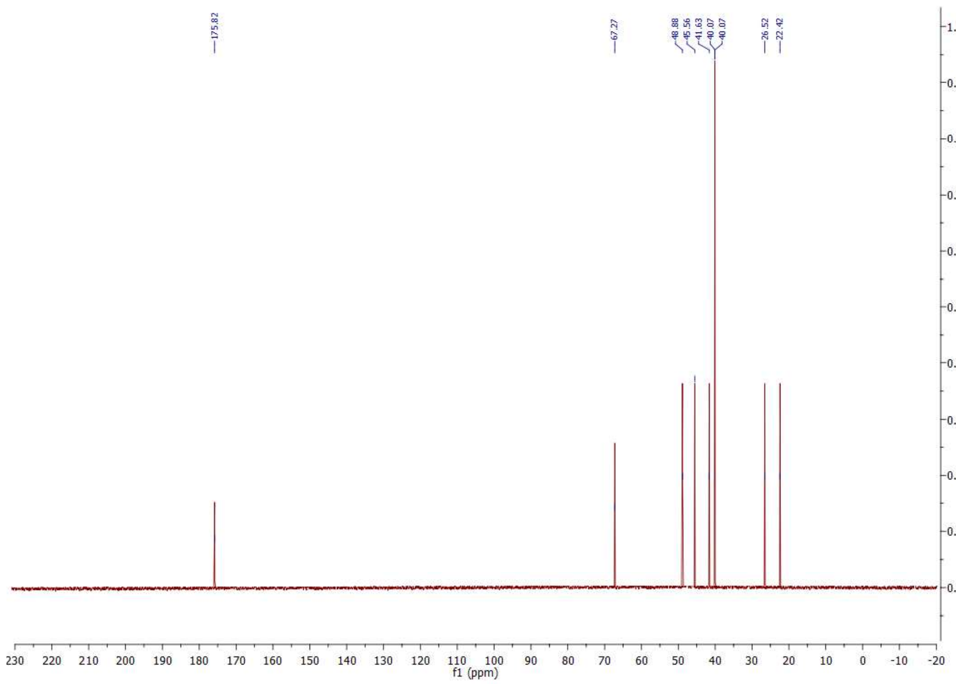

Figure S82. The ^{13}C NMR spectrum of **Pro-R12**.

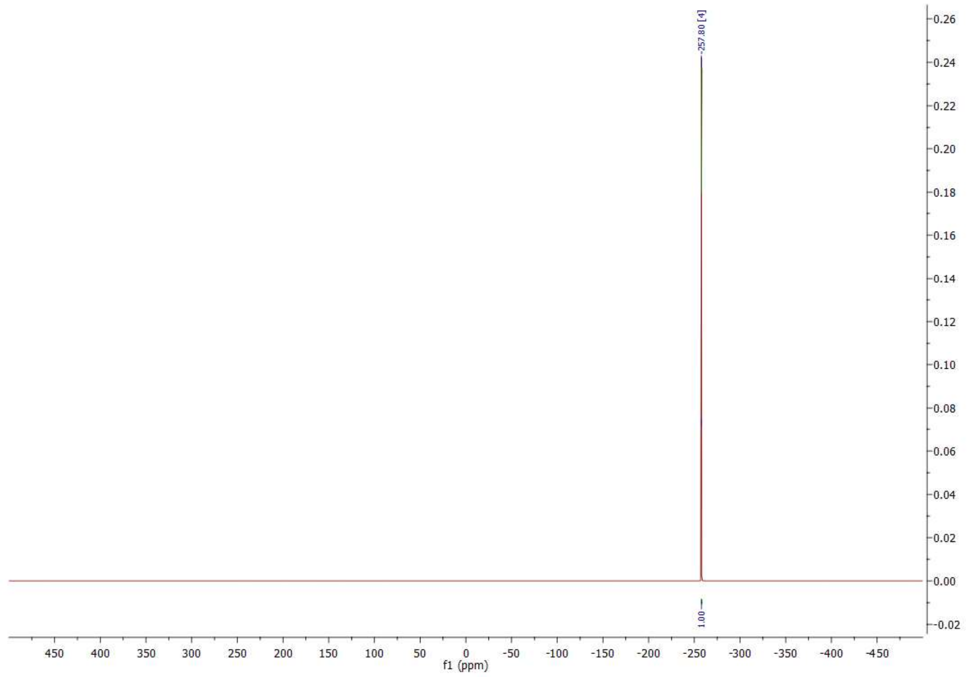

Figure S83. The ^{15}N NMR spectrum of **Pro-R12**.

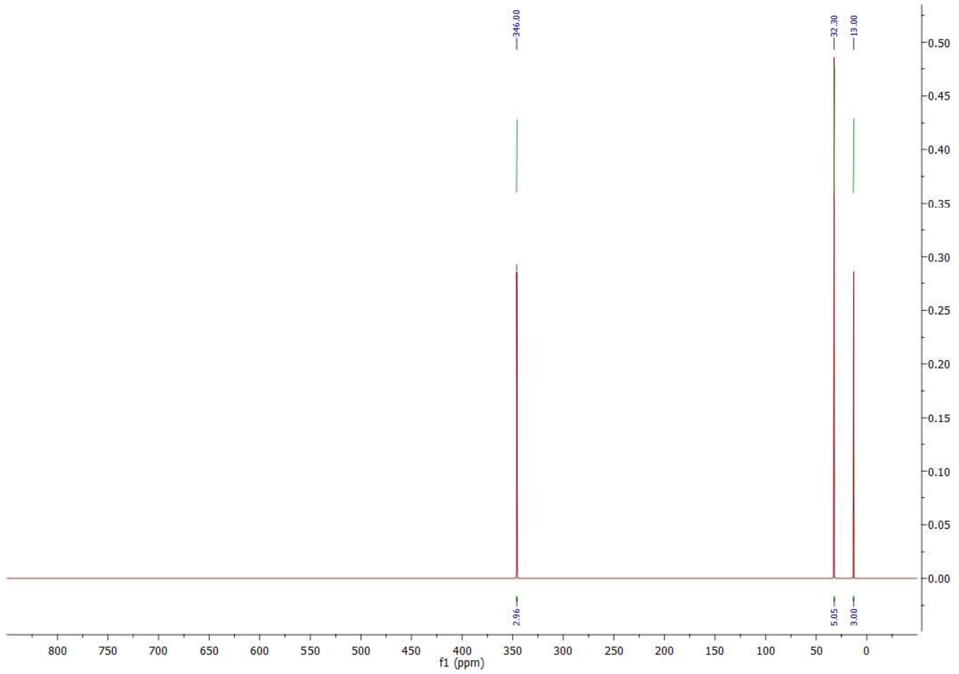

Figure S84. The ^{17}O NMR spectrum of **Pro-R12**.

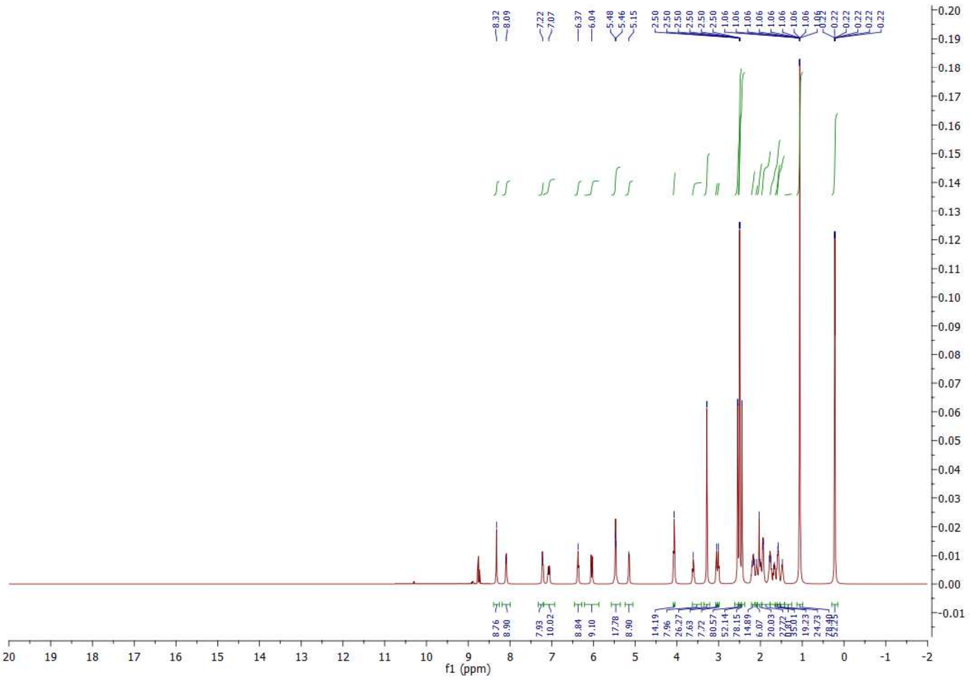

Figure S85. The ^{1}H NMR spectrum of **Pro-3DPQ-1**.

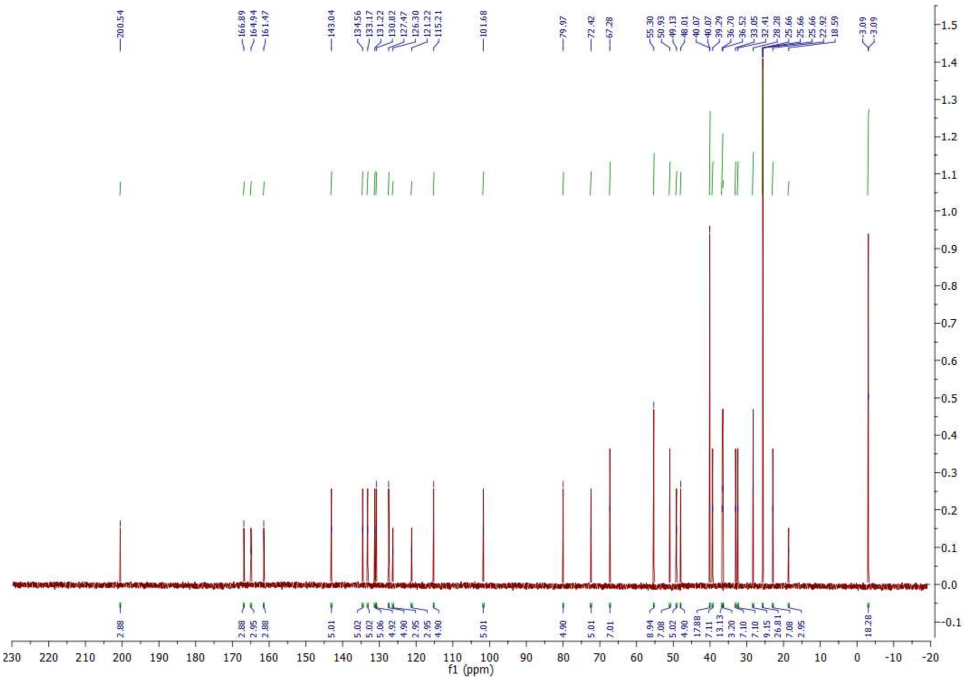

Figure S86. The ^{13}C NMR spectrum of **Pro-3DPQ-1**.

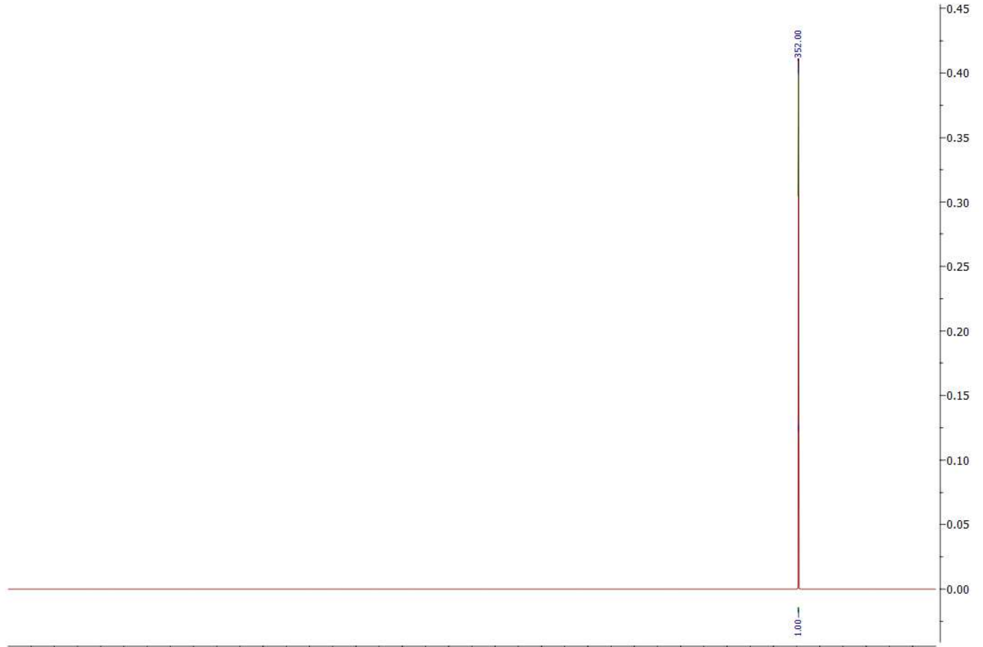

Figure S87. The ^{13}N NMR spectrum of **Pro-3DPQ-1**.

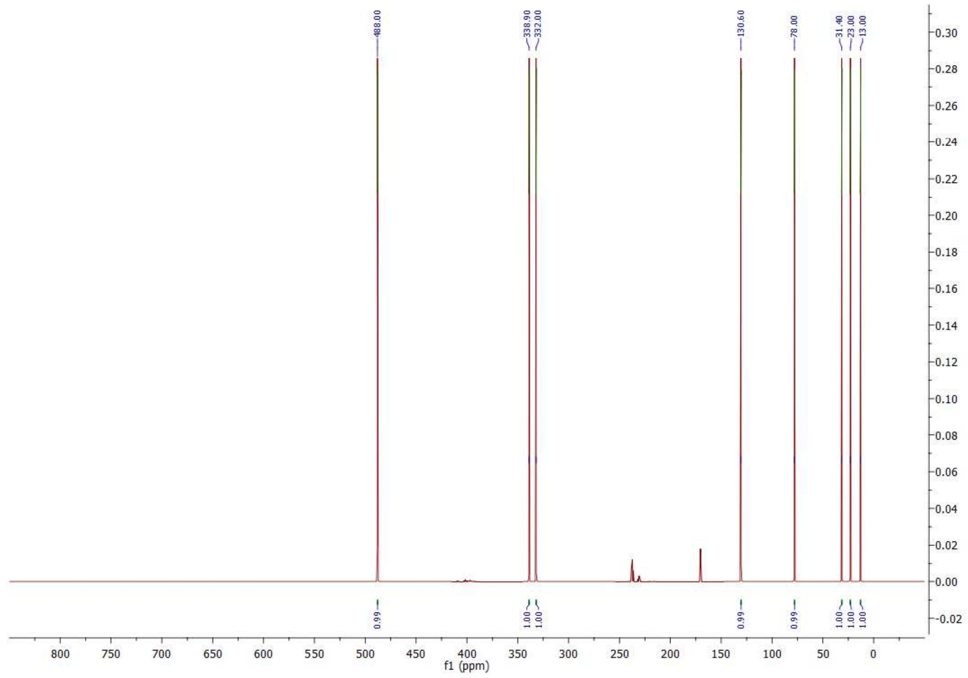

Figure S88. The ^{13}O NMR spectrum of **Pro-3DPQ-1**.

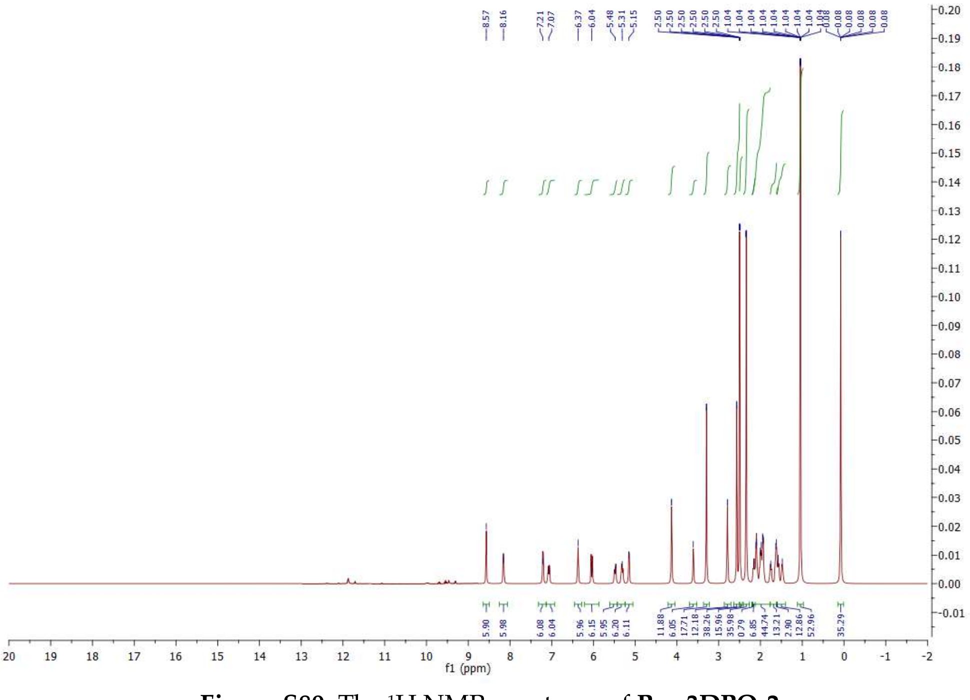

Figure S89. The ^{1}H NMR spectrum of **Pro-3DPQ-2**.

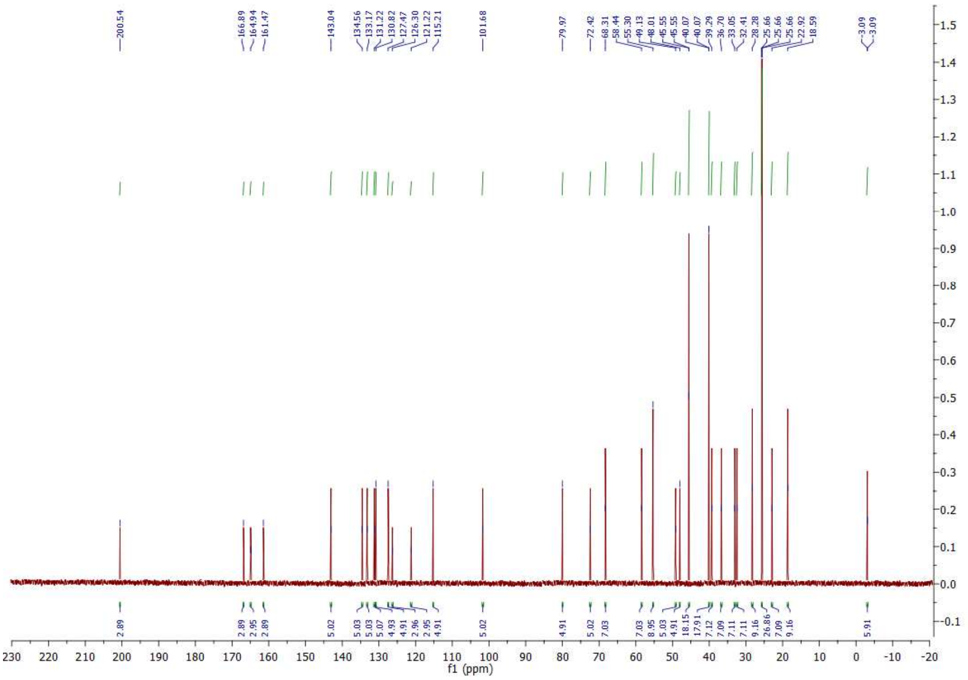

Figure S90. The ^{13}C NMR spectrum of **Pro-3DPQ-2.**

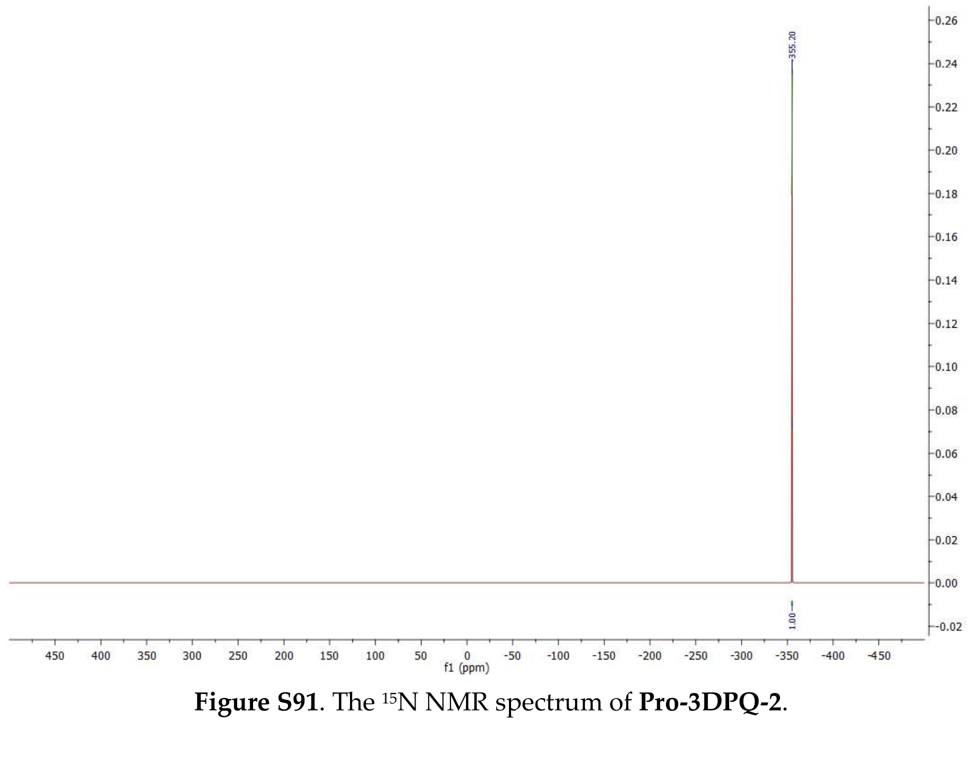

Figure S91. The ^{15}N NMR spectrum of **Pro-3DPQ-2**.

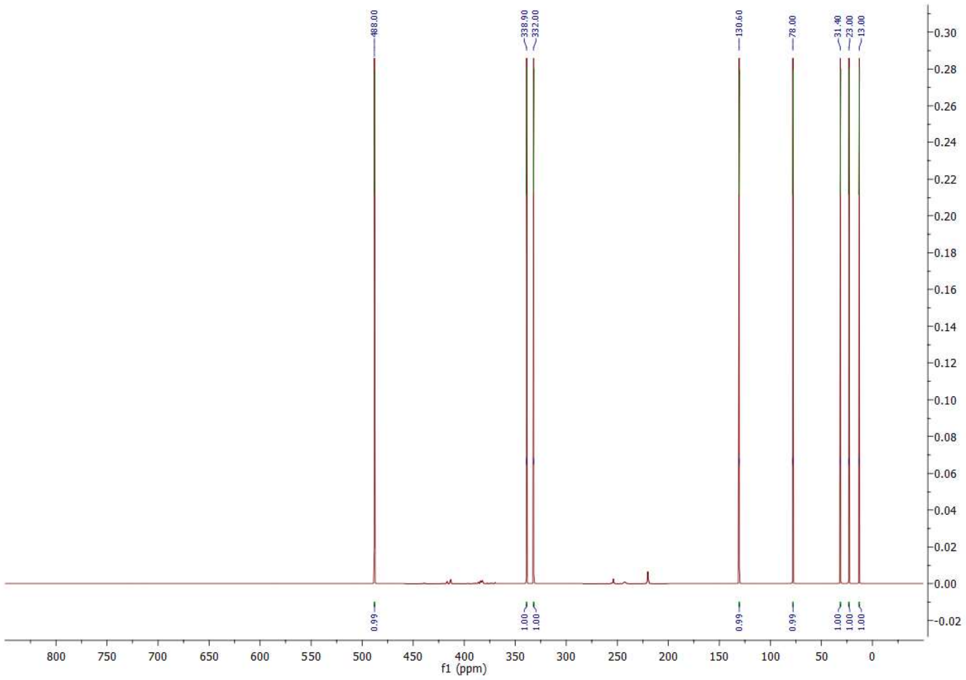

Figure S92. The ^{17}O NMR spectrum of **Pro-3DPQ-2**.

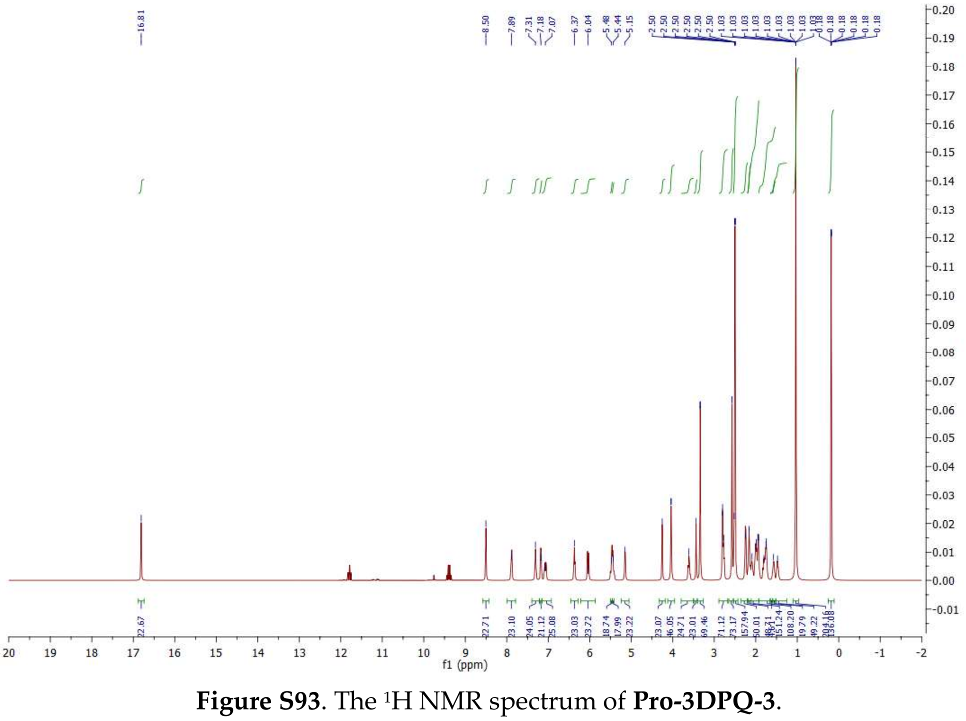

Figure S93. The ^{1}H NMR spectrum of **Pro-3DPQ-3**.

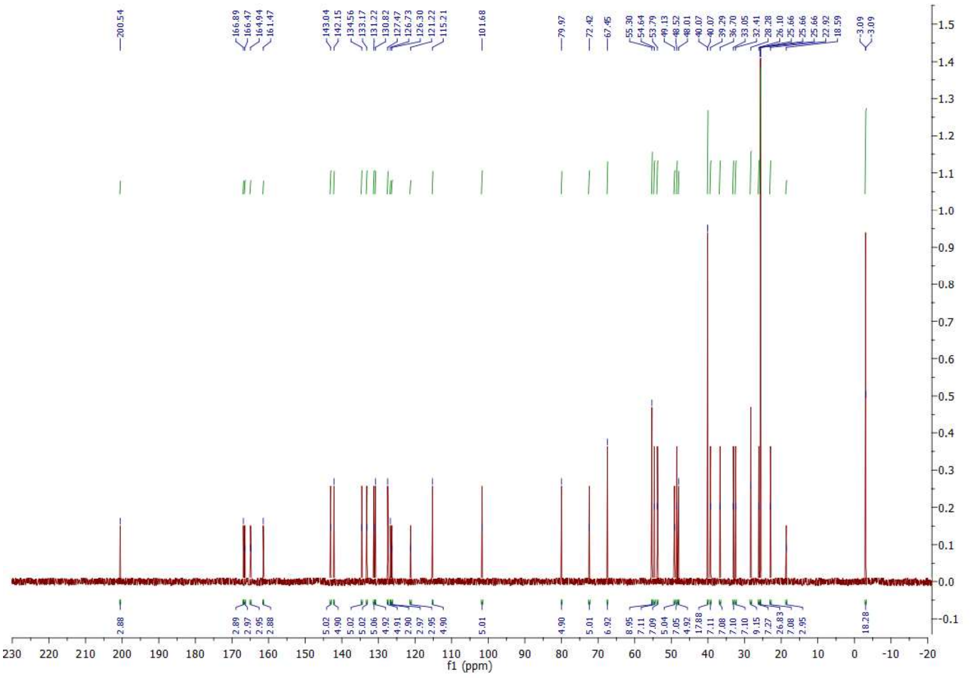

Figure S94. The ^{13}C NMR spectrum of **Pro-3DPQ-3**.

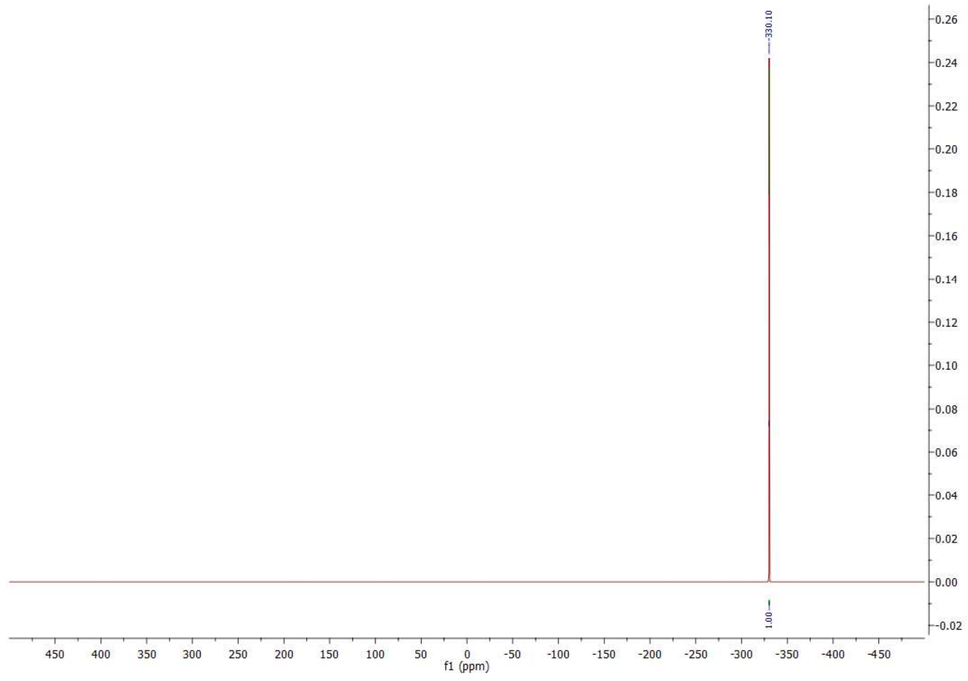

Figure S95. The ^{15}N NMR spectrum of **Pro-3DPQ-3**.

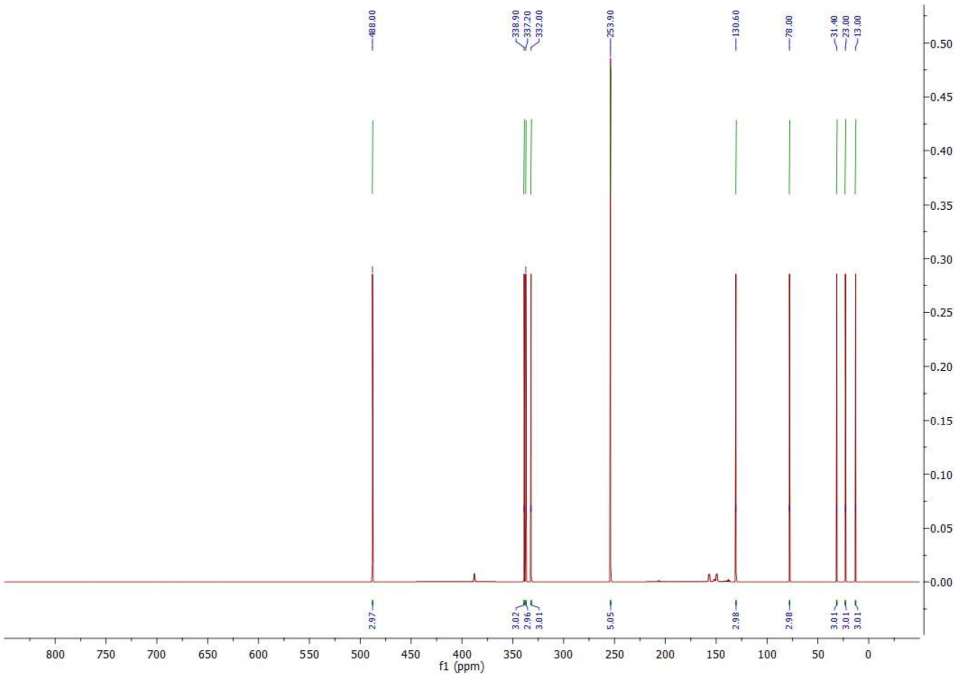

Figure S96. The ^{17}O NMR spectrum of **Pro-3DPQ-3**.

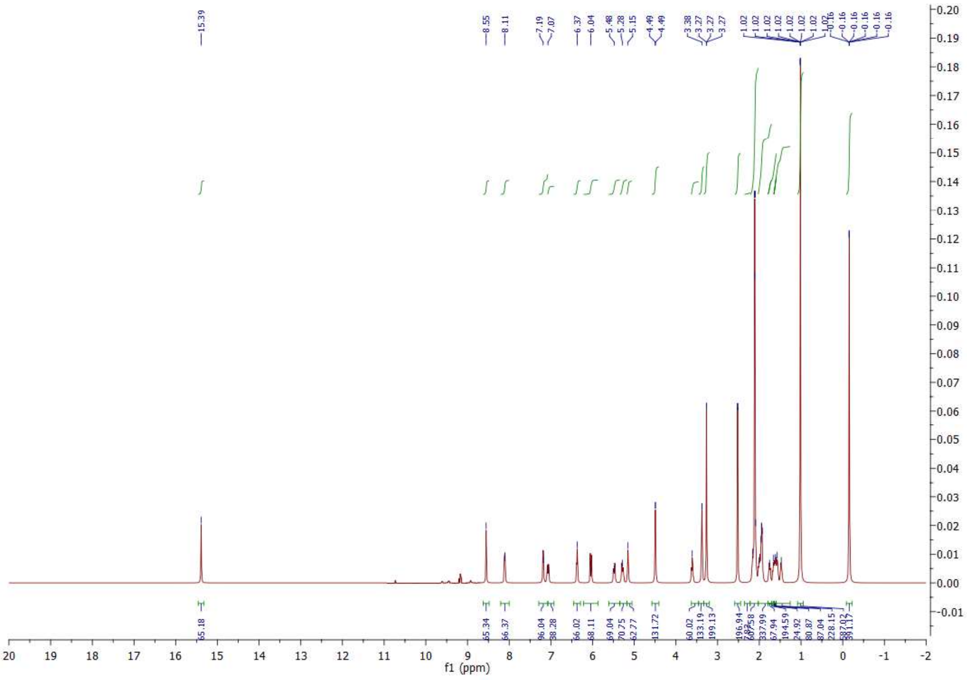

Figure S97. The ^{1}H NMR spectrum of **Pro-3DPQ-4**.

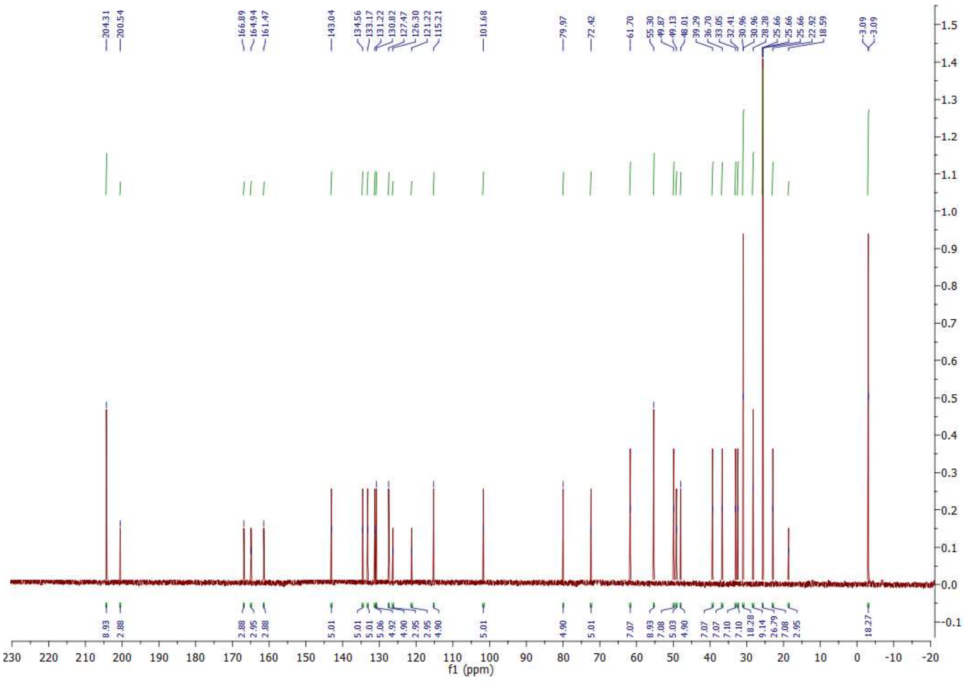

Figure S98. The ^{13}C NMR spectrum of **Pro-3DPQ-4**.

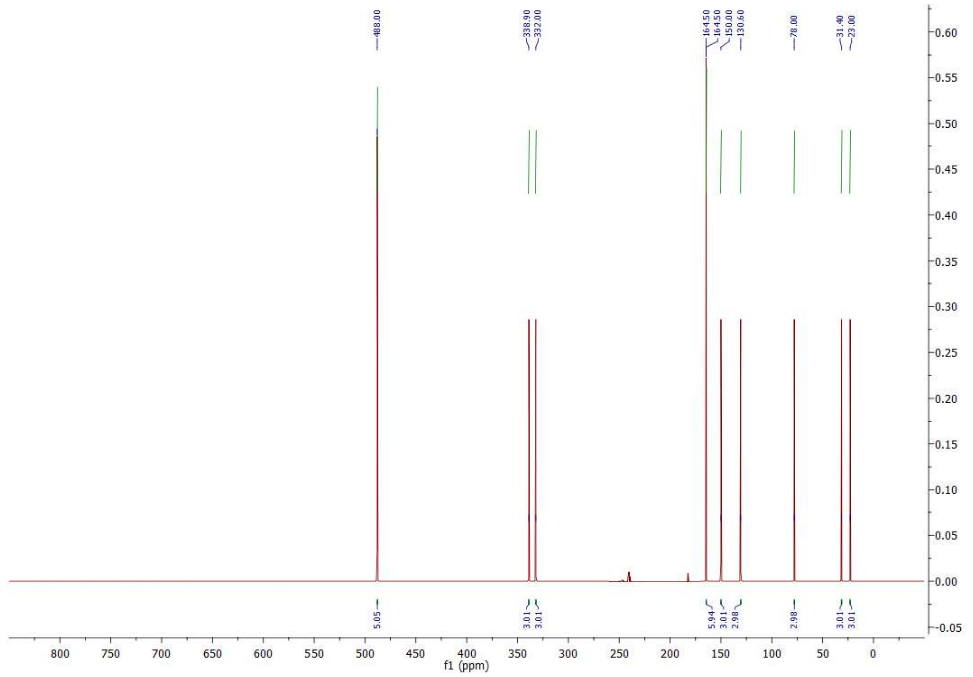

Figure S99. The ^{17}O NMR spectrum of **Pro-3DPQ-4**.

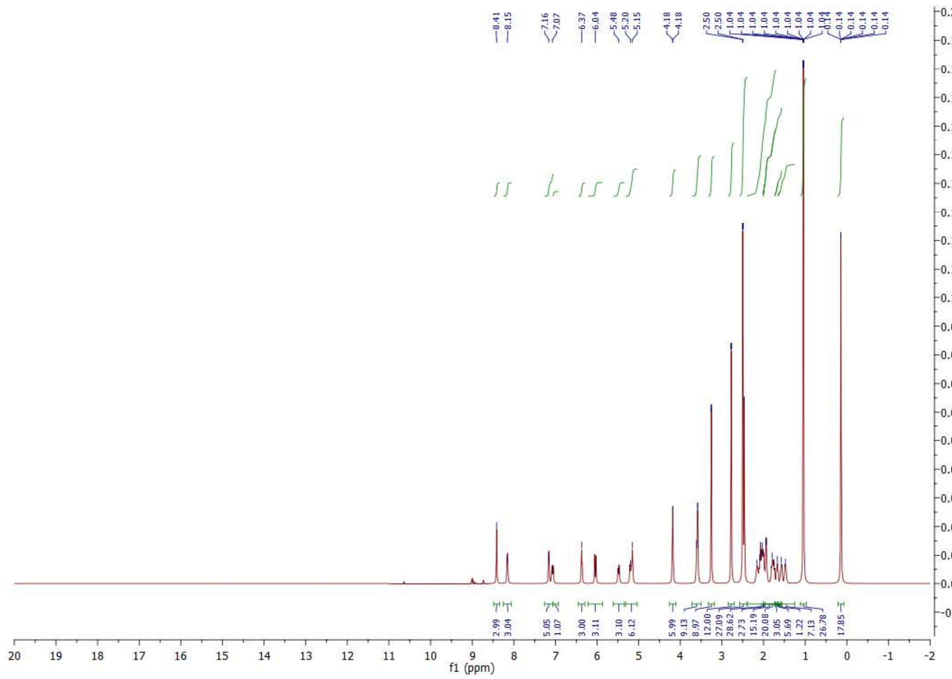

Figure S100. The ^{1}H NMR spectrum of **Pro-3DPQ-5**.

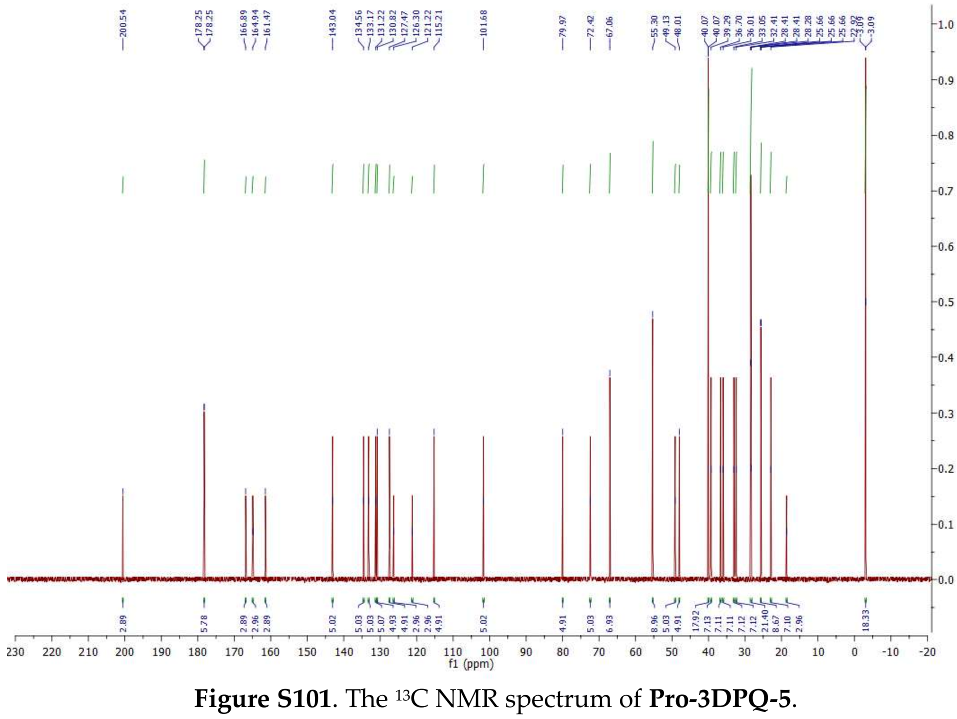

Figure S101. The ^{13}C NMR spectrum of **Pro-3DPQ-5**.

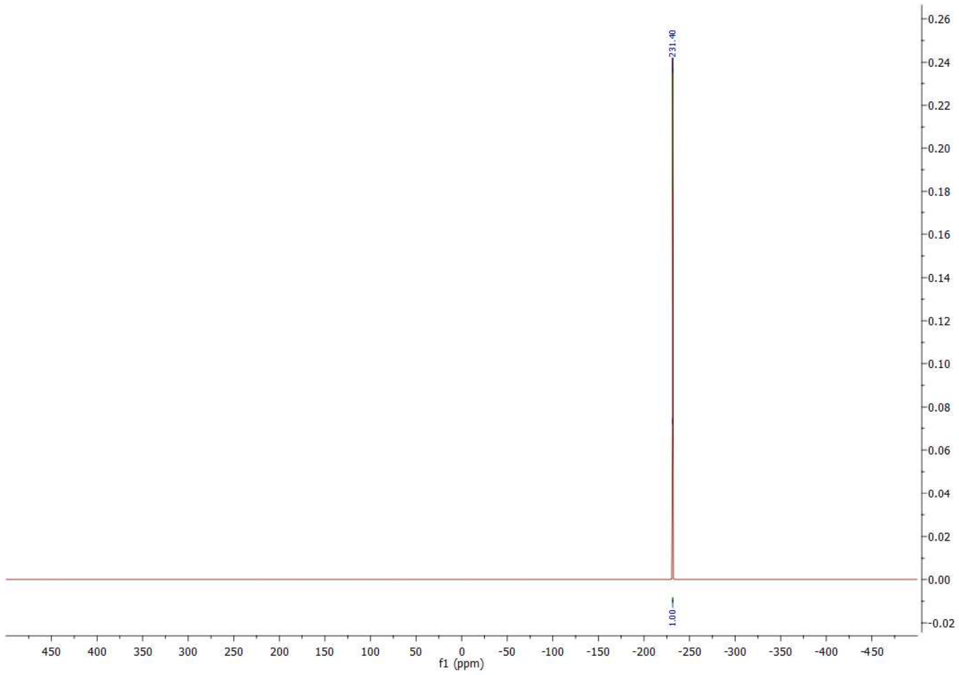

Figure S102. The ^{15}N NMR spectrum of **Pro-3DPQ-5**.

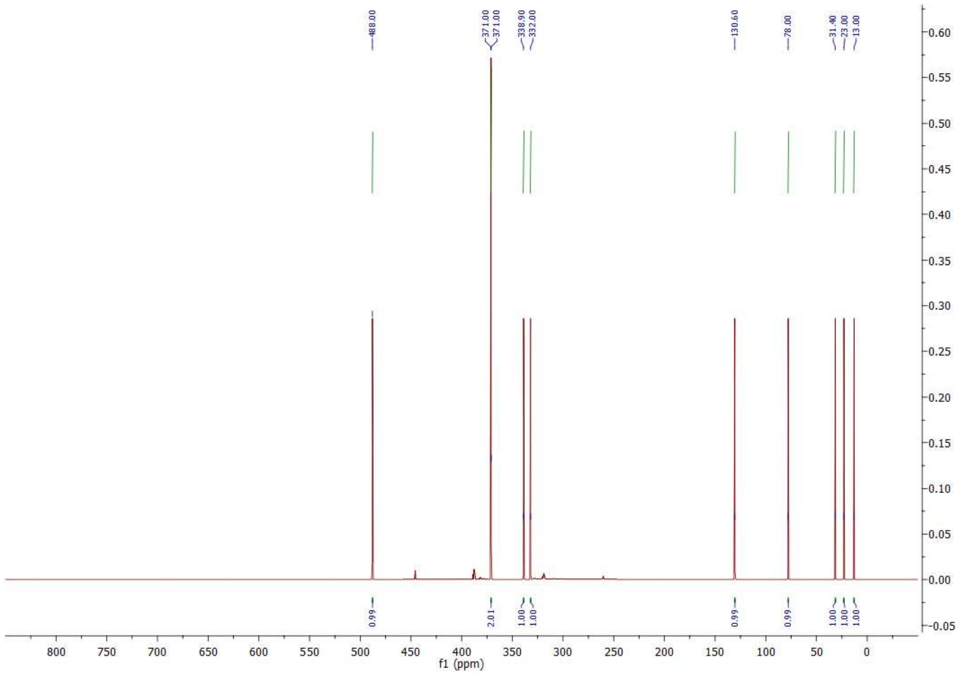

Figure S103. The ^{17}O NMR spectrum of **Pro-3DPQ-5**.

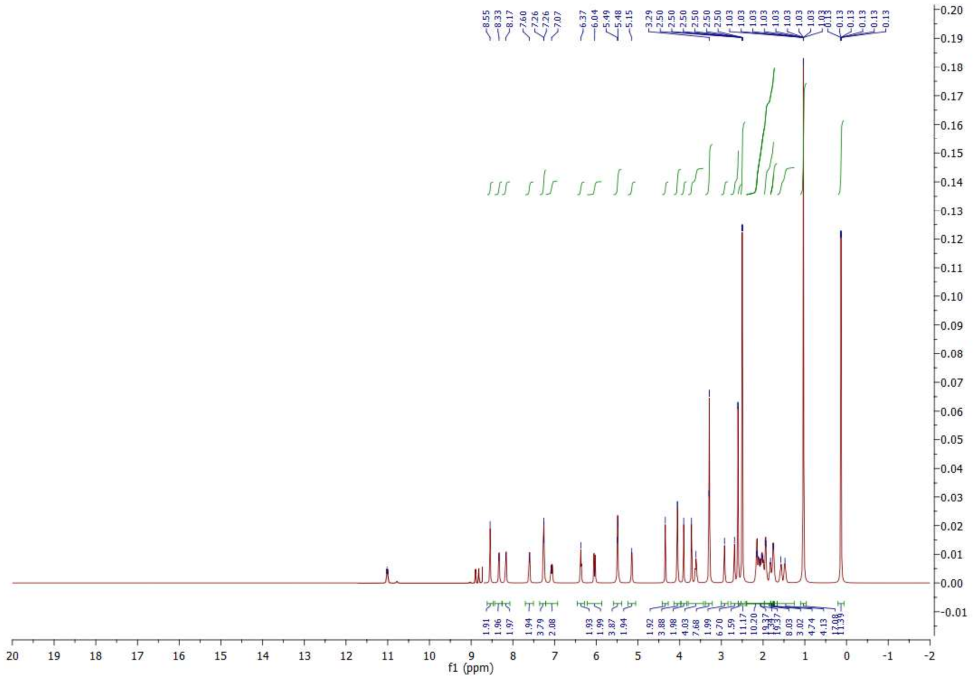

Figure S104. The ^{1}H NMR spectrum of **Pro-3DPQ-6**.

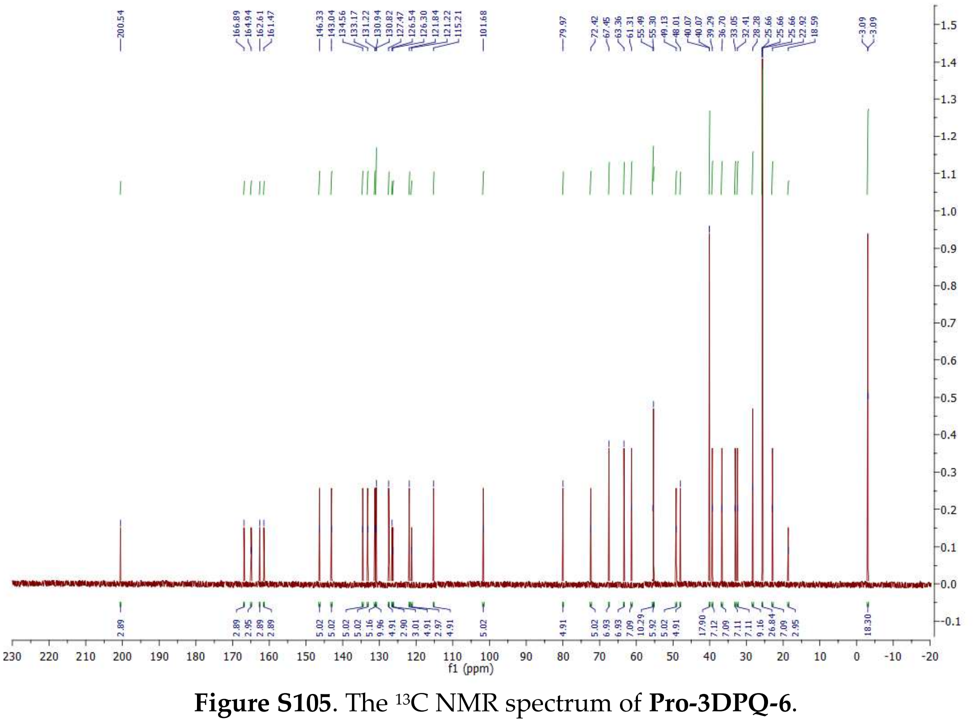

Figure S105. The ^{13}C NMR spectrum of **Pro-3DPQ-6**.

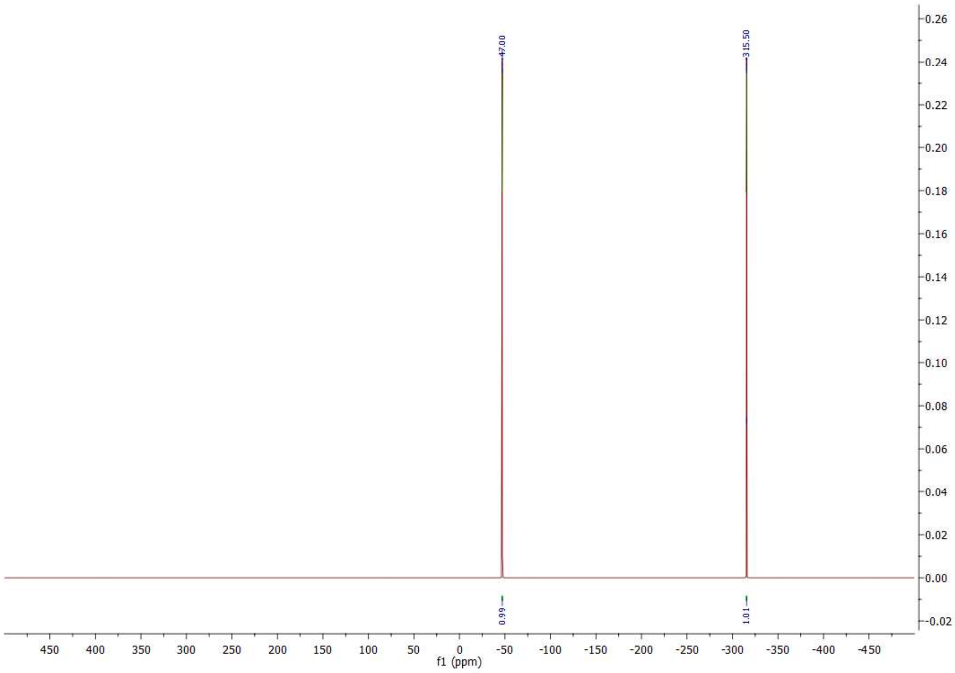

Figure S106. The ^{15}N NMR spectrum of **Pro-3DPQ-6**.

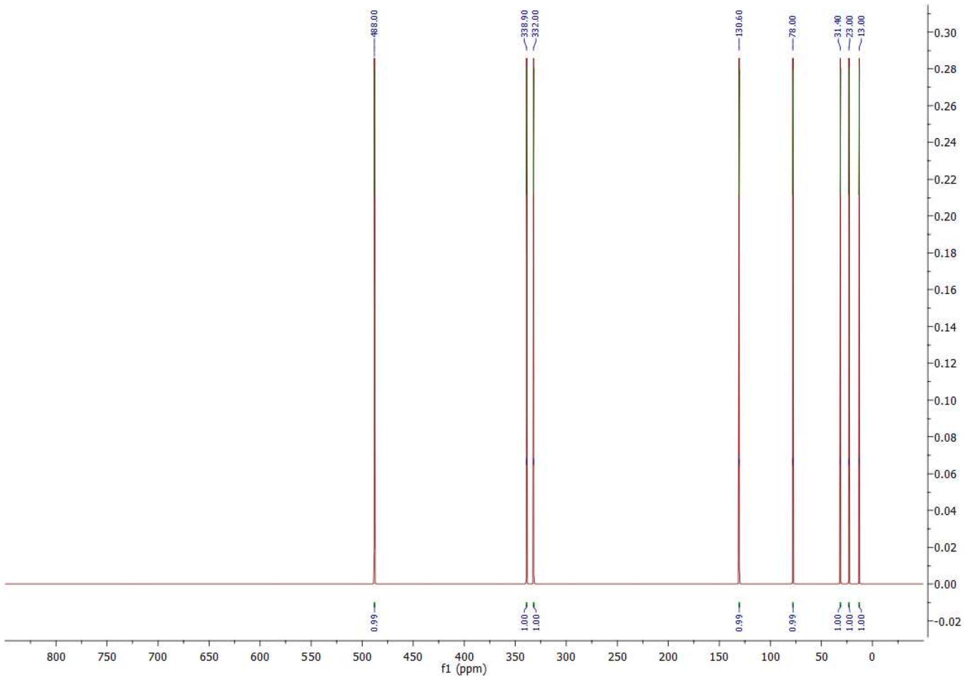

Figure S107. The ^{17}O NMR spectrum of **Pro-3DPQ-6**.

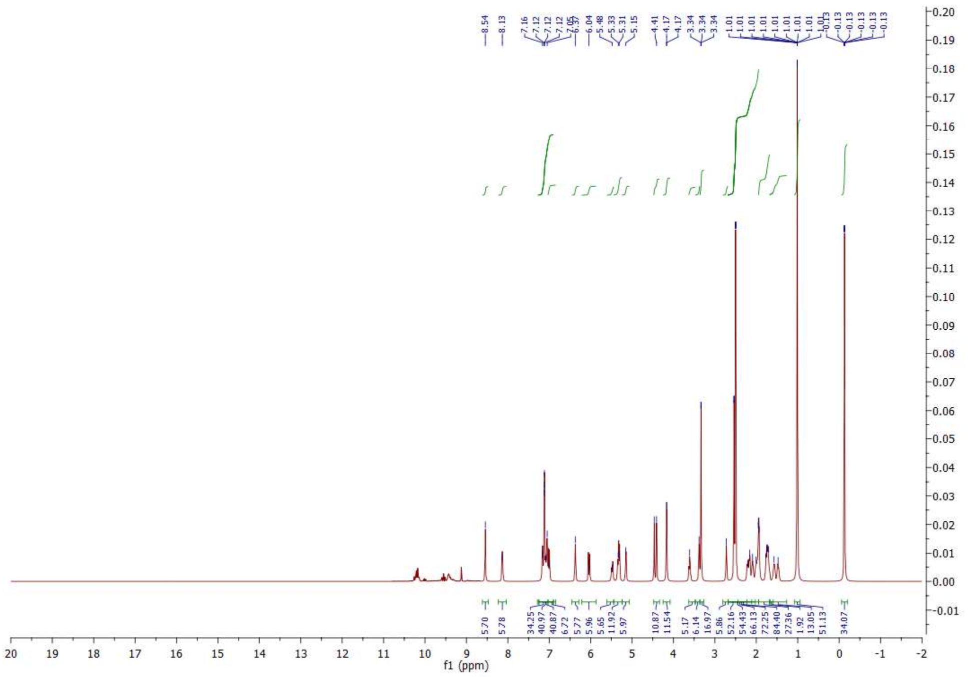

Figure S108. The ^{1}H NMR spectrum of **Pro-3DPQ-7**.

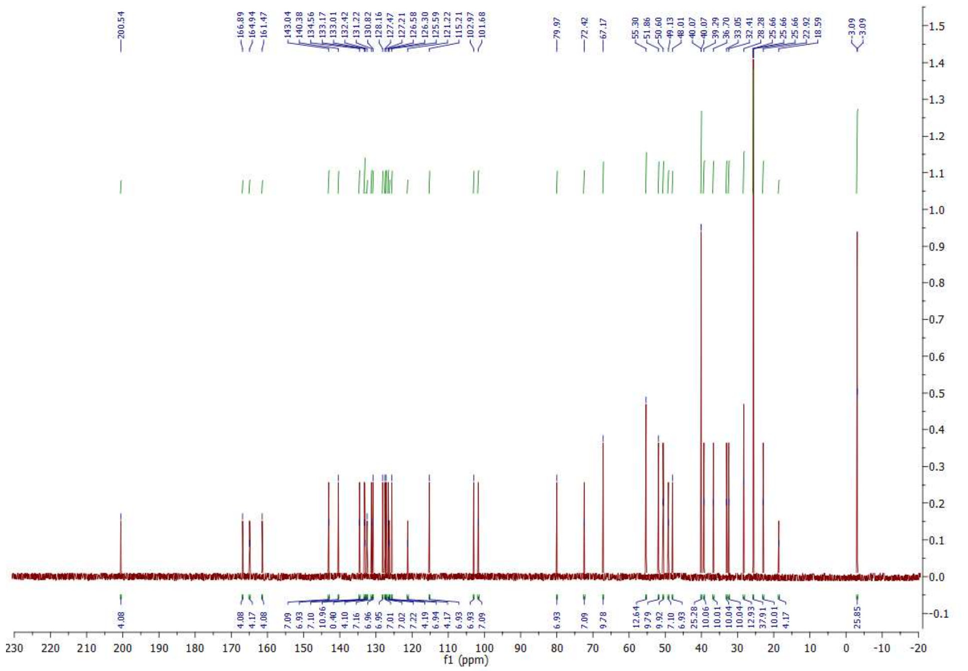

Figure S109. The ^{13}C NMR spectrum of **Pro-3DPQ-7**.

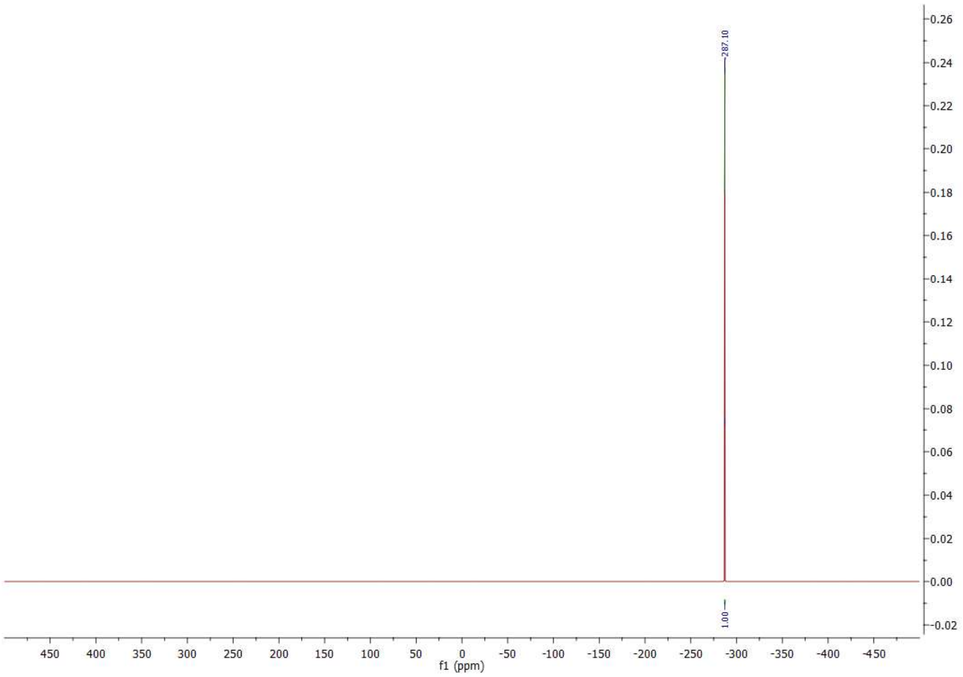

Figure S110. The ^{15}N NMR spectrum of **Pro-3DPQ-7**.

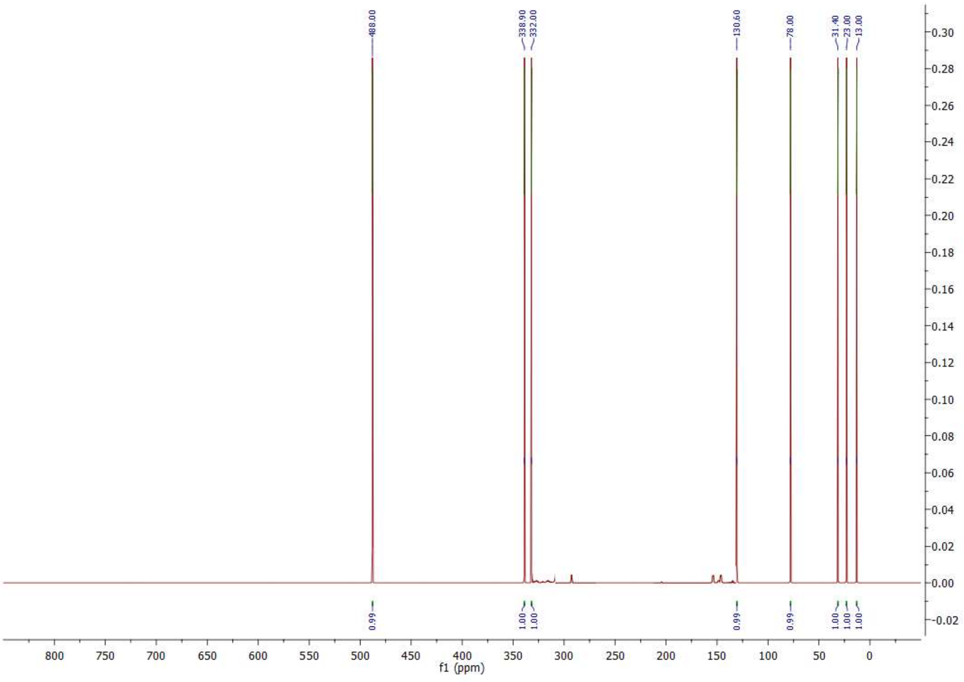

Figure S111. The ^{17}O NMR spectrum of **Pro-3DPQ-7**.

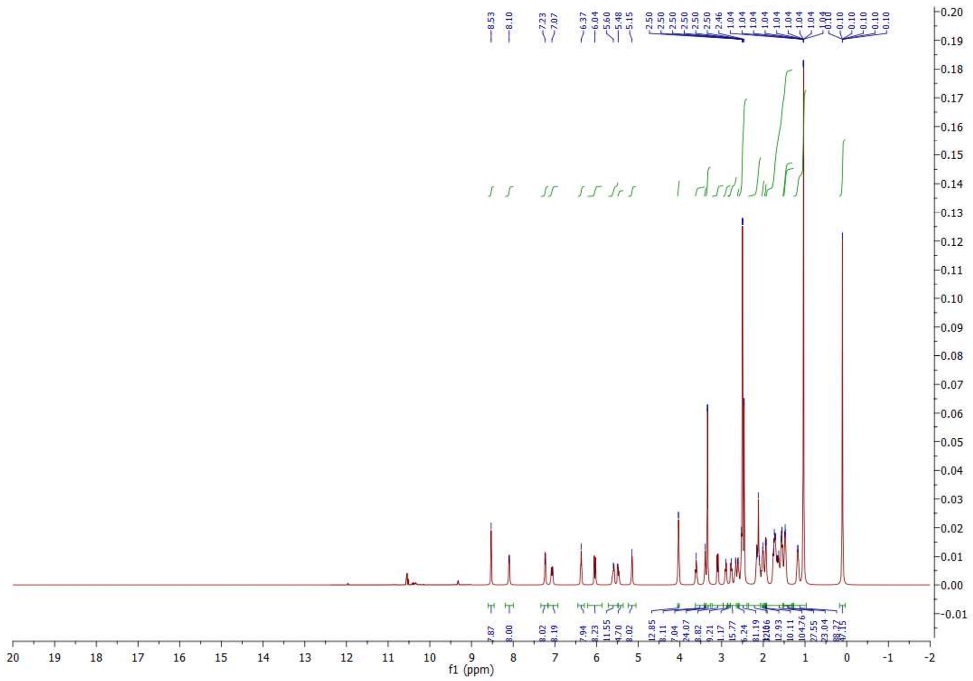

Figure S112. The ¹H NMR spectrum of **Pro-3DPQ-8**.

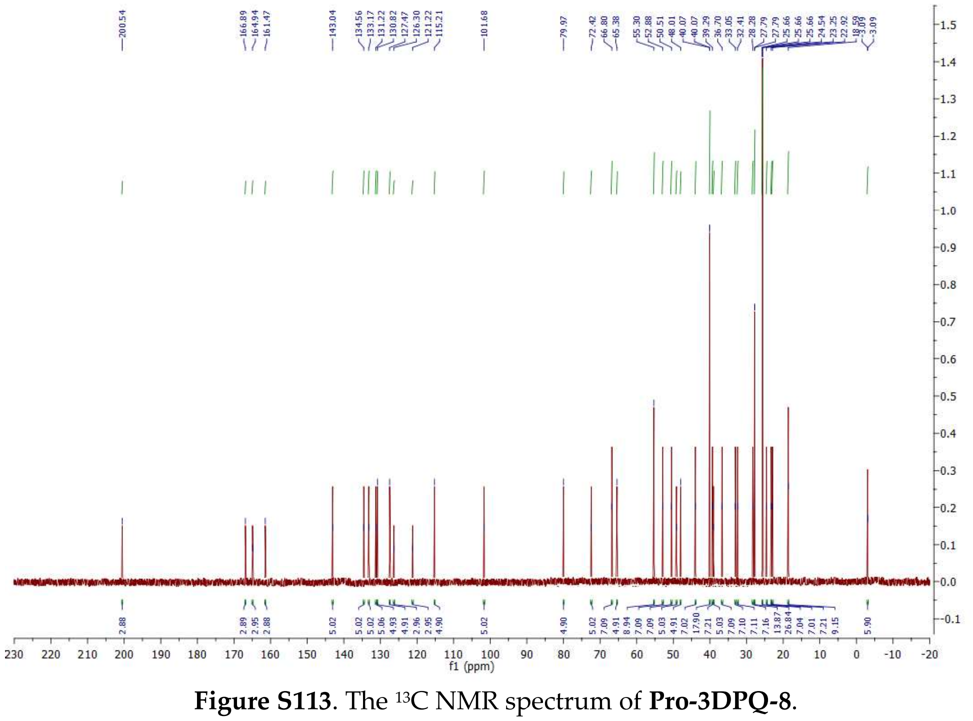

Figure S113. The ¹³C NMR spectrum of **Pro-3DPQ-8**.

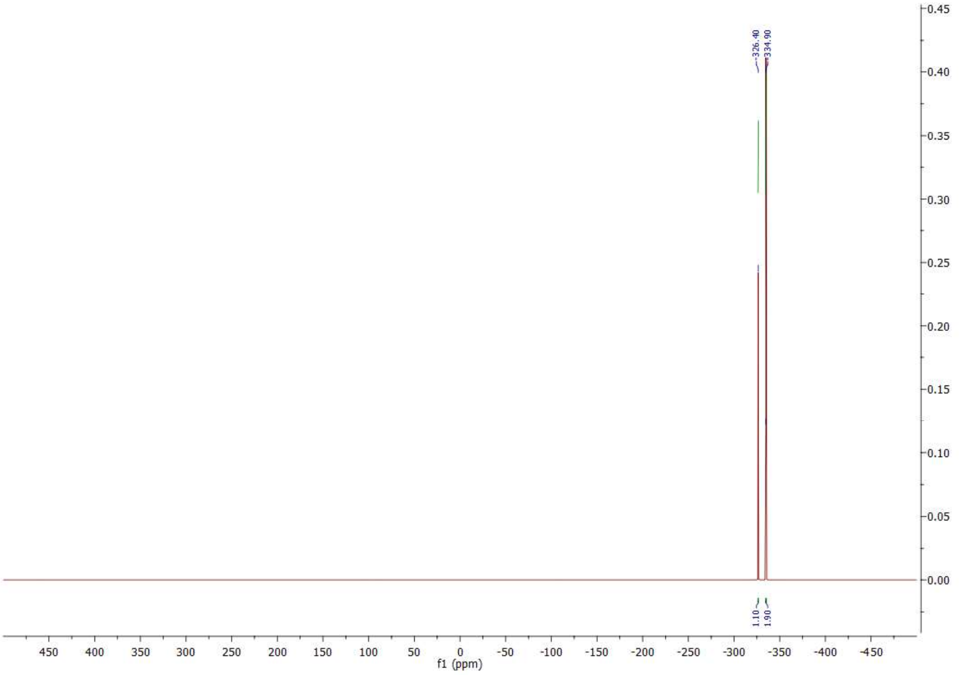

Figure S114. The ^{15}N NMR spectrum of **Pro-3DPQ-8**.

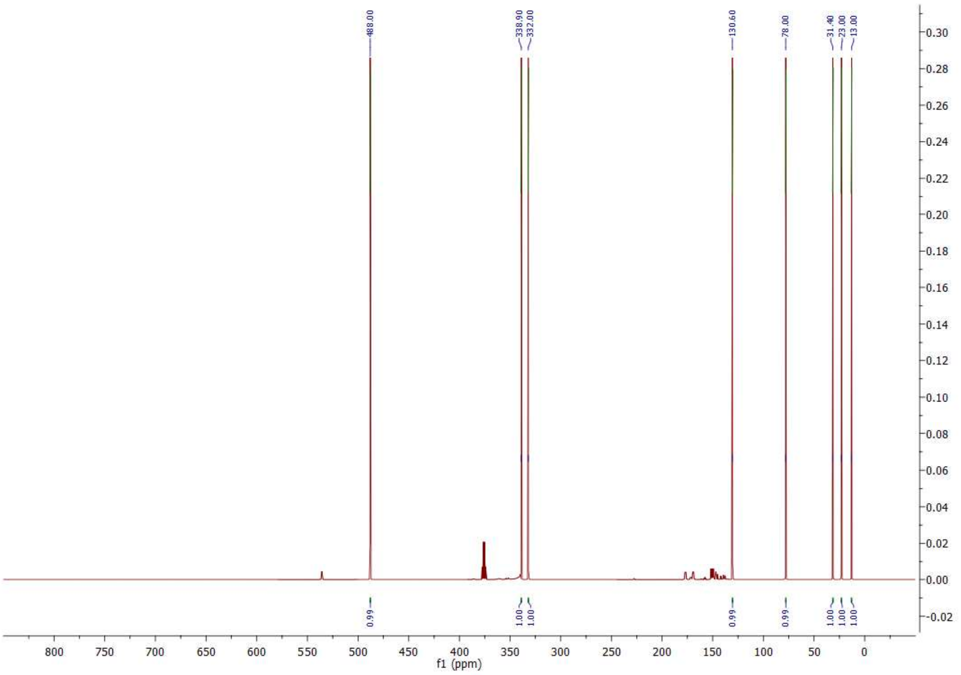

Figure S115. The ^{17}O NMR spectrum of **Pro-3DPQ-8**.

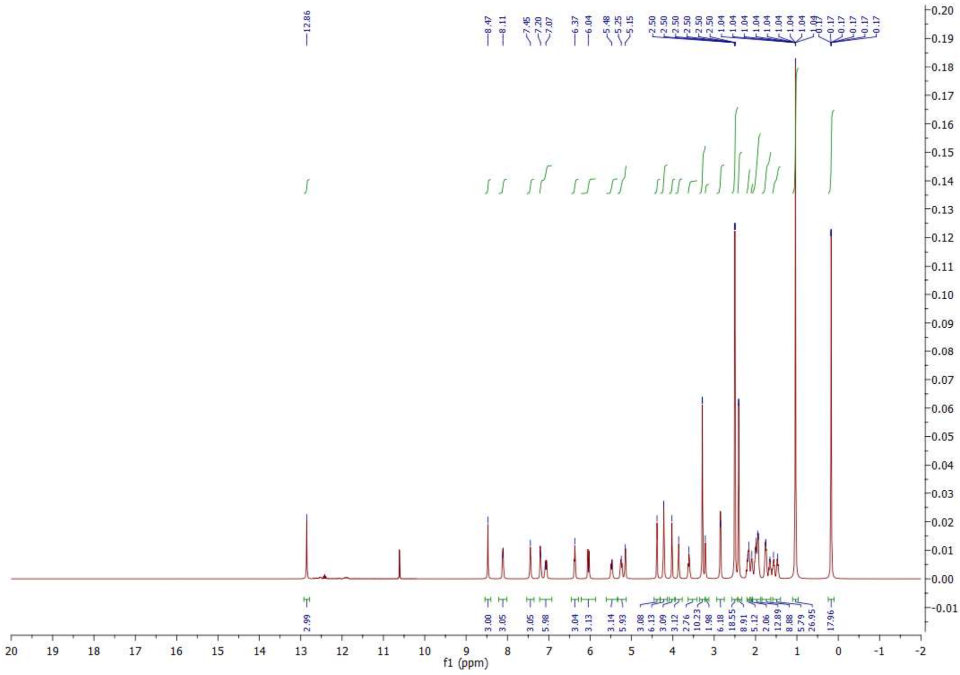

Figure S116. The ¹H NMR spectrum of **Pro-3DPQ-9**.

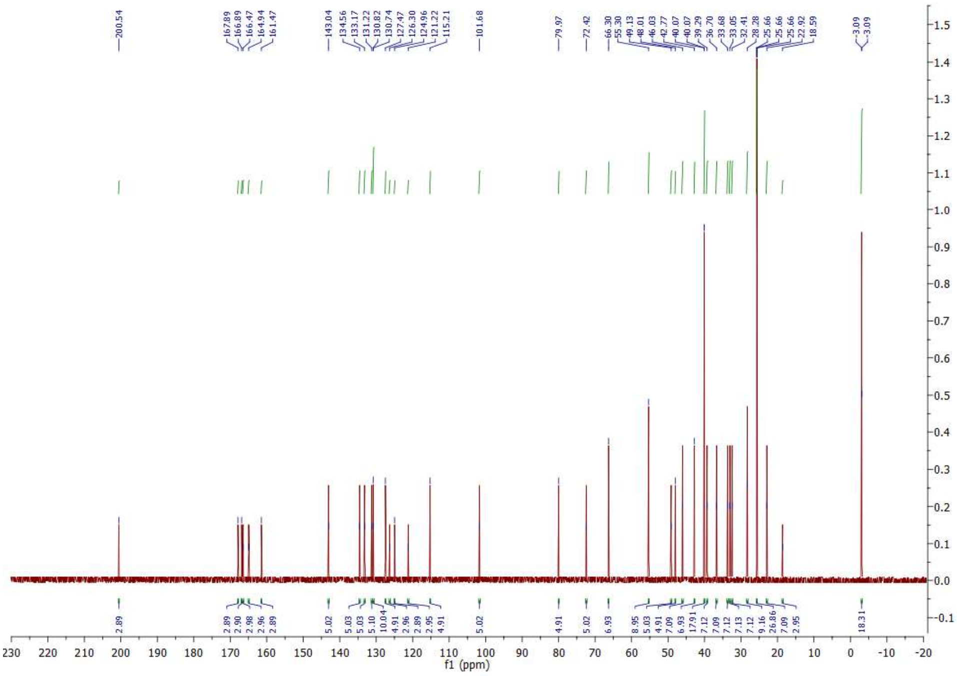

Figure S117. The ¹³C NMR spectrum of **Pro-3DPQ-9**.

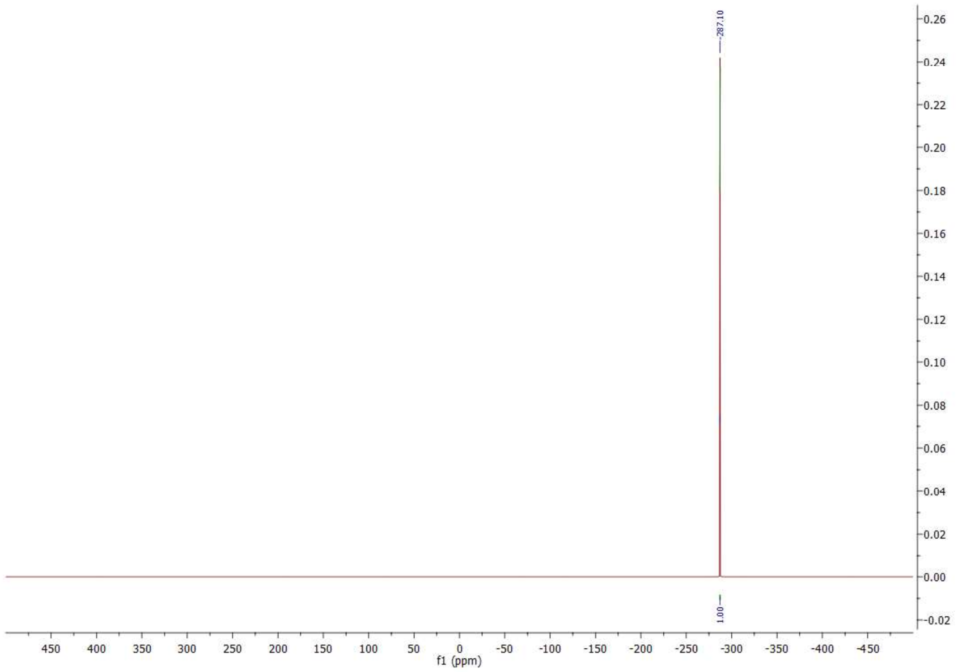

Figure S118. The ^{15}N NMR spectrum of **Pro-3DPQ-9**.

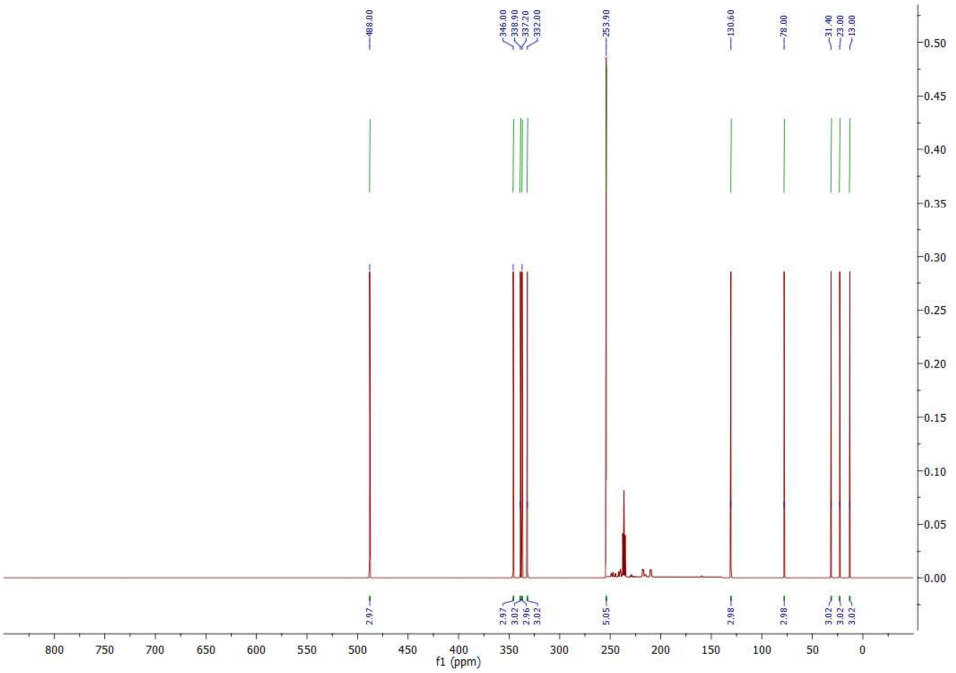

Figure S119. The ^{17}O NMR spectrum of **Pro-3DPQ-9**.

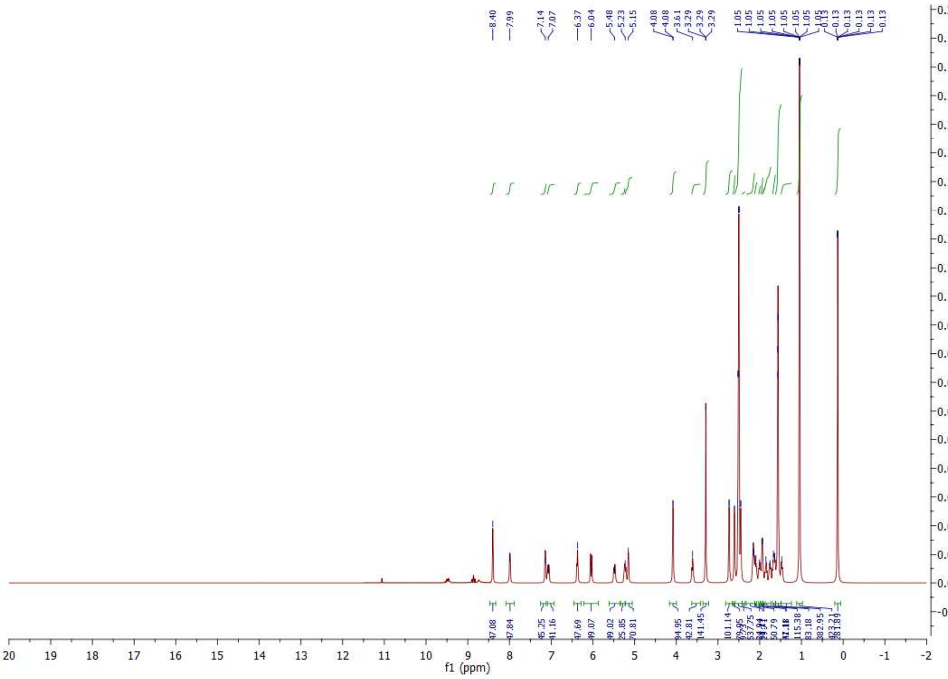

Figure S120. The ^{1}H NMR spectrum of **Pro-3DPQ-10**.

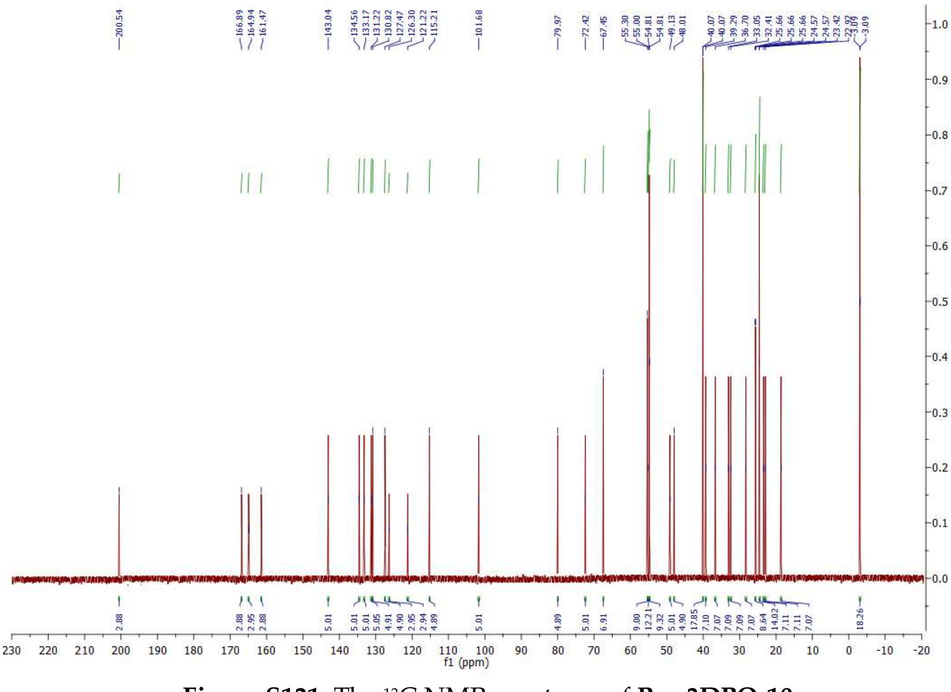

Figure S121. The ^{13}C NMR spectrum of **Pro-3DPQ-10**.

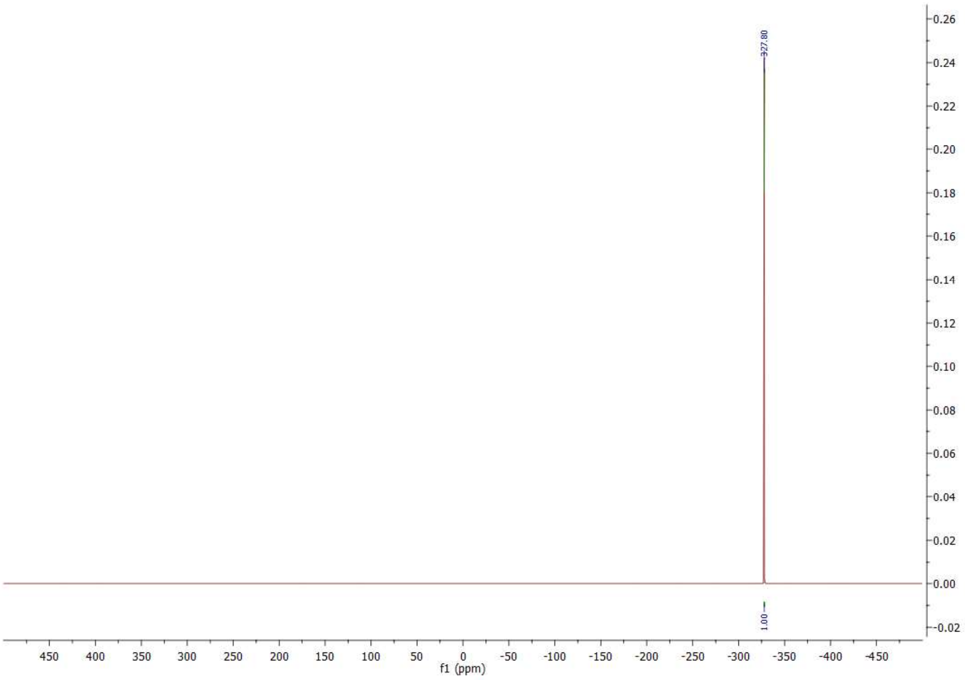

Figure S122. The ^{15}N NMR spectrum of **Pro-3DPQ-10**.

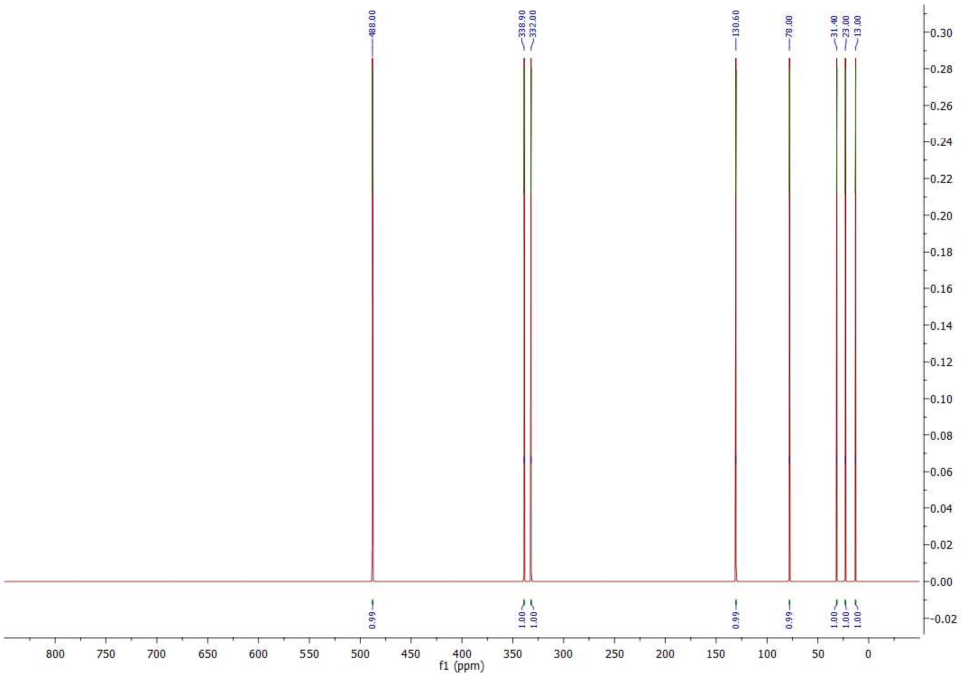

Figure S123. The ^{17}O NMR spectrum of **Pro-3DPQ-10**.

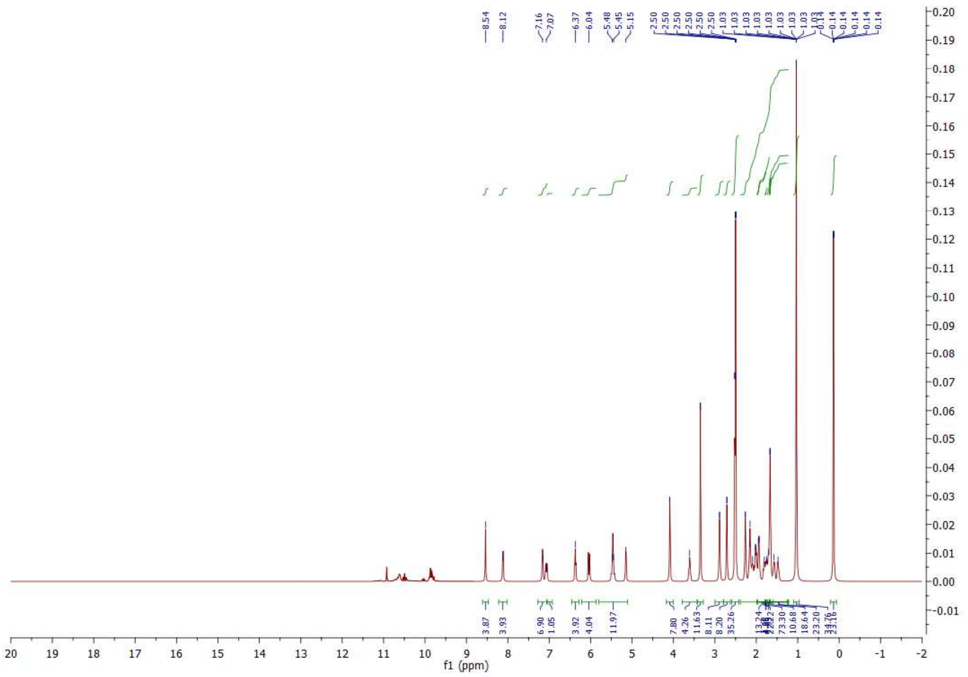

Figure S124. The ¹H NMR spectrum of **Pro-3DPQ-11**.

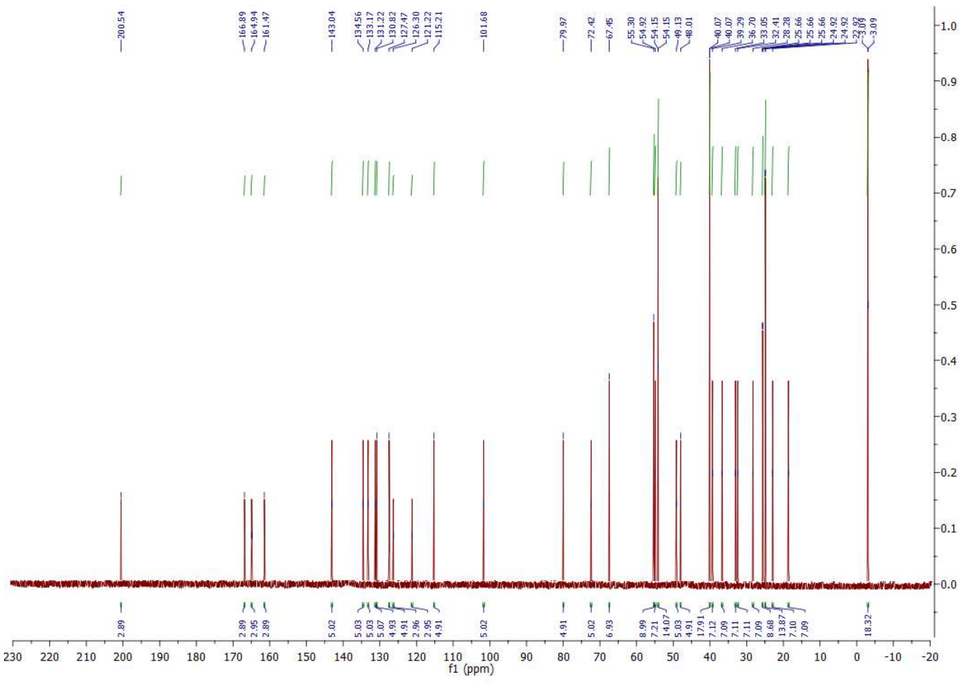

Figure S125. The ¹³C NMR spectrum of **Pro-3DPQ-11**.

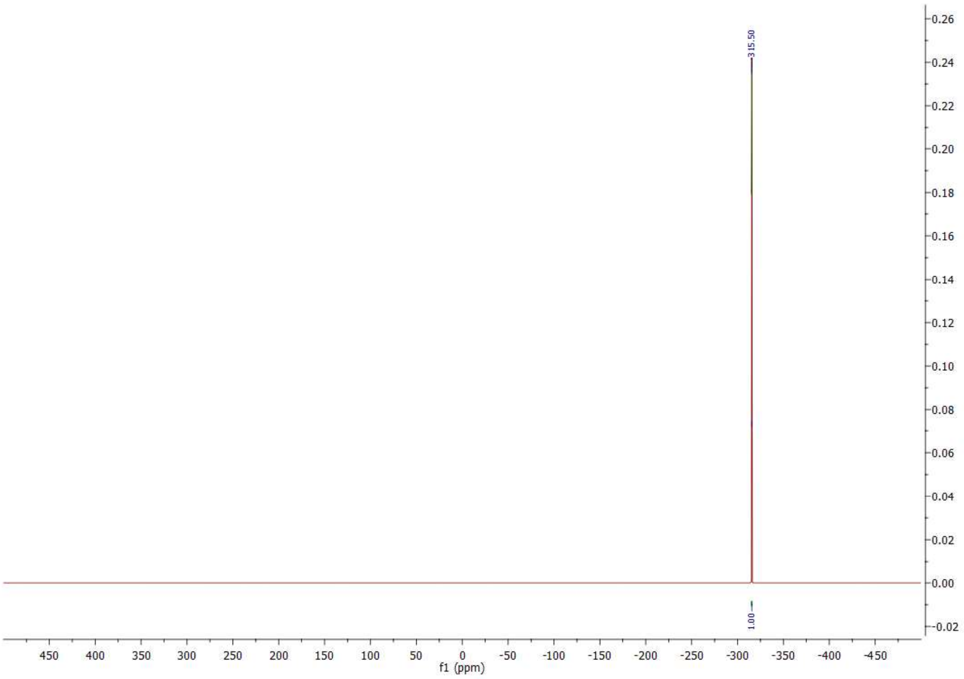

Figure S126. The ^{15}N NMR spectrum of **Pro-3DPQ-11**.

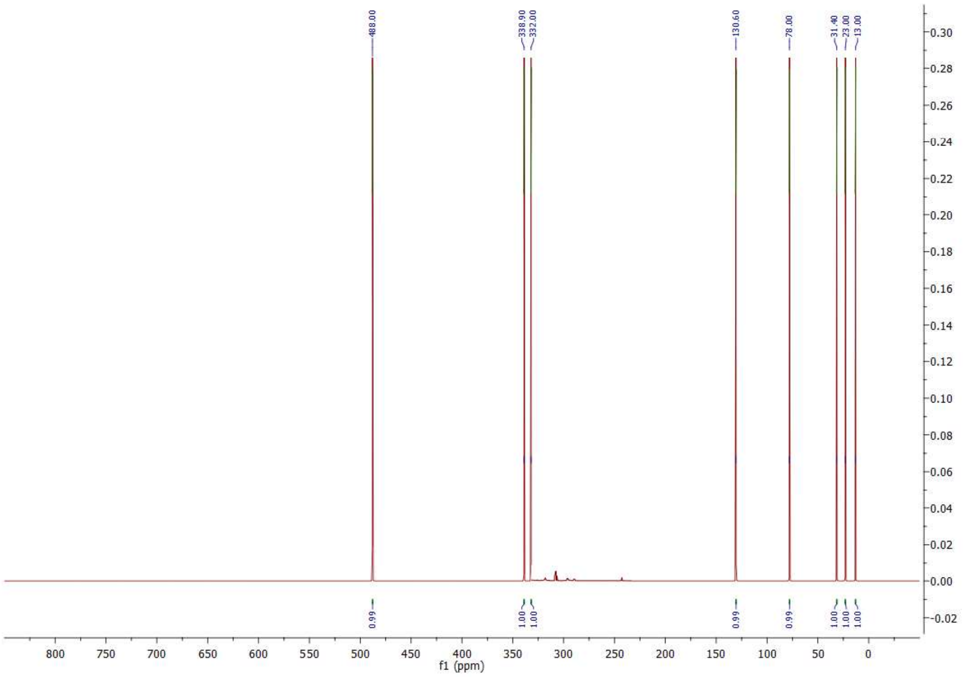

Figure S127. The ^{17}O NMR spectrum of **Pro-3DPQ-11**.

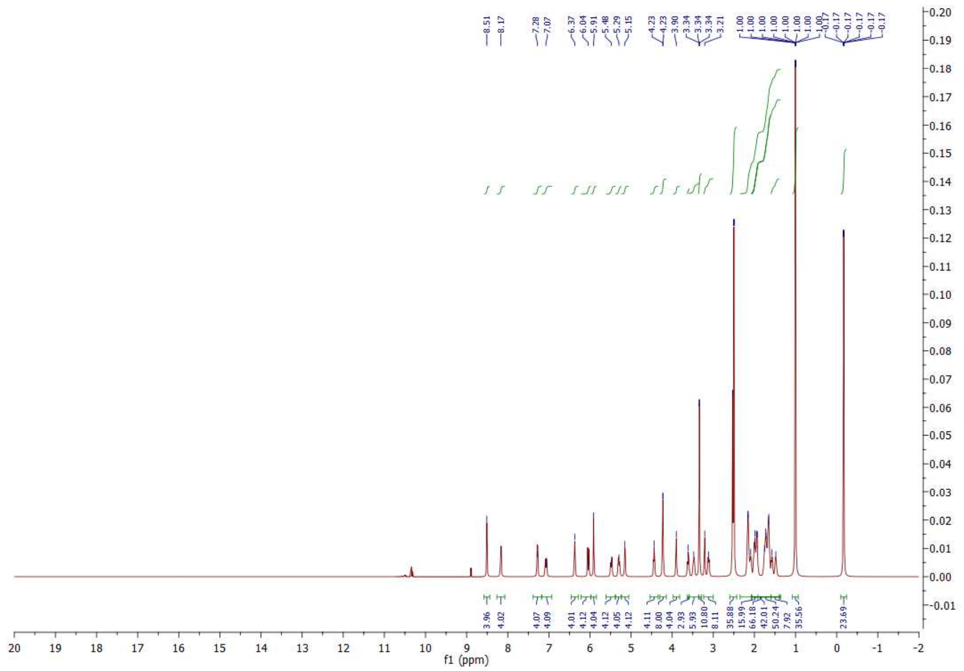

Figure S128. The ^{1}H NMR spectrum of **Pro-3DPQ-12**.

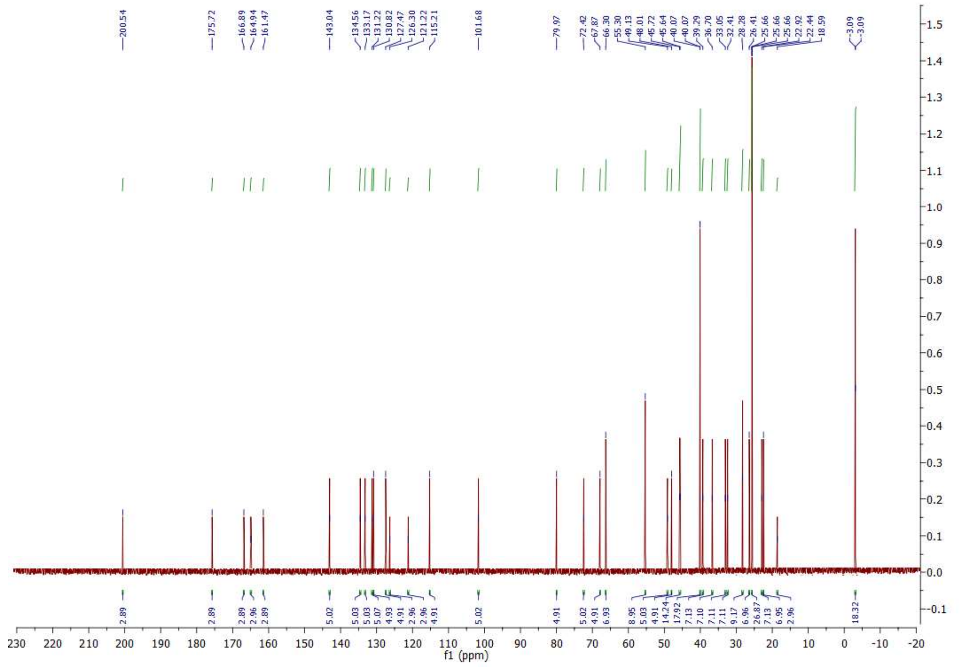

Figure S129. The ^{13}C NMR spectrum of **Pro-3DPQ-12**.

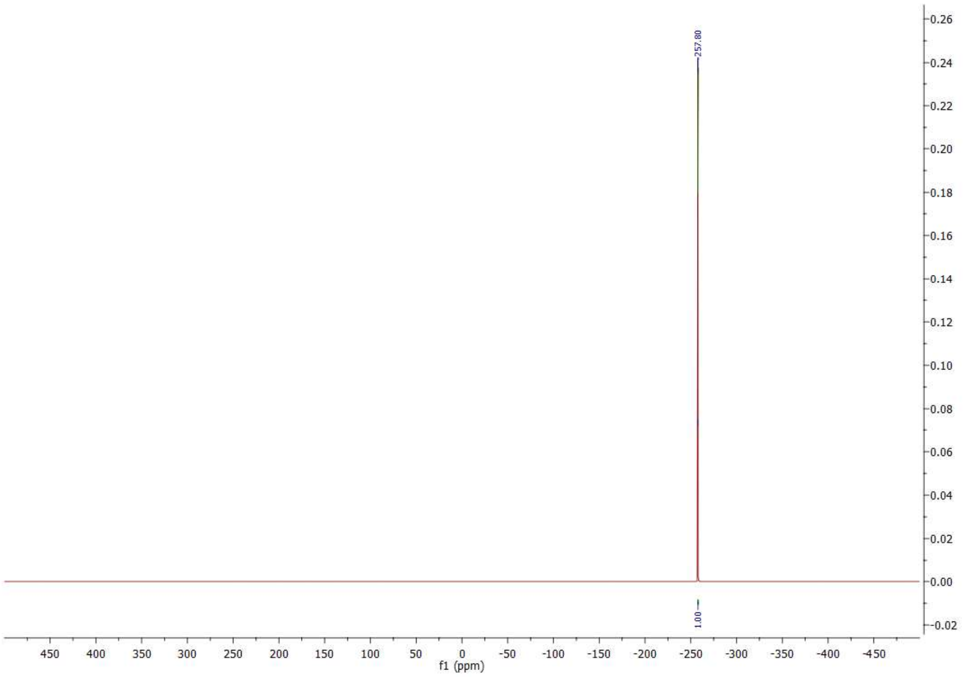

Figure S130. The ^{15}N NMR spectrum of **Pro-3DPQ-12**.

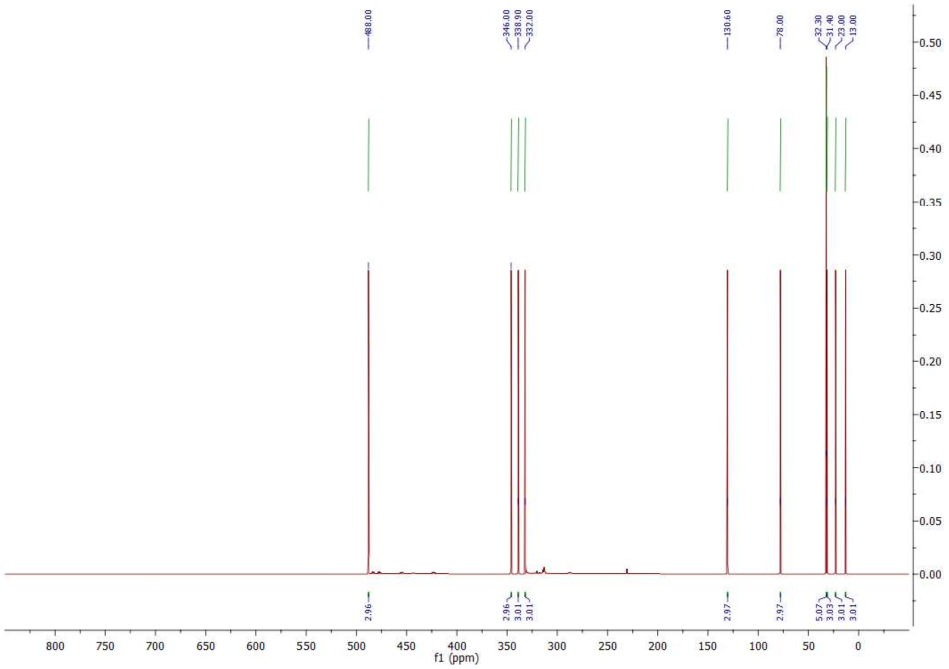

Figure S131. The ^{17}O NMR spectrum of **Pro-3DPQ-12**.

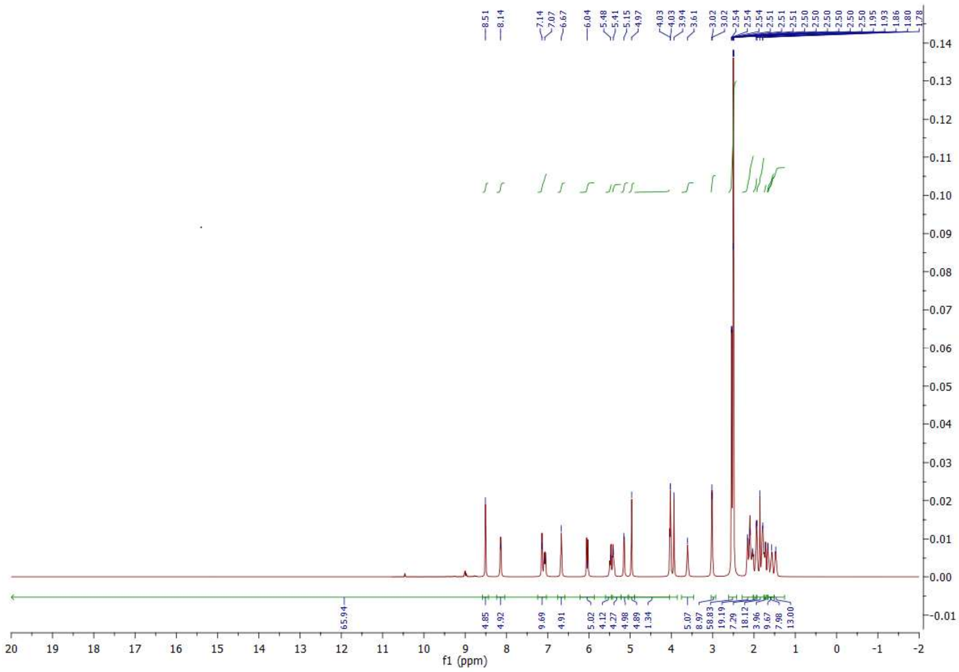

Figure S132. The ^{1}H NMR spectrum of **3DPQ-1**.

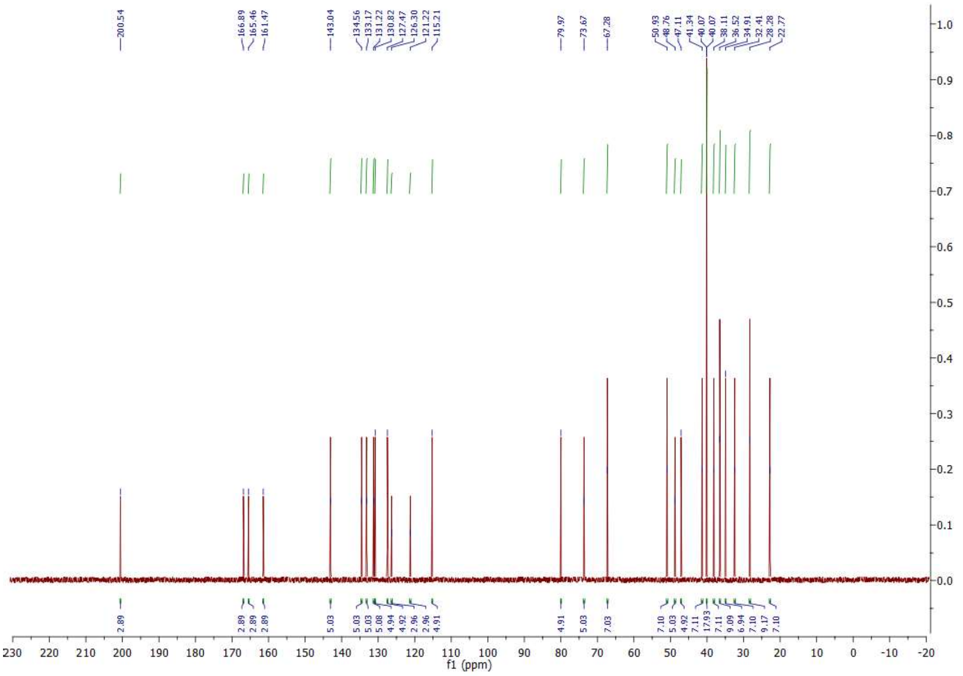

Figure S133. The ^{13}C NMR spectrum of **3DPQ-1**.

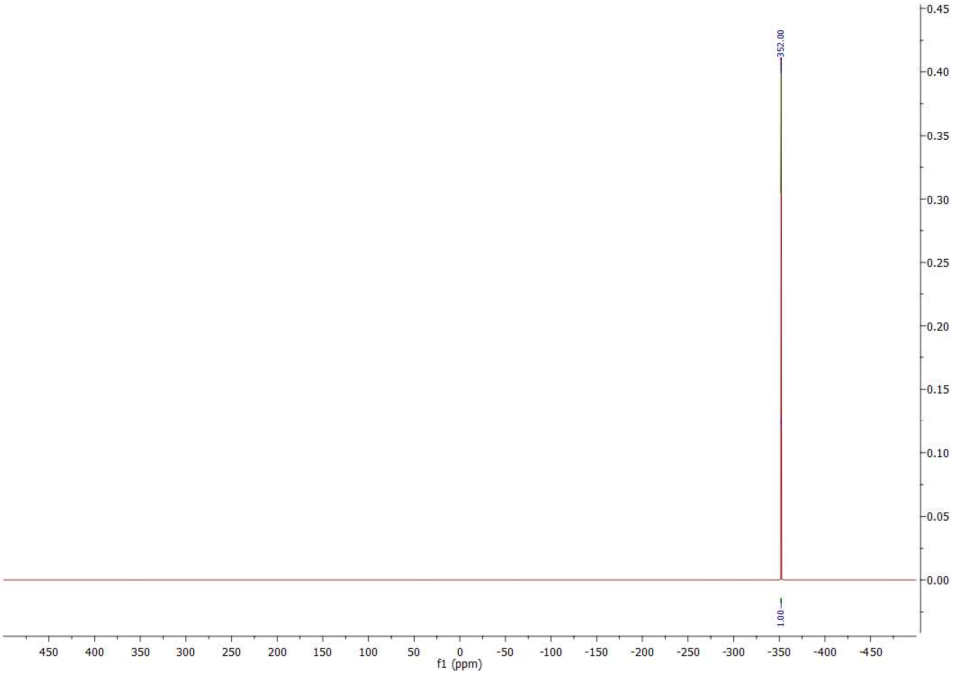

Figure S134. The ^{15}N NMR spectrum of **3DPQ-1**.

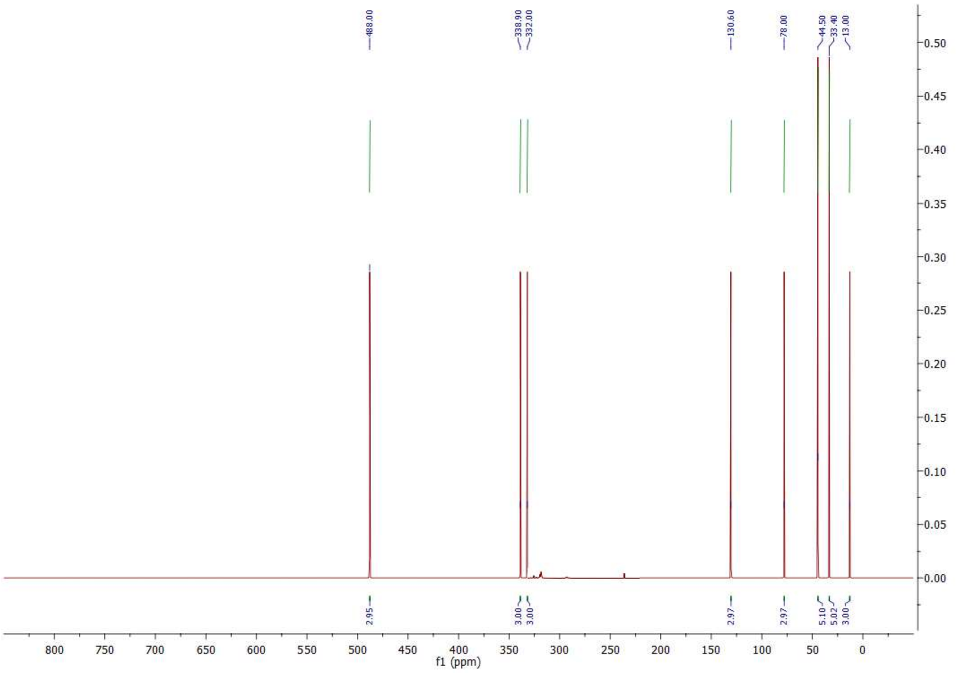

Figure S135. The ^{17}O NMR spectrum of **3DPQ-1**.

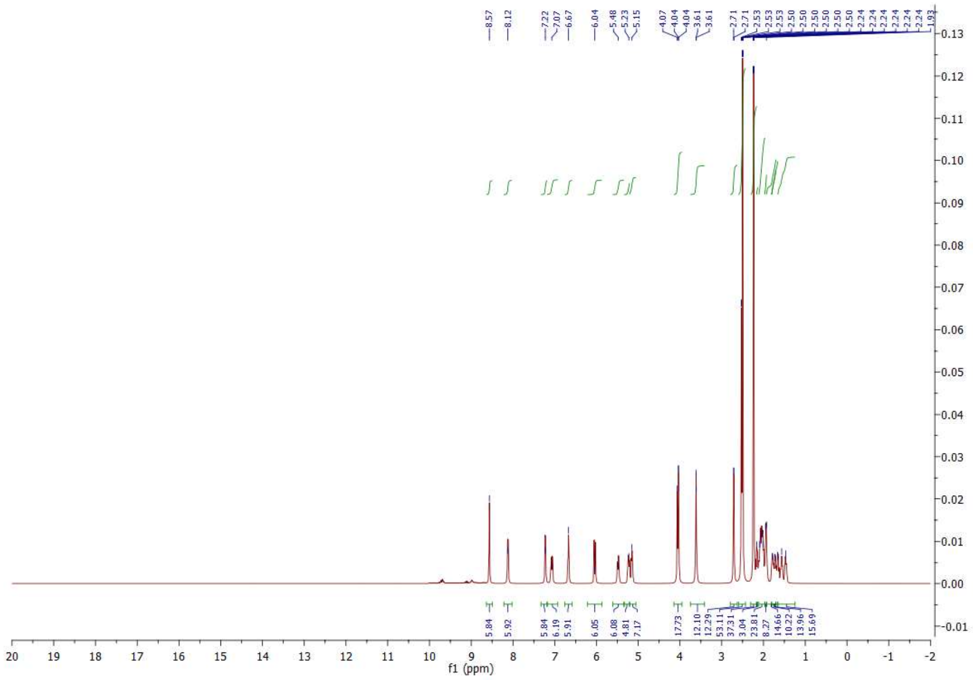

Figure S136. The ^{1}H NMR spectrum of **3DPQ-2**.

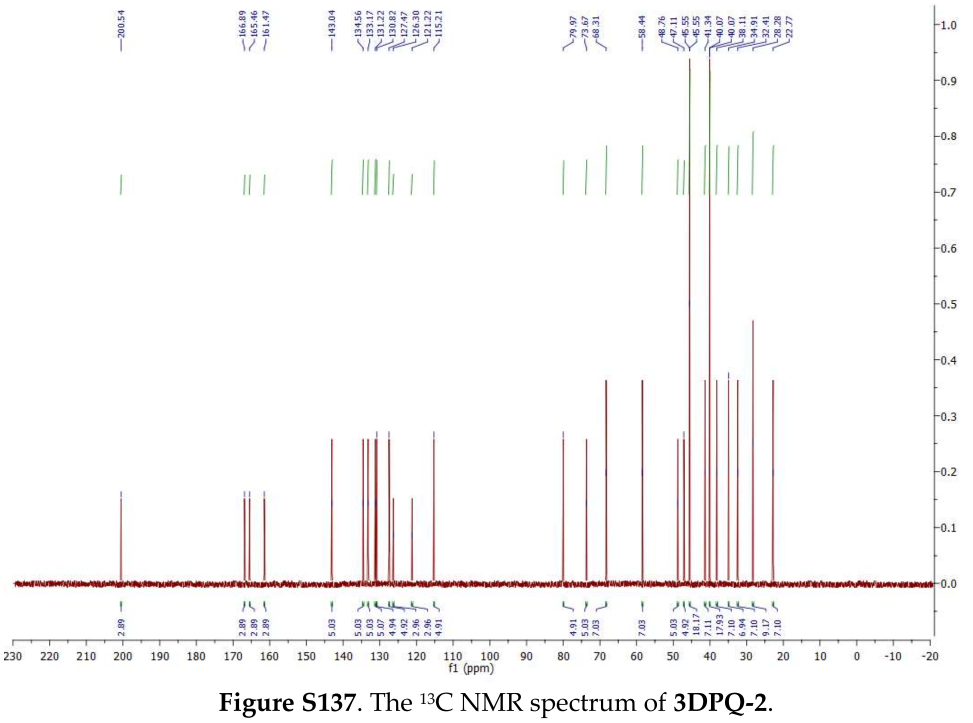

Figure S137. The ^{13}C NMR spectrum of **3DPQ-2**.

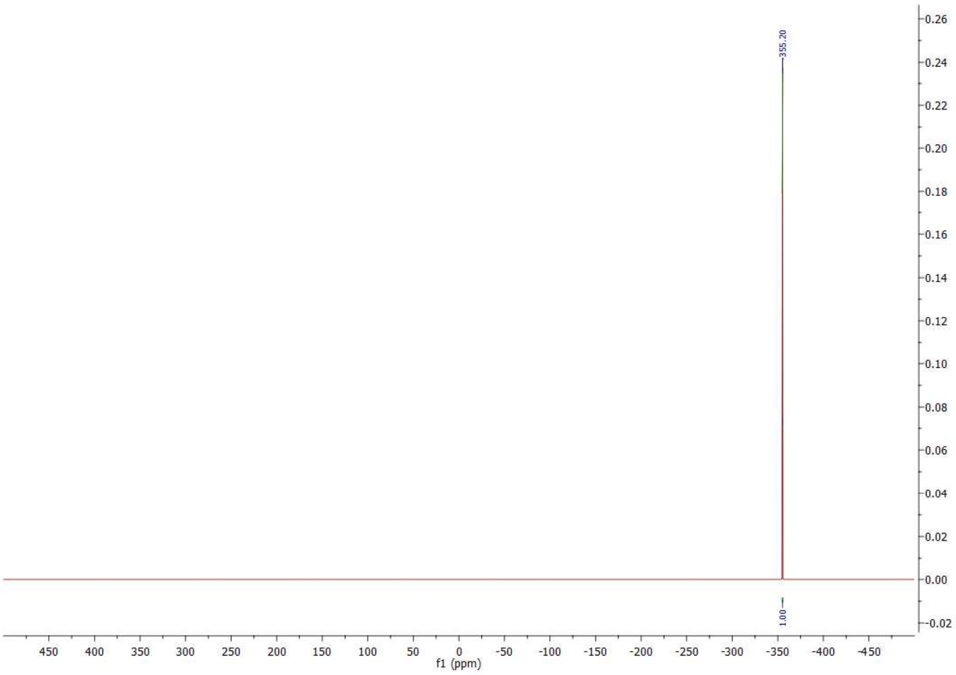

Figure S138. The ^{15}N NMR spectrum of **3DPQ-2**.

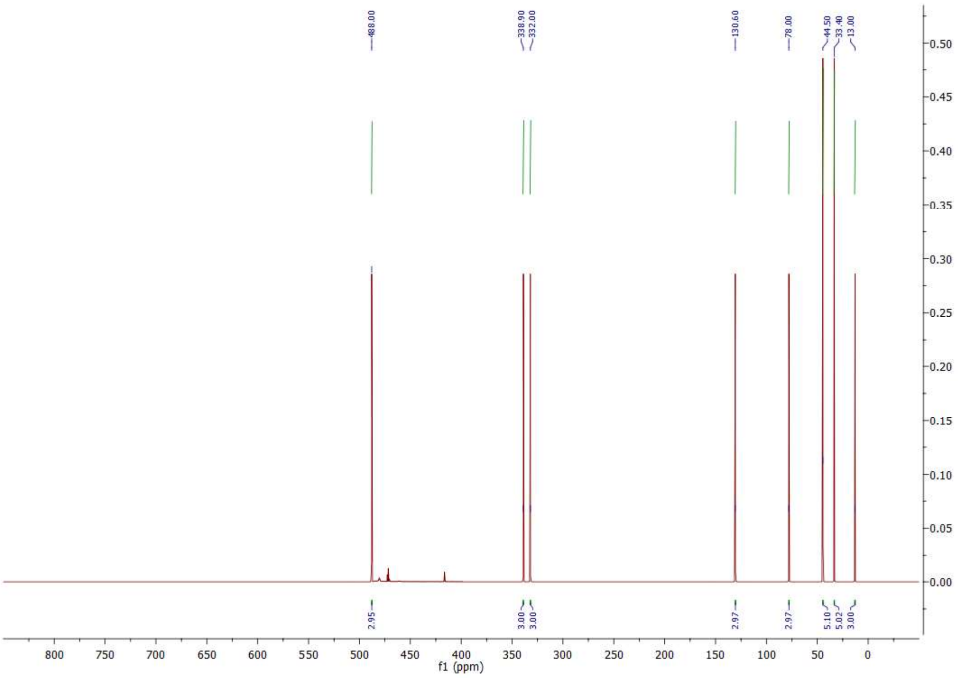

Figure S139. The ^{17}O NMR spectrum of **3DPQ-2**.

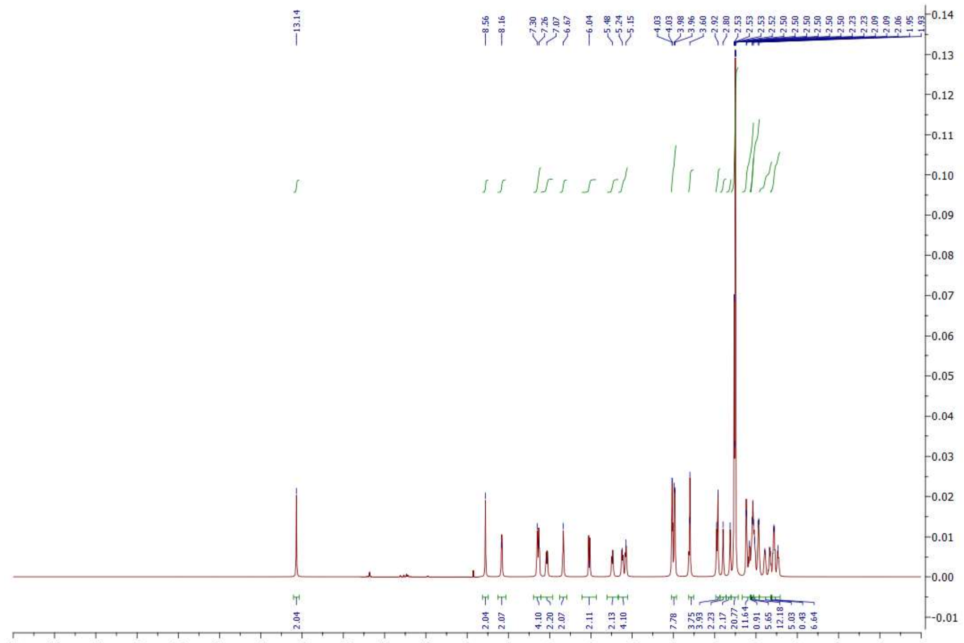

Figure S140. The ^{1}H NMR spectrum of **3DPQ-3**.

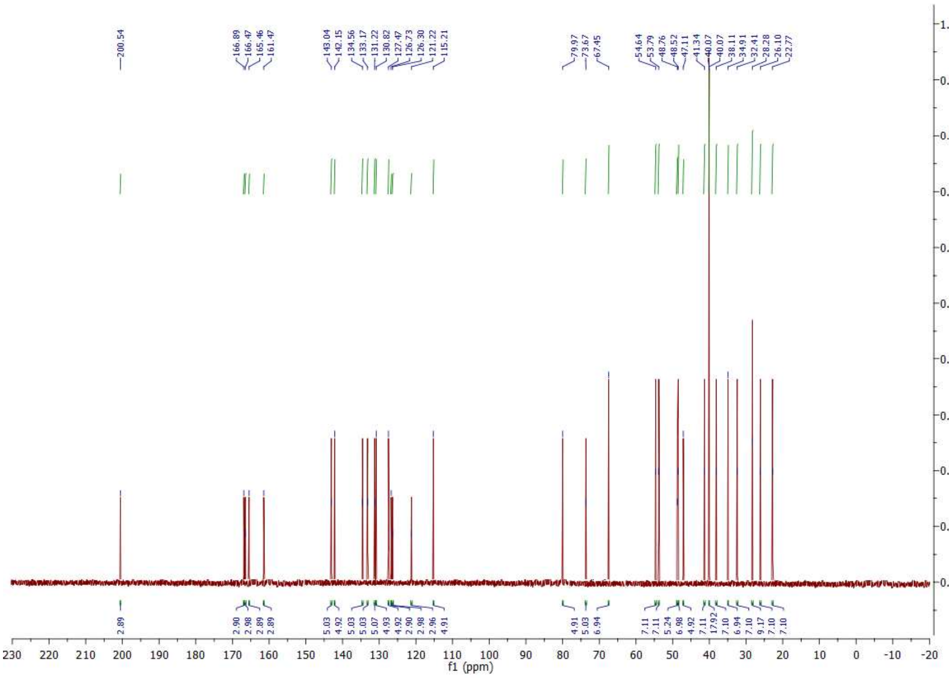

Figure S141. The ^{13}C NMR spectrum of **3DPQ-3**.

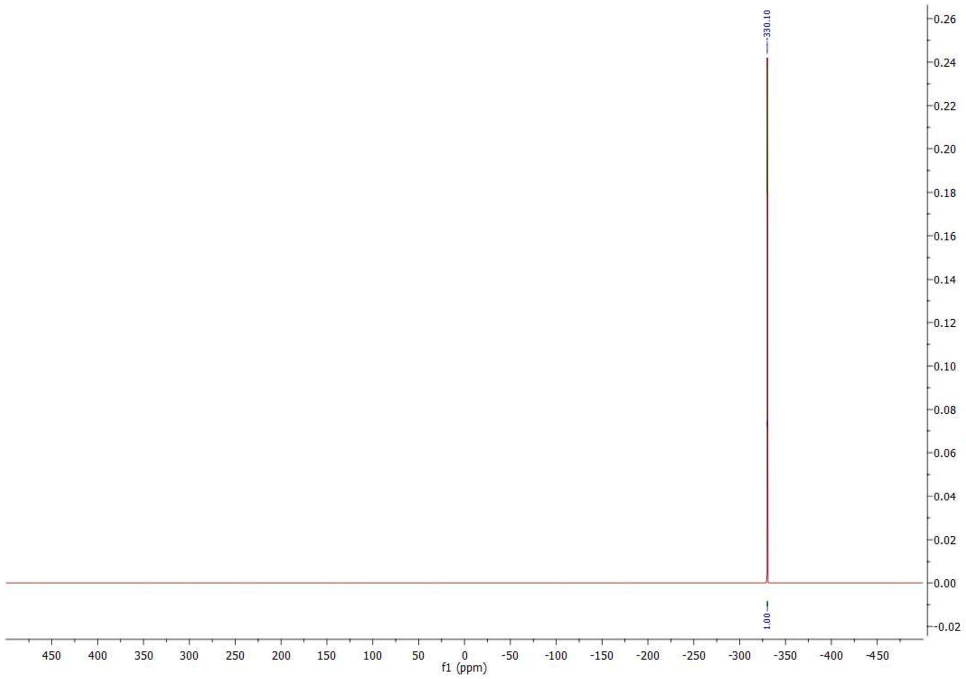

Figure S142. The ^{15}N NMR spectrum of **3DPQ-3**.

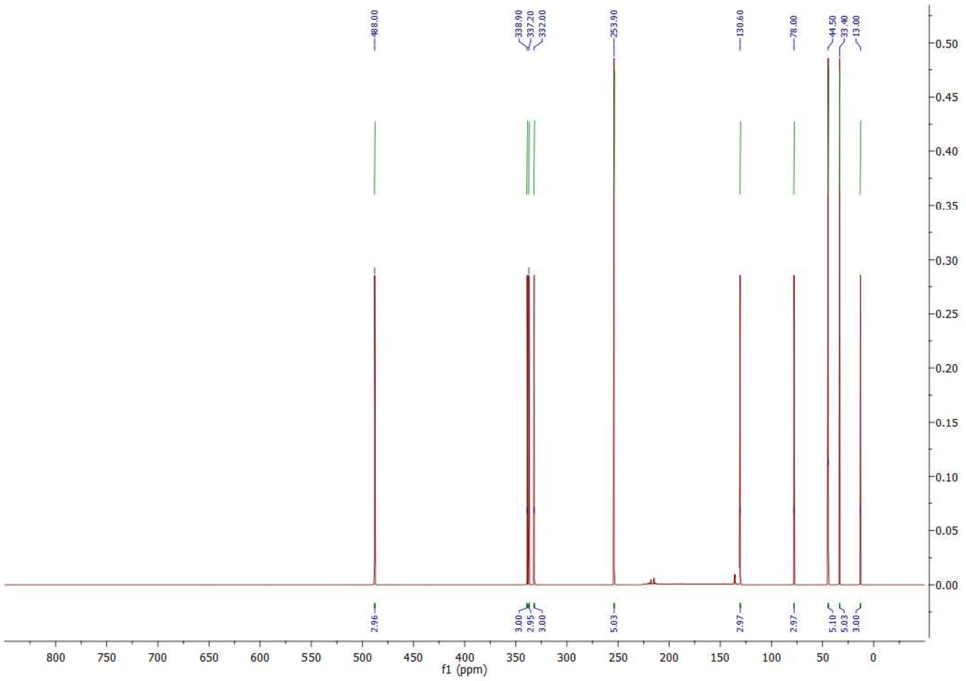

Figure S143. The ^{17}O NMR spectrum of **3DPQ-3**.

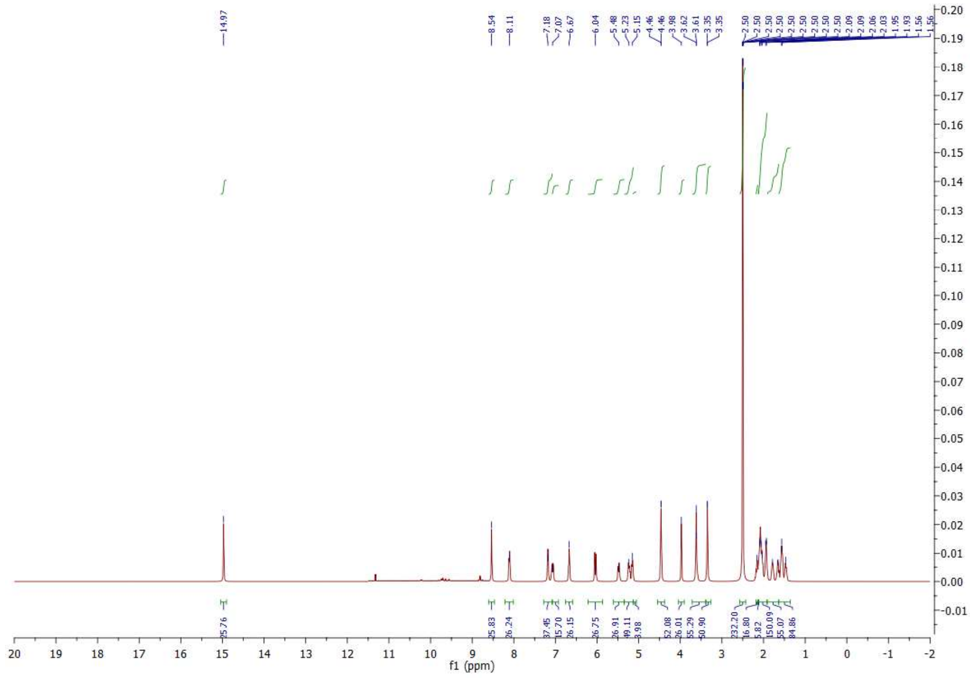

Figure S144. The ^{1}H NMR spectrum of **3DPQ-4**.

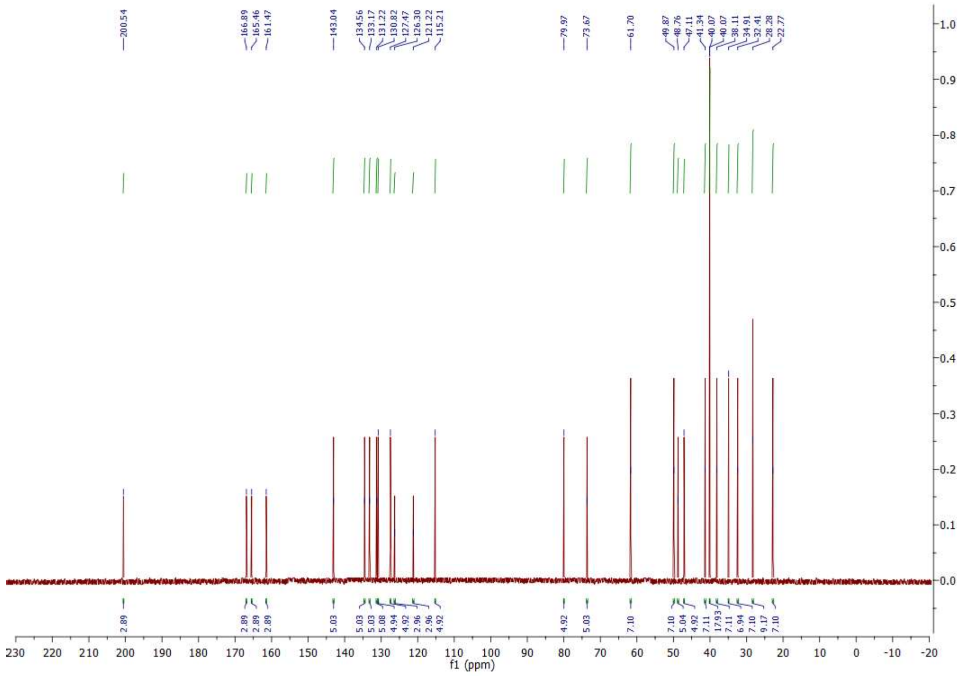

Figure S145. The ^{13}C NMR spectrum of **3DPQ-4**.

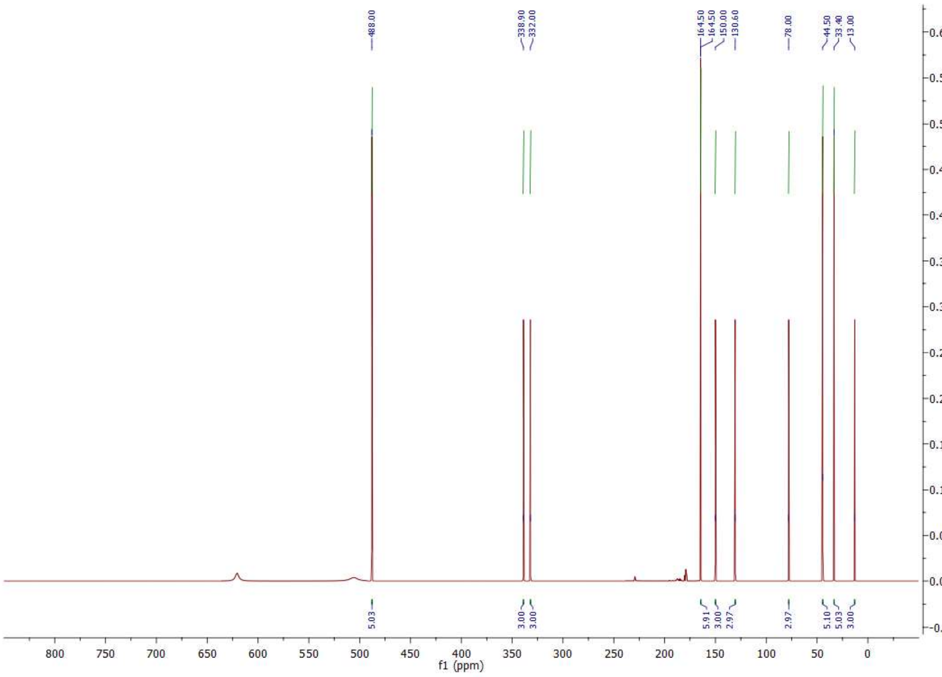

Figure S146. The ^{17}O NMR spectrum of **3DPQ-4**.

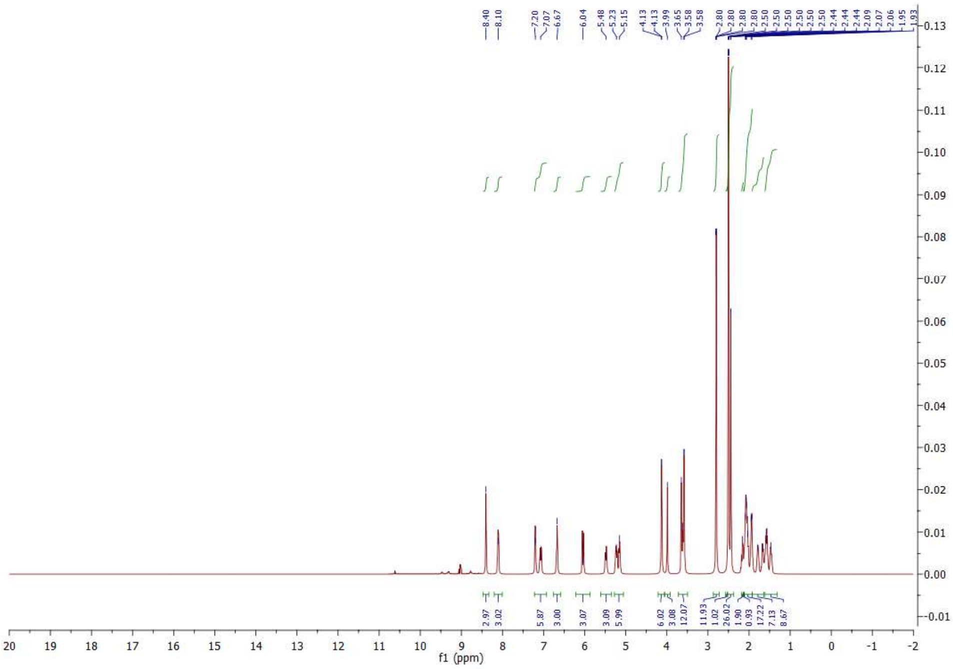

Figure S147. The ^{1}H NMR spectrum of **3DPQ-5**.

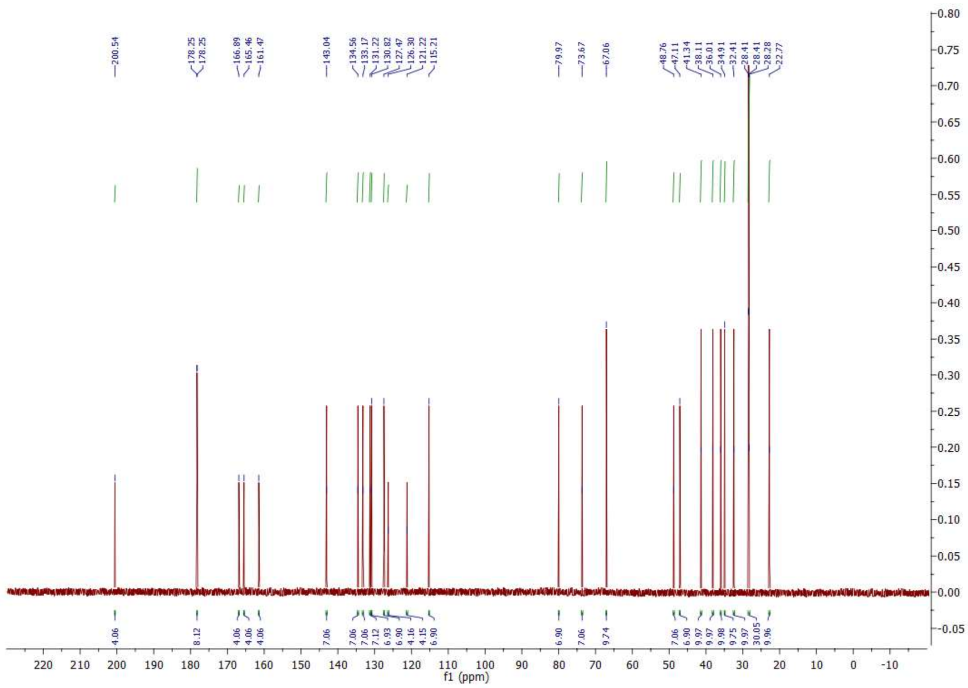

Figure S148. The ^{13}C NMR spectrum of **3DPQ-5**.

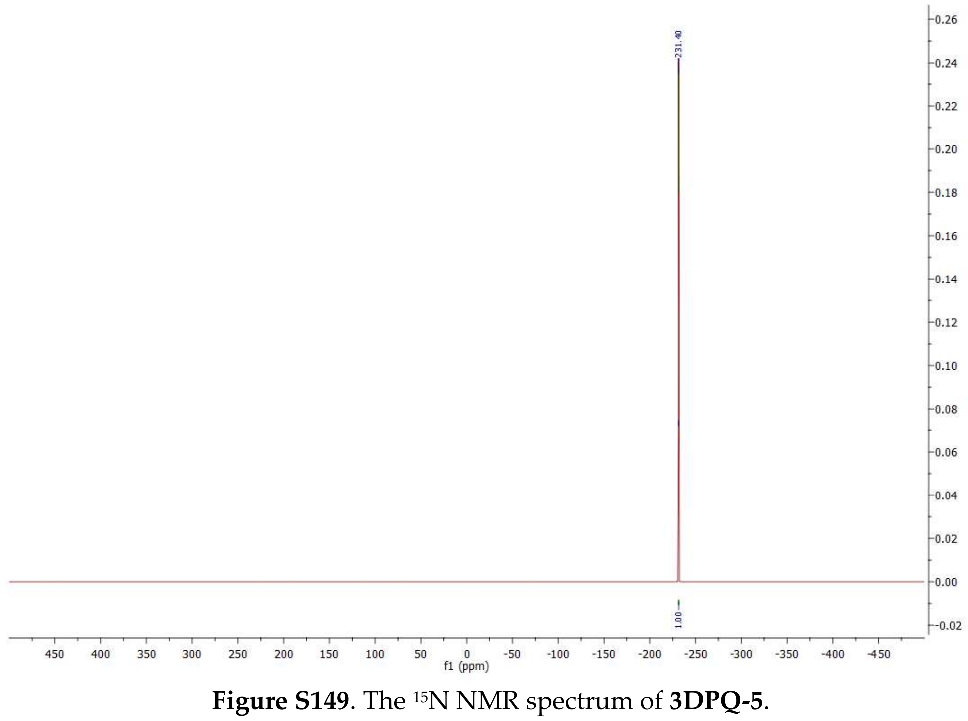

Figure S149. The ^{15}N NMR spectrum of **3DPQ-5**.

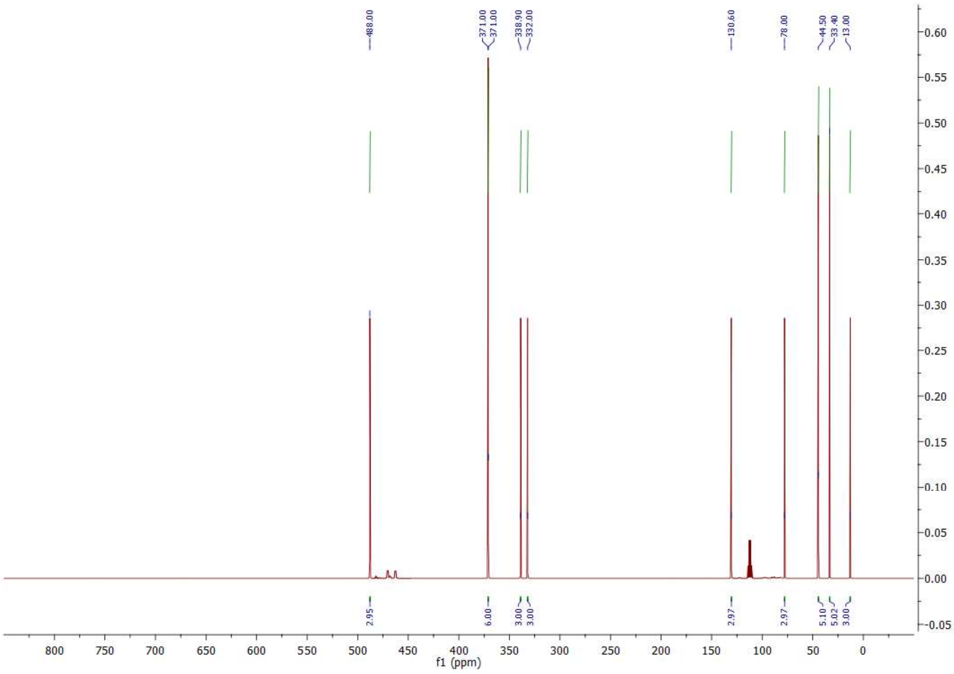

Figure S150. The ^{17}O NMR spectrum of **3DPQ-5**.

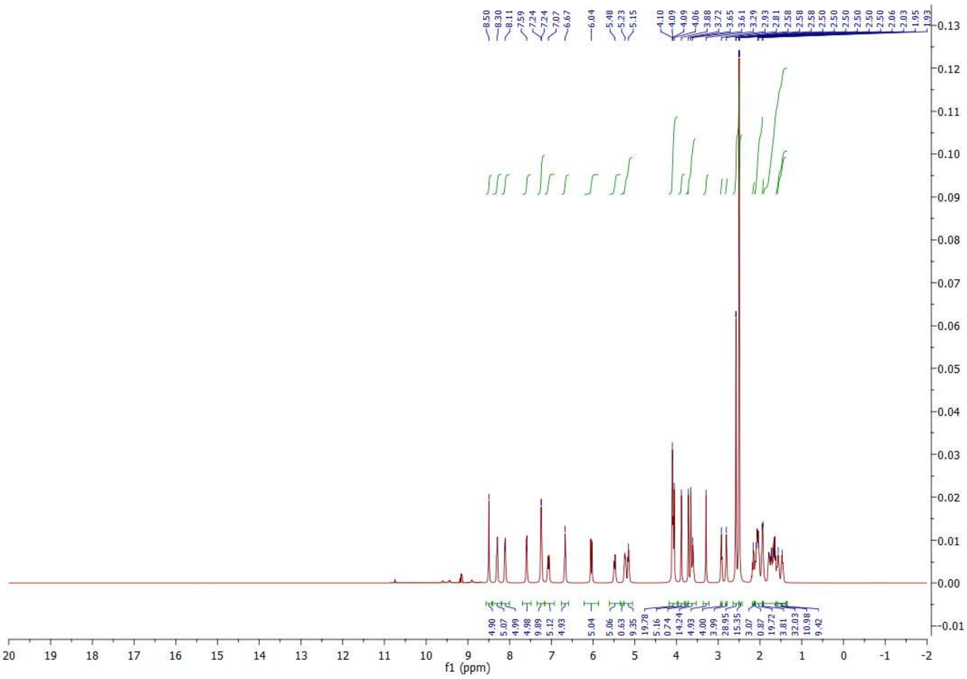

Figure S151. The ^{1}H NMR spectrum of **3DPQ-6**.

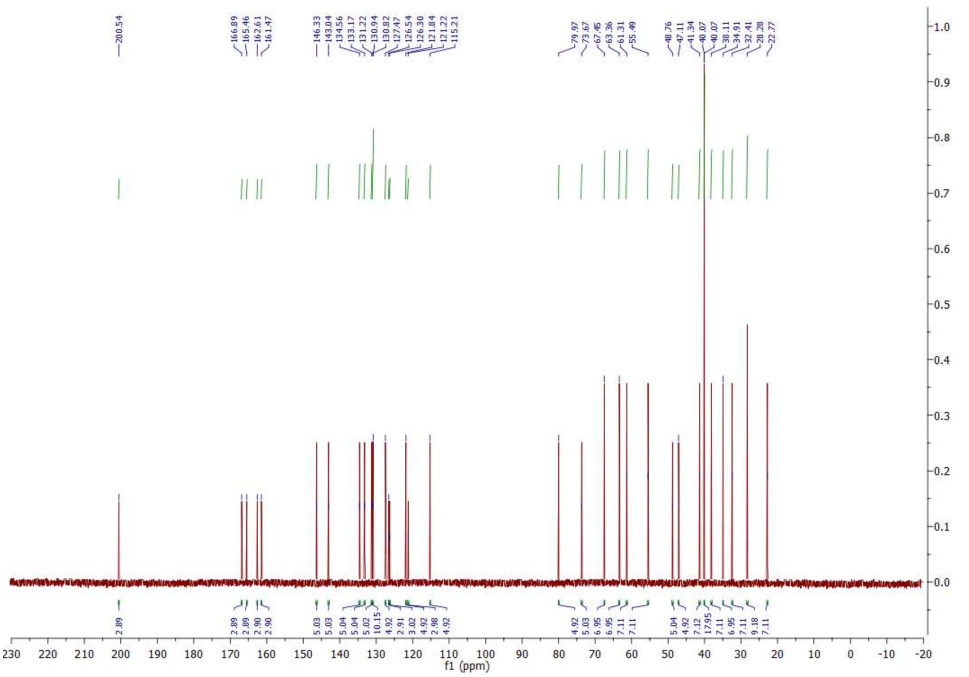

Figure S152. The ^{13}C NMR spectrum of **3DPQ-6**.

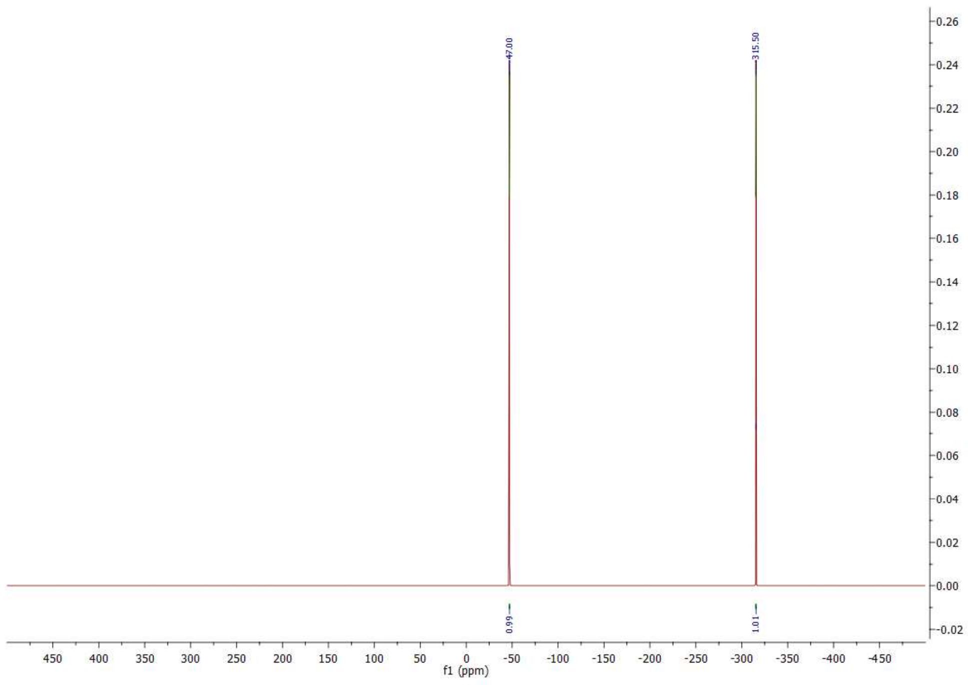

Figure S153. The ^{15}N NMR spectrum of **3DPQ-6**.

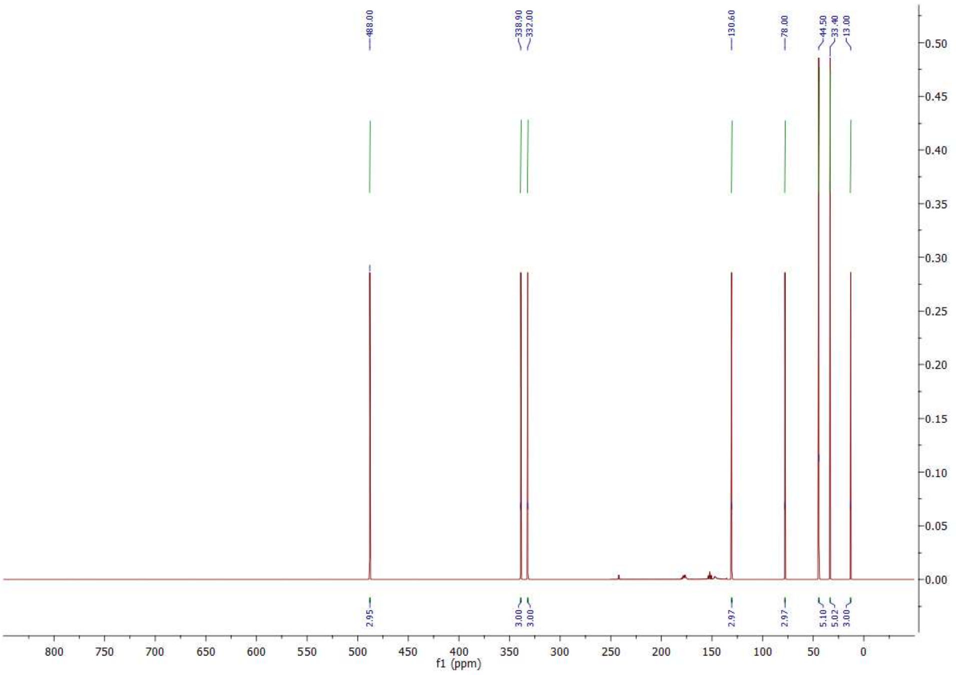

Figure S154. The ^{17}O NMR spectrum of **3DPQ-6**.

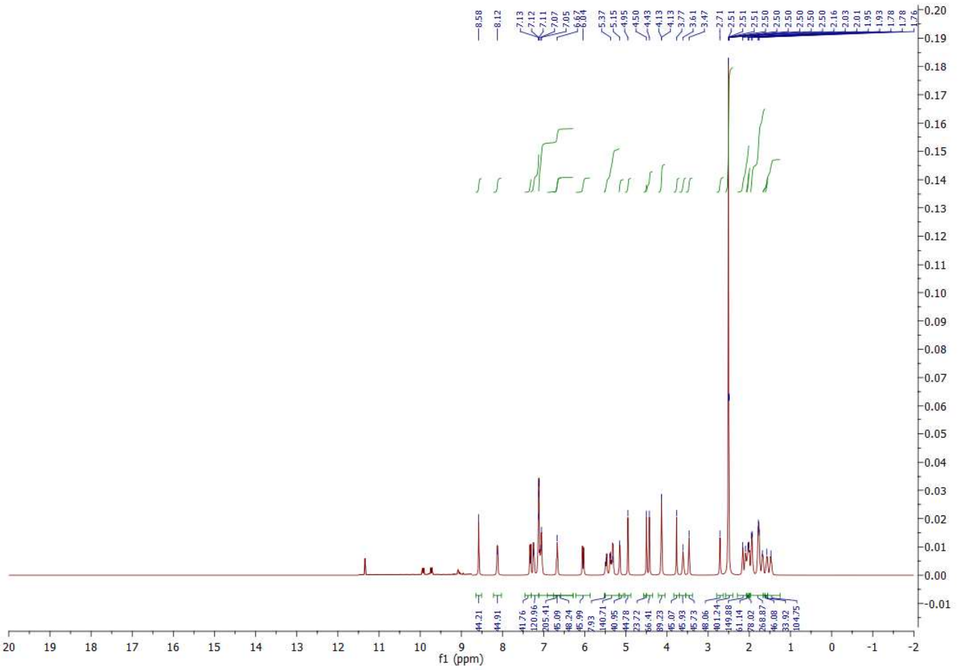

Figure S155. The ^{1}H NMR spectrum of **3DPQ-7**.

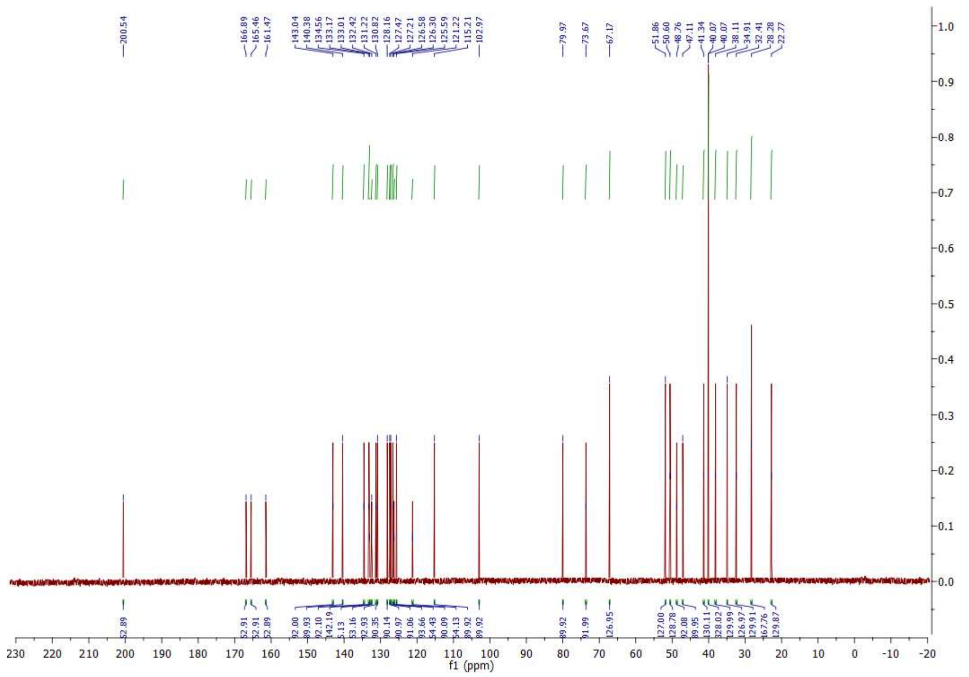

Figure S156. The ^{13}C NMR spectrum of **3DPQ-7**.

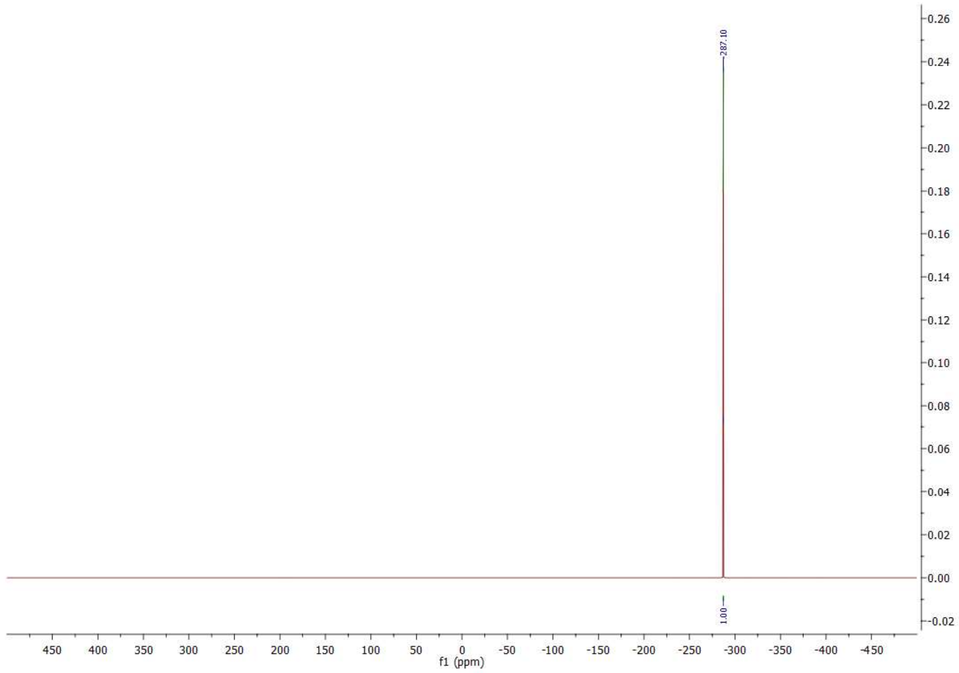

Figure S157. The ^{15}N NMR spectrum of **3DPQ-7**.

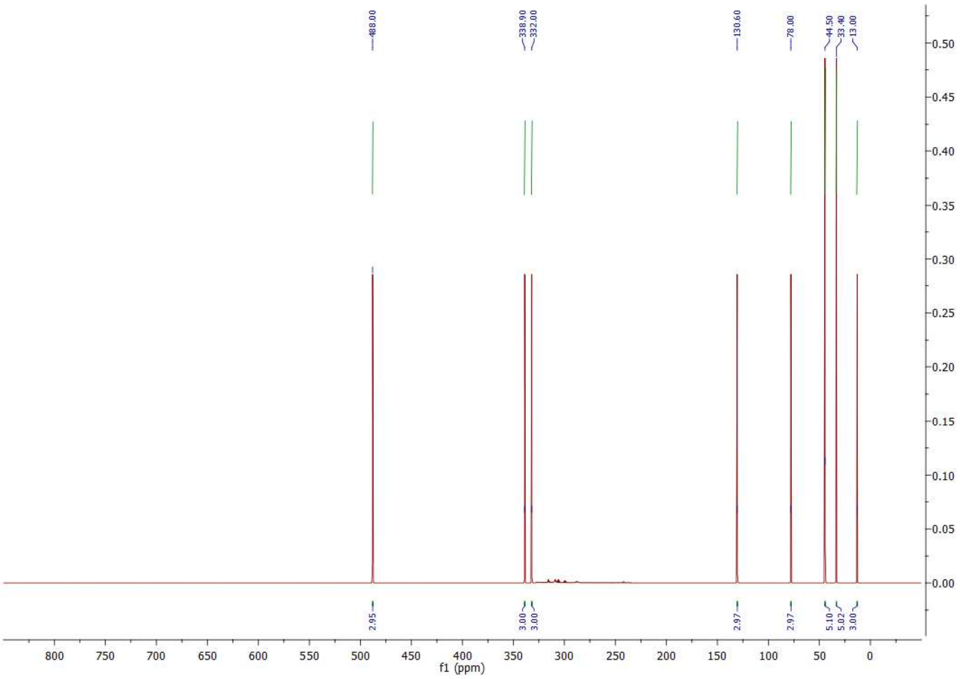

Figure S158. The ^{17}O NMR spectrum of **3DPQ-7**.

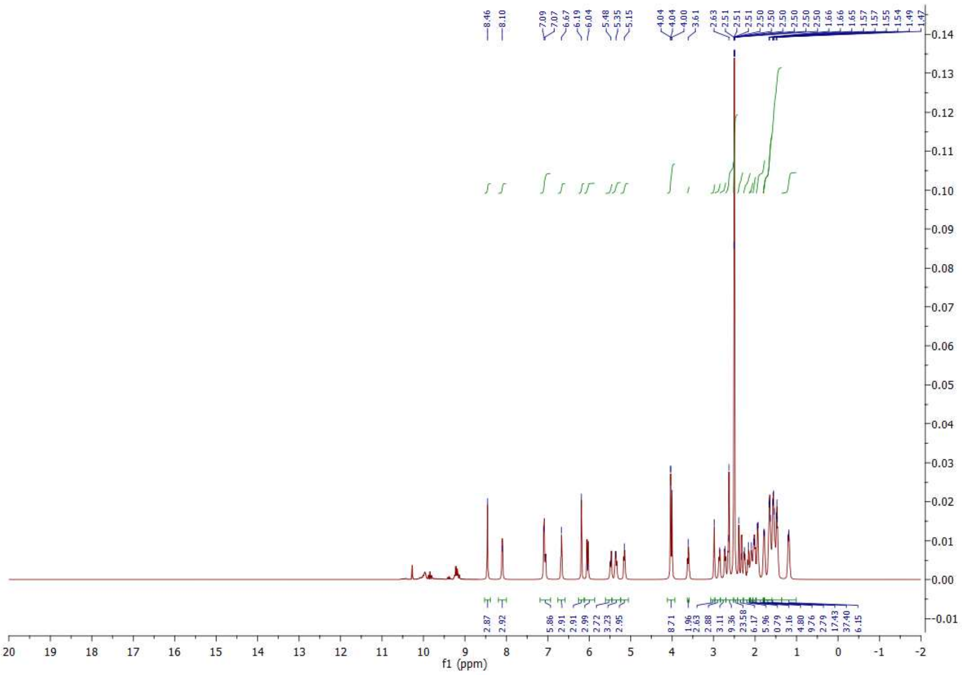

Figure S159. The ^{1}H NMR spectrum of **3DPQ-8**.

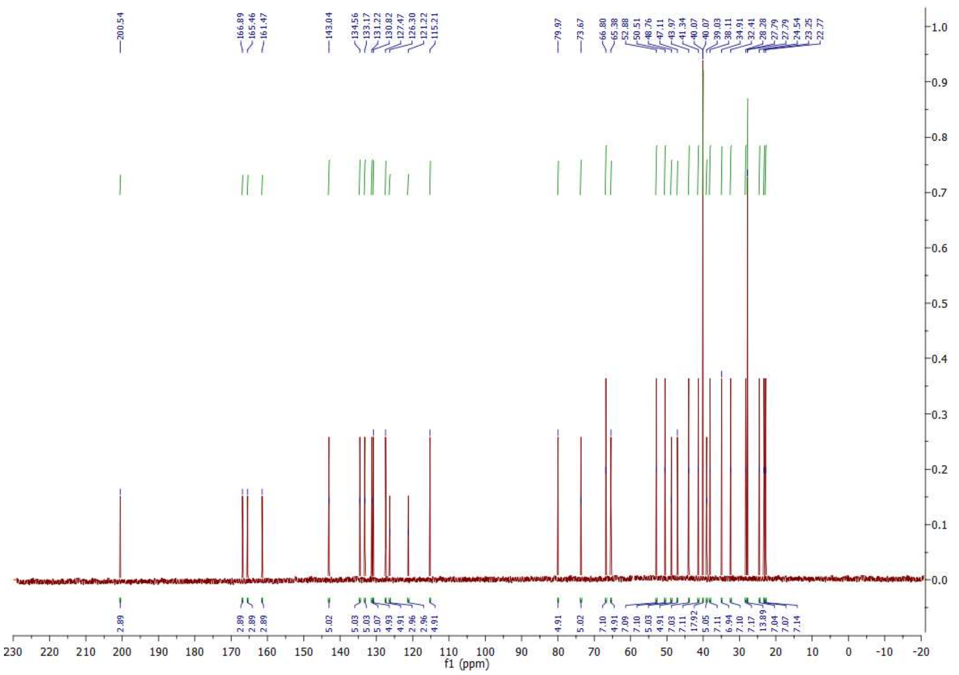

Figure S160. The ^{13}C NMR spectrum of **3DPQ-8**.

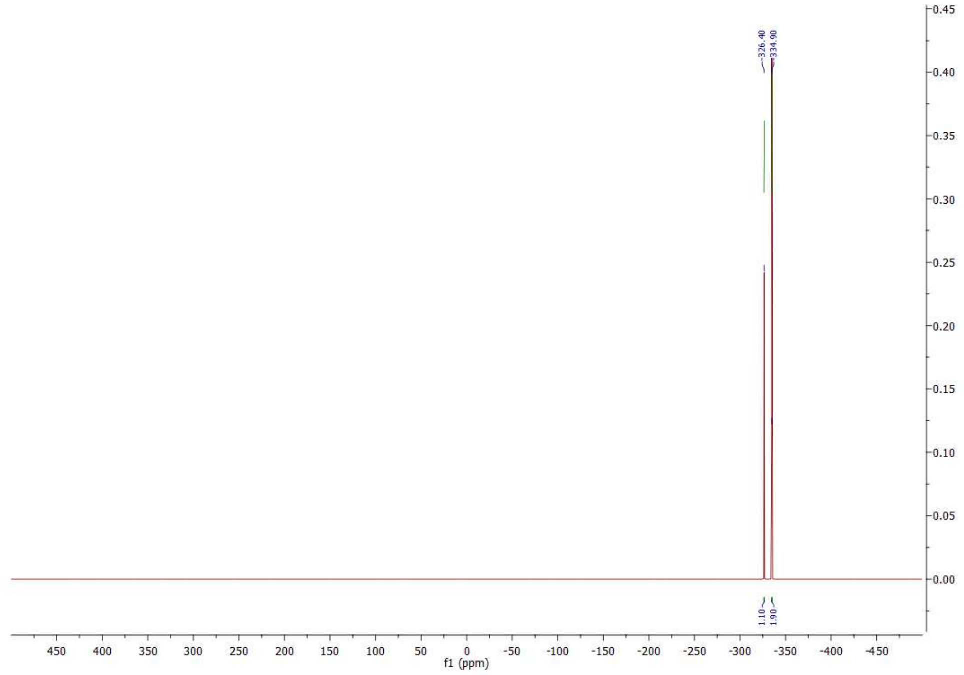

Figure S161. The ^{15}N NMR spectrum of **3DPQ-8**.

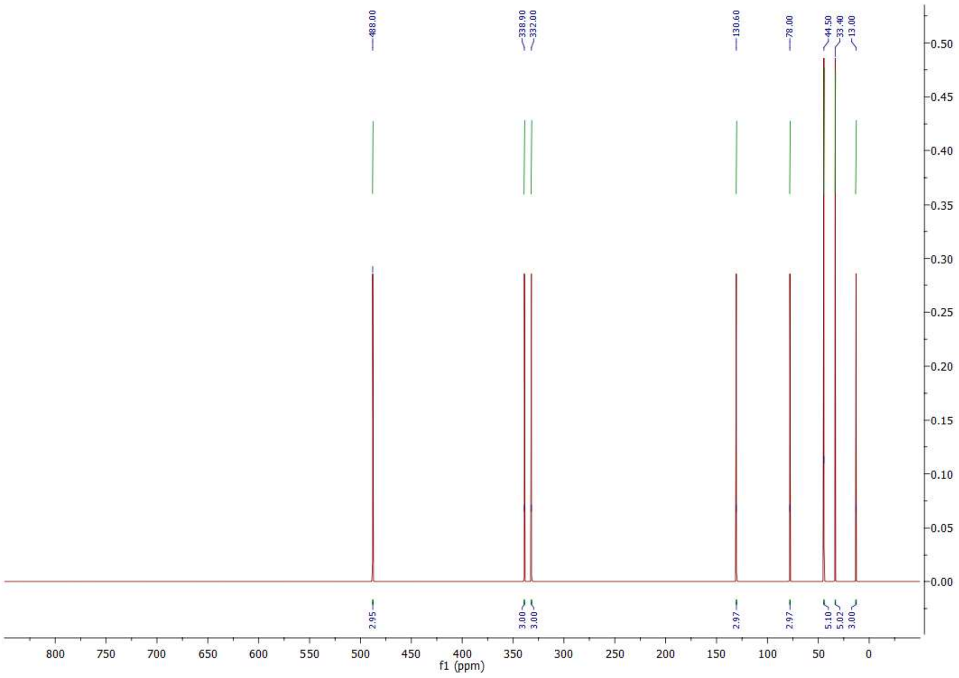

Figure S162. The ^{17}O NMR spectrum of **3DPQ-8**.

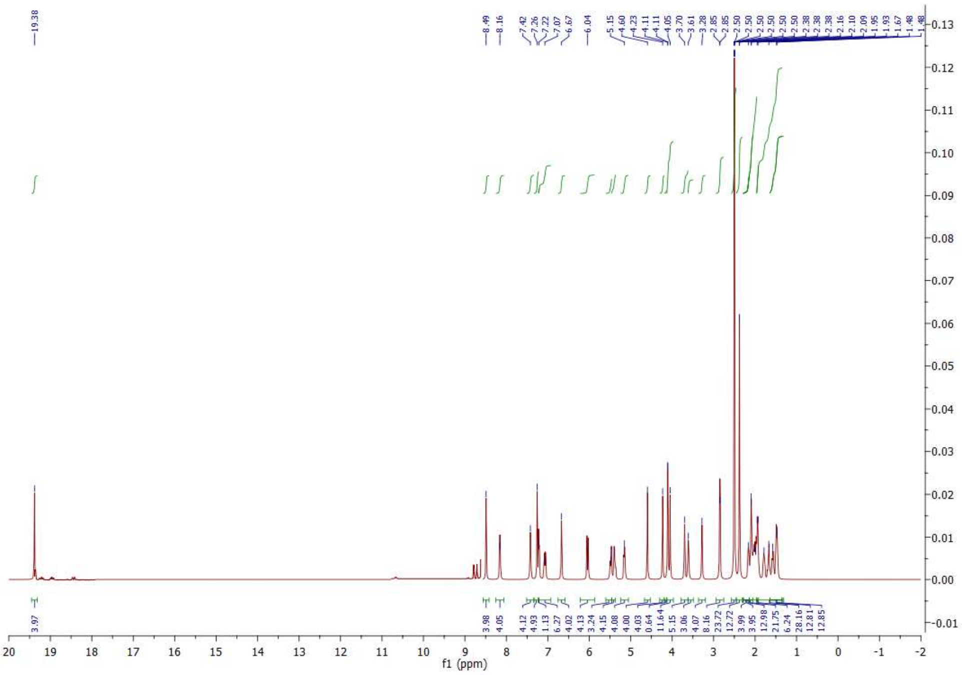

Figure S163. The ^{1}H NMR spectrum of **3DPQ-9**.

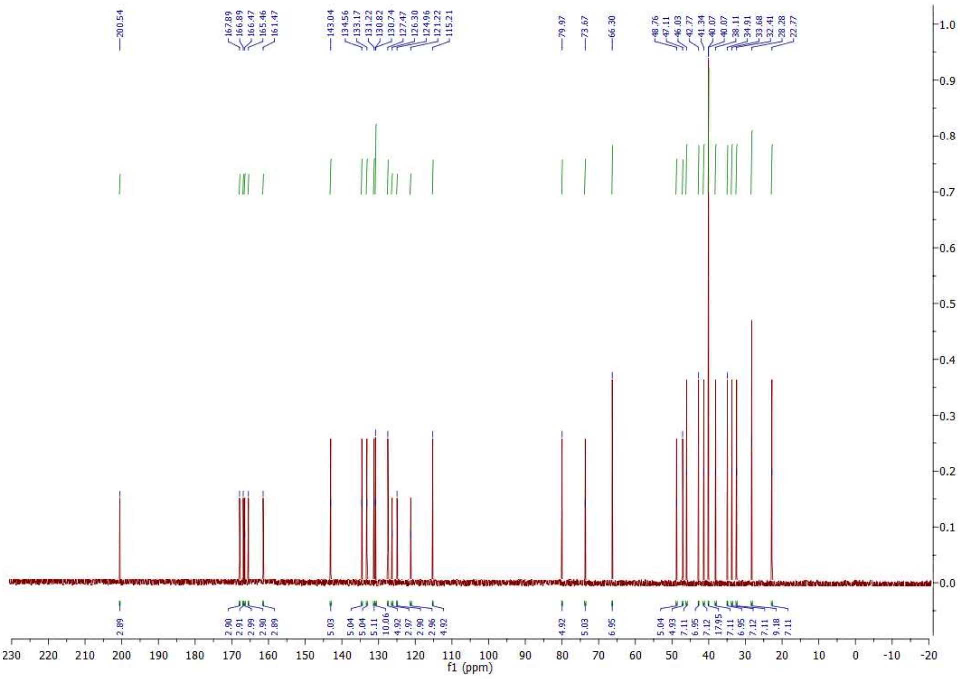

Figure S164. The ^{13}C NMR spectrum of **3DPQ-9**.

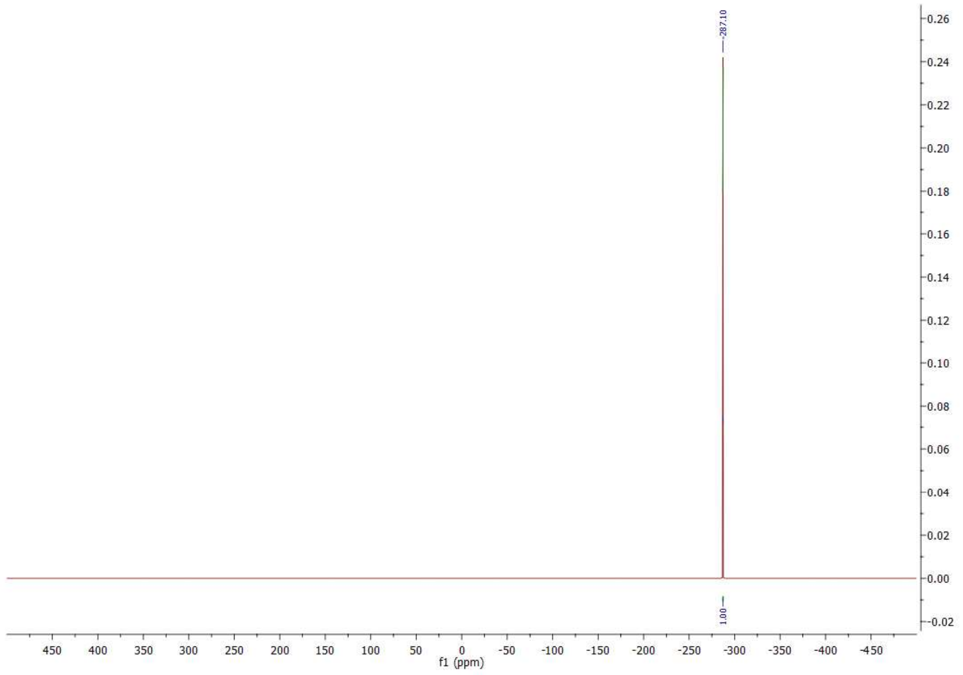

Figure S165. The ^{15}N NMR spectrum of **3DPQ-9**.

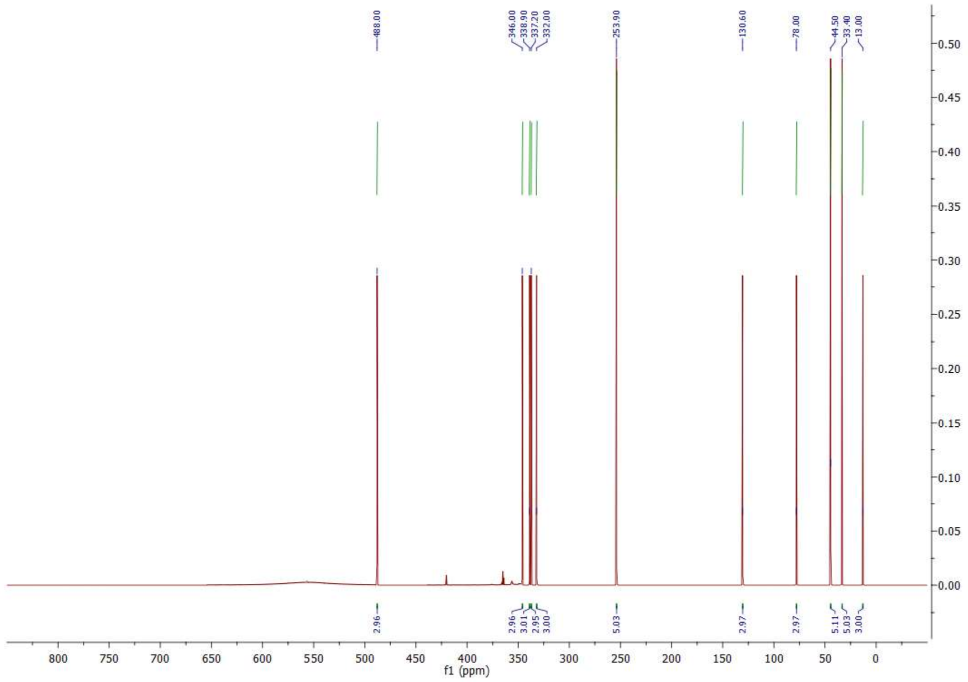

Figure S166. The ^{17}O NMR spectrum of **3DPQ-9**.

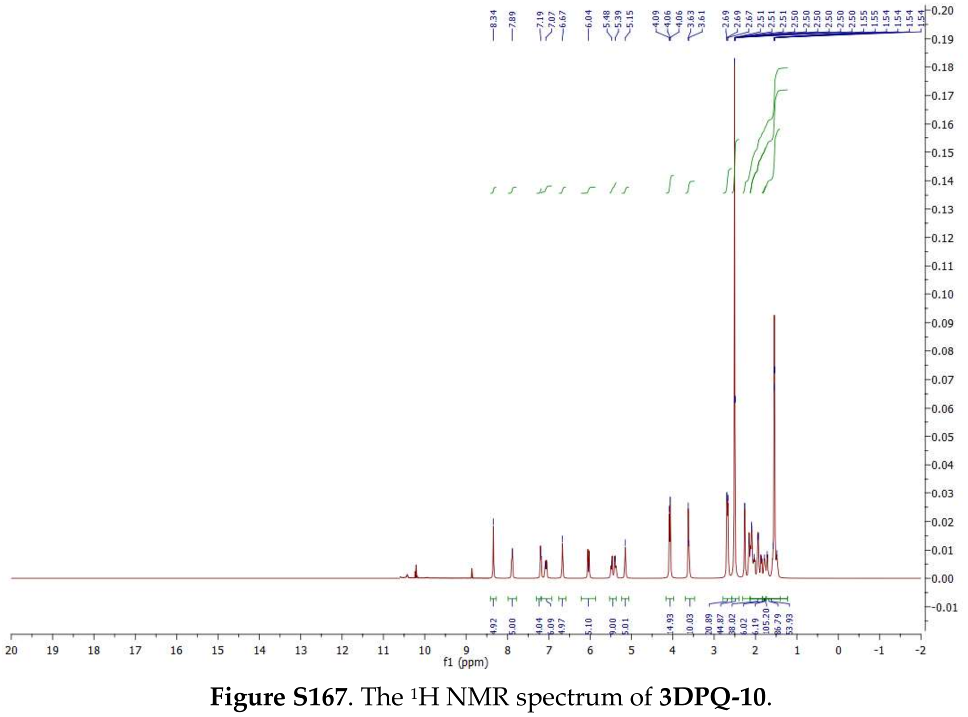

Figure S167. The ^{1}H NMR spectrum of **3DPQ-10**.

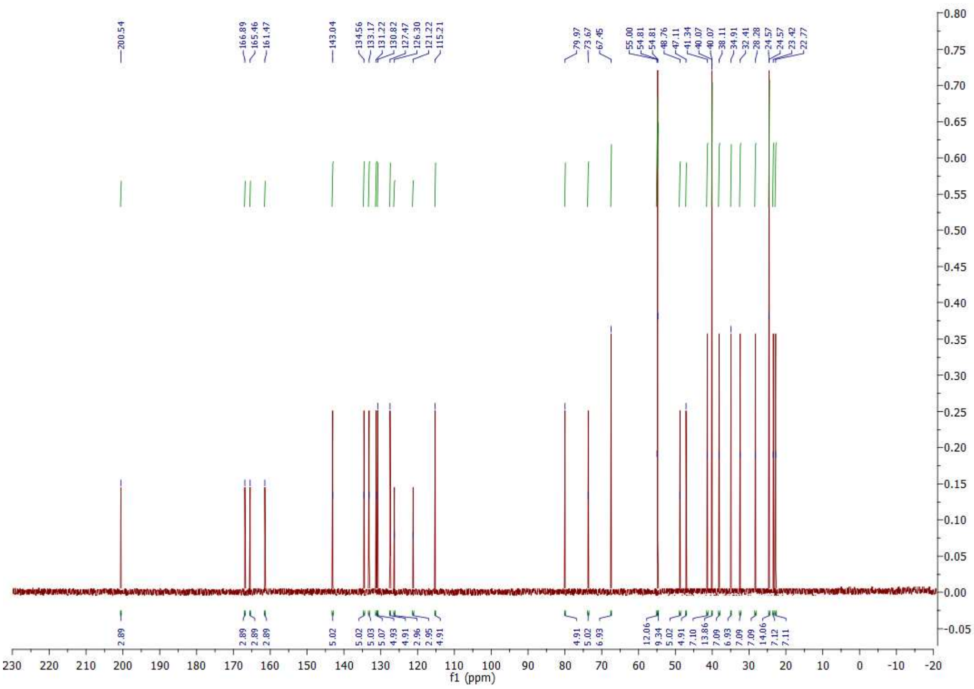

Figure S168. The ^{13}C NMR spectrum of **3DPQ-10**.

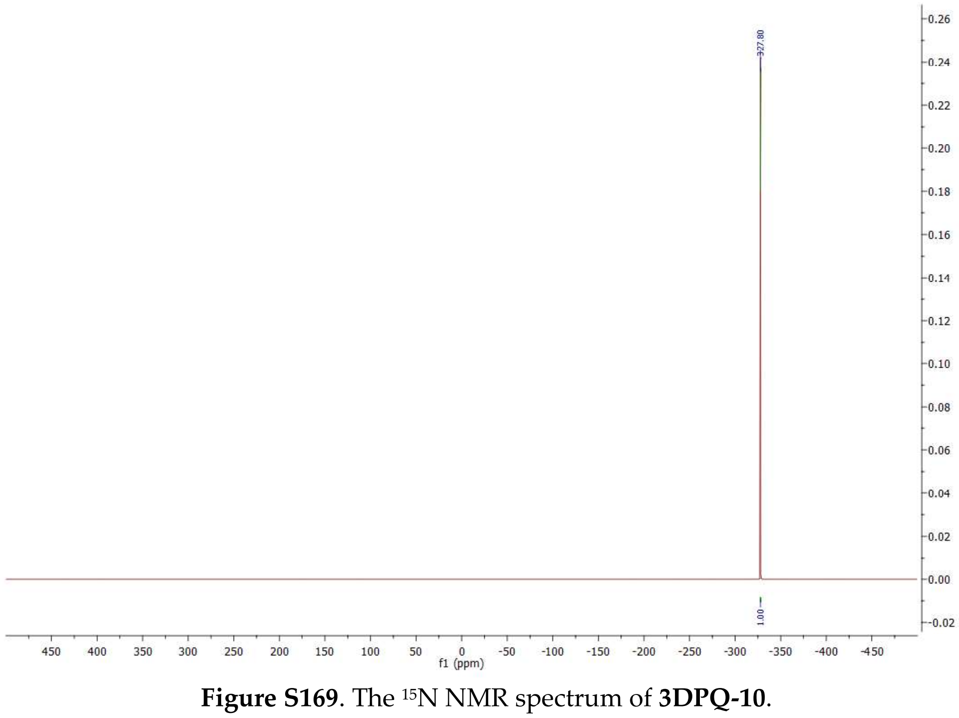

Figure S169. The ^{15}N NMR spectrum of **3DPQ-10**.

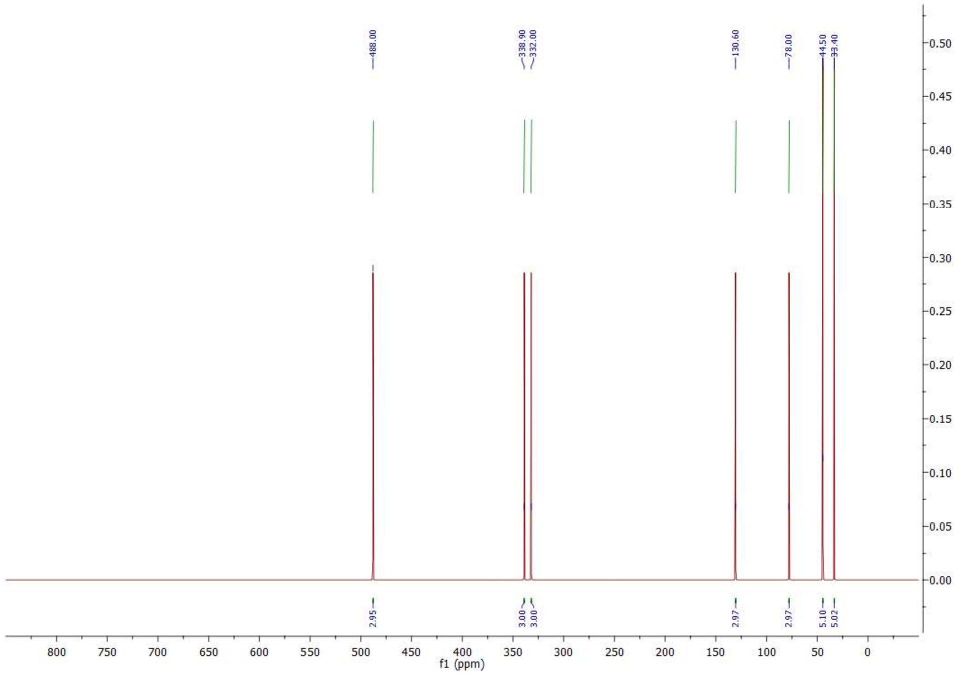

Figure S170. The ^{17}O NMR spectrum of **3DPQ-10**.

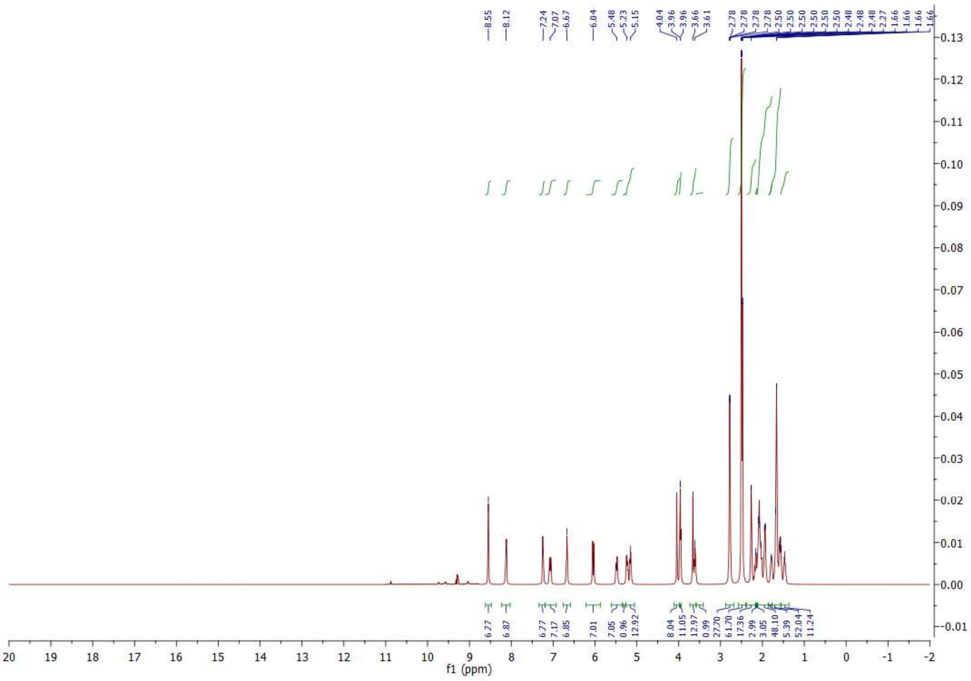

Figure S171. The ^{1}H NMR spectrum of **3DPQ-11**.

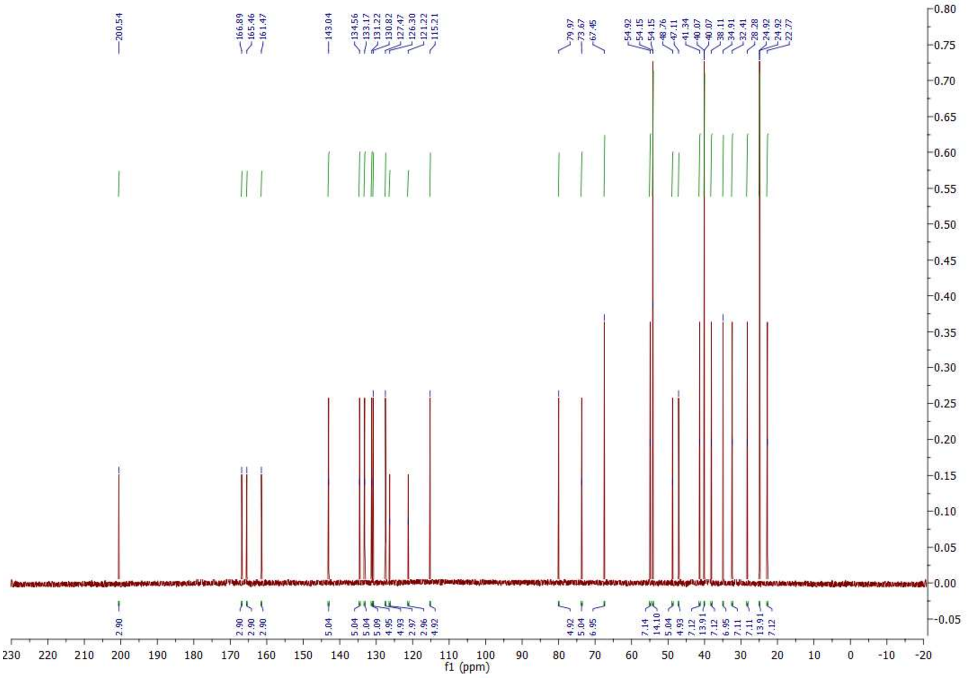

Figure S172. The ^{13}C NMR spectrum of **3DPQ-11**.

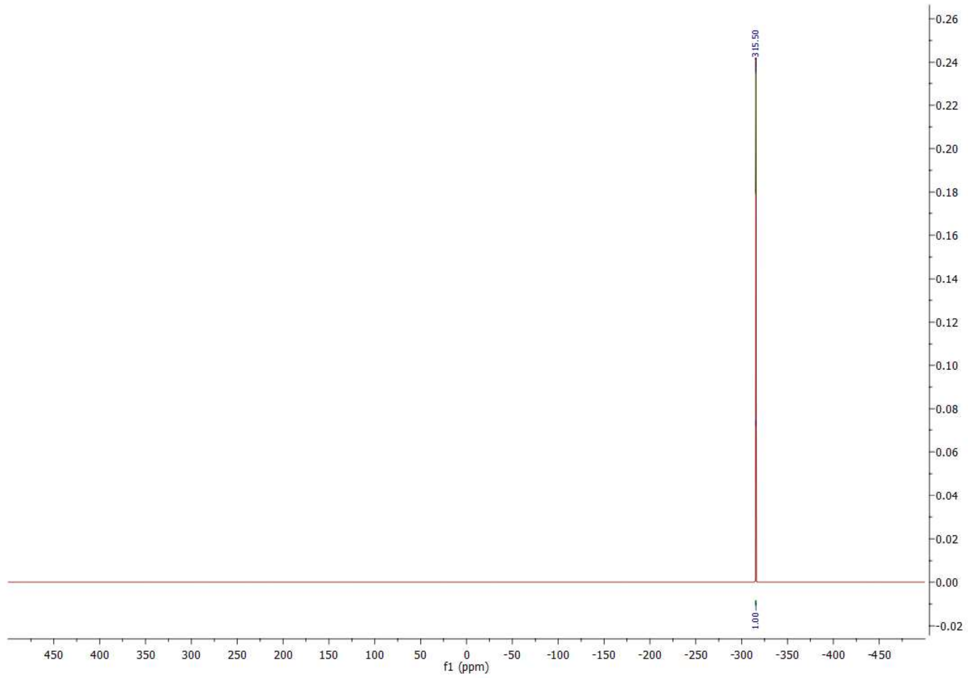

Figure S173. The ^{15}N NMR spectrum of **3DPQ-11**.

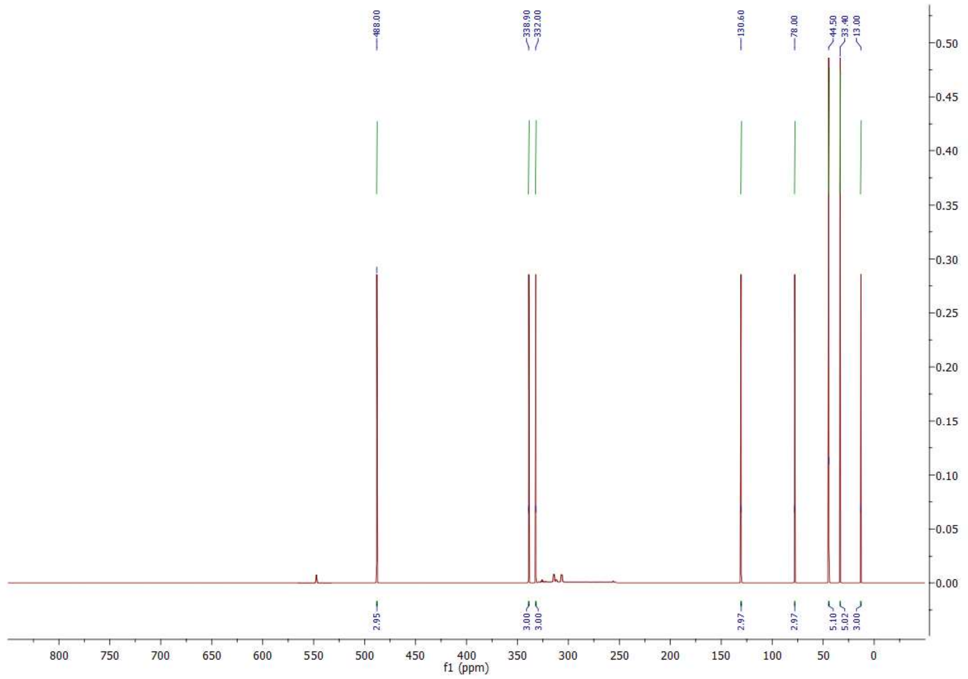

Figure S174. The ^{17}O NMR spectrum of **3DPQ-11**.

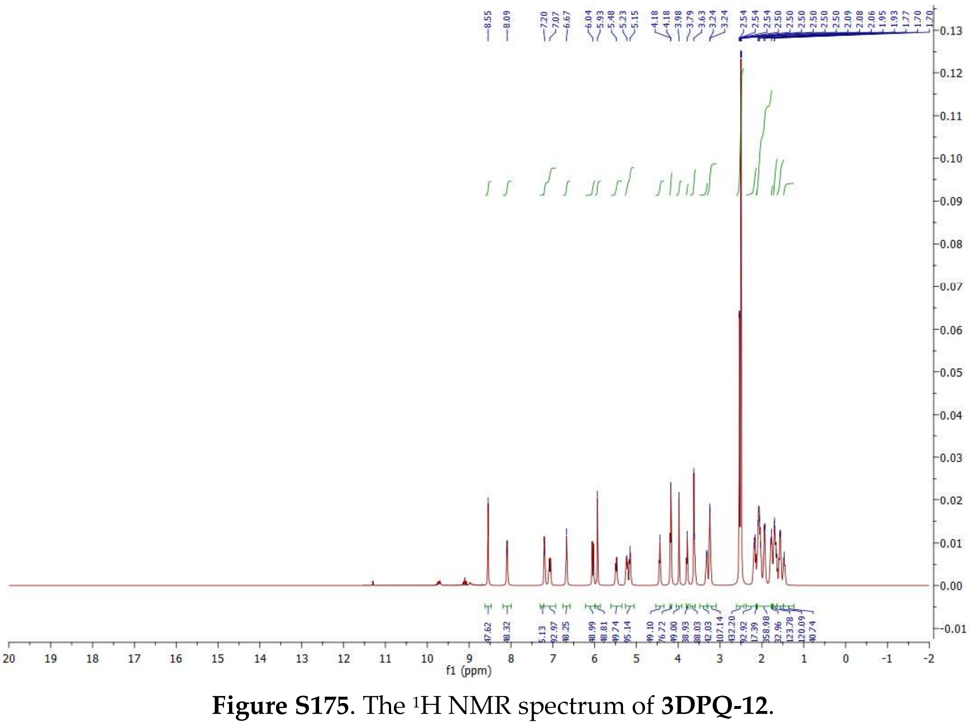

Figure S175. The ^{1}H NMR spectrum of **3DPQ-12**.

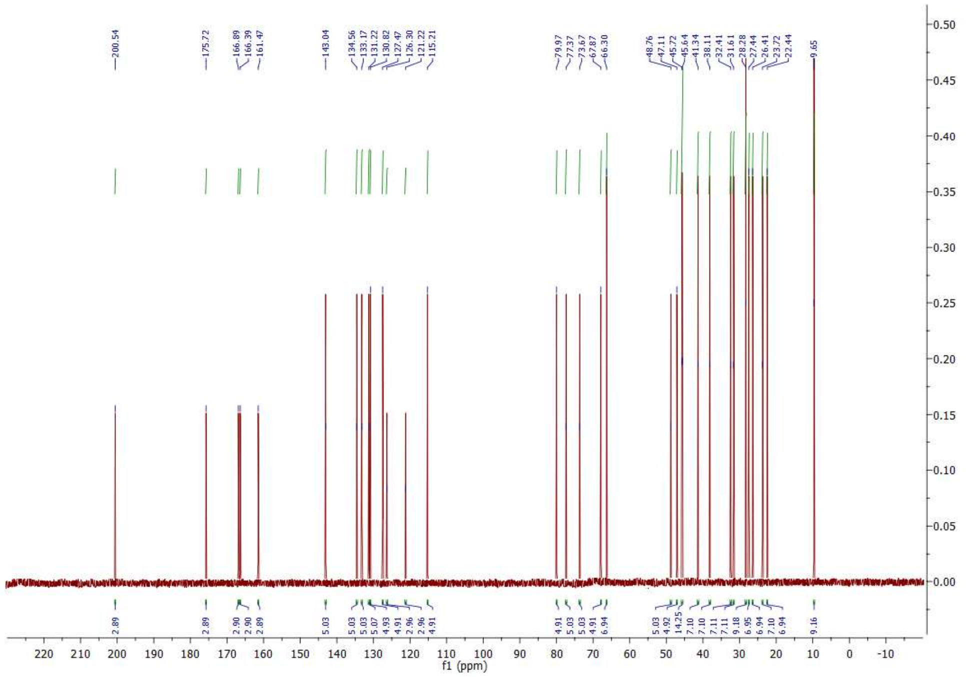

Figure S176. The ^{13}C NMR spectrum of **3DPQ-12**.

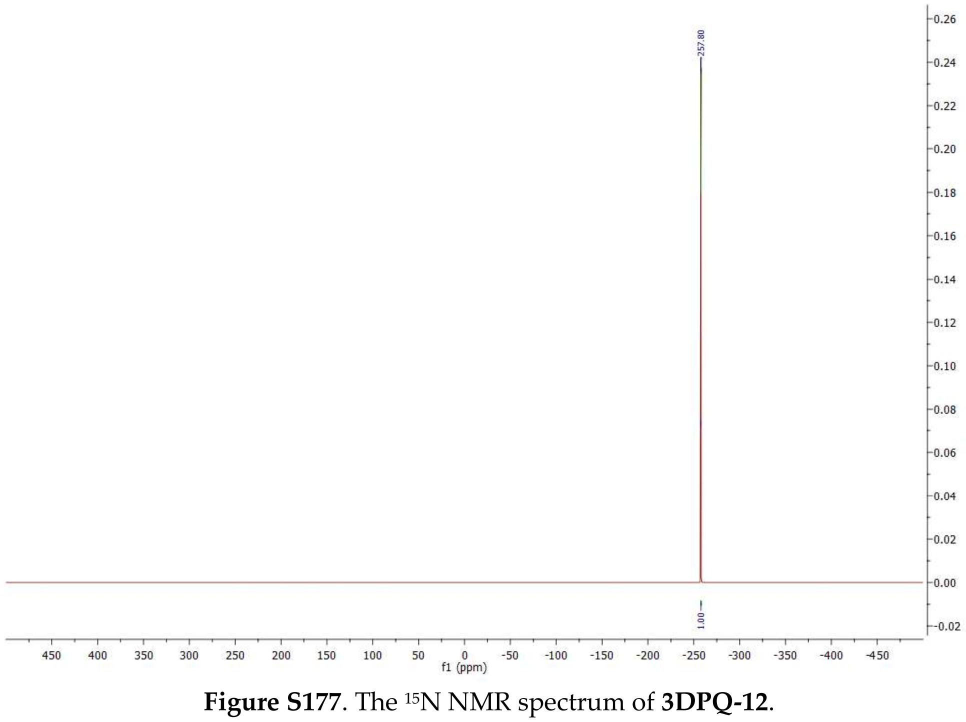

Figure S177. The ^{15}N NMR spectrum of **3DPQ-12**.

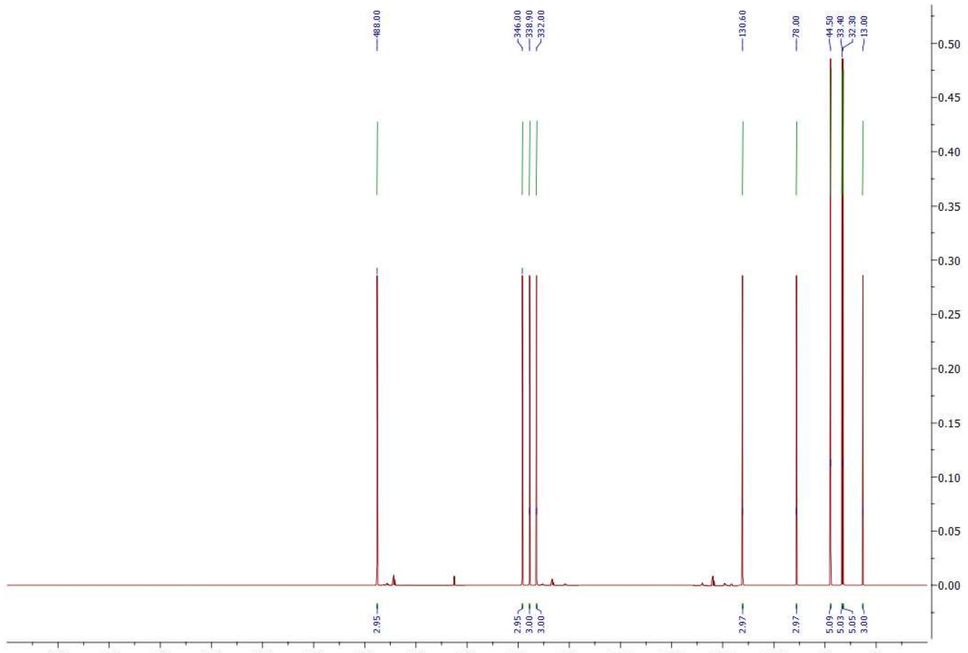

Figure S178. The ^{17}O NMR spectrum of **3DPQ-12**.

HPLC REPORT	
Compound ID	: 3DPQ-1
Filename/Sample ID	: D:\HPLC\2021\September\3DPQ-1a
Method Name	: D:\method\2021\MILAN-3dpq.met
Instrument and Column	: Agilent 1260 Infinity System, Agilent Zorbax AB-C18
Run Time	: 9/20/2021/08:10:37

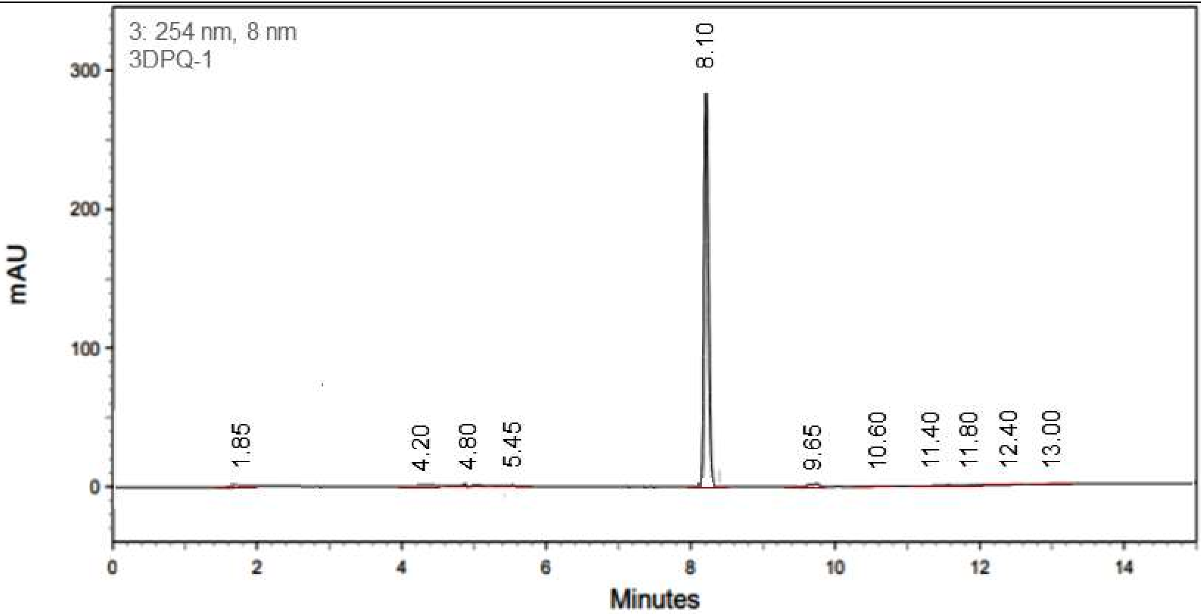

254 nm

Retention time	*Height*	*Area*	*Area Percent*
1.85	1928	5714	0.36
4.20	1795	5632	0.25
4.80	1759	5234	0.17
5.45	1495	5236	0.10
8.10	426953	1185632	98.01
9.65	1655	5342	0.18
10.60	1429	5146	0.35
11.40	1412	5339	0.19
11.80	1235	5623	0.14
12.40	1129	5842	0.11
13.00	1265	5128	0.14

Figure S179. The HPLC spectrum of **3DPQ-1**

HPLC REPORT	
Compound ID	: 3DPQ-2
Filename/Sample ID	: D:\HPLC\2021\September\3DPQ-2
Method Name	: D:\method\2021\MILAN-3dpq.met
Instrument and Column	: Agilent 1260 Infinity System, Agilent Zorbax AB-C18
Run Time	: 9/20/2021/08:30:47

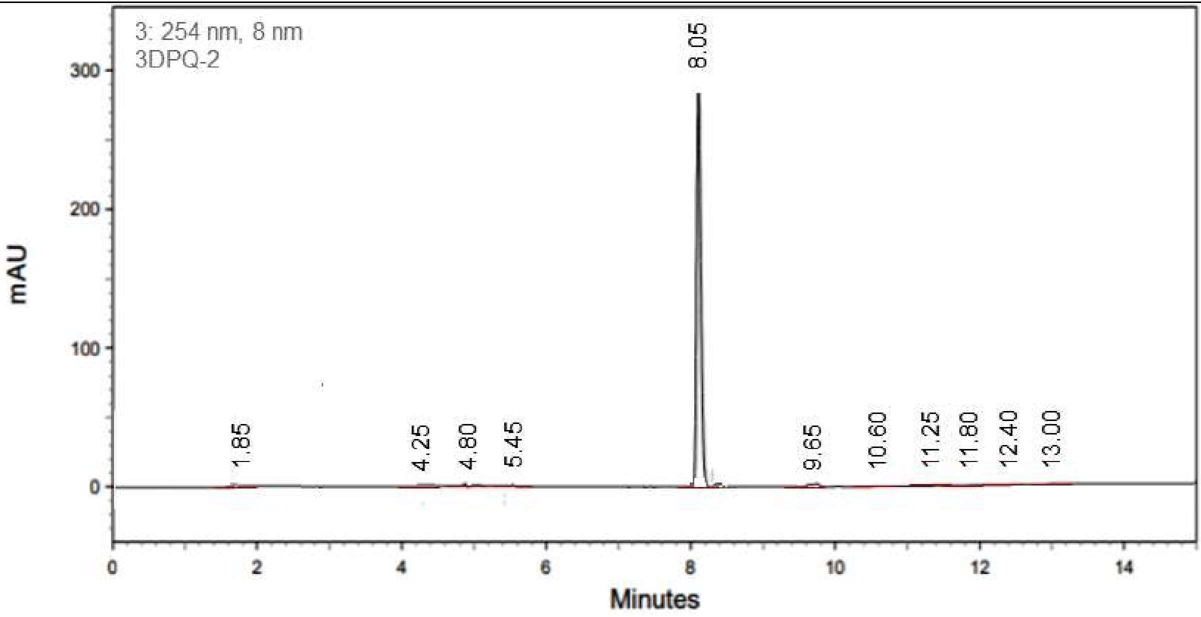

254 nm

Retention time	*Height*	*Area*	*Area Percent*
1.85	1913	5756	0.32
4.25	1784	5652	0.21
4.80	1742	5217	0.14
5.45	1456	5211	0.13
8.05	423453	1262941	98.13
9.65	1641	5128	0.13
10.60	1433	5163	0.31
11.25	1427	5128	0.12
11.80	1241	5584	0.17
12.40	1131	5518	0.15
13.00	1270	5228	0.19

Figure S180. The HPLC spectrum of **3DPQ-2**

HPLC REPORT	
Compound ID	: 3DPQ-3
Filename/Sample ID	: D:\HPLC\2021\September\3DPQ-3
Method Name	: D:\method\2021\MILAN-3dpq.met
Instrument and Column	: Agilent 1260 Infinity System, Agilent Zorbax AB-C18
Run Time	: 9/20/2021/08:55:47

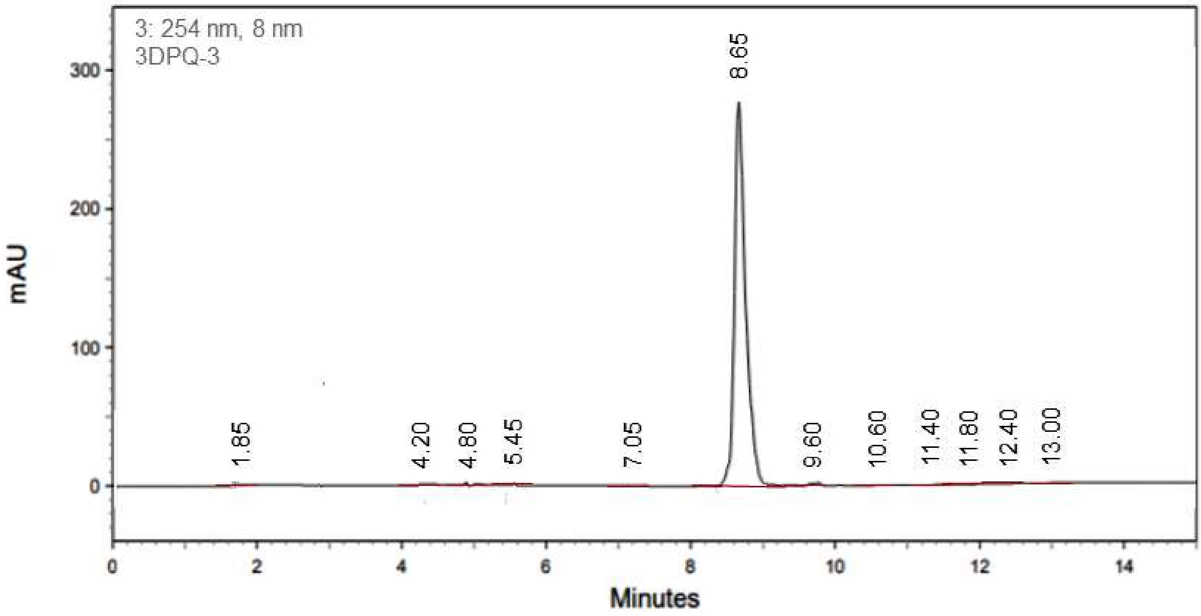

254 nm

Retention time	*Height*	*Area*	*Area Percent*
1.85	1919	5763	0.29
4.20	1752	5628	0.19
4.80	1728	5249	0.15
5.45	1417	5244	0.14
7.05	1366	4958	0.12
8.66	435214	1285615	98.13
9.60	1651	5539	0.16
10.60	1427	5142	0.29
11.40	1446	5538	0.14
11.80	1281	5451	0.15
12.40	1176	5259	0.17
13.00	1228	5895	0.21

Figure S181. The HPLC spectrum of **3DPQ-3**

HPLC REPORT

Compound ID	: 3DPQ-4
Filename/Sample ID	: D:\HPLC\2021\September\3DPQ-4
Method Name	: D:\method\2021\MILAN-3dpq.met
Instrument and Column	: Agilent 1260 Infinity System, Agilent Zorbax AB-C18
Run Time	: 9/20/2021/09:20:13

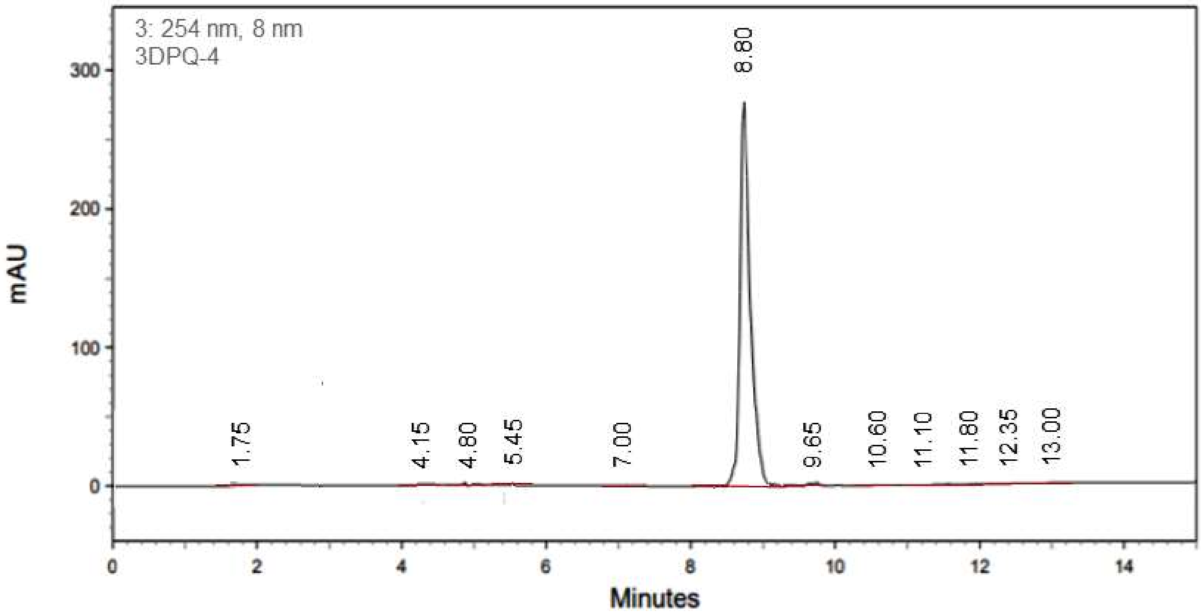

254 nm

Retention time	*Height*	*Area*	*Area Percent*
1.75	1924	5796	0.27
4.15	1769	5645	0.16
4.80	1734	5228	0.11
5.45	1445	5218	0.15
7.00	1358	4974	0.14
8.80	442365	1295361	98.16
9.66	1698	5518	0.18
10.60	1492	5184	0.22
11.10	1418	5519	0.15
11.80	1249	5449	0.12
12.35	1187	5362	0.14
13.00	1245	5695	0.20

Figure S182. The HPLC spectrum of **3DPQ-4**

HPLC REPORT	
Compound ID	: 3DPQ-5
Filename/Sample ID	: D:\HPLC\2021\September\3DPQ-5
Method Name	: D:\method\2021\MILAN-3dpq.met
Instrument and Column	: Agilent 1260 Infinity System, Agilent Zorbax AB-C18
Run Time	: 9/20/2021/09:50:46

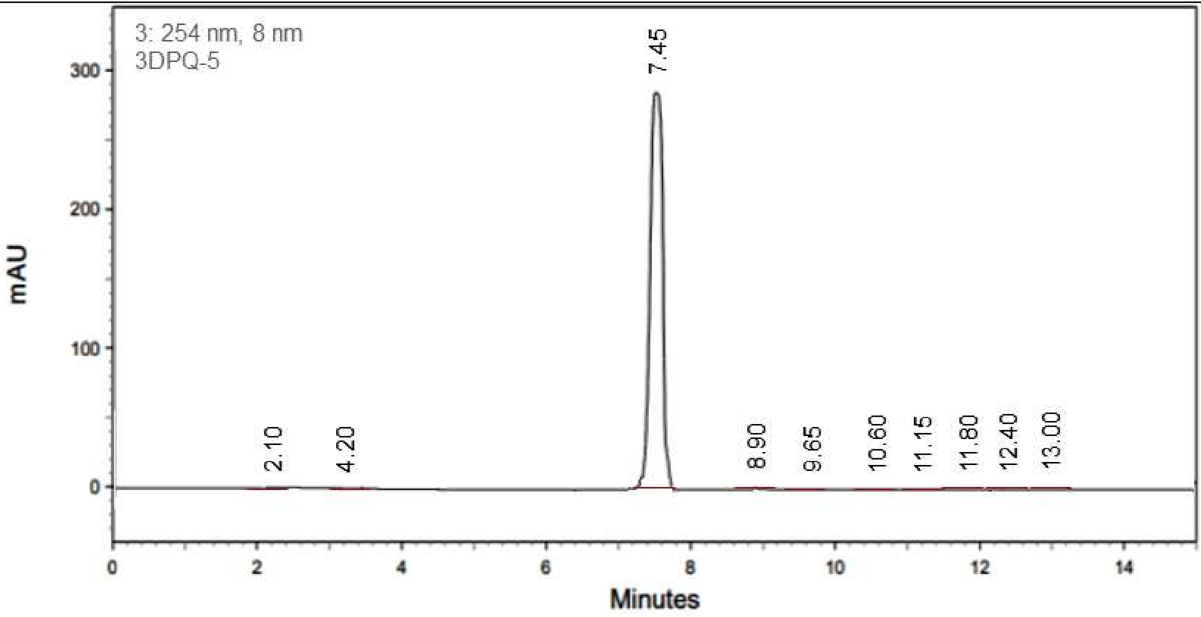

254 nm

Retention time	*Height*	*Area*	*Area Percent*
2.10	1937	5759	0.14
4.20	1716	5618	0.15
7.45	456912	1362510	98.71
8.90	1878	5562	0.12
9.65	1648	5495	0.14
10.60	1419	5289	0.17
11.15	1456	5594	0.14
11.80	1258	5239	0.17
12.40	1167	5528	0.11
13.00	1285	5489	0.15

Figure S183. The HPLC spectrum of **3DPQ-5.**

HPLC REPORT

Compound ID	: 3DPQ-6
Filename/Sample ID	: D:\HPLC\2021\September\3DPQ-6
Method Name	: D:\method\2021\MILAN-3dpq.met
Instrument and Column	: Agilent 1260 Infinity System, Agilent Zorbax AB-C18
Run Time	: 9/20/2021/10:30:17

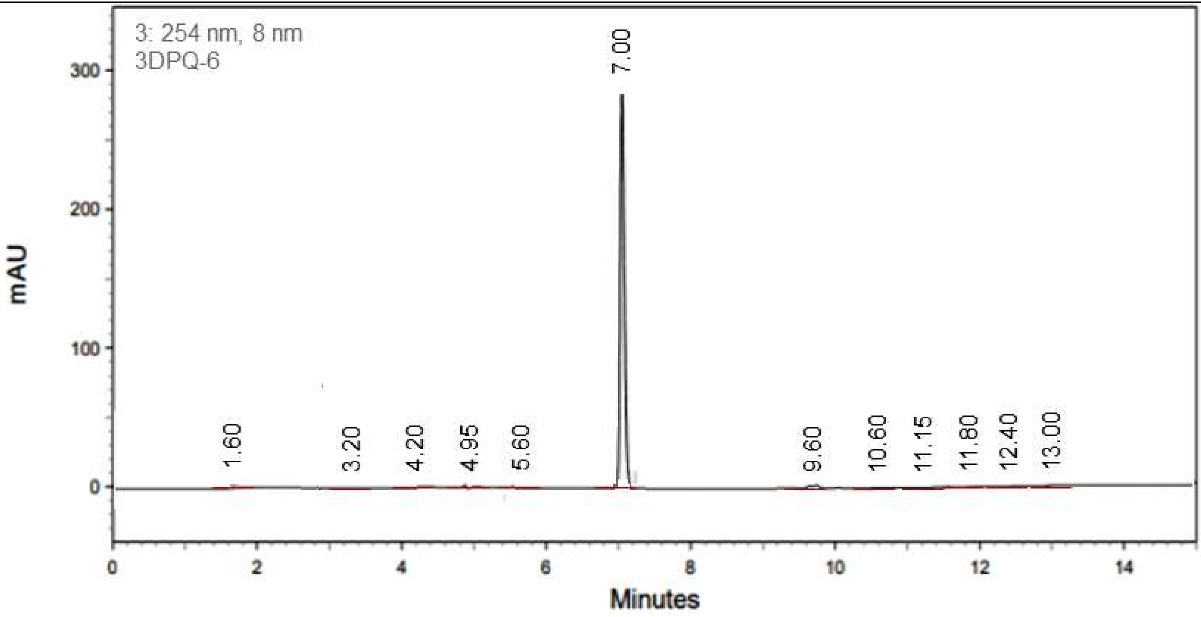

254 nm

Retention time	*Height*	*Area*	*Area Percent*
1.60	1918	5765	0.18
3.20	1756	5642	0.19
4.20	1792	5295	0.14
4.95	1365	5261	0.19
5.60	1312	4949	0.18
7.00	443563	1269541	98.10
9.60	1352	5953	0.14
10.60	1448	5595	0.19
11.15	1419	5892	0.16
11.80	1244	5894	0.19
12.40	1186	5539	0.15
13.00	1247	5519	0.19

Figure S184. The HPLC spectrum of **3DPQ-6.**

HPLC REPORT	
Compound ID	: 3DPQ-7
Filename/Sample ID	: D:\HPLC\2021\September\3DPQ-7
Method Name	: D:\method\2021\MILAN-3dpq.met
Instrument and Column	: Agilent 1260 Infinity System, Agilent Zorbax AB-C18
Run Time	: 9/20/2021/10:55:24

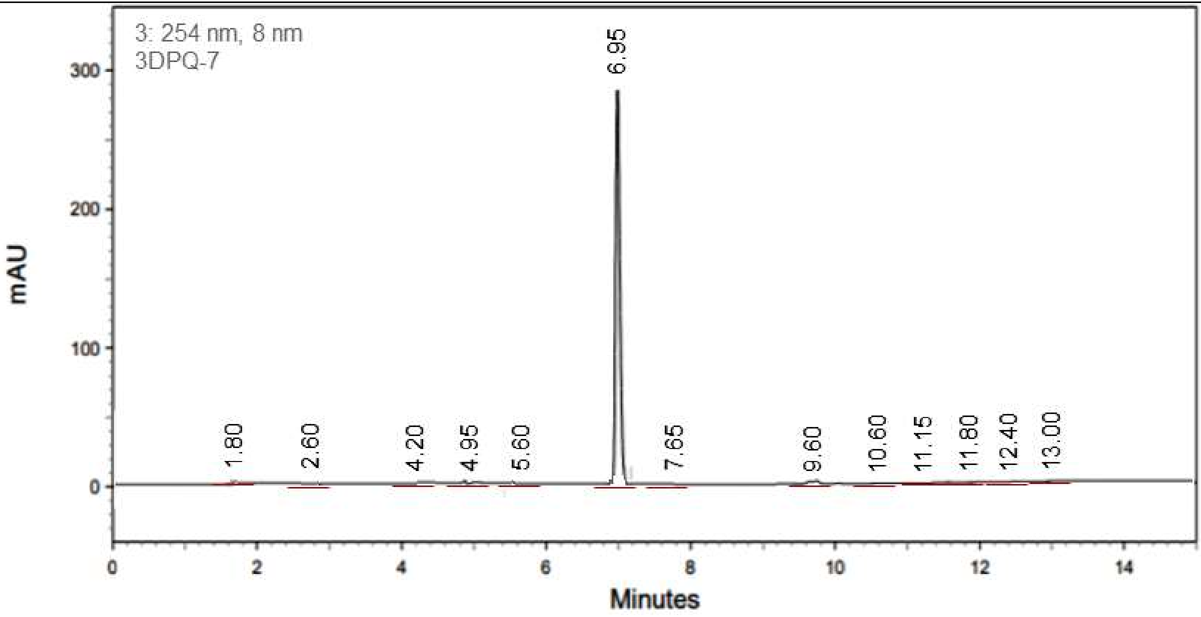

254 nm

Retention time	*Height*	*Area*	*Area Percent*
1.80	1900	5652	0.16
2.60	1775	5518	0.14
4.20	1741	5202	0.18
4.95	1385	5539	0.15
5.60	1341	4532	0.19
6.95	451249	1128562	98.14
7.65	1421	4569	0.17
9.60	1846	5459	0.17
10.60	1512	5845	0.19
11.15	1624	5746	0.15
11.80	1589	5825	0.14
12.40	1512	5365	0.16
13.00	1556	5152	0.21

Figure S185. The HPLC spectrum of **3DPQ-7.**

HPLC REPORT	
Compound ID	: 3DPQ-8
Filename/Sample ID	: D:\HPLC\2021\September\3DPQ-8
Method Name	: D:\method\2021\MILAN-3dpq.met
Instrument and Column	: Agilent 1260 Infinity System, Agilent Zorbax AB-C18
Run Time	: 9/20/2021/11:30:23

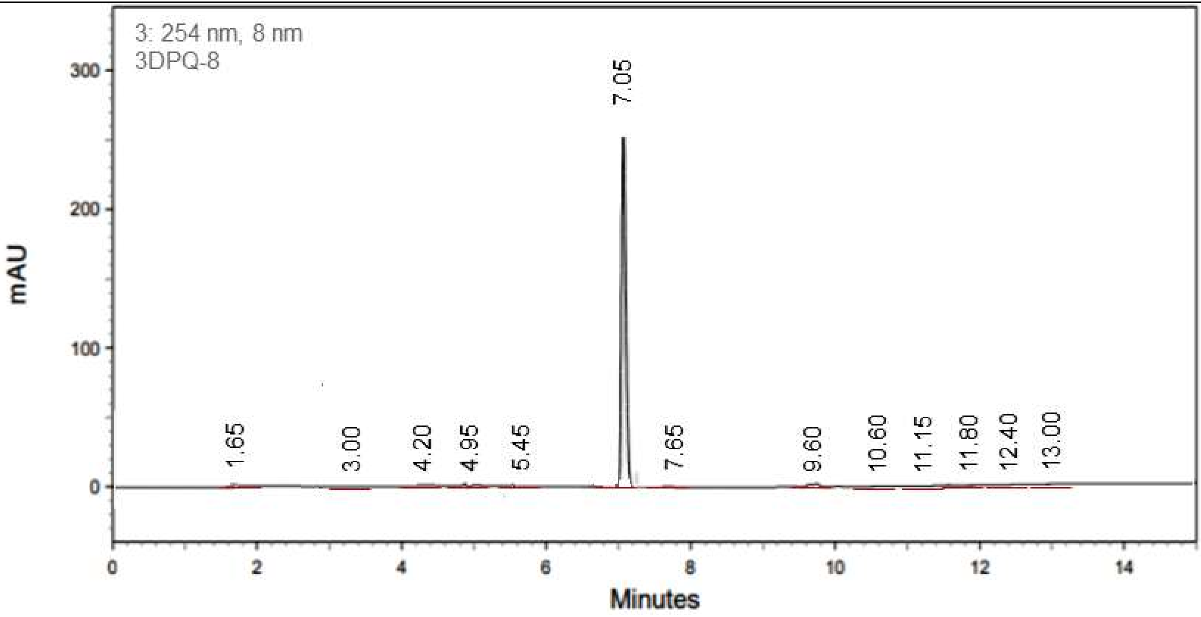

254 nm

Retention time	*Height*	*Area*	*Area Percent*
1.65	1895	5591	0.17
3.00	1802	5562	0.11
4.20	1765	5594	0.12
4.95	1491	5121	0.14
5.45	1341	4559	0.14
7.05	449532	1245165	98.31
7.65	1539	4632	0.11
9.60	1845	4532	0.14
10.60	1591	5433	0.13
11.15	1695	5542	0.16
11.80	1592	5984	0.16
12.40	1533	5543	0.12
13.00	1551	5421	0.18

Figure S186. The HPLC spectrum of **3DPQ-8.**

HPLC REPORT	
Compound ID	: 3DPQ-9
Filename/Sample ID	: D:\HPLC\2021\September\3DPQ-9
Method Name	: D:\method\2021\MILAN-3dpq.met
Instrument and Column	: Agilent 1260 Infinity System, Agilent Zorbax AB-C18
Run Time	: 9/20/2021/11:50:17

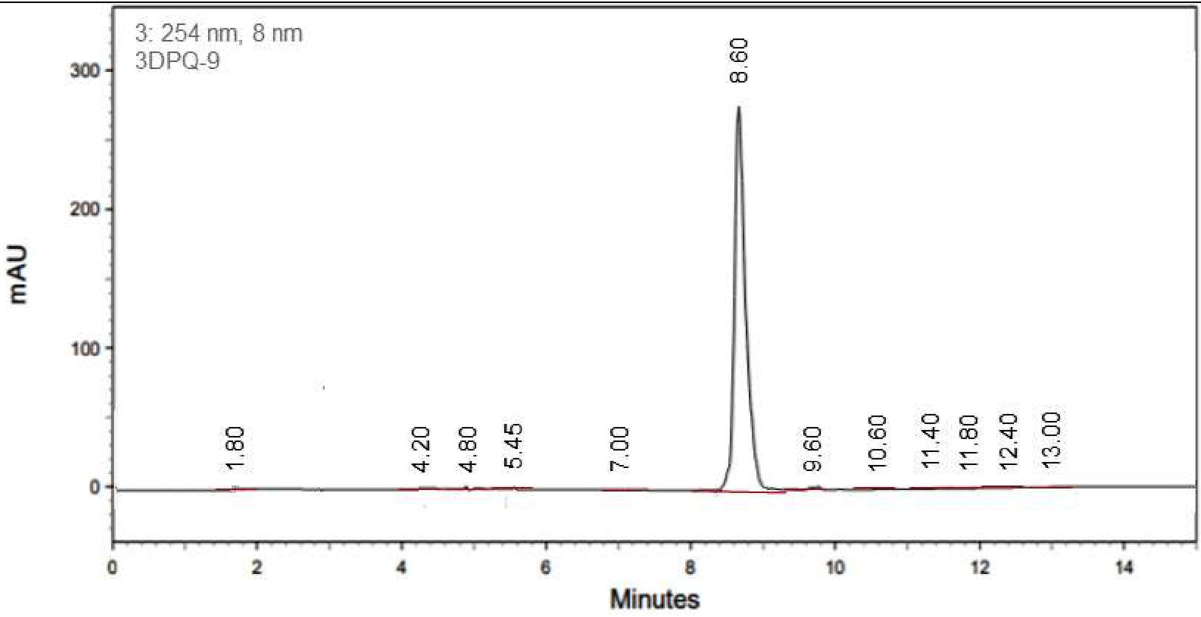

254 nm

Retention time	*Height*	*Area*	*Area Percent*
1.80	1795	5596	0.13
4.20	1748	5541	0.17
4.85	1562	5286	0.11
5.45	1418	4295	0.15
7.00	1326	4419	0.16
8.60	496515	1356215	98.58
9.60	1831	4184	0.12
10.60	1558	5639	0.13
11.15	1618	5152	0.11
11.80	1495	5185	0.12
12.40	1495	5632	0.13
13.00	1501	5123	0.09

Figure S187. The HPLC spectrum of **3DPQ-9.**

HPLC REPORT	
Compound ID	: 3DPQ-10
Filename/Sample ID	: D:\HPLC\2021\September\3DPQ-10
Method Name	: D:\method\2021\MILAN-3dpq.met
Instrument and Column	: Agilent 1260 Infinity System, Agilent Zorbax AB-C18
Run Time	: 9/20/2021/12:22:17

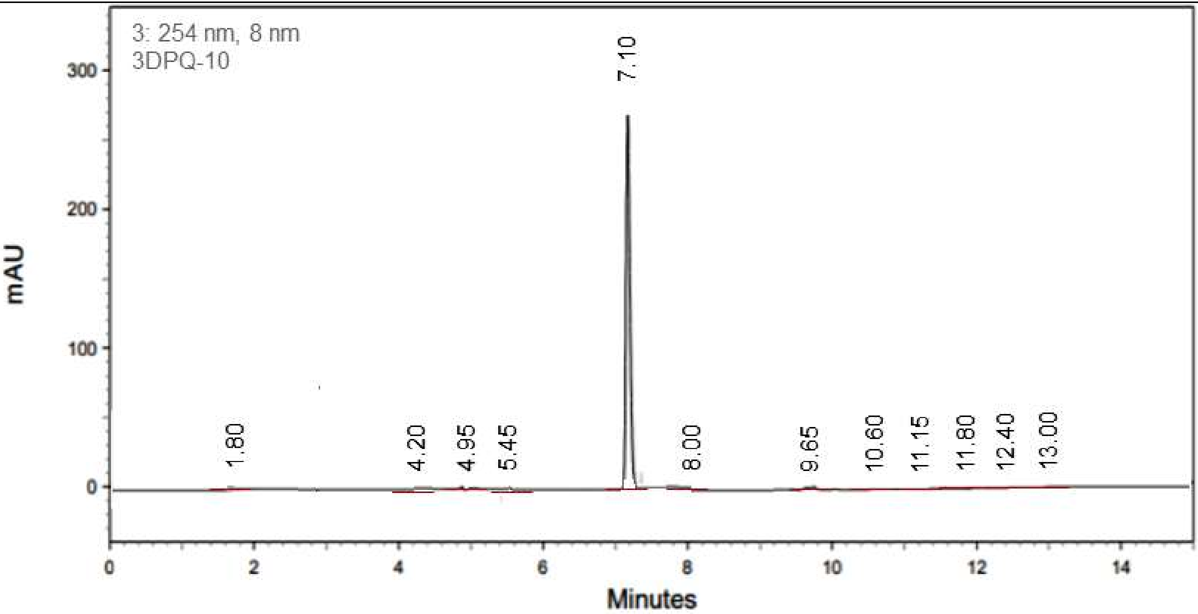

254 nm

Retention time	*Height*	*Area*	*Area Percent*
1.80	1784	5562	0.13
4.20	1736	5541	0.12
4.95	1545	5225	0.15
5.45	1458	4277	0.13
7.10	1342	4461	0.18
8.00	496515	1356215	98.51
9.65	1818	4141	0.13
10.60	1515	5651	0.12
11.15	1685	5145	0.15
11.80	1452	5112	0.13
12.40	1441	5668	0.16
13.00	1352	5326	0.09

Figure S188. The HPLC spectrum of **3DPQ-10.**

HPLC REPORT	
Compound ID	: 3DPQ-11
Filename/Sample ID	: D:\HPLC\2021\September\3DPQ-11
Method Name	: D:\method\2021\MILAN-3dpq.met
Instrument and Column	: Agilent 1260 Infinity System, Agilent Zorbax AB-C18
Run Time	: 9/20/2021/12:55:34

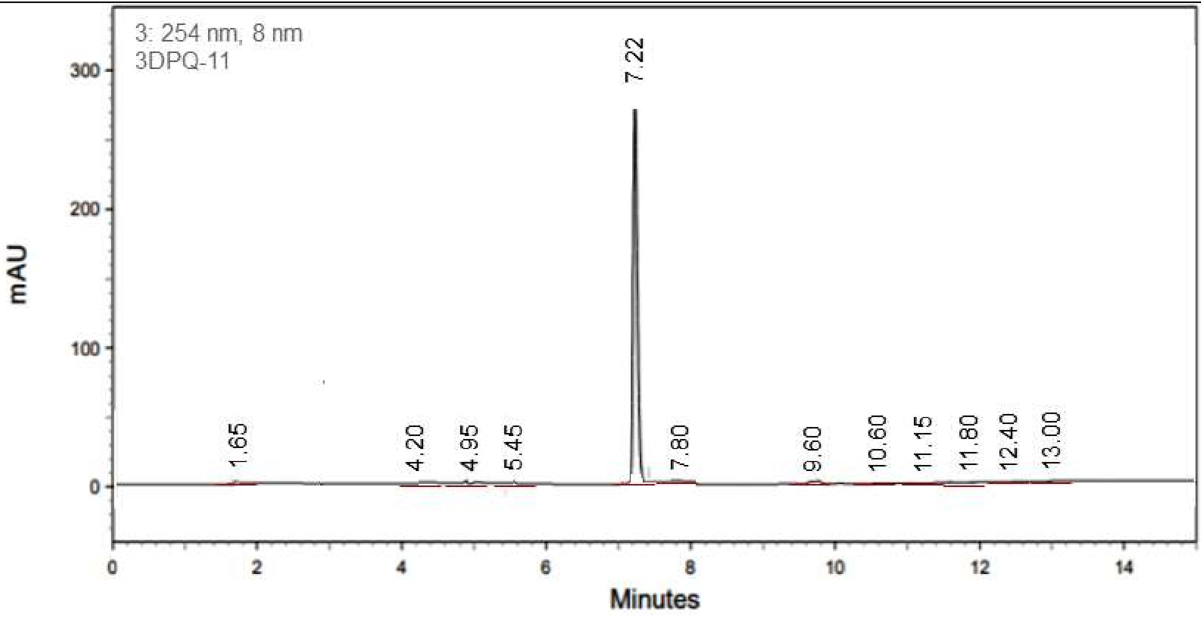

254 nm

Retention time	*Height*	*Area*	*Area Percent*
1.65	1715	5574	0.12
4.20	1755	5536	0.11
4.95	1565	5251	0.17
5.45	1445	4251	0.17
7.22	1318	4445	0.15
7.80	486523	1365284	98.42
9.65	1845	4261	0.17
10.60	1451	5421	0.15
11.15	1592	5536	0.19
11.80	1428	5251	0.14
12.40	1482	5562	0.11
13.00	1523	5856	0.10

Figure S189. The HPLC spectrum of **3DPQ-11**

HPLC REPORT

Compound ID	: 3DPQ-12
Filename/Sample ID	: D:\HPLC\2021\September\3DPQ-12
Method Name	: D:\method\2021\MILAN-3dpq.met
Instrument and Column	: Agilent 1260 Infinity System, Agilent Zorbax AB-C18
Run Time	: 9/20/2021/13:30:19

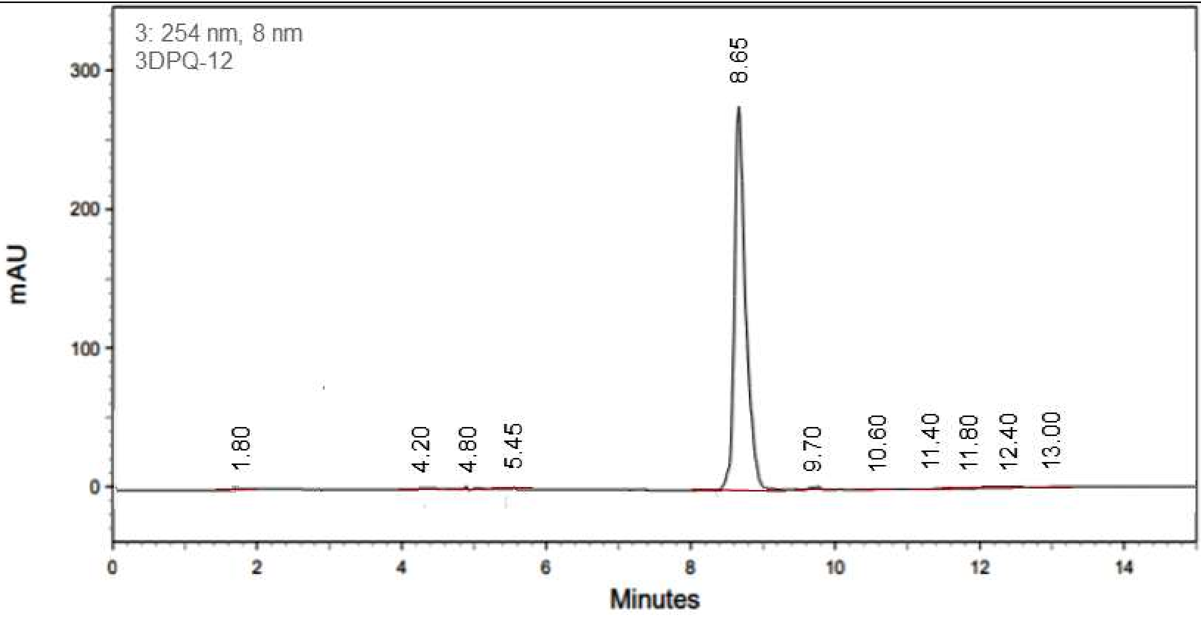

254 nm

Retention time	*Height*	*Area*	*Area Percent*
1.80	1694	5533	0.14
4.20	1732	5515	0.13
4.80	1517	5225	0.12
5.45	1502	4235	0.17
8.65	496521	1435265	98.69
9.70	1569	4543	0.15
10.60	1451	5321	0.19
11.40	1544	556	0.16
11.80	1484	5443	0.16
12.40	1416	5673	0.12
13.00	1556	5421	0.13

Figure S190. The HPLC spectrum of **3DPQ-12.**

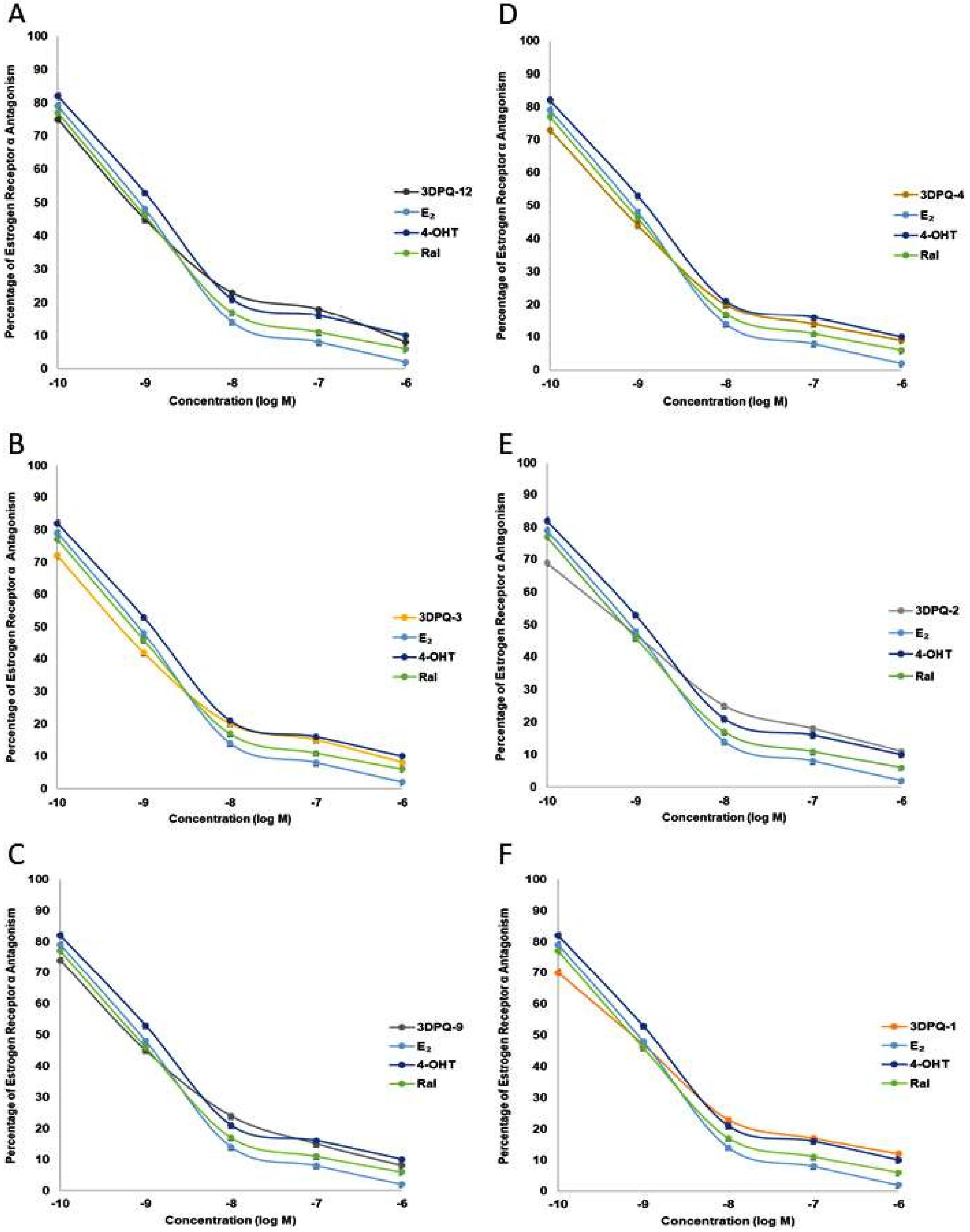

Figure S191. The estrogen receptor α antagonism in the presence of **3DPQ-12** (**A**); **3DPQ-3** (**B**); **3DPQ-9** (**C**); **3DPQ-4** (**D**); **3DPQ-2** (**E**); **3DPQ-1** (**F**).

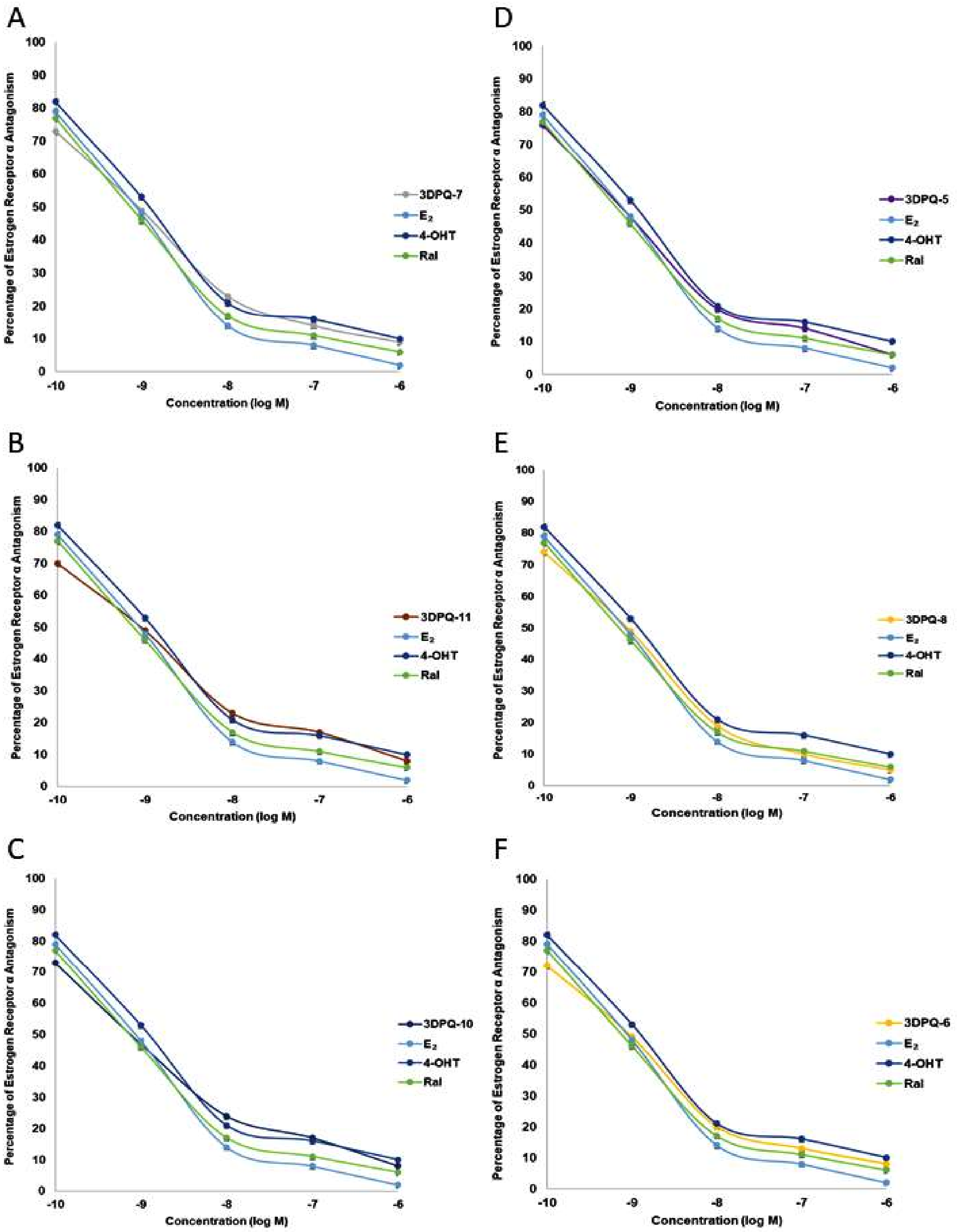

Figure S192. The estrogen receptor α antagonism in the presence of **3DPQ-7** (A); **3DPQ-11** (B); **3DPQ-10** (C); **3DPQ-5** (D); **3DPQ-8** (E); **3DPQ-6** (F).

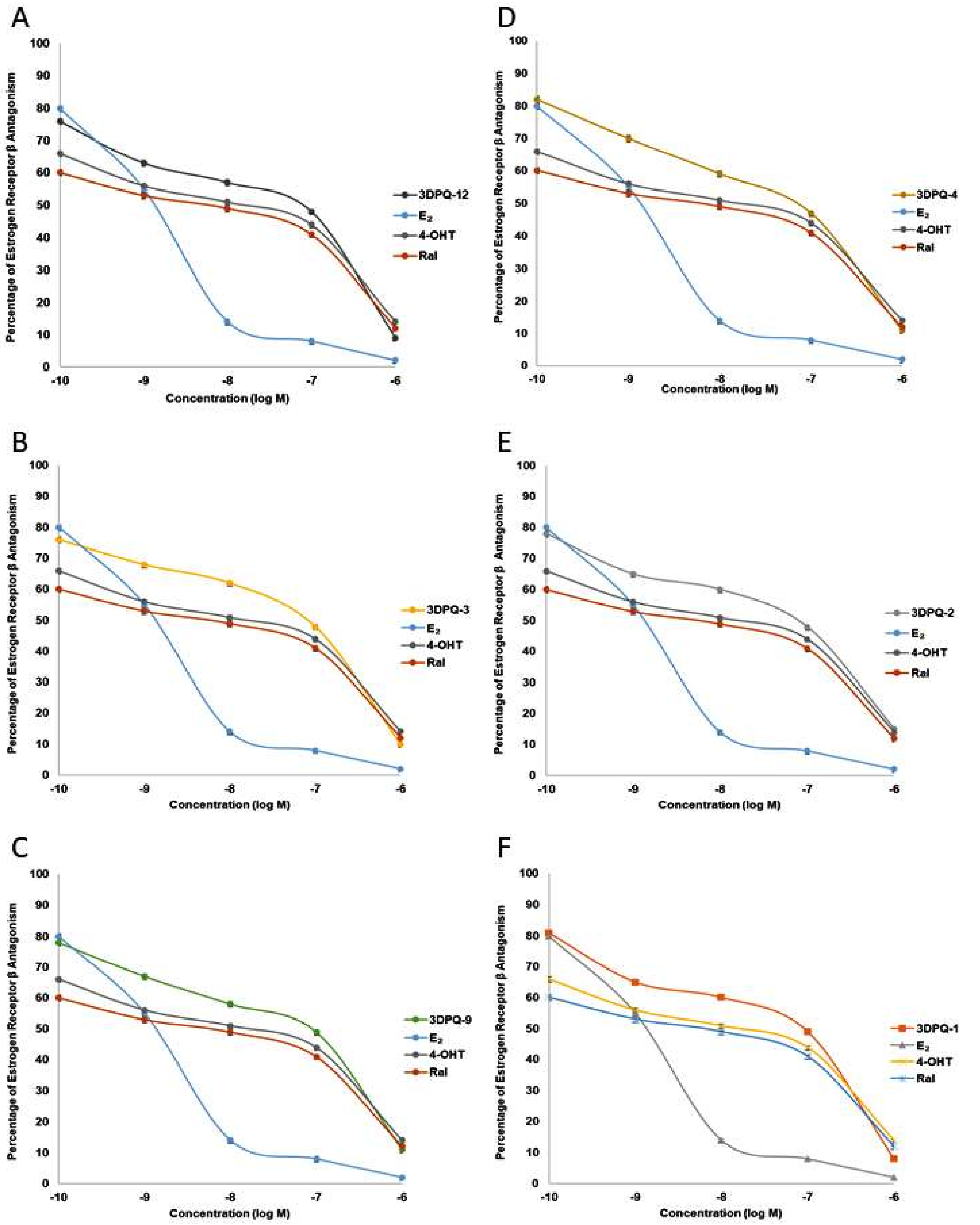

Figure S193. The estrogen receptor β antagonism in the presence of **3DPQ-12** (A); **3DPQ-3** (B); **3DPQ-9** (C); **3DPQ-4** (D); **3DPQ-2** (E); **3DPQ-1** (F).

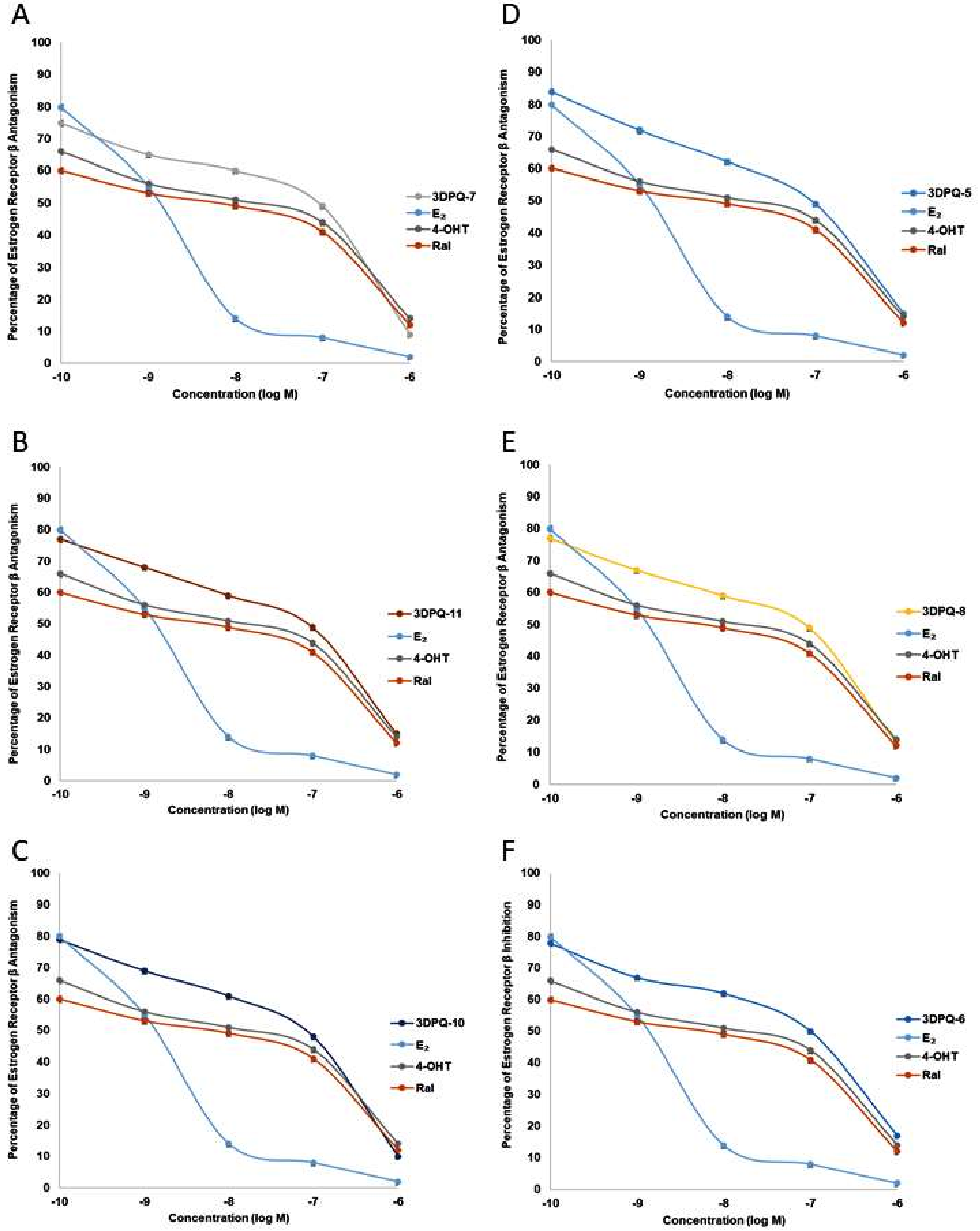

Figure S194. The estrogen receptor β antagonism in the presence of **3DPQ-7** (A); **3DPQ-11** (B); **3DPQ-10** (C); **3DPQ-5** (D); **3DPQ-8** (E); **3DPQ-6** (F).

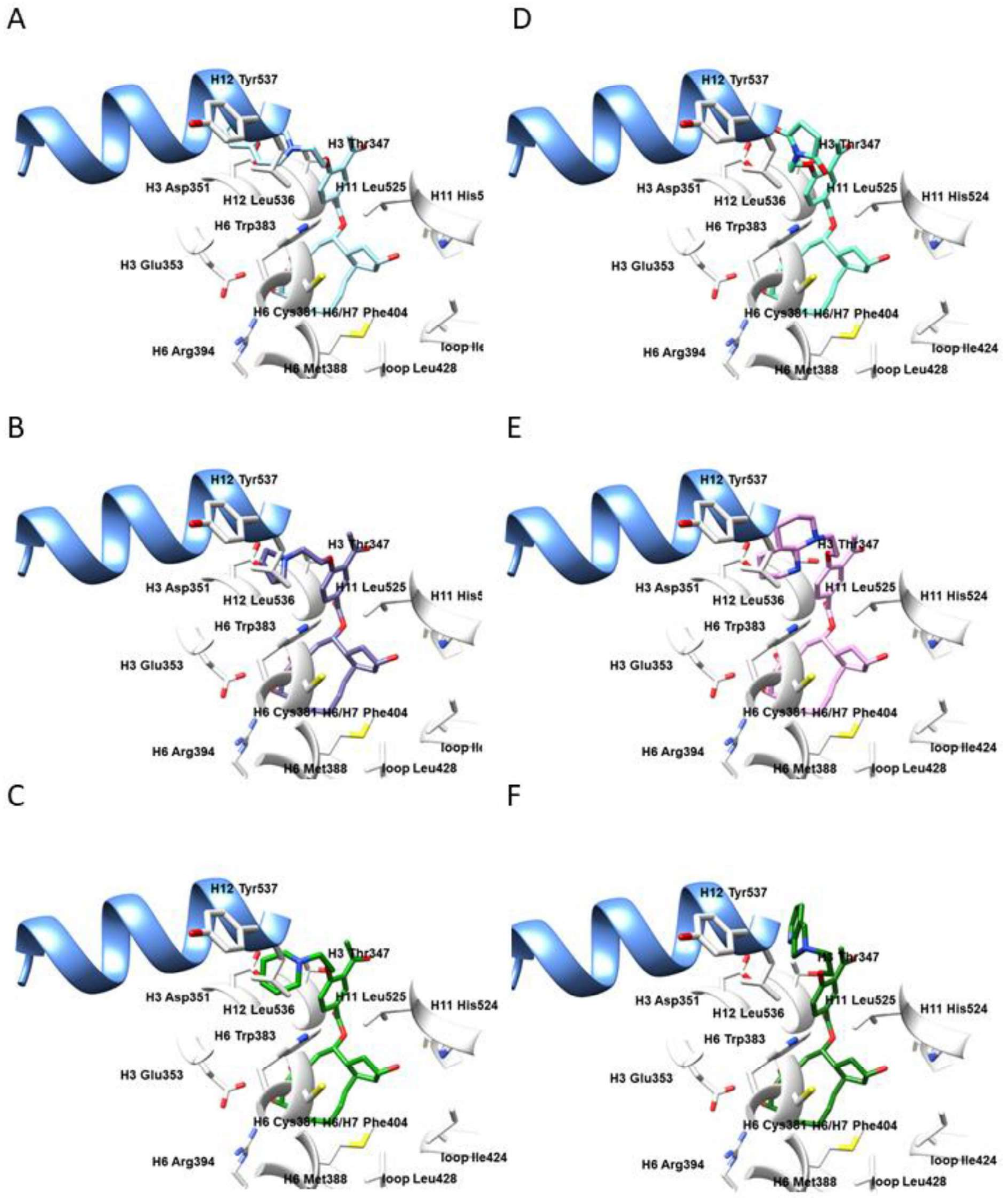

Figure S195. The bioactive conformations of **3DPQ-7** (A); **3DPQ-11** (B); **3DPQ-10** (C); **3DPQ-5** (D); **3DPQ-8** (E); **3DPQ-6** (F) within the ER*α* active site. Amino acid residues are depicted in white, H12 helix is presented in cornflower blue ribbon.

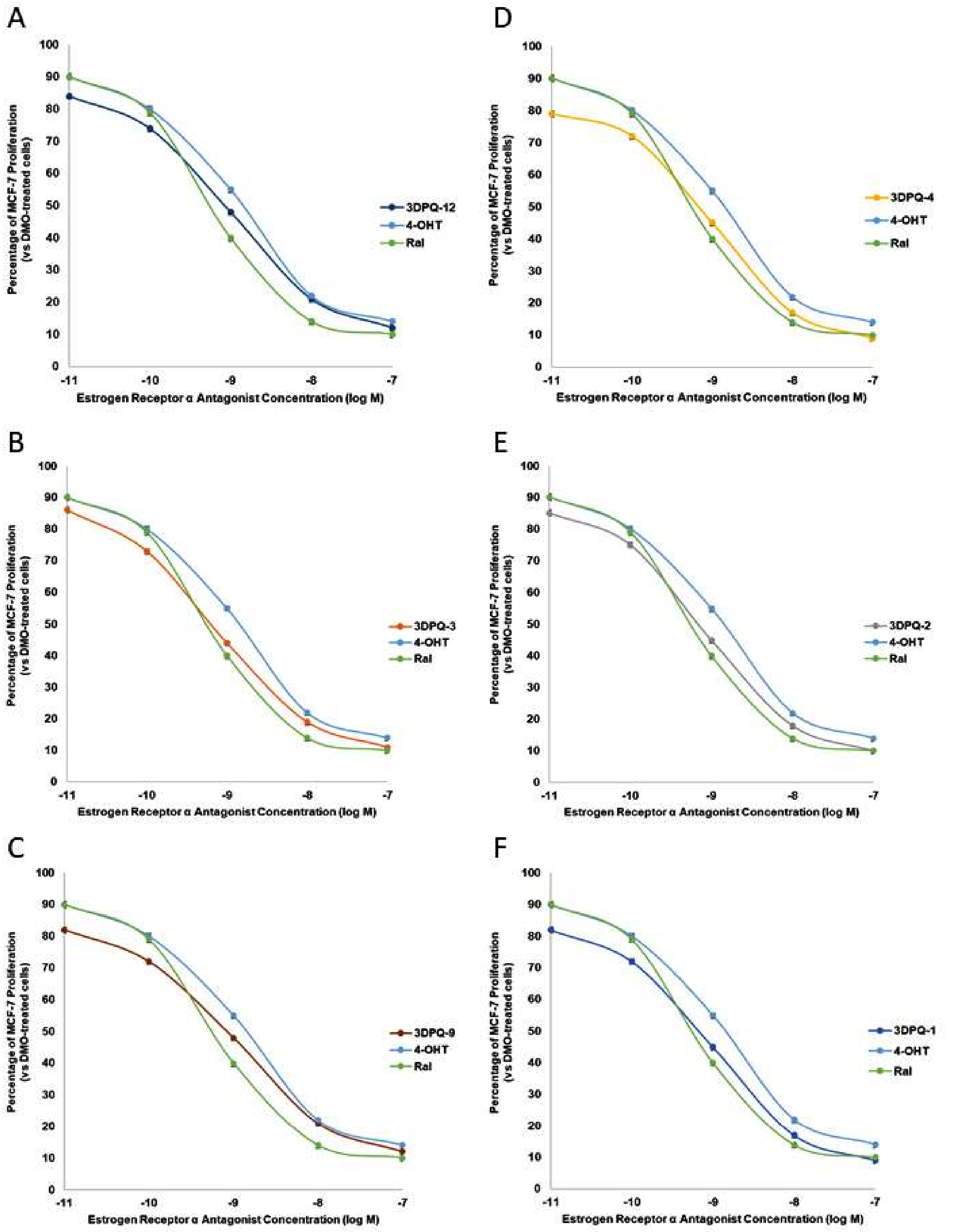

Figure S196. The antiproliferative activity of **3DPQ-12** (**A**); **3DPQ-3** (**B**); **3DPQ-9** (**C**); **3DPQ-4** (**D**); **3DPQ-2** (**E**); **3DPQ-1** (**F**) against MCF-7 cell line.

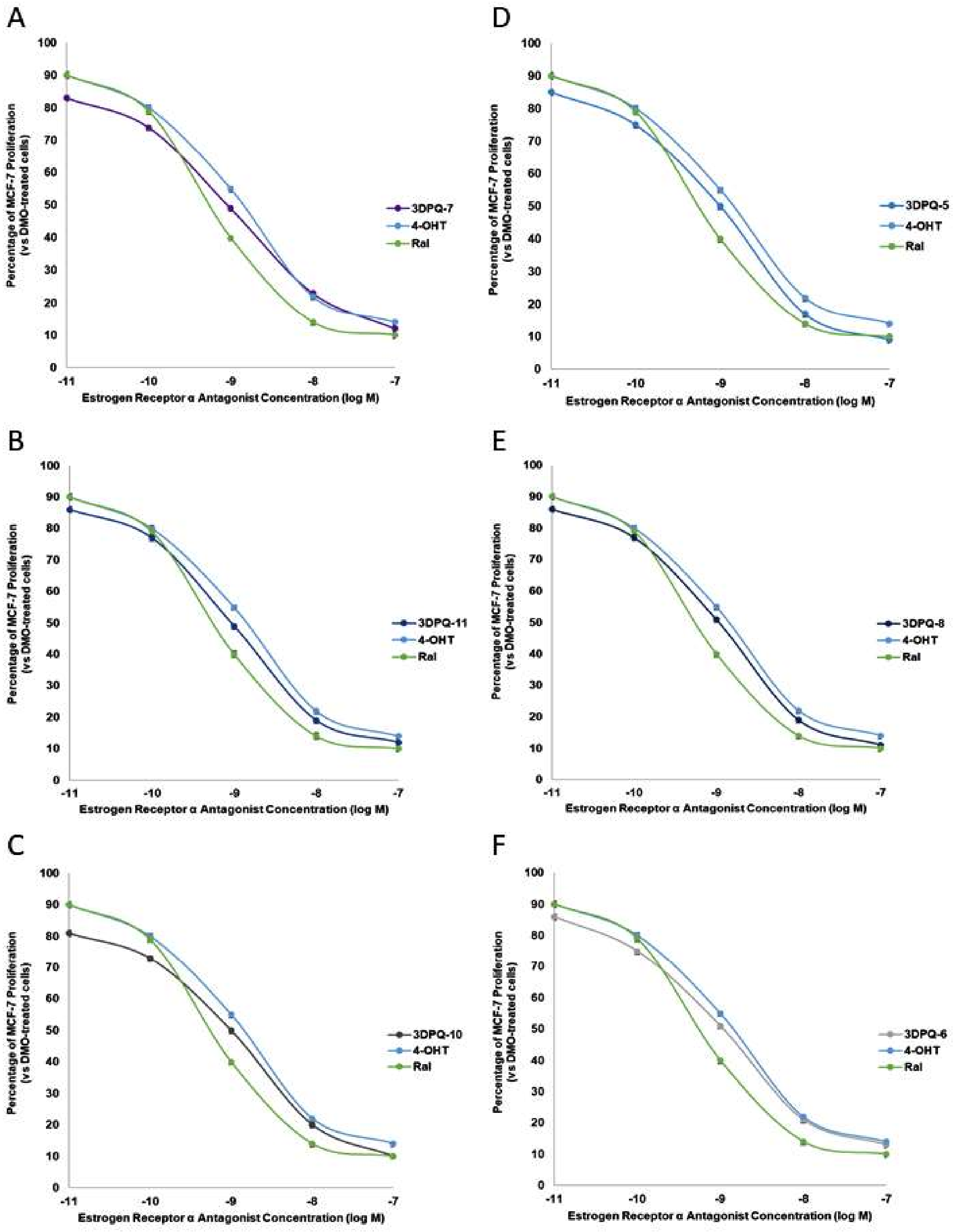

Figure S197. The antiproliferative activity of **3DPQ-7** (A); **3DPQ-11** (B); **3DPQ-10** (C); **3DPQ-5** (D); **3DPQ-8** (E); **3DPQ-6** (F) against MCF-7 cell lines.

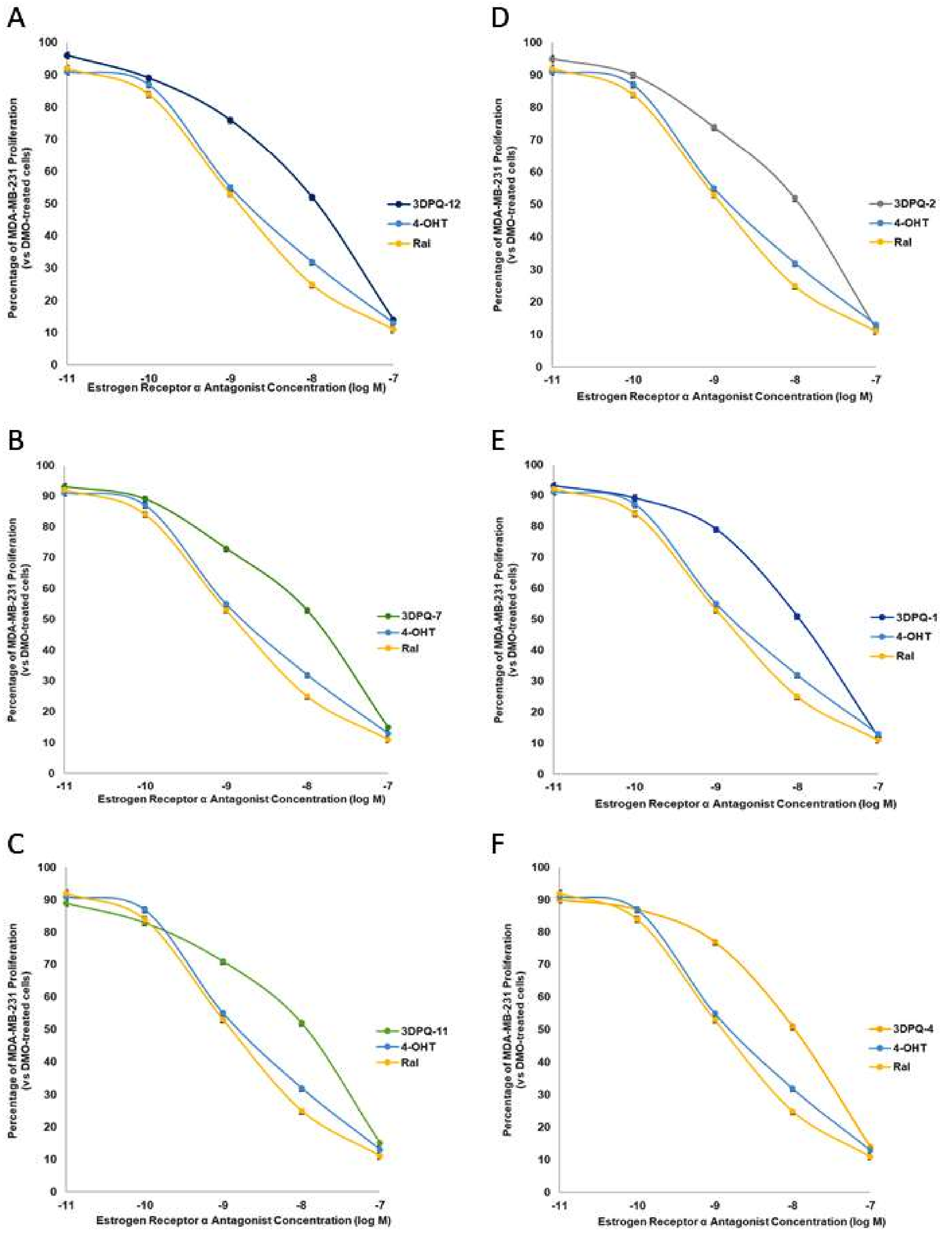

Figure S198. The antiproliferative activity of **3DPQ-12** (A); **3DPQ-3** (B); **3DPQ-9** (C); **3DPQ-4** (D); **3DPQ-2** (E); **3DPQ-1** (F) against MDA-MB-231 cell lines.

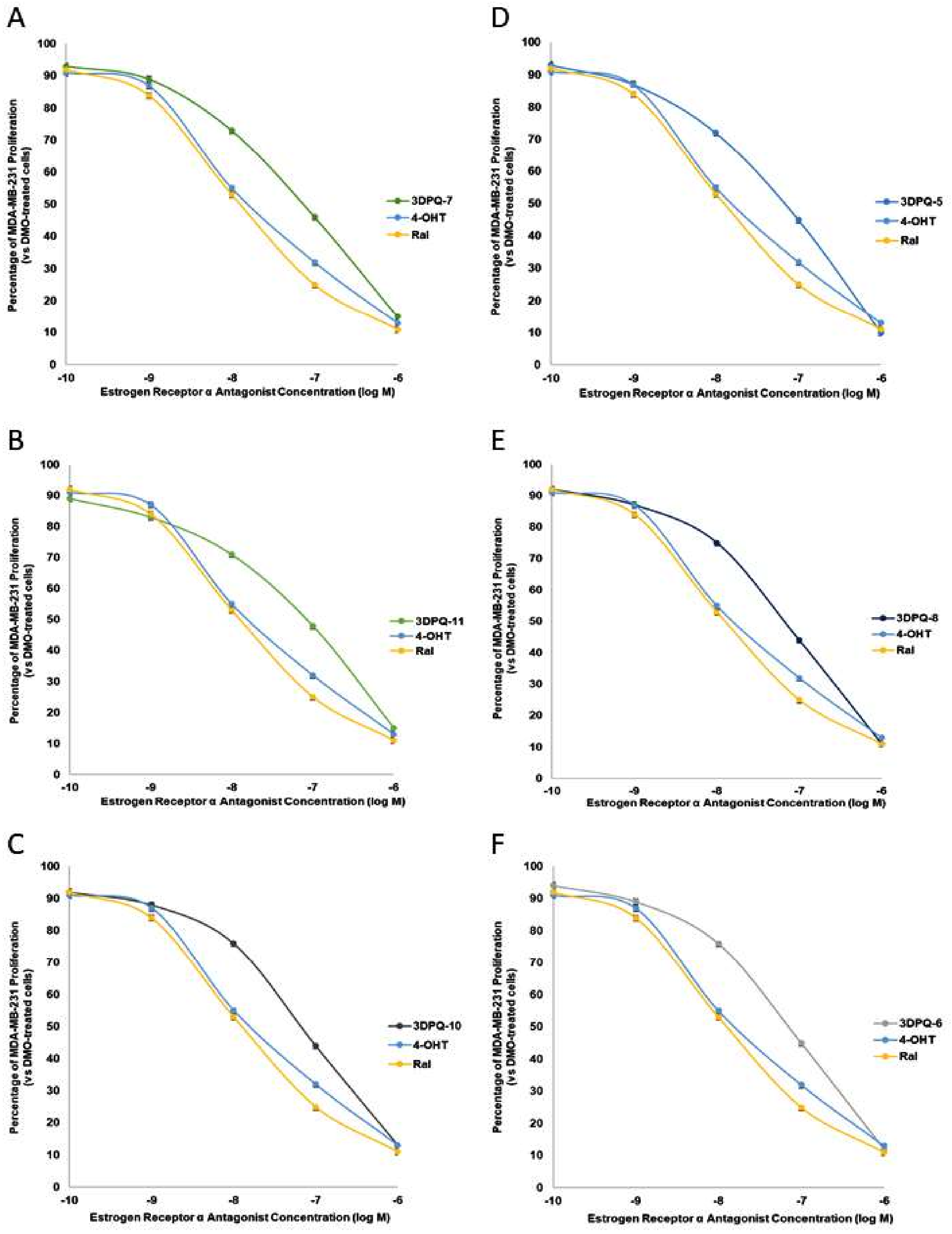

Figure S199. The antiproliferative activity of **3DPQ-7** (A); **3DPQ-11** (B); **3DPQ-10** (C); **3DPQ-5** (D); **3DPQ-8** (E); **3DPQ-6** (F) against MDA-MB-231 cell lines.

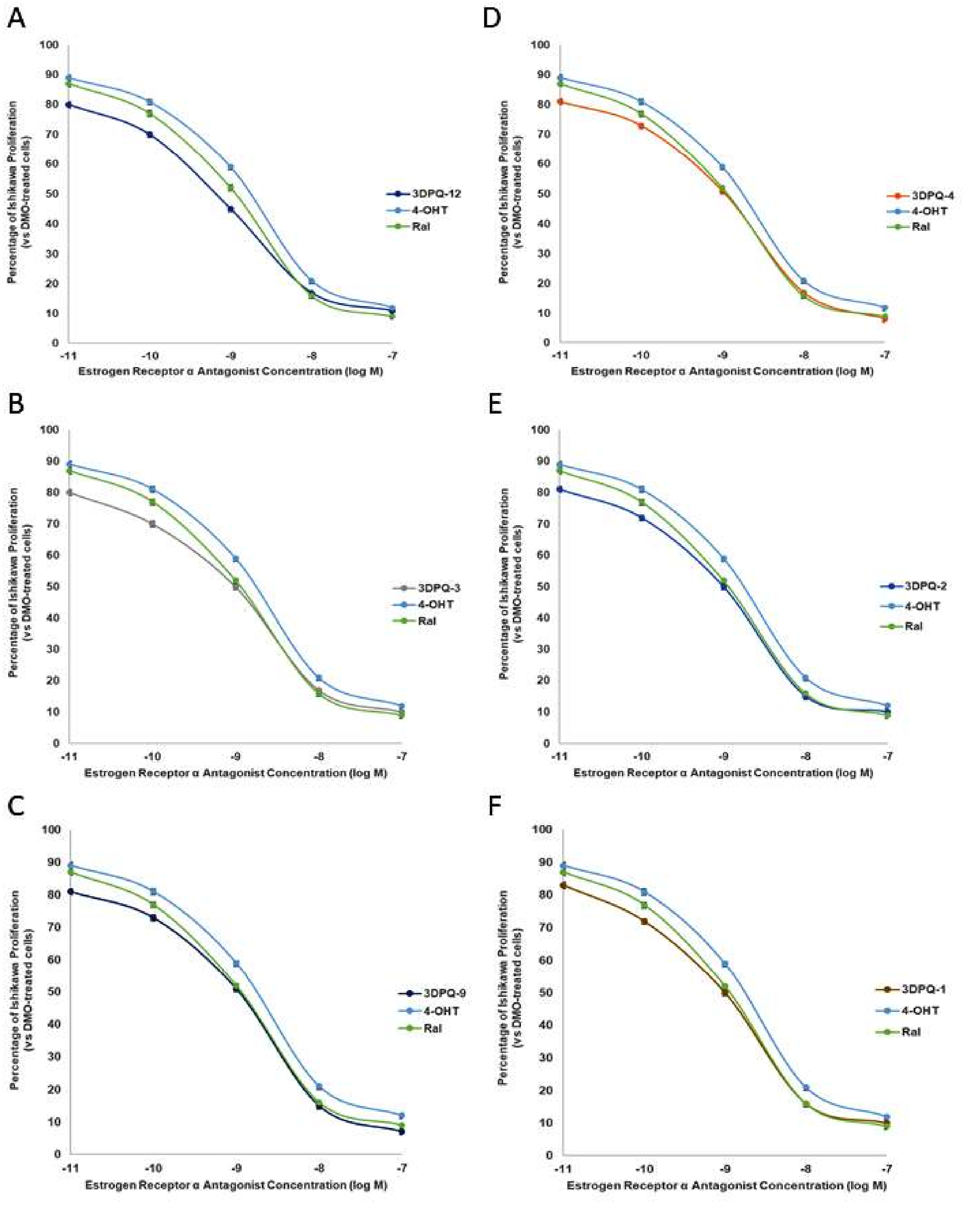

Figure S200. The antiproliferative activity of **3DPQ-12** (A); **3DPQ-3** (B); **3DPQ-9** (C); **3DPQ-4** (D); **3DPQ-2** (E); **3DPQ-1** (F) against Ishikawa cell lines.

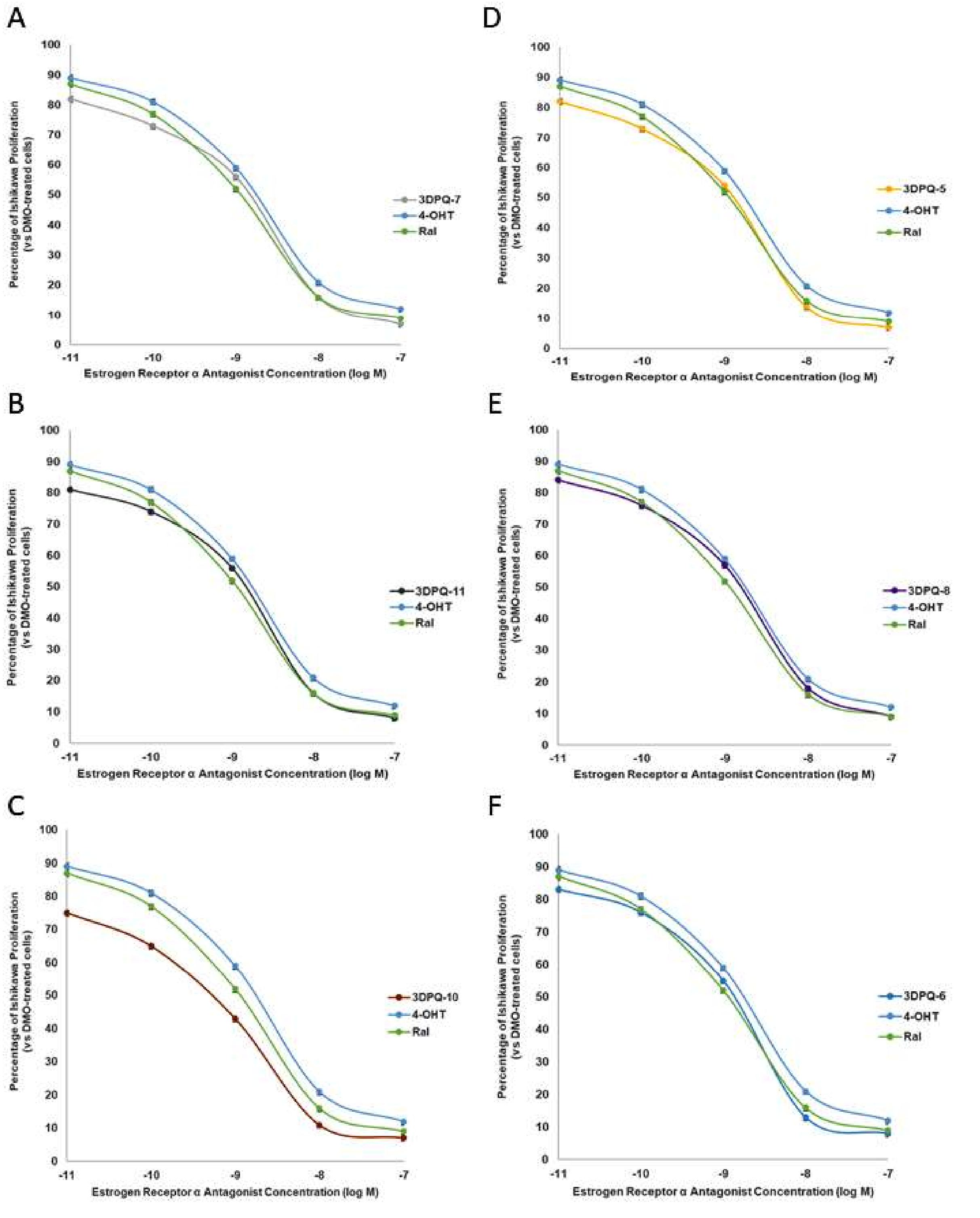

Figure S201. The antiproliferative activity of **3DPQ-7** (A); **3DPQ-11** (B); **3DPQ-10** (C); **3DPQ-5** (D); **3DPQ-8** (E); **3DPQ-6** (F) against Ishikawa cell lines.

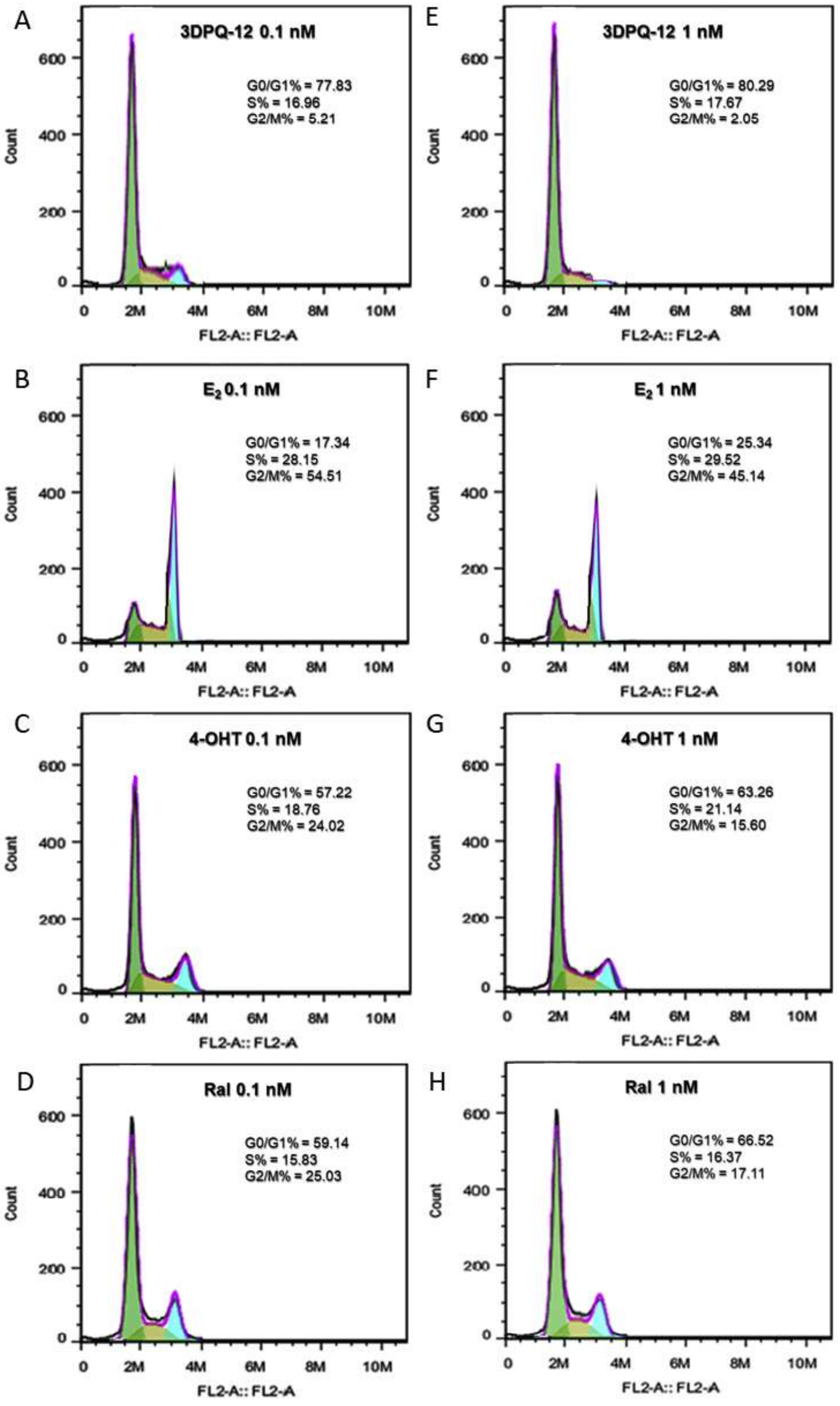

Figure S202. Cell cycle analysis of MCF-7 cell lines treated with **3DPQ-12** in concentration of 0.1 nM (A); **E**$_2$ in concentration of 0.1 nM (B); **4-OHT** in concentration of 0.1 nM (C); **Ral** in concentration of 0.1 nM (D); **3DPQ-12** in concentration of 1 nM (E); **E**$_2$ in concentration of 1 nM (F); **4-OHT** in concentration of 1 nM (G); **Ral** in concentration of 1 nM.

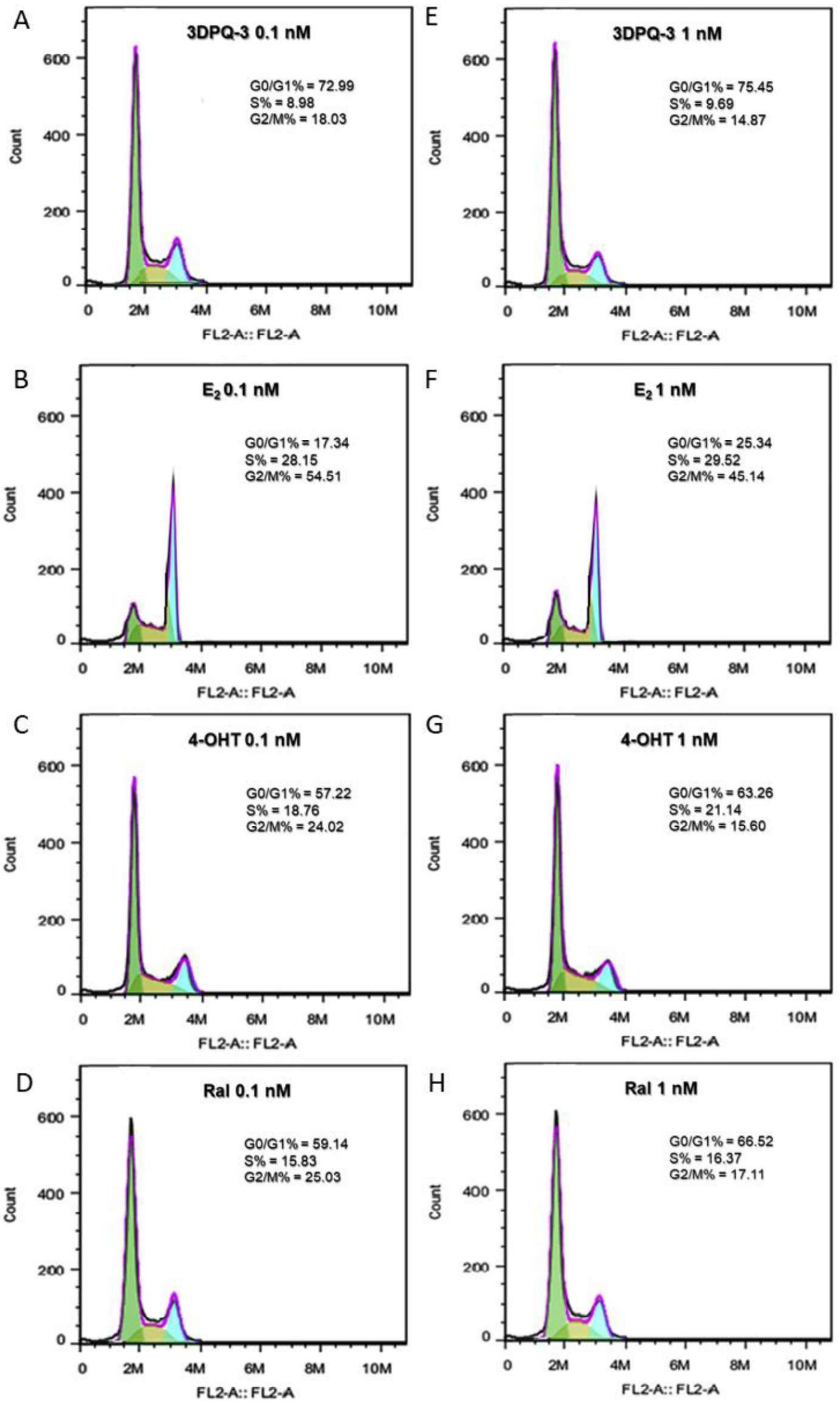

Figure S203. Cell cycle analysis of MCF-T cell lines treated with **3DPQ-3** in concentration of 0.1 nM (A); **E_2** in concentration of 0.1 nM (B); **4-OHT** in concentration of 0.1 nM (C); **Ral** in concentration of 0.1 nM (D); **3DPQ-3** in concentration of 1 nM (E); **E_2** in concentration of 1 nM (F); **4-OHT** in concentration of 1 nM (G); **Ral** in concentration of 1 nM.

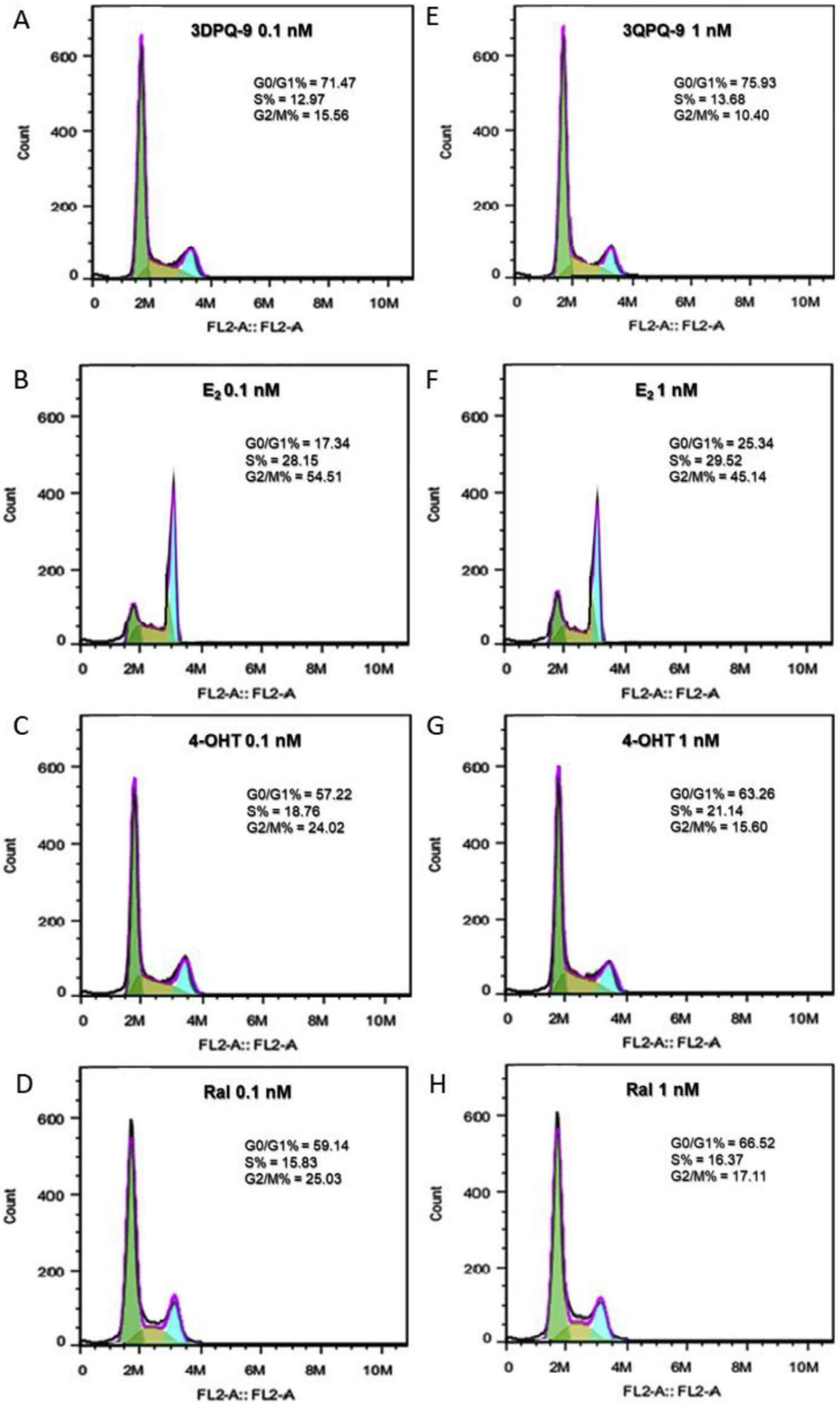

Figure S204. Cell cycle analysis of MCF-T cell lines treated with **3DPQ-9** in concentration of 0.1 nM (A); **E**$_2$ in concentration of 0.1 nM (B); **4-OHT** in concentration of 0.1 nM (C); **Ral** in concentration of 0.1 nM (D); **3DPQ-9** in concentration of 1 nM (E); **E**$_2$ in concentration of 1 nM (F); **4-OHT** in concentration of 1 nM (G); **Ral** in concentration of 1 nM.

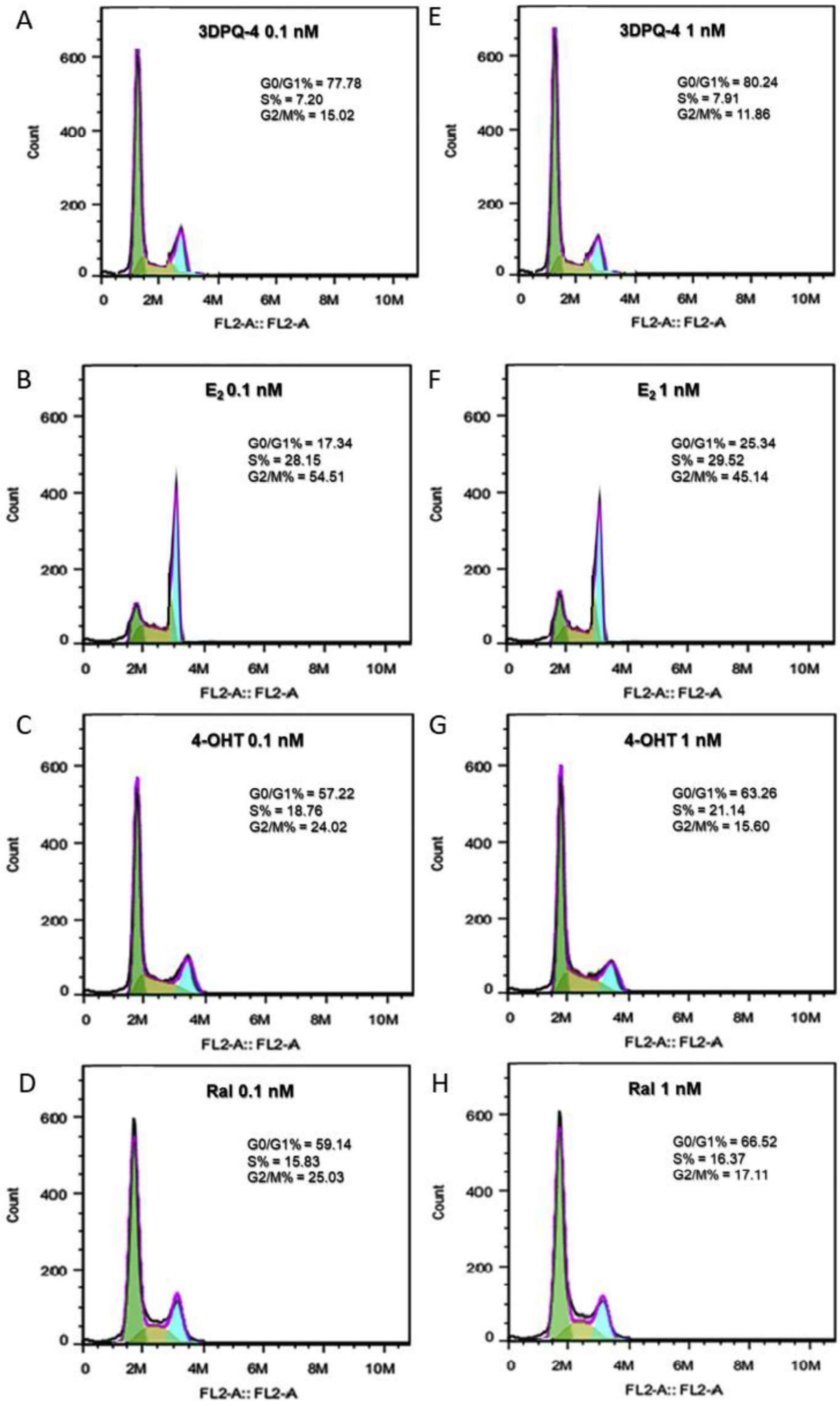

Figure S205. Cell cycle analysis of MCF-T cell lines treated with **3DPQ-4** in concentration of 0.1 nM (A); **E_2** in concentration of 0.1 nM (B); **4-OHT** in concentration of 0.1 nM (C); **Ral** in concentration of 0.1 nM (D); **3DPQ-4** in concentration of 1 nM (E); **E_2** in concentration of 1 nM (F); **4-OHT** in concentration of 1 nM (G); **Ral** in concentration of 1 nM.

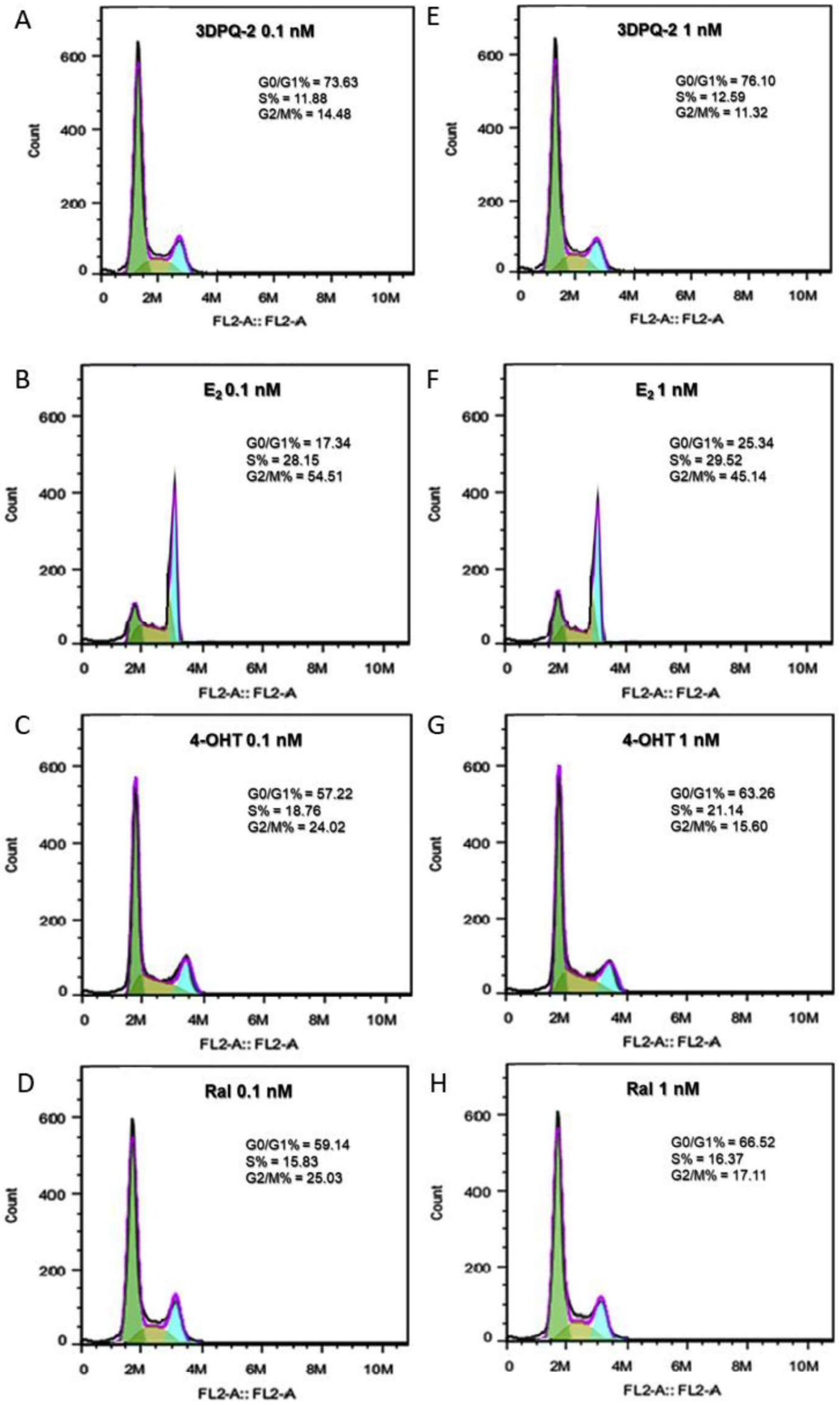

Figure S206. Cell cycle analysis of MCF-T cell lines treated with **3DPQ-2** in concentration of 0.1 nM (A); **E**$_2$ in concentration of 0.1 nM (B); **4-OHT** in concentration of 0.1 nM (C); **Ral** in concentration of 0.1 nM (D); **3DPQ-2** in concentration of 1 nM (E); **E**$_2$ in concentration of 1 nM (F); **4-OHT** in concentration of 1 nM (G); **Ral** in concentration of 1 nM.

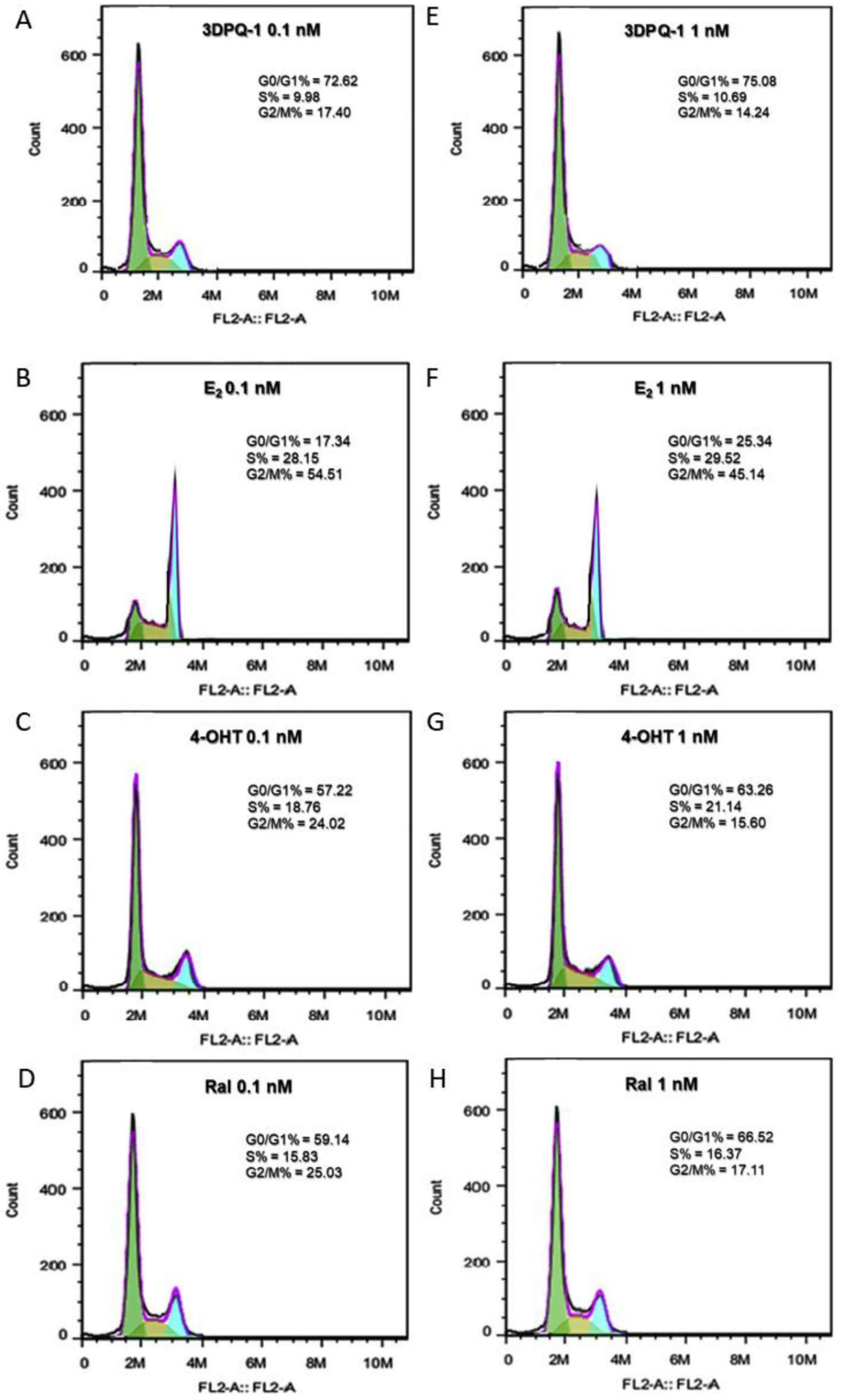

Figure S207. Cell cycle analysis of MCF-T cell lines treated with **3DPQ-1** in concentration of 0.1 nM (A); **E**$_2$ in concentration of 0.1 nM (B); **4-OHT** in concentration of 0.1 nM (C); **Ral** in concentration of 0.1 nM (D); **3DPQ-1** in concentration of 1 nM (E); **E**$_2$ in concentration of 1 nM (F); **4-OHT** in concentration of 1 nM (G); **Ral** in concentration of 1 nM.

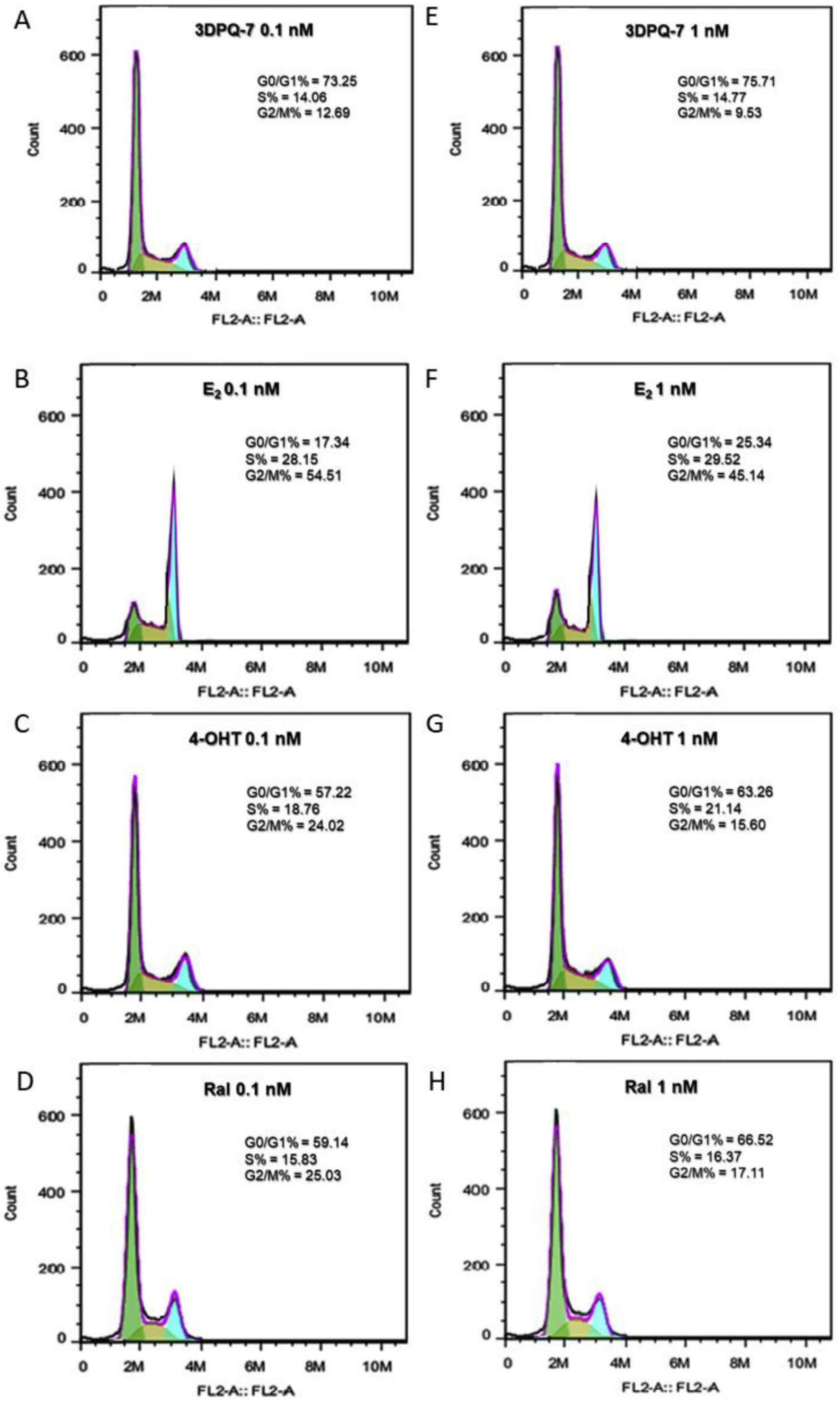

Figure S208. Cell cycle analysis of MCF-T cell lines treated with **3DPQ-7** in concentration of 0.1 nM (A); **E_2** in concentration of 0.1 nM (B); **4-OHT** in concentration of 0.1 nM (C); **Ral** in concentration of 0.1 nM (D); **3DPQ-7** in concentration of 1 nM (E); **E_2** in concentration of 1 nM (F); **4-OHT** in concentration of 1 nM (G); **Ral** in concentration of 1 nM.

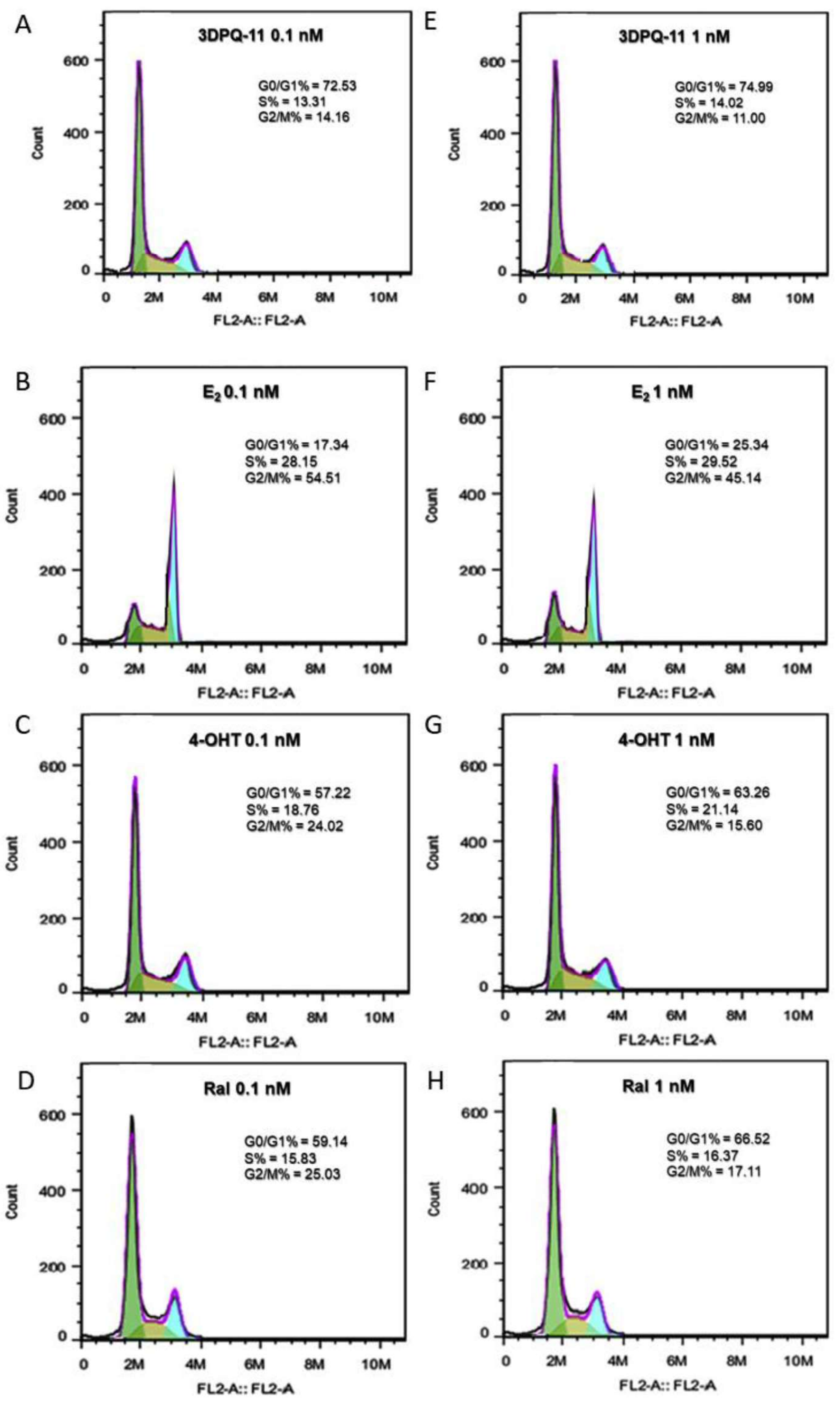

Figure S209. Cell cycle analysis of MCF-T cell lines treated with **3DPQ-11** in concentration of 0.1 nM (A); **E**$_2$ in concentration of 0.1 nM (B); **4-OHT** in concentration of 0.1 nM (C); **Ral** in concentration of 0.1 nM (D); **3DPQ-11** in concentration of 1 nM (E); **E**$_2$ in concentration of 1 nM (F); **4-OHT** in concentration of 1 nM (G); **Ral** in concentration of 1 nM.

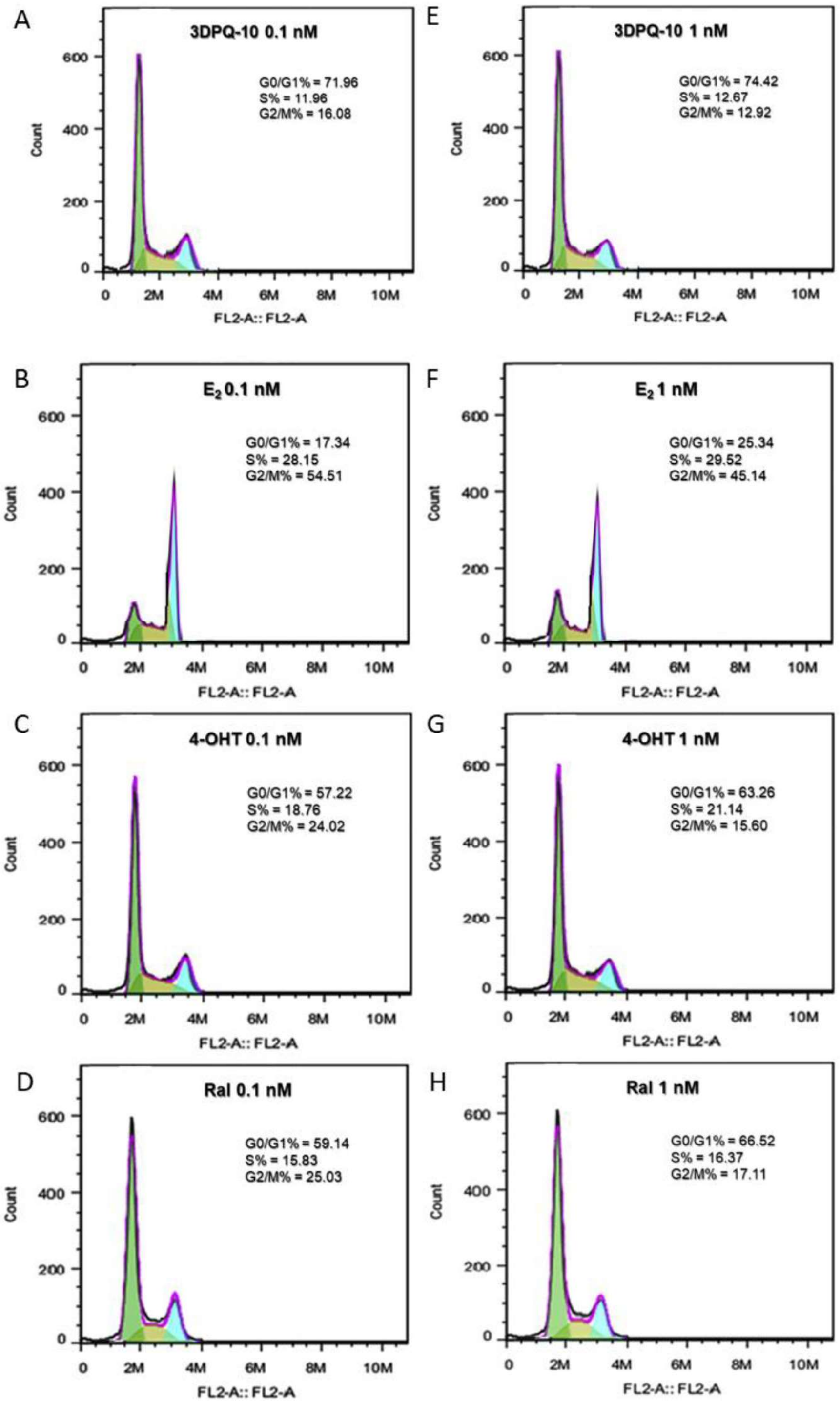

Figure S210. Cell cycle analysis of MCF-T cell lines treated with **3DPQ-10** in concentration of 0.1 nM (A); **E2** in concentration of 0.1 nM (B); **4-OHT** in concentration of 0.1 nM (C); **Ral** in concentration of 0.1 nM (D); **3DPQ-10** in concentration of 1 nM (E); **E2** in concentration of 1 nM (F); **4-OHT** in concentration of 1 nM (G); **Ral** in concentration of 1 nM.

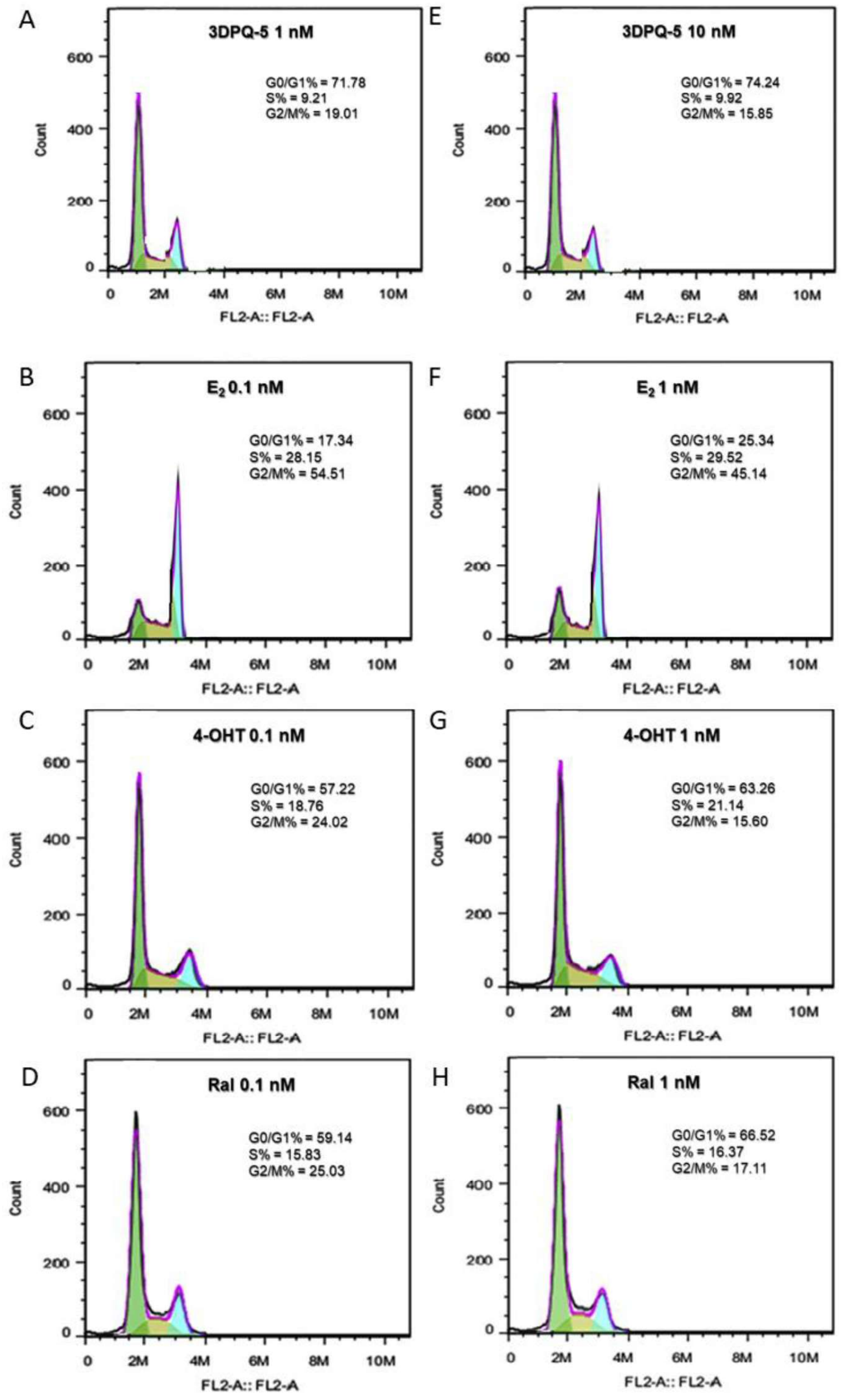

Figure S211. Cell cycle analysis of MCF-T cell lines treated with **3DPQ-5** in concentration of 1 nM (A); $\mathbf{E_2}$ in concentration of 0.1 nM (B); **4-OHT** in concentration of 0.1 nM (C); **Ral** in concentration of 0.1 nM (D); **3DPQ-5** in concentration of 10 nM (E); $\mathbf{E_2}$ in concentration of 1 nM (F); **4-OHT** in concentration of 1 nM (G); **Ral** in concentration of 1 nM.

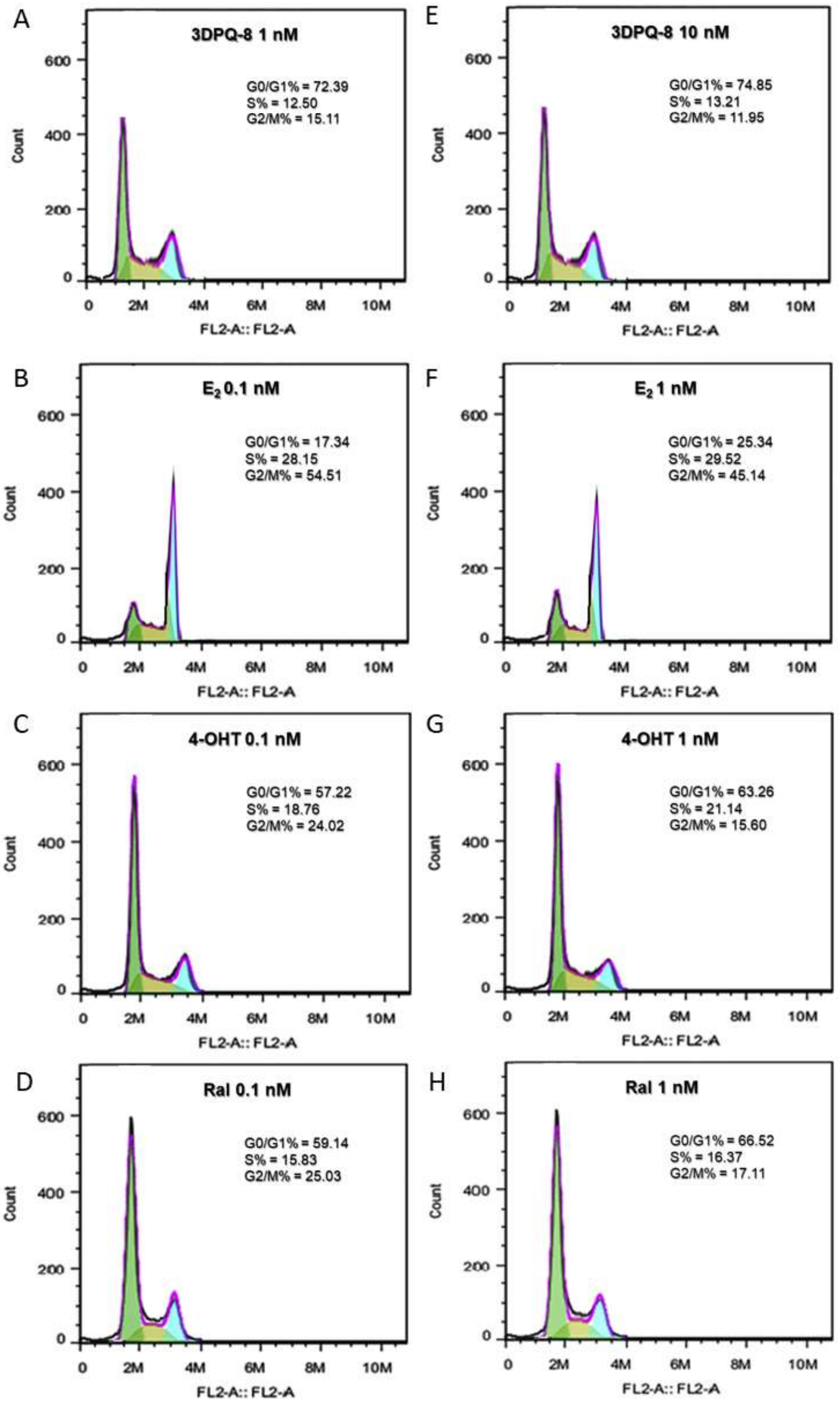

Figure S212. Cell cycle analysis of MCF-T cell lines treated with **3DPQ-8** in concentration of 1 nM (A); **E**$_2$ in concentration of 0.1 nM (B); **4-OHT** in concentration of 0.1 nM (C); **Ral** in concentration of 0.1 nM (D); **3DPQ-8** in concentration of 10 nM (E); **E**$_2$ in concentration of 1 nM (F); **4-OHT** in concentration of 1 nM (G); **Ral** in concentration of 1 nM.

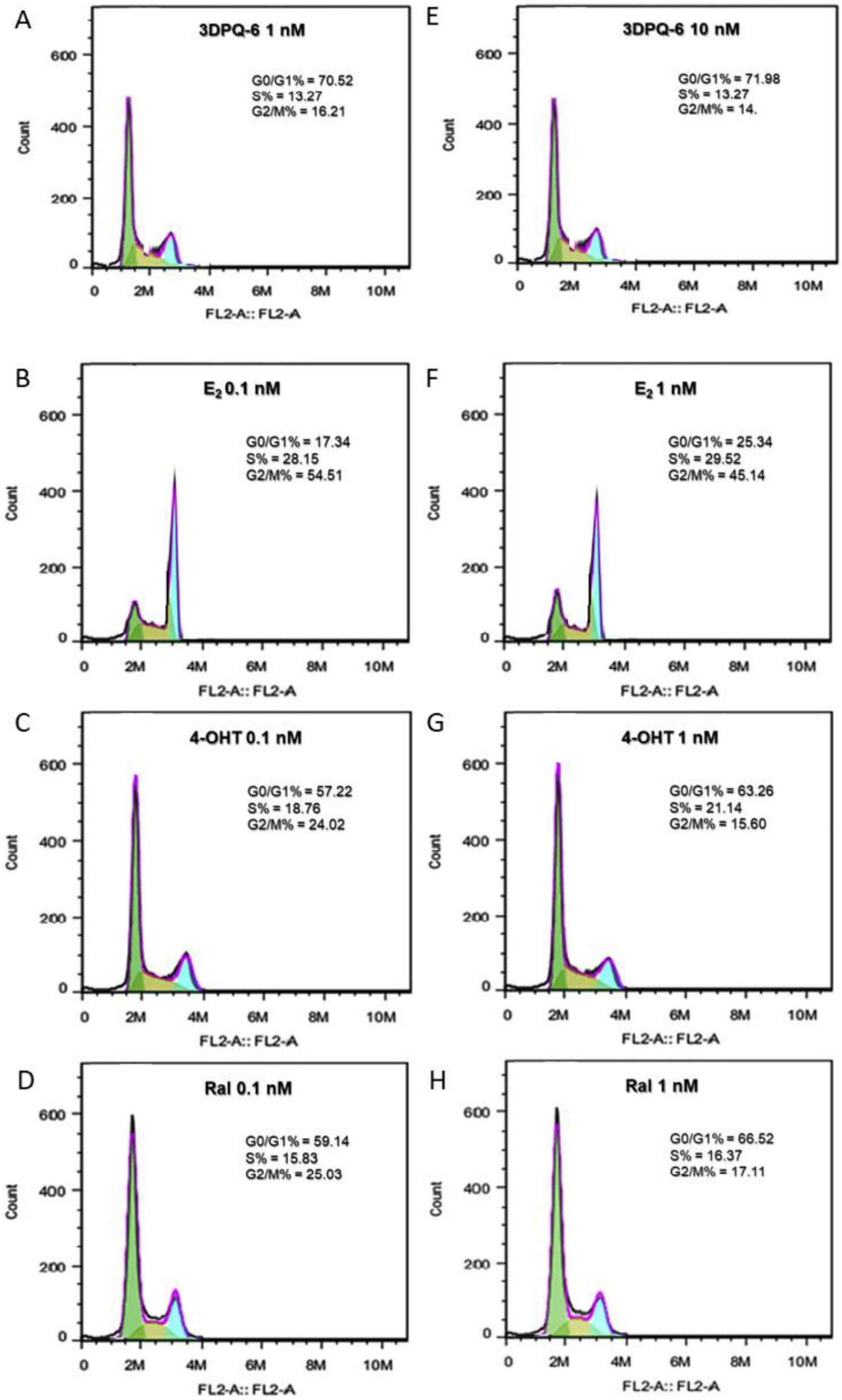

Figure S213. Cell cycle analysis of MCF-T cell lines treated with **3DPQ-6** in concentration of 1 nM (A); **E**$_2$ in concentration of 0.1 nM (B); **4-OHT** in concentration of 0.1 nM (C); **Ral** in concentration of 0.1 nM (D); **3DPQ-6** in concentration of 10 nM (E); **E**$_2$ in concentration of 1 nM (F); **4-OHT** in concentration of 1 nM (G); **Ral** in concentration of 1 nM.

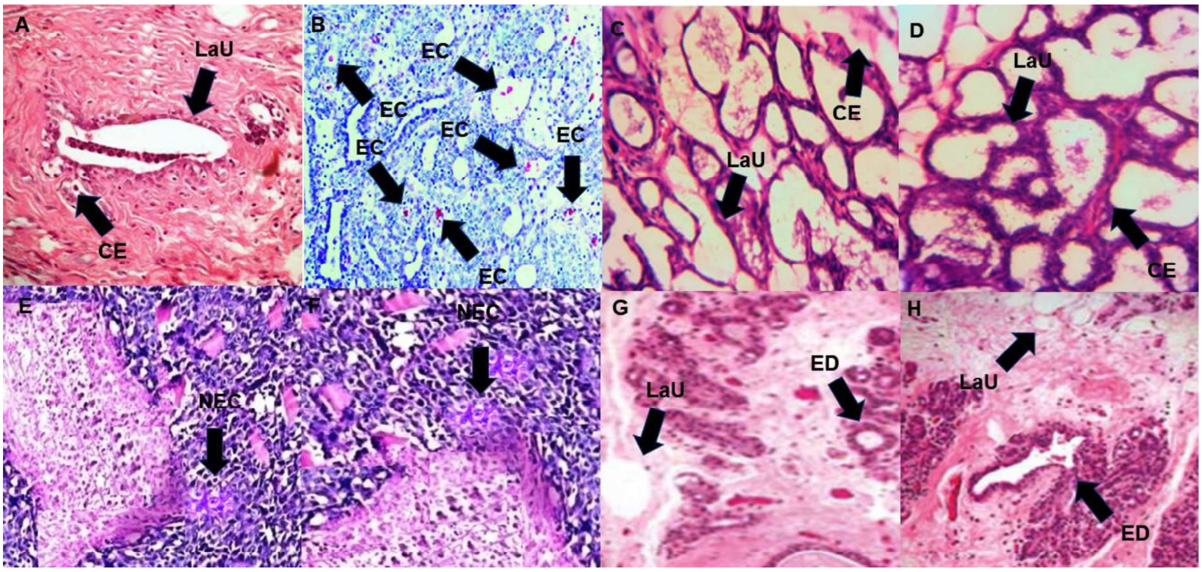

Figure S214. Photomicrograph of breast section of normal control rat showing lobuloalveolar unit (LaU) and cuboidal epithelial cells (CE) (A); photomicrograph of breast section treated with **MNU** showing mammary gland carcinoma alongside with massive proliferation of neoplastic epithelial cells (EC) (B); photomicrograph of breast section treated with **3DPQ-3** in concentration of 5 mg/kg of bwt showing lobuloalveolar unit (LaU) and cuboidal epithelial cells (CE) (C); photomicrograph of breast section treated with **3DPQ-3** in concentration of 50 mg/kg of bwt showing lobuloalveolar unit (LaU) and cuboidal epithelial cells (CE) (D); photomicrograph of breast section treated with **4-OHT** in concentration of 5 mg/kg of bwt showing necrosis (NEC) (E); photomicrograph of breast section treated with **4-OHT** in concentration of 50 mg/kg of bwt showing necrosis (NEC) (F); photomicrograph of breast section treated with **Ral** in concentration of 5 mg/kg of bwt showing differentiated extralobular ducts (ED) (G); photomicrograph of breast section treated with **Ral** in concentration of 50 mg/kg of bwt showing differentiated extralobular ducts (ED) (H), shown in x200 magnification and stained with hematoxylin and eosin.

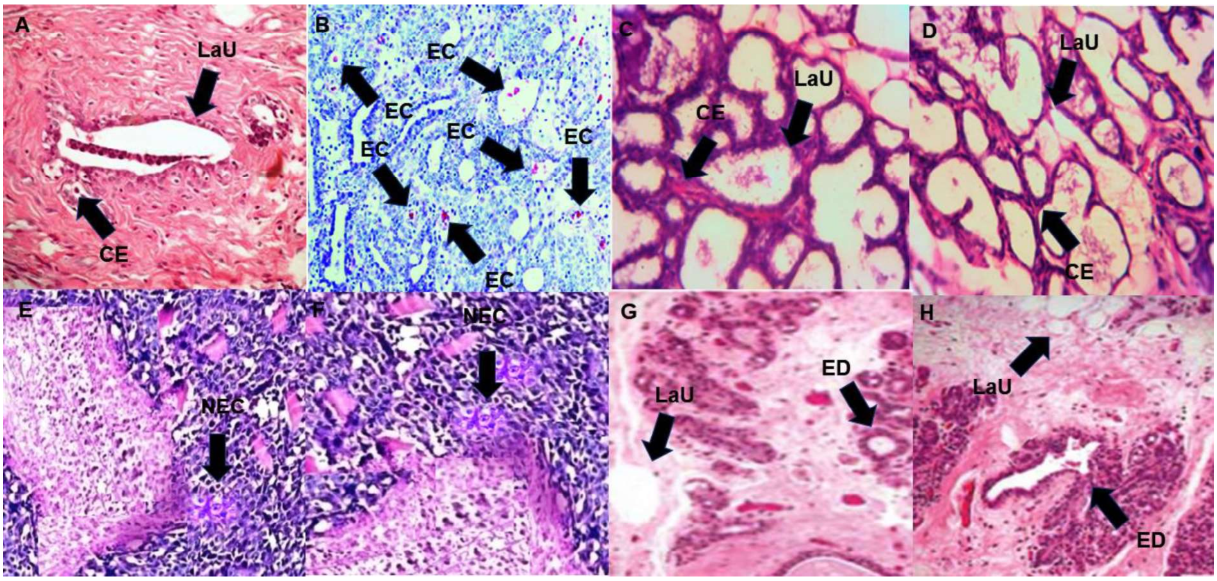

Figure S215. Photomicrograph of breast section of normal control rat showing lobuloalveolar unit (LaU) and cuboidal epithelial cells (CE) (A); photomicrograph of breast section treated with **MNU** showing mammary gland carcinoma alongside with massive proliferation of neoplastic epithelial cells (EC) (B); photomicrograph of breast section treated with **3DPQ-9** in concentration of 5 mg/kg of bwt showing lobuloalveolar unit (LaU) and cuboidal epithelial cells (CE) (C); photomicrograph of breast section treated with **3DPQ-9** in concentration of 50 mg/kg of bwt showing lobuloalveolar unit (LaU) and cuboidal epithelial cells (CE) (D); photomicrograph of breast section treated with **4-OHT** in concentration of 5 mg/kg of bwt showing necrosis (NEC) (E); photomicrograph of breast section treated with **4-OHT** in concentration of 50 mg/kg of bwt showing necrosis (NEC) (F); photomicrograph of breast section treated with **Ral** in concentration of 5 mg/kg of bwt showing differentiated extralobular ducts (ED) (G); photomicrograph of breast section treated with **Ral** in concentration of 50 mg/kg of bwt showing differentiated extralobular ducts (ED) (H), shown in x200 magnification and stained with hematoxylin and eosin.

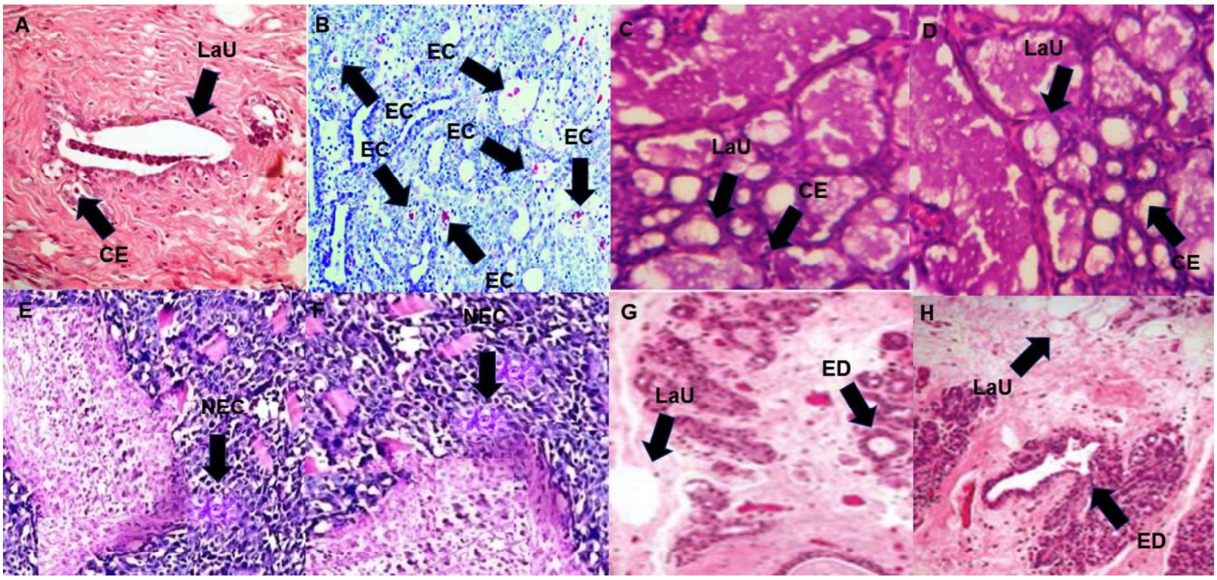

Figure S216. Photomicrograph of breast section of normal control rat showing lobuloalveolar unit (LaU) and cuboidal epithelial cells (CE) (A); photomicrograph of breast section treated with **MNU** showing mammary gland carcinoma alongside with massive proliferation of neoplastic epithelial cells (EC) (B); photomicrograph of breast section treated with **3DPQ-4** in concentration of 5 mg/kg of bwt showing lobuloalveolar unit (LaU) and cuboidal epithelial cells (CE) (C); photomicrograph of breast section treated with **3DPQ-4** in concentration of 50 mg/kg of bwt showing lobuloalveolar unit (LaU) and cuboidal epithelial cells (CE) (D); photomicrograph of breast section treated with **4-OHT** in concentration of 5 mg/kg of bwt showing necrosis (NEC) (E); photomicrograph of breast section treated with **4-OHT** in concentration of 50 mg/kg of bwt showing necrosis (NEC) (F); photomicrograph of breast section treated with **Ral** in concentration of 5 mg/kg of bwt showing differentiated extralobular ducts (ED) (G); photomicrograph of breast section treated with **Ral** in concentration of 50 mg/kg of bwt showing differentiated extralobular ducts (ED) (H), shown in x200 magnification and stained with hematoxylin and eosin.

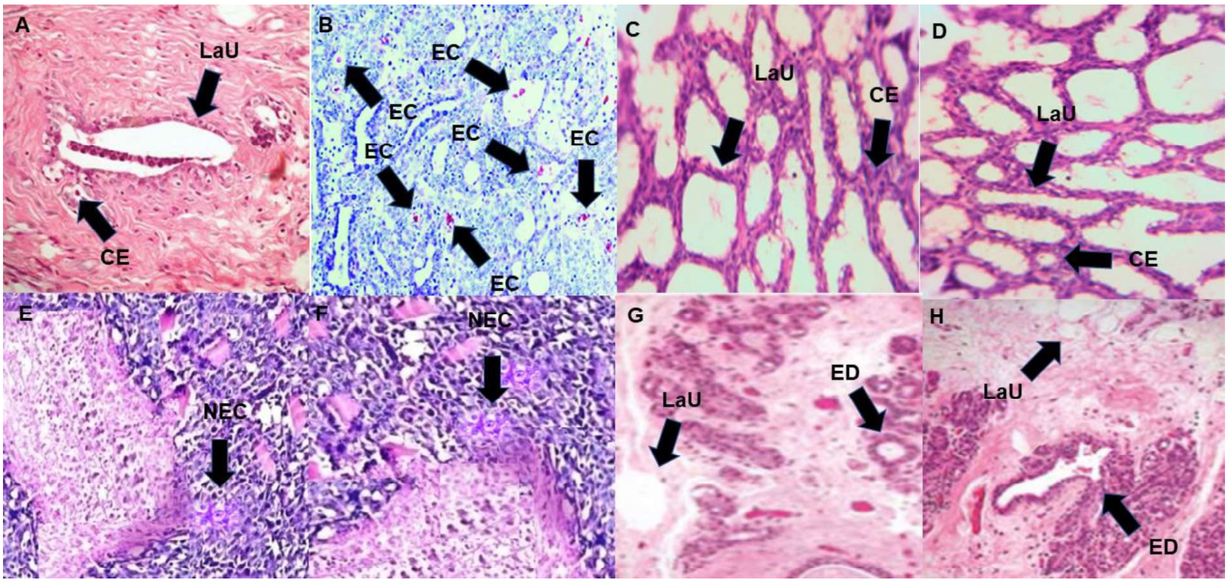

Figure S217. Photomicrograph of breast section of normal control rat showing lobuloalveolar unit (LaU) and cuboidal epithelial cells (CE) (A); photomicrograph of breast section treated with **MNU** showing mammary gland carcinoma alongside with massive proliferation of neoplastic epithelial cells (EC) (B); photomicrograph of breast section treated with **3DPQ-2** in concentration of 5 mg/kg of bwt showing lobuloalveolar unit (LaU) and cuboidal epithelial cells (CE) (C); photomicrograph of breast section treated with **3DPQ-1** in concentration of 50 mg/kg of bwt showing lobuloalveolar unit (LaU) and cuboidal epithelial cells (CE) (D); photomicrograph of breast section treated with **4-OHT** in concentration of 5 mg/kg of bwt showing necrosis (NEC) (E); photomicrograph of breast section treated with **4-OHT** in concentration of 50 mg/kg of bwt showing necrosis (NEC) (F); photomicrograph of breast section treated with **Ral** in concentration of 5 mg/kg of bwt showing differentiated extralobular ducts (ED) (G); photomicrograph of breast section treated with **Ral** in concentration of 50 mg/kg of bwt showing differentiated extralobular ducts (ED) (H), shown in x200 magnification and stained with hematoxylin and eosin.

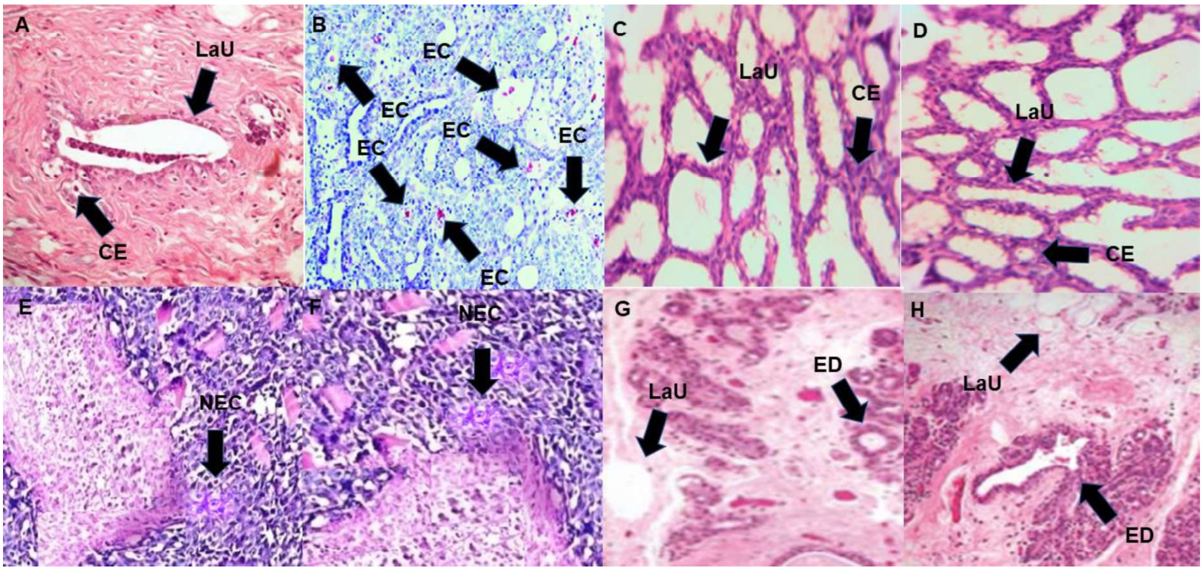

Figure S218. Photomicrograph of breast section of normal control rat showing lobuloalveolar unit (LaU) and cuboidal epithelial cells (CE) (A); photomicrograph of breast section treated with **MNU** showing mammary gland carcinoma alongside with massive proliferation of neoplastic epithelial cells (EC) (B); photomicrograph of breast section treated with **3DPQ-1** in concentration of 5 mg/kg of bwt showing lobuloalveolar unit (LaU) and cuboidal epithelial cells (CE) (C); photomicrograph of breast section treated with **3DPQ-1** in concentration of 50 mg/kg of bwt showing lobuloalveolar unit (LaU) and cuboidal epithelial cells (CE) (D); photomicrograph of breast section treated with **4-OHT** in concentration of 5 mg/kg of bwt showing necrosis (NEC) (E); photomicrograph of breast section treated with **4-OHT** in concentration of 50 mg/kg of bwt showing necrosis (NEC) (F); photomicrograph of breast section treated with **Ral** in concentration of 5 mg/kg of bwt showing differentiated extralobular ducts (ED) (G); photomicrograph of breast section treated with **Ral** in concentration of 50 mg/kg of bwt showing differentiated extralobular ducts (ED) (H), shown in x200 magnification and stained with hematoxylin and eosin.

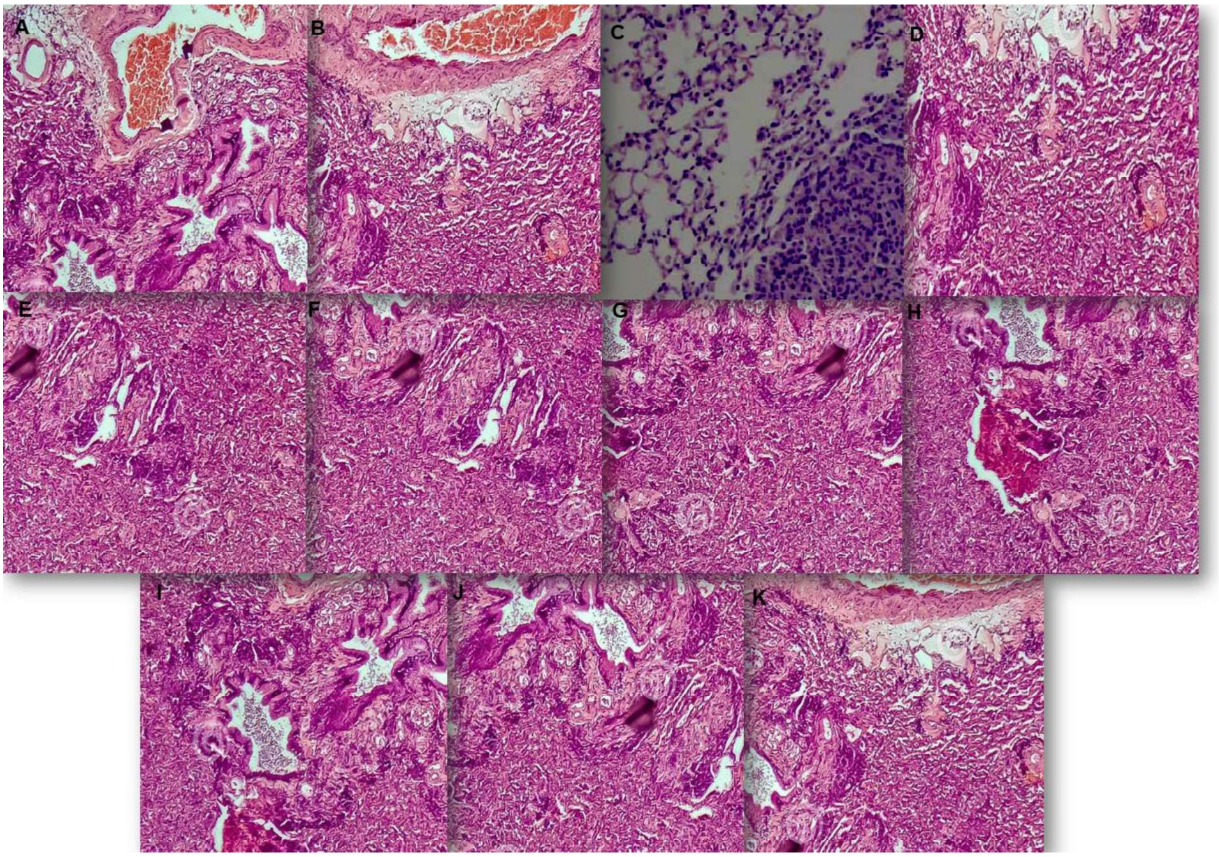

Figure S219. Photomicrograph of a lung section of a rat being treated with placebo (2% carboxylmethyl cellulose) showing no morphological changes (A); photomicrograph of a lung section of a rat being treated with saline showing no morphological changes (B); photomicrograph of a lung section of a rat being treated with **MNU** at the maximum tolerated dose of 100 mg/kg bwt showing alveolar bronchiolar single adenoma (C); photomicrograph of a lung section of a rat being treated with **4-OHT** at the maximum feasible dose of 1000 mg/kg showing no morphological changes (D); photomicrograph of a lung section of a rat being treated with **Ral** at the maximum feasible dose of 1000 mg/kg showing no morphological changes (E); photomicrograph of a lung section of a rat being treated with **3DPQ-1** at the maximum feasible dose of 1000 mg/kg showing no morphological changes (F); photomicrograph of a lung section of a rat being treated with **3DPQ-2** at the maximum feasible dose of 1000 mg/kg showing no morphological changes (G); photomicrograph of a lung section of a rat being treated with **3DPQ-3** at the maximum feasible dose of 1000 mg/kg showing no morphological changes (H); photomicrograph of a lung section of a rat being treated with **3DPQ-4** at the maximum feasible dose of 1000 mg/kg showing no morphological changes (I); photomicrograph of a lung section of a rat being treated with **3DPQ-9** at the maximum feasible dose of 1000 mg/kg showing no morphological changes (J); photomicrograph of a lung section of a rat being treated with **3DPQ-12** at the maximum feasible dose of 1000 mg/kg showing no morphological changes (K); shown in x200 magnification and stained with hematoxylin and eosin.

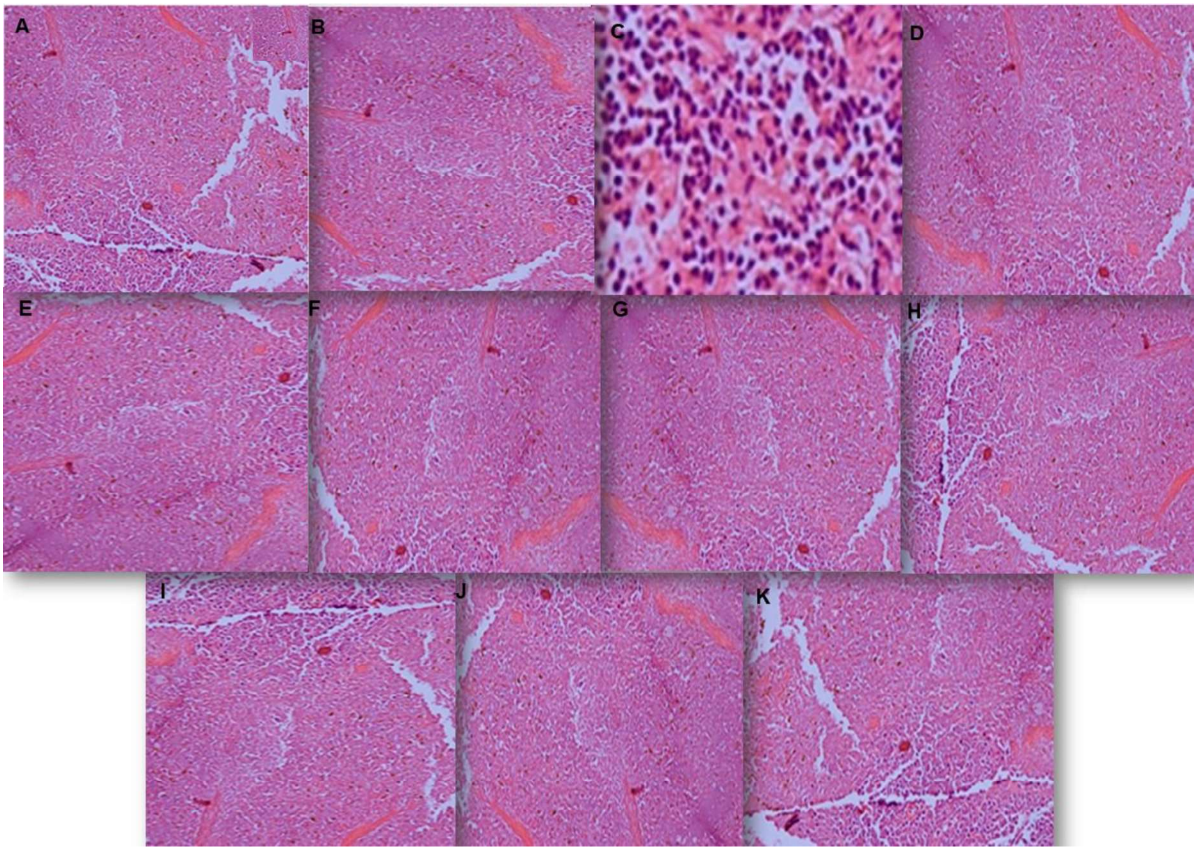

Figure S220. Photomicrograph of a spleen section of a rat being treated with placebo (2% carboxylmethyl cellulose) showing no morphological changes (A); photomicrograph of a spleen section of a rat being treated with saline showing no morphological changes (B); photomicrograph of a spleen lung section of a rat being treated with **MNU** at the maximum tolerated dose of 100 mg/kg bwt showing faded red pulp with numerous numbers of medium to large sized undifferentiated, pleomorphic nuclei (C); photomicrograph of a spleen section of a rat being treated with **4-OHT** at the maximum feasible dose of 1000 mg/kg showing no morphological changes (D); photomicrograph of a spleen section of a rat being treated with **Ral** at the maximum feasible dose of 1000 mg/kg showing no morphological changes (E); photomicrograph of a spleen section of a rat being treated with **3DPQ-1** at the maximum feasible dose of 1000 mg/kg showing no morphological changes (F); photomicrograph of a spleen section of a rat being treated with **3DPQ-2** at the maximum feasible dose of 1000 mg/kg showing no morphological changes (G); photomicrograph of a spleen section of a rat being treated with **3DPQ-3** at the maximum feasible dose of 1000 mg/kg showing no morphological changes (H); photomicrograph of a spleen section of a rat being treated with **3DPQ-4** at the maximum feasible dose of 1000 mg/kg showing no morphological changes (I); photomicrograph of a spleen section of a rat being treated with **3DPQ-9** at the maximum feasible dose of 1000 mg/kg showing no morphological changes (J); photomicrograph of a spleen section of a rat being treated with **3DPQ-12** at the maximum feasible dose of 1000 mg/kg showing no morphological changes (K); shown in x200 magnification and stained with hematoxylin and eosin.

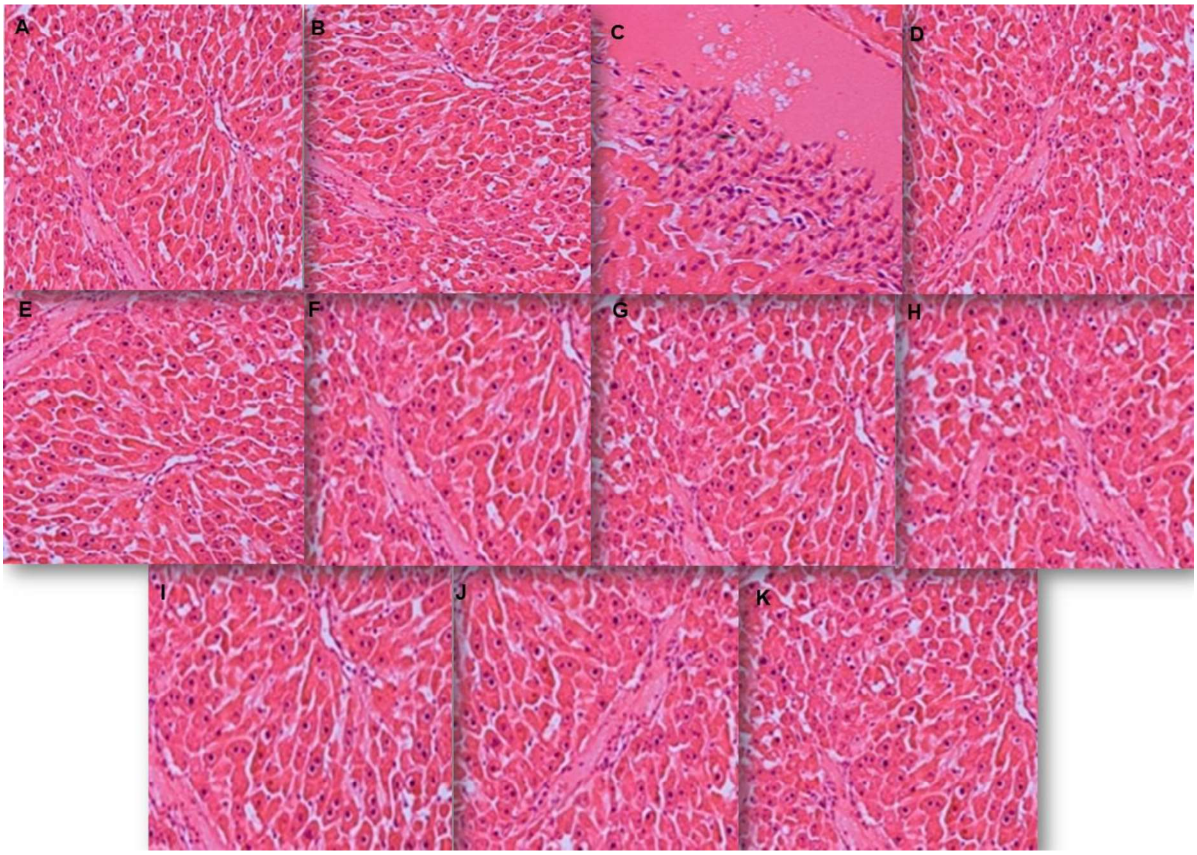

Figure S221. Photomicrograph of a liver section of a rat being treated with placebo (2% carboxylmethyl cellulose) showing no morphological changes (A); photomicrograph of a liver section of a rat being treated with saline showing no morphological changes (B); photomicrograph of a liver section of a rat being treated with **MNU** at the maximum tolerated dose of 100 mg/kg bwt showing metastasis of numerous numbers of medium to large size neoplastic lymphocytes to the liver (C); photomicrograph of a liver section of a rat being treated with **4-OHT** at the maximum feasible dose of 1000 mg/kg showing no morphological changes (D); photomicrograph of a liver section of a rat being treated with **Ral** at the maximum feasible dose of 1000 mg/kg showing no morphological changes (E); photomicrograph of a liver section of a rat being treated with **3DPQ-1** at the maximum feasible dose of 1000 mg/kg showing no morphological changes (F); photomicrograph of a liver section of a rat being treated with **3DPQ-2** at the maximum feasible dose of 1000 mg/kg showing no morphological changes (G); photomicrograph of a liver section of a rat being treated with **3DPQ-3** at the maximum feasible dose of 1000 mg/kg showing no morphological changes (H); photomicrograph of a liver section of a rat being treated with **3DPQ-4** at the maximum feasible dose of 1000 mg/kg showing no morphological changes (I); photomicrograph of a liver section of a rat being treated with **3DPQ-9** at the maximum feasible dose of 1000 mg/kg showing no morphological changes (J); photomicrograph of a liver section of a rat being treated with **3DPQ-12** at the maximum feasible dose of 1000 mg/kg showing no morphological changes (K); shown in x200 magnification and stained with hematoxylin and eosin.

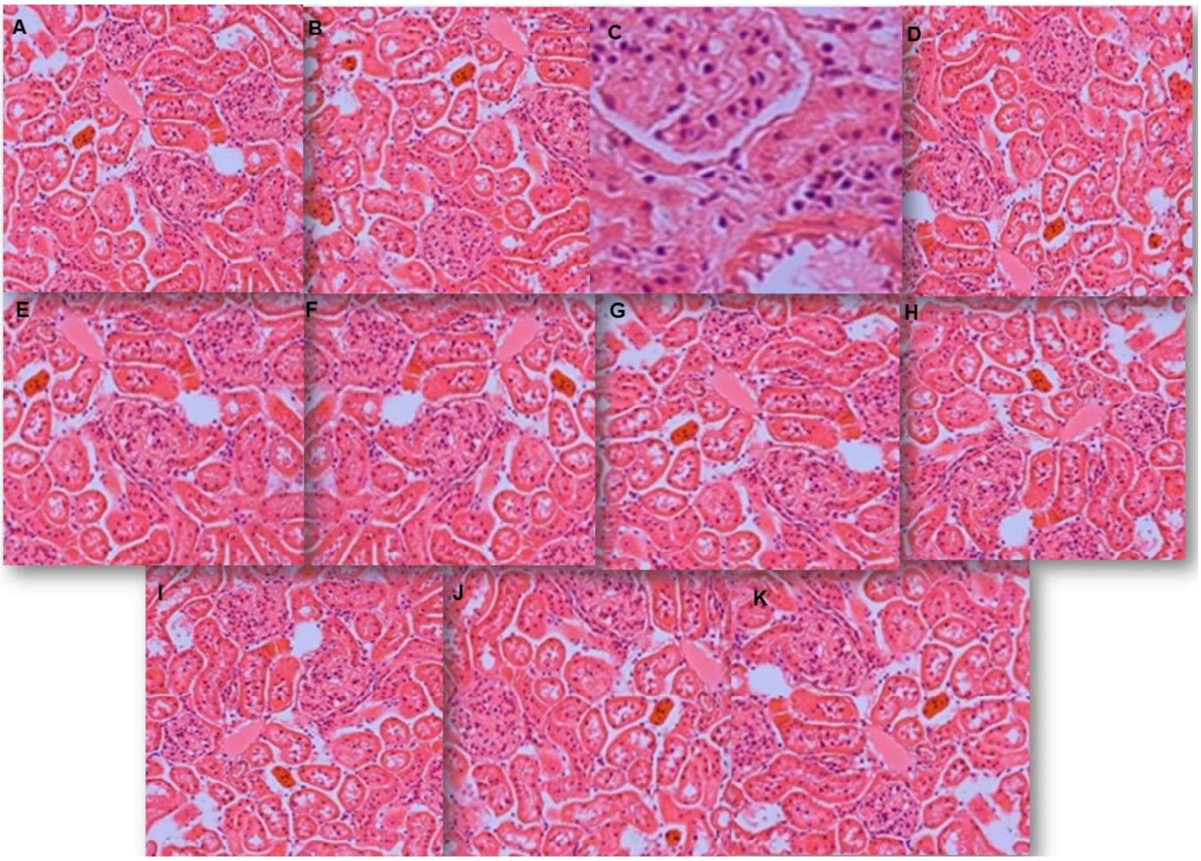

Figure S222. Photomicrograph of a kidney section of a rat being treated with placebo (2% carboxylmethyl cellulose) showing no morphological changes (A); photomicrograph of a kidney section of a rat being treated with saline showing no morphological changes (B); photomicrograph of a kidney section of a rat being treated with **MNU** at the maximum tolerated dose of 100 mg/kg bwt showing clumps of medium to large size neoplastic lymphocytes infiltrated into the interstitial space of the renal tubules and around the renal artery (C); photomicrograph of a kidney section of a rat being treated with **4-OHT** at the maximum feasible dose of 1000 mg/kg showing no morphological changes (D); photomicrograph of a kidney section of a rat being treated with **Ral** at the maximum feasible dose of 1000 mg/kg showing no morphological changes (E); photomicrograph of a kidney section of a rat being treated with **3DPQ-1** at the maximum feasible dose of 1000 mg/kg showing no morphological changes (F); photomicrograph of a kidney section of a rat being treated with **3DPQ-2** at the maximum feasible dose of 1000 mg/kg showing no morphological changes (G); photomicrograph of a kidney section of a rat being treated with **3DPQ-3** at the maximum feasible dose of 1000 mg/kg showing no morphological changes (H); photomicrograph of a kidney section of a rat being treated with **3DPQ-4** at the maximum feasible dose of 1000 mg/kg showing no morphological changes (I); photomicrograph of a kidney section of a rat being treated with **3DPQ-9** at the maximum feasible dose of 1000 mg/kg showing no morphological changes (J); photomicrograph of a kidney section of a rat being treated with **3DPQ-12** at the maximum feasible dose of 1000 mg/kg showing no morphological changes (K); shown in x200 magnification and stained with hematoxylin and eosin.

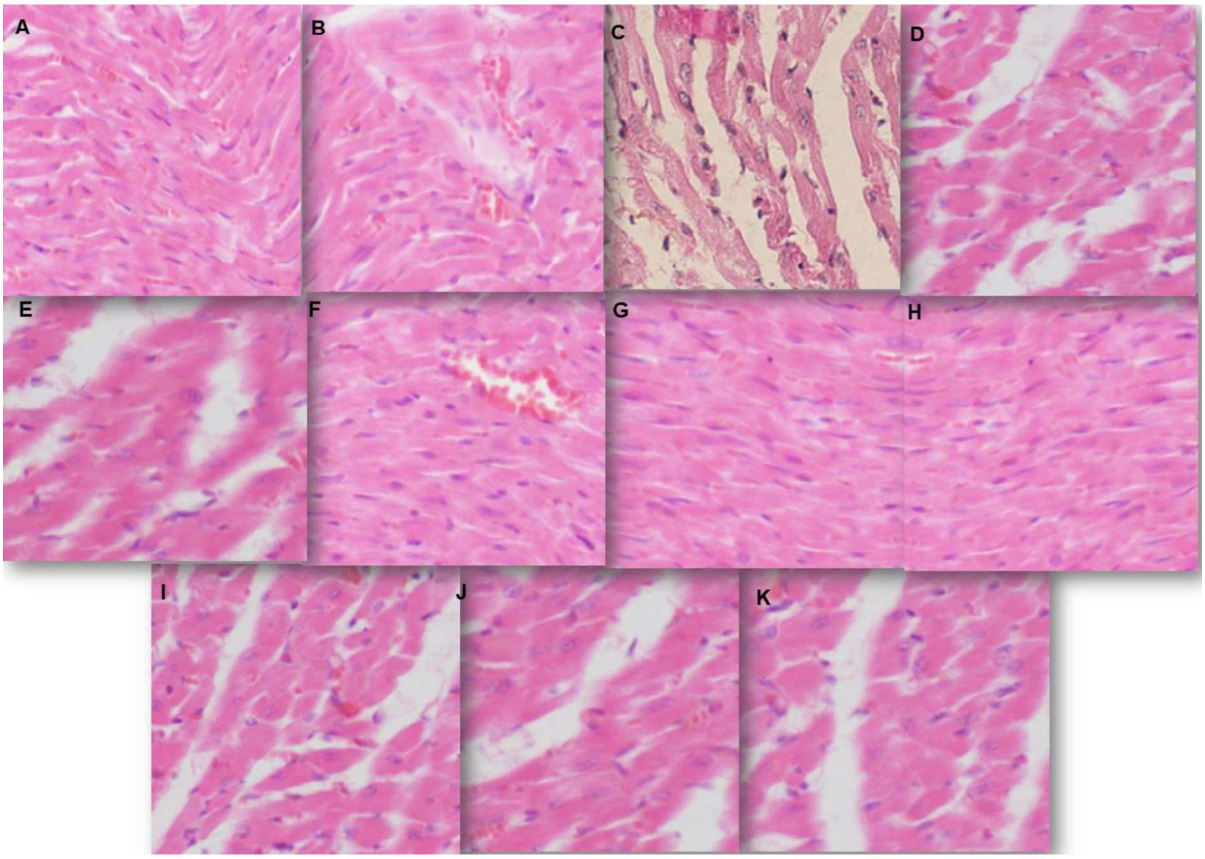

Figure S223. Photomicrograph of a heart section of a rat being treated with placebo (2% carboxylmethyl cellulose) showing no morphological changes (A); photomicrograph of a heart section of a rat being treated with saline showing no morphological changes (B); photomicrograph of a heart section of a rat being treated with **MNU** at the maximum tolerated dose of 100 mg/kg bwt showing interstitial hydrops (C); photomicrograph of a heart section of a rat being treated with **4-OHT** at the maximum feasible dose of 1000 mg/kg showing no morphological changes (D); photomicrograph of a heart section of a rat being treated with **Ral** at the maximum feasible dose of 1000 mg/kg showing no morphological changes (E); photomicrograph of a heart section of a rat being treated with **3DPQ-1** at the maximum feasible dose of 1000 mg/kg showing no morphological changes (F); photomicrograph of a heart section of a rat being treated with **3DPQ-2** at the maximum feasible dose of 1000 mg/kg showing no morphological changes (G); photomicrograph of a heart section of a rat being treated with **3DPQ-3** at the maximum feasible dose of 1000 mg/kg showing no morphological changes (H); photomicrograph of a heart section of a rat being treated with **3DPQ-4** at the maximum feasible dose of 1000 mg/kg showing no morphological changes (I); photomicrograph of a heart section of a rat being treated with **3DPQ-9** at the maximum feasible dose of 1000 mg/kg showing no morphological changes (J); photomicrograph of a heart section of a rat being treated with **3DPQ-12** at the maximum feasible dose of 1000 mg/kg showing no morphological changes (K); shown in x200 magnification and stained with hematoxylin and eosin.

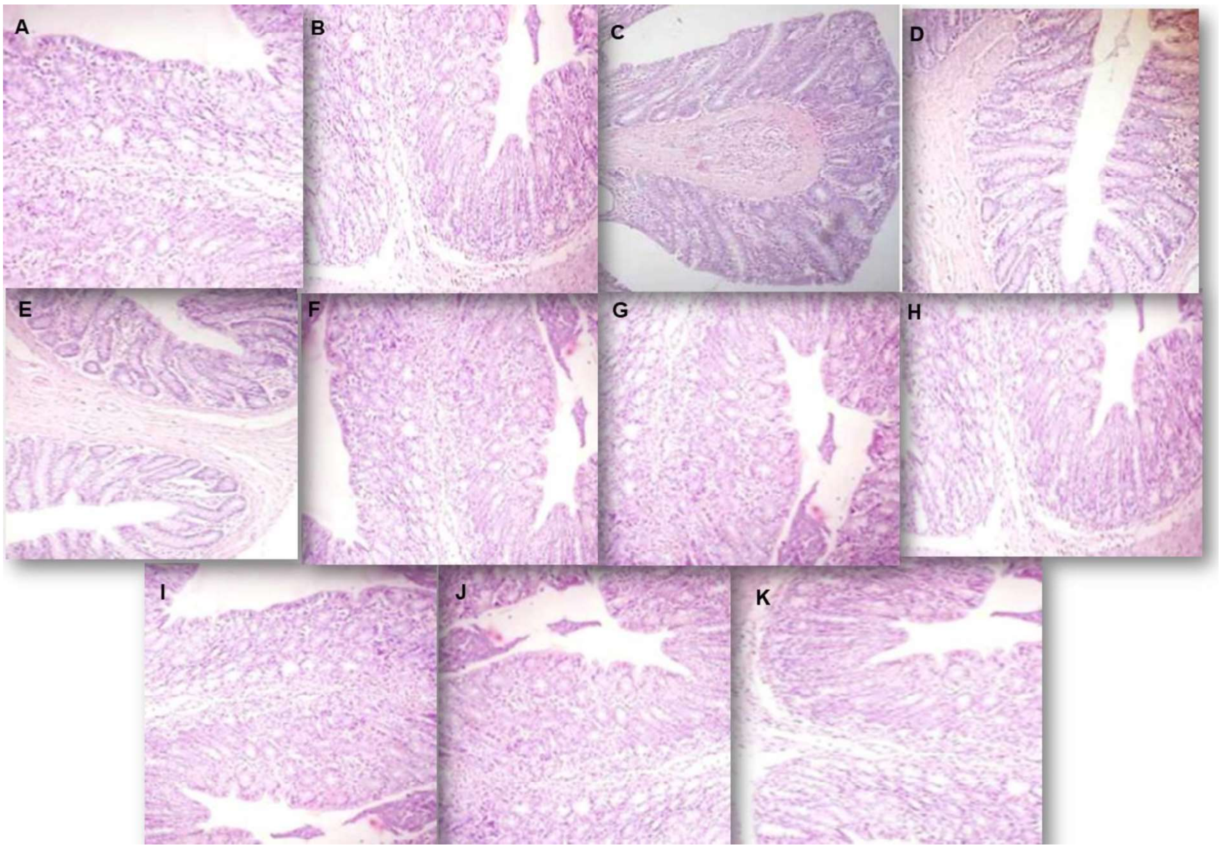

Figure S224. Photomicrograph of a colon section of a rat being treated with placebo (2% carboxylmethyl cellulose) showing no morphological changes (A); photomicrograph of a colon section of a rat being treated with saline showing no morphological changes (B); photomicrograph of a colon section of a rat being treated with **MNU** at the maximum tolerated dose of 100 mg/kg bwt showing the induced colon cancer (C); photomicrograph of a colon section of a rat being treated with **4-OHT** at the maximum feasible dose of 1000 mg/kg showing no morphological changes (D); photomicrograph of a colon section of a rat being treated with **Ral** at the maximum feasible dose of 1000 mg/kg showing no morphological changes (E); photomicrograph of a colon section of a rat being treated with **3DPQ-1** at the maximum feasible dose of 1000 mg/kg showing no morphological changes (F); photomicrograph of a colon section of a rat being treated with **3DPQ-2** at the maximum feasible dose of 1000 mg/kg showing no morphological changes (G); photomicrograph of a colon section of a rat being treated with **3DPQ-3** at the maximum feasible dose of 1000 mg/kg showing no morphological changes (H); photomicrograph of a heart section of a rat being treated with **3DPQ-4** at the maximum feasible dose of 1000 mg/kg showing no morphological changes (I); photomicrograph of a colon section of a rat being treated with **3DPQ-9** at the maximum feasible dose of 1000 mg/kg showing no morphological changes (J); photomicrograph of a colon section of a rat being treated with **3DPQ-12** at the maximum feasible dose of 1000 mg/kg showing no morphological changes (K); shown in x200 magnification and stained with hematoxylin and eosin.

Experimental

Crystal Structures Compilation and Preparation

For the purpose of TR and TS_{CRY} collection, all the available ERα complexes were initially filtered by discarding those for which any associate activity was not found in the literature (Supplementary Materials Part 1 Table S1) [13,22-43,SMR38-87]. Further selection was made by removing duplicates. Thus, estradiol as a ligand was complexed within **1ERE** [13], **1A5A** [82], **1G50** [83], **1QKU** [45] crystals, and only **1ERE** was retained. Differently, both complexes containing the genistein (**1X7R** and **2QA8**) were kept in TR as the ligands were co-crystallized within either wild-type (**1X7R**) or mutated (**2QA8**) receptors. The **5AAU** [87] and **5AAV** [87] crystals were not included in the TR since the herein 3-D QSAR models and SB and LB modeling have been already done before the particular crystals were deposited in Protein Data Bank.

All of the 39 selected ERα-antagonist complexes (Tables 1-3) were loaded into UCSF Chimera v1.10.1 software [51] and visually inspected. Complexes were superimposed using **1ERR** as a template (the best-resolved complex with the resolution of 1.6 Å) by means of the MatchMaker module and then separated in chains using the command line implementation of the Chimera split command. Compared to other chains, chains B were complete by means of antagonists' and partial agonists' presence, and therefore only chains B were retained. The antagonists were extracted from each chain B complexes, added of hydrogens, and Amber parameters were calculated by means of Antechamber using the semiempirical QM method. The protein parts of the saved monomers were improved by adding hydrogen atoms using the embedded leap module of Amber 12 suite [SMR55], upon which the correct hydrogen atoms, appropriate for pH 7.4, were assigned to each amino acid residue. Upon preparation, proteins were merged with corresponding ligands and complexes were energy minimized as follows: through the leap module, they were solvated with water molecules (TIP3P model, SOLVATEOCT Chimera command) in a box extending 10 Å in all directions, neutralized with either Na^+ or Cl^- ions, and refined by a single point minimization using the Sander module of Amber suite with maximum 1000 steps of the steepest-descent energy minimization and maximum 4000 steps of conjugate-gradient energy minimization, with a non-bonded cuttoff of 5 Å. Minimized complexes were re-aligned (**1ERR** as template), after which all of the ligands were extracted to compose the SB aligned training set ready to be utilized for the subsequent 3-D QSAR model building.

Pharmacophore modeling.

The prealigned training set was submitted to the 3-D pharmacophore and 3-D QSAR modeling by means of the "Develop Pharmacophore Hypothesys" module of PHASE [88]. Observed activities (IC_{50}) were converted to the form of the negative logarithm (pIC_{50}) before pharmacophore generation. Besides the fact that all the TR molecules were physiologically highly active, to derive the statistically best hypotheses (associated with the highest q^2 values [48,49]), the pIC_{50} activity threshold was set to 7.30 (according to the default PHASE setup) thus dividing the TR compounds on "actives" (activity higher than 7.30 pIC_{50} units, 21 compounds), *i.e.* compounds form wich the hypotheses were made, and "inactives (activity equal or lower than 7.30 pIC_{50} units, 5 compounds), *i.e.* internal prediction set. While searching for the optimal hypotheses, all the available pharmacophoric feature combinations were iteratively explored as well, taking the maximal number of features in the hypothesis available and a preferred minimum number of features as 4, keeping in mind that at least 50% of ligands should match the developed hypotheses. The generated pharmacophore hypotheses were scored with default parameters. The top two hypotheses were selected for further generation of 3-D QSAR models.

3-D QSAR modeling

The atom-based 3-D QSAR models were generated from the TR. The 3D-QSAR partial least-squares (PLS) models were built with 5 maximum PLS factors in the regression model and a grid spacing equal to 1 Å. The 3-D QSAR models were validated with test set compounds.

Alignment assessment rules.

The 3-D PhypI/3-D QSAR procedure was further ennobled with the alignment rules that give the ‘‘know-how’’ instructions for the introduction of compounds with verified activity and unknown bioactive conformation into the virtual screening/design of novel ERα antagonists [90,91]. Therefore, to learn how to align a new molecule, the reproduction bioactive conformations of the co-crystalized ERα antagonists were carefully carried out and assessed by means of either SB or LB alignment. In the case of SB alignment assessment, the reproduction of co-crystallized bioactive conformations was performed by means of molecular docking, using the scoring functions as implementED in Schrödinger’s [89] Glide [104-106], in a verified procedure comprised of *Experimental Conformation Re-Docking* (ECRD), *Randomized Conformation Re-Docking* (RCRD), *Experimental Conformation Cross-Docking* (ECCD), and *Randomized Conformation Cross-Docking* (RCCD) [80,92]. Nevertheless, docking algorithms are not yet fully optimized and reliable, and during the simulation, the proteins are still mainly considered rigid [90,91]. Therefore, the SB alignment of the new molecules could result in non-optimal positioning and lead to a higher prediction error. The latter can be in part bypassed if LB approaches are taken into account as an alternative aligning methodology. Hence, an LB alignment assessment was performed utilizing *Experimental Conformation Re-Alignment* (ECRA), *Randomized Conformation Re-Alignment* (RCRA), *Experimental Conformation Cross-Alignment* (ECCA), and *Randomized Conformation Cross-Alignment* (RCCA), by virtue of algorithms implanted in Schrödinger’s Flexible Ligand Alignment tool [89]. Further details are reported in Supplementary Materials (*Alignment assessments* subsection). Moreover, the best performing SB and LB protocols were utilized to align four test sets (TS_{CRY} and three modeled TSs: TS_{MOD1}-TS_{MOD3}, Tables 3 and 5, Supplementary Materials Tables S10-S15) without the known binding mode, as well as to perform virtual screening of 4411compounds form National Institute of Health database (*i.e.* NCI Natural Products 3, 486 compounds; NCI Diversity Sets 2 and 3, 1574 and 2351 compounds), and prioritize them for biological assay.

Structure-Based and Ligand-Based Alignment. Regarding SB alignment, Schrödinger’s [89] Glide [53-55] was evaluated for the ability to reproduce the binding mode of reversible ERα inhibitors, by means of SP and XP docking. The whole SB procedure was assessed through the four difficulty levels [44,50]:

1. *Experimental Conformation Re-Docking (ECRD)*: a procedure in which the experimental conformations (EC) are flexibly docked back into the corresponding protein, evaluating the program for its ability to reproduce the observed bound conformations.

2. *Randomized Conformation Re-Docking (RCRD)*: similar assessment to ECRD with a difference that the active site of protein is virtually occupied by conformations initially obtained from computational random optimization of corresponding co-crystallized molecules coordinates and positions. Thus, ligands were initially displaced from the active site and their experimental coordinates were changed by means of assigning new coordinates values (here by means of molecular dynamics, see *Ligand’s experimental conformations randomizations* section). Then, allocated conformations were energy minimized. Here the programs are evaluated for their ability to find the experimental pose starting from the randomized minimized conformation.

3. *Experimental Conformation Cross-Docking (ECCD)*: comparable to ECRD, but the molecular docking was performed on all the training set proteins except the corresponding natives. Here the programs are evaluated to find the ligand binding mode in the active site like the native one by means of

amino acids configuration but different in terms of amino acids induced-fit conformations, mimicking at the same time discrete protein flexibility.

4. *Randomized Conformation Cross-Docking (RCCD)*: same as the ECCD but using RCs as starting docking conformations. This is the highest level of difficulty since the program is demanded to dock any given molecule into an ensemble of protein conformations not containing the native one. The outcome is considered the most important ability of the docking program as the most accurate scoring function in the RCCD experiment is subsequently applied to any test set molecules whose experimental binding mode is unknown. The related docking accuracy (DA) is a direct function of the program's probability to find a correct binding mode for an active molecule.

LB molecular alignment was conducted with the algorithms as implemented in Schrödinger's Flexible Ligand Alignment tool (FLA) [47]. Similarly, as in the SB procedure, LB alignment assessment was conducted at four different increasing difficulty levels [80,92]:

1. *Experimental Conformation Re-Alignment (ECRA)*: extracted co-crystallized ligand structures (EC) are subjected to conformational search after which conformers ensembles are generated and superimposed to the corresponding EC, evaluating the program for its ability to reproduce the experimental conformation of a molecule using itself as template.

2. *Randomized Conformation Re-Alignment (RCRA)*: similarly to ECRA, the initial conformations used to generate the conformers ensembles were produced by a computational procedure that randomizes their coordinates and positions. As for SB align, ligands were initially displaced energy minimized to produce the random conformations. Here the program is evaluated for its ability to align an ensemble of conformers generated by known experimental conformation.

3. *Experimental Conformation Cross-Alignment (ECCA)*: like ECRA, but the molecular superimposition was performed on all of the training set molecules except the one used to generate the conformers. Here the programs are evaluated to superimpose a molecule conformers' ensemble on pre-aligned template molecules.

4. *Randomized Conformation Cross-Alignment (RCCA)*: similar to the previous but the generations of conformers were obtained from the RC conformation. As for RCCD, this procedure also represents the most difficult one for LB approach. This is the scenario for any test set molecules to be aligned and will be applied to align model's external molecules.

The alignment fitness was quantified through the evaluation of RMSD values and the subsequent docking and alignment accuracy (DA or AA) values, for SB and LB, respectively. As previously reported [44,50], DA and AA can be used to test how the docking or alignment algorithms, respectively, are capable to predict a ligand pose as close as possible to the experimentally observed and can be calculated by the following equation SM eq. 1:

$$xA = f_{rmsd} \leq a + 0.5\ (f_{rmsd} \leq b - f_{rmsd} \leq a) \qquad (SM\ eq. 1)$$

In particular xA is equal to DA in the case of docking accuracy or xA is identical to AA in the case of alignment accuracy, whereas $frmsd \leq a$ and $frmsd \leq b$ represent the fraction of aligned ligands showing an RMSD value less than 2 Å or equal to 2 Å (a coefficient) and less than 3 Å or equal to 3Å (b coefficient), respectively. The widely accepted standard is that the correctly docked/aligned conformations are those displaying an RMSD value lower than 2 Å on all heavy atoms from the crystallographic structure of the ligand conformation as found in the inhibitor-enzyme complex. Structures with RMSD between 2 and 3 Å are considered partially docked/aligned, whereas those with RMSD higher than 3 were mis-docked/misaligned and thus not considered in the DA/AA calculation.

Ligand's experimental conformations randomizations

For the purpose of randomized conformations structure-based and ligand-based alignment (see further text), ligands' experimental conformations were subjected to the simulated annealing molecular dynamics procedure implemented in MacroModel as follows [146]. Each structure was energy minimized to a low gradient. The non-bonded cutoff distances for van der Waals interactions were set to 8 Å while distances for electrostatic ones were set to 20 Å. An initial random velocity to all atoms corresponding to 310 K was applied. Three subsequent molecular dynamics were then performed. The first was carried out for 10 ps with a 1.5 fs time-step at a constant temperature of 310 K for equilibration purposes. The next molecular dynamics was carried out for 20 ps, during which the system was coupled to a 150 K thermal bath with a time constant of 5 ps. The time constant represents approximately the half-life for equilibration with the bath; consequently, the second molecular dynamics command caused the molecule to slowly cool to approximately 150 K. The third and last dynamics cooled the molecule to 50 K over 20 ps. Final energy minimization was then carried out for 250 iterations using a conjugate gradient. The minimizations and molecular dynamics were in all cases performed by implicit salvation in simulated water aqueous solution using GB/SA (Generalized Born solvent accessible surface area) keyword and the OPLS2005 force field.

Glide settings.

The Schrödinger's Glide module was used for docking analysis. Protein preparation is done by using the Protein Preparation Wizard module of Glide [104-106]. Initially, all the protein structures were preprocessed to be used as receptors for docking with the aid of (*i*) hydrogen atoms addition of; (*ii*) atomic charges assignment; (*iii*) elimination of water molecules that are not involved in ligand binding; (*iv*) missing chains and loops addition if necessary. Preprocessed protein was optimized with PROPKA and then minimized with OPSL3 force field function, which is followed by a convergence of heavy atoms of RMSD 0.3 Å. Then, the Glide's receptor grid generation wizard was used to generate a three-dimensional (3D) grid where the xyz coordinates (in Ångströms) for the computation were Xmin/Xmax = 90.000/116.000, Ymin/Ymax = 13.000/26.000, Zmin/Zmax = 84.000/110.000; the coordinates setup was performed in a manner to embrace the minimized inhibitor spanning 10 Å in all three dimensions. Flexible docking was performed by means of either the SP (standard precision) mode, for reliably docking of ligands with high accuracy, or the extra precision (XP) mode, where further elimination of false positives is accomplished by more extensive sampling and advanced scoring, resulting in even higher enrichment.

Flexible Ligand Alignment tool settings.

During the ligand-based alignment assessment, the experiments were performed by virtue of Schrödinger's Flexible Ligand Alignment tool (FLA) [89], evaluating both Flexible shape-based alignment (FSBA) and Common scaffold alignment (CSA) modes, alongside with its available modules: Largest common Bemis-Murcko scaffold (LCBMC), Maximum common substructure (MCS), and SMARTS, using the default settings.

Generation of modeled and designed compounds.

Either test sets' TS_{MOD1}, TS_{MOD2}, and TS_{MOD3} or designed compounds were drawn through the Chemaxon's msketch module [103], by means of molecular mechanics' optimization upon which the hydrogen atoms were assigned at pH 7.4. Upon structures' generation, compounds were uploaded into previously described SB and LB protocols to obtain the bioactive conformations.

Test sets alignment.

Four different external test sets, with known activity and unknown binding mode were aligned applying either the best performing SB or LB protocols (see **Results and discussion** section). The test set composing molecules were retrieved from literature reporting active ERα antagonists.

Virtual screening

Based on DA and AA values, RCCD and RCCA procedures were applied on the 4411compounds from the National Institute of Health database (*i.e.* NCI Natural Products 3, 486 compounds; NCI Diversity Sets 2 and 3, 1574 and 2351 compounds), to obtain two conformations for each compound [44,50,58,59]. Therefore, two external prediction sets (herein called SP/**3-D PhypI/3-D QSAR** and FLA's FSBA/**3-D PhypI/3-D QSAR**, respectively) were obtained and predicted by the selected **3-D PhypI/3-D QSAR model** ensemble: a score was derived by listing the average predicted pIC_{50} values obtained from the four selected 3-D QSAR models. These scores were then used, together with the corresponding predicted free binding energy from the Glide [104-106] and FLA's [89] FSBA alignment scores to compose a hit parade. Considering these three factors, according to rank-by-rank strategy, the top 18 compounds were selected and tested.

ADMETox predictions

The ADMETox properties were predicted by means of Schrödinger's QikProp module [144] and admetSAR 2.0 webserver (http://lmmd.ecust.edu.cn/admetsar2) [95], using the default setup.

Chemistry (the equipment)

Microwave-assisted reactions were carried out in a MICROSYNT Microwave Synthesis System (serial number: 130486, terminal: T640, serial number: 0804000100), manufactured by Milestone Inc. 25 Controls Dr. Shelton, CT 06484, USA. The microwaves were generated by a magnetron (serial number: 133462/13346) at a frequency of 2450 MHz, having an output energy range of 100–500 W. The flash column chromatography was performed by Sepacore® Flash System X10 (BUCHI Corporation, USA) on silica gel, pore size 60 Å, 220-440 mesh particle size, 40 µm particle size (Sigma Aldrich). The purity of obtained compounds was checked routinely by TLC using Merck Kieselgel 60 $PF_{254+366}$ silica gel. The TLC spots were detected under the UV light. Purity was also checked by gas-chromatography/mass spectrometry. The ^{1}H NMR spectra, at 500 MHz, and ^{13}C NMR spectra, at 125 MHz, were recorded on a Bruker AV-500 spectrometer (Bruker, USA) in DMSO-d_6 as solvent, using TMS ($SiMe_4$) as the internal standard. The ^{15}N NMR spectra, at 51 MHz, and ^{17}O NMR spectra, at 68 MHz, were recorded on a Bruker AVANCE III spectrometer in DMSO-d_6 as solvent, using TMS ($SiMe_4$) as the internal standard. Chemical shifts are given in ppm (abbreviations: s-singlet, d-doublet, t-triplet, q-quartet, ABq-AB quartet, m-multiplet). Coupling constants are reported in hertz. Each compound is characterized by the appropriate NMR spectrum (either ^{1}H NMR, ^{13}C NMR, ^{15}N NMR, or ^{17}O NMR, Supplementary material Figs. S1-S154), as well as with GC/MS $[M + H]^+$ ions. The thorough structural analysis of each of the synthesized compounds is reported as Supplementary material (*Synthesized compounds spectral data interpretation* section). GC/MS analyses were carried out in an Agilent 6890N/5975B gas chromatograph. All melting points of the synthesized compounds were recorded on a Kofler hot-stage apparatus (C. Reichert, Vienna, Austria) and uncorrected. The purity of the compounds was confirmed by means of HPLC chromatographic analysis, conducted with an Agilent 1260 Infinity System (Agilent Technologies, Palo Alto, USA) which consisted of a G1311C VL quaternary pump, a G1329B ALS autosampler, a G1316A thermostatted column compartment, and G1315D diode array detector. The column employed is an Agilent Zorbax AB-C18 column (5 µm, 4.6 × 250 mm) and the mobile phase consisted of water (A) and methanol (B) aqueous solution at a flow rate of 1 mL/min. The column temperature was controlled at

25°C. The injection volume of each sample was 20 μL and the chromatogram was acquired at 254 nm wavelength.

Chemistry (commercial compounds supply).

Unless otherwise noted, all reagents and solvents were obtained from commercial suppliers and used without further purification. Thus, 1,5-pentanediol (CAS No. 111-29-5), *tert*-butyldimethylsilyl chloride (CAS No. 18162-48-6), dimethylzinc solution (CAS No. 544-97-8), (-)-1,8-diazabicyclo[5.4.0]undec-7-ene ((-)-DBNE) (EC No. 229-713-7), 1-phenyl-1*H*-tetrazole-5-thiol sodium salt (CAS No. 15052-19-4), imidazole (CAS No. 288-32-4), 4-(dimethylamino)pyridine (CAS No. 1122-58-3), ammonium molybdate tetrahydrate (CAS No. 1122-58-3), 2-methoxyethoxymethyl chloride (CAS No. 3970-21-6), *N,N*-Diisopropylethylamine (CAS No. 7087-68-5), potassium osmate(VI) dihydrate (CAS No. 10022-66-9), 4-methylmorpholine N-oxide (CAS No. 7529-22-8), Sodium (meta)periodate (CAS No. 7790-28-5), potassium hexamethyldisilazide (CAS No. 40949-94-8), toluene (CAS No. 108-88-3), 2,4,6-trichlorobenzoyl chloride (CAS No. 4136-95-2), triethylamine (CAS No. 121-44-8), 4-(dimethylamino)pyridine (DMAP) (CAS No. 1122-58-3), *tert*-butyldimethylsilyl trifluoromethanesulfonate (TBSOTf) (CAS No. 69739-34-0), 2,6-lutidine (CAS No. 108-48-5), 3-acetyl-4-hydroxybenzoic acid (Product No. PH000237), 1-ethyl-3-(3-dimethylaminopropyl) carbodiimide hydrochloride (EDAC·HCl) (CAS No. 25952-53-8), 1,2-dichloroethane (CAS No. 107-06-2), *tert*-butylammonium fluoride (CAS No. 429-41-4), tetrahydrofuran (CAS No. 109-99-9), boron tribromide (CAS No. 10294-33-4) were obtained from Sigma Aldrich. More over, Brefeldin A (CAS No. 20350-15-6) was ordered from Santa Cruz, 2-chloro-*N*-methylethanamine (CAS No. 4535-90-4) was obtained from Sinfoo Biotech, 2-chloro-*N,N*-dimethylethanamine (CAS No. 107-99-3) was purchased from Allbio Pharm Co., Ltd, 2-chloroethanesulfonic acid (CAS No. 18024-00-5) was supplied by BOC Sciences, 1-(2-chloroethyl)pyrrolidine-2,5-dione (CAS No. 18024-00-5) was obtained from Clearsynth, 1-(2-chloroethyl)piperidine (CAS No. 1932-03-2) was purchased from BLD Pharm, 1-(2-chloroethyl)pyrrolidine (CAS No. 5050-41-9) was supplied by BLD Pharm, 1,2,5,6-tetrahydropyridine-3-carboxylic acid (CAS No. 498-96-4) was obtained from Aurum Pharmatech LLC, 6,7-dihydro-5*H*-pyrrolo[3,4-b]pyridine (CAS No. 147739-88-6) was purchased from MolCore BioPharmatech, 1,2-dihydroisoquinoline (CAS No. 64973-79-1) was supplied by Chemhere; decahydro-1,8-naphthyridine (CAS No. 13993-60-7) was obtained from MolPort; 6-oxo-1,2,5,6-tetrahydropyridine-3-carboxylic acid (ID: SCHEMBL11498473) was purchased from PubChem, 3-hydroxypiperidin-2-one (CAS No. 19365-08-3) was supplied by Alfa Chemistry.

Synthetic procedures

*Synthesis of 5-((tert-butyldimethylsilyl)oxy)pentanal (**R1**)*

The reagent **R1** has been prepared according to the procedure described elsewhere as white oil (12.07 g, 87%). ^{1}H NMR (500 MHz, DMSO-*d*$_6$) 9.65 (s, 10H), 3.82 – 3.66 (m, 22H), 2.57 (s, 1H), 2.70 – 2.34 (m, 89H), 1.66 (s, 1H), 1.66 – 1.38 (m, 44H), 1.12 – 0.98 (m, 97H), 0.20 – 0.07 (m, 65H). ^{13}C NMR (125 MHz, DMSO-*d*$_6$) δ 198.49 (s), 63.02 (s), 40.17 – 39.96 (m), 36.09 (s), 30.03 (s), 25.72 – 25.33 (m), 22.78 (s), 18.42 (s), -3.02 – -3.41 (m).^{17}O NMR (68 MHz, DMSO-*d*$_6$) δ 13.00 (s), 6.00 (s). MS m/z calcd. for $C_{11}H_{24}O_2Si$: $[M + H]^+$; 216.15, found 216.39.

*Synthesis of 5-methoxy-2,2,3,3,11,11,12,12-octamethyl-4,10-dioxa-3,11-disilatridecane (**R2**)*

A 1 M solution of Me_2Zn (96 mL, 96 mmol) in hexane was added to a mixture of (-)-DBNE (2.410 mL, 8.7 mmol) in toluene (24 mL) at 0°C. Then a solution of aldehyde **R1** (9.42 g, 43.56 mmol) in toluene (140 mL) was added dropwise, and the reaction mixture was stirred for 24 h at 0°C and monitored by TLC (SiO_2: Et_2O:EtOAc = 2:1 v/v as eluent). Saturated NH_4Cl solution (160 mL) was added, and the

aqueous phase was extracted with CH_2Cl_2 (3 x 100 mL). The combined organic layers were dried over Na_2SO_4, filtered, and the solvent was removed *in vacuo*. The crude product was purified by silica gel flash chromatography (Et_2O:EtOAc = 10:1 v/v as eluent) to obtain **R2** as a colorless oil in high yield (9.42 g, 88%). ^1H NMR (500 MHz, DMSO-d_6) 5.30 (s, 10H), 4.24 (s, 10H), 3.83 – 3.66 (m, 20H), 3.40 – 3.26 (m, 30H), 2.57 – 2.43 (m, 60H), 1.58 (dd, *J* = 27.9, 6.4 Hz, 42H), 1.43 (s, 3H), 1.53 – 1.22 (m, 24H), 1.10 – 0.96 (m, 90H), 0.22 – 0.08 (m, 60H). ^{13}C NMR (125 MHz, DMSO-d_6) 63.02 (s), 53.07 (s), 40.17 – 39.96 (m), 35.64 (s), 31.13 (s), 25.72 – 25.33 (m), 21.12 (s), 18.42 (s), -3.02 – -3.41 (m).^{17}O NMR (68 MHz, DMSO-d_6) δ 44.50 (s), 23.00 (s), 13.00 (s), 6.00 (s). MS m/z calcd. for $C_{12}H_{28}O_3Si$: $[M + H]^+$; 248.18, found 248.18.

*Synthesis of 1-methoxypentane-1,5-diol (**R3**)*

To the solution of **R2** (6.32 g, 25.48 mmol) in THF (260 mL), the 1*N* HCl (520 mL, 0.52 mol) was added at room temperature and the mixture was stirred for 20 min before treating with solid $NaHCO_3$. Upon adding water (20 mL), the mixture was extracted with ethyl acetate (3 x 160 mL). The combined organic layers were dried over Na_2SO_4, filtered and concentrated *in vacuo*. The crude product was purified by flash chromatography on silica gel (Et_2O:EtOAc = 10:1 v/v as eluent) to give **R3** (3.24 g. 95%) as colorless oil. ^1H NMR (500 MHz, DMSO-d_6) δ 7.08 (s, 3H), 5.30 (s, 3H), 3.80 (s, 3H), 3.46 – 3.42 (m, 5H), 3.37 – 3.23 (m, 9H), 2.57 – 2.43 (m, 18H), 1.60 (d, *J* = 20.0 Hz, 7H), 1.51 – 1.15 (m, 12H). ^{13}C NMR (125 MHz, DMSO-d_6) δ 62.44 (s), 53.07 (s), 40.17 – 39.96 (m), 35.64 (s), 33.43 (s), 21.88 (s). ^{17}O NMR (68 MHz, DMSO-d_6) δ 44.50 (s), 23.00 (s), 13.00 (s), -6.00 (s). MS m/z calcd. for $C_6H_{14}O_3$: $[M + H]^+$; 134.17, found 134.09.

*Synthesis of 5-((5-((tert-butyldimethylsilyl)oxy)-5-methoxypentyl)thio)-1-phenyl-1H-tetrazole (**R4**)*

To a cooled (0°C) solution of **R3** (2.92 g, 21.80 mmol), PPh_3 (6.28 g, 23.96 mmol) and 1-phenyl-1*H*-tetrazole-5-thiol (4.28 g, 24.00 mmol) in dry THF (220 mL), DEAD (40 wt% in toluene, 10.80 mL, 23.56 mmol) was added dropwise. After 10 min the reaction mixture was allowed to warm to room temperature and stirred for 16 h. The reaction mixture was concentrated *in vacuo* and the residue was subjected to flash chromatography on silica gel (Et_2O:EtOAc = 2:1 v/v as eluent). The crude product was dissolved in dry CH_2Cl_2 (56 mL) and the solution was cooled to 0°C. Then imidazole (3.72 g, 54.80 mmol), DMAP (664 mg, 5.55 mmol) and TBS-Cl (3.92 g, 26 mmol) were added and the solution was allowed to warm to room temperature and, stirred for 3 h. Water (120 mL) was added, and the aqueous phase was separated and extracted with CH_2Cl_2 (3 x 80 mL). The combined organic layers were dried over Na_2SO_4, filtered and concentrated in vacuo. The crude product was subjected to flash chromatography on silica gel (Et_2O:EtOAc = 40:1 v/v as eluent) to yield **R4** (6.22 g, 70 %) as colorless oil. ^1H NMR (500 MHz, DMSO-d_6) δ 7.91 – 7.71 (m, 10H), 7.33 – 7.29 (m, 8H), 7.12 (s, 5H), 5.30 (s, 5H), 3.40 – 3.26 (m, 15H), 3.00 – 2.96 (m, 8H), 2.57 – 2.43 (m, 30H), 1.68 (dd, *J* = 25.9, 20.4 Hz, 20H), 1.59 – 1.51 (m, 1H), 1.51 – 1.17 (m, 11H), 1.10 – 0.96 (m, 45H), 0.28 – 0.14 (m, 30H). ^{13}C NMR (125 MHz, DMSO-d_6) δ 157.35 (s), 134.73 (s), 129.78 – 129.46 (m), 125.12 – 124.73 (m), 100.70 (s), 55.30 (s), 40.17 – 39.96 (m), 33.89 (s), 33.21 (s), 29.33 (s), 25.76 – 25.55 (m), 23.25 (s), 18.59 (s), -2.89 – -3.29 (m). ^{15}N NMR (51 MHz, DMSO-d_6) δ 5.10 (s), -30.50 (s), -100.50 (s), -148.10 (s). ^{17}O NMR (68 MHz, DMSO-d_6) δ 23.00 (s), 16.80 (s), 13.00 (s). MS m/z calcd. for $C_{19}H_{32}O_2SSi$: $[M + H]^+$; 408.20, found 408.63.

*Synthesis of 5-((5-((tert-butyldimethylsilyl)oxy)-5-methoxypentyl)sulfonyl)-1-phenyl-1H-tetrazole (**R5**)*

To a a solution of **R4** (6.02 g, 14.76 mmol) in EtOH (50 mL) the $(NH_4)_6Mo_7O_4{\cdot}4H_2O$ (3.64 g, 2.96 mmol) was added, followed by H_2O_2 (30 wt% in H_2O, 16.8 mL, 158.4 mmol) in small portions. The reaction mixture was stirred for 16 h at room temperature and then water (200 mL) and CH_2Cl_2 (200 mL) were added. The aqueous layer was separated and extracted with CH_2Cl_2 (3 x 120 mL). The organic layers were washed with brine (200 mL), combined and dried over Na_2SO_4. After filtration, the solvent was evaporate *in vacuo* and the residue was subjected to flash chromatography on silica gel (Et_2O:EtOAc

= 2:1 v/v as eluent) to give **R5** as colorless oil (4.88 g, 75%). ^1H NMR (500 MHz, DMSO-d_6) δ 8.38 – 8.19 (m, 12H), 7.48 – 7.37 (m, 11H), 7.14 (s, 6H), 5.08 (s, 6H), 3.54 – 3.50 (m, 9H), 3.40 – 3.26 (m, 18H), 2.57 – 2.43 (m, 36H), 1.98 – 1.94 (m, 8H), 1.68 (d, *J* = 49.0 Hz, 11H), 1.62 – 1.56 (m, 2H), 1.56 – 1.22 (m, 13H), 1.13 – 0.99 (m, 54H), 0.27 – 0.13 (m, 36H). ^{13}C NMR (125 MHz, DMSO-d_6) δ 159.95 (s), 134.73 (s), 129.78 – 129.46 (m), 125.12 – 124.73 (m), 100.70 (s), 55.30 (s), 49.88 (s), 40.17 – 39.96 (m), 33.89 (s), 25.76 – 25.55 (m), 23.24 (d, *J* = 2.4 Hz), 18.59 (s), -2.89 – -3.29 (m). ^{15}N NMR (51 MHz, DMSO-d_6) δ 5.10 (s), -30.50 (s), -81.50 (s), -148.10 (s). ^{17}O NMR (68 MHz, DMSO-d_6) δ 147.15 – 146.63 (m), 23.00 (s), 16.80 (s), 13.00 (s). MS m/z calcd. for $C_{19}H_{32}O_4SSi$: [M + H]$^+$; 440.19, found 440.19.

*Synthesis of (R,E)-methyl 5-((1R,2R,4R)-2-formyl-4-((2-methoxyethoxy)methoxy)cyclopentyl)-5-((2-methoxyethoxy)methoxy)pent-3-enoate (**R6**)*

The reagent **R6** has been prepared according to the procedure described elsewhere as colorless oil (5.42 g, 68%) [67,68]. ^1H NMR (500 MHz, DMSO-d_6) δ 9.72 (s, 2H), 6.03 (s, 2H), 5.56 (s, 2H), 4.37 – 4.23 (m, 8H), 3.89 – 3.55 (m, 9H), 3.55 (s, 2H), 3.73 – 3.39 (m, 18H), 3.39 – 3.23 (m, 14H), 3.01 – 2.82 (m, 4H), 2.57 – 2.43 (m, 12H), 2.14 (s, 2H), 2.08 – 1.84 (m, 6H), 1.70 (s, 2H), 1.46 (s, 2H). 13C NMR (125 MHz, DMSO-d_6) 205.39 (s), 173.10 (s), 134.50 (s), 131.67 (s), 94.14 (s), 93.28 (s), 80.61 (s), 78.25 (s), 73.77 – 73.55 (m), 67.93 – 67.53 (m), 57.91 – 57.70 (m), 52.86 (s), 51.82 (s), 43.69 (s), 40.17 – 39.96 (m), 37.56 (s), 36.28 (s), 34.95 (s). ^{17}O NMR (68 MHz, DMSO-d_6) δ 363.00 (s), 142.70 (s), 38.65 – 38.09 (m), 18.36 – 17.60 (m), 13.00 (s), -23.44 – -24.21 (m). MS m/z calcd. for $C_{20}H_{34}O_9$: [M + H]$^+$; 418.22, found 418.48.

*Synthesis of (4R,E)-*methyl *4-((1R,2S,4S)-2-((E)-*6-((tert-butyldimethylsilyl)oxy)-6-methoxyhex-1-en-1-yl)-4-((2-methoxyethoxy)methoxy)cyclopentyl)-4-((2-methoxyethoxy)methoxy)but-2-enoate (**R7**)

To a cooled (-78°C) solution of **R5** (3.7 g, 8.4 mmol) in dry 1,2-dimethoxyethane (5.2 mL, freshly dried over sodium), a solution of KHMDS (0.5 M in toluene, 15.6 mL, 7.8 mmol) was added dropwise. After 30 min a solution of freshly prepared aldehyde **R6** (2.52 g, 6.24 mmol) in dry 1,2-dimethoxyethane (31.2 mL) was added dropwise over a period of 10 min. After 2 h at -78 °C, the solution was allowed to warm to room temperature and was stirred for further 18 h. Then water (100 mL) and brine (100 mL) were added, and the mixture was extracted with ethyl acetate (3 x 120 mL). The combined organic layers were dried over Na_2SO_4, filtered, and the solvent was evaporated in vacuo. The residue was subjected to flash chromatography on silica gel (Et_2O:EtOAc = 3:1 v/v as eluent) to give **R7** (3.9 g, 75%) as colorless oil. ^1H NMR (500 MHz, DMSO-d_6) 6.82 (s, 2H), 6.22 (s, 2H), 5.46 (t, *J* = 9.2 Hz, 6H), 5.24 (s, 2H), 4.82 (s, 2H), 4.37 – 4.23 (m, 4H), 3.91 – 3.80 (m, 6H), 3.79 (d, *J* = 4.6 Hz, 1H), 3.79 – 3.56 (m, 9H), 3.55 – 3.36 (m, 9H), 3.47 – 3.36 (m, 3H), 3.35 (dtd, *J* = 36.9, 3.3, 1.6 Hz, 23H), 2.57 – 2.43 (m, 12H), 2.02 (s, 1H), 1.84 (t, *J* = 27.8 Hz, 7H), 1.77 (s, 2H), 1.77 (s, 2H), 1.62 (dd, *J* = 17.6, 6.3 Hz, 8H), 1.38 (dd, *J* = 26.5, 4.8 Hz, 8H), 1.12 – 0.98 (m, 18H), 0.24 – 0.10 (m, 12H). ^{13}C NMR (125 MHz, DMSO-d_6) δ 167.40 (s), 146.54 (s), 132.97 (s), 130.82 (s), 127.49 (s), 100.70 (s), 94.14 (s), 93.28 (s), 80.42 (s), 78.94 (s), 73.77 – 73.55 (m), 67.93 – 67.53 (m), 57.91 – 57.70 (m), 55.30 (s), 51.97 (s), 49.03 (s), 44.98 (s), 40.17 – 39.96 (m), 39.54 (s), 37.46 (s), 32.63 (s), 32.41 (s), 25.76 – 25.55 (m), 22.92 (s), 18.59 (s), -2.89 – -3.29 (m). ^{17}O NMR (68 MHz, DMSO-d_6) δ 335.30 (s), 130.70 (s), 38.65 – 38.09 (m), 23.00 (s), 18.36 – 17.60 (m), 16.80 (s), 13.00 (s), -23.44 – -24.21 (m). MS m/z calcd. for $C_{31}H_{58}O_{10}Si$: [M + H]$^+$; 618.38, found 618.87.

*Synthesis of (4R,E)-methyl 4-((1R,2S,4S)-2-((E)-6-hydroxy-6-methoxyhex-1-en-1-yl)-4-((2-methoxyethoxy)methoxy)cyclopentyl)-4-((2-methoxyethoxy)methoxy)but-2-enoate (**R8**)*

To a solution of **R7** (3.22 g, 5.2 mmol) in THF (90 mL) 1*N* HCl (15 mL, 15 mmol) has been added at room temperature and the mixture was stirred for 2 h before it was cautiously treated with solid $NaHCO_3$. Water (200 mL) was added, and the mixture was extracted with CH_2Cl_2 (3 x 120 mL). The organic layers were dried over Na_2SO_4, filtered and concentrated *in vacuo*. The crude product was purified by flash chromatography on silica (Et_2O:EtOAc = 3:1 v/v as eluent) to give **R8** (2.34 g, 89%) as

colorless oil. ^{1}H NMR (500 MHz, DMSO-d_6) δ 6.82 (s, 16H), 6.08 (s, 16H), 5.46 (t, *J* = 3.9 Hz, 48H), 5.30 (s, 15H), 5.24 (s, 17H), 4.37 – 4.22 (m, 46H), 3.89 – 3.67 (m, 64H), 3.63 – 3.26 (m, 283H), 3.26 – 3.20 (m, 4H), 2.57 – 2.43 (m, 93H), 2.07 – 1.83 (m, 76H), 1.77 (s, 13H), 1.74 – 1.48 (m, 50H), 1.49 – 1.18 (m, 50H). ^{13}C NMR (125 MHz, DMSO-d_6) δ 167.40 (s), 146.54 (s), 132.97 (s), 130.82 (s), 127.49 (s), 94.14 (s), 93.28 (s), 80.42 (s), 78.94 (s), 73.77 – 73.55 (m), 67.93 – 67.53 (m), 57.91 – 57.70 (m), 53.07 (s), 51.97 (s), 49.03 (s), 44.98 (s), 40.17 – 39.96 (m), 39.54 (s), 37.46 (s), 34.25 (s), 32.41 (s), 22.77 (s). ^{17}O NMR (68 MHz, DMSO-d_6) δ 335.30 (s), 130.70 (s), 44.50 (s), 38.65 – 38.09 (m), 23.00 (s), 18.36 – 17.60 (m), 13.00 (s), -23.44 – -24.21 (m). MS m/z calcd. for $C_{25}H_{44}O_{10}$: $[M + H]^+$; 504.29, found 504.61.

*Synthesis of (1R,2E,6R,10E,11aS,13S,14aR)-6-methoxy-1,13-bis((2-methoxyethoxy)methoxy)-6,7,8,9,12,13,14,14a-octahydro-1H-cyclopenta[f][1]oxacyclotridecin-4(11aH)-one (**BFA-D1**)*

To a stirred solution of **R8** (2 g, 3.96 mmol) in THF/H_2O (1.3:1, 90 mL) 1*N* LiOH (9 mL, 9.2 mmol) has been added and the mixture was stirred at room temperature for 2 h. Following, the solution was treated with the acidic ion exchange resin Amberlite® IR-120 until the mixture reached pH 7. Upon filtration through a pad of Celite®, the solvent was removed *in vacuo*, the residue was dissolved in EtOAc and filtered to give a colorless oil. Then, NEt_3 (1020 µL, 7.2 mmol) was added to a solution of the crude product in dry THF (40 mL), followed by 2,4,6-trichlorobenzoyl chloride (1060 µL, 6.72 mmol). The mixture was stirred for 1.5 h at room temperature before dilution in dry toluene (600 mL) and transfer dropwise *via* syringe (20 mL/h) to a refluxing solution of DMAP (1.22 g, 10 mmol) in dry toluene (24 mL). The mixture was than refluxed for 5 h, cooled down to room temperature and treated with Et_2O (420 mL) and water (210 mL). The aqueous layer was separated and extracted with Et_2O (2 x 100 mL). The organic layers were washed with saturated $NaHCO_3$ solution (160 mL) and brine (160 mL). The combined organic layers were dried over Na_2SO_4, filtered and concentrated *in vacuo*. The crude product was purified by flash chromatography on silica gel (Et_2O:EtOAc = 3:1 v/v as eluent) to give **BFA-D1** (1.68 g, 90%) as colorless oil. ^{1}H NMR (500 MHz, DMSO-d_6) δ 7.07 (s, 41H), 6.37 (s, 40H), 6.04 (s, 42H), 5.46 (t, *J* = 7.5 Hz, 122H), 5.14 (s, 41H), 4.37 – 4.23 (m, 80H), 3.78 (s, 46H), 3.68 – 3.56 (m, 162H), 3.56 – 3.39 (m, 168H), 3.39 – 3.28 (m, 356H), 3.28 – 3.22 (m, 46H), 2.57 – 2.43 (m, 239H), 2.23 – 2.03 (m, 118H), 2.00 (s, 51H), 1.97 – 1.77 (m, 128H), 1.78 – 1.41 (m, 201H), 1.45 (s, 7H). ^{13}C NMR (125 MHz, DMSO-d_6) δ 164.94 (s), 147.26 (s), 133.17 (s), 130.82 (s), 127.66 (s), 101.68 (s), 94.14 (s), 93.28 (s), 79.82 (d, *J* = 10.3 Hz), 73.77 – 73.55 (m), 67.93 – 67.53 (m), 57.91 – 57.70 (m), 55.30 (s), 51.19 (s), 48.01 (s), 40.17 – 39.96 (m), 38.81 (s), 36.73 (s), 33.05 (s), 32.41 (s), 22.92 (s). ^{17}O NMR (68 M Hz, DMSO-d_6) δ 332.00 (s), 38.65 – 38.09 (m), 23.00 (s), 18.36 – 17.60 (m), 13.00 (s), -23.44 – -24.21 (m). MS m/z calcd. for $C_{24}H_{40}O_9$: $[M + H]^+$; 472.27, found 472.27.

*Synthesis of (1R,2E,6R,10E,11aS,13S,14aR)-1,13-dihydroxy-6-methoxy-6,7,8,9,12,13,14,14a-octahydro-1H-cyclopenta[f][1]oxacyclotridecin-4(11aH)-one (**BFA-D2**)*

To a solution of **BFA-D1** (1600 mg, 3.38 mmol) in THF (120 mL) the cc. HBr (120 mL) has been added and the mixture was stirred for 1.5 h at room temperature. Then solid $NaHCO_3$ was cautiously added before diluting with water (300 mL). The mixture was extracted with EtOAc (3 x 90 mL). The organic layers were combined, dried over Na_2SO_4, filtered and concentrated in vacuo. The residue was subjected to flash chromatography on silica gel (Et_2O:EtOAc = 1:1 v/v as eluent), followed by recrystallization from MeOH/H_2O to give **BFA-D2** (800 mg, 80%) as colorless needles. ^{1}H NMR (500 MHz, DMSO-d_6) δ 7.07 (s, 1H), 6.37 (s, 1H), 6.04 (s, 1H), 5.46 (d, *J* = 17.9 Hz, 2H), 4.19 (s, 1H), 4.06 (d, *J* = 17.0 Hz, 2H), 3.61 (s, 1H), 3.38 – 3.24 (m, 3H), 2.57 – 2.43 (m, 6H), 2.08 (d, *J* = 12.8 Hz, 2H), 1.97 (dd, *J* = 25.3, 12.0 Hz, 4H), 1.74 (d, *J* = 14.1 Hz, 2H), 1.59 – 1.48 (m, 3H), 1.46 (s, 1H). ^{13}C NMR (125 MHz, DMSO-d_6) δ 164.94 (s), 150.10 (s), 133.17 (s), 130.82 (s), 124.30 (s), 101.68 (s), 75.76 (s), 73.67 (s), 55.30 (s), 50.14 (s), 47.03 (s), 41.34 (s), 40.17 – 39.96 (m), 37.97 (s), 33.05 (s), 32.41 (s), 22.92 (s). ^{17}O NMR (68 MHz, DMSO-d_6) δ 332.00 (s), 33.40 (s), 32.30 (s), 23.00 (s), 13.00 (s). MS m/z calcd. for $C_{16}H_{24}O_5$: $[M + H]^+$ m/z: 296.16, found 296.36.

*Synthesis of (1R,2E,6R,10E,11aS,13S,14aR)-13-((tert-butyldimethylsilyl)oxy)-1-hydroxy-6-methoxy-6,7,8,9,12,13,14,14a-octahydro-1H-cyclopenta[f][1]oxacyclotridecin-4(11aH)-one (**BFA-D3**)*

The **BFA-D2** (700 mg, 3.36 mmol) has been dissolved in dichloromethane (2.3 mL) at 0°C in round-bottomed flask after which the 2,6-lutidine (285 µL) was added. Then, the TBSOTf (0.825 mL, 3.3 mmol) was added dropwise and the reaction mixture was allowed to warm to room temperature and stir for 8 h. The reaction was quenched by the addition of a 5% $NaHCO_3$ solution (10 mL). Upon extraction with CH_2Cl_2 (3 × 45 mL) and drying over Na_2SO_4, the solvent was evaporated *in vaccuo*, and the crude product was purified by flash chromatography on silica gel (*n*-hexane:EtOAc = 8:2 v/v as eluent) to give **BFA-D3** was isolated as a thick clear oil (356 mg, 25%). ^{1}H NMR (500 MHz, DMSO-d_6) δ 7.07 (s, 2H), 6.37 (s, 2H), 6.04 (s, 2H), 5.47 (d, *J* = 13.9 Hz, 4H), 4.08 (s, 2H), 3.83 (s, 2H), 3.61 (s, 2H), 3.36 – 3.22 (m, 6H), 2.57 – 2.43 (m, 12H), 2.01 (d, *J* = 4.8 Hz, 4H), 2.04 – 1.94 (m, 6H), 2.16 – 1.69 (m, 18H), 2.04 – 1.71 (m, 14H), 2.07 – 1.69 (m, 15H), 1.71 – 1.36 (m, 7H), 1.10 – 0.96 (m, 18H), 0.23 – 0.09 (m, 12H). ^{13}C NMR (125 MHz, DMSO-d_6) δ 164.94 (s), 150.10 (s), 132.97 (s), 130.82 (s), 124.30 (s), 101.68 (s), 75.73 (s), 73.06 (s), 55.30 (s), 48.29 (s), 44.80 (s), 40.05 (t, *J* = 3.2 Hz), 37.10 (s), 33.05 (s), 32.41 (s), 25.76 – 25.55 (m), 22.92 (s), 18.59 (s), -2.89 – -3.29 (m). ^{17}O NMR (68 MHz, DMSO-d_6) δ 332.00 (s), 32.30 (s), 31.40 (s), 23.00 (s), 13.00 (s). MS m/z calcd. for $C_{23}H_{40}O_5Si$: $[M + H]^+$ m/z: 424.26, found 424.65.

*Synthesis of (1R,2E,6R,10E,11aS,13S,14aR)-13-((tert-butyldimethylsilyl)oxy)-6-methoxy-4-oxo-4,6,7,8,9,11a,12,13,14,14a-decahydro-1H-cyclopenta[f][1]oxacyclotridecin-1-yl 3-acetyl-4-hydroxybenzoate (**BFA-D4**)*

To a suspension of **BFA-D3** (300 mg, 0.7 mmol) was dissolved 30.0 mL anhydrous dichloromethane under argon, and commercial 3-acetyl-4-hydroxybenzoic acid (4 equiv, 426 mg, 2.8 mmol) was added slowly, followed by 1-ethyl-3-(3-dimethylaminopropyl) carbodiimide hydrochloride (EDC·HCl, 4 equiv, 537 mg, 2.8 mmol) and 4-dimethylamiopryidine (DMAP, 1 equiv, 85.52 mg, 0.7 mmol). The mixture maintained at 40-50°C with stirring for 24 h. The progress of the reaction was monitored by silica gel TLC. The reaction was quenched with water (30 mL), and the mixture was extracted with dichloromethane (3 x 30 mL), then dried with Na_2SO_4, and the solvent was removed under reduced pressure. The crude product was purified by column chromatography on silica gel (*n*-hexane:EtOAc = 1:1 as eluent) to obtain **BFA-D4** as white solid (368 mg, 95%). ^{1}H NMR (500 MHz, DMSO-d_6) δ 8.62 (s, 1H), 8.44 (s, 1H), 8.00 (s, 1H), 7.07 (s, 1H), 7.00 (s, 1H), 6.37 (s, 1H), 6.04 (s, 1H), 5.48 (s, 1H), 5.19 (d, *J* = 39.5 Hz, 2H), 3.61 (s, 1H), 3.40 – 3.26 (m, 3H), 2.60 – 2.43 (m, 9H), 2.36 – 1.71 (m, 8H), 2.05 – 1.71 (m, 5H), 2.02 – 1.71 (m, 5H), 1.75 (s, 1H), 1.75 (s, 1H), 1.66 (d, *J* = 13.6 Hz, 2H), 1.52 (d, *J* = 49.0 Hz, 2H), 1.07 – 0.93 (m, 9H), -0.20 – -0.34 (m, 6H). ^{13}C NMR (125 MHz, DMSO-d_6) δ 202.48 (s), 166.92 (s), 165.45 (s), 164.94 (s), 143.04 (s), 136.88 (s), 132.97 (s), 131.08 (s), 130.82 (s), 127.47 (s), 122.75 (s), 119.54 (s), 116.90 (s), 101.68 (s), 79.05 (s), 73.06 (s), 55.30 (s), 46.98 (s), 44.98 (s), 40.05 (t, *J* = 3.2 Hz), 37.43 (s), 33.05 (s), 32.41 (s), 28.28 (s), 25.76 – 25.55 (m), 22.92 (s), 18.59 (s), -2.89 – -3.29 (m). ^{17}O NMR (68 MHz, DMSO-d_6) δ 488.00 (s), 338.90 (s), 332.00 (s), 130.60 (s), 85.80 (s), 31.40 (s), 23.00 (s), 13.00 (s). MS m/z calcd. for $C_{31}H_{44}O_8Si$: $[M + H]^+$ m/z: 572.28, found 572.76.

*General procedure for the synthesis of **Pro-R3**, **Pro-R6**, **Pro-R7**, **Pro-R8**, **Pro-R9**, and **Pro-R12***

While the pro-reagents **Pro-R1, Pro-R2**, **Pro-R4**, **Pro-R5**, **Pro-R10** and **Pro-R11** were commercially available, **Pro-R3**, **Pro-R6**, **Pro-R7**, **Pro-R8**, **Pro-R9,** and **Pro-R12** required to be synthesized. In that sense, an amount of 2-chloroethan-1-ol (50 mg, 625 µmol) was dissolved in 45 mL of absolute acetone. Then, potassium carbonate (25.4 mg, 2 mmol), potassium iodide (103.8 mg, 625 µmol), and either commercially available 1,2,5,6-tetrahydropyridine-3-carboxylic acid (79.41 mg, 625 µmol), 6,**7**-dihydro-5*H*-pyrrolo[3,4-*b*]pyridine (75.05 mg, 625 µmol), 1,2-dihydroisoquinoline (81.98 mg, 625 µmol), decahydro-1,8-naphthyridine (87.58 mg, 625 µmol), 6-oxo-1,2,5,6-tetrahydropyridine-3-carboxylic acid (88.15 mg, 625 µmol), or 3-hydroxypiperidin-2-one (71.95 mg, 625 µmol) were added. The mixture was refluxed for 72 h.

After cooling, the product (either **Pro-R3**, **Pro-R6**, **Pro-R7**, **Pro-R8**, **Pro-R9,** and **Pro-R12**, 112.25 mg, 95%, 104.67 mg, 92 %, 109.80 mg, 91 %, 120.00 mg, 95 %, 119.28 mg, 94 %, 105.13 mg, 95%, respectively) crystallized as white solids from the resulting solution.

*1-(2-chloroethyl)-1,2,5,6-tetrahydropyridine-3-carboxylic acid (**Pro-R3**).* ^{1}H NMR (500 MHz, DMSO-*d*$_6$) δ 13.18 (s, 1H), 7.31 (s, 1H), 4.19 (s, 1H), 3.78 – 3.61 (m, 2H), 3.29 (s, 1H), 2.85 (s, 1H), 2.75 (s, 1H), 2.63 (s, 1H), 2.54 – 2.41 (m, 7H), 2.34 – 2.14 (m, 2H). ^{13}C NMR (125 MHz, DMSO-*d*$_6$) δ 166.47 (s), 142.15 (s), 126.73 (s), 56.04 (s), 53.79 (s), 48.52 (s), 40.27 (s), 40.08 – 39.96 (m), 26.10 (s). ^{15}N NMR (51 MHz, DMSO-*d*$_6$) δ -330.10 (s). ^{17}O NMR (68 MHz, DMSO-*d*$_6$) δ 337.20 (s), 253.90 (s), 13.00 (s). MS m/z calcd. for $C_8H_{12}ClNO_2$: [M + H]$^+$; 189.06, found 182.64.

*6-(2-chloroethyl)-6,7-dihydro-5H-pyrrolo[3,4-b]pyridine (**Pro-R6**).* ^{1}H NMR (500 MHz, DMSO-*d*$_6$) δ 8.30 (s, 5H), 7.76 (s, 5H), 7.28 (s, 4H), 4.17 (d, *J* = 1.2 Hz, 10H), 3.71 – 3.67 (m, 8H), 3.58 (s, 5H), 3.26 (s, 5H), 2.89 (s, 4H), 2.61 (s, 4H), 2.57 – 2.43 (m, 30H). ^{13}C NMR (125 MHz, DMSO-*d*$_6$) δ 162.61 (s), 146.33 (s), 130.94 (s), 126.54 (s), 121.84 (s), 63.36 (s), 61.31 (s), 55.47 (s), 40.27 (s), 40.17 – 39.96 (m). ^{15}N NMR (51 MHz, DMSO-*d*$_6$) δ -47.00 (s), -315.50 (s) MS m/z calcd. for $C_9H_{11}ClN_2$: [M + H]$^+$; 182.06, found 182.65.

*2-(2-chloroethyl)-1,2-dihydroisoquinoline (**Pro-R7**).* ^{1}H NMR (500 MHz, DMSO-*d*$_6$) δ 7.41 (d, *J* = 6.3 Hz, 2H), 7.33 (d, *J* = 66.8 Hz, 8H), 7.12 (d, *J* = 4.5 Hz, 10H), 7.07 (s, 5H), 5.33 (s, 5H), 4.44 (d, *J* = 11.7 Hz, 10H), 3.70 – 3.66 (m, 8H), 3.57 (s, 6H), 2.79 (s, 4H), 2.57 – 2.43 (m, 30H). ^{13}C NMR (125 MHz, DMSO-*d*$_6$) δ 140.38 (s), 133.01 (s), 132.42 (s), 128.16 (s), 127.21 (s), 126.58 (s), 125.59 (s), 102.97 (s), 52.74 (s), 51.86 (s), 41.07 (s), 40.17 – 39.96 (m). ^{15}N NMR (51 MHz, DMSO-*d*$_6$) δ -287.10 (s). -287.10 (s). MS m/z calcd. for $C_{11}H_{11}ClN$: [M + H]$^+$ 193.07, found 193.67.

*1-(2-chloroethyl)decahydro-1,8-naphthyridine (**Pro-R8**).* ^{1}H NMR (500 MHz, DMSO-*d*$_6$) δ 3.75 – 3.47 (m, 23H), 2.88 (dd, *J* = 38.7, 38.0 Hz, 30H), 2.74 (d, *J* = 26.5 Hz, 17H), 2.57 – 2.43 (m, 45H), 2.27 (s, 8H), 1.86 – 1.25 (m, 57H), 1.26 (d, *J* = 7.3 Hz, 3H), 1.22 (d, *J* = 21.0 Hz, 16H). ^{13}C NMR (125 MHz, DMSO-*d*$_6$) δ 65.38 (s), 54.16 (s), 50.35 (s), 43.97 (s), 41.01 (s), 40.17 – 39.96 (m), 38.16 (s), 27.90 (d, *J* = 0.5 Hz), 24.53 (s), 23.23 (s). ^{15}N NMR (51 MHz, DMSO-*d*$_6$) δ -326.40 (s), -334.90 (s). MS m/z calcd. for $C_{10}H_{19}ClN_2$: [M + H]$^+$; 202.12, found 202.72.

*1-(2-chloroethyl)-6-oxo-1,2,5,6-tetrahydropyridine-3-carboxylic acid (**Pro-R9**).* ^{1}H NMR (500 MHz, DMSO-*d*$_6$) δ 15.83 (s, 2H), 7.44 (s, 2H), 4.50 (s, 2H), 4.04 (s, 2H), 3.75 (s, 2H), 3.64 – 3.60 (m, 3H), 3.37 (s, 2H), 2.94 – 2.76 (m, 4H), 2.57 – 2.43 (m, 12H). ^{13}C NMR (125 MHz, DMSO-*d*$_6$) δ 167.89 (s), 166.47 (s), 130.74 (s), 124.96 (s), 49.61 (s), 42.77 (s), 41.63 (s), 40.17 – 39.96 (m), 33.68 (s). ^{15}N NMR (51 MHz, DMSO-*d*$_6$) δ -287.10 (s). MS m/z calcd. for $C_8H_{10}ClNO_3$: [M + H]$^+$; 203.03, found 202.62. ^{17}O NMR (68 MHz, DMSO-*d*$_6$) δ 346.00 (s), 337.20 (s), 253.90 (s), 13.00 (s).

*1-(2-chloroethyl)-3-hydroxypiperidin-2-one (**Pro-R12**).* ^{1}H NMR (500 MHz, DMSO-*d*$_6$) δ 5.44 (s, 35H), 4.44 (s, 36H), 3.56 (dd, *J* = 47.9, 36.1 Hz, 145H), 3.42 – 3.41 (m, 3H), 3.29 (d, *J* = 50.4 Hz, 72H), 3.22 – 3.11 (m, 6H), 2.57 – 2.43 (m, 212H), 2.16 (s, 38H), 1.74 (s, 51H), 1.67 (d, *J* = 1.6 Hz, 59H). ^{13}C NMR (125 MHz, DMSO-*d*$_6$) δ 175.82 (s), 67.27 (s), 48.88 (s), 45.56 (s), 41.63 (s), 40.17 – 39.96 (m), 26.52 (s), 22.42 (s). ^{15}N NMR (51 MHz, DMSO-*d*$_6$) δ -257.80 (s). ^{17}O NMR (68 MHz, DMSO-*d*$_6$) δ 346.00 (s), 32.30 (s), 13.00 (s). MS m/z calcd. for $C_7H_{12}ClNO_2$: [M + H]$^+$; 177.06, found 177.63.

*General procedure for the synthesis of **Pro-3DPQ-1** to **Pro-3DPQ-12***

An amount of **BFA-D4** (25 mg, 45 μmol) was dissolved in 10 mL of hot ethanol. Then, potassium carbonate (6.20 mg, 45 μmol) and an equimolar concentration of either **Pro-R1** (2-chloro-*N*-methylethanamine, 4.18 mg, 45 μmol), **Pro-R2** (2-chloro-*N*,*N*-dimethylethanamine, 4.82 mg, 45 μmol),

Pro-R3 (1-(2-chloroethyl)-1,2,5,6-tetrahydropyridine-3-carboxylic acid, 8.51 mg, 45 μmol, see Supplementary Materials for synthesis details), **Pro-R4** (2-chloroethanesulfonic acid, 6.48 mg, 45 μmol), **Pro-R5** (1-(2-chloroethyl)pyrrolidine-2,5-dione, 7.25 mg, 45 μmol), **Pro-R6** (6-(2-chloroethyl)-6,7-dihydro-5*H*-pyrrolo[3,4-b]pyridine, 8.19 mg, 45 μmol, see Supplementary Materials for synthesis details), **Pro-R7** (2-(2-chloroethyl)-1,2-dihydroisoquinoline, 8.69 mg, 45 μmol, see Supplementary Materials for synthesis details), **Pro-R8** (1-(2-chloroethyl)decahydro-1,8-naphthyridine, 9.10 mg, 45 μmol, see Supplementary Materials for synthesis details), **Pro-R9** (*1-(2-chloroethyl)-6-oxo-1,2,5,6-tetrahydropyridine-3-carboxylic acid*, 9,13 mg, 45 μmol, see Supplementary Materials for synthesis details), **Pro-R10** (1-(2-chloroethyl)piperidine, (6.62 mg, 45 μmol), **Pro-R11** (1-(2-chloroethyl)pyrrolidine, (5.99 mg, 45 μmol), or **Pro-R12** (1-(2-chloroethyl)-3-hydroxypiperidin-2-one, 6.62 mg, 45 μmol) was added. The mixture was refluxed for 2 h. After cooling, the inorganic residue was filtered off and the product (either **Pro-3DPQ-1**, **Pro-3DPQ-2**, **Pro-3DPQ-3**, **Pro-3DPQ-4**, **Pro-3DPQ-5**, **Pro-3DPQ-6**, **Pro-3DPQ-7**, **Pro-3DPQ-8**, **Pro-3DPQ-9**, **Pro-3DPQ-10**, **Pro-3DPQ-11**, or **Pro-3DPQ-12**, respectively) crystallized from the resulting solution.

*(1R,2E,6R,10E,11aS,13S,14aR)-13-((tert-butyldimethylsilyl)oxy)-6-methoxy-4-oxo-4,6,7,8,9,11a,12,13,14,14a-decahydro-1H-cyclopenta[f][1]oxacyclotridecin-1-yl 3-acetyl-4-(2-(methylamino)ethoxy)benzoate (**Pro-3DPQ-1**)*. Pale yellow solid (25.70 mg, 95%). ^{1}H NMR (500 MHz, DMSO-d_6) 8.32 (s, 9H), 8.09 (s, 9H), 7.22 (s, 8H), 7.07 (s, 10H), 6.37 (s, 9H), 6.04 (s, 9H), 5.47 (d, *J* = 8.7 Hz, 18H), 5.15 (s, 9H), 4.09 – 4.05 (m, 14H), 3.61 (s, 8H), 3.35 – 3.21 (m, 26H), 3.05 (s, 8H), 3.01 (s, 8H), 2.62 – 2.46 (m, 81H), 2.52 – 2.46 (m, 52H), 2.52 – 2.37 (m, 78H), 2.18 (d, *J* = 18.4 Hz, 15H), 2.09 (s, 6H), 2.02 (d, *J* = 14.7 Hz, 20H), 1.97 – 1.77 (m, 28H), 1.65 (dd, *J* = 66.3, 24.0 Hz, 35H), 1.58 (d, *J* = 4.8 Hz, 19H), 1.60 – 1.42 (m, 25H), 1.25 (s, 1H), 1.13 – 0.99 (m, 78H), 0.29 – 0.15 (m, 52H). ^{13}C NMR (125 MHz, DMSO-d_6) δ 200.54 (s), 166.89 (s), 164.94 (s), 161.47 (s), 143.04 (s), 134.56 (s), 133.17 (s), 131.22 (s), 130.82 (s), 127.47 (s), 126.30 (s), 121.22 (s), 115.21 (s), 101.68 (s), 79.97 (s), 72.42 (s), 67.28 (s), 55.30 (s), 50.93 (s), 49.13 (s), 48.01 (s), 40.17 – 39.96 (m), 39.29 (s), 36.61 (d, *J* = 22.7 Hz), 36.51 – 36.31 (m), 33.05 (s), 32.41 (s), 28.28 (s), 25.76 – 25.55 (m), 22.92 (s), 18.59 (s), -2.89 – -3.29 (m). ^{15}N NMR (51 MHz, DMSO-d_6) δ -352.00 (s). ^{17}O NMR (68 MHz, DMSO-d_6) δ 488.00 (s), 338.90 (s), 332.00 (s), 130.60 (s), 78.00 (s), 31.40 (s), 23.00 (s), 13.00 (s). MS m/z calcd. for $C_{34}H_{51}NO_8Si$: [M + H]$^+$ m/z: 629.34, found 629.86.

(1R,2E,6R,10E,11aS,13S,14aR)-13-((tert-butyldimethylsilyl)oxy)-6-methoxy-4-oxo-4,6,7,8,9,11a,12,13,14,14a-decahydro-1H-cyclopenta[f][1]oxacyclotridecin-1-yl 3-acetyl-4-(2-(dimethylamino)ethoxy)benzoate (**Pro-3DPQ-2**). Pale yellow solid (26.30 mg, 95%). ^{1}H NMR (500 MHz, DMSO-d_6) δ 8.57 (s, 6H), 8.16 (s, 6H), 7.21 (s, 6H), 7.07 (s, 6H), 6.37 (s, 6H), 6.04 (s, 6H), 5.48 (s, 6H), 5.31 (s, 6H), 5.15 (s, 6H), 4.21 – 4.05 (m, 12H), 3.61 (s, 6H), 3.36 – 3.22 (m, 18H), 2.87 – 2.71 (m, 12H), 2.64 – 2.50 (m, 38H), 2.50 – 2.43 (m, 16H), 2.41 – 2.27 (m, 36H), 2.18 (d, *J* = 3.5 Hz, 1H), 2.16 (s, 7H), 2.20 – 1.77 (m, 45H), 1.77 – 1.61 (m, 13H), 1.61 – 1.60 (m, 3H), 1.53 (d, *J* = 49.0 Hz, 13H), 1.11 – 0.97 (m, 53H), 0.15 – 0.01 (m, 35H). ^{13}C NMR (125 MHz, DMSO-d_6) δ 200.54 (s), 166.89 (s), 164.94 (s), 161.47 (s), 143.04 (s), 134.56 (s), 133.17 (s), 131.22 (s), 130.82 (s), 127.47 (s), 126.30 (s), 121.22 (s), 115.21 (s), 101.68 (s), 79.97 (s), 72.42 (s), 68.31 (s), 58.44 (s), 55.30 (s), 49.13 (s), 48.01 (s), 45.66 – 45.35 (m), 40.17 – 39.96 (m), 39.29 (s), 36.70 (s), 33.05 (s), 32.41 (s), 28.28 (s), 25.76 – 25.55 (m), 22.92 (s), 18.59 (s), -2.89 – -3.29 (m). ^{15}N NMR (51 MHz, DMSO-d_6) δ -355.20 (s). ^{17}O NMR (68 MHz, DMSO-d_6) δ 488.00 (s), 338.90 (s), 332.00 (s), 130.60 (s), 78.00 (s), 31.40 (s), 23.00 (s), 13.00 (s). MS m/z calcd. for $C_{35}H_{53}NO_8Si$: [M + H]$^+$ m/z: 643.35, found 643.88.

1-(2-(2-acetyl-4-((((1R,2E,6R,10E,11aS,13S,14aR)-13-((tert-butyldimethylsilyl)oxy)-6-methoxy-4-oxo-4,6,7,8,9,11a,12,13,14,14a-decahydro-1H-cyclopenta[f][1]oxacyclotridecin-1-yl)oxy)carbonyl)phenoxy)ethyl)-1,2,5,6-tetrahydropyridine-3-carboxylic acid (**Pro-3DPQ-3**). Pale yellow solid (29.70 mg, 91%). ^{1}H NMR (500 MHz, DMSO-d_6) δ 16.81 (s, 23H), 8.50 (s, 23H), 7.89 (s, 23H), 7.31 (s, 24H), 7.18 (s, 21H), 7.07 (s, 25H), 6.37 (s, 23H), 6.04 (s, 24H), 5.48 (s, 19H), 5.44 (s, 18H), 5.15 (s, 23H), 4.25 (s, 23H), 4.12 – 3.96 (m, 46H), 3.61 (s, 25H), 3.44 (s, 23H), 3.41 – 3.27 (m, 69H), 2.89 – 2.68 (m, 71H), 2.65 – 2.54 (m, 73H), 2.54 – 2.43 (m, 158H), 2.36 – 2.20 (m, 50H), 2.16 (d, *J* = 1.4 Hz, 49H), 2.20 – 1.93 (m, 151H), 1.72 (dd, *J* = 59.9, 55.2 Hz, 108H), 1.59

(d, *J* = 1.8 Hz, 5H), 1.57 (s, 20H), 1.52 (d, *J* = 49.0 Hz, 49H), 1.10 – 0.96 (m, 204H), 0.25 – 0.11 (m, 136H). ^{13}C NMR (125 MHz, DMSO-d_6) δ 200.54 (s), 166.89 (s), 166.47 (s), 164.94 (s), 161.47 (s), 143.04 (s), 142.15 (s), 134.56 (s), 133.17 (s), 131.22 (s), 130.82 (s), 127.47 (s), 126.73 (s), 126.30 (s), 121.22 (s), 115.21 (s), 101.68 (s), 79.97 (s), 72.42 (s), 67.45 (s), 55.30 (s), 54.64 (s), 53.79 (s), 49.13 (s), 48.52 (s), 48.01 (s), 40.17 – 39.96 (m), 39.29 (s), 36.70 (s), 33.05 (s), 32.41 (s), 28.28 (s), 26.10 (s), 25.76 – 25.55 (m), 22.92 (s), 18.59 (s), -2.89 – -3.29 (m). ^{15}N NMR (51 MHz, DMSO-d_6) δ -330.10 (s). ^{17}O NMR (68 MHz, DMSO-d_6) δ 488.00 (s), 338.90 (s), 337.20 (s), 332.00 (s), 253.90 (s), 130.60 (s), 78.00 (s), 31.40 (s), 23.00 (s), 13.00 (s). MS m/z calcd. for $C_{34}H_{48}O_{10}Si$: $[M + H]^+$ m/z: 644.30, found 644.82.

*2-(2-acetyl-4-((((1R,2E,6R,10E,11aS,13S,14aR)-13-((tert-butyldimethylsilyl)oxy)-6-methoxy-4-oxo-4,6,7,8,9,11a,12,13,14,14a-decahydro-1H-cyclopenta[f][1]oxacyclotridecin-1-yl)oxy)carbonyl)phenoxy)ethanesulfonic acid (**Pro-3DPQ-4**).* Pale yellow solid (26.38 mg, 93%). ^{1}H NMR (500 MHz, DMSO-d_6) δ 15.39 (s, 65H), 8.55 (s, 65H), 8.11 (s, 66H), 7.19 (s, 96H), 7.07 (s, 38H), 6.37 (s, 66H), 6.04 (s, 68H), 5.48 (s, 69H), 5.28 (s, 71H), 5.15 (s, 63H), 4.58 – 4.41 (m, 132H), 3.61 (s, 60H), 3.46 – 3.33 (m, 133H), 3.33 – 3.20 (m, 199H), 2.59 – 2.45 (m, 197H), 2.22 (s, 8H), 2.22 – 2.03 (m, 608H), 2.03 – 1.70 (m, 338H), 1.75 (s, 68H), 1.79 – 1.60 (m, 195H), 1.64 (s, 25H), 1.61 (s, 81H), 1.61 (s, 87H), 1.65 – 1.26 (m, 228H), 1.08 – 0.94 (m, 587H), -0.09 – -0.23 (m, 391H). ^{13}C NMR (125 MHz, DMSO-d_6) 204.31 (s), 200.54 (s), 166.89 (s), 164.94 (s), 161.47 (s), 143.04 (s), 134.56 (s), 133.17 (s), 131.22 (s), 130.82 (s), 127.47 (s), 126.30 (s), 121.22 (s), 115.21 (s), 101.68 (s), 79.97 (s), 72.42 (s), 61.70 (s), 55.30 (s), 49.87 (s), 49.13 (s), 48.01 (s), 39.29 (s), 36.70 (s), 33.05 (s), 32.41 (s), 31.16 – 30.76 (m), 28.28 (s), 25.76 – 25.55 (m), 22.92 (s), 18.59 (s), -2.89 – -3.29 (m). ^{17}O NMR (68 MHz, DMSO-d_6) δ 488.00 (s), 338.90 (s), 332.00 (s), 164.65 – 164.13 (m), 150.00 (s), 130.60 (s), 78.00 (s), 31.40 (s), 23.00 (s). MS m/z calcd. for $C_{33}H_{48}O_{11}SSi$: $[M + H]^+$ m/z: 680.27, found 680.88.

*(1R,2E,6R,10E,11aS,13S,14aR)-13-((tert-butyldimethylsilyl)oxy)-6-methoxy-4-oxo-4,6,7,8,9,11a,12,13,14,14a-decahydro-1H-cyclopenta[f][1]oxacyclotridecin-1-yl 3-acetyl-4-(2-(2,5-dioxopyrrolidin-1-yl)ethoxy)benzoate (**Pro-3DPQ-5**).* Yellow solid (26.38 mg, 93%). δ 8.41 (s, 3H), 8.15 (s, 3H), 7.11 (d, *J* = 44.7 Hz, 5H), 7.05 (s, 1H), 6.37 (s, 3H), 6.04 (s, 3H), 5.48 (s, 3H), 5.17 (d, *J* = 22.6 Hz, 6H), 4.26 – 4.11 (m, 6H), 3.72 – 3.50 (m, 9H), 3.32 – 3.18 (m, 9H), 2.85 – 2.71 (m, 12H), 2.57 – 2.39 (m, 27H), 2.00 (s, 3H), 2.39 – 1.70 (m, 29H), 2.01 – 1.70 (m, 15H), 2.01 – 1.56 (m, 20H), 1.67 (s, 3H), 1.65 – 1.59 (m, 1H), 1.62 (d, *J* = 49.2 Hz, 6H), 1.52 (d, *J* = 49.0 Hz, 7H), 1.11 – 0.97 (m, 27H), 0.21 – 0.07 (m, 18H). ^{13}C NMR (125 MHz, DMSO-d_6) δ 200.54 (s), 178.36 – 178.14 (m), 166.89 (s), 164.94 (s), 161.47 (s), 143.04 (s), 134.56 (s), 133.17 (s), 131.22 (s), 130.82 (s), 127.47 (s), 126.30 (s), 121.22 (s), 115.21 (s), 101.68 (s), 79.97 (s), 72.42 (s), 67.06 (s), 55.30 (s), 49.13 (s), 48.01 (s), 40.17 – 39.96 (m), 39.29 (s), 36.70 (s), 36.01 (s), 33.05 (s), 32.41 (s), 28.61 – 28.07 (m), 25.76 – 25.55 (m), 22.92 (s), 18.59 (s), -2.89 – -3.29 (m). ^{15}N NMR (51 MHz, DMSO-d_6) δ -231.40 (s). ^{17}O NMR (68 MHz, DMSO-d_6) δ 488.00 (s), 371.37 – 370.60 (m), 338.90 (s), 332.00 (s), 130.60 (s), 78.00 (s), 31.40 (s), 23.00 (s), 13.00 (s). MS m/z calcd. for $C_{37}H_{51}NO_{10}Si$: $[M + H]^+$ m/z: m/z: 697.33, found 697.89.

*(1R,2E,6R,10E,11aS,13S,14aR)-13-((tert-butyldimethylsilyl)oxy)-6-methoxy-4-oxo-4,6,7,8,9,11a,12,13,14,14a-decahydro-1H-cyclopenta[f][1]oxacyclotridecin-1-yl 4-(2-(5H-pyrrolo[3,4-b]pyridin-6(7H)-yl)ethoxy)-3-acetylbenzoate (**Pro-3DPQ-6**).* Brown solid (29.51 mg, 95%). ^{1}H NMR (500 MHz, DMSO-d_6) δ 8.55 (s, 2H), 8.33 (s, 2H), 8.17 (s, 2H), 7.60 (s, 2H), 7.26 (d, *J* = 2.3 Hz, 4H), 7.07 (s, 2H), 6.37 (s, 2H), 6.04 (s, 2H), 5.49 (d, *J* = 7.3 Hz, 4H), 5.15 (s, 2H), 4.35 (s, 2H), 4.14 – 3.97 (m, 4H), 3.90 (s, 2H), 3.66 (d, *J* = 53.4 Hz, 4H), 3.39 – 3.22 (m, 8H), 2.93 (s, 2H), 2.78 – 2.60 (m, 7H), 2.53 (s, 2H), 2.53 – 2.43 (m, 11H), 2.40 – 1.97 (m, 10H), 2.40 – 1.73 (m, 19H), 2.40 – 1.73 (m, 19H), 1.97 – 1.75 (m, 8H), 1.82 – 1.77 (m, 1H), 1.75 (d, *J* = 1.1 Hz, 3H), 1.75 (d, *J* = 1.1 Hz, 5H), 1.52 (d, *J* = 49.0 Hz, 4H), 1.10 – 0.96 (m, 17H), 0.20 – 0.06 (m, 11H). ^{13}C NMR (125 MHz, DMSO-d_6) δ 200.54 (s), 166.89 (s), 164.94 (s), 162.61 (s), 161.47 (s), 146.33 (s), 143.04 (s), 134.56 (s), 133.17 (s), 131.22 (s), 130.88 (d, *J* = 14.6 Hz), 127.47 (s), 126.54 (s), 126.30 (s), 121.84 (s), 121.22 (s), 115.21 (s), 101.68 (s), 79.97 (s), 72.42 (s), 67.45 (s), 63.36 (s), 61.31 (s), 55.39 (d, *J* = 23.2 Hz), 55.30 – 55.19 (m), 49.13 (s), 48.01 (s), 40.17 – 39.96 (m), 39.29 (s), 36.70 (s), 33.05 (s), 32.41 (s), 28.28 (s), 25.76 – 25.55 (m), 22.92 (s), 18.59 (s), -2.89 – -3.29 (m). ^{15}N NMR (51 MHz, DMSO-d_6) δ -47.00 (s), -315.50 (s). ^{17}O NMR (68 MHz,

DMSO-d_6) δ 488.00 (s), 338.90 (s), 332.00 (s), 130.60 (s), 78.00 (s), 31.40 (s), 23.00 (s), 13.00 (s). MS m/z calcd. for $C_{40}H_{54}N_2O_8Si$: [M + H]$^+$ m/z: 718.36, found 718.95.

*(2E,10E,11aS,14aR)-13-((tert-butyldimethylsilyl)oxy)-6-methoxy-4-oxo-4,6,7,8,9,11a,12,13,14,14a-decahydro-1H-cyclopenta[f][1]oxacyclotridecin-1-yl 4-(2-(isoquinolin-2(1H)-yl)ethoxy)-3-methylbenzoate (**Pro-3DPQ-7**).* Brown solid (29.99 mg, 95%). ^{1}H NMR (500 MHz, DMSO-d_6) δ 8.54 (s, 6H), 8.13 (s, 6H), 7.25 – 7.03 (m, 34H), 7.25 – 6.90 (m, 41H), 7.28 – 6.90 (m, 41H), 7.01 (s, 7H), 6.37 (s, 6H), 6.04 (s, 6H), 5.48 (s, 6H), 5.32 (d, *J* = 10.1 Hz, 12H), 5.15 (s, 6H), 4.44 (d, *J* = 27.8 Hz, 11H), 4.25 – 4.08 (m, 12H), 3.61 (s, 5H), 3.38 (s, 6H), 3.36 – 3.27 (m, 17H), 2.72 (s, 6H), 2.68 – 2.44 (m, 52H), 2.68 – 2.23 (m, 54H), 2.68 – 2.11 (m, 66H), 2.68 – 2.02 (m, 72H), 2.68 – 1.95 (m, 84H), 1.78 (dd, *J* = 61.9, 51.5 Hz, 27H), 1.59 (d, *J* = 1.6 Hz, 2H), 1.52 (d, *J* = 49.0 Hz, 13H), 1.08 – 0.94 (m, 51H), -0.06 – -0.20 (m, 34H). ^{13}C NMR (125 MHz, DMSO-d_6) δ 200.54 (s), 166.89 (s), 164.94 (s), 161.47 (s), 143.04 (s), 140.38 (s), 134.56 (s), 133.09 (d, *J* = 20.1 Hz), 132.97 – 132.81 (m), 132.42 (s), 131.22 (s), 130.82 (s), 128.16 (s), 127.47 (s), 127.21 (s), 126.58 (s), 126.30 (s), 125.59 (s), 121.22 (s), 115.21 (s), 102.97 (s), 101.68 (s), 79.97 (s), 72.42 (s), 67.17 (s), 55.30 (s), 51.86 (s), 50.60 (s), 49.13 (s), 48.01 (s), 40.17 – 39.96 (m), 39.29 (s), 36.70 (s), 33.05 (s), 32.41 (s), 28.28 (s), 25.76 – 25.55 (m), 22.92 (s), 18.59 (s), -2.89 – -3.29 (m). ^{15}N NMR (51 MHz, DMSO-d_6) δ -287.10 (s). ^{17}O NMR (68 MHz, DMSO-d_6) δ 488.00 (s), 338.90 (s), 332.00 (s), 130.60 (s), 78.00 (s), 31.40 (s), 23.00 (s), 13.00 (s). MS m/z calcd. for $C_{41}H_{55}NO_7Si$: [M + H]$^+$ m/z: 701.37, found 701.37.

*(1R,2E,6R,10E,11aS,13S,14aR)-13-((tert-butyldimethylsilyl)oxy)-6-methoxy-4-oxo-4,6,7,8,9,11a,12,13,14,14a-decahydro-1H-cyclopenta[f][1]oxacyclotridecin-1-yl 3-acetyl-4-(2-(octahydro-1,8-naphthyridin-1(2H)-yl)ethoxy)benzoate (**Pro-3DPQ-8**).* Pale yellow solid (30.05 mg, 94%). ^{1}H NMR (500 MHz, DMSO-d_6) δ 8.53 (s, 8H), 8.10 (s, 8H), 7.23 (s, 8H), 7.07 (s, 8H), 6.37 (s, 8H), 6.04 (s, 8H), 5.60 (s, 12H), 5.48 (s, 5H), 5.15 (s, 8H), 4.06 – 4.02 (m, 13H), 3.61 (s, 8H), 3.39 (s, 7H), 3.37 – 3.27 (m, 24H), 3.10 (s, 9H), 2.90 (s, 9H), 2.79 (s, 1H), 2.72 (d, *J* = 51.7 Hz, 16H), 2.60 (s, 6H), 2.58 – 2.39 (m, 81H), 2.13 (dd, *J* = 23.4, 11.6 Hz, 32H), 2.01 (d, *J* = 6.2 Hz, 13H), 1.95 (s, 10H), 1.93 (s, 1H), 1.93 – 1.31 (m, 105H), 1.51 – 1.28 (m, 23H), 1.53 – 1.31 (m, 28H), 1.28 – 0.97 (m, 88H), 0.17 – 0.03 (m, 47H). ^{13}C NMR (125 MHz, DMSO-d_6) 200.54 (s), 166.89 (s), 164.94 (s), 161.47 (s), 143.04 (s), 134.56 (s), 133.17 (s), 131.22 (s), 130.82 (s), 127.47 (s), 126.30 (s), 121.22 (s), 115.21 (s), 101.68 (s), 79.97 (s), 72.42 (s), 66.80 (s), 65.38 (s), 55.30 (s), 52.88 (s), 50.51 (s), 49.13 (s), 48.01 (s), 43.97 (s), 40.17 – 39.96 (m), 39.29 (s), 39.03 (s), 36.70 (s), 33.05 (s), 32.41 (s), 28.28 (s), 27.79 (d, *J* = 0.5 Hz), 25.76 – 25.55 (m), 24.54 (s), 23.25 (s), 22.92 (s), 18.59 (s), -2.89 – -3.29 (m). ^{15}N NMR (51 MHz, DMSO-d_6) δ -326.40 (s), -334.90 (s). ^{17}O NMR (68 MHz, DMSO-d_6) δ 488.00 (s), 338.90 (s), 332.00 (s), 130.60 (s), 78.00 (s), 31.40 (s), 23.00 (s), 13.00 (s). MS m/z calcd. for $C_{41}H_{62}N_2O_8Si$: [M + H]$^+$ m/z: 738.43, found 739.03.

*1-(2-(2-acetyl-4-((((1R,2E,6R,10E,11aS,13S,14aR)-13-((tert-butyldimethylsilyl)oxy)-6-methoxy-4-oxo-4,6,7,8,9,11a,12,13,14,14a-decahydro-1H-cyclopenta[f][1]oxacyclotridecin-1-yl)oxy)carbonyl)phenoxy)ethyl)-6-oxo-1,2,5,6-tetrahydropyridine-3-carboxylic acid (**Pro-3DPQ-9**).* Whitish solid (29.94 mg, 90%). ^{1}H NMR (500 MHz, DMSO-d_6) δ 12.86 (s, 3H), 8.47 (s, 3H), 8.11 (s, 3H), 7.45 (s, 3H), 7.13 (d, *J* = 64.3 Hz, 6H), 6.37 (s, 3H), 6.04 (s, 3H), 5.48 (s, 3H), 5.20 (d, *J* = 48.5 Hz, 6H), 4.38 (s, 3H), 4.30 – 4.13 (m, 6H), 4.02 (s, 3H), 3.86 (s, 3H), 3.61 (s, 3H), 3.36 – 3.22 (m, 10H), 3.21 (s, 2H), 2.94 – 2.76 (m, 6H), 2.57 – 2.43 (m, 19H), 2.43 – 2.33 (m, 9H), 2.17 (d, *J* = 14.7 Hz, 5H), 2.09 (s, 2H), 1.97 (dd, *J* = 27.1, 10.2 Hz, 13H), 1.85 – 1.63 (m, 9H), 1.52 (d, *J* = 49.0 Hz, 6H), 1.10 – 0.96 (m, 27H), 0.24 – 0.10 (m, 18H). ^{13}C NMR (125 MHz, DMSO-d_6 δ 200.54 (s), 167.89 (s), 166.89 (s), 166.47 (s), 164.94 (s), 161.47 (s), 143.04 (s), 134.56 (s), 133.17 (s), 131.22 (s), 130.78 (d, *J* = 10.1 Hz), 127.47 (s), 126.30 (s), 124.96 (s), 121.22 (s), 115.21 (s), 101.68 (s), 79.97 (s), 72.42 (s), 66.30 (s), 55.30 (s), 49.13 (s), 48.01 (s), 46.03 (s), 42.77 (s), 40.17 – 39.96 (m), 39.29 (s), 36.70 (s), 33.68 (s), 33.05 (s), 32.41 (s), 28.28 (s), 25.76 – 25.55 (m), 22.92 (s), 18.59 (s), -2.89 – -3.29 (m). ^{15}N NMR (51 MHz, DMSO-d_6) δ -287.10 (s). ^{17}O NMR (68 MHz, DMSO-d_6) δ 488.00 (s), 346.00 (s), 338.90 (s), 337.20 (s), 332.00 (s), 253.90 (s), 130.60 (s), 78.00 (s), 31.40 (s), 23.00 (s), 13.00 (s). MS m/z calcd. for $C_{33}H_{47}NO_{10}Si$: [M + H]$^+$ m/z: 645.30, found 645.81.

*(1R,2E,6R,10E,11aS,13S,14aR)-13-((tert-butyldimethylsilyl)oxy)-6-methoxy-4-oxo-4,6,7,8,9,11a,12,13,14,14a-decahydro-1H-cyclopenta[f][1]oxacyclotridecin-1-yl 3-acetyl-4-(2-(piperidin-1-yl)ethoxy)benzoate (**Pro-3DPQ-10**).* Whitish solid (28.02 mg, 95%).^{1}H NMR (500 MHz, DMSO-d_6) δ 8.40 (s, 47H), 7.99 (s, 48H), 7.14 (s, 45H), 7.07 (s, 41H), 6.37 (s, 48H), 6.04 (s, 49H), 5.48 (s, 49H), 5.23 (s, 26H), 5.15 (s, 71H), 4.16 – 4.00 (m, 95H), 3.61 (s, 43H), 3.36 – 3.22 (m, 141H), 2.82 – 2.65 (m, 101H), 2.65 – 2.59 (m, 80H), 2.59 – 2.42 (m, 538H), 2.42 – 2.35 (m, 10H), 2.15 (d, *J* = 10.2 Hz, 91H), 2.09 (s, 51H), 2.00 (s, 35H), 1.94 (d, *J* = 10.0 Hz, 71H), 1.87 (d, *J* = 2.8 Hz, 30H), 1.80 (d, *J* = 47.8 Hz, 115H), 1.66 (d, *J* = 15.7 Hz, 83H), 1.62 – 1.49 (m, 383H), 1.47 (s, 47H), 1.11 – 0.98 (m, 423H), 0.20 – 0.06 (m, 282H). ^{13}C NMR (125 MHz, DMSO-d_6) δ 200.54 (s), 166.89 (s), 164.94 (s), 161.47 (s), 143.04 (s), 134.56 (s), 133.17 (s), 131.22 (s), 130.82 (s), 127.47 (s), 126.30 (s), 121.22 (s), 115.21 (s), 101.68 (s), 79.97 (s), 72.42 (s), 67.45 (s), 55.30 (s), 55.19 – 54.80 (m), 54.80 – 54.60 (m), 49.13 (s), 48.01 (s), 40.17 – 39.96 (m), 39.29 (s), 36.70 (s), 33.05 (s), 32.41 (s), 28.28 (s), 25.76 – 25.55 (m), 24.68 – 24.37 (m), 23.42 (s), 22.92 (s), 18.59 (s), -2.89 – -3.29 (m). ^{15}N NMR (51 MHz, DMSO-d_6) δ -327.80 (s). ^{17}O NMR (68 MHz, DMSO-d_6) δ 488.00 (s), 338.90 (s), 332.00 (s), 130.60 (s), 78.00 (s), 31.40 (s), 23.00 (s), 13.00 (s). MS m/z calcd. for $C_{38}H_{57}NO_8Si$: [M + H]$^+$ m/z: 683.39, found 683.95.

*(1R,2E,6R,10E,11aS,13S,14aR)-13-((tert-butyldimethylsilyl)oxy)-6-methoxy-4-oxo-4,6,7,8,9,11a,12,13,14,14a-decahydro-1H-cyclopenta[f][1]oxacyclotridecin-1-yl 3-acetyl-4-(2-(pyrrolidin-1-yl)ethoxy)benzoate (**Pro-3DPQ-11**).* Whitish solid (24.42 mg, 95%).^{1}H NMR (500 MHz, DMSO-d_6) δ 8.54 (s, 4H), 8.12 (s, 4H), 7.12 (d, *J* = 45.8 Hz, 7H), 7.05 (s, 1H), 6.37 (s, 4H), 6.04 (s, 4H), 5.81 – 5.11 (m, 12H), 4.17 – 4.01 (m, 8H), 3.61 (s, 4H), 3.42 – 3.28 (m, 12H), 3.00 – 2.79 (m, 8H), 2.79 – 2.63 (m, 8H), 2.60 – 2.42 (m, 35H), 1.97 – 1.77 (m, 13H), 2.39 – 1.22 (m, 73H), 1.98 – 1.68 (m, 22H), 1.77 (d, *J* = 3.3 Hz, 2H), 1.75 (s, 4H), 1.71 (s, 10H), 1.67 – 1.59 (m, 11H), 1.67 – 1.25 (m, 19H), 1.68 – 1.22 (m, 23H), 1.10 – 0.96 (m, 35H), 0.20 – 0.07 (m, 23H). ^{13}C NMR (125 MHz, DMSO-d_6) δ 200.54 (s), 166.89 (s), 164.94 (s), 161.47 (s), 143.04 (s), 134.56 (s), 133.17 (s), 131.22 (s), 130.82 (s), 127.47 (s), 126.30 (s), 121.22 (s), 115.21 (s), 101.68 (s), 79.97 (s), 72.42 (s), 67.45 (s), 55.30 (s), 54.92 (s), 54.26 – 53.95 (m), 49.13 (s), 48.01 (s), 40.17 – 39.96 (m), 39.29 (s), 36.70 (s), 33.05 (s), 32.41 (s), 28.28 (s), 25.76 – 25.55 (m), 25.02 – 24.81 (m), 22.92 (s), 18.59 (s), -2.89 – -3.29 (m). ^{15}N NMR (51 MHz, DMSO-d_6) δ -315.50 (s). ^{17}O NMR (68 MHz, DMSO-d_6) δ 488.00 (s), 338.90 (s), 332.00 (s), 130.60 (s), 78.00 (s), 31.40 (s), 23.00 (s), 13.00 (s). MS m/z calcd. for $C_{37}H_{55}NO_8Si$: [M + H]$^+$ m/z: 669.37, found 669.92.

(1R,2E,6R,10E,11aS,13S,14aR)-13-((tert-butyldimethylsilyl)oxy)-6-methoxy-4-oxo-4,6,7,8,9,11a,12,13,14,14a-decahydro-1H-cyclopenta[f][1]oxacyclotridecin-1-yl 3-acetyl-4-(2-(3-hydroxy-2-oxopiperidin-1-yl)ethoxy)benzoate (**Pro-3DPQ-12**). Whitish solid (28.04 mg, 95%). ^{1}H NMR (500 MHz, DMSO-d_6) δ 8.51 (s, 4H), 8.17 (s, 4H), 7.28 (s, 4H), 7.07 (s, 4H), 6.37 (s, 4H), 6.04 (s, 4H), 5.91 (s, 4H), 5.48 (s, 4H), 5.29 (s, 4H), 5.15 (s, 4H), 4.44 (s, 4H), 4.30 – 4.14 (m, 8H), 3.90 (s, 4H), 3.61 (s, 3H), 3.48 (s, 6H), 3.35 – 3.29 (m, 11H), 3.16 (d, *J* = 46.6 Hz, 8H), 2.60 – 2.43 (m, 36H), 1.97 (dd, *J* = 27.6, 9.7 Hz, 16H), 1.85 (dddd, *J* = 92.6, 88.9, 45.9, 24.9 Hz, 66H), 2.07 – 1.60 (m, 42H), 2.07 – 1.37 (m, 50H), 1.53 (d, *J* = 49.0 Hz, 8H), 1.07 – 0.93 (m, 36H), -0.10 – -0.24 (m, 24H). ^{13}C NMR (125 MHz, DMSO-d_6) δ 200.54 (s), 175.72 (s), 166.89 (s), 164.94 (s), 161.47 (s), 143.04 (s), 134.56 (s), 133.17 (s), 131.22 (s), 130.82 (s), 127.47 (s), 126.30 (s), 121.22 (s), 115.21 (s), 101.68 (s), 79.97 (s), 72.42 (s), 67.87 (s), 66.30 (s), 55.30 (s), 49.13 (s), 48.01 (s), 45.68 (d, *J* = 10.7 Hz), 40.17 – 39.96 (m), 39.29 (s), 36.70 (s), 33.05 (s), 32.41 (s), 28.28 (s), 26.41 (s), 25.76 – 25.55 (m), 22.92 (s), 22.44 (s), 18.59 (s), -2.89 – -3.29 (m). ^{15}N NMR (51 MHz, DMSO-d_6) δ -257.80 (s). ^{17}O NMR (68 MHz, DMSO-d_6) δ 488.00 (s), 346.00 (s), 338.90 (s), 332.00 (s), 130.60 (s), 78.00 (s), 32.30 (s), 31.40 (s), 23.00 (s), 13.00 (s). MS m/z calcd. for $C_{38}H_{55}NO_{10}Si$: [M + H]$^+$ m/z: 713.36, found 713.93.

*General procedure for the synthesis of **3DPQ-1** to **3DPQ-12***

To a solution of either **Pro-3DPQ-1** (20 mg, 33 μmol), **Pro-3DPQ-2** (20.30 mg, 33 μmol), **Pro-3DPQ-3** (23.94 mg, 33 μmol), **Pro-3DPQ-4** (20.80 mg, 33 μmol), **Pro-3DPQ-5** (22.09 mg, 33 μmol), **Pro-3DPQ-6** (22.78 mg, 33 μmol), **Pro-3DPQ-7** (23.15 mg, 33 μmol), **Pro-3DPQ-8** (23.44 mg, 33 μmol), **Pro-3DPQ-9** (24.40 mg, 33 μmol), **Pro-3DPQ-10** (21.63 mg, 33 μmol), **Pro-3DPQ-11** (21.16 mg, 33 μmol), or **Pro-3DPQ-12** (21.63 mg, 33 μmol) in dry dichloromethane at 0°C, 1 M TBAF in THF (100 μL, 0.1 mmol)

was added dropwise over 2 h, followed by the dropwise addition of boron tribromide (2 equiv. per methoxy function) under an argon atmosphere. The resulting mixture was then refluxed for 3 h and poured into an ice/water mixture after it was cooled to room temperature. The aqueous layer was extracted with ethyl acetate. The combined organic layers were washed with brine, dried over anhydrous Na_2SO_4, and then filtered and concentrated *in vacuo*. The resulting products, **3DPQ-1** to **3DPQ-12** were purified by column chromatography (25% EtOAc in Et_2O as eluent).

(1R,2E,6R,10E,11aS,13S,14aR)-6,13-dihydroxy-4-oxo-4,6,7,8,9,11a,12,13,14,14a-decahydro-1H-cyclopenta[f][1]oxacyclotridecin-1-yl 3-acetyl-4-(2-(methylamino)ethoxy)benzoate (**3DPQ-1**). Yellowish solid (13.28 mg, 85%) ^{1}H NMR (500 MHz, DMSO-d_6) δ 8.51 (s, 5H), 8.14 (s, 5H), 7.10 (d, *J* = 34.2 Hz, 10H), 6.67 (s, 5H), 6.04 (s, 5H), 20.00 – 3.86 (m, 66H), 5.48 (s, 4H), 5.41 (s, 4H), 5.15 (s, 5H), 4.97 (s, 5H), 4.05 (s, 1H), 3.61 (s, 5H), 3.02 (d, *J* = 1.4 Hz, 9H), 2.62 – 2.42 (m, 59H), 2.10 (dd, *J* = 37.5, 25.2 Hz, 19H), 1.95 (s, 7H), 1.84 (dd, *J* = 51.4, 21.3 Hz, 18H), 1.73 (s, 4H), 1.62 (d, *J* = 42.7 Hz, 8H), 1.62 (d, *J* = 42.7 Hz, 10H), 1.66 – 1.26 (m, 13H). ^{13}C NMR (125 MHz, DMSO-d_6) δ 200.54 (s), 166.89 (s), 165.46 (s), 161.47 (s), 143.04 (s), 134.56 (s), 133.17 (s), 131.22 (s), 130.82 (s), 127.47 (s), 126.30 (s), 121.22 (s), 115.21 (s), 79.97 (s), 73.67 (s), 67.28 (s), 50.93 (s), 48.76 (s), 47.11 (s), 41.34 (s), 40.17 – 39.96 (m), 38.11 (s), 36.52 (s), 34.91 (s), 32.41 (s), 28.28 (s), 22.77 (s). ^{15}N NMR (51 MHz, DMSO-d_6) δ -352.00 (s).^{17}O NMR (68 MHz, DMSO-d_6) δ 488.00 (s), 338.90 (s), 332.00 (s), 130.60 (s), 78.00 (s), 44.50 (s), 33.40 (s), 13.00 (s). MS m/z calcd. for $C_{27}H_{35}NO_8$: [M + H]$^+$ m/z: m/z: 501.24, found 501.57.

(1R,2E,6R,10E,11aS,13S,14aR)-6,13-dihydroxy-4-oxo-4,6,7,8,9,11a,12,13,14,14a-decahydro-1H-cyclopenta[f][1]oxacyclotridecin-1-yl 3-acetyl-4-(2-(dimethylamino)ethoxy)benzoate (**3DPQ-2**). Yellowish solid (13.66 mg, 85%). ^{1}H NMR (500 MHz, DMSO-d_6) δ 8.57 (s, 6H), 8.12 (s, 6H), 7.22 (s, 6H), 7.07 (s, 6H), 6.67 (s, 6H), 6.04 (s, 6H), 5.48 (s, 6H), 5.23 (s, 5H), 5.15 (s, 7H), 4.14 – 3.95 (m, 18H), 3.61 (d, *J* = 2.5 Hz, 12H), 2.80 – 2.63 (m, 12H), 2.60 – 2.43 (m, 53H), 2.31 – 2.17 (m, 37H), 2.16 (s, 3H), 2.05 (dd, *J* = 26.4, 14.7 Hz, 24H), 1.94 (d, *J* = 10.0 Hz, 8H), 1.75 (d, *J* = 32.5 Hz, 10H), 1.75 (d, *J* = 32.5 Hz, 14H), 1.75 (d, *J* = 32.5 Hz, 15H), 1.66 – 1.24 (m, 16H). ^{13}C NMR (125 MHz, DMSO-d_6) δ 200.54 (s), 166.89 (s), 165.46 (s), 161.47 (s), 143.04 (s), 134.56 (s), 133.17 (s), 131.22 (s), 130.82 (s), 127.47 (s), 126.30 (s), 121.22 (s), 115.21 (s), 79.97 (s), 73.67 (s), 68.31 (s), 58.44 (s), 48.76 (s), 47.11 (s), 45.66 – 45.35 (m), 41.34 (s), 40.17 – 39.96 (m), 38.11 (s), 34.91 (s), 32.41 (s), 28.28 (s), 22.77 (s). ^{15}N NMR (51 MHz, DMSO-d_6) δ -355.20 (s). ^{17}O NMR (68 MHz, DMSO-d_6) δ 488.00 (s), 338.90 (s), 332.00 (s), 130.60 (s), 78.00 (s), 44.50 (s), 33.40 (s), 13.00 (s). MS m/z calcd. for $C_{28}H_{37}NO_8$: [M + H]$^+$ m/z: 515.25, found 515.60.

1-(2-(2-acetyl-4-((((2E,6R,10E,11aS,13S,14aR)-6,13-dihydroxy-4-oxo-4,6,7,8,9,11a,12,13,14,14a-decahydro-1H-cyclopenta[f][1]oxacyclotridecin-1-yl)oxy)carbonyl)phenoxy)ethyl)-1,2,5,6-tetrahydropyridine-3-carboxylic acid (**3DPQ-3**). Yellowish solid (16.75 mg, 85%). ^{1}H NMR (500 MHz, DMSO-d_6) δ 13.14 (s, 2H), 8.56 (s, 2H), 8.16 (s, 2H), 7.28 (d, *J* = 19.1 Hz, 4H), 7.07 (s, 2H), 6.67 (s, 2H), 6.04 (s, 2H), 5.48 (s, 2H), 5.19 (d, *J* = 44.9 Hz, 4H), 4.00 (dd, *J* = 29.8, 5.5 Hz, 8H), 3.61 (d, *J* = 4.5 Hz, 4H), 2.94 (d, *J* = 15.9 Hz, 4H), 2.80 (s, 2H), 2.63 (s, 2H), 2.60 – 2.43 (m, 21H), 2.33 – 2.06 (m, 12H), 2.11 (d, *J* = 3.2 Hz, 1H), 2.14 – 2.06 (m, 6H), 2.13 – 1.92 (m, 12H), 1.72 (d, *J* = 61.2 Hz, 5H), 1.62 (d, *J* = 10.2 Hz, 1H), 1.64 – 1.41 (m, 7H). ^{13}C NMR (125 MHz, DMSO-d_6) δ 200.54 (s), 166.89 (s), 166.47 (s), 165.46 (s), 161.47 (s), 143.04 (s), 142.15 (s), 134.56 (s), 133.17 (s), 131.22 (s), 130.82 (s), 127.47 (s), 126.73 (s), 126.30 (s), 121.22 (s), 115.21 (s), 79.97 (s), 73.67 (s), 67.45 (s), 54.64 (s), 53.79 (s), 48.76 (s), 48.52 (s), 47.11 (s), 41.34 (s), 40.17 – 39.96 (m), 38.11 (s), 34.91 (s), 32.41 (s), 28.28 (s), 26.10 (s), 22.77 (s). ^{15}N NMR (51 MHz, DMSO-d_6) δ -330.10 (s). ^{17}O NMR (68 MHz, DMSO-d_6) δ 488.00 (s), 338.90 (s), 337.20 (s), 332.00 (s), 253.90 (s), 130.60 (s), 78.00 (s), 44.50 (s), 33.40 (s), 13.00 (s). MS m/z calcd. for $C_{27}H_{32}O_{10}$: [M + H]$^+$ m/z: 516.20, found 516.54.

2-(2-acetyl-4-((((1R,2E,6R,10E,11aS,13S,14aR)-6,13-dihydroxy-4-oxo-4,6,7,8,9,11a,12,13,14,14a-decahydro-1H-cyclopenta[f][1]oxacyclotridecin-1-yl)oxy)carbonyl)phenoxy)ethanesulfonic acid (**3DPQ-4**). Yellowish solid (14.10 mg, 85%). ^{1}H NMR (500 MHz, DMSO-d_6) δ 14.97 (s, 26H), 8.54 (s, 26H), 8.11 (s, 26H), 7.18 (s, 37H), 7.07 (s, 16H), 6.67 (s, 26H), 6.04 (s, 27H), 5.48 (s, 27H), 5.19 (d, *J* = 42.2 Hz, 49H), 5.13 – 5.06 (m, 4H), 4.55 – 4.38 (m, 52H), 3.98 (s, 26H), 3.61 (d, *J* = 4.5 Hz, 55H), 3.39 – 3.27 (m, 51H), 2.57 – 2.43 (m, 232H),

2.16 (s, 17H), 2.12 (d, *J* = 4.0 Hz, 6H), 2.12 – 1.92 (m, 150H), 1.72 (d, *J* = 65.6 Hz, 55H), 1.63 – 1.36 (m, 85H). ^{13}C NMR (125 MHz, DMSO-*d*$_6$) δ 200.54 (s), 166.89 (s), 165.46 (s), 161.47 (s), 143.04 (s), 134.56 (s), 133.17 (s), 131.22 (s), 130.82 (s), 127.47 (s), 126.30 (s), 121.22 (s), 115.21 (s), 79.97 (s), 73.67 (s), 61.70 (s), 49.87 (s), 48.76 (s), 47.11 (s), 41.34 (s), 40.17 – 39.96 (m), 38.11 (s), 34.91 (s), 32.41 (s), 28.28 (s), 22.77 (s). ^{17}O NMR (68 MHz, DMSO-*d*$_6$) 488.00 (s), 338.90 (s), 332.00 (s), 164.65 – 164.13 (m), 150.00 (s), 130.60 (s), 78.00 (s), 44.50 (s), 33.40 (s), 13.00 (s). MS m/z calcd. for $C_{26}H_{32}O_{11}S$: [M + H]$^+$ m/z: 552.17, found 552.59.

(1R,2E,6R,10E,11aS,13S,14aR)-6,13-dihydroxy-4-oxo-4,6,7,8,9,11a,12,13,14,14a-decahydro-1H-cyclopenta[f][1]oxacyclotridecin-1-yl 3-acetyl-4-(2-(2,5-dioxopyrrolidin-1-yl)ethoxy)benzoate (**3DPQ-5**). Yellowish solid (15.18 mg, 85%) ^{1}H NMR (500 MHz, DMSO-*d*$_6$) δ 8.40 (s, 3H), 8.10 (s, 3H), 7.14 (d, *J* = 65.0 Hz, 6H), 6.67 (s, 3H), 6.04 (s, 3H), 5.48 (s, 3H), 5.19 (d, *J* = 37.9 Hz, 6H), 4.21 – 4.06 (m, 6H), 3.99 (s, 3H), 3.60 (dd, *J* = 23.7, 9.2 Hz, 12H), 2.87 – 2.73 (m, 12H), 2.52 (s, 1H), 2.52 – 2.37 (m, 26H), 2.16 (s, 2H), 2.12 (d, *J* = 6.8 Hz, 1H), 2.13 – 1.92 (m, 17H), 1.72 (d, *J* = 60.0 Hz, 7H), 1.61 – 1.32 (m, 9H). ^{13}C NMR (125 MHz, DMSO-*d*$_6$) δ 200.54 (s), 178.36 – 178.14 (m), 166.89 (s), 165.46 (s), 161.47 (s), 143.04 (s), 134.56 (s), 133.17 (s), 131.22 (s), 130.82 (s), 127.47 (s), 126.30 (s), 121.22 (s), 115.21 (s), 79.97 (s), 73.67 (s), 67.06 (s), 48.76 (s), 47.11 (s), 41.34 (s), 40.17 – 39.96 (m), 38.11 (s), 36.01 (s), 34.91 (s), 32.41 (s), 28.61 – 28.07 (m), 22.77 (s). ^{15}N NMR (51 MHz, DMSO-*d*$_6$) δ -231.40 (s). ^{17}O NMR (68 MHz, DMSO-*d*$_6$) δ 488.00 (s), 371.37 – 370.60 (m), 338.90 (s), 332.00 (s), 130.60 (s), 78.00 (s), 44.50 (s), 33.40 (s), 13.00 (s). MS m/z calcd. for $C_{30}H_{35}NO_{10}$: [M + H]$^+$ m/z: 569.23, found 569.60.

(1R,2E,6R,10E,11aS,13S,14aR)-6,13-dihydroxy-4-oxo-4,6,7,8,9,11a,12,13,14,14a-decahydro-1H-cyclopenta[f][1]oxacyclotridecin-1-yl 4-(2-(5H-pyrrolo[3,4-b]pyridin-6(7H)-yl)ethoxy)-3-acetylbenzoate (**3DPQ-6**). Pale brown solid (15.77 mg, 85%). ^{1}H NMR (500 MHz, DMSO-*d*$_6$) δ 8.50 (s, 5H), 8.30 (s, 5H), 8.11 (s, 5H), 7.59 (s, 5H), 7.34 – 7.16 (m, 10H), 7.07 (s, 5H), 6.67 (s, 5H), 6.04 (s, 5H), 5.48 (s, 5H), 5.26 (s, 1H), 5.19 (d, *J* = 41.1 Hz, 9H), 4.18 – 3.98 (m, 20H), 3.88 (s, 5H), 3.73 (s, 1H), 3.73 – 3.53 (m, 14H), 3.29 (s, 5H), 2.93 (s, 4H), 2.81 (s, 4H), 2.65 – 2.50 (m, 29H), 2.50 – 2.43 (m, 15H), 2.16 (s, 3H), 2.11 (s, 1H), 2.03 (dd, *J* = 40.6, 29.4 Hz, 20H), 1.93 (s, 4H), 1.92 – 1.35 (m, 32H), 1.52 (d, *J* = 49.0 Hz, 11H), 1.52 (d, *J* = 49.0 Hz, 9H). ^{13}C NMR (125 MHz, DMSO-*d*$_6$) δ 200.54 (s), 166.89 (s), 165.46 (s), 162.61 (s), 161.47 (s), 146.33 (s), 143.04 (s), 134.56 (s), 133.17 (s), 131.22 (s), 130.88 (d, *J* = 14.6 Hz), 127.47 (s), 126.54 (s), 126.30 (s), 121.84 (s), 121.22 (s), 115.21 (s), 79.97 (s), 73.67 (s), 67.45 (s), 63.36 (s), 61.31 (s), 55.49 (s), 48.76 (s), 47.11 (s), 41.34 (s), 40.17 – 39.96 (m), 38.11 (s), 34.91 (s), 32.41 (s), 28.28 (s), 22.77 (s). ^{15}N NMR (51 MHz, DMSO-*d*$_6$) δ -47.00 (s), -315.50 (s). ^{17}O NMR (68 MHz, DMSO-*d*$_6$) δ 488.00 (s), 338.90 (s), 332.00 (s), 130.60 (s), 78.00 (s), 44.50 (s), 33.40 (s), 13.00 (s). MS m/z calcd. for $C_{33}H_{38}N_2O_8$: [M + H]$^+$ m/z: 590.26, found 590.66.

(1R,2E,6R,10E,11aS,13S,14aR)-6,13-dihydroxy-4-oxo-4,6,7,8,9,11a,12,13,14,14a-decahydro-1H-cyclopenta[f][1]oxacyclotridecin-1-yl 3-acetyl-4-(2-(isoquinolin-2(1H)-yl)ethoxy)benzoate (**3DPQ-7**). Pale brown solid (16.08 mg, 85%). ^{1}H NMR (500 MHz, DMSO-*d*$_6$) δ 8.58 (s, 44H), 8.12 (s, 45H), 7.32 (s, 42H), 7.30 – 7.12 (m, 121H), 6.98 (dd, *J* = 115.0, 105.8 Hz, 205H), 6.67 (s, 45H), 6.67 (s, 48H), 6.04 (s, 46H), 5.50 (s, 8H), 5.52 – 5.17 (m, 141H), 5.15 (s, 41H), 4.95 (s, 45H), 4.50 (s, 24H), 4.43 (s, 66H), 4.21 – 4.05 (m, 89H), 3.77 (s, 45H), 3.61 (s, 46H), 3.47 (s, 46H), 2.71 (s, 48H), 2.58 – 2.40 (m, 401H), 2.29 – 2.01 (m, 150H), 2.03 (s, 61H), 2.02 (d, *J* = 12.6 Hz, 78H), 1.96 – 1.62 (m, 269H), 1.57 (s, 46H), 1.57 (s, 34H), 1.52 (d, *J* = 49.0 Hz, 105H). ^{13}C NMR (125 MHz, DMSO-*d*$_6$) δ 200.54 (s), 166.89 (s), 165.46 (s), 161.47 (s), 143.04 (s), 140.38 (s), 134.56 (s), 133.09 (d, *J* = 20.1 Hz), 132.97 – 132.81 (m), 132.42 (s), 131.22 (s), 130.82 (s), 128.16 (s), 127.47 (s), 127.21 (s), 126.58 (s), 126.30 (s), 125.59 (s), 121.22 (s), 115.21 (s), 102.97 (s), 79.97 (s), 73.67 (s), 67.17 (s), 51.86 (s), 50.60 (s), 48.76 (s), 47.11 (s), 41.34 (s), 40.17 – 39.96 (m), 38.11 (s), 34.91 (s), 32.41 (s), 28.28 (s), 22.77 (s). ^{15}N NMR (51 MHz, DMSO-*d*$_6$) δ -287.10 (s). ^{17}O NMR (68 MHz, DMSO-*d*$_6$) δ 488.00 (s), 338.90 (s), 332.00 (s), 130.60 (s), 78.00 (s), 44.50 (s), 33.40 (s), 13.00 (s). MS m/z calcd. for $C_{35}H_{39}NO_8$: [M + H]$^+$ m/z: 601.27, found 601.69.

(1R,2E,6R,10E,11aS,13S,14aR)-6,13-dihydroxy-4-oxo-4,6,7,8,9,11a,12,13,14,14a-decahydro-1H-cyclopenta[f][1]oxacyclotridecin-1-yl 3-acetyl-4-(2-(octahydro-1,8-naphthyridin-1(2H)-yl)ethoxy)benzoate (**3DPQ-8**). Whitish solid (16.33 mg, 85%). ^{1}H NMR (500 MHz, DMSO-*d*$_6$) δ 8.46 (s, 3H), 8.10 (s, 3H), 7.08 (d, *J* = 11.1 Hz, 6H), 6.67 (s, 3H), 6.19 (s, 3H), 6.04 (s, 3H), 5.48 (s, 3H), 5.35 (s, 3H), 5.15 (s, 3H), 4.12 – 3.93 (m, 9H),

3.61 (s, 2H), 2.99 (s, 3H), 2.86 (s, 3H), 2.72 (s, 3H), 2.64 (d, *J* = 6.2 Hz, 9H), 2.52 – 2.42 (m, 24H), 2.36 (d, *J* = 31.9 Hz, 6H), 2.21 (d, *J* = 47.9 Hz, 6H), 2.12 (s, 1H), 2.09 (s, 3H), 2.02 (d, *J* = 12.3 Hz, 5H), 1.86 (dd, *J* = 78.9, 6.1 Hz, 10H), 1.78 (d, *J* = 2.3 Hz, 3H), 1.80 – 1.59 (m, 17H), 1.80 – 1.36 (m, 37H), 1.19 (d, *J* = 5.5 Hz, 6H). ^{13}C NMR (125 MHz, DMSO-*d*$_6$) δ 200.54 (s), 166.89 (s), 165.46 (s), 161.47 (s), 143.04 (s), 134.56 (s), 133.17 (s), 131.22 (s), 130.82 (s), 127.47 (s), 126.30 (s), 121.22 (s), 115.21 (s), 79.97 (s), 73.67 (s), 66.80 (s), 65.38 (s), 52.88 (s), 50.51 (s), 48.76 (s), 47.11 (s), 43.97 (s), 41.34 (s), 40.17 – 39.96 (m), 39.03 (s), 38.11 (s), 34.91 (s), 32.41 (s), 28.28 (s), 27.79 (d, *J* = 0.5 Hz), 24.54 (s), 23.25 (s), 22.77 (s). ^{15}N NMR (51 MHz, DMSO-*d*$_6$) δ -326.40 (s), -334.90 (s). ^{17}O NMR (68 MHz, DMSO-*d*$_6$) δ 488.00 (s), 338.90 (s), 332.00 (s), 130.60 (s), 78.00 (s), 44.50 (s), 33.40 (s), 13.00 (s). MS m/z calcd. for $C_{34}H_{46}N_2O_8$: [M + H]$^+$ m/z: 610.33, found 610.74.

1-(2-(2-acetyl-4-((((1R,2E,6R,10E,11aS,13S,14aR)-6,13-dihydroxy-4-oxo-4,6,7,8,9,11a,12,13,14,14a-decahydro-1H-cyclopenta[f][1]oxacyclotridecin-1-yl)oxy)carbonyl)phenoxy)ethyl)-6-oxo-1,2,5,6-tetrahydropyridine-3-carboxylic acid (**3DPQ-9**). Whitish solid (16.54 mg, 82%). ^{1}H NMR (500 MHz, DMSO-*d*$_6$) δ 19.38 (s, 4H), 8.49 (s, 4H), 8.16 (s, 4H), 7.42 (s, 4H), 7.26 (s, 5H), 7.22 (s, 1H), 7.07 (s, 6H), 6.67 (s, 4H), 6.04 (s, 4H), 5.48 (s, 3H), 5.39 (s, 4H), 5.15 (s, 4H), 4.60 (s, 4H), 4.23 (s, 4H), 4.13 (s, 1H), 4.13 – 3.97 (m, 12H), 3.70 (s, 5H), 3.61 (s, 3H), 3.28 (s, 4H), 2.94 – 2.76 (m, 8H), 2.57 – 2.45 (m, 24H), 2.45 – 2.31 (m, 13H), 2.16 (s, 4H), 2.16 (s, 4H), 2.12 (t, *J* = 17.5 Hz, 13H), 2.29 – 1.96 (m, 22H), 1.94 (d, *J* = 10.0 Hz, 6H), 1.96 – 1.35 (m, 28H), 1.64 – 1.35 (m, 13H), 1.64 – 1.31 (m, 13H). ^{13}C NMR (125 MHz, DMSO-*d*$_6$) δ 200.54 (s), 167.89 (s), 166.89 (s), 166.47 (s), 165.46 (s), 161.47 (s), 143.04 (s), 134.56 (s), 133.17 (s), 131.22 (s), 130.78 (d, *J* = 10.1 Hz), 127.47 (s), 126.30 (s), 124.96 (s), 121.22 (s), 115.21 (s), 79.97 (s), 73.67 (s), 66.30 (s), 48.76 (s), 47.11 (s), 46.03 (s), 42.77 (s), 41.34 (s), 40.17 – 39.96 (m), 38.11 (s), 34.91 (s), 33.68 (s), 32.41 (s), 28.28 (s), 22.77 (s). ^{15}N NMR (51 MHz, DMSO-*d*$_6$) δ -287.10 (s). ^{17}O NMR (68 MHz, DMSO-*d*$_6$) δ 488.00 (s), 346.00 (s), 338.90 (s), 337.20 (s), 332.00 (s), 253.90 (s), 130.60 (s), 78.00 (s), 44.50 (s), 33.40 (s), 13.00 (s). MS m/z calcd. for $C_{26}H_{31}NO_{10}$: [M + H]$^+$ m/z: 517.19, found 517.53.

(1R,2E,6R,10E,11aS,13S,14aR)-6,13-dihydroxy-4-oxo-4,6,7,8,9,11a,12,13,14,14a-decahydro-1H-cyclopenta[f][1]oxacyclotridecin-1-yl 3-acetyl-4-(2-(piperidin-1-yl)ethoxy)benzoate (**3DPQ-10**). Whitish solid (14.79 mg, 85%). δ 8.34 (s, 5H), 7.89 (s, 5H), 7.19 (s, 4H), 7.07 (s, 6H), 6.67 (s, 5H), 6.04 (s, 5H), 5.44 (d, *J* = 43.8 Hz, 9H), 5.15 (s, 5H), 4.16 – 3.98 (m, 15H), 3.62 (d, *J* = 8.5 Hz, 10H), 2.79 – 2.57 (m, 21H), 2.57 – 2.40 (m, 45H), 2.13 – 1.75 (m, 38H), 2.31 – 1.22 (m, 105H), 1.78 (s, 6H), 1.78 (s, 6H), 2.13 – 1.22 (m, 87H), 1.83 – 1.41 (m, 54H). ^{13}C NMR (125 MHz, DMSO-*d*$_6$) δ 200.54 (s), 166.89 (s), 165.46 (s), 161.47 (s), 143.04 (s), 134.56 (s), 133.17 (s), 131.22 (s), 130.82 (s), 127.47 (s), 126.30 (s), 121.22 (s), 115.21 (s), 79.97 (s), 73.67 (s), 67.45 (s), 55.19 – 54.80 (m), 54.80 – 54.60 (m), 48.76 (s), 47.11 (s), 41.34 (s), 40.17 – 39.96 (m), 38.11 (s), 34.91 (s), 32.41 (s), 28.28 (s), 24.68 – 24.37 (m), 23.42 (s), 22.77 (s). ^{15}N NMR (51 MHz, DMSO-*d*$_6$) δ -327.80 (s).^{17}O NMR (68 MHz, DMSO-*d*$_6$) δ 488.00 (s), 338.90 (s), 332.00 (s), 130.60 (s), 78.00 (s), 44.50 (s), 33.40 (s), 13.00 (s). MS m/z calcd. for $C_{31}H_{41}NO_8$: [M + H]$^+$ m/z: 555.28, found 555.66.

(1R,2E,6R,10E,11aS,13S,14aR)-6,13-dihydroxy-4-oxo-4,6,7,8,9,11a,12,13,14,14a-decahydro-1H-cyclopenta[f][1]oxacyclotridecin-1-yl 3-acetyl-4-(2-(pyrrolidin-1-yl)ethoxy)benzoate (**3DPQ-11**). Whitish solid (14.39 mg, 85%). ^{1}H NMR (500 MHz, DMSO-*d*$_6$) δ 8.55 (s, 7H), 8.12 (s, 7H), 7.24 (s, 7H), 7.07 (s, 7H), 6.67 (s, 7H), 6.04 (s, 7H), 5.48 (s, 7H), 5.26 (s, 1H), 5.19 (d, *J* = 42.4 Hz, 13H), 4.04 (s, 8H), 3.98 – 3.94 (m, 11H), 3.64 (d, *J* = 26.0 Hz, 13H), 3.58 (s, 1H), 2.88 – 2.68 (m, 28H), 2.58 – 2.40 (m, 62H), 2.38 – 2.16 (m, 17H), 2.16 (s, 3H), 2.11 (d, *J* = 3.4 Hz, 3H), 2.13 – 1.77 (m, 48H), 1.78 (s, 5H), 1.85 – 1.56 (m, 52H), 1.47 (s, 11H). ^{13}C NMR (125 MHz, DMSO-*d*$_6$) δ 200.54 (s), 166.89 (s), 165.46 (s), 161.47 (s), 143.04 (s), 134.56 (s), 133.17 (s), 131.22 (s), 130.82 (s), 127.47 (s), 126.30 (s), 121.22 (s), 115.21 (s), 79.97 (s), 73.67 (s), 67.45 (s), 54.92 (s), 54.26 – 53.95 (m), 48.76 (s), 47.11 (s), 41.34 (s), 40.17 – 39.96 (m), 38.11 (s), 34.91 (s), 32.41 (s), 28.28 (s), 25.02 – 24.81 (m), 22.77 (s). ^{15}N NMR (51 MHz, DMSO-*d*$_6$) δ -315.50 (s). ^{17}O NMR (68 MHz, DMSO-*d*$_6$) δ 488.00 (s), 338.90 (s), 332.00 (s), 130.60 (s), 78.00 (s), 44.50 (s), 33.40 (s), 13.00 (s). MS m/z calcd. for $C_{30}H_{39}NO_8$: [M + H]$^+$ m/z: 541.27, found 541.63.

(1R,2E,6R,10E,11aS,13S,14aR)-6,13-dihydroxy-4-oxo-4,6,7,8,9,11a,12,13,14,14a-decahydro-1H-cyclopenta[f][1]oxacyclotridecin-1-yl 3-acetyl-4-(2-(3-hydroxy-2-oxopiperidin-1-yl)ethoxy)benzoate (**3DPQ-12**).

Whitish solid (13.28 mg, 85%). δ 8.55 (s, 48H), 8.09 (s, 48H), 7.21 (s, 5H), 7.13 (d, *J* = 62.9 Hz, 93H), 6.67 (s, 48H), 6.04 (s, 49H), 5.93 (s, 49H), 5.48 (s, 50H), 5.19 (d, *J* = 39.1 Hz, 95H), 4.44 (s, 49H), 4.20 – 4.16 (m, 77H), 3.98 (s, 49H), 3.79 (s, 39H), 3.62 (d, *J* = 8.5 Hz, 88H), 3.32 (s, 42H), 3.24 (d, *J* = 1.4 Hz, 107H), 2.61 – 2.43 (m, 432H), 2.17 (d, *J* = 13.8 Hz, 93H), 2.12 (d, *J* = 6.8 Hz, 17H), 2.13 – 1.77 (m, 359H), 1.77 (s, 33H), 1.72 – 1.64 (m, 124H), 1.64 – 1.48 (m, 120H), 1.47 (s, 41H). ^{13}C NMR (125 MHz, DMSO-d_6) δ 200.54 (s), 175.72 (s), 166.89 (s), 165.46 (s), 161.47 (s), 143.04 (s), 134.56 (s), 133.17 (s), 131.22 (s), 130.82 (s), 127.47 (s), 126.30 (s), 121.22 (s), 115.21 (s), 79.97 (s), 73.67 (s), 67.87 (s), 66.30 (s), 48.76 (s), 47.11 (s), 45.68 (d, *J* = 10.7 Hz), 41.34 (s), 40.17 – 39.96 (m), 38.11 (s), 34.91 (s), 32.41 (s), 28.28 (s), 26.41 (s), 22.77 (s), 22.44 (s). ^{15}N NMR (51 MHz, DMSO-d_6) δ -257.80 (s). ^{17}O NMR (68 MHz, DMSO-d_6) δ 488.00 (s), 346.00 (s), 338.90 (s), 332.00 (s), 130.60 (s), 78.00 (s), 44.50 (s), 33.40 (s), 32.30 (s), 13.00 (s). MS m/z calcd. for $C_{31}H_{39}NO_{10}$: $[M + H]^+$ m/z: 585.26, found 585.64.

Synthesized Compounds Antagonistic Potency and Relative Binding Affinities to ERα And ERβ [128,129]

The recombinant ERα and ERβ (Thermo Fisher Scientific Inc., Invitrogen, USA) and the fluorescent estradiol (Estradiol Glow, Jena Bioscience) were removed from the -80°C freezer and thawed on ice for 1 h before use. The fluorescent estradiol was added to either ERα or ERβ and screening buffer (ES2 Screening Buffer, Invitrogen, USA) was added to make the 100 to 0.01 nM range of concentrations (using 10-fold dilutions) for fluorescent hormone and 30 nM for ERα or ERβ. The 4-hydroxytamoxifen (Sigma Aldrich, CAS No 68047-06-3,) and raloxifene (Sigma Aldrich, CAS No. 82640-04-8), as the positive controls alongside with the synthesized compounds were accurately weighed and dissolved in DMSO, screening buffer was added to dilute to required concentration (100 to 0.01 nM, using 10-fold dilutions). Either positive control or synthesized compound (1 mL) was added to 49 mL screening buffer in each well (384-well microplate, Corning, USA). To this 50 mL of the fluorescent estrogen/ERα(β) complex was added to make up a final volume of 100 mL. The negative control contained 50 mL screening buffer and 50 mL fluorescent estrogen/ER complex. The negative control was used to determine the polarization value when no competitor was present (theoretical maximum polarization). The microplate was incubated in the dark at room temperature for 2 h and shaken on a plate shaker. The polarization values were read on a Safire microplate reader and used to calculate the IC_{50} values. To calculate the IC_{50} values, the percentage of estrogen receptor α antagonism was plotted against the concentration of either substrate, positive controls, and synthesized compounds. Following, the relative binding affinities (RBAs),[77] with the RBA of estradiol set to 100%, were calculated by using the following formula (SI eq. 2):

$$RBA = \frac{AC_{50}\, for\ estradiol}{AC_{50}\, for\ antagonist}\ x\ 100 \qquad \text{(SI eq. 2)}$$

The values given are the average ± range or SD of three independent determinations. The K_a (*i.e.* the K_i) values of each antagonist for each receptor were obtained from the RBA values by using the formula (SI eq. 3):

$$K_a = \frac{100}{RBA}\ x\ K_d \qquad \text{(SI eq. 3)}$$

where K_d is a dissociation constant of either **E**$_2$-ERα or **E**$_2$-ERβ complex. Estradiol binds to ERα with a K_d of 0.2 nM and ERβ with a K_d of 0.5 nM.

Synthesized Compounds Antiproliferative Activity against ERα(+)- and ERα(-)-Dependent Breast Cancer Cell Lines [130]

The antiproliferative activities of the compounds in various cell lines were determined using the MTT reduction assay. The human breast cancer MCF-7 and MDA-MB231 cell lines, as well as the normal human MRC-5 cell lines, have been purchased from American Type Culture Collection (Manassas, VA,

USA). Cells were routinely cultivated in DMEM (Dulbecco's Modified Eagle's Medium, Sigma Aldrich), supplemented with 10% fetal bovine serum (FBS. Sigma Aldrich), 100 U/mL Penicillin, and 100 mg/mL Streptomycin. Cells have been trypsinized and seeded in the volume of 100 mL/well at a density of 2.5 x 10^3 cells per well into 96-well microculture plates (ThermoFisher Scientific) and incubated for 24 h at 37°C (95% humidity and 5% CO_2) in an IncuSafe CO_2 incubator (PHC Europe B.V., Netherlands). Upon the incubation, cells were treated with a 100 μL/well medium containing the test compounds, by diluting E_1, positive controls, and synthesized compounds in the culture medium to the desired concentration (100 to 0.01 nM concentration range, 10-fold dilutions). Negative control was added to the equivalent volume of medium containing the 1% (v/v) DMSO (Sigma Aldrich, CAS No. 67-68-5). After 48 h of incubation, the media were removed and 100 μL of 0.5 mg mL^{-1} of 3-(4,5-dimethyl-2-thiazolyl)-2,5-diphenyl-2*H*-tetrazolium bromide (MTT, Sigma Aldrich, CAS No. 298-93-1) was added to each well and the plates were further incubated for 4 h. Supernatant from each well was carefully removed, formazan crystals were dissolved in 100 μL DMSO, and absorbance at a wavelength of 540 nm was recorded for the determination of IC_{50} values. All the experiments were performed during the exponential growth phase of the cell line

ERα Down-Regulation [15,74,131]

For this experiment, the MCF-7 cells were grown and treated with tested compounds same as the previous after which they were fixed using a 3.7% (v/v) final formaldehyde concentration for 20 min. The cells were washed three times with 250 μl phosphate-buffered saline (PBS) solution using a BioTek ELx405 plate washer (BioTek Instruments, Winooski, VT), and 50 μl of PBS was added to all wells. The PBS was aspirated, and the cells were permeabilized with 40 μl PBS containing 0.5% (v/v) Tween 20 for 1 h at room temperature. The cells were then washed three times in 250 μl of PBS/0.05% (v/v) Tween 20 (PBST) using a BioTek EL406 plate washer. Twenty microliters were added per well of a mixture of 1:1000 dilution of ERα [Clone SP1 (epitope contained within a synthetic peptide derived from C-terminal of human estrogen receptor)] monoclonal rabbit antibody (#RM-9101-S, Thermo Scientific) and a 1:1000 dilution of PR [Clone PgR 636 (epitope contained within recombinant full-length PR-A and hence specific for both PR-A and PR-B isoforms)] monoclonal mouse antibody (#M3569; Dako, Carpinteria, CA USA) in PBST/3% (w/v) bovine serum albumin (BSA), and the plates were incubated overnight at 4°C. The cells were washed three times in 250 μl of PBST and then incubated with 20 μL per well of a 1:500 dilution of goat antirabbit IgG Alexa Fluor 594 (Molecular Probes, Life Technologies, Paisley, UK), a 1:500 dilution of goat antimouse Alexa Fluor 488 antibody (Molecular Probes), and a 1:5000 dilution of Hoechst (Molecular Probes) in PBST/3% (w/v) BSA for 1 h at room temperature. The cells were washed three times in 250 μl of PBST, 40 μL of PBS was then added per well, and the plates were covered with a black plate seal. Cell images were acquired using a Cellomics Arrayscan VTI HCS reader (Thermo Scientific) using a 10× 0.3NA objective and applying the filters BGFR_386_23 and BGFR_549_15 to detect Hoechst and Alexa Fluor 594 signals. Using the Compartmental Analysis v4 bio-application, nuclear masks were generated from the Hoechst stained nuclei in channel 1, and a suitable threshold was applied in channel 2 to quantify ERα within the nuclear masks. The mean total intensity was normalized for cell number, giving the total intensity of ERα staining per cell. To determine the PR-associated signal, the plates were measured using an Acumen Explorer eX3 microplate cytometer (TTP LabTech, Melbourn, UK). Following excitation at 405 nm and 488 nm, cell object fluorescence and PR-associated fluorescence were detected at the two emission wavelength ranges of 420–500 and 500–530, respectively. The data were normalized for cell number by calculating a ratio of cell objects to fluorescence intensity.

ERα Functional Antagonism Cell Assay [15,74,131]

The ERα regulated gene expression of PR was measured as a biomarker for ERα antagonism and agonism. MCF-7 cells were plated in reduced serum media in a 384 well assay plate the day prior to the

assay. Antagonism: cells were pretreated for 30 min with 0.1 nM **E**$_2$ followed by a concentration range of each test compound (100 to 0.01 nM concentration range, 10-fold dilutions) for 24 h before being fixed and immunostained for PR. Agonism: cells were treated with a concentration range of each test compound (100 to 0.01 nM concentration range, 10-fold dilutions) for 24 h before being fixed and immunostained for PR. The PR levels were detected using a laser scanning imaging Acumen Explorer eX3 microplate cytometer.

The Impact of Targeted ERα Antagonists on the MCF-7 Cells Signaling [132-135]

Antibodies, drugs, and western analysis.

Antibodies were obtained from the following companies: anti-p53 (DO-1), -ERα (HC-20), -p21, -SMRT, and normal rabbit and mouse serum from Santa Cruz; anti-NCoR and -HDAC1 from Upstate Biotechnology; and anti-α-tubulin and β-actin (A2066) from Sigma-Aldrich. Cell lysates, saturated with the examined compounds in two concentrations encircling the IC_{50} values against MCF-7 cells (Table 10): all the compounds, except **3DPQ-5**, **3DPQ-6**, and **3DPQ-8**, have been re-administered in 0.1 and 1 nM dosage; for the marked compounds, the concentrations were 1 and 10 nM, were analyzed on SDS/PAGE gels, followed by western blotting with antibodies against various proteins, as noted in the figure legends. Specific proteins were detected by the enhanced chemiluminescence method (Amersham Biosciences).

qPCR.

Total cellular RNA was isolated using the Absolutely RNA Miniprep kit (Stratagene). Up to 1 μg of total RNA from individual samples was reverse transcribed in 20 μL of reaction using the iScript cDNA synthesis kit (Bio-Rad). One microliter of the resulting cDNA was used in a total volume of 25 μL of PCR; qPCR was performed on an ABI Prism 7300 sequence detection system with SYBR Green PCR Master Mix (Bio-Rad) using specific primers. Relative mRNA levels or DNA levels (in the case of qChIP) were calculated using the ΔΔCt method. For mRNA analysis, β-actin mRNA was used as an endogenous control. p53 binding to the p21 promoter was used to normalize q-ChIP. The following primers were used for experiments performed on human cells:

BTG2 forward (159–179): 5′ GTG AGC GAG CAG AGG CTT AAG 3′
BTG2 reverse (259–242): 5′ GAG CCC TTG GAC GGC TTT 3′
PUMA forward (1442–1461): 5′ ATG CCT GCC TCA CCT TCA TC 3′
PUMA reverse (1566–1544): 5′ TCA CAC GTC GCT CTC TCT AAA CC 3′
BAX forward (290–310): 5′ TGG AGC TGC AGA GGA TGA TTG 3′
BAX reverse (390–370): 5′ CCA GTT GAA GTT GCC GTC AGA 3′
DR5 forward (1747–1766): 5′ CCC AGT GGA TGG AAC ATC CT 3′
DR5 reverse (1854–1833): 5′ CAC AAA CGG AAT GAT CCA GAC A 3′
GADD45A forward (434–453): 5′ CTC AAC GTC GAC CCC GAT AA 3′
GAD45A reverse (534–515): 5′ GCC TGG ATC AGG GTG AAG TG 3′
Actin forward (212–234): 5′ ATG GGT CAG AAG GAT TCC TAT GT 3′
Actin reverse (453–432): 5′ AAG GTC TCA AAC ATG ATC TGG G 3′

ChIP, qChIP, and sequential ChIP

ChIP assays were performed on MCF-7 and Saos2 cells using the ChIP kit from Upstate Biotechnology as per the manufacturer's instructions with minor modifications. Chromatin was cross-linked using 1.5% formaldehyde for 10 min at 37 °C, and the cells were collected after two washings with PBS-containing protease inhibitor mixture. For qChIP, DNA in the final step was analyzed by qPCR. The following primers were used for analysis of ChIP DNA by real-time PCR and semiquantitative PCR:

PCNA, p53 site, forward (−284 to −265): 5′ CCA CCA TAA AGC TGG GGC TT 3′

PCNA, p53 site, reverse (−182 to −201): 5′ TCT CCC CGC CTC TTT GAC TC 3′
p21, 5′ p53 site, forward (−2313 to −2293): 5′ GCT GTG GCT CTG ATT GGC TTT 3′
p21, 5′ p53 site, reverse (−2212 to −2231): 5′ ACA GGC AGC CCA AGG ACA AA 3′
p21, 3′ p53 site, forward (−1452 to −1432): 5′ CAT CCC CAC AGC AGA GGA GAA 3′
p21, 3′ p53 site, reverse (−1310 to −1329): 5′ACC CAG GCT TGG AGC AGC TA 3′
p21, NS site, forward (−4443 to −4422): 5′ GAG TCC TGT TTG CTT CTG GGC A 3′
p21, NS site, reverse (−4199 to −4220): 5′ CTG CAT TGG GGC TGC CTA TGT A 3′

For sequential ChIP assays, cell lysates were initially incubated with anti-p53 antibody, and the immunocomplexes were eluted at 37 °C for 30 min in rechip buffer (1% Triton X-100, 2 mM EDTA, 150 mM NaCl, 20 mM Tris-HCl, pH 8.1), followed by incubation with anti-ERα antibody. Secondary immunocomplexes were eluted in rechip buffer and incubated with antibodies against NCoR, SMRT, RIP140, and HDAC1. Final immunocomplexes were eluted in elution buffer (500 μL 1% SDS, 0.1 M $NaHCO_3$) and processed for DNA analysis.

Effects of Synthesized Compounds on Cytotoxicity and Cell Cycle Distribution in ERα(+)Dependent Breast Cancer Cell Lines [130]

For all the considered compounds, except **3DPQ-5, 3DPQ-6, and 3DPQ-8**, the MCF-7 cells were treated with 0.1 and 1 nM dosage for 24 h, thus covering the range of in which the IC_{50} values were found; for the marked compounds, the concentrations were 1 and 10 nM. After treatment, the cells were washed twice with ice-cold PBS (Gibco, Cat. no. 20012), collected by centrifugation, and fixed in ice-cold 70% (v/v) ethanol, washed with PBS, resuspended with 0.1 mg/mL RNase (Biocompare, San Francisco California, USA) stained with 40 mg/mL PI (Sigma Aldrich, CAS No. 25535-16-4), and analyzed by flow cytometry using FACScalibur (Becton Dickinson). The cell cycle distributions were calculated using Flowjo 7.6.1 software.

Determination of Lipophilicity [75,156]

The $logD_{7.4}$ values were experimentally obtained by the shake-flask method. A calibration graph was plotted using five different concentrations of compound (5, 10, 20, 30, 40, and 50 mg/kg of bwt) in *n*-octanol. Then, each of the applied concentrations of tested compounds was dissolved in 100 μL of DMSO and diluted with *n*-octanol to 25 mL in a volumetric flask. From this solution, 10 mL was diluted with *n*-octanol to 10 mL. The absorbance of the compounds in these *n*-octanol solutions was measured by UV–Vis spectrophotometry. The biphasic system containing 4 mL of previously prepared *n*-octanol solutions and 8 mL of phosphate buffer (pH = 7.4) was shaken on a mechanical shaker for 30 min. After complete phase separation, the *n*-octanol layer was dried over anhydrous sodium sulfate and absorbance was measured. The concentration was calculated from the calibration graph and the $logD_{7.4}$ value was determined using the SM eq. 4:

$$logD_{7.4} = \log(\frac{y}{x-y}) \qquad \text{(SM eq. 4)}$$

where x represents the concentration of the compound in the *n*-octanol phase before shaking and y represents the concentration of the compound in the *n*-octanol phase after the treatment on a mechanical shaker. For each compound, five independent measurements were performed.

In Vivo Anticancer Screening [146]

Female virgin Wistar rats were obtained from the Military Medical Academy of the University of Defense, University of Belgrade, Serbia, at 35 days of age. All the animal procedures were approved by the Committee for Ethical Animal Care and Use of the Institute for Biological Research, Belgrade, which

acts following the Guide for the Care and Use of Laboratory Animals, published by the US National Institute of Health (NIH Publication No. 85/23, revised in 1986). Rats were housed at 3 per cage and maintained at 25±2°C under a 12 h dark/light cycle with access to a standard diet and water *ad libitum*. The breast cancer was induced after the MNU (Sigma Aldrich CAS No. 684-93-5) administration. An aqueous solution at a concentration of 10 mg/ml was made by wetting the NMU powder with 3% acetic acid and then dissolving it in 0.9% NaCl solution; a fresh solution was prepared for each injection. Rats were given *intraperitoneal (i.p.)* 50 mg/kg of MNU on the 50th day of age. Animals were divided into different groups with six animals in each group. Group I (intact control) received 0.9% NaCl solution. Groups II-IV and V-VII were introduced with MNU. Two weeks after MNU treatment, animals were treated orally with synthesized compounds and control substances (5 and 50 mg/kg, for groups II-IV and V-VII, respectively) by gavage once per day for six weeks. Animals in the intact control group and untreated MNU group were given vehicle (Tween 80) according to the experimental protocol. After MNU treatment the animals were palpated weekly to detect the presence of mammary tumors. The time of appearance of the first tumor (latency period), the number of tumors/rat (tumor burden), and the relative size of every tumor were recorded. The tumor diameter was measured by micrometer caliper, and volume was calculated using the formula (SI eq. 5):

$$V = \frac{4}{3}\pi r^3 \qquad \text{(SI eq. 5)}$$

where r is half of the average diameter. Upon finishing the treatment with synthesized compounds, animals were sacrificed, and plasma, serum, and liver samples were collected [147].

Measurement of Serum Biochemical Markers [159]

The activities of AST and ALT on 340 nm, and ALP on 405 nm, were determined by UV-Vis kinetic methods according to recommendations of the Expert Panel of the IFCC (International Federation of Clinical Chemistry) [148,149]. The colorimetric methods were used for the determination of total bilirubin (Jendrassik-Grof 550 nm method) [150,151]. All kinetic and colorimetric measurements were recorded using a Perkin-Elmer Lambda 25 UV/Vis spectrophotometer.

Determination of Antioxidant Markers in Liver Homogenate [159]

Rat liver samples were homogenized in phosphate buffer (5 mM, pH = 7.4) to give a 10% (w/v) liver homogenate and then centrifuged at 4000 rpm for 15 min at 4°C. The supernatant of liver homogenate was used for the estimation of reduced glutathione (GSH) [152] and catalase (CAT) [153] levels by the colorimetric method. The contents of malondialdehyde (MDA) [154] in the supernatant of the liver homogenates were determined using 1,1,3,3-tetraethoxypropane as standard. Total protein concentrations were determined according to the method of Lowry [155] using bovine serum albumin as standard. All colorimetric measurements were recorded using a Perkin-Elmer Lambda 25 UV/Vis spectrophotometer.

Plasma Protein Binding Determination [157]

Rat plasma containing either 5 or 50 mg/kg bwt of the tested compounds was incubated at 37°C for 2 h. Following incubation, 400 μl samples were transferred in triplicate at each concentration to the upper reservoir of the washed Centri-free YM-30 ultrafiltration units (Millipore, Bedford, MA) and then centrifuged for 20 min at 2,000*g*. Aliquots of the ultrafiltrates and unfiltered incubation mixtures were analyzed for the total protein concentration by the bicinchoninic acid assay for protein concentration (BCA assay; Pierce, Rockford, IL).

Determination of the Intrinsic Clearance of Hepatocytes [158]

Preparation of rat hepatocytes.

Isolation of rat hepatocytes was performed essentially using the two-step *in situ* collagenase perfusion method. Briefly, the hepatic portal vein of an anesthetized male Wistar rat (weight 200 –300 g) was cannulated just above the junction of the splenic and pyloric veins. Liver perfusion medium (Invitrogen, Paisley, UK) was perfused *via* the hepatic portal vein until the liver cleared to an even tan color (usually 7– 8 min at a perfusion rate of 30 mL/min). Liver digestion medium (Invitrogen) was then perfused until the liver displayed evidence of extensive dissociation (usually a further 6 – 8 min at a perfusion rate of 30 mL/min). The liver was dissected from the rat, and cells were gently teased out of the liver capsule into a beaker containing ice-cold hepatocyte suspension buffer [2.34 g of sodium HEPES, 0.4 g of D-fructose, 2.0 g of bovine serum albumin (BSA), 1-liter powder equivalent of Dulbecco's modified Eagle's medium (Sigma, Gillingham, UK] diluted in 1 liter of water and adjusted to pH 7.4 with 1 M HCl). The cell suspension was passed through a 250-μm mesh into a precooled tube and centrifuged at 50g for 2 min at 4°C. The supernatant was decanted, the cell pellet was resuspended in suspension buffer (without BSA), and the centrifugation step was repeated. The resulting pellet of cells was resuspended in 10 mL of suspension buffer (without BSA), and estimation of hepatocyte yield and viability was obtained using the trypan blue exclusion method. Only cells with a viability of 80% were used.

Determination of metabolic CL_{int} using rat hepatocytes.

NCE stocks were prepared in dimethyl sulfoxide at 100-fold incubation concentration (100 μM), and 10 μL of this 100 μM stock was added to a vial containing 490 μL of hepatocyte suspension buffer (without serum). A vial containing rat hepatocytes at a concentration of 2 million viable cells/mL was preincubated for 5 min in a shaking (80 oscillations/min) water bath at 37°C along with the vial containing the drug-buffer mix. Reactions were initiated by adding 500 μL of hepatocyte suspension to the 500 μL of drug/buffer mix. Aliquots (40 μL) were removed at 0, 2, 6, 15, 30, 45, 60, and 90 min, and reactions were quenched in 120 μL of ice-cold methanol. Samples were subsequently frozen for 1 h at 20°C and then centrifuged at 2000g for 20 min at 4°C. The supernatants were removed and analyzed as described below. CL_{int} was estimated using SI eq. 6:

$$CL_{int} = V\ x\ k \qquad \text{(SI eq. 6)}$$

where V is the incubation volume and k is the elimination rate constant. The unbound drug intrinsic clearance *in vivo* (CL_{int} *in vivo*) was calculated from hepatic blood clearance using the parallel tube model, as shown below (SI eq. 7):

$$CL_{int} = \frac{Q_h}{fu_b\ x \ln\frac{(Q_h - CL_h)}{Q_h}} \qquad \text{(SI eq. 7)}$$

where CL_h is hepatic blood clearance, fu_b is the fraction of drug unbound in blood, and Q_h is blood flow (70 mL/min/kg in the rat).

Pharmacokinetics Studies in Rats [158]

For the definitive oral bioavailability and pharmacokinetic study, male and female Wistar rats, jugular vein catheterized, n = 3 per treatment group, were administered a single dose of each compound by intravenous treatment (*iv*) (5 mg/kg; 6mg/m2) *via* the tail vein or oral gavage (5, or 50 mg/kg; 60, 180 mg/m^2) routes. The volumes administered were 5 mL/kg for iv and 5 mg/L *per os*. The rats have fasted overnight before compounds' administration. For *iv* treatment, compounds were was solubilized in DMSO:PEG300 (15:85) at 5 mg/mL and for oral gavage, it was formulated in PEG400:Labrasol (1:1, v:1) at concentrations of 0.5 and 5 mg/mL. Blood samples (~200 μL) were collected at multiple time points post-dose through 24 h from the jugular canula and transferred to tubes with EDTA to obtain whole

anticoagulated blood. Plasma (~200 μL of blood collected at the 2-h time point) was prepared by centrifugation at 2–8°C and stored frozen at approximately −70°C until analysis to determine the concentration of tested compounds.

Pharmacokinetic analysis was performed on whole blood drug concentration data for individual animals using non-compartmental methods and WinNonlin® Professional (Version 5.2, Pharsight, Mountain View, CA). Pharmacokinetic parameters, calculated for each animal, included observed maximal plasma concentration (Cp), extrapolated plasma concentration at time 0 (C_0; *iv* group only), area under the plasma concentration-time curve from time 0 to the last time point (AUC_{last}), and extrapolated to infinity ($AUCi_{nf}$), terminal elimination half-life ($t_{1/2}$), apparent volume of distribution (V), and total clearance (Cl). Mean residence time (MRT) was calculated by dividing the area under the first moment curve (AUMC) by the AUC. Bioavailability (F) was estimated using the following formula (SI eq. 8):

$$F = AUC_{\inf po}\, x\, \frac{Dose_{iv}}{AUC_{inv\ iv}}\, Dose_{po} \qquad \text{(SI eq. 8)}$$

Histopathological Studies [159]

The breast cancer samples, as well as the other organs samples slices were fixed with 10% formalin in phosphate-buffered saline for 24 h and embedded in paraffin. Sections 5 μm thick were made using a microtome, stained with haematoxylin-eosin, and observed under a microscope to determine histopathological changes. Photographs of each slide were taken at 200x magnification

Maximum-Tolerated-Dose (MTD) Study [160]

Female virgin Wistar rats were obtained from the Military Medical Academy of the University of Defense, University of Belgrade, Serbia, at 35 days of age. All the animal procedures were approved by the Committee for Ethical Animal Care and Use of the Institute for Biological Research, Belgrade, which acts following the Guide for the Care and Use of Laboratory Animals, published by the US National Institute of Health (NIH Publication No. 85/23, revised in 1986). Rats were housed at 3 per cage and maintained at 25±2°C under a 12 h dark/light cycle with access to a standard diet and water *ad libitum*. Rats were randomly assigned to vehicle-treated controls and tested compounds-treated dose groups as follows: one placebo-treated control group (vehicle alone; 2% carboxymethylcellulose) and four multiple-dose groups of tested compounds, **MNU**, **4-OHT**, and **Ral** (5, 50, 100, 500, 1000 mg/kg/day suspended in the vehicle). The rats (5 females per group) in these groups were orally administered the placebo or treatment once daily for 5 days. The animals were sacrificed after 5 days of treatment, and blood was analysed using the above-specified methods. Major organs (heart, lung, liver, kidney, spleen, intestine, brain) were histologically examined at necropsy.
